# Differential protein profiling as a potential multi-marker approach for TSE diagnosis

**DOI:** 10.1186/1471-2334-9-188

**Published:** 2009-11-27

**Authors:** Janice B Barr, Michael Watson, Mark W Head, James W Ironside, Nathan Harris, Caroline Hogarth, Janet R Fraser, Rona Barron

**Affiliations:** 1 The Roslin Institute & R(D)SVS, University of Edinburgh, Roslin, Midlothian, EH25 9PS, UK; 2 Institute for Animal Health, Compton, RG20 7NN, UK; 3 National CJD Surveillance Unit, University of Edinburgh, Western General Hospital, Crewe Road, Edinburgh, EH4 2XU, UK; 4 Molecular Sensing Inc, 1409 Main St, Montara, CA94037, USA; 5 Bio-rad Laboratories, Hercules, CA 94547, USA

## Abstract

**Background:**

Transmissible spongiform encephalopathy describes a family of diseases affecting both man and animals. Current tests for the diagnosis of these diseases are based on the detection of an abnormal misfolded form of the host protein PrP which is found within the central nervous and lymphoreticular systems of affected animals. Recently, concern that this marker may not be as reliable as previously thought, coupled with an urgentneed for a pre-clinical live animal test, has led to the search for alternative assays for the detection of TSE disease.

**Methods:**

This "proof of concept" study, examines the use of differential protein expression profiling using surface enhanced laser desorption and ionisationtime of flight mass spectrometry (SELDI-TOF) for the diagnosis of TSE disease. Spectral output from all proteins selectively captured from individual murine brain homogenate samples, are compared as "profiles" in groups of infected and non-infected animals. Differential protein expression between groups is thus highlighted and statistically significant protein "peaks" used to construct a panel of disease specific markers.

Studies at both terminal stages of disease and throughout the time course of disease have shown a disease specific protein profile or "disease fingerprint" which could be used to distinguish between groups of TSE infected and uninfected animals at an early time point of disease.

**Results:**

Our results show many differentially expressed proteins in diseased and control animals, some at early stages of disease. Three proteins identified by SELDI-TOF analysis were verified by immunohistochemistry in brain tissue sections. We demonstrate that by combining the most statistically significant changes in expression, a panel of markers can be constructed that can distinguish between TSE diseased and normal animals.

**Conclusion:**

Differential protein expression profiling has the potential to be used for the detection of disease in TSE infected animals. Having established that a "training set" of potential markers can be constructed, more work would be required to further test the specificity and sensitivity of the assay in a "testing set". Based on these promising results, further studies are being performed using blood samples from infected sheep to assess the potential use of SELDI-TOF as a pre-mortem blood based diagnostic.

## Background

Transmissible spongiform encephalopathies (TSEs) are a group of diseases affecting the central nervous and lymphoreticular system of both man (Creutzfeldt-Jakob disease (CJD))[1] and animals (e.g. bovine spongiform encephalopathy (BSE)[2], scrapie in sheep [3] and chronic wasting disease (CWD)[4] in deer). The diseases typically have a long asymptomatic stage before a rapid neurodegenerative stage leading to death. Definitive diagnosis has historically therefore, been restricted to post -mortem examination of brain tissue for the presence of pathological hallmarks of the disease such as spongiform changes and the deposition of an abnormal disease related form of the prion protein termed PrP^d^(^d^denotes a disease associated form of the host encoded protein PrP^c^) [5]. Following an outbreak of BSE in cattle which was subsequently linked to the emergence of a new human form of the disease, vCJD, commercial testing for surveillance purposes has focused on detection of this protein (PrP^d^) in brain tissue homogenates. Most assays, such as immunoassay, utilise the distinct protease resistance property of PrP^d ^by digesting samples with proteinase K, which increases the specificity and sensitivity of PrP^d ^detection. These assay systems [6] are only reliable if PrP^d ^always associates with TSE infectivity, however several models of disease have now been described in which transmission of disease was achieved from tissue with little or no detectable PrP^d ^[7-9]. In addition, atypical forms of sheep scrapie have recently been described in Europe [10] which are not conclusively identified by most of the current commercially available diagnostic assay systems due to differences in the degree or extent of PrP^d ^resistance to proteinase K. PrP^d ^is also difficult to detect in blood, and following the possible transmission of vCJD through the transfusion of blood or blood products in humans[11,12], there is now an urgency to seek new types of testing regimes which could be applied at a pre-clinical stage of disease in both man and animals. Several other candidate proteins have been proposed to be markers of TSE disease [13] however as single markers their reliability (with the exception of PrP^d^)has remained unproven.

The emerging field of proteomic technology offers the potential to discover new putative markers for diagnosis in many diseases. Assays are currently being developed for other diseases such as cancer [14,15], combining the strengths of proteomics with powerful bioinformatics tools, utilising multiple protein biomarkers in data driven predictive assays. The benefits of high throughput automated analysis with the potential of high sensitivity and specificity make this type of assay an attractive alternative to single marker assays. In this study we investigate a proteomic approach to establish a multi marker pattern recognition diagnostic assay using surface enhanced laser desorption and ionisation time of flight spectrometry (SELDI -TOF MS) and bioinformatics to identify differences in protein expression between TSE diseased and non-infected murine brain tissue samples, and create a mathematical model of disease (disease profile map). As it is the combined profile or pattern of protein expression differences which is applied as a mathematical algorithm, the identity of each component protein is not required. In contrast to other mass spectrometry approaches where the complexity of the proteome is addressed by pre-fractionation processes, in this technology the crude sample is applied directly to a specialised chromatographic surface which selectively binds subsets of proteins according to their biochemical properties.

Other proteomic and genetic human wide population studies have highlighted the need to restrict variables in sample sets, such as, genetic, gender, and age background, in order to achieve interpretable results. TSE disease research is further complicated by the long asymptomatic incubation associated with disease. Taking these factors into consideration we examined the feasibility of applying this novel multi-marker approach to TSE diagnosis using a well characterised experimental murine model of TSE disease, initially using brain tissue in these studies as the major pathology in TSE disease is confined almost without exception to the CNS. The experimental model used in these studies (CVF1 mouse model infected with an experimental TSE strain (scrapie isolate ME7) [16,17] is a highly reproducible model of TSE disease in an inbred mouse line and has been used extensively in studies within our laboratory. This model produces a severe and defined pathology in the CA1 region of the hippocampus providing the opportunity to study differential protein expression in an area of the brain which has the highest probability of discovering TSE disease specific differences. The establishment of a SELDI directed diagnostic panel in CNS could then be translated to large animal studies and extended in accessible tissues such as blood with the aim of developing a robust pre-mortem diagnostic.

## Methods

### Animals

All animal experiments were approved by the local Ethical Review Committee and performed under licence to the UK Home Office in accordance with the Animals (Scientific Procedures) Act 1986.

In the terminal study a group(n = 12) of ~10 ten week old male mice (F1 cross between C57Bl/Dk and VM/Dk ([18]) were intracerebrally injected (0.02 ml 1% (w/v)) with a brain suspension infected with ME7 scrapie isolate. A control group (n = 12) ten week old mice were similarly injected with a normal brain suspension. The time course experiment was set up in the same way as above with two groups of animals (n = 6NB n = 6ME7) which were serially culled at thirty day intervals. Animals were monitored for clinical signs of disease and on reaching the terminal stage of disease were culled by cervical dislocation. Brain samples were flash frozen and stored at -70°C. Microdissection of the brain was carried out as described in Barr et al (2004) [19].

### Sample preparation

Brain tissue samples were homogenised in buffer (100 mM HEPES pH7, 100 mM NaCl, 0.05% CHAPS) placed on ice for 30 minutes, centrifuged and the supernatant removed (S1). The remaining pellet was re-suspended in 90 μl buffer (5 M guanidine-HCl, 50 mM Tris pH8, 0.5% CHAPS) incubated on ice for 3 hours centrifuged and the supernatant removed (S2). Total protein was estimated using a Protein BCA kit (Pierce).

### SELDI -TOF analysis

Two array types were used for analysis, a strong anionic exchange surface (SAX/Q10), and a weak cationic exchange surface (WCX/CM10). Arrays were prepared in a bioprocessor by washing with appropriate buffer (CM10-100 mM ammonium acetate pH4.5 Q10-100 mM Tris pH8), and then 5 μg of each sample was applied to wells containing 90 μl buffer and incubated for 40 minutes at room temperature. After washing in buffer the arrays were dried and two applications of 0.8 μl matrix solutions (sinnapinic acid/50% acetonitrile/0.5% TFA) made to each spot on the array.

Arrays were analyzed on a PBSII ProteinChip^® ^reader (Ciphergen Biosystems, Feemont, CA.) using the accompanying software (ProteinChip^® ^Version 3.2), two scans of each sample optimized a range 3000-30,000 m/z and 25,000-100,000 m/z. Data was averaged over 300 transients on each spot and externally calibrated using a protein standard mixture (All-in-1 Protein Standard, Ciphergen Biosystems). All data collected from individual arrays used at the same settings were saved in a separate experimental files e.g. WCX low laser S1, SAX high laser S2. Data files were corrected by subtracting the background noise and normalized to the same total ion current. Peak identification and clustering was achieved using the Biomarker Wizard software (Ciphergen Biosystems). Settings for cluster formation were first pass S/N 5 in 20% of all spectra and second pass S/N 2. Cluster mass window was 0.3% of the mass. Single marker statistics were calculated using the non-parametric Mann-Whitney (*U *test) on the peak intensities.

### Statistical analysis

The statistical package *R *http://www.R-project.org was used to carry out statistical analysis of the data. For each supernatant/array type/laser combination the output from the Biomarker Wizard software was read into *R*. A two sample t-test was used to identify significant proteins which were then examined pair wise using scatterplots. The subset of significant proteins was used as an input to cluster analysis and principal components analysis.

### Protein purification and identification

Supernatants from infected (× 2) and uninfected (× 2) brain tissue samples were fractionated on minispin columns (Sigma-Aldrich) packed with 200 μl QHyperDF resin (Biosepra). Columns were washed sequentially and fractions eluted with 500 μl of buffers Tris-HCl pH9, HEPES pH7, NaAcetate, pH4 and 5 NaCitrate pH3 and finally an organic fraction with a solution of 33% isopropanol/0.1% TFA. Each fraction was spotted on a NP20 and CM10 array and analyzed on the ProteinChip^® ^reader to assess the presence of the protein peak of interest. The fractions pH3 and "flow through" displaying the presence of the peak of interest were further fractionated on a hydrophobic column (Pierce Pepclean C18) by sequentially washing with 10%, 40% and 70% acetonitrile/0.1%TFA. Again these fractions were spotted on a NP20 array and analyzed on the ProteinChip^® ^reader. The fractions with displaying the presence of the peak of interest were then run on an SDS-PAGE gel (Invitrogen NuPAGE system using 12% Bis-Tris Nu-PAGE gel). The gel was stained with Coomassie blue (R-250 Sigma-Aldrich). Two standard protein ladders (See-blue, Multimark, Invitrogen) were used to indicate the approximate mass weight of the bands and a bands at 10-12 kDa were excised by punching with a small diameter needle (Harris punch, Sigma-Aldrich) and placing in a micro centrifuge tube. Samples were frozen and kept at 70°C.

### In-gel digestion

The excised gel bands were treated to remove the Coomassie stain and SDS by incubating successively with 200 μl of 50% methanol/10% acetic acid for 30 minutes, 200 μl of 50% acetonitrile/100 mM ammonium bicarbonate (pH 8) for 30 minutes, and 100 μl acetonitrile for 10 minutes. The gel pieces were dried in a Speed-Vac. The dried gel pieces were rehydrated with 20 μl of 50 mM ammonium bicarbonate (pH8) containing 10 ng/μl modified trypsin (Roche Applied Science) and incubated for 16 hours at 37°C.

Protein identification by peptide fragmentation using a tandem mass spectrometer equipped with a PCI-1000 ProteinChip Interface Single MS and MS/MS spectra were acquired on a tandem mass spectrometer equipped with a Ciphergen PCI-1000 ProteinChip Interface. A 1 μl aliquot of each protease digest was spotted on a NP20 ProteinChip Array. 1 μl of saturated CHCA in 50% ACN, 0.5% TFA was immediately applied to the spot and the two solutions were mixed by pipeting. Spectra were collected from 800 Da to 3500 Da in single MS mode. After reviewing the spectra, specific ions were selected for MS/MS analysis. The CID spectra were submitted to the database-mining tool Mascot (Matrix Sciences) for identification of Cpn10 and FKBP12 (See additional file 1: Hsp10 ID and additional file 2: FKBP12 ID). For the identification of DBI spectra were collected directly on a ProteinChip Enterprise instrument and CID spectra submitted to Profound http://prowl.rockefeller.edu/prowl-cgi/profound.exe (See additional file 3: DBI ID).

### Immunocytochemistry and confocal microscopy

Coronally cut 6 μm paraffin embedded formol fixed brain sections from terminal animals (~260 dpi) were de-waxed, microwaved in citrate buffer quenched with methyl peroxide and blocked using normal goat serum. Sections were incubated for one hour with either the primary antibody (anti-Cpn10 rabbit polyclonal antibody, Stressgen Bioreagents, (SP110) 1:400 dilution, anti-DBI/ACBP rabbit polyclonal antibody, Santa Cruz Biotechnologies,1:200 dilution, anti- FKBP12 rabbit polyclonal Affinity Bioreagents PA1026A 1:500, and anti-Cpn60 mouse monoclonal, Stressgen Bioreagents,(BAF2584). Sections from animals inoculated with normal brain homogenate and sacrificed at the same time as terminal animals were similarly treated. Control sections to check for background interference were incubated as the other sections substituting the primary antibody with normal rabbit or mouse serum. Sections were washed and a biotinylated secondary antibody goat anti-rabbit for the polyclonal rabbit primaries or goat anti-mouse for mouse primary (Jackson Laboratories) applied at 1:600 dilution for one hour. Steptavidin was applied (Vector Labs. Inc. Vectastain ABC kit) followed by DAB for 4 minutes and counterstaining was achieved using heamatoxylin and Tapps reagent. For confocal analysis of Cpn10 the first steps of the protocol were followed then the fluorescent secondary antibody (Alexa 488 goat anti-rabbit, Molecular Probes) was applied for one hour, washed and mounted with fluorescent mounting medium (DAKO). Imaging was achieved using a Zeiss LSMS PASCAL confocal microscope with a FITC filter.

## Results

A series of experiments were carried out to assess the capability of the SELDI approach in distinguishing pathological changes both between different brain areas and between TSE infected and non infected animals. A small pilot study (no. = 2 infected, no. = 2 uninfected at terminal stage of disease (260 dpi)) examined if any differences between normal and diseased groups could be detected in particular brain areas (hippocampus and cerebellum) of the murine TSE model. The main studies focused on results in brain tissue samples from animals (no = 12 infected, no = 12 uninfected) clinically assessed to be at the terminal stage of disease (~260 days post injection) and from animals (no. = 6 infected, no. = 6 uninfected) culled at intervals of thirty days post injection (30, 60, 90, 120, 150, 180, 210, 240 and terminal) throughout the course of infection. Trials with fewer samples were used to establish standardised protocols. In all the experiments samples of brain tissue were microdissected [19] from areas of the brain (hippocampus and cerebellum) of groups of age matched (~10 weeks old) animals injected intracerebrally with either TSE (scrapie isolate, ME7) infected or normal brain homogenate inocula. We hypothesized that protein differences between normal and scrapie infected animals would be more apparent in the hippocampus where pathology is severe, compared with the cerebellum where there is no obvious pathology in this model. As described in the Methods section, supernatant solutions of homogenized samples, S1 (soluble proteins) and S2 (insoluble proteins) were spotted on two array types, weak cationic (WCX/CM10 ProteinChip^® ^Array) and strong anionic (Q10/SAX ProteinChip^® ^Array) exchange surfaces. For each brain therefore there were four samples, cerebellum S1, S2, hippocampus S1 and S2 on two different array surfaces which were designed to isolate different sub-groups of proteins from the proteome.

### Protein expression differences are detected in a brain area known to exhibit TSE specific pathology

**Figure 1:**
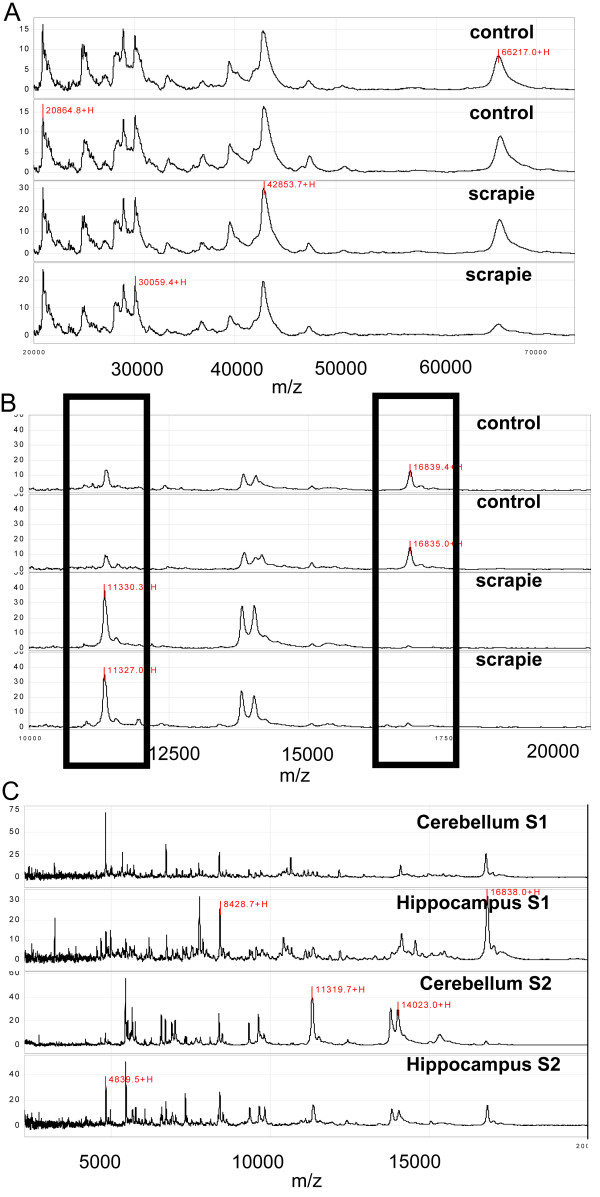
**Differential protein expression profiling spectra displayed by ProteinChip software**. Spectra of proteins expressed in scrapie infected (n = 2) and control (n = 2) brain homogenates from animals at the terminal stage of disease (~260 dpi) (A) displaying similar spectral peaks in all samples. (B) spectra as in (A) at a different part of the spectrum displaying differential expression at a lower molecular weight. (C) differential expression shown in different areas of the same brain.

In the pilot study, spectra from the same brain area in both the control and diseased groups displayed protein expression profile similarities (Figure 1A) and differences (Figure 1B). Expression within the same brain can be seen in Figure 1C, with clear differences between the cerebellum and the hippocampus. Differences between the supernatant extracts were also apparent. This confirmed that we had isolated different sub groups of proteins which were reproducible between similar sample types. Protein peaks found in all samples generated under the same conditions e.g. all cerebellum S1 samples on WCX array at a particular laser power, were then clustered (Biomarker Wizard™) and the peak intensities of diseased and control samples statistically compared (Mann-Witney). Clusters of statistically significant proteins at particular m/z ratios can be seen in Table 1. Using this method only two biomarkers in the cerebellum were revealed compared to thirty in the hippocampus, indicating that the differences observed in protein expression reflected the extent and severity of disease specific pathological change. If the markers were indicative of differences unrelated to scrapie infection we would have expected to find a more evenly distributed number of markers over both brain areas.

**Table 1:** Comparison of brain areas in scrapie infected murine model

Brain Area	Potential scrapie biomarkers	
	**SAX array**	**WCX Array**
	
Cerebellum		
Supernatant S1	none	none
Supernatant S2	4.9, 5.4 kDa	none
Hippocampus		
Supernatant S1	4.2, 4.6, 5.2, 7.4, 7.8, 22.3, 28, 29 kDa	5.1, 5.4, 6.2, 6.7, 7.1, 7.5, 9.9, 20.8, 35.1, 77 kDa
Supernatant S2	5.7, 6.9, 11.3, 16.8, 20.2, 20.9, 22.0, 22.7 kDa	4.8, 6.0, 11.9, 13.4, 14.2, 24.0 kDa

### Differential protein expression profiling is reproducible

**Figure 2:**
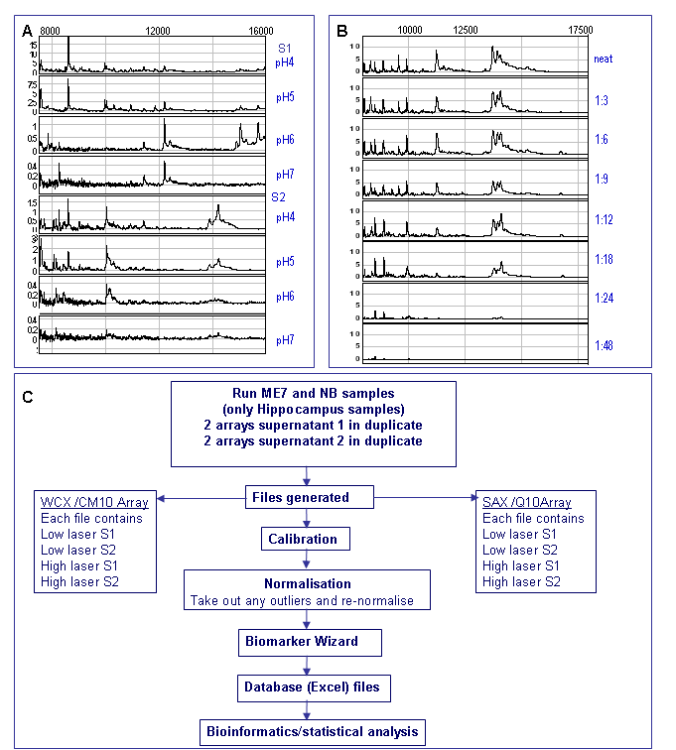
**Optimisation**. (A) Effect of pH. Supernatant 1 and supernatant 2 fractions were applied to arrays and buffers of various pH used for incubating samples and washing of arrays. (B) Effect of dilution. Same sample diluted and applied to array (further explored in Additional File 4: optimising experiments). (C) Established protocol for the preparation of samples, handling and analysis of data.

Having established differential protein expression between normal and diseased groups in the pilot study we then proceeded to extend the sample group numbers with the aim of consolidating the results. This type of assay relies on a high degree of reproducibility to achieve robust data for the incorporation into the final disease profile therefore we focused on developing a robust protocol which could be used throughout the course of the study. For this a series of optimisation experiments were performed on brain tissue samples (no. = 4 ME7 terminal 4 NB). The effect of pH (Figure 2A) and concentration (Figure 2B) were examined. Samples were incubated and arrays washed with a range of buffers at different pH (WCX/CM10 pH4-7, SAX/Q10 pH6-9). The optimal sample concentration was examined with a range of dilutions (undiluted, 1:5, 10, 20, 30, 40) of samples of a known total protein concentration. We devised a scoring system to visually assess the quality of the peaks, for example, resolution of peaks, height of intensity and quality of baseline (See additional file 4: Optimising experiments, for an example of scoring system). In conclusion, 5 μg total protein was determined as optimal for application to the array using a pH of 4.5 for the WCX/CM10 array and pH8 for the SAX/Q10 array (see Methods). As in the pilot study, there were very few clusters found in the cerebellum which were significantly different between diseased and normal groups therefore we concentrated on the hippocampus samples. Variation in peak intensity was determined by examining at least ten peaks in several samples and a coefficient of variation of 7 - 26% on the WCX and 9 - 36% on the SAX arrays was achieved. Laser power was also examined. This allows better resolution of peaks in the lower molecular mass range (2 kDa - 30 kDa) and the higher molecular range (30 kDa -100 kDa). In the terminal study two laser settings are displayed in the results (low and high). The data from one laser setting was used in the time course study for statistical analysis. An example of the effect of laser settings can be seen in Additional file 4: Optimising experiments, aliquots sheet, where one brain sample which was prepared using the protocol described in "Methods" and aliquoted into several tubes was spotted on each spot of two Q10 arrays. Three laser settings were applied and covariance analysis applied. The average values were similar in the lower (CV = 22%) and mid laser (CV = 20%) strengths but reach an unacceptably high value (CV = 300%) at the extreme high laser strength. A schematic diagram of the final protocol can be seen in Figure 2C.

During the course of this study the SELDI technology evolved rapidly resulting in better array surface and instrument technology becoming available with older instruments becoming obsolete. For this reason the time course study was carried out separately from the terminal study on a different instrument with modified arrays. Despite this we found protein markers identified in the terminal study (Table 2) also appearing in the time course study (Tables 3, 4, 5 and 6). The difference in daltons between the clusters found in the terminal study and the time course study is shown comprehensively in Table 7. Nevertheless there was found to be a close correlation between the results obtained using different machines in different laboratories and at different times. The variability within individual animals should also be considered, the terminal study running several months before the time course study. Several proteins of the same mass were found at many time intervals throughout the course of disease some at early stages of disease (see Tables 3, 4, 5 and 6), adding to confidence that the system is reliable and is reproducible.

**Table 2:** Terminal Study - List of protein peaks (m/z)

**Array type**					
**S1 WCX**			**S2 WCX**	**S1 SAX**	**S2 SAX**
**p = 0.000005**			**p = <0.005**	**p = <0.01**	**p = <0.01**
**m/z**	**m/z**	**m/z**	**m/z**	**m/z**	**m/z**
4807 ↓	9270 ↓	29186 ↓	6026 ↑	6311 ↑	10911 ↑
5242 ↓	**10101 ↑**	31074 ↓	7621 ↑	9102 ↑	18482 ↓
5289↓	**10300 ↑**	31590 ↑	7834 ↓	**10183 ↑**	22260 ↓
5303 ↓	10438 ↑	31769 ↑	8280 ↑	**10341 ↑**	25108 ↓
5471 ↓	10834 ↑	34529 ↑	8371 ↓	**10403 ↑**	28176 ↓
5780 ↓	11314 ↑	39288 ↓	8808 ↓	10535 ↑	39393 ↑
5873 ↓	11926 ↓	39656 ↓	8878 ↑	**10627 ↑**	44659 ↑
6120 ↓	12240 ↓	39112 ↓	9183 ↓	**10786 ↑**	50452 ↑
6456 ↓	12317 ↓	42708 ↓	9989 ↑	10902 ↑	67092 ↑
6558 ↓	12449 ↓	43020 ↓	10097 ↑	12302 ↑	97601 ↑
6673 ↓	12523 ↓	56090 ↑	10996 ↓	14694 ↑	
7063 ↓	13594 ↑	58596 ↓	18492 ↓	21276 ↑	
7184 ↑	13876 ↓		21043 ↓	28175 ↓	
7550 ↑	14187 ↓		33122 ↓	57798 ↓	
7847 ↓	15826 ↑		39115 ↓	66480 ↑	
8207 ↓	17840 ↓		39353 ↓		
8375 ↓	22067 ↓		47205 ↓		
8811 ↓	22431 ↓		53790 ↑		
9011 ↓	28691 ↓		66509 ↑		

p value -- the cut off value at which proteins are significant

Diseased samples significantly ↓ down regulated ↑ up regulated.

Figures in bold denote peaks which completely separate the groups

### SELDI analysis produces numerous 'clusters' common to both TSE infected and normal animals

**Figure 3:**
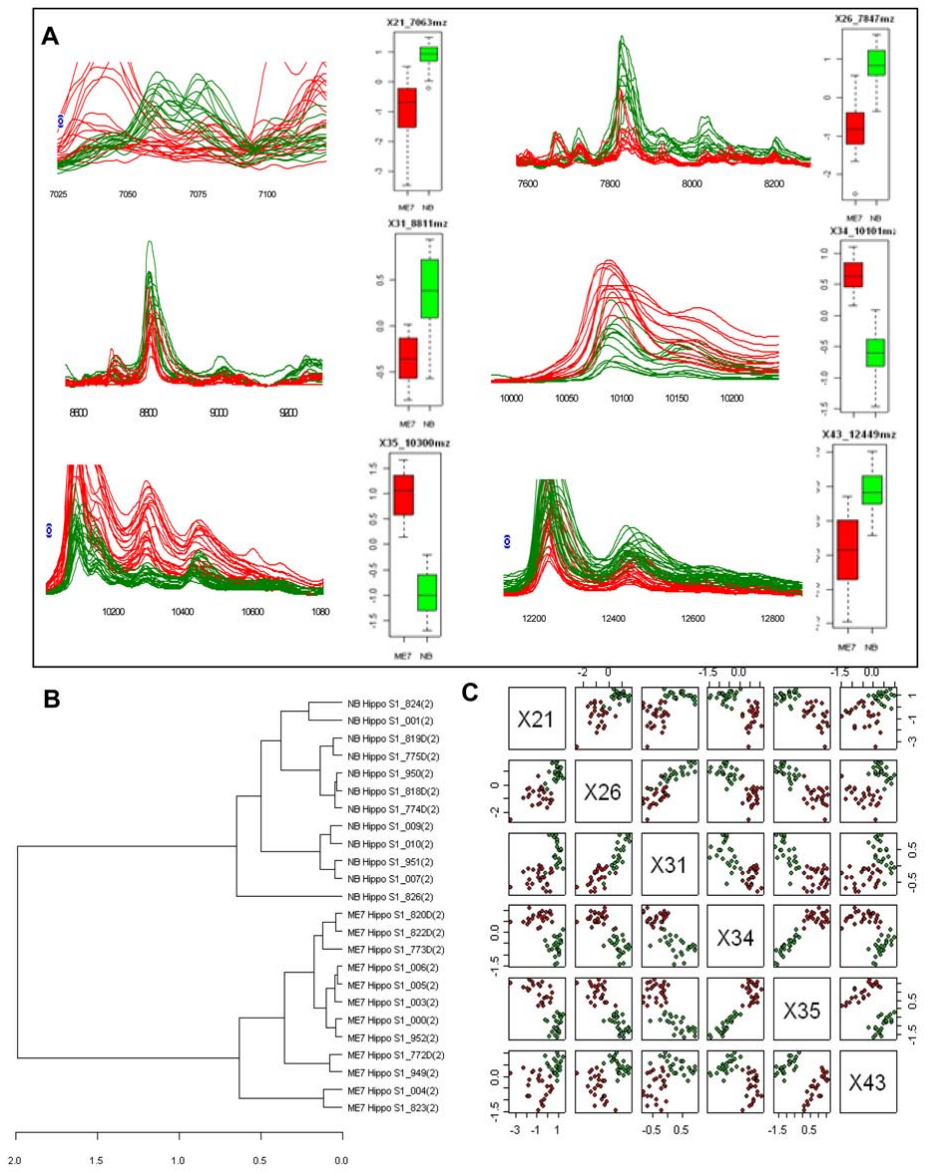
**Panel of biomarkers**. (A) data from the ProteinChip^® ^Reader is visualized in spectral format as clusters of differentially expressed protein peaks at 7063 (cluster X21), 7847(X26), 8811 (X31), 10101 (X34), 10300 (X35), 12449 (X43) m/z. Each peak within a cluster represents an individual brain sample i.e. 12 scrapie infected (red) and 12 uninfected (green) animals. Corresponding box plots for each marker displays the separation of markers based on peak height intensity, scrapie infected (red) uninfected (green). (B) cluster analysis shows separation of groups, scrapie infected (ME7) and uninfected (NB) samples. (C) pairwise plots of the above six highly significant proteins one protein against the other (red indicates scrapie infected green indicates control).

In the study of the terminal brains, spectra were generated from the two fractions (S1-soluble and S2-insoluble) in each of the TSE and uninfected brain samples. Each spot on the arrays in the terminal study was also subjected to two laser conditions (high laser and low laser). This generated 48 spectra for each 'set' i.e. S1 low, S1 high laser, S2 low and high laser on the two array surfaces (WCX and SAX). The spectral data was analyzed, by clustering peaks of similar mass (m/z ratio >2000) common to all the samples followed by statistically analyzing (Mann-Whitney (*U *test)) the peak intensities between normal and disease groups (See additional file 4: Optimising experiments, Biomarker Wizard sheet for an example). A total of 159 clusters were identified on the WCX array and 165 clusters on the SAX array. Protein peaks from individual samples displaying high statistical significance can be seen as an overlay in spectral format in Figure 3A. A full list of clusters which displayed statistically significant differences between normal and diseased groups can be viewed in Table 2.

### Statistical analysis reveals many statistically significant proteins differentially expressed between diseased and normal groups

The data files were also extensively tested using the statistical package *R *(see Methods) and were examined by cluster analysis (Figure 3B) and pair wise using scatterplots (Figure 3C).

**Figure 4:**
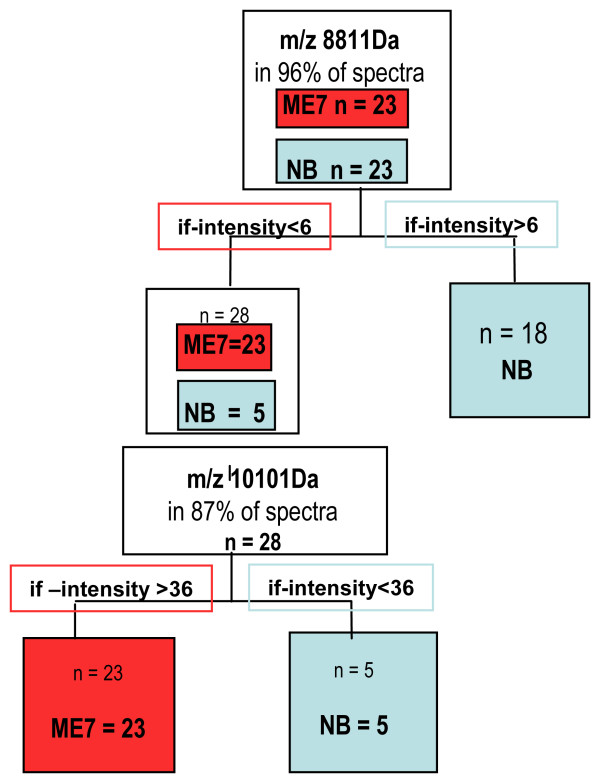
**Decision tree**. The intensity thresholds of two markers 8811 Da and 10101 Da from the panel of markers in Figure 3 were applied to mixed group data. Used in partnership these successfully identified the diseased group.

Results show many statistically significant peaks, 68 with p-value < 0.005 and 25 with p-value of < 0.01 across the two array surfaces (Table 2) separating samples into two distinct groups, TSE infected (ME7) and normal uninfected (NB). Increased statistical power was achieved by partnering six of the most highly significant protein peak intensities at masses (m/z) 7063, 7847, 8811, 10101, 10300 and 12449 (WCX array surface) showing total separation of the groups (see Figure 3A, B and 3C). This panel of markers represents the basis of a diagnostic test using pattern recognition as a 'fingerprint' for the diagnosis of scrapie. Using intensity level thresholds a decision tree algorithm can be constructed as in Figure 4 to distinguish between normal and diseased samples. The full data analysis detailing the mass to charge ratios of each significant peak can be viewed in the supplementary files (See additional file 5: Full statistical analysis of terminal study).

### Temporal protein differential expression profiles are also statistically significant

**Figure 5:**
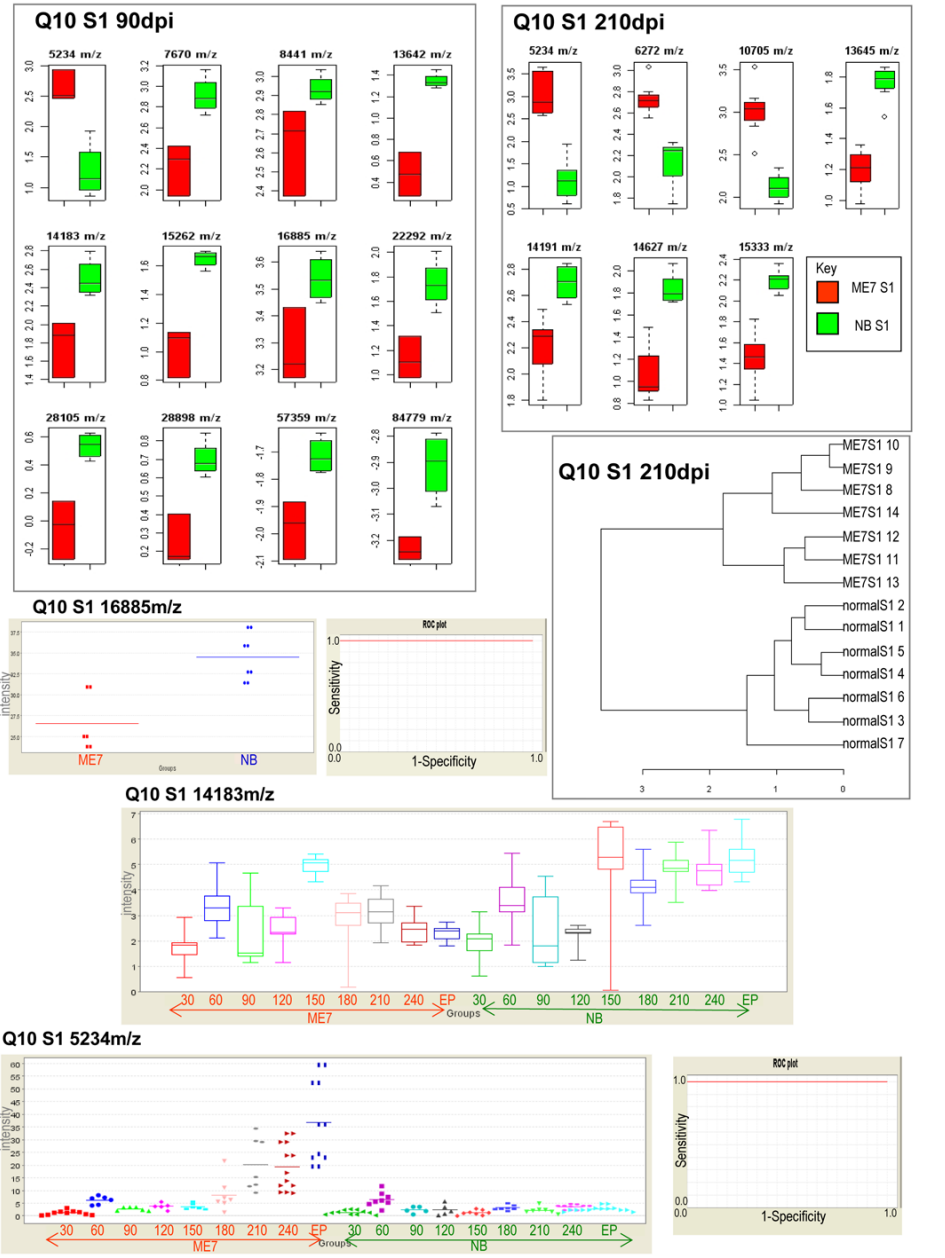
**Q10 array statistics**. Panels show box plots of proteins found to be significantly different and separate groups at 90 days post injection(dpi) and 210(dpi). A cluster analysis of the proteins at 210 dpi displays separation of groups. Examples of protein marker differences over time (S1 16885 m/z, S1 14183 m/z, S1 5234 m/z) with corresponding ROC plots(5234,16885 m/z) showing diagnostic potential.

**Figure 6:**
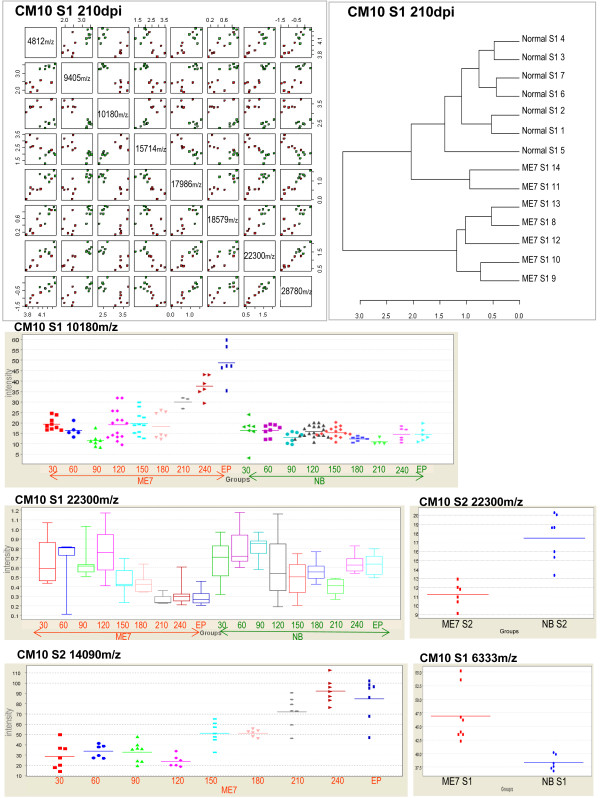
**CM10 array statistics**. Panels show a dot plot of statistically significant protein peaks at 210 days post injection, a cluster analysis of the same proteins displaying separation between groups. Protein markers at 10180, 22300 m/z supernatant 1 fraction, and 14090 m/z supernatant 2 fraction plotted over time course of disease. 22300 m/z supernatant 2 fractions and 6333 m/z supernatant 1 fraction separation of groups.

Having established that differential protein profiling was possible at the terminal stage of disease we were interested to establish if this approach could also be applied to the earlier pre-clinical stages of TSE disease. Brain samples were collected from animals at thirty day intervals and processed as in the terminal study. The resultant data from the analysis of the individual time points was statistically tested as before thus producing statistic reports for each of the eight time points. An extensive coverage of the statistical analysis can be viewed in the additional files (See additional files 6: full statistical analysis of CM10 S1, additional file 7: full statistical analysis of Q10 S1, additional file 8: full statistical analysis of CM10 S2 and additional file 9: full statistical analysis of Q10 S2). The data files were separately analysed by "Ciphergen Express^®^" software which was used to examine the relationship between individual peaks over the time course of disease. In summary, many differential protein expression profile differences were found to be statistically significant as shown in Tables 3, 4, 5 and 6. Proteins were found to be up or down-regulated during the course of disease as would be expected (for example, see Figures 5, 6). Some statistically significant markers were also found at early stages of disease (30-90 days post injection) and many well before clinical signs were apparent (240 days post injection). Differential expression between diseased and normal samples over both array types (Q10 and CM10) and supernatant fractions was found to be statistically significant. Six proteins captured on the Q10 array from supernatant 1 were significant (p <= 0.05) at 30 days post injection. Total separation of groups was achieved at 90 days post injection on both the Q10 (12 proteins p <= 0.01 Figure 5) and CM10 arrays (2 proteins p <= 0.05). Eight proteins clustered together, on the CM10 array, separated the groups at 210 dpi (Figure 6). As in the terminal study a cluster at 10180 Da (CM10) was found to separate the groups at 210 days post injection (p <= 0.01) and at End Point (p <= 0.01) (see Figure 6). A cluster at 22300 m/z displayed significant differential expression in both supernatant fractions on the CM10 array (Figure 6). A receiver operation characteristic (ROC) analysis, which is a measure the sensitivity and specificity of a marker, was applied to a few markers with encouraging results. Clusters at 16885 (p = 0.00194), 5235(p = 0.0019) (Figure 5), 8445(p = 0.0095) and 6333(p = 0.0094) m/z gave values of 1 indicating that these would be ideal candidates for inclusion in a panel of markers, distinguishing between diseased and normal groups at an early stage of disease (90 dpi). Several proteins displayed the same expression difference (up-regulated or down regulated) in most of the time points for example peaks on the Q10 array at 5334 m/z displayed up regulation in diseased tissue at 90,120, 210, 240 and EP whilst 13642 and 14183 m/z displayed down regulation at the same time points.

**Table 3:** Table of protein peaks (m/z) Q10 array Supernatant 1

**DPI**	**30**	**60**	**90**	**120**	**180**	**210**	**240**	**EP**	**Terminal study**
**P value**	**<0.05**	**<0.05**	**<0.01**	**<1.00E-04**	**<0.05**	**<0.001**	**<0.001**	**<1.00E-04**	
m/z									
2400	↑								
3243		↑							
5020						↑			
5234			↑s	↑s		↑s	↑s	↑s	
5469								↓	
6054				↓s				↓s	
6272						↑s			6311↑
7670			↓s				↓		
7800						↑		↓	
8445			↓s	↓s				↓s	
10085				↑s				↑s	10183↑
10483				↑s				↑s	10403 ↑
10707				↑s		↑s		↑s	10786 ↑
12246	↑	↓							
13037	↑								
13642			↓s	↓s		↓s	↓s	↓s	
14183			↓s	↓s		↓s	↓s	↓s	
14620			↓			↓s	↓s	↓	
14863				↑s				↑s	
15234			↓s				↓		
15333						↓s			
15460				↓s				↓s	
16885			↓s	↓s			↓s	↓s	
17090			↓s	↓s			↓	↓s	
17327				↓s				↓s	
18759								↓	
22292			↓s	↓s			↓s	↓s	
22484					↓				
26207				↓s				↓s	
28099			↓s				↓s		
28226				↓s				↓s	28175 ↓
28898			↓s				↓	↓	
35510	↑								
42483					↓				
57359			↓s						
59642	↓								
64051	↓								
84779			↓s						

DPI -- days post injection

p value -- the cut off value at which proteins are significant diseased samples significantly ↓ down regulated ↑ up regulated

**S **- significance completely separates groups

**Table 4:** Table of protein peaks (m/z) Q10 array Supernatant 2

**DPI**	**30**	**60**	**90**	**150**	**180**	**210**	**240**	**EP**	**Terminal study**
p value	<0.05	<0.05	<0.05	<0.05	<0.05	<0.05	<0.01	<0.001	
m/z									
3643					↑				
5790				↑					
5485								↓	
6740	↓								
7354	↓s								
8436		↑						↓s	
8543								↓s	
9376	↓						↓	↓	
9677	↓							↓	
10712						↑s	↑	↑	
11145						↓			
11264				↑					
11374	↓					↓			
12246							↑		
14199							↓		
14625					↓	↓s			
16880				↑					
17084				↑		↓		↓s	
18507				↓					18492↓
22297						↓s			22260 ↓
22496						↓s	↓		
25072					↓s				25108 ↓
25007								↑	
28325					↓				
30887			↓						
33748				↑				↑s	
39479					↓				
42895						↑			
44721							↑s		44659 ↑
45959								↑s	
50631			↑						
50458						↑s	↑s		50452 ↑
66563						↑		↑	
66930					↓				
67150							↑s		67092 ↑
88134					↑				
89273								↑s	
98034						↑s			
98318			↑						
97076								↑s	
96118							↑s		
101150							↑s		

DPI -- days post injection

p value -- the cut off value at which proteins are significant diseased samples significantly ↓ down regulated ↑ up regulated

**S **- significance completely separates groups

**Table 5:** Table of protein peaks (m/z) CM10 array Supernatant 1

**DPI**	**60**	**90**	**180**	**210**	**240**	**EP**	**Terminal study**
P value	<0.05	<0.05		<0.01	<0.05	<0.01	
m/z							
4817		↑		↓			4807 ↓
6333		↑s					
6435			↓				6456 ↓
6756	↓						
7599		↑					7550 ↑
8595		↑					
9405				↓s			
10180				↑s		↑s	10101 ↑
13912						↓s	13876 ↓
12426			↓				
15094		↓					
15730		↓		↑s			
17991			↓	↓		↓	
18579				↓			
19956						↑s	
21102			↓				
22322			↓	↓s	↓	↓s	
24920	↑	↑					
24848						↑s	
26014						↑	
28780				↓		↓	28691 ↓
28960			↓				
34200						↑s	
39492			↓				
42436	↑						
42509		↑					
152289		↑					
184754		↑s					
187228	↑						

p value -- the cut off value at which proteins are significant

↓ diseased samples significantly down regulated ↑ up regulated

**S **- significance completely separates groups

**Table 6:** Table of protein peaks (m/z) CM10 array Supernatant 2

**DPI**	**30**	**90**	**180**	**210**	**240**	**EP**	**Terminal**
P value	<0.05	<0.05	<0.05	<0.01	<0.01	<0.01	
m/z							
3376			↑				
4116					↑s		
5706					↓s	↓	
5749			↓				
7615				↓			7621↑
7856						↓	7834↓
8154						↓	
8437						↓	
9023				↓s		↓s	
9683						↓s	
9973				↑s	↑s		9989↑
9862			↑				
10325			↑s	↑s		↓s	
10799						↓	
11386					↑s		
12508		↓	↓			↓s	
17096					↓s		
17266			↓			↓	
18505	↑		↓				18492↓
19988						↓	
21027						↓s	21043↓
22172			↓				
22303				↓s			
24879						↑	
25129					↑s		
28118	↑						
28163		↓					
32517			↓				
32813	↑						
34052					↑s		
36515	↑						
39522			↓	↓			
42278	↑		↓				
50556					↑s		
51238	↑						
56855			↓				
66211					↑s		
83532		↑					
107215					↑s		

p value -- the cut off value at which proteins are significant

↓ diseased samples significantly down regulated ↑ up regulated

**S **- significance completely separates groups

A column at the end of each table numbered 3, 4, 5 and 6 compares the mass of significant markers found in the terminal study with the time course study. A comprehensive list comparing common peaks found in the terminal study with the temporal study is found in Table 7.

**Table 7:** Comparison Terminal study v Time course study

**S1 WCX**			**S2 WCX**			**S1 SAX**			**S2 SAX**		
**T**	**TC**	**%**	**T**	**TC**	**%**	**T**	**TC**	**%**	**T**	**TC**	**%**
m/z	m/z		m/z	m/z		m/z	m/z		m/z	m/z	
4807	4817	0.2	7621	7615	0.08	6311	6272	0.62	18492	18507	0.08
6456	6435	0.3	7834	7856	0.28	10183	10085	0.97	22260	22297	0.17
7550	7599	0.6	18492	18505	0.07	10403	10483	0.76	25108	25072	0.14
10101	10180	0.8	21043	21027	0.08	10786	10707	0.74	44659	44721	0.14
13876	13912	0.3	9989	9973	0.16	28175	28226	0.18	50452	50458	0.01
28691	28780	0.3							67092	67150	0.09

**T **- terminal study, **TC **-- time course study

### SELDI Directed Protein Identification

**Figure 7:**
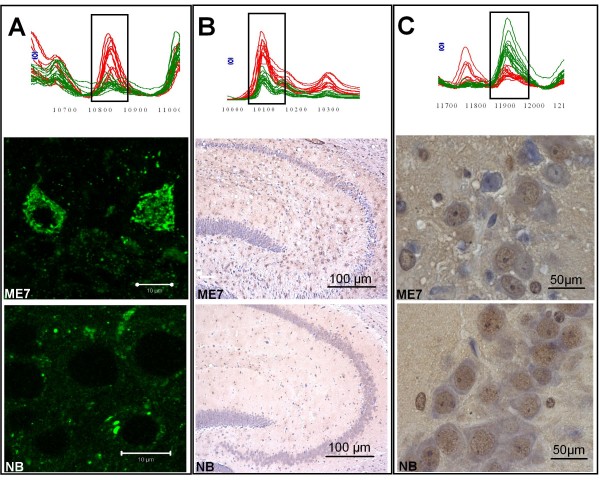
**Protein identification**. (A-C) data from the ProteinChip^® ^Reader is visualized in spectral format as a cluster of peaks (individual animals n = 12 normal and n = 12 scrapie infected) at 10834, 10101, 11784 m/z. Immunocytochemistry brain sections from terminally ill animals:-infected animals, (A-C ME7) and uninfected animals (A-C NB). (A) Cpn10, CA3 region of hippocampus × 60 oil magnification cropped confocal Z series ME7 (upper panel), NB (lower panel).(B) DBI staining in the hippocampus and dentate gyrus of scrapie infected animal × 20 magnification(ME7 upper panel), NB(lower panel). (C) FKBP12 CA3 hippocampus ME7 (upper panel) and NB (lower panel) ×100 oil magnification.

SELDI technology can be used, in addition to profiling, for the identification of individual proteins by targeting a protein of interest for isolation and purification. Whilst for the purposes of this study, the identification of each individual protein contributing to the final mathematical model was not necessary; we were interested to explore the possibility of identifying differentially expressed proteins from the murine brain tissue samples which may be important in disease mechanisms. Protein peaks shown to be statistically significant were detected in the range of ~10-12 kDa. The conditions with which the proteins were visualised i.e. a weak cationic exchange array washed with ammonium acetate pH 4.5, directed the fractionation process (see Methods). Each fraction generated was applied to the CM10 array and examined for the presence of the desired peak. Fractions containing the peaks were then further purified by 1D SDS-PAGE gel electrophoresis. The candidate proteins were excised from gels and prepared for identification by peptide mass fingerprinting using a tandem mass spectrometer equipped with a ProteinChip^® ^interface. A peak profile with a mean m/z of 10825 (Figure 7A) displaying up-regulation in the diseased samples was identified as Hsp/Cpn10 Mascot score 41. Further peaks at 10 (Figure 7B) and 11 kDa (Figure 7C) were also isolated and identified by the same methods. The 10 kDa up-regulated peak was identified by tandem mass spectrometry as diazepam binding inhibitory protein (DBI also known as Acyl Co A binding protein (ACBP) (minimum sequence coverage 48%) and a down-regulated peak at 11 kDa was identified as FK506 binding protein 12 (FKBP12, mascot score 53). The gene for FKBP12 was shown to be up-regulated in a similar murine scrapie model by Brown et al [20]. In a recent systems approach study, DBI gene expression was found to be up - regulated in TSE disease [21] which would be in agreement with our results. The yeast protein homologue to mammalian Cpn10, GroES, has been shown to inhibit GroEL in the conversion of PrP^c ^to PrP^Sc^[22].

### Identified proteins localised by immunohistochemistry

We were also interested if the observed protein expression differences seen in the SELDI spectra could be validated by immunohistochemistry. In contrast to western blotting which detects the total protein concentration of interest in samples, immunohistochemistry is more applicable for detecting subtle changes in the concentration and distribution of proteins at a cellular level.

In Figure 7A, Hsp/Cpn10 staining is visible in the pyramidal cell layer of the CA3 region of the hippocampus. Similar staining was apparent in the retrosplenial cortex and giant cells in the dorsal raphe region of the medulla. In the normal animals immunoreactivity was contained to small discrete regions near the cell surface in contrast to the diseased animals where several cells with heavily punctuate, perineuronal staining was apparent. DBI/ACBP (Figure 7B) immunoreactivity was strongly detected in the diseased animals with an up-regulation in the cytoplasm of glial like cells.

FKBP12 staining was very diffuse and widespread in both normal and diseased brain sections. Less staining was apparent in the diseased brains (Figure 7C).

## Discussion

Diagnosis of TSE infection in animals is currently only achieved by the identification of PrP^d ^in post-mortem or biopsy tissue samples usually of brain. Efforts to produce a robust ante mortem diagnostic which could be applied to crude body fluids such as urine or blood has yet to produce a commercial assay. This may be due to the reliance on identification of PrP^d ^in these samples as the nature of the infectious agent or isoform of infectious PrP in blood is currently unknown. Indeed several models now exist of disease transmission in the presence of low or undetectable levels of PrP^d^. Whilst several other protein markers have been shown to up or down regulate during TSE disease, none individually have provided adequate specificity and sensitivity for disease. The novelty of the SELDI approach is the grouping together of several individual biomarkers, displaying statistically significant differential expression, in a data driven, decision tree algorithm which is specific for the detection of TSE infection. Hence it is not the power of an individual marker but the combination of several differentially expressed markers into a TSE specific profile that is diagnostic.

To establish the validity of using this technology and the possibility of a data driven diagnostic assay for the detection of TSE disease, we decided to base this study on CNS tissue with a view to extending to blood in the longer term. The TSE's are primarily a disease of the nervous system and there is much published evidence of temporal pathological events where one would expect changes in protein expression, for example, in the murine model described in this study, changes in the synapses and in the dendrites are present at an early stage of disease [18,23,24]. Protein expression differences identified using the SELDI-TOF technology, although not definitive, may be indicative of these pathological changes. There is no such published data on the pathogenesis of TSE disease in blood. Low levels of the prion protein in blood coupled with difficulty in detection due to the inability to distinguish the abnormal disease form PrP^d ^in a background of normal PrP^c ^have hindered the application of a blood based pre-mortem assay using PrP^d ^as a marker. In this study we sought to determine if the SELDI approach would both distinguish diseased animals from uninfected animals and determine the earliest time point at which this is possible. By building confidence in the SELDI approach using the CNS material we would be better equipped to interpret results from any subsequent experiments in body fluids such as blood.

We have established in this study that there are many potential biomarkers which could be used together as a panel for the construction of a decision tree classifier which could be used to identify the presence of TSE disease. In addition we have confirmed the differential expression of three proteins identified by SELDI using immunohistochemistry.

The results of our initial experiments comparing an area of the brain with no obvious pathology (cerebellum), with an area displaying severe pathology in the same animal (hippocampus) supported our hypothesis that the number of differential protein expression differences correlated with the observed pathological changes already documented in this murine model. At this terminal stage of disease there are arguably many events which occur that are not specific responses to TSE disease, but rather to the more general responses to injection and disease in the brain. However if this was a general effect we would have also expected more expression differences in the cerebellum. Building on these results, stringent quality controlled protocols were developed to address reproducibility by optimising sample preparation, instrument conditions and data management.

The terminal study consolidated the initial results in a larger group of samples with many differential expression differences apparent. This experiment represented the most extreme comparisons, infected versus non-infected at the end stage of disease, therefore it was necessary to examine time points during disease progression to ascertain subtle changes occurring throughout the course of disease which would indicate potential biomarkers for a pre-clinical diagnostic panel. In this temporal study there were numerous proteins in both uninfected and infected groups which clustered together over a wide molecular weight range and over several time points. Statistically significant differentially expressed proteins grouped together separated the scrapie brain homogenate inoculated group from the normal brain homogenate inoculated group at several time points however statistical significance for the proteins individually was not achieved at every time point throughout the course of disease. Further investigation of the data (Tables 3, 4, 5 and 6) was carried out in the Ciphergen Express software to ascertain if these protein clusters observed as statistically significant at particular time points were present at all time points. This analysis shows a clear relationship between time points as demonstrated in Figures 5 &6, with trends of up regulation or down regulation apparent over the course of infection. Given that these data are derived from individual animals and the samples analysed in "batches" of samples over time the results in expression differences are extremely consistent. Several proteins displayed consistent up or down regulation as displayed by the peak seen in Figure 7 at 10180 m/z which is particularly striking as the normal group remains consistently low at all the time points whereas the diseased group displays an up regulated trend from an early time point. Utilizing similarly robust biomarkers and combining highly significant markers at specific time points, a diagnostic panel could be compiled to be applied as a diagnostic decision tree algorithm for the presence of TSE disease.

As previously stated in this study our primary aim was to establish a data driven approach to diagnosing the presence of TSE disease at a pre-clinical time point. Although there is no necessity to establish the identity of each protein for successful application of the diagnostic panel as a data driven assay, we identified three differentially expressed proteins (Cpn10, FKBP12, DBI) using data from the SELDI analysis to support the strength of this approach. Immunohistochemistry results confirmed the differential expression observed in the SELDI data for all three proteins identified. DBI was first isolated from brain material[25] and is found in glial cells. As this scrapie model displays extensive gliosis in the hippocampus it is perhaps not surprising that the immunohistochemistry results show increased staining in glial-like cells within the scrapie brain. This gives us confidence that we are indeed isolating disease specific differentially expressed proteins from the brain homogenates. Further investigation of possible roles in the pathogenesis of TSE disease for these proteins will be required.

## Conclusion

The project aimed to establish if this approach was feasible starting with tissue samples from the CNS which would be the basis of a post-mortem test, still a surveillance requirement for conclusive evidence of disease status. We have targeted known areas where disease associated changes occur in the murine scrapie model and successfully demonstrated that a training set, based on discriminating protein peaks can be obtained to form a pattern based algorithm for the detection of TSE disease in unknown samples. Having established that the technique is valid based on these studies in CNS tissue, our ongoing studies are being performed using a large archive of blood samples from an ovine time course TSE infection study. The training set of samples will be used to establish a protein fingerprint for TSE infection in blood, which will then be tested against a testing set of samples (including blood from sheep with other neurological diseases) to assess the sensitivity and specificity of the assay. Markers will be compared with those identified in the mouse studies to determine whether any common patterns of differential protein expression in TSE disease exist across different species.

## Competing interests

The authors declare that they have no competing interests.

## Authors' contributions

JB and JF conceived and designed the study. JB analyzed samples, collated and interpreted results and wrote the manuscript. MW carried out all statistical analysis involved in study and contributed to drafting the manuscript. MWH, JWI, RB contributed to the drafting of the manuscript and advised JB on intellectual content. NH and CH advised JB on the technical aspects of the study and contributed to the drafting of the manuscript. NH provided technical data including the identification of proteins on MS/MS platform. CH provided data analysis on Ciphergen Express for the time course study.

## Pre-publication history

The pre-publication history for this paper can be accessed here:

http://www.biomedcentral.com/1471-2334/9/188/prepub

## Supplementary Material

Additional file 1 Hsp10 protein identification Click here for file

Additional file 2 FKBP12 protein identification Click here for file

Additional file 3 DBI protein identification Click here for file

Additional file 4 **Optimisation of SELDI conditions**. Sheet 1, Optimising experiments - Traces from instrument illustrating the effects of concentration and laser power on the resolution of peaks. Sheet 2, Aliquots - Replication of one sample over array. Sheet 3, Biomarker Wizard - Visualisation by Biomarker Wizard (Mann -Whitney analysis) of primary experiments. Click here for file

Additional file 5 Full statistical analysis of Terminal study Click here for file

Additional file 6 Full statistical analysis of CM10 supernatant 1 arrays Click here for file

Additional file 7 Full statistical analysis of Q10 supernatant 1 arrays Click here for file

Additional file 8 Full statistical analysis of CM10 supernatant 2 arrays Click here for file

Additional file 9 Full statistical analysis of Q10 supernatant 2 arrays Click here for file
