# Supplementary figures and images for: Differential protein profiling as a potential multi-marker approach for TSE diagnosis
Source: BMC Infect Dis. 2009 Nov 27;9:188. doi: 10.1186/1471-2334-9-188 (PMC2794872; doi:10.1186/1471-2334-9-188)

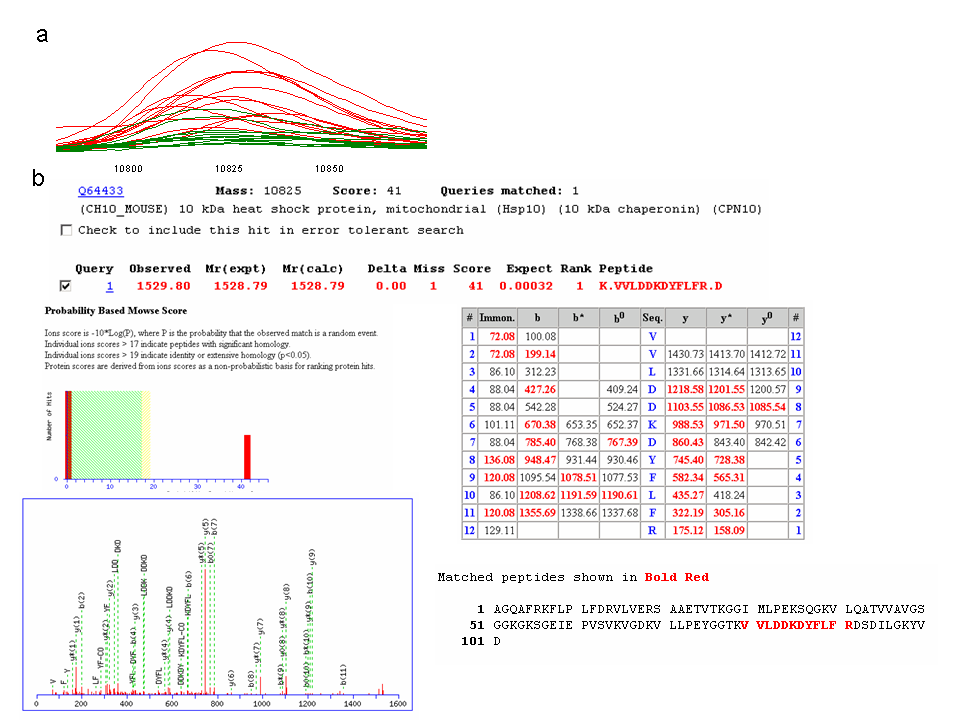

Supplement: Additional file 1 — Hsp10 protein identification [file 1471-2334-9-188-S1.TIFF]

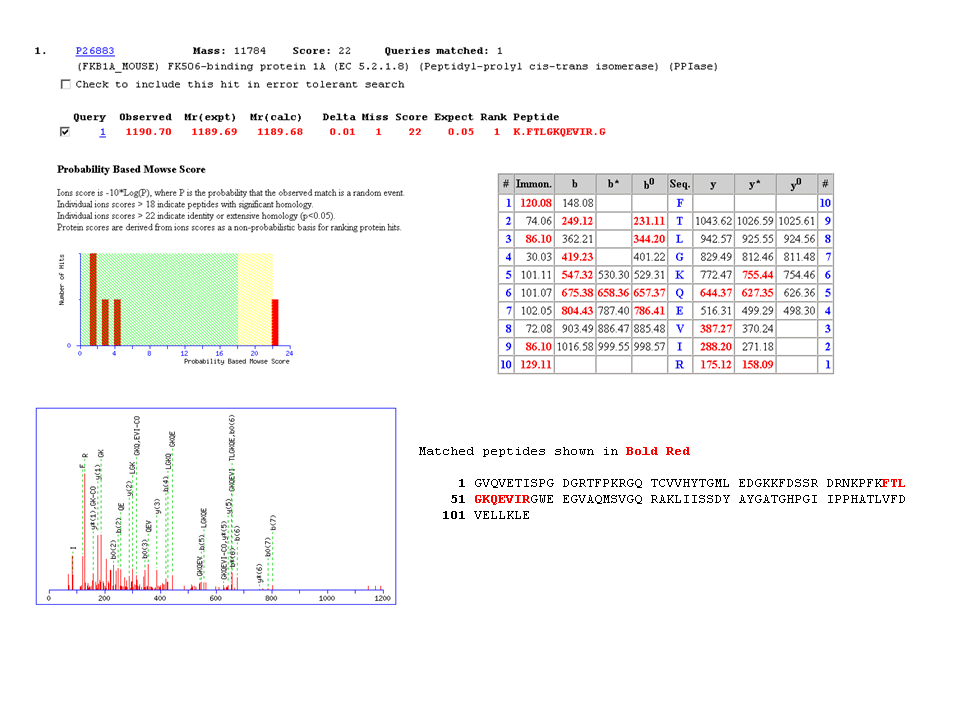

Supplement: Additional file 2 — FKBP12 protein identification [file 1471-2334-9-188-S2.TIFF]

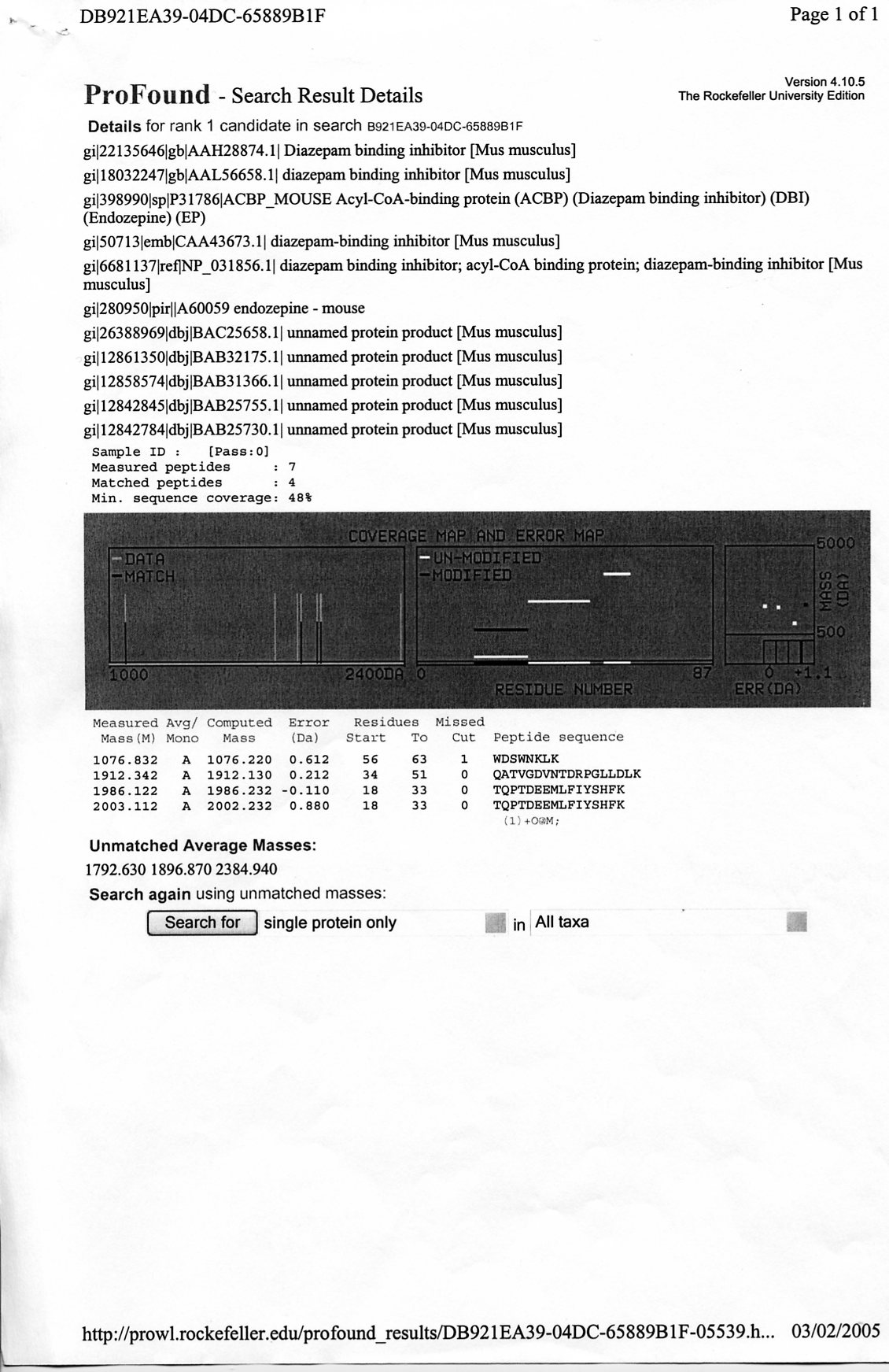


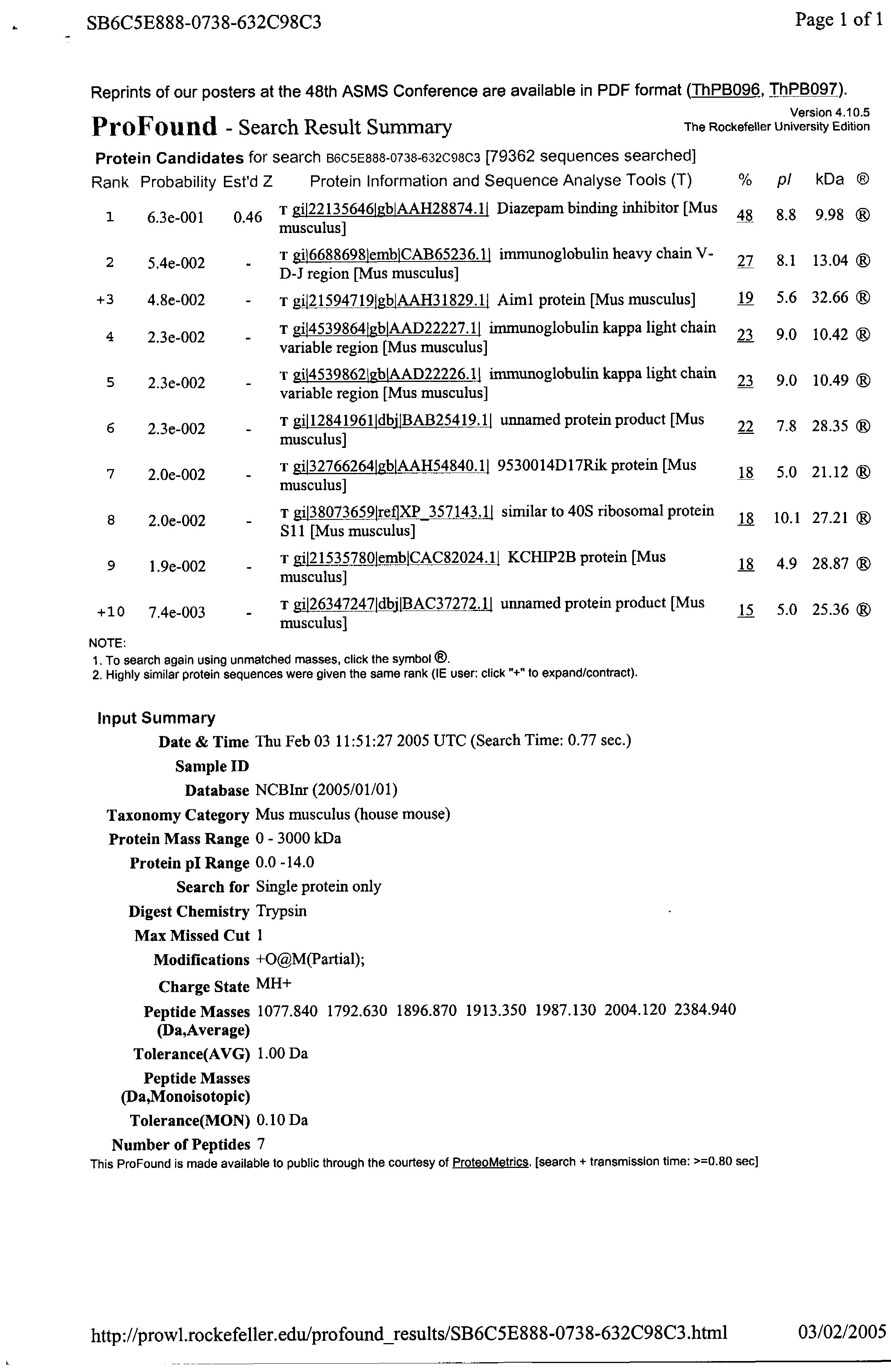

Supplement: Additional file 3 — DBI protein identification [file 1471-2334-9-188-S3.DOC]
