# Supplementary material for: Differential protein profiling as a potential multi-marker approach for TSE diagnosis
Source: BMC Infect Dis. 2009 Nov 27;9:188. doi: 10.1186/1471-2334-9-188 (PMC2794872; doi:10.1186/1471-2334-9-188)
Supplement: Additional file 5 — Full statistical analysis of Terminal study [file 1471-2334-9-188-S5.PDF]

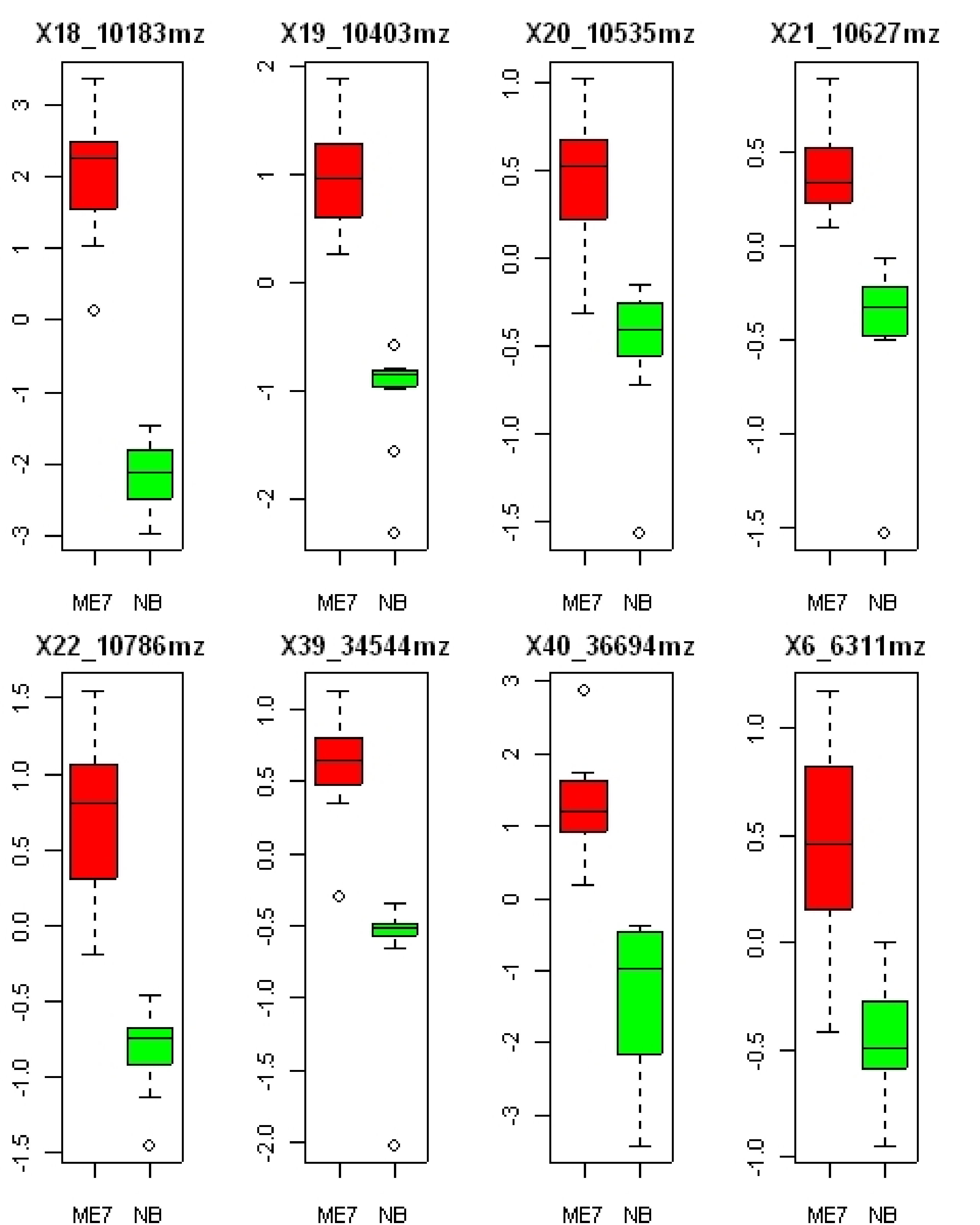

X18_10183mz
X19_10403mz
X20_10535mz
X21_10627mz
X22_10786mz
X39_34544mz
X40_36694mz
X6_6311mz
ME7
NB

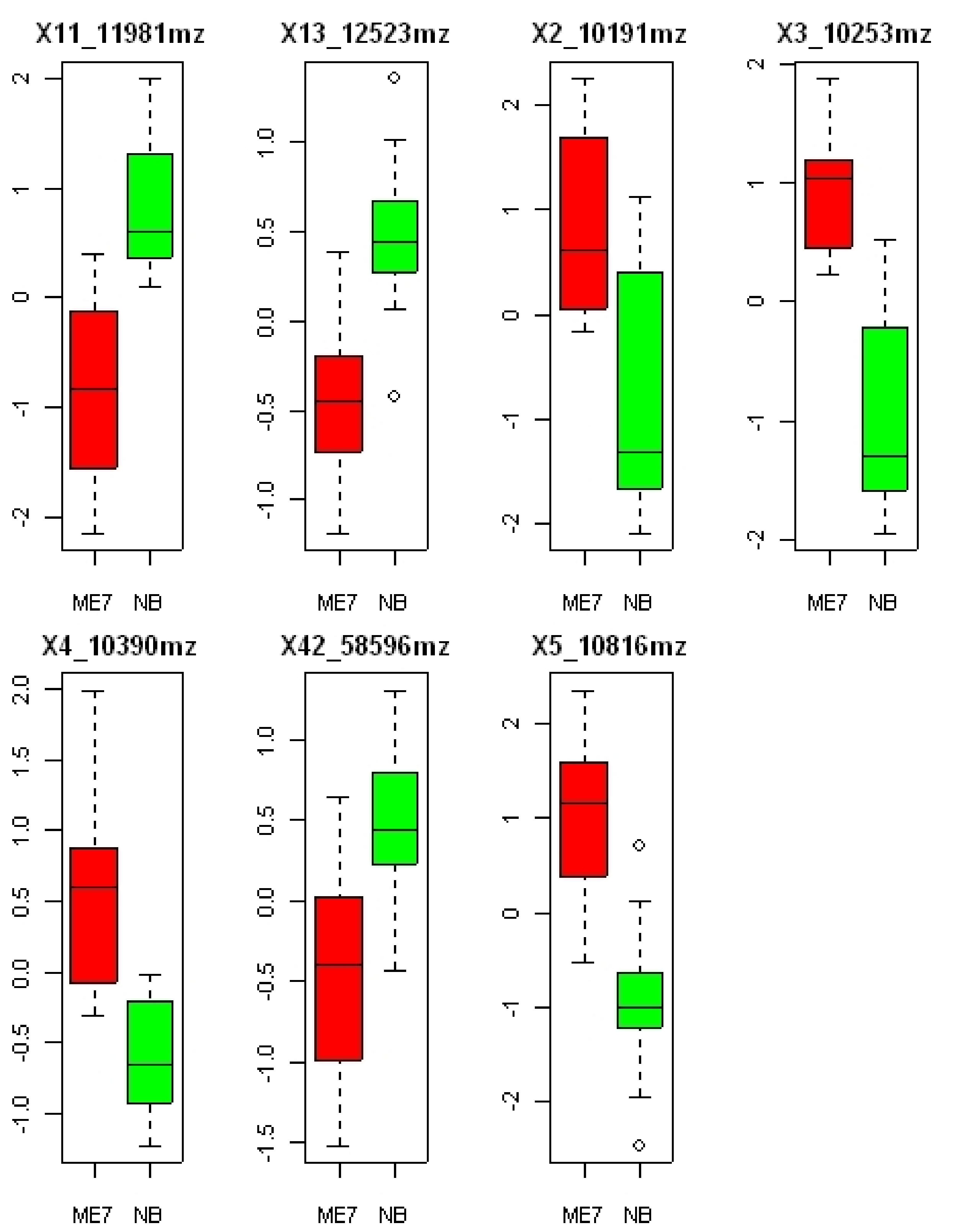
X11_11981mz
X13_12523mz
X2_10191mz
X3_10253mz
X4_10390mz
X42_58596mz
X5_10816mz
ME7
NB

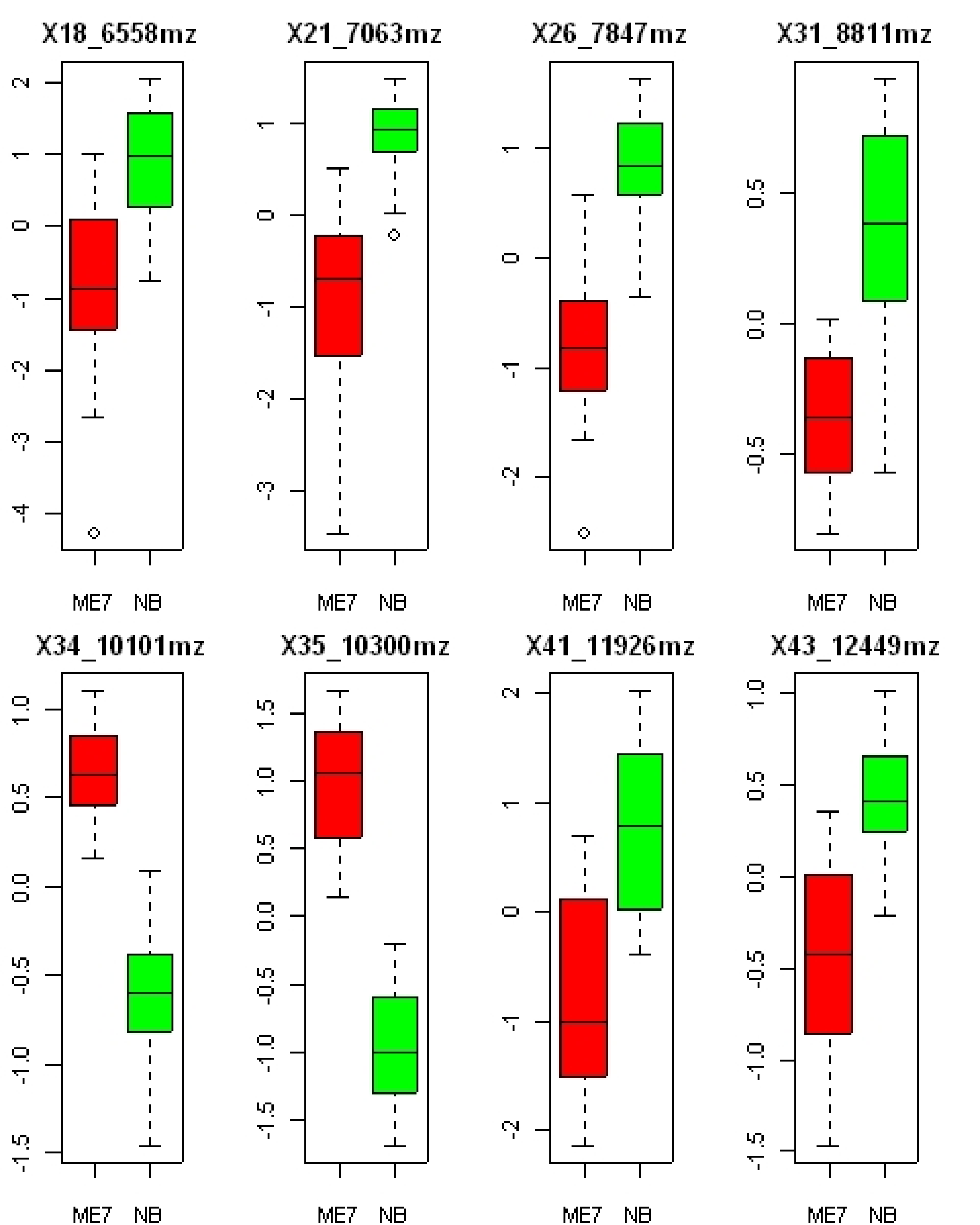

X18_6558mz
X21_7063mz
X26_7847mz
X31_8811mz
X34_10101mz
X35_10300mz
X41_11926mz
X43_12449mz
ME7
NB

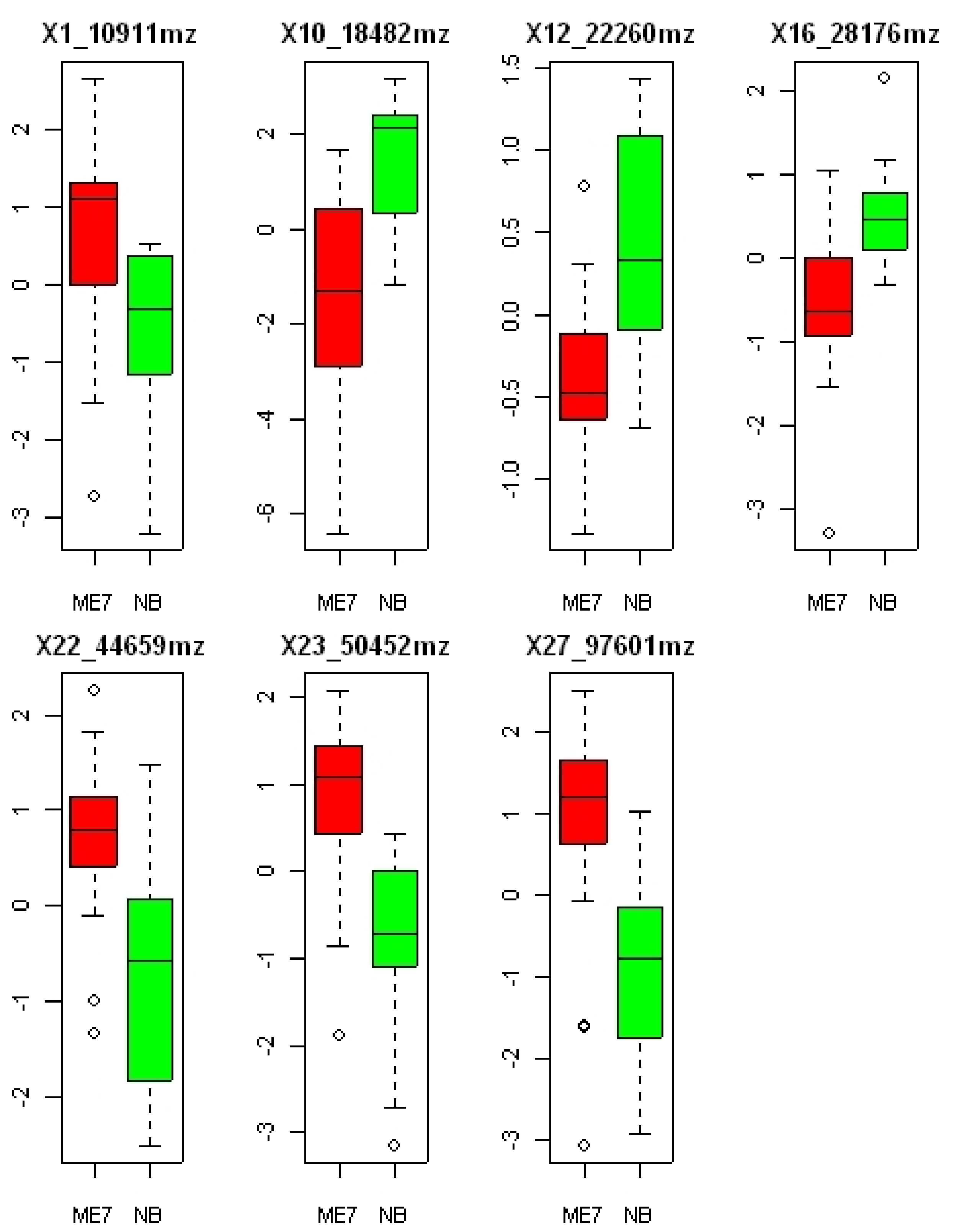
X1_10911mz
X10_18482mz
X12_22260mz
X16_28176mz
X22_44659mz
X23_50452mz
X27_97601mz
ME7
NB

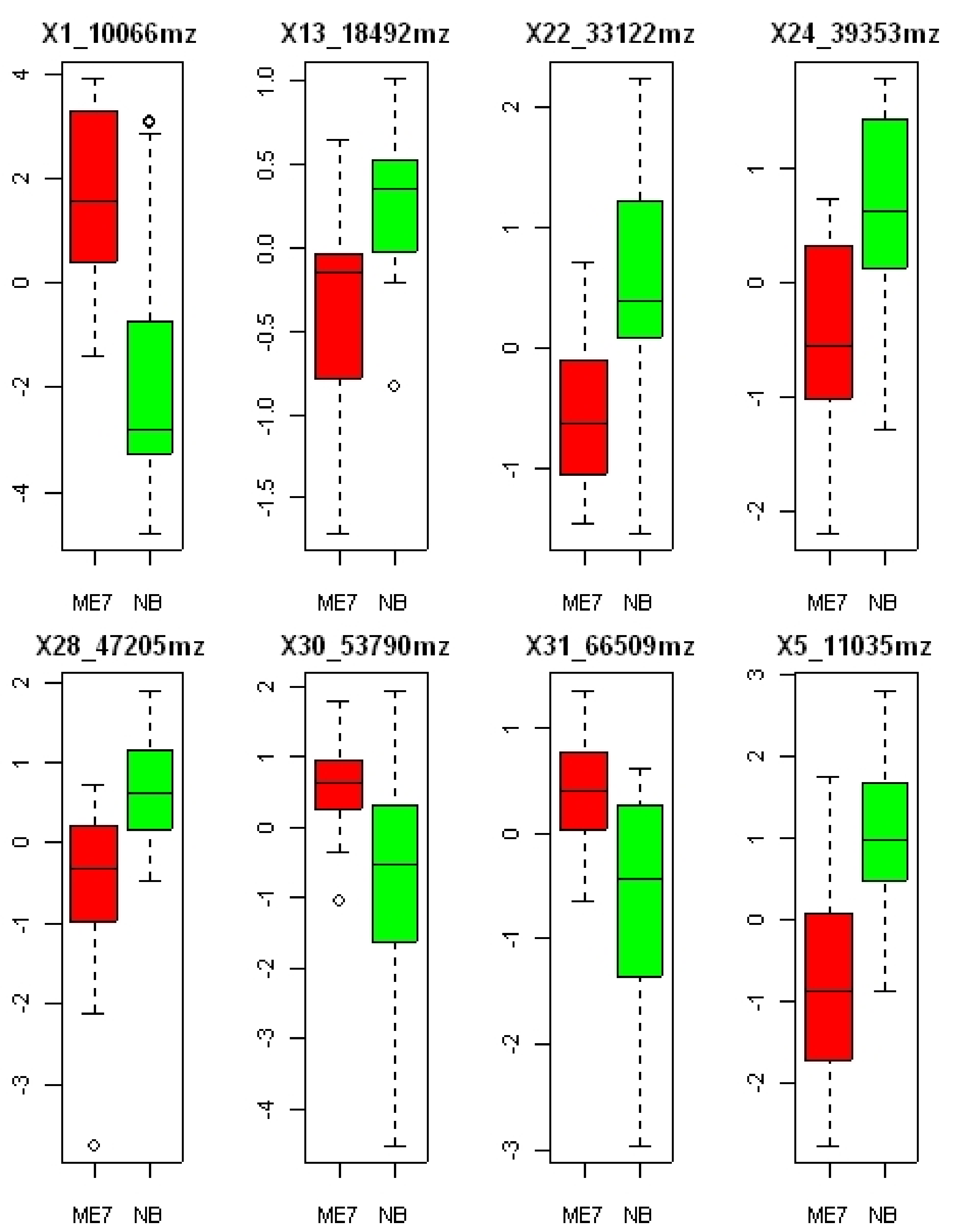
X1_10066mz
X13_18492mz
X22_33122mz
X24_39353mz
X28_47205mz
X30_53790mz
X31_66509mz
X5_11035mz
ME7
NB

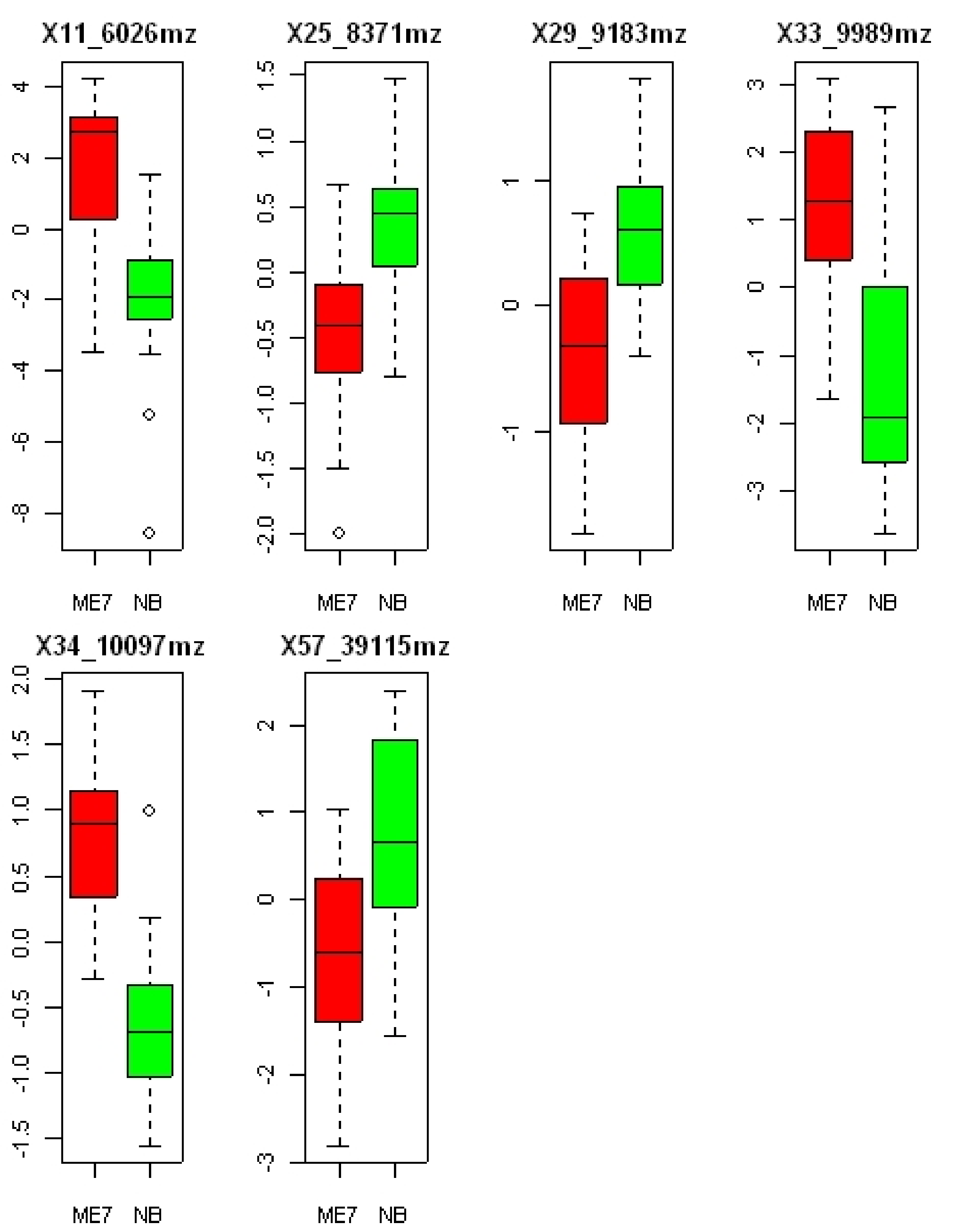

X11_6026mz
X25_8371mz
X29_9183mz
X33_9989mz
X34_10097mz
X57_39115mz
ME7
NB

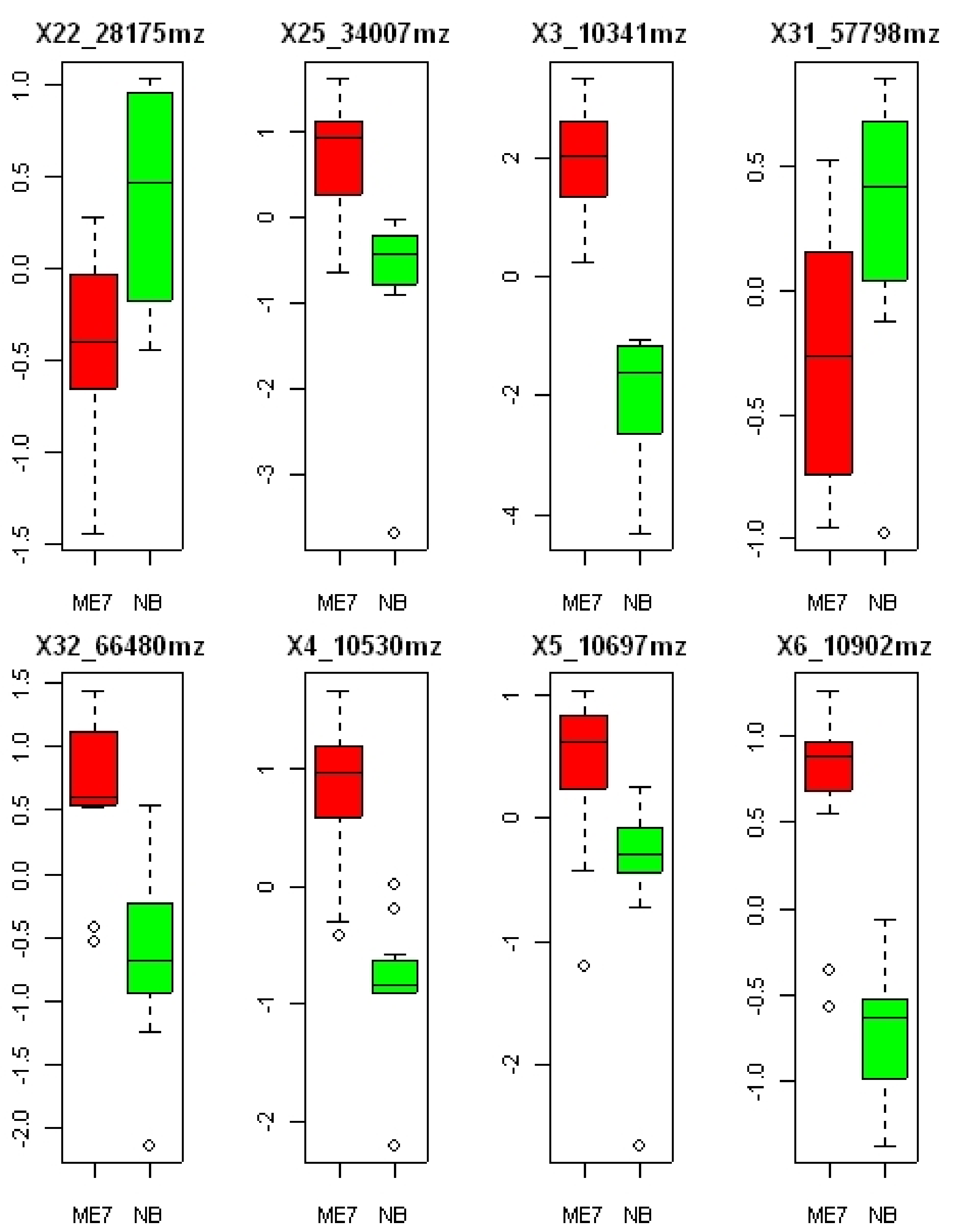

X22_28175mz
X25_34007mz
X3_10341mz
X31_57798mz
X32_66480mz
X4_10530mz
X5_10697mz
X6_10902mz
ME7
NB

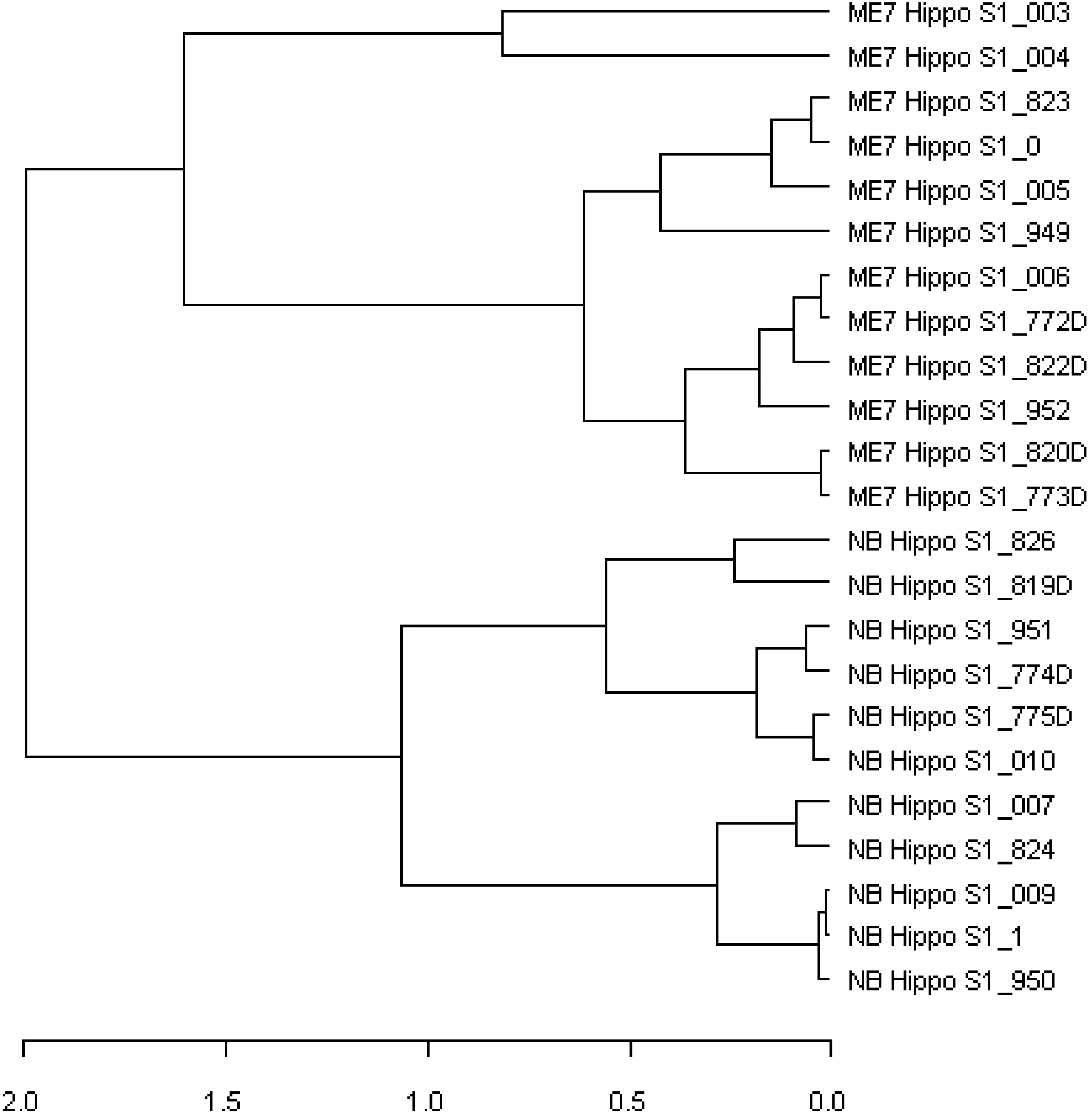

ME7 Hippo S1_003
ME7 Hippo S1_004
ME7 Hippo S1_823
ME7 Hippo S1_0
ME7 Hippo S1_005
ME7 Hippo S1_949
ME7 Hippo S1_006
ME7 Hippo S1_772D
ME7 Hippo S1_822D
ME7 Hippo S1_952
ME7 Hippo S1_820D
ME7 Hippo S1_773D
NB Hippo S1_826
NB Hippo S1_819D
NB Hippo S1_951
NB Hippo S1_774D
NB Hippo S1_775D
NB Hippo S1_010
NB Hippo S1_007
NB Hippo S1_824
NB Hippo S1_009
NB Hippo S1_1
NB Hippo S1_950
2.0
1.5
1.0
0.5
0.0

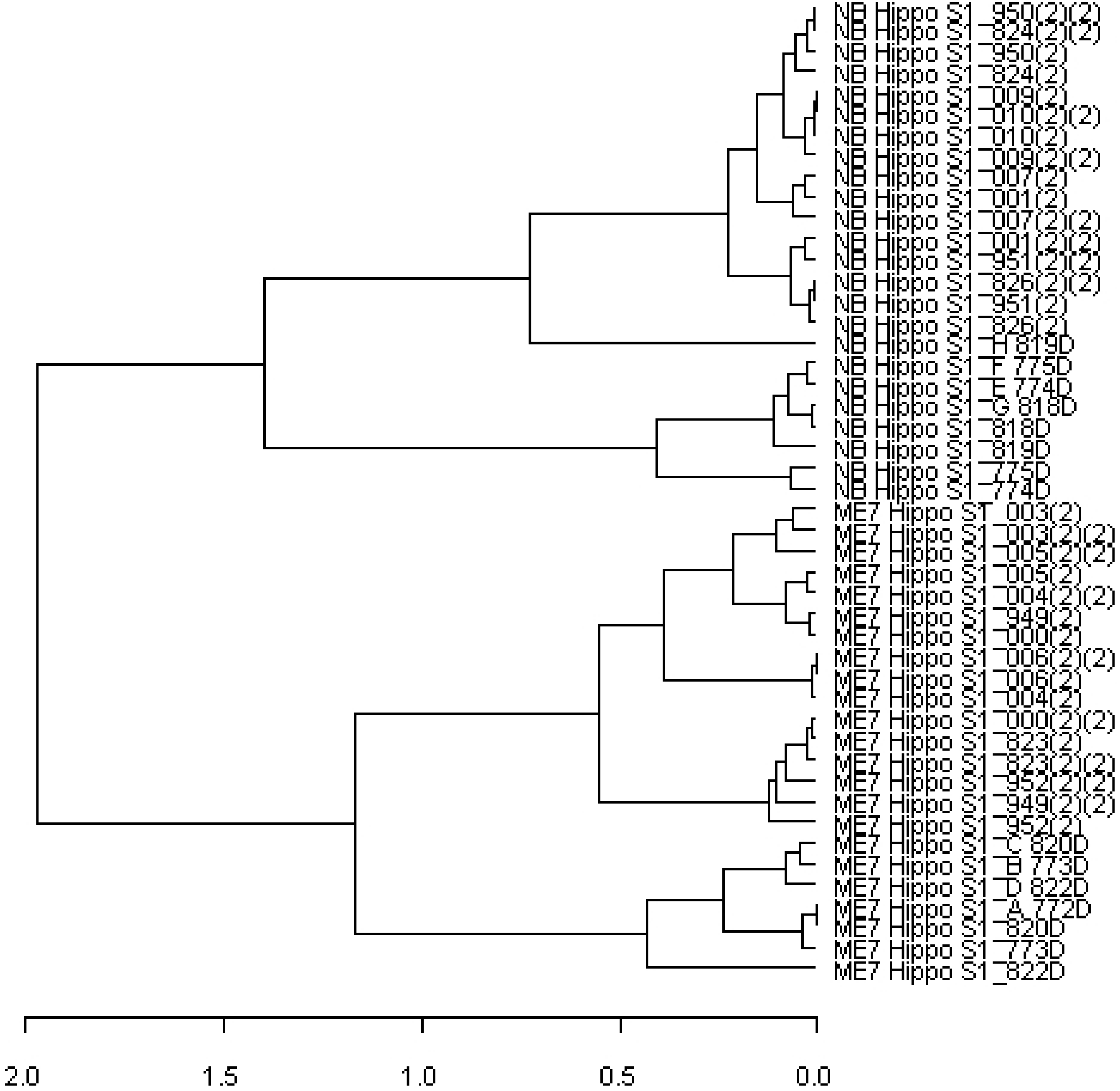
NB Hippo S1_950(2)(2)
NB Hippo S1_824(2)(2)
NB Hippo S1_950(2)
NB Hippo S1_824(2)
NB Hippo S1_009(2)
NB Hippo S1_010(2)(2)
NB Hippo S1_010(2)
NB Hippo S1_009(2)(2)
NB Hippo S1_007(2)
NB Hippo S1_001(2)
NB Hippo S1_007(2)(2)
NB Hippo S1_001(2)(2)
NB Hippo S1_951(2)(2)
NB Hippo S1_826(2)(2)
NB Hippo S1_951(2)
NB Hippo S1_826(2)
NB Hippo S1_H 819D
NB Hippo S1_F 775D
NB Hippo S1_E 774D
NB Hippo S1_G 818D
NB Hippo S1_818D
NB Hippo S1_819D
NB Hippo S1_775D
NB Hippo S1_774D
ME7 Hippo S1_003(2)
ME7 Hippo S1_003(2)(2)
ME7 Hippo S1_005(2)(2)
ME7 Hippo S1_005(2)
ME7 Hippo S1_004(2)(2)
ME7 Hippo S1_949(2)
ME7 Hippo S1_000(2)
ME7 Hippo S1_006(2)(2)
ME7 Hippo S1_006(2)
ME7 Hippo S1_004(2)
ME7 Hippo S1_000(2)(2)
ME7 Hippo S1_823(2)
ME7 Hippo S1_823(2)(2)
ME7 Hippo S1_952(2)(2)
ME7 Hippo S1_949(2)(2)
ME7 Hippo S1_952(2)
ME7 Hippo S1_C 820D
ME7 Hippo S1_B 773D
ME7 Hippo S1_D 822D
ME7 Hippo S1_A 772D
ME7 Hippo S1_820D
ME7 Hippo S1_773D
ME7 Hippo S1_822D
2.0
1.5
1.0
0.5
0.0

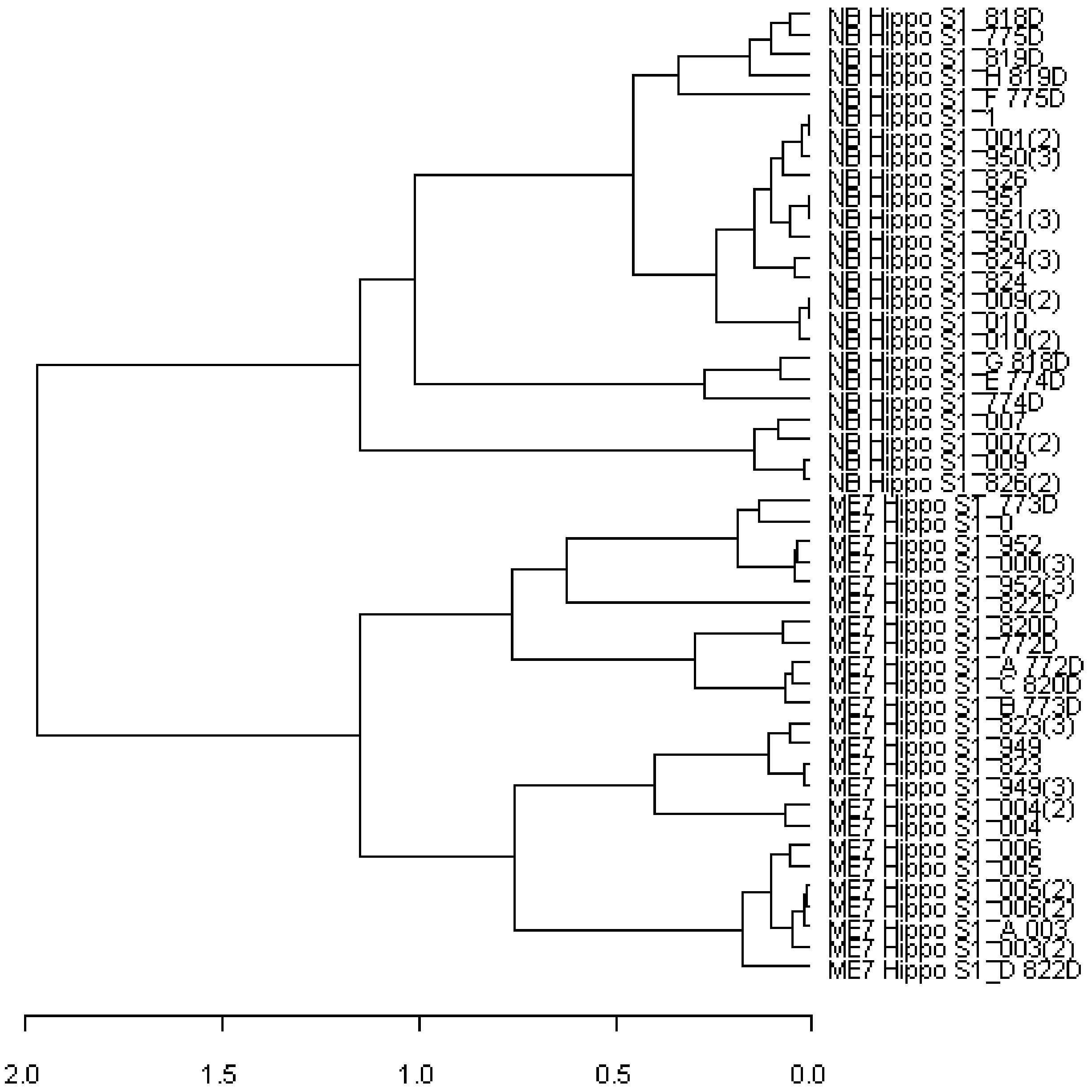
NB Hippo S1_818D
NB Hippo S1_775D
NB Hippo S1_819D
NB Hippo S1_H 819D
NB Hippo S1_F 775D
NB Hippo S1_1
NB Hippo S1_001(2)
NB Hippo S1_950(3)
NB Hippo S1_826
NB Hippo S1_951
NB Hippo S1_951(3)
NB Hippo S1_950
NB Hippo S1_824(3)
NB Hippo S1_824
NB Hippo S1_009(2)
NB Hippo S1_010
NB Hippo S1_010(2)
NB Hippo S1_G 818D
NB Hippo S1_E 774D
NB Hippo S1_774D
NB Hippo S1_007
NB Hippo S1_007(2)
NB Hippo S1_009
NB Hippo S1_826(2)
ME7 Hippo S1_773D
ME7 Hippo S1_0
ME7 Hippo S1_952
ME7 Hippo S1_000(3)
ME7 Hippo S1_952(3)
ME7 Hippo S1_822D
ME7 Hippo S1_820D
ME7 Hippo S1_772D
ME7 Hippo S1_A 772D
ME7 Hippo S1_C 820D
ME7 Hippo S1_B 773D
ME7 Hippo S1_823(3)
ME7 Hippo S1_949
ME7 Hippo S1_823
ME7 Hippo S1_949(3)
ME7 Hippo S1_004(2)
ME7 Hippo S1_004
ME7 Hippo S1_006
ME7 Hippo S1_005
ME7 Hippo S1_005(2)
ME7 Hippo S1_006(2)
ME7 Hippo S1_A 003
ME7 Hippo S1_003(2)
ME7 Hippo S1_D 822D
2.0
1.5
1.0
0.5
0.0

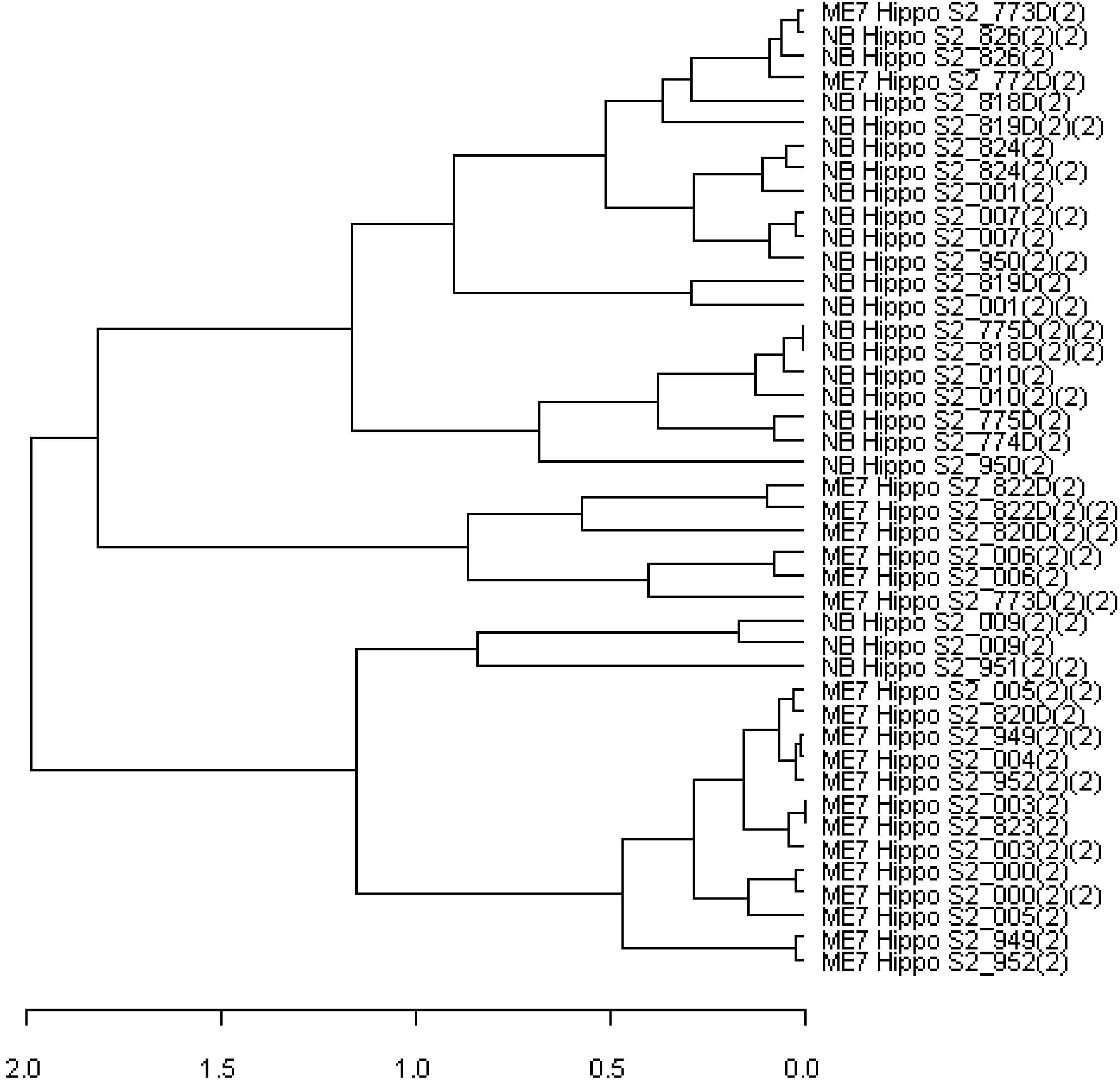
ME7 Hippo S2_773D(2)
NB Hippo S2_826(2)(2)
NB Hippo S2_826(2)
ME7 Hippo S2_772D(2)
NB Hippo S2_818D(2)
NB Hippo S2_819D(2)(2)
NB Hippo S2_824(2)
NB Hippo S2_824(2)(2)
NB Hippo S2_001(2)
NB Hippo S2_007(2)(2)
NB Hippo S2_007(2)
NB Hippo S2_950(2)(2)
NB Hippo S2_819D(2)
NB Hippo S2_001(2)(2)
NB Hippo S2_775D(2)(2)
NB Hippo S2_818D(2)(2)
NB Hippo S2_010(2)
NB Hippo S2_010(2)(2)
NB Hippo S2_775D(2)
NB Hippo S2_774D(2)
NB Hippo S2_950(2)
ME7 Hippo S2_822D(2)
ME7 Hippo S2_822D(2)(2)
ME7 Hippo S2_820D(2)(2)
ME7 Hippo S2_006(2)(2)
ME7 Hippo S2_006(2)
ME7 Hippo S2_773D(2)(2)
NB Hippo S2_009(2)(2)
NB Hippo S2_009(2)
NB Hippo S2_951(2)(2)
ME7 Hippo S2_005(2)(2)
ME7 Hippo S2_820D(2)
ME7 Hippo S2_949(2)(2)
ME7 Hippo S2_004(2)
ME7 Hippo S2_952(2)(2)
ME7 Hippo S2_003(2)
ME7 Hippo S2_823(2)
ME7 Hippo S2_003(2)(2)
ME7 Hippo S2_000(2)
ME7 Hippo S2_000(2)(2)
ME7 Hippo S2_005(2)
ME7 Hippo S2_949(2)
ME7 Hippo S2_952(2)
2.0
1.5
1.0
0.5
0.0

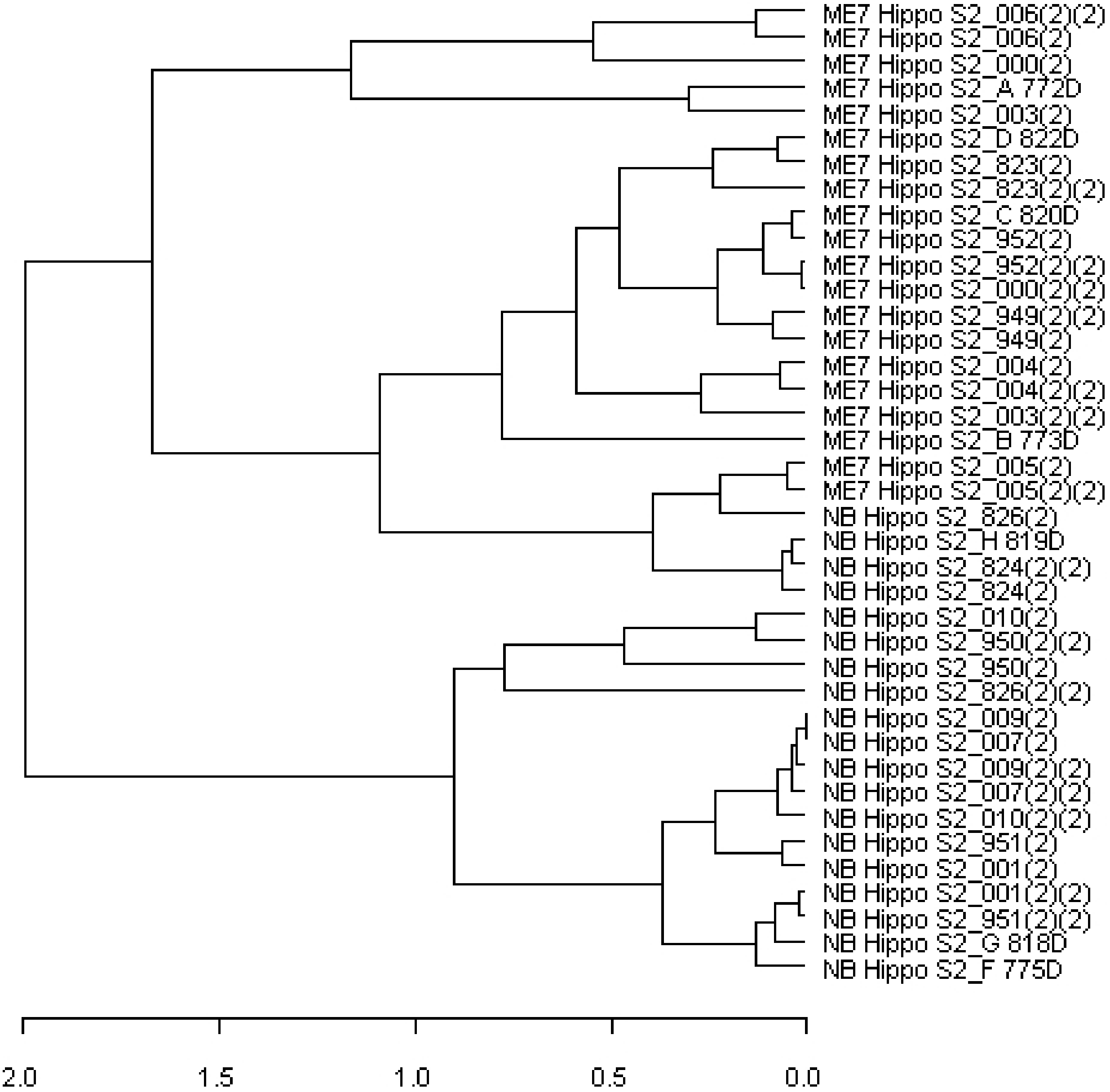

ME7 Hippo S2_006(2)(2)
ME7 Hippo S2_006(2)
ME7 Hippo S2_000(2)
ME7 Hippo S2_A 772D
ME7 Hippo S2_003(2)
ME7 Hippo S2_D 822D
ME7 Hippo S2_823(2)
ME7 Hippo S2_823(2)(2)
ME7 Hippo S2_C 820D
ME7 Hippo S2_952(2)
ME7 Hippo S2_952(2)(2)
ME7 Hippo S2_000(2)(2)
ME7 Hippo S2_949(2)(2)
ME7 Hippo S2_949(2)
ME7 Hippo S2_004(2)
ME7 Hippo S2_004(2)(2)
ME7 Hippo S2_003(2)(2)
ME7 Hippo S2_B 773D
ME7 Hippo S2_005(2)
ME7 Hippo S2_005(2)(2)
NB Hippo S2_826(2)
NB Hippo S2_H 819D
NB Hippo S2_824(2)(2)
NB Hippo S2_824(2)
NB Hippo S2_010(2)
NB Hippo S2_950(2)(2)
NB Hippo S2_950(2)
NB Hippo S2_826(2)(2)
NB Hippo S2_009(2)
NB Hippo S2_007(2)
NB Hippo S2_009(2)(2)
NB Hippo S2_007(2)(2)
NB Hippo S2_010(2)(2)
NB Hippo S2_951(2)
NB Hippo S2_001(2)
NB Hippo S2_001(2)(2)
NB Hippo S2_951(2)(2)
NB Hippo S2_G 818D
NB Hippo S2_F 775D
2.0
1.5
1.0
0.5
0.0

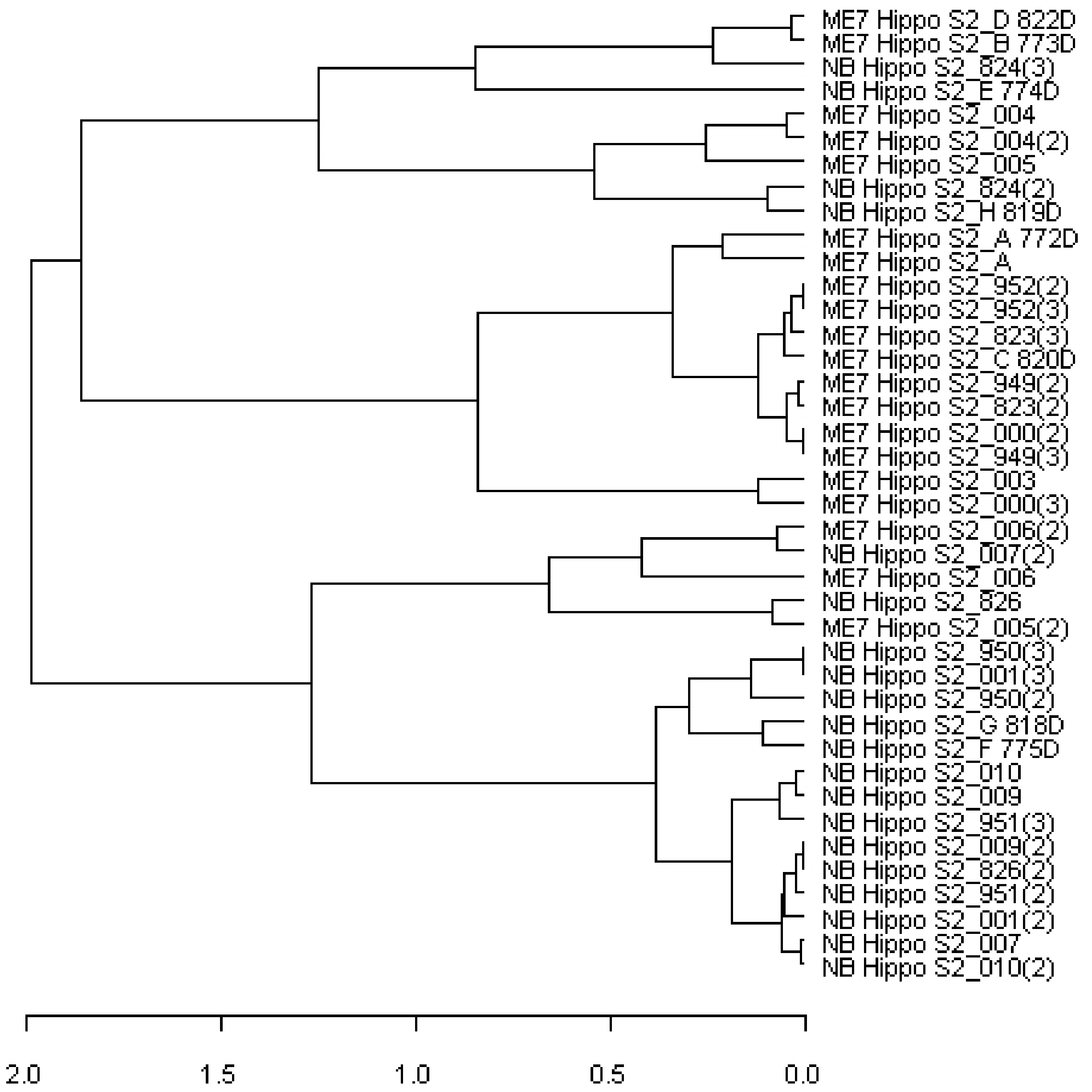
ME7 Hippo S2_D 822D
ME7 Hippo S2_B 773D
NB Hippo S2_824(3)
NB Hippo S2_E 774D
ME7 Hippo S2_004
ME7 Hippo S2_004(2)
ME7 Hippo S2_005
NB Hippo S2_824(2)
NB Hippo S2_H 819D
ME7 Hippo S2_A 772D
ME7 Hippo S2_A
ME7 Hippo S2_952(2)
ME7 Hippo S2_952(3)
ME7 Hippo S2_823(3)
ME7 Hippo S2_C 820D
ME7 Hippo S2_949(2)
ME7 Hippo S2_823(2)
ME7 Hippo S2_000(2)
ME7 Hippo S2_949(3)
ME7 Hippo S2_003
ME7 Hippo S2_000(3)
ME7 Hippo S2_006(2)
NB Hippo S2_007(2)
ME7 Hippo S2_006
NB Hippo S2_826
ME7 Hippo S2_005(2)
NB Hippo S2_950(3)
NB Hippo S2_001(3)
NB Hippo S2_950(2)
NB Hippo S2_G 818D
NB Hippo S2_F 775D
NB Hippo S2_010
NB Hippo S2_009
NB Hippo S2_951(3)
NB Hippo S2_009(2)
NB Hippo S2_826(2)
NB Hippo S2_951(2)
NB Hippo S2_001(2)
NB Hippo S2_007
NB Hippo S2_010(2)
2.0
1.5
1.0
0.5
0.0

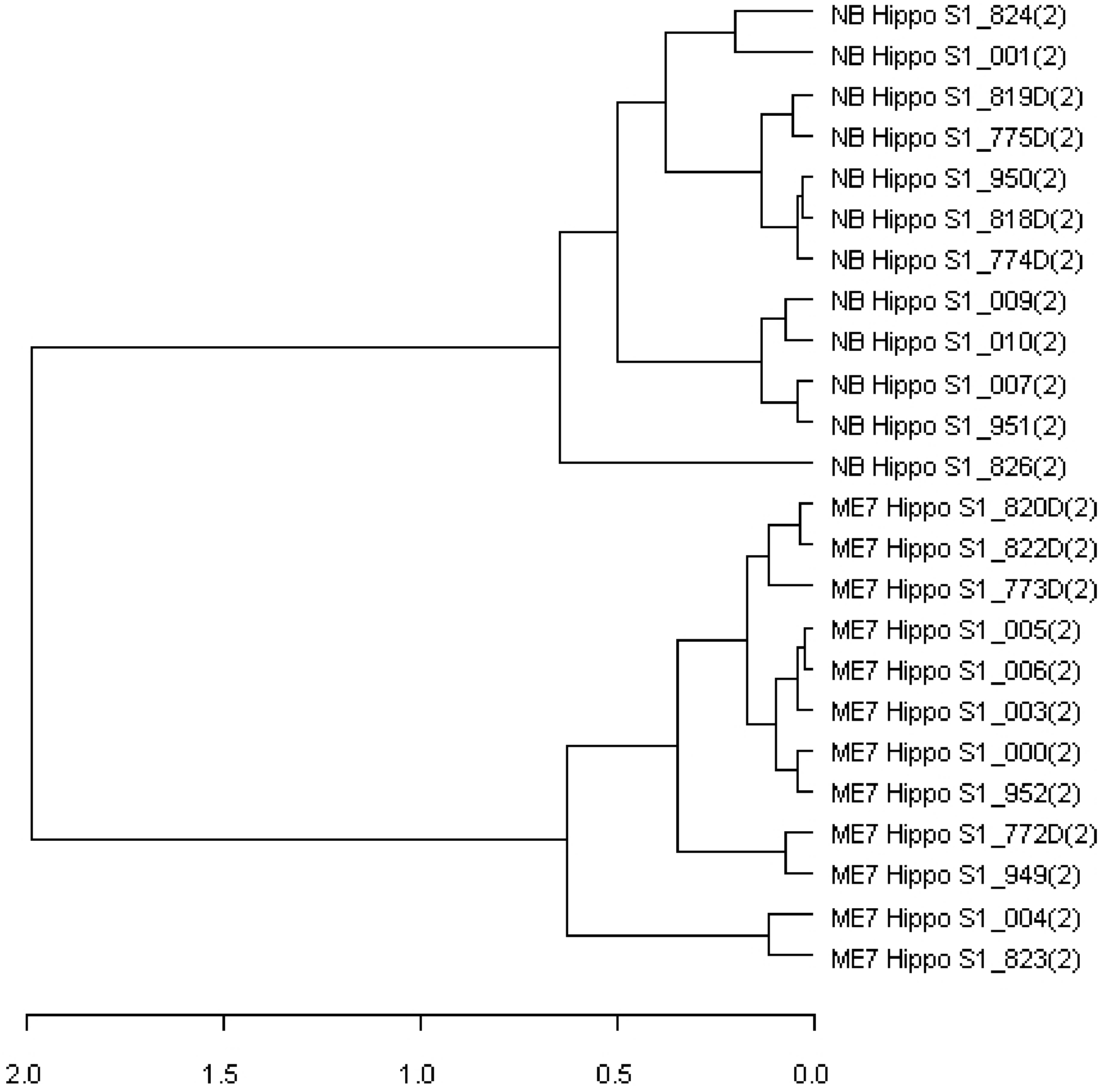
NB Hippo S1 _824(2)
NB Hippo S1 _001(2)
NB Hippo S1 _819D(2)
NB Hippo S1 _775D(2)
NB Hippo S1 _950(2)
NB Hippo S1 _818D(2)
NB Hippo S1 _774D(2)
NB Hippo S1 _009(2)
NB Hippo S1 _010(2)
NB Hippo S1 _007(2)
NB Hippo S1 _951(2)
NB Hippo S1 _826(2)
ME7 Hippo S1 _820D(2)
ME7 Hippo S1 _822D(2)
ME7 Hippo S1 _773D(2)
ME7 Hippo S1 _005(2)
ME7 Hippo S1 _006(2)
ME7 Hippo S1 _003(2)
ME7 Hippo S1 _000(2)
ME7 Hippo S1 _952(2)
ME7 Hippo S1 _772D(2)
ME7 Hippo S1 _949(2)
ME7 Hippo S1 _004(2)
ME7 Hippo S1 _823(2)
2.0
1.5
1.0
0.5
0.0

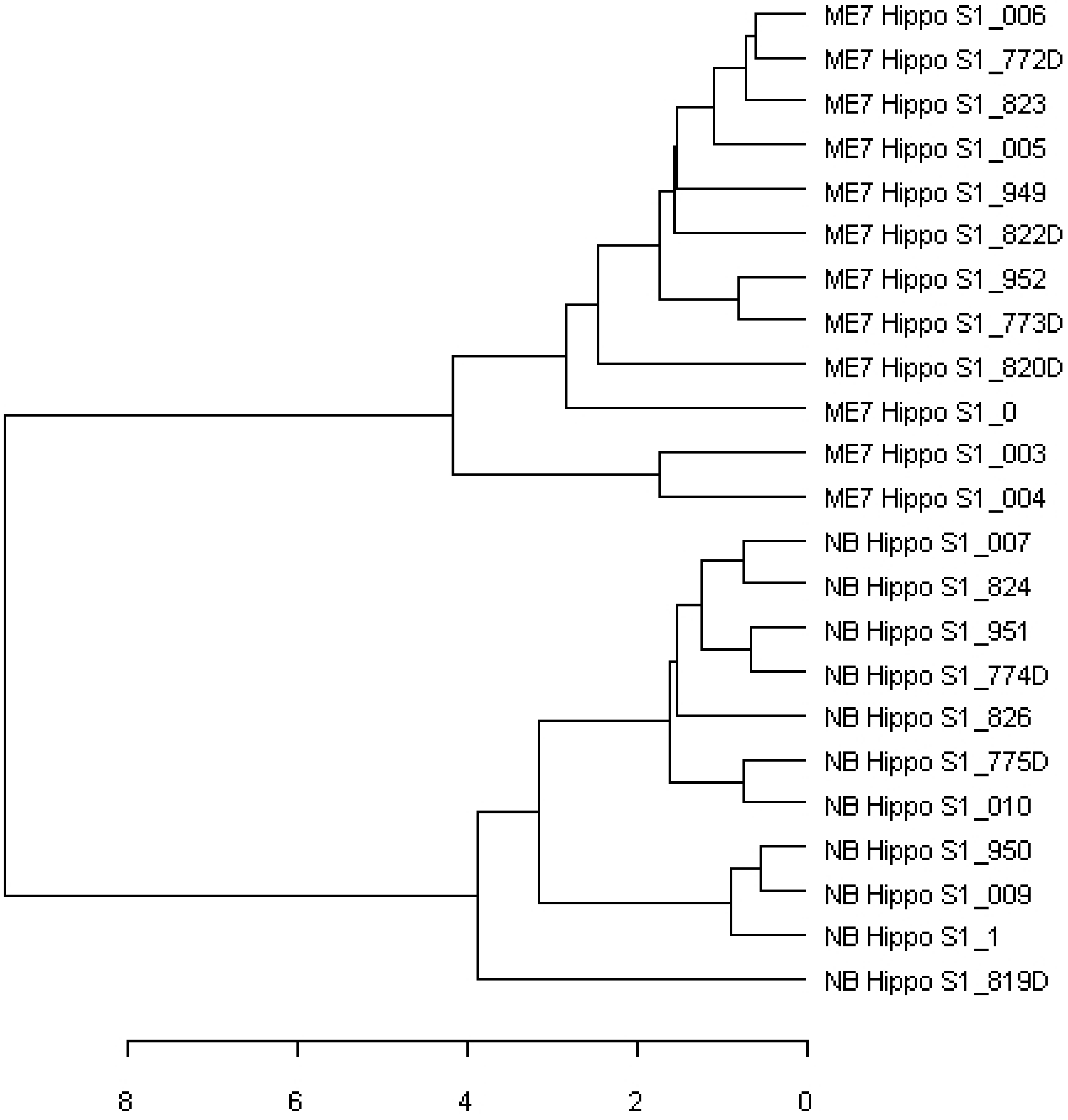

ME7 Hippo S1 _006
ME7 Hippo S1 _772D
ME7 Hippo S1 _823
ME7 Hippo S1 _005
ME7 Hippo S1 _949
ME7 Hippo S1 _822D
ME7 Hippo S1 _952
ME7 Hippo S1 _773D
ME7 Hippo S1 _820D
ME7 Hippo S1 _0
ME7 Hippo S1 _003
ME7 Hippo S1 _004
NB Hippo S1 _007
NB Hippo S1 _824
NB Hippo S1 _951
NB Hippo S1 _774D
NB Hippo S1 _826
NB Hippo S1 _775D
NB Hippo S1 _010
NB Hippo S1 _950
NB Hippo S1 _009
NB Hippo S1 _1
NB Hippo S1 _819D
8
6
4
2
0

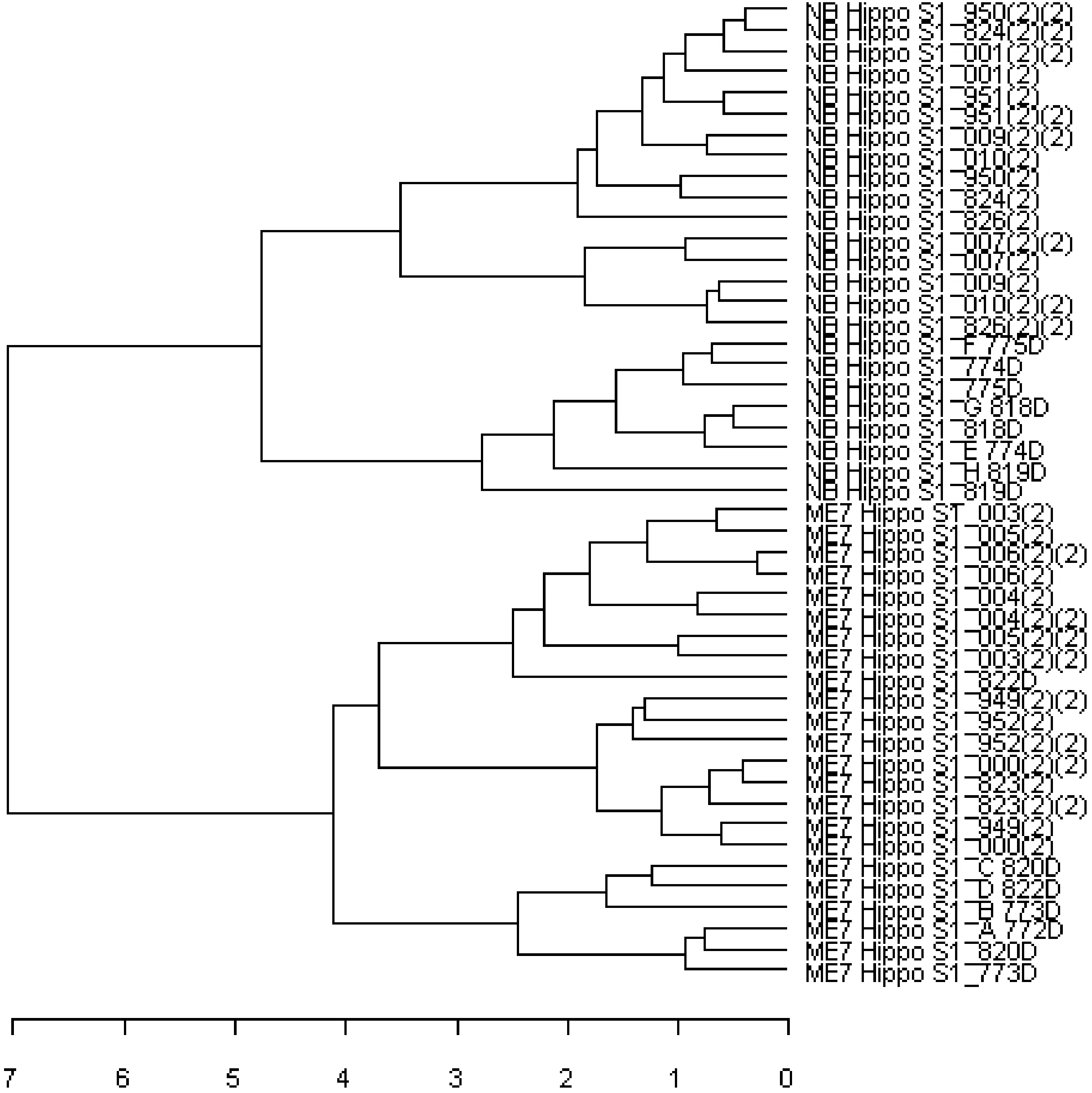
NB Hippo S1_950(2)(2)
NB Hippo S1_824(2)(2)
NB Hippo S1_001(2)(2)
NB Hippo S1_001(2)
NB Hippo S1_951(2)
NB Hippo S1_951(2)(2)
NB Hippo S1_009(2)(2)
NB Hippo S1_010(2)
NB Hippo S1_950(2)
NB Hippo S1_824(2)
NB Hippo S1_826(2)
NB Hippo S1_007(2)(2)
NB Hippo S1_007(2)
NB Hippo S1_009(2)
NB Hippo S1_010(2)(2)
NB Hippo S1_826(2)(2)
NB Hippo S1_F 775D
NB Hippo S1_774D
NB Hippo S1_775D
NB Hippo S1_G 818D
NB Hippo S1_818D
NB Hippo S1_E 774D
NB Hippo S1_H 819D
NB Hippo S1_819D
ME7 Hippo S1_003(2)
ME7 Hippo S1_005(2)
ME7 Hippo S1_006(2)(2)
ME7 Hippo S1_006(2)
ME7 Hippo S1_004(2)
ME7 Hippo S1_004(2)(2)
ME7 Hippo S1_005(2)(2)
ME7 Hippo S1_003(2)(2)
ME7 Hippo S1_822D
ME7 Hippo S1_949(2)(2)
ME7 Hippo S1_952(2)
ME7 Hippo S1_952(2)(2)
ME7 Hippo S1_000(2)(2)
ME7 Hippo S1_823(2)
ME7 Hippo S1_823(2)(2)
ME7 Hippo S1_949(2)
ME7 Hippo S1_000(2)
ME7 Hippo S1_C 820D
ME7 Hippo S1_D 822D
ME7 Hippo S1_B 773D
ME7 Hippo S1_A 772D
ME7 Hippo S1_820D
ME7 Hippo S1_773D
7
6
5
4
3
2
1
0

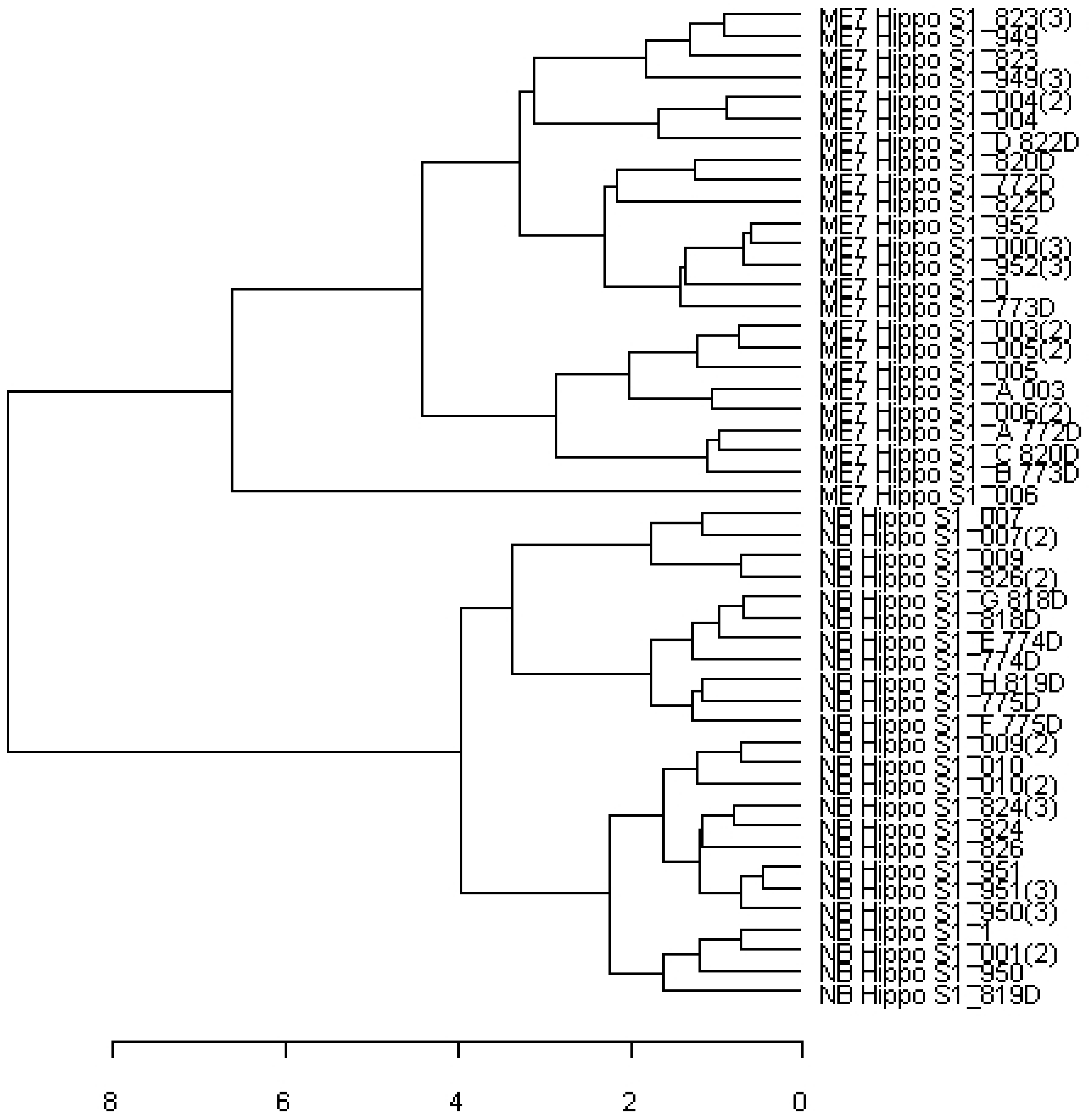
ME7 Hippo S1_823(3)
ME7 Hippo S1_949
ME7 Hippo S1_823
ME7 Hippo S1_949(3)
ME7 Hippo S1_004(2)
ME7 Hippo S1_004
ME7 Hippo S1_D 822D
ME7 Hippo S1_820D
ME7 Hippo S1_772D
ME7 Hippo S1_822D
ME7 Hippo S1_952
ME7 Hippo S1_000(3)
ME7 Hippo S1_952(3)
ME7 Hippo S1_0
ME7 Hippo S1_773D
ME7 Hippo S1_003(2)
ME7 Hippo S1_005(2)
ME7 Hippo S1_005
ME7 Hippo S1_A 003
ME7 Hippo S1_006(2)
ME7 Hippo S1_A 772D
ME7 Hippo S1_C 820D
ME7 Hippo S1_B 773D
ME7 Hippo S1_006
NB Hippo S1_007
NB Hippo S1_007(2)
NB Hippo S1_009
NB Hippo S1_826(2)
NB Hippo S1_G 818D
NB Hippo S1_818D
NB Hippo S1_E 774D
NB Hippo S1_774D
NB Hippo S1_H 819D
NB Hippo S1_775D
NB Hippo S1_F 775D
NB Hippo S1_009(2)
NB Hippo S1_010
NB Hippo S1_010(2)
NB Hippo S1_824(3)
NB Hippo S1_824
NB Hippo S1_826
NB Hippo S1_951
NB Hippo S1_951(3)
NB Hippo S1_950(3)
NB Hippo S1_1
NB Hippo S1_001(2)
NB Hippo S1_950
NB Hippo S1_819D
8
6
4
2
0

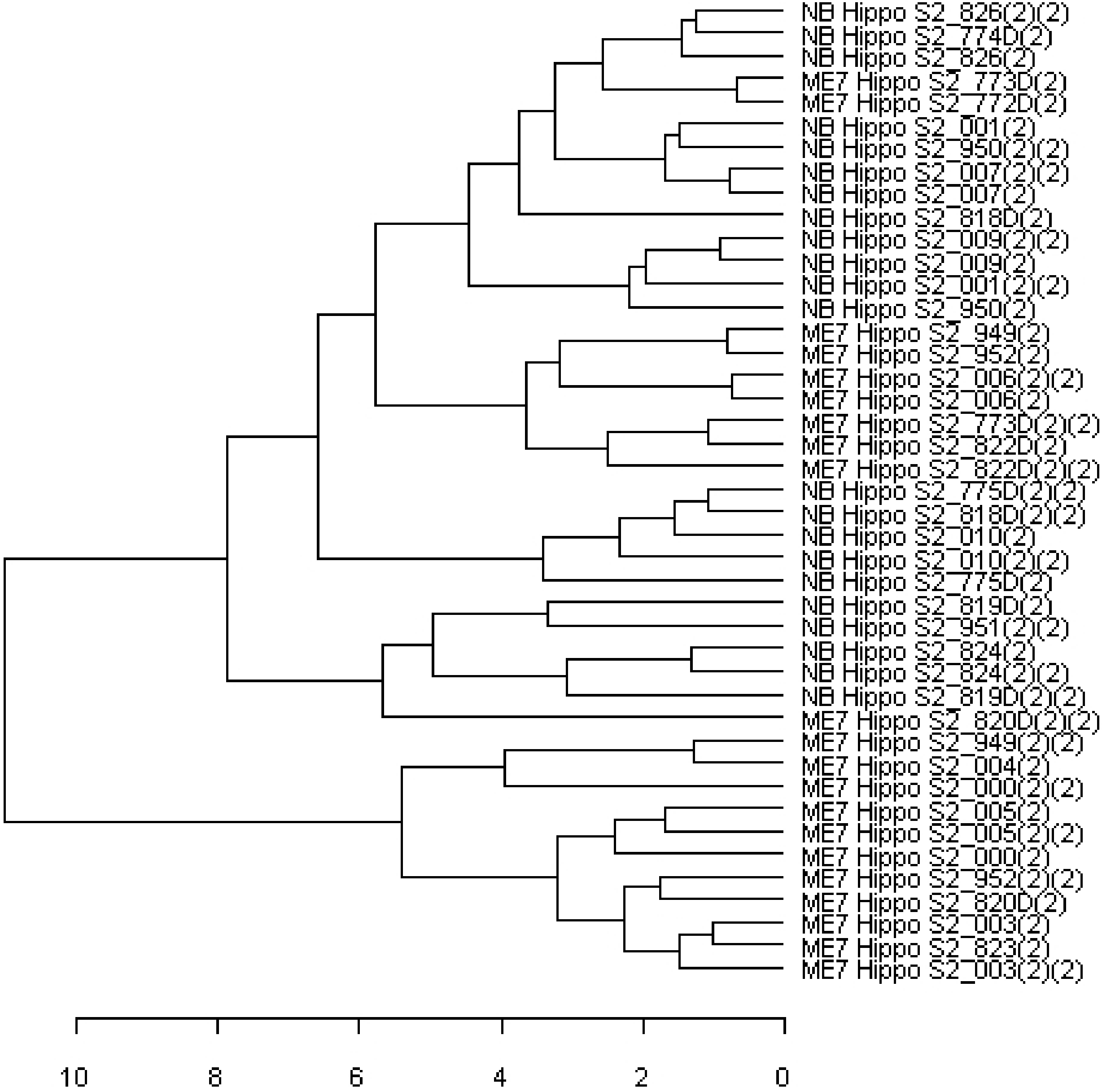

NB Hippo S2_826(2)(2)
NB Hippo S2_774D(2)
NB Hippo S2_826(2)
ME7 Hippo S2_773D(2)
ME7 Hippo S2_772D(2)
NB Hippo S2_001(2)
NB Hippo S2_950(2)(2)
NB Hippo S2_007(2)(2)
NB Hippo S2_007(2)
NB Hippo S2_818D(2)
NB Hippo S2_009(2)(2)
NB Hippo S2_009(2)
NB Hippo S2_001(2)(2)
NB Hippo S2_950(2)
ME7 Hippo S2_949(2)
ME7 Hippo S2_952(2)
ME7 Hippo S2_006(2)(2)
ME7 Hippo S2_006(2)
ME7 Hippo S2_773D(2)(2)
ME7 Hippo S2_822D(2)
ME7 Hippo S2_822D(2)(2)
NB Hippo S2_775D(2)(2)
NB Hippo S2_818D(2)(2)
NB Hippo S2_010(2)
NB Hippo S2_010(2)(2)
NB Hippo S2_775D(2)
NB Hippo S2_819D(2)
NB Hippo S2_951(2)(2)
NB Hippo S2_824(2)
NB Hippo S2_824(2)(2)
NB Hippo S2_819D(2)(2)
ME7 Hippo S2_820D(2)(2)
ME7 Hippo S2_949(2)(2)
ME7 Hippo S2_004(2)
ME7 Hippo S2_000(2)(2)
ME7 Hippo S2_005(2)
ME7 Hippo S2_005(2)(2)
ME7 Hippo S2_000(2)
ME7 Hippo S2_952(2)(2)
ME7 Hippo S2_820D(2)
ME7 Hippo S2_003(2)
ME7 Hippo S2_823(2)
ME7 Hippo S2_003(2)(2)
10
8
6
4
2
0

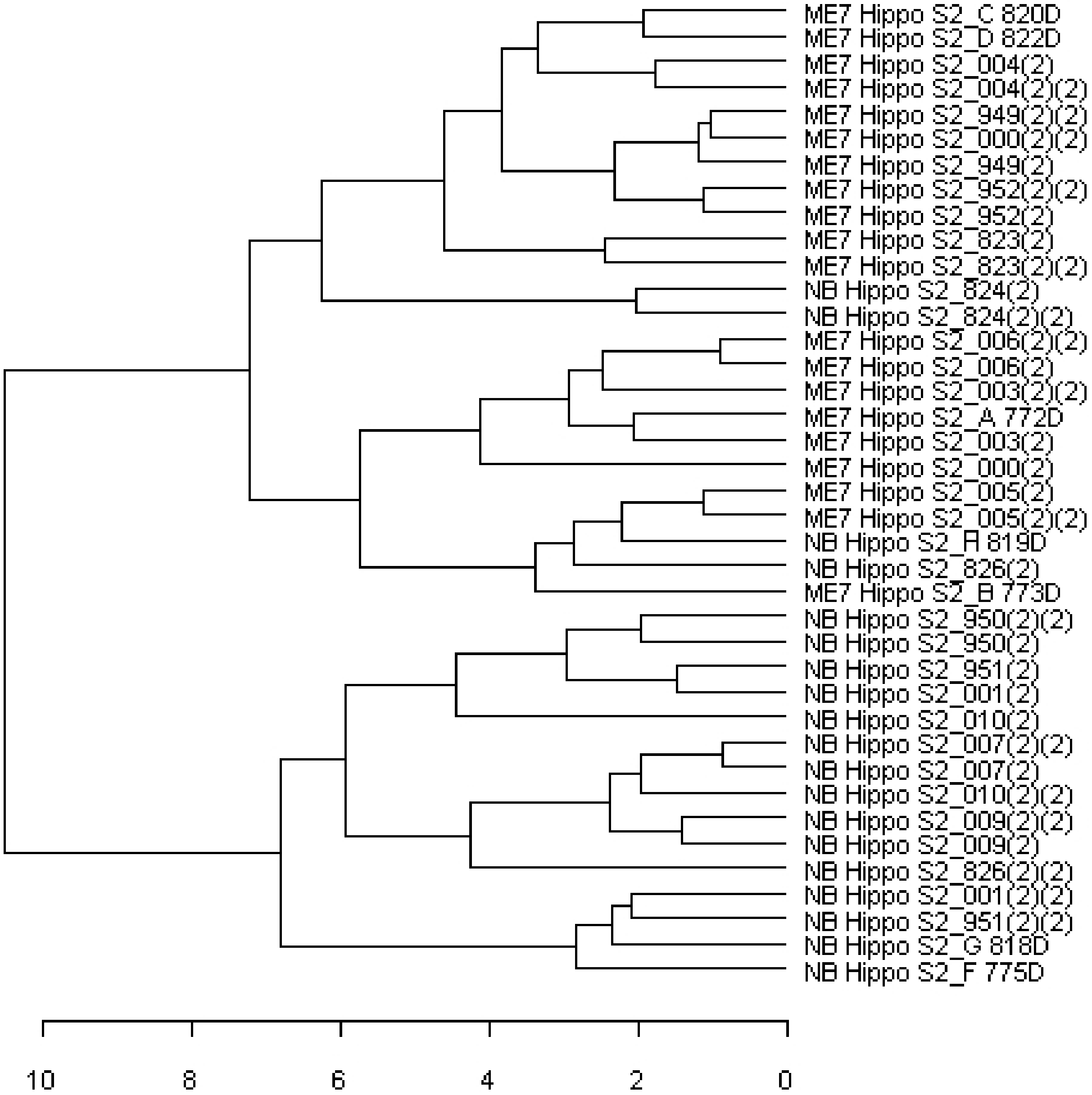
ME7 Hippo S2_C 820D
ME7 Hippo S2_D 822D
ME7 Hippo S2_004(2)
ME7 Hippo S2_004(2)(2)
ME7 Hippo S2_949(2)(2)
ME7 Hippo S2_000(2)(2)
ME7 Hippo S2_949(2)
ME7 Hippo S2_952(2)(2)
ME7 Hippo S2_952(2)
ME7 Hippo S2_823(2)
ME7 Hippo S2_823(2)(2)
NB Hippo S2_824(2)
NB Hippo S2_824(2)(2)
ME7 Hippo S2_006(2)(2)
ME7 Hippo S2_006(2)
ME7 Hippo S2_003(2)(2)
ME7 Hippo S2_A 772D
ME7 Hippo S2_003(2)
ME7 Hippo S2_000(2)
ME7 Hippo S2_005(2)
ME7 Hippo S2_005(2)(2)
NB Hippo S2_H 819D
NB Hippo S2_826(2)
ME7 Hippo S2_B 773D
NB Hippo S2_950(2)(2)
NB Hippo S2_950(2)
NB Hippo S2_951(2)
NB Hippo S2_001(2)
NB Hippo S2_010(2)
NB Hippo S2_007(2)(2)
NB Hippo S2_007(2)
NB Hippo S2_010(2)(2)
NB Hippo S2_009(2)(2)
NB Hippo S2_009(2)
NB Hippo S2_826(2)(2)
NB Hippo S2_001(2)(2)
NB Hippo S2_951(2)(2)
NB Hippo S2_G 818D
NB Hippo S2_F 775D
10
8
6
4
2
0

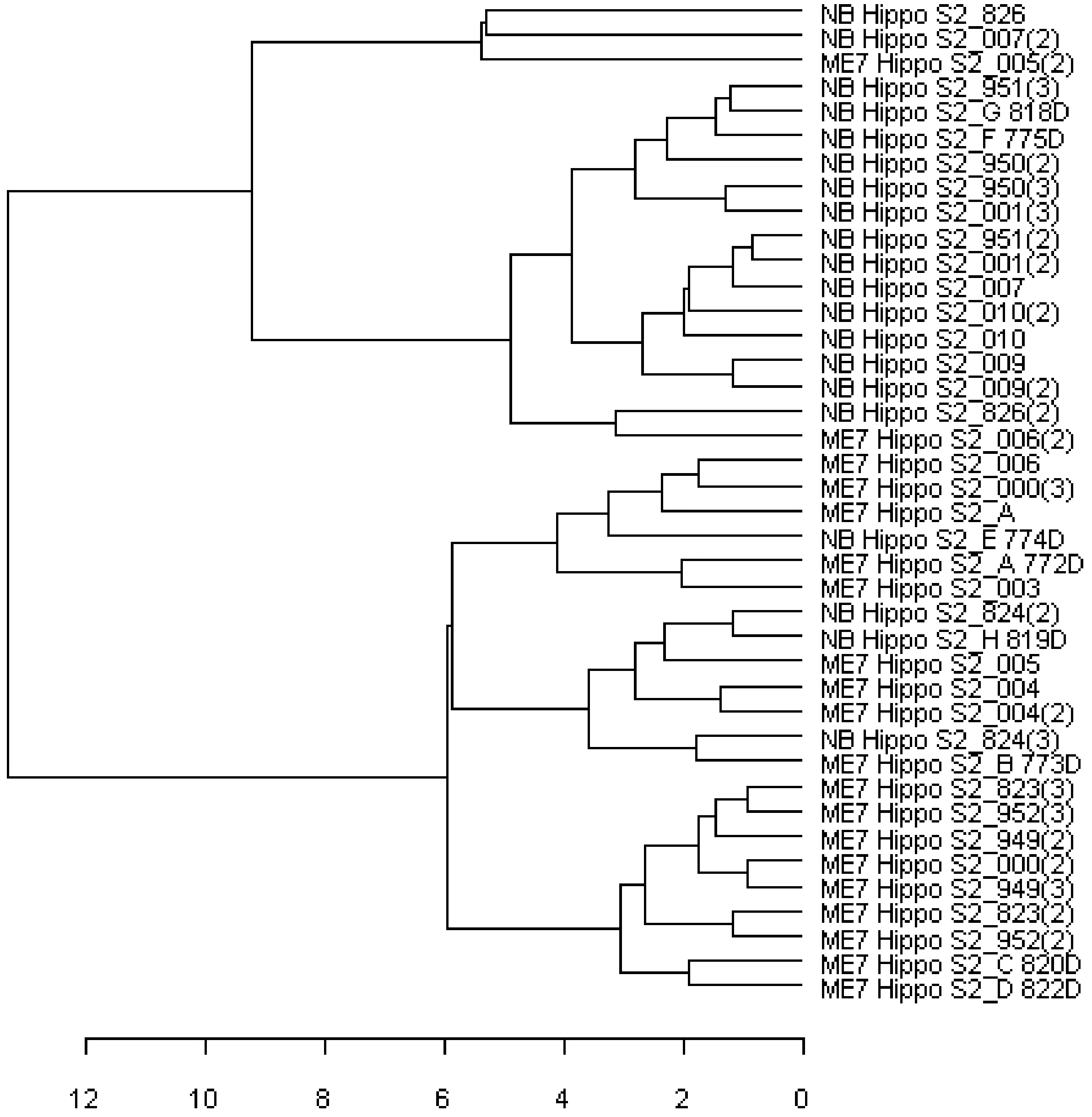
NB Hippo S2_826
NB Hippo S2_007(2)
ME7 Hippo S2_005(2)
NB Hippo S2_951(3)
NB Hippo S2_G 818D
NB Hippo S2_F 775D
NB Hippo S2_950(2)
NB Hippo S2_950(3)
NB Hippo S2_001(3)
NB Hippo S2_951(2)
NB Hippo S2_001(2)
NB Hippo S2_007
NB Hippo S2_010(2)
NB Hippo S2_010
NB Hippo S2_009
NB Hippo S2_009(2)
NB Hippo S2_826(2)
ME7 Hippo S2_006(2)
ME7 Hippo S2_006
ME7 Hippo S2_000(3)
ME7 Hippo S2_A
NB Hippo S2_E 774D
ME7 Hippo S2_A 772D
ME7 Hippo S2_003
NB Hippo S2_824(2)
NB Hippo S2_H 819D
ME7 Hippo S2_005
ME7 Hippo S2_004
ME7 Hippo S2_004(2)
NB Hippo S2_824(3)
ME7 Hippo S2_B 773D
ME7 Hippo S2_823(3)
ME7 Hippo S2_952(3)
ME7 Hippo S2_949(2)
ME7 Hippo S2_000(2)
ME7 Hippo S2_949(3)
ME7 Hippo S2_823(2)
ME7 Hippo S2_952(2)
ME7 Hippo S2_C 820D
ME7 Hippo S2_D 822D
12
10
8
6
4
2
0

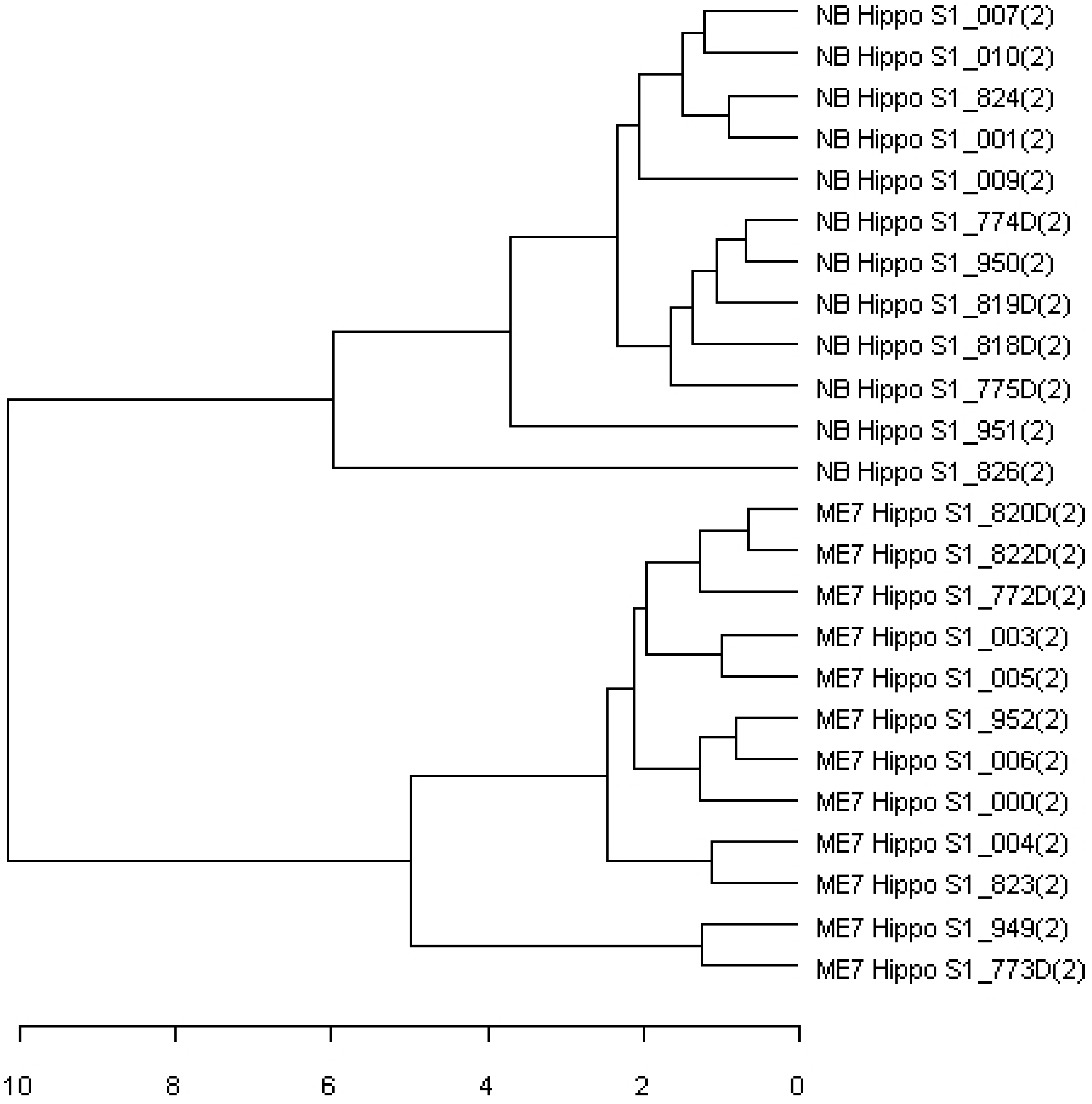
NB Hippo S1_007(2)
NB Hippo S1_010(2)
NB Hippo S1_824(2)
NB Hippo S1_001(2)
NB Hippo S1_009(2)
NB Hippo S1_774D(2)
NB Hippo S1_950(2)
NB Hippo S1_819D(2)
NB Hippo S1_818D(2)
NB Hippo S1_775D(2)
NB Hippo S1_951(2)
NB Hippo S1_826(2)
ME7 Hippo S1_820D(2)
ME7 Hippo S1_822D(2)
ME7 Hippo S1_772D(2)
ME7 Hippo S1_003(2)
ME7 Hippo S1_005(2)
ME7 Hippo S1_952(2)
ME7 Hippo S1_006(2)
ME7 Hippo S1_000(2)
ME7 Hippo S1_004(2)
ME7 Hippo S1_823(2)
ME7 Hippo S1_949(2)
ME7 Hippo S1_773D(2)
10
8
6
4
2
0

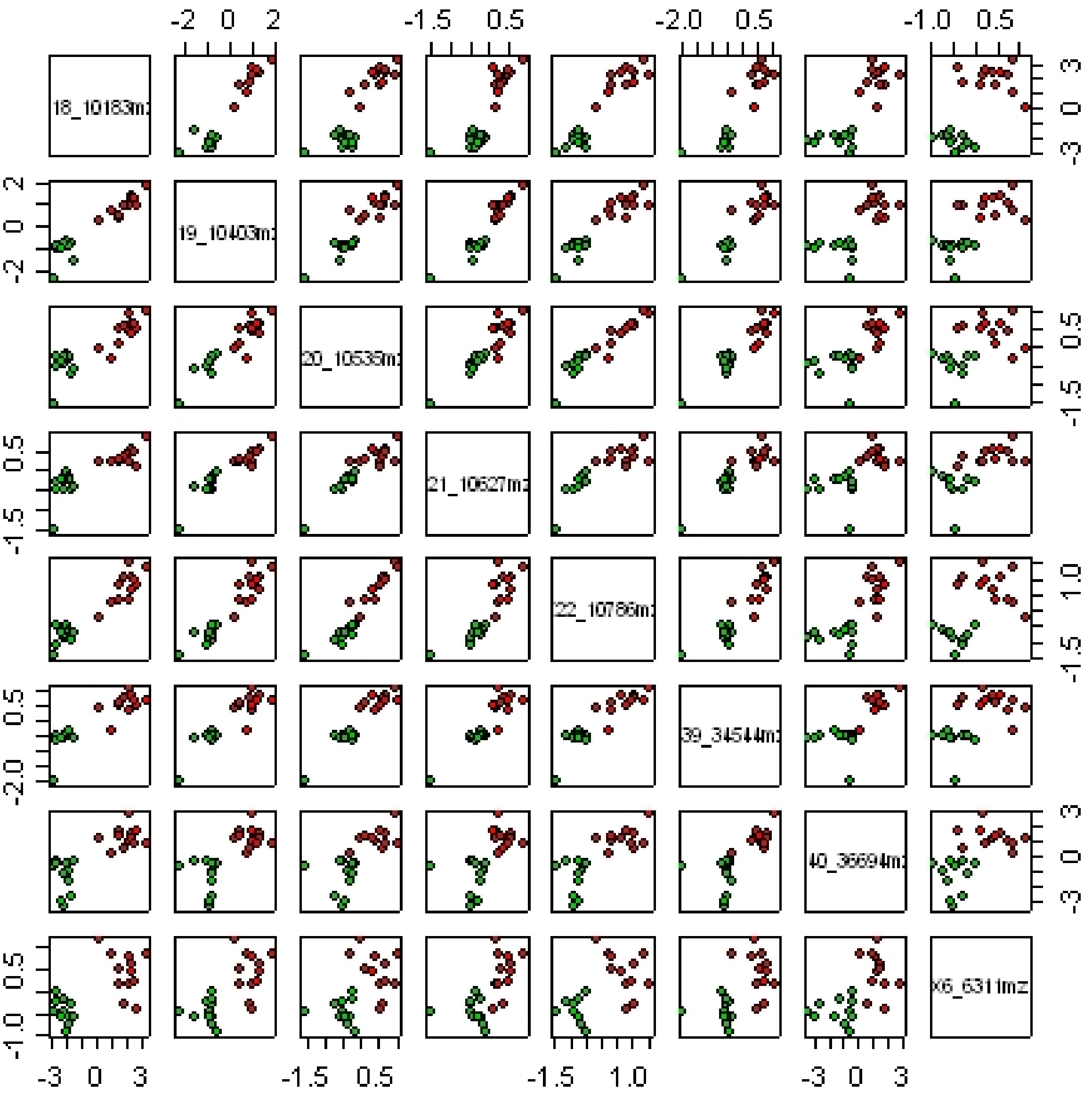
18_10183m
19_10403m
20_10535m
21_10627m
22_10786m
39_34544m
40_36694m
X6_6311mz

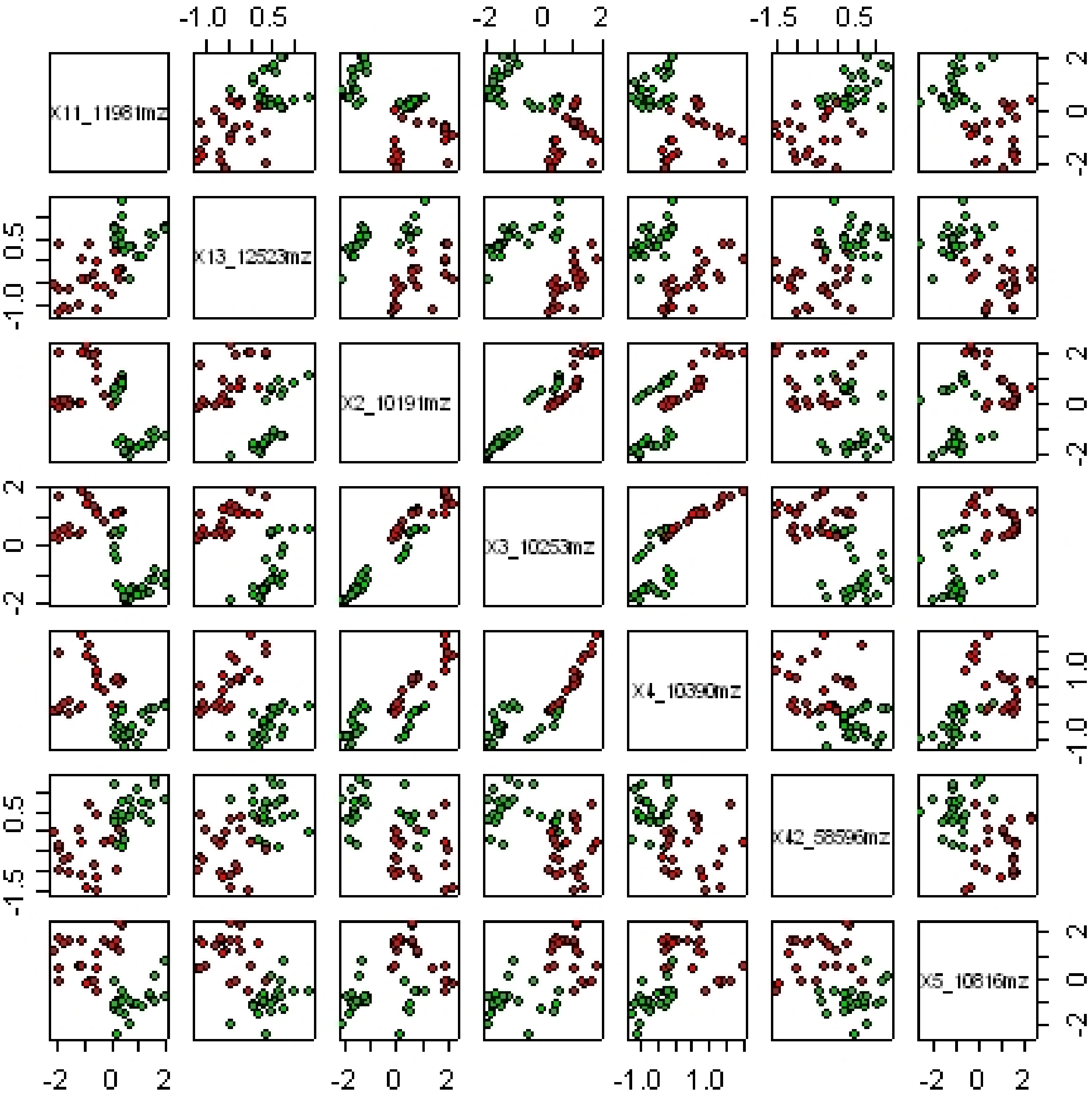

X11_11981mz
X13_12523mz
X2_10191mz
X3_10253mz
X4_10390mz
X42_58596mz
X5_10616mz

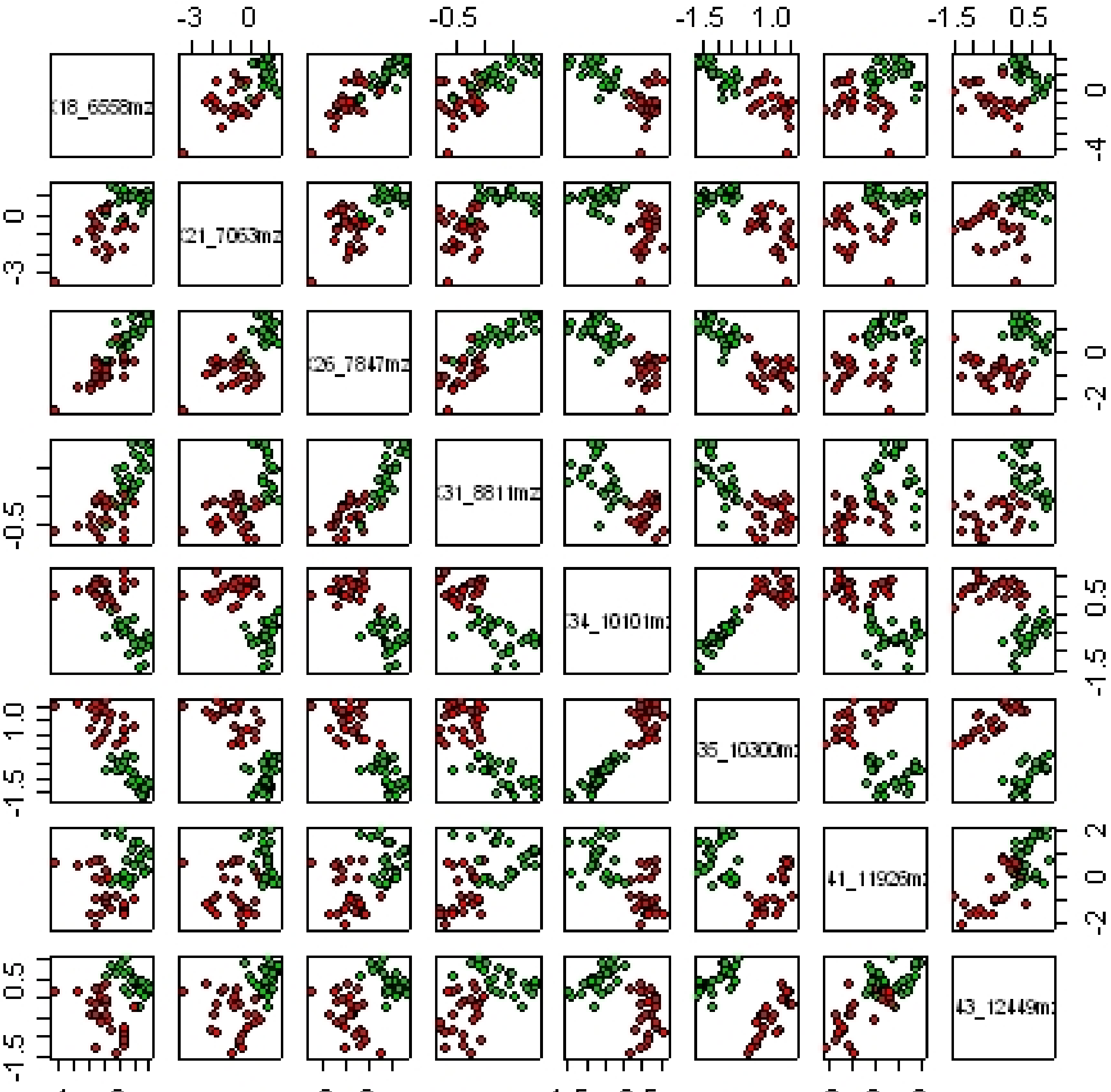
18_6558mz
21_7063mz
26_7847mz
31_8811mz
34_10101m
35_10300m
41_11926m
43_12449m

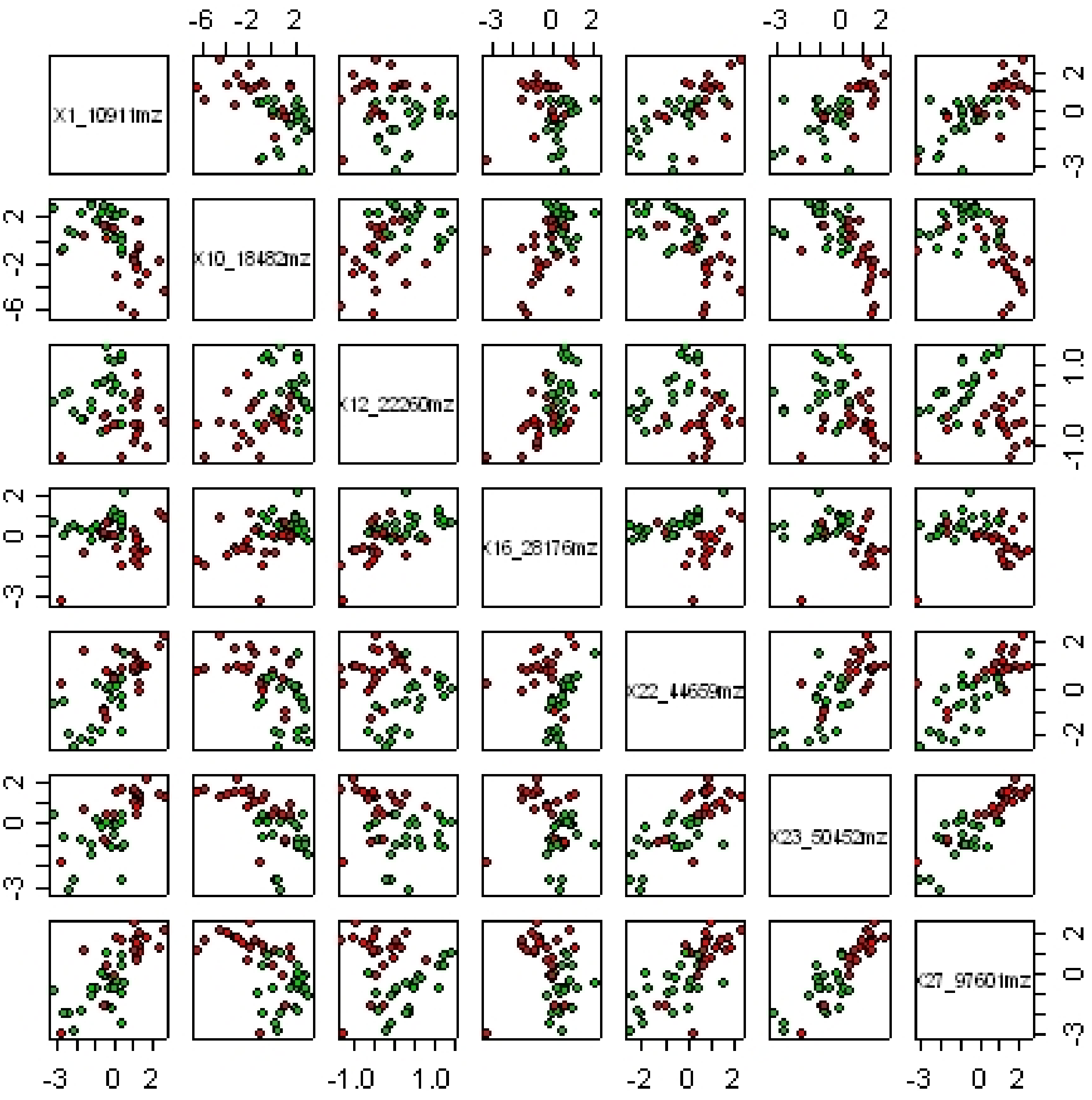
X1_10911mz
X10_18482mz
X12_22260mz
X16_28176mz
X22_44659mz
X23_50452mz
X27_97601mz

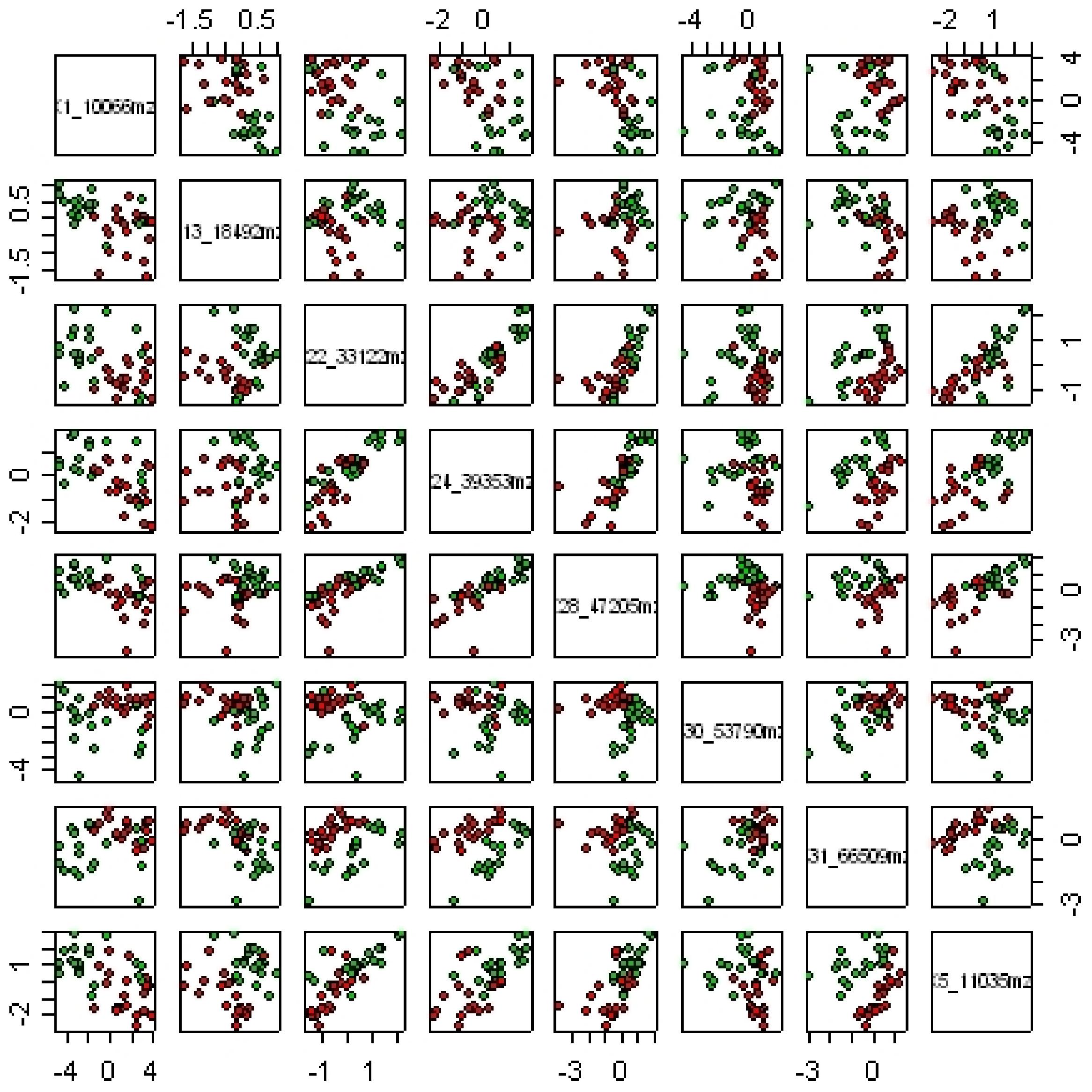

-1.5 0.5
-2 0
-4 0
-2 1
4 0 -4
0.5 -1.5
1 -1
0 -2
0 -3
0 -4
0 -3
1 -2
-4 0 4
-1 1
-3 0
-3 0

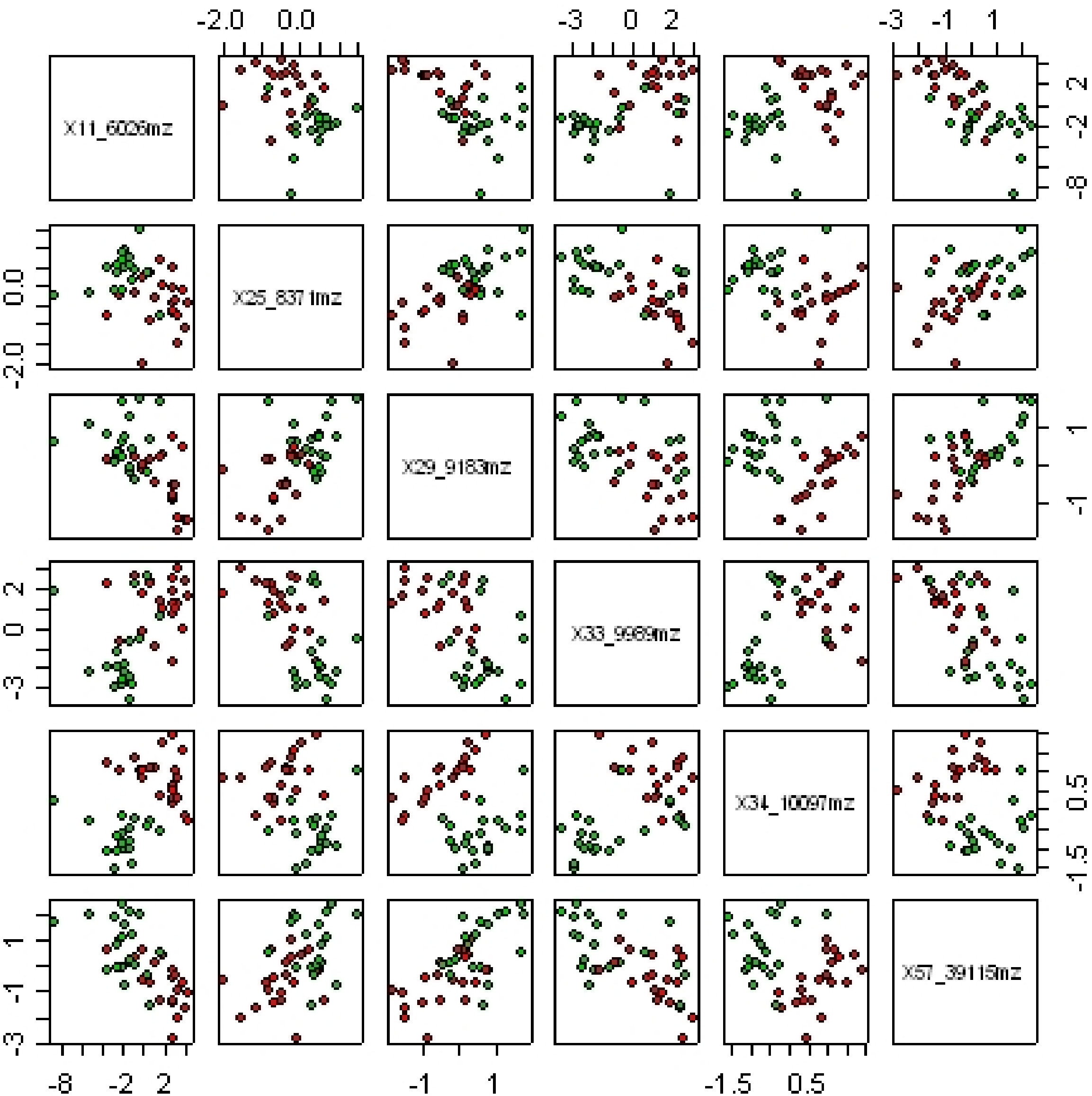

X11_6026mz
X25_8371mz
X29_9183mz
X33_9989mz
X34_10097mz
X57_39115mz
-2.0
0.0
-3
0
2
-3
-1
1
-8
-2
2
-1
1
-1.5
0.5

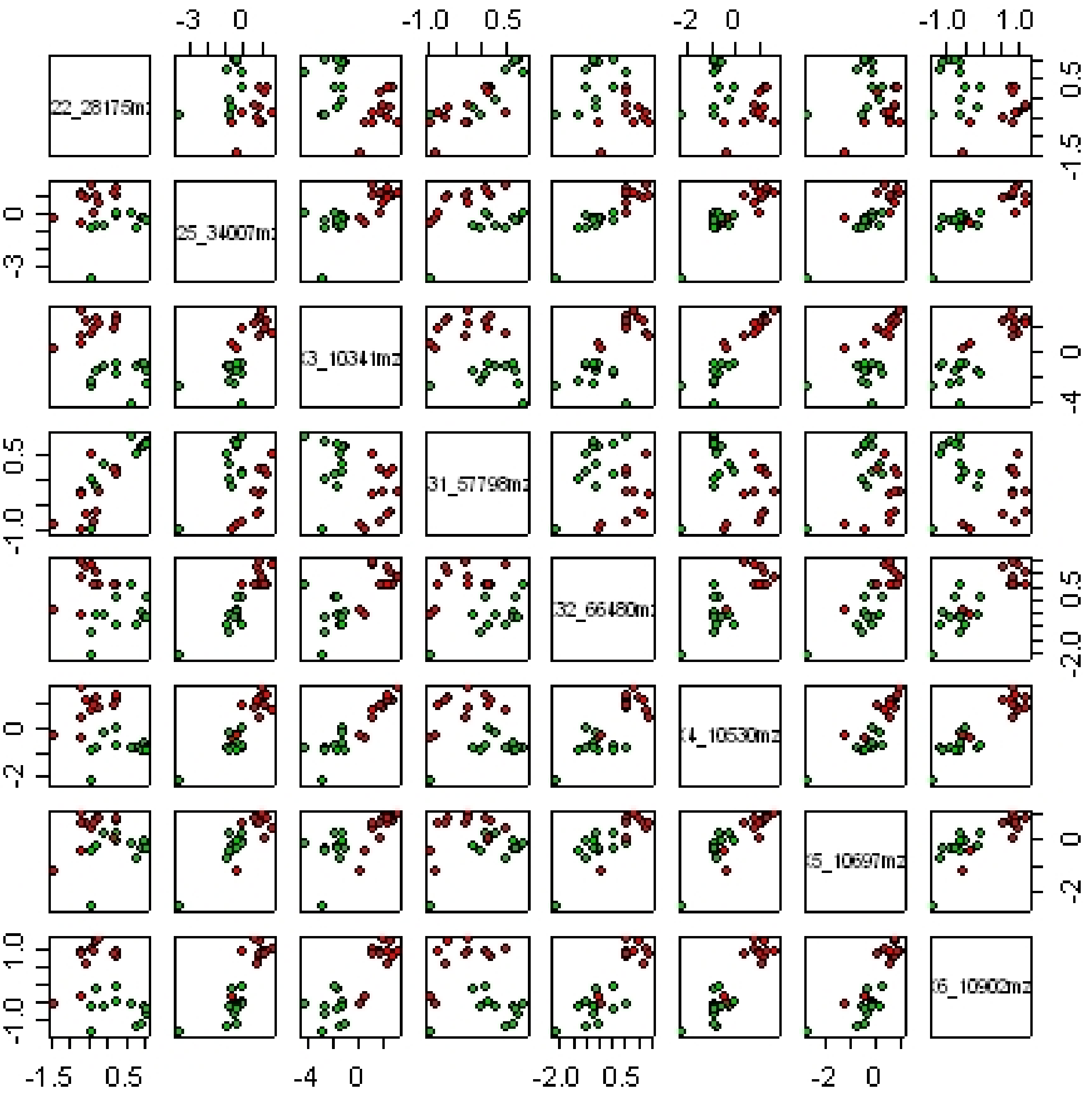

22_28175mz
25_34007mz
3_10341mz
31_57798mz
32_66480mz
4_10530mz
5_10697mz
6_10902mz

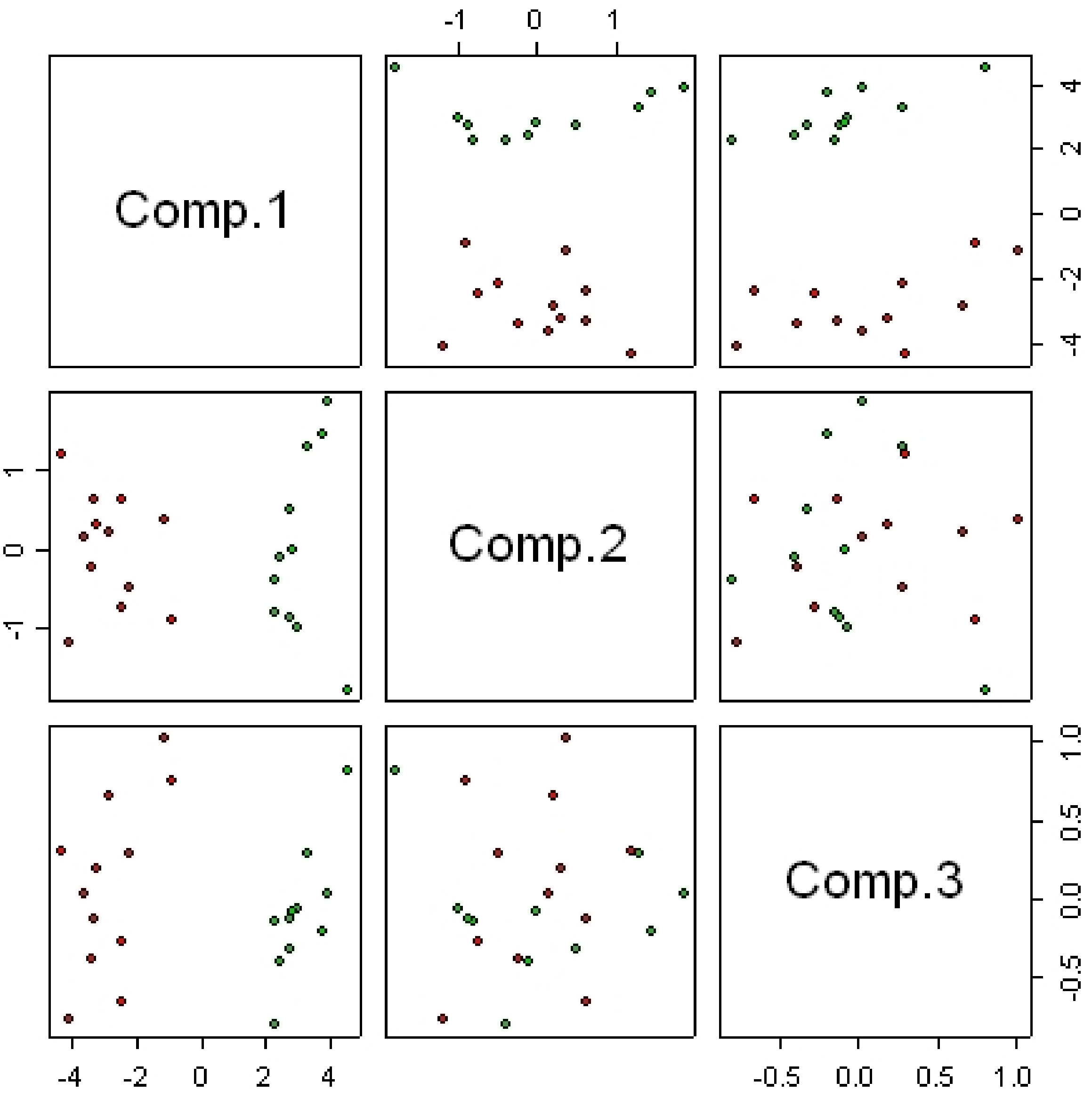
Comp.1
Comp.2
Comp.3
-1
0
1
4
2
0
-2
-4
1
0
-1
1.0
0.5
0.0
-0.5
-4
-2
0
2
4
-0.5
0.0
0.5
1.0

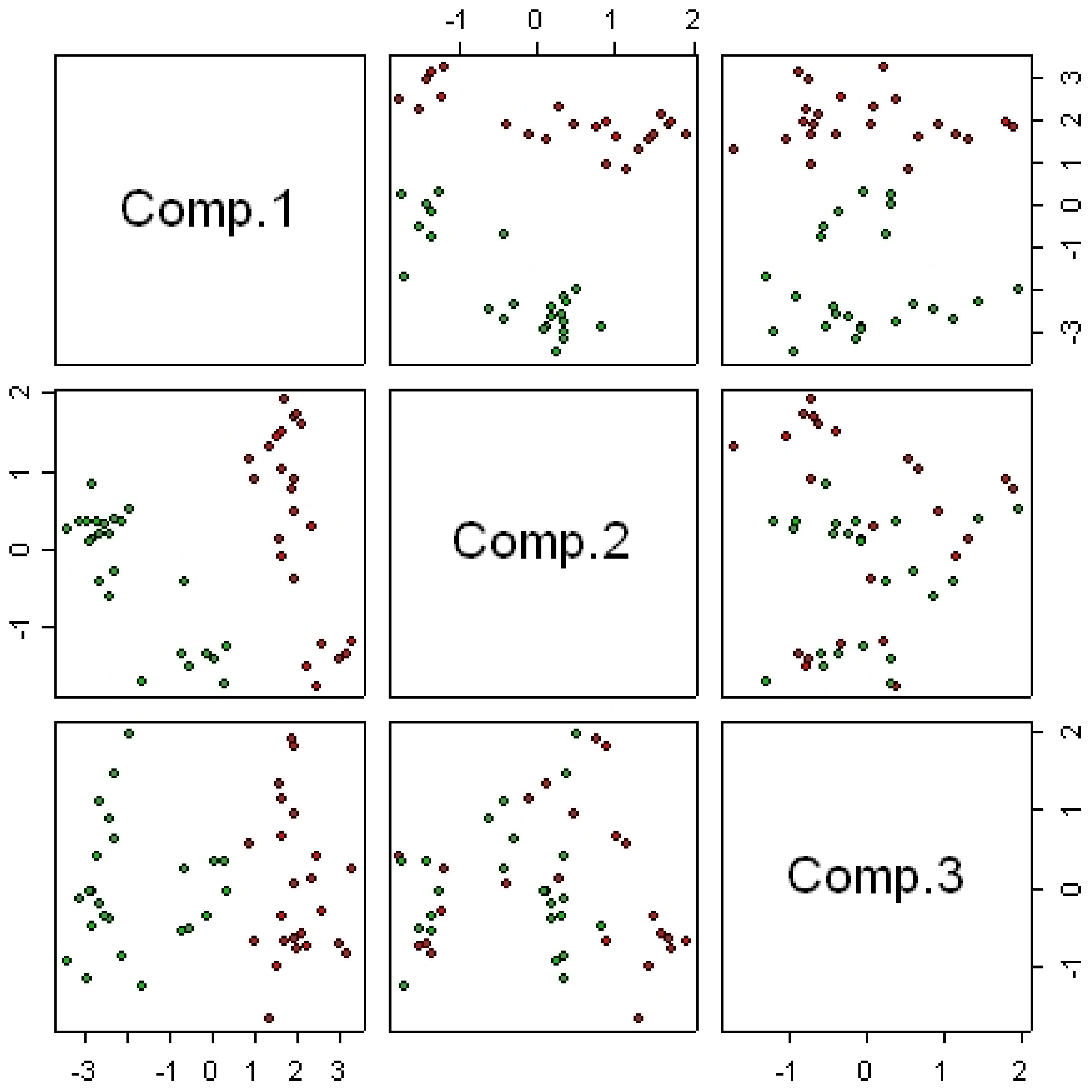

Comp.1
Comp.2
Comp.3
-1 0 1 2
-3 -1 0 1 2 3
-3 -1 0 1 2 3
-1 0 1 2
-1 0 1 2
-1 0 1 2

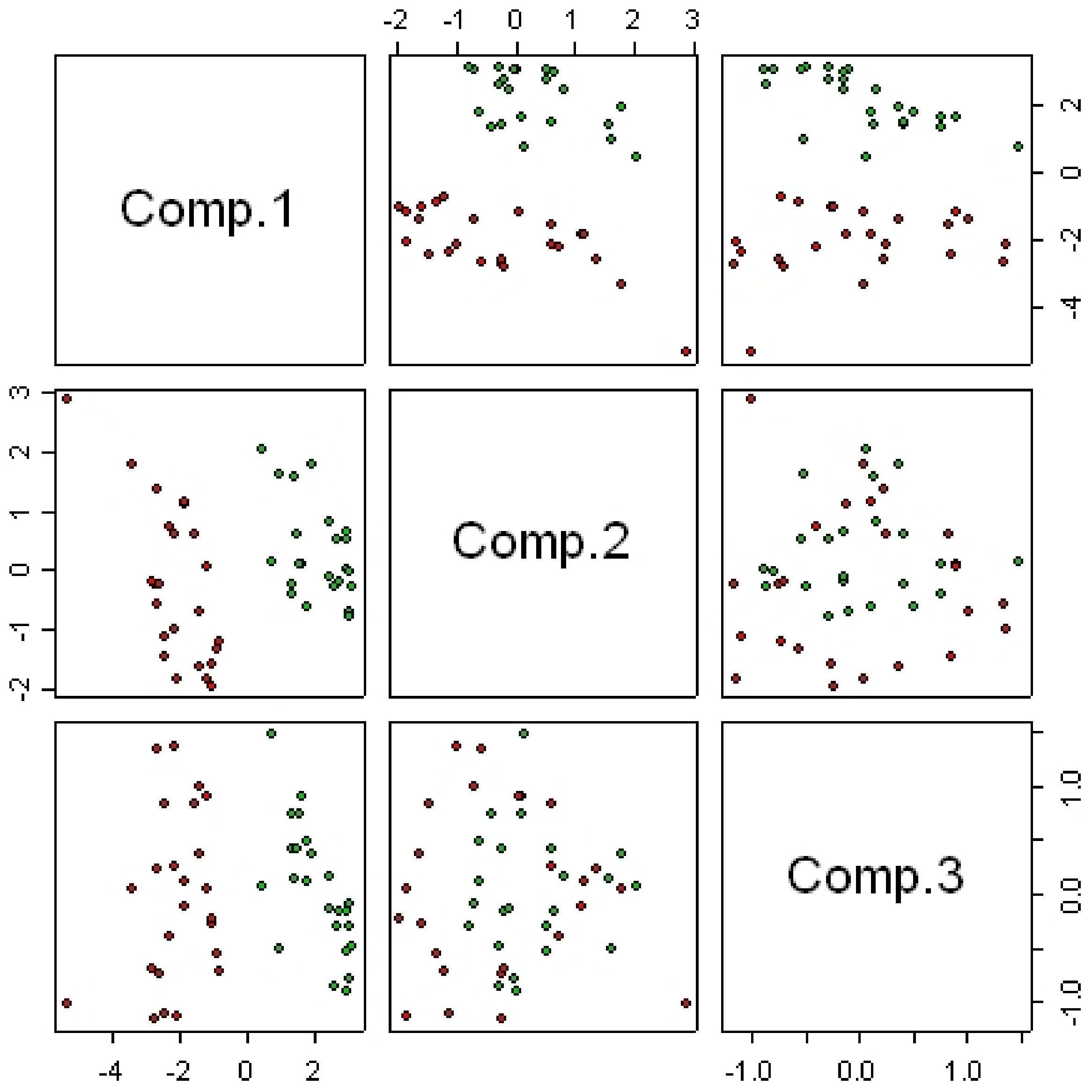

Comp.1
Comp.2
Comp.3
-2 -1 0 1 2 3
-4 -2 0 2
-1.0 0.0 1.0

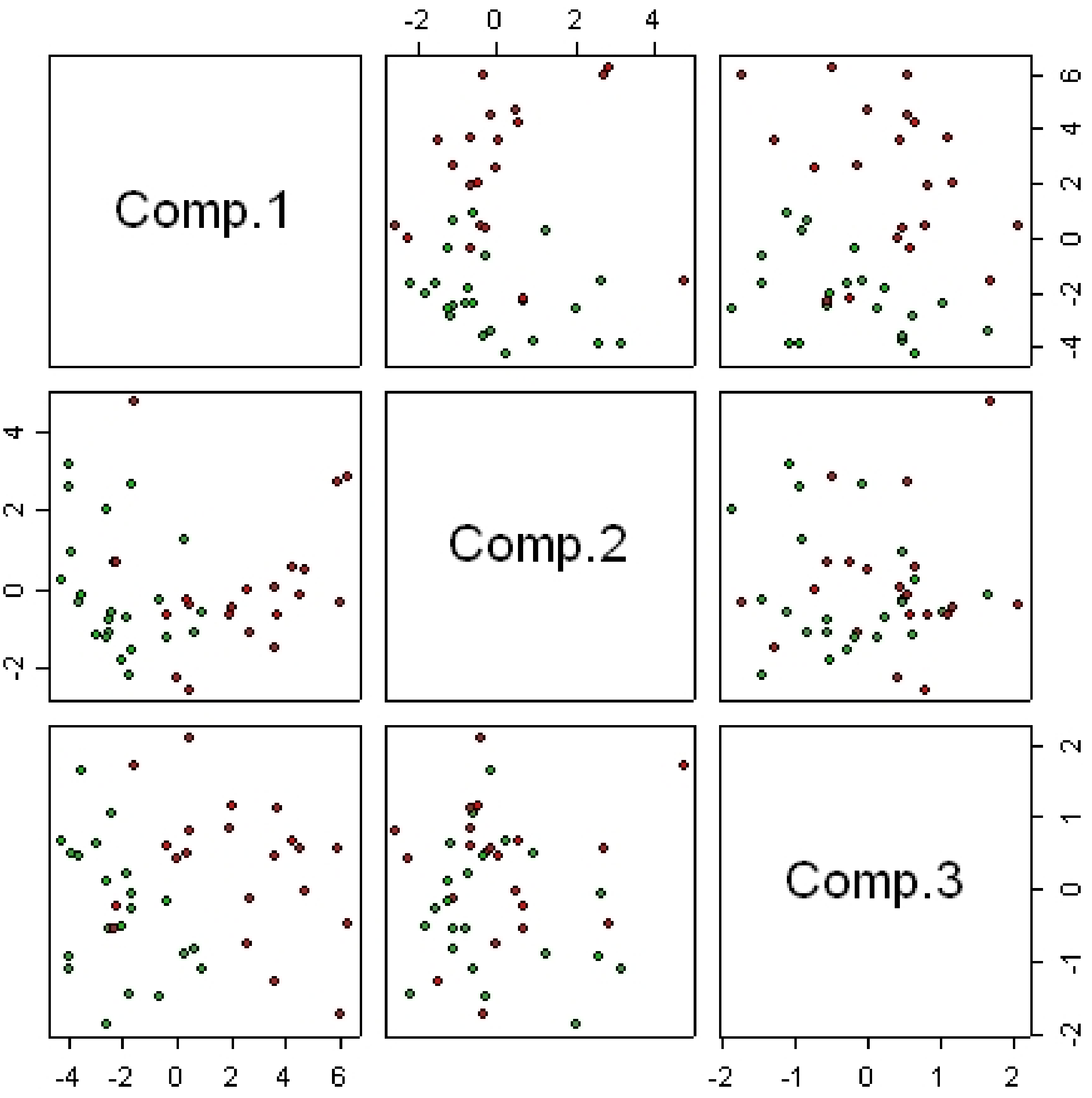
Comp.1
Comp.2
Comp.3
-2
0
2
4
6
-4
-2
0
2
4
6
-2
-1
0
1
2

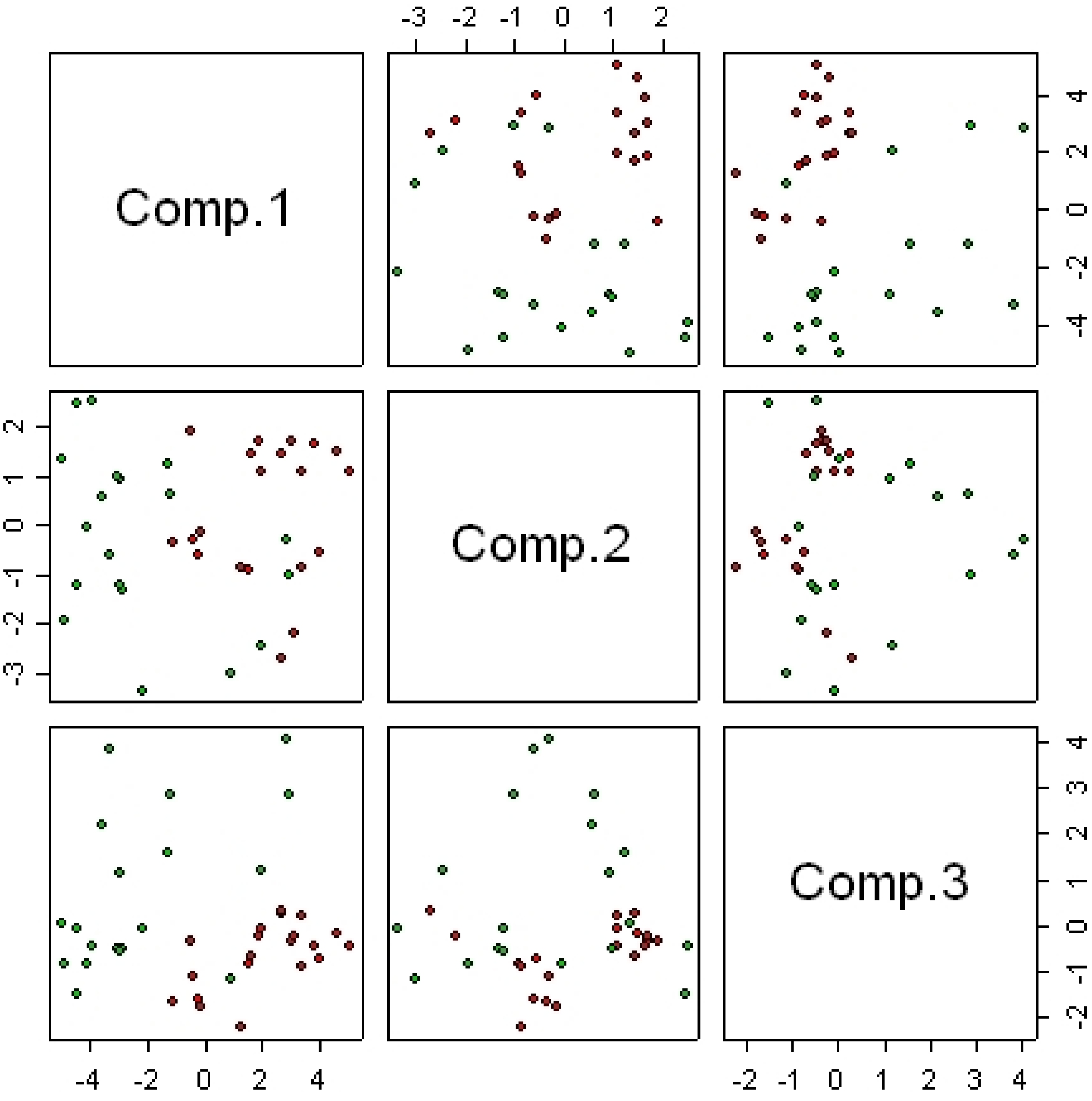

Comp.1
Comp.2
Comp.3

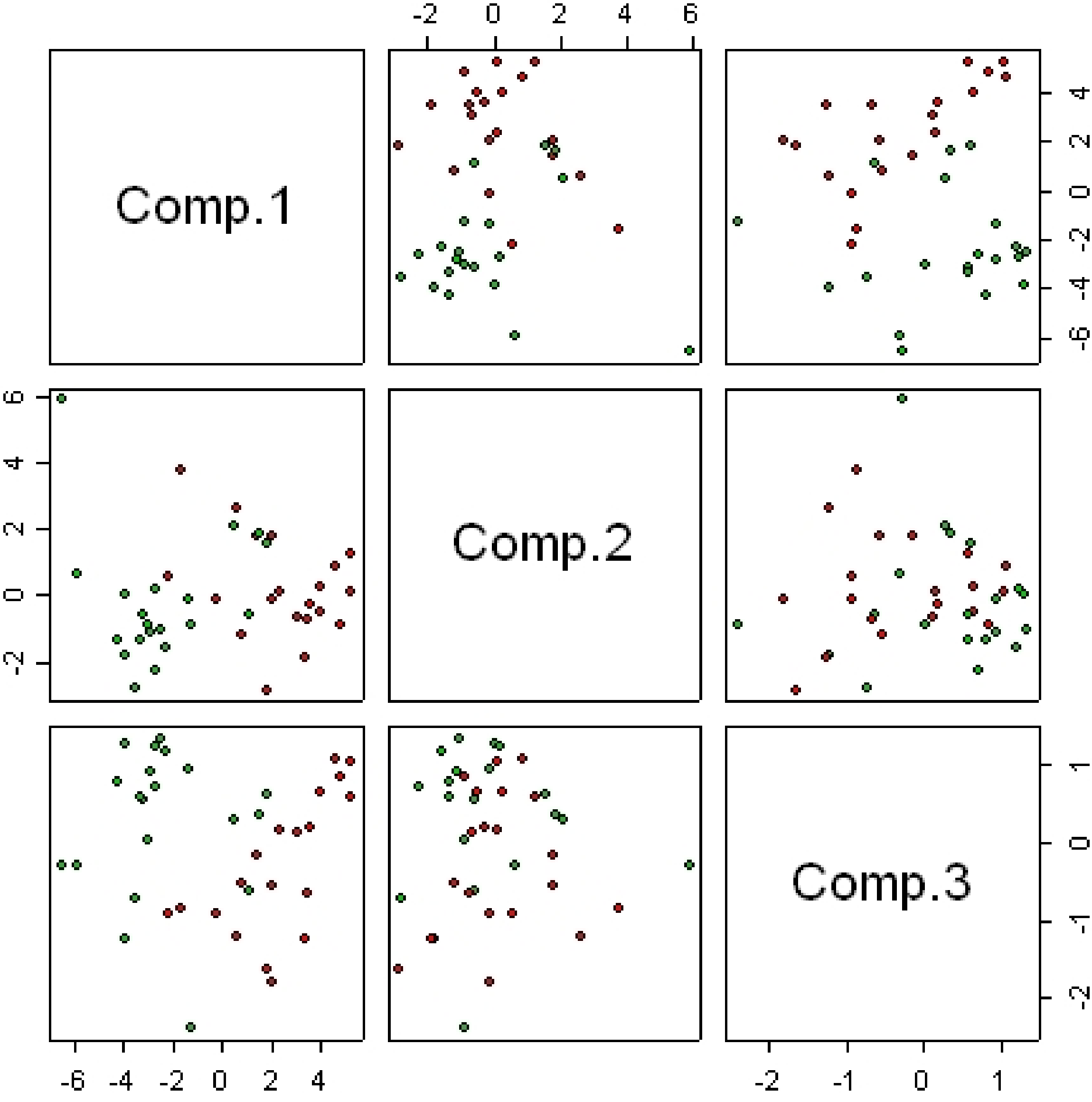
Comp.1
Comp.2
Comp.3
-2
0
2
4
6
-6
-4
-2
0
2
4
6
-6
-4
-2
0
2
4
-2
-1
0
1

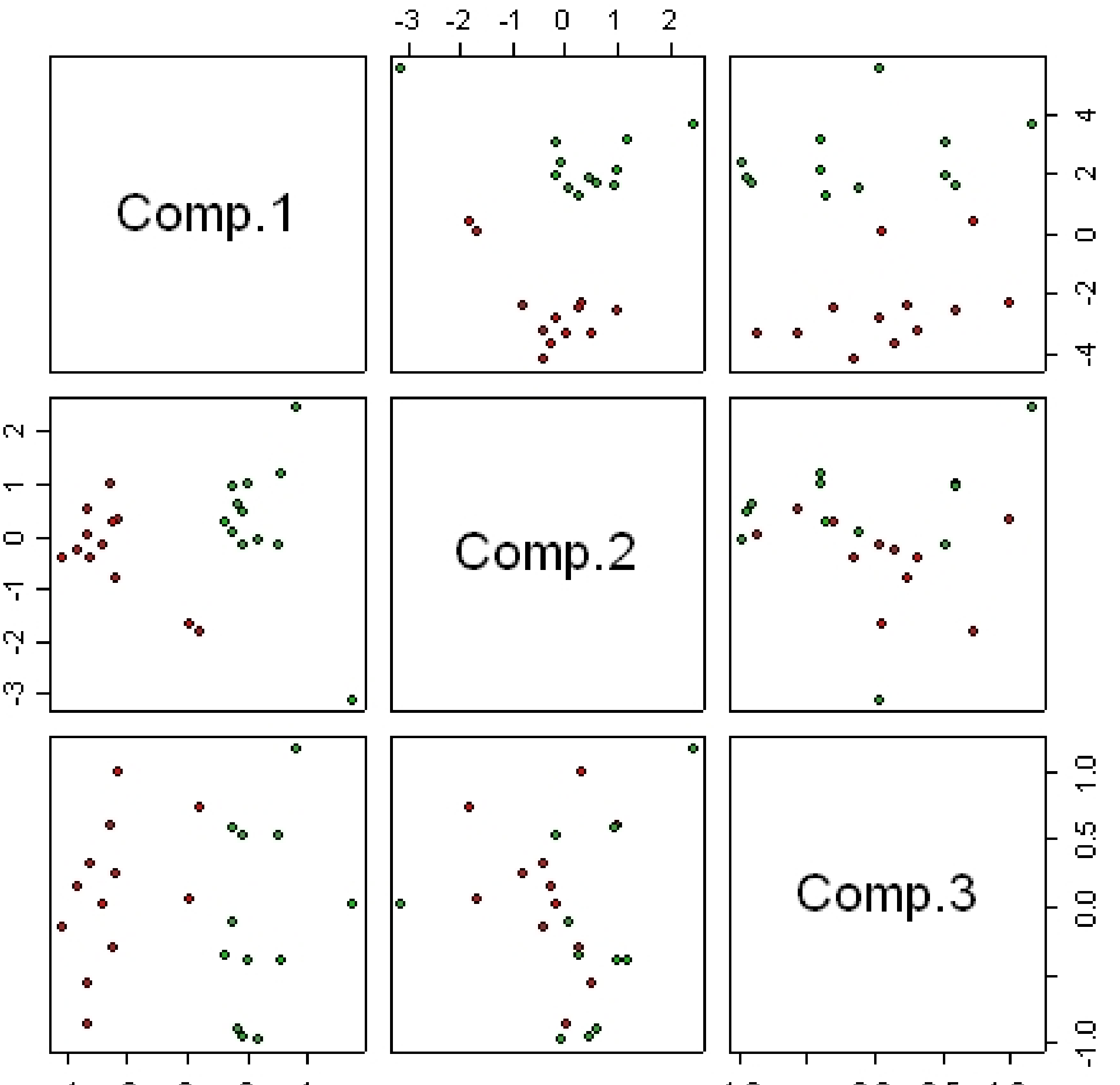

Comp.1
Comp.2
Comp.3
-3 -2 -1 0 1 2
4 2 0 -2 -4
2 1 0 -1 -2 -3
1.0 0.5 0.0 -1.0
-4 -2 0 2 4
-1.0 0.0 0.5 1.0
