# Supplementary material for: Differential protein profiling as a potential multi-marker approach for TSE diagnosis
Source: BMC Infect Dis. 2009 Nov 27;9:188. doi: 10.1186/1471-2334-9-188 (PMC2794872; doi:10.1186/1471-2334-9-188)

S1 CM10 30

Proteins showing total separation

No proteins showed complete separation

Significant data (p<=0.05)

No Significant Proteins

Data for Significant proteins

	C0GROUP	C0GRP_NA	C0Spectr
8	0	ME7 S1	B32943
9	0	ME7 S1	B32943
10	0	ME7 S1	B32944
1	1	Normal S1	B32937
2	1	Normal S1	B32937
3	1	Normal S1	B32938
4	1	Normal S1	B32939
5	1	Normal S1	B32940
6	1	Normal S1	B32941
7	1	Normal S1	B32942

All data

Boxplot of all proteins

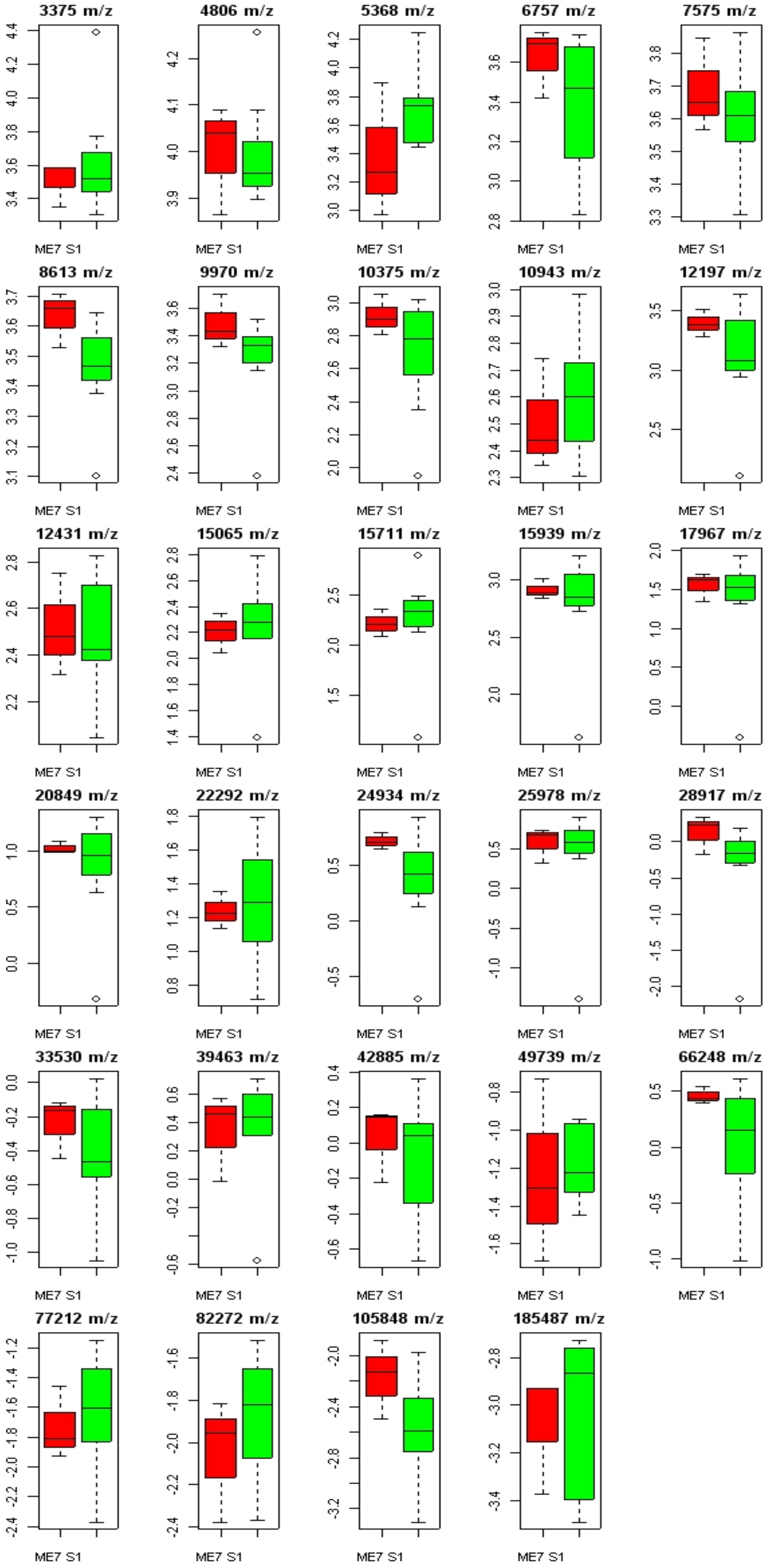

Boxplot of all proteins

Cluster Analysis of samples (Euclidean distance)

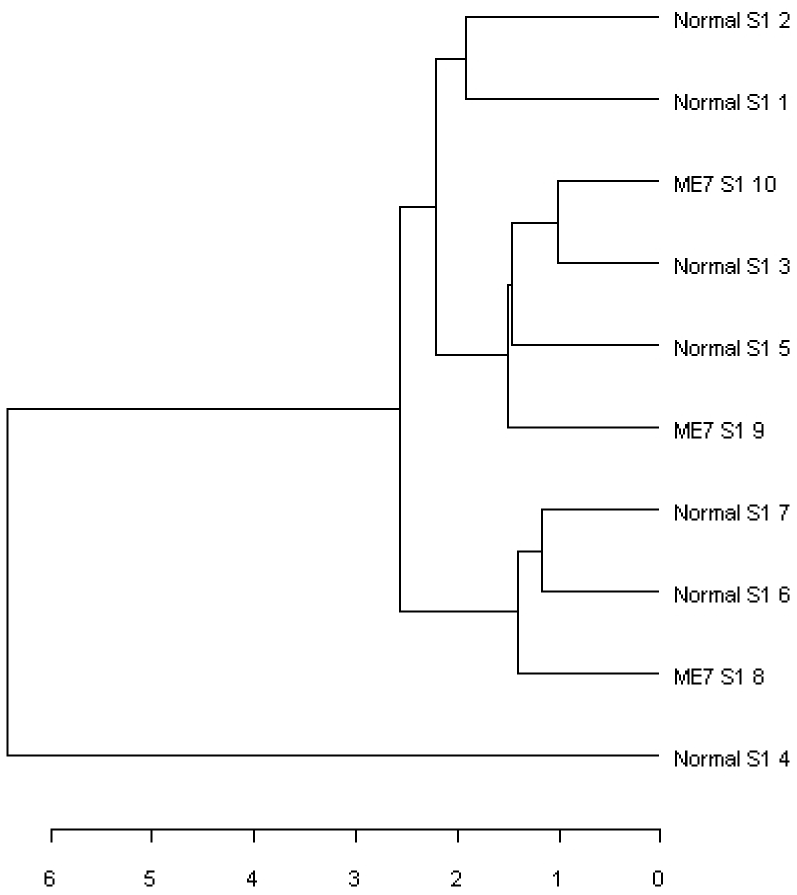

Cluster Analysis of samples (Euclidean distance)

S1 CM10 60

Proteins showing total separation

No proteins showed complete separation

Significant data (p<=0.05)

Significant Proteins (t-test; p<=0.05)

	name	mz	ME7.avg	NORM.avg	t	p
5	C06756_9	6756	3.467	3.74	-3.0	0.0094
21	C024920_	24920	0.545	0.38	2.1	0.0490
26	C042436_	42436	-0.014	-0.21	2.3	0.0340
32	C0187228	187228	-3.055	-3.43	3.5	0.0032

Data for Significant proteins

	C0GROUP	C0GRP_NA	C0Spectr	C06756_9	C024920_	C042436_	C0187228
1	0	ME7 S1	B32945	3.0	0.63	-0.056	-3.0
2	0	ME7 S1	B32945	3.5	0.78	0.141	-3.4
3	0	ME7 S1	B32946	3.2	0.50	0.054	-2.7
4	0	ME7 S1	B32947	3.3	0.63	0.060	-2.9
5	0	ME7 S1	B33445	3.5	0.32	-0.222	-3.6
13	0	ME7 S1	B33451	3.3	0.89	0.355	-2.9
14	0	ME7 S1	B33452	3.7	0.58	0.323	-2.8
15	0	ME7 S1	B33453	3.8	0.54	-0.258	-3.0
16	0	ME7 S1	B33454	3.6	0.31	-0.396	-3.5
17	0	ME7 S1	B33455	3.8	0.47	-0.131	-3.2
18	0	ME7 S1	B33456	3.3	0.34	-0.023	-2.7
6	1	Normal S1	B33446	3.7	0.32	-0.138	-3.5
7	1	Normal S1	B33447	3.9	0.16	-0.435	-3.3
8	1	Normal S1	B33447	3.6	0.52	-0.094	-3.5
9	1	Normal S1	B33448	3.9	0.30	-0.250	-3.3
10	1	Normal S1	B33449	3.6	0.39	-0.255	-3.4
11	1	Normal S1	B33449	3.7	0.57	-0.059	-3.3
12	1	Normal S1	B33450	3.7	0.41	-0.256	-3.7

Boxplot of significant proteins

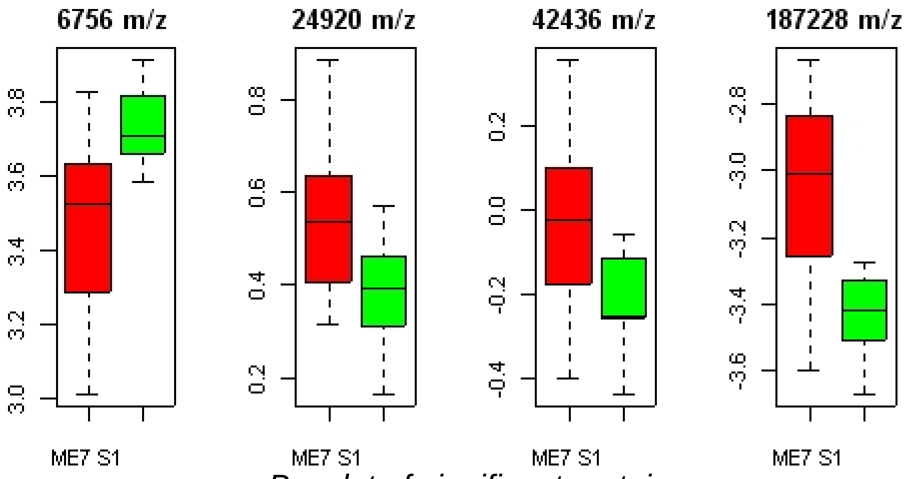

Boxplot of significant proteins

Pairwise Scatterplots of Significant Proteins

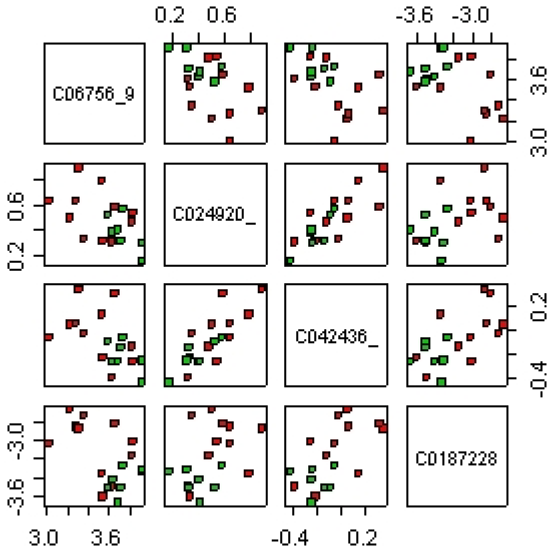

Pairwise Scatterplots of Significant Proteins

Cluster Analysis of samples (Euclidean distance)

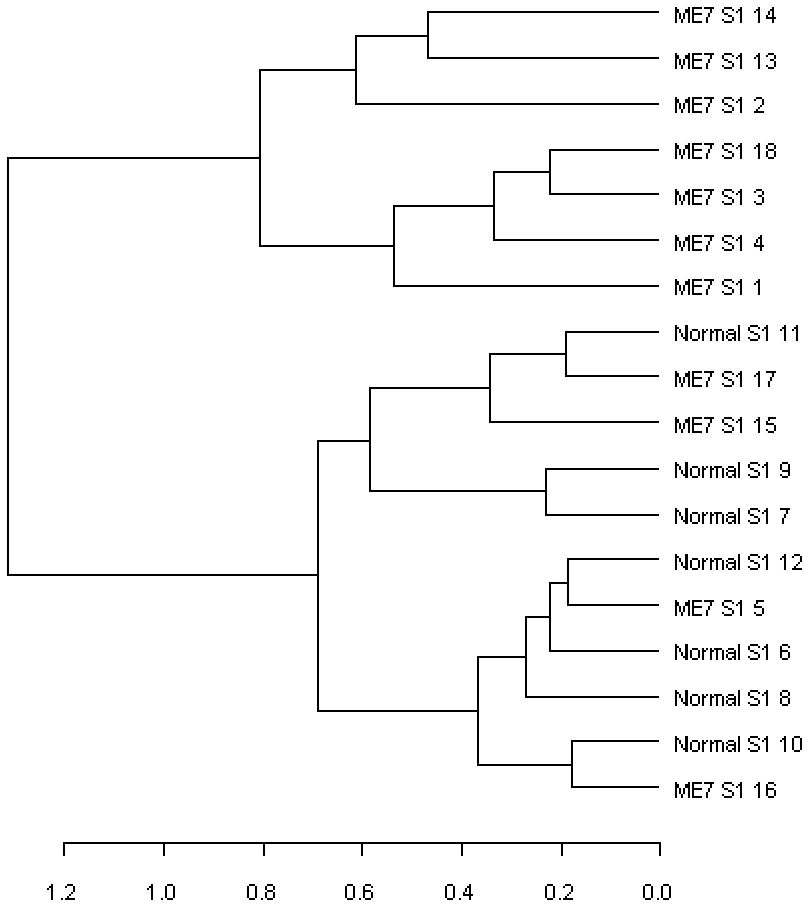

Cluster Analysis of samples (Euclidean distance)

Plot of first three principal components

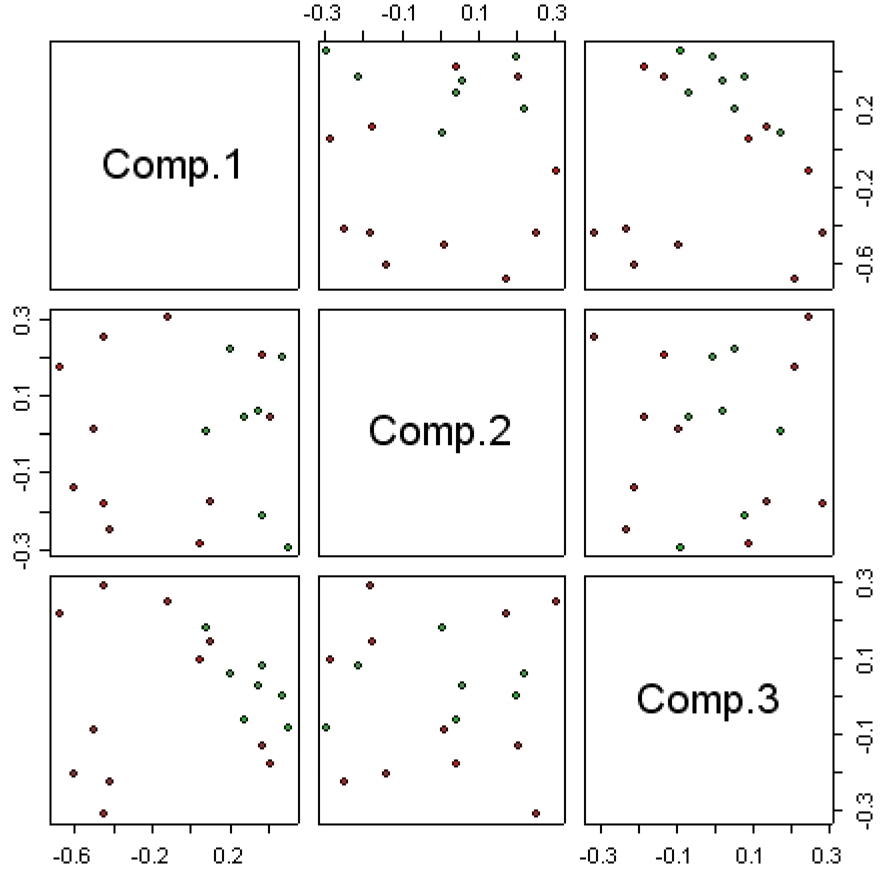

Plot of first three principal components

Scatterplot of linear discriminant function (x-axis)

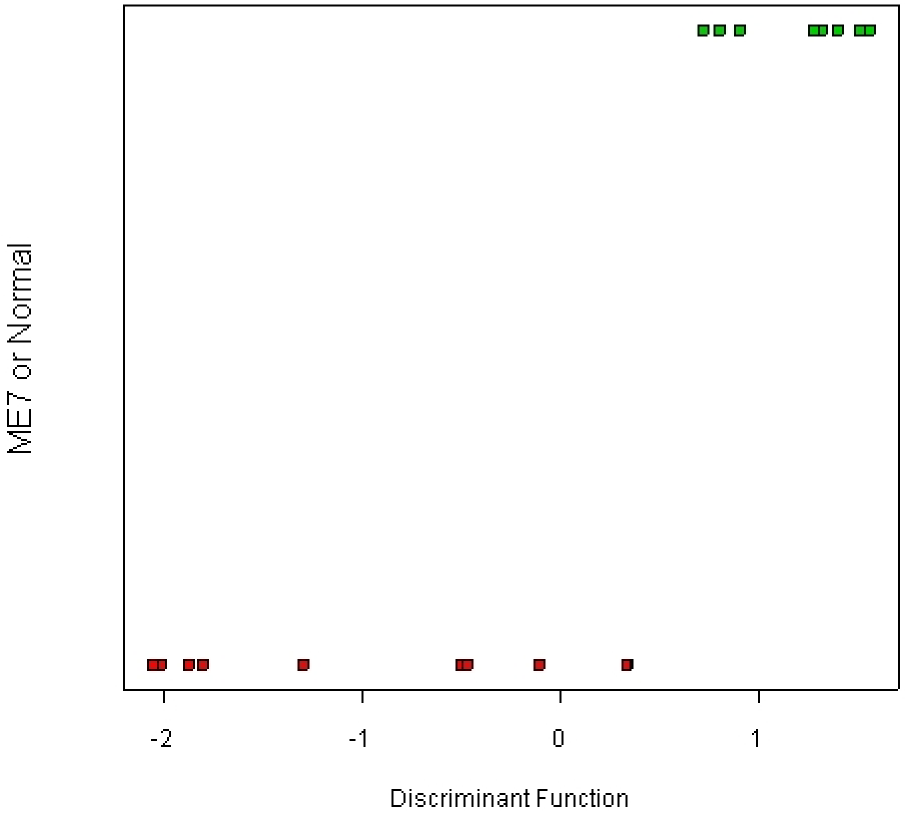

Scatterplot of linear discriminant function (x-axis)

All data

Boxplot of all proteins

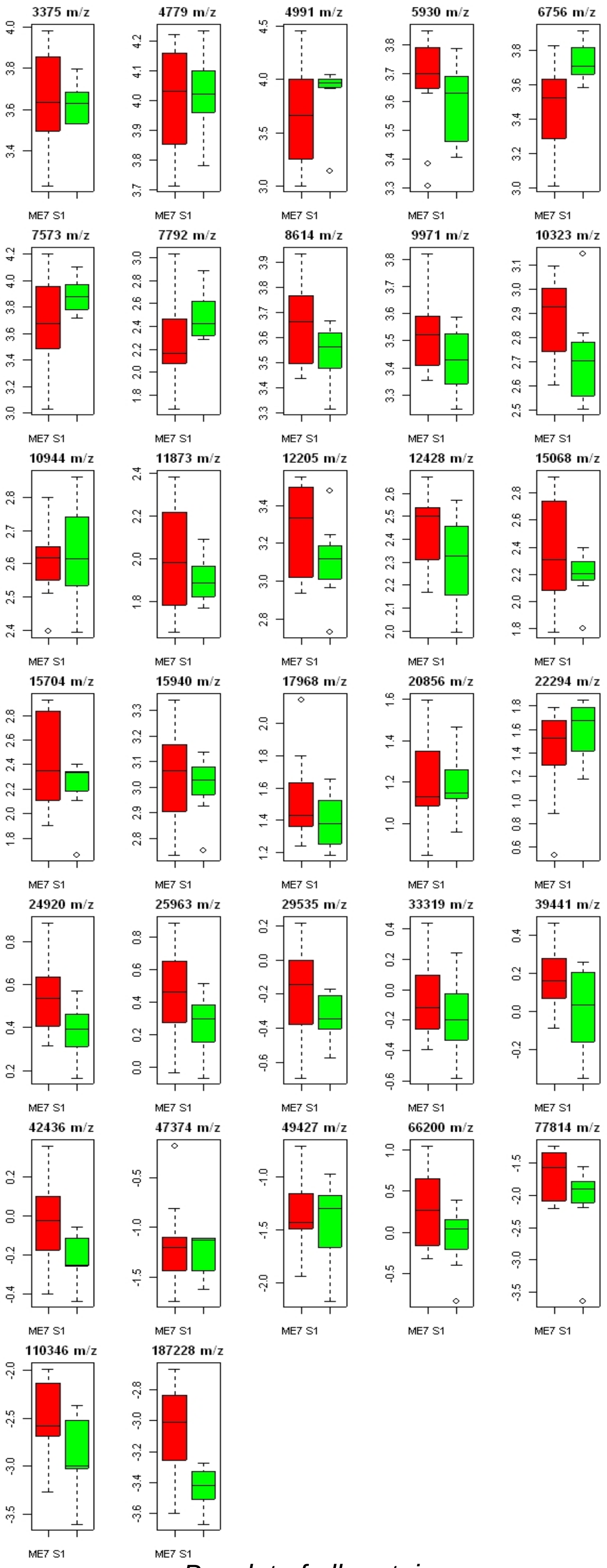

Boxplot of all proteins

Cluster Analysis of samples (Euclidean distance)

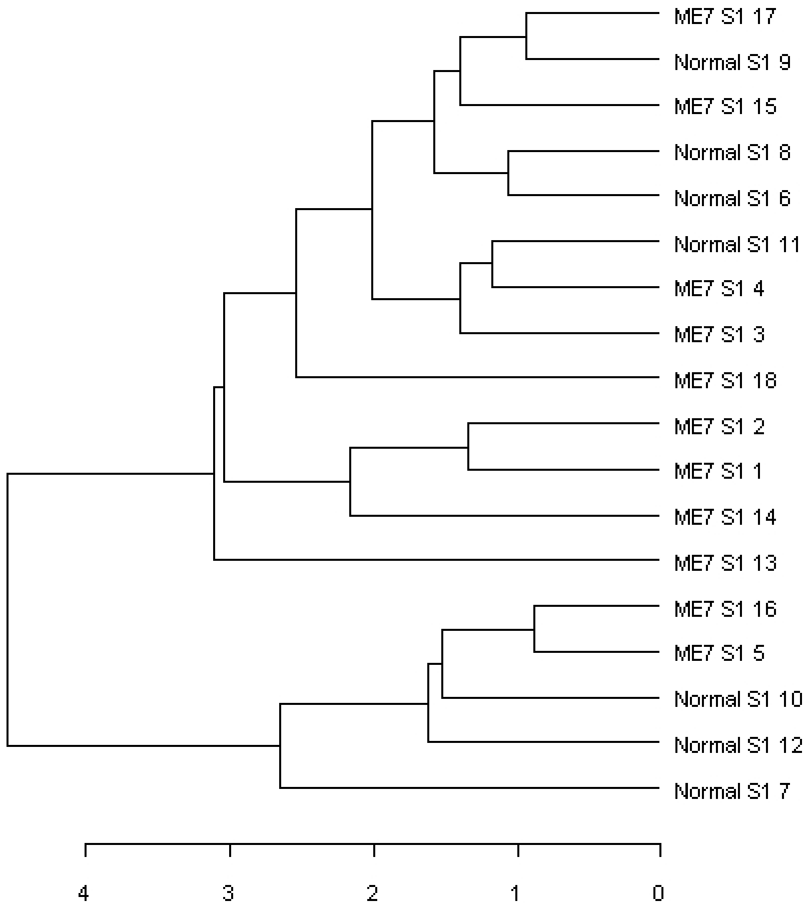

Cluster Analysis of samples (Euclidean distance)

S1 CM10 90

Proteins showing total separation

	C0GROUP	C0GRP_NA	C0Spectr	C06333_6	C0184754
7	0	ME7 S1	B34041	3.8	-3.9
8	0	ME7 S1	B34041	3.7	-3.7
9	0	ME7 S1	B34043	3.8	-3.8
10	0	ME7 S1	B34044	3.8	-3.6
11	0	ME7 S1	B34045	4.0	-3.3
12	0	ME7 S1	B34045	4.0	-3.2
13	0	ME7 S1	B34046	3.8	-4.0
14	0	ME7 S1	B34047	3.8	-3.4
1	1	Normal S1	B34033	3.6	-4.2
2	1	Normal S1	B34034	3.7	-4.3
3	1	Normal S1	B34035	3.6	-4.3
4	1	Normal S1	B34036	3.6	-4.2
5	1	Normal S1	B34039	3.6	-4.4
6	1	Normal S1	B34040	3.7	-4.1

Boxplot of proteins showing complete separation

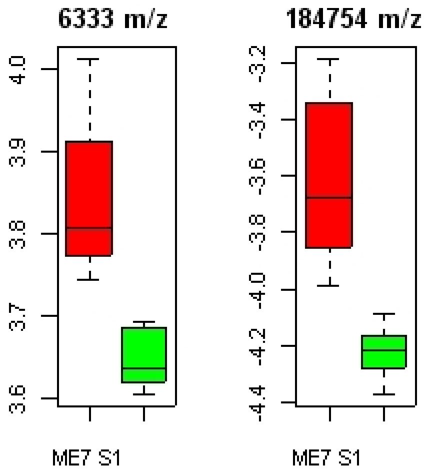

Pairwise Scatterplots of Proteins showing complete separation

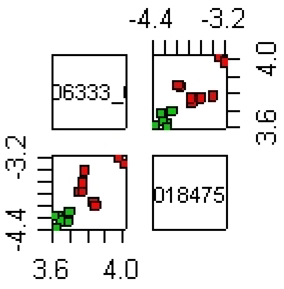

Significant data (p<=0.05)

Significant Proteins (t-test; p<=0.05)

	name	mz	ME7.avg	NORM.avg	t	p
1	C04817_9	4817	3.95	3.63	4.1	0.00143
2	C06333_6	6333	3.84	3.65	5.1	0.00057
3	C07599_7	7599	3.81	3.59	2.9	0.02214
4	C08595_6	8595	3.84	3.55	3.6	0.00523
11	C015094_	15094	1.50	2.09	-3.7	0.00534
12	C015730_	15730	1.51	2.08	-2.8	0.02596
18	C024918_	24918	0.59	0.27	2.6	0.03030
22	C042509_	42509	-0.34	-0.79	2.5	0.03278
28	C0152289	152289	-3.45	-3.87	3.3	0.01504
29	C0184754	184754	-3.62	-4.22	5.4	0.00045

Data for Significant proteins

	C0GROUP	C0GRP_NA	C0Spectr	C04817_9	C06333_6	C07599_7	C08595_6	C015094_	C015730_	C024918_	C042509_	C0152289	C0184754
7	0	ME7 S1	B34041	3.9	3.8	3.7	3.8	1.9	1.9	0.749	-0.1904	-3.5	-3.9
8	0	ME7 S1	B34041	3.8	3.7	3.7	3.7	1.6	1.7	0.466	-0.7201	-3.6	-3.7
9	0	ME7 S1	B34043	3.9	3.8	3.7	3.8	1.4	1.4	0.593	0.0170	-3.4	-3.8
10	0	ME7 S1	B34044	3.7	3.8	3.6	3.5	1.6	1.7	0.794	-0.4006	-3.6	-3.6
11	0	ME7 S1	B34045	4.0	4.0	3.9	4.0	1.7	1.6	0.879	0.0031	-3.3	-3.3
12	0	ME7 S1	B34045	4.3	4.0	4.3	4.3	1.2	1.1	0.287	-0.4199	-3.6	-3.2
13	0	ME7 S1	B34046	3.9	3.8	3.7	3.8	1.4	1.4	0.488	-0.5928	-3.3	-4.0
14	0	ME7 S1	B34047	4.0	3.8	3.8	3.9	1.2	1.3	0.481	-0.4459	-3.4	-3.4
1	1	Normal S1	B34033	3.7	3.6	3.6	3.5	2.1	2.2	0.071	-0.9361	-3.3	-4.2
2	1	Normal S1	B34034	3.8	3.7	3.6	3.6	2.3	2.4	0.095	-0.9715	-3.9	-4.3
3	1	Normal S1	B34035	3.5	3.6	3.6	3.5	2.1	2.2	0.756	-0.3750	-4.2	-4.3
4	1	Normal S1	B34036	3.6	3.6	3.6	3.5	2.4	2.5	0.343	-0.5581	-4.0	-4.2
5	1	Normal S1	B34039	3.6	3.6	3.6	3.5	2.2	2.1	0.193	-1.3412	-3.9	-4.4
6	1	Normal S1	B34040	3.6	3.7	3.6	3.7	1.5	1.2	0.145	-0.5311	-3.9	-4.1

Boxplot of significant proteins

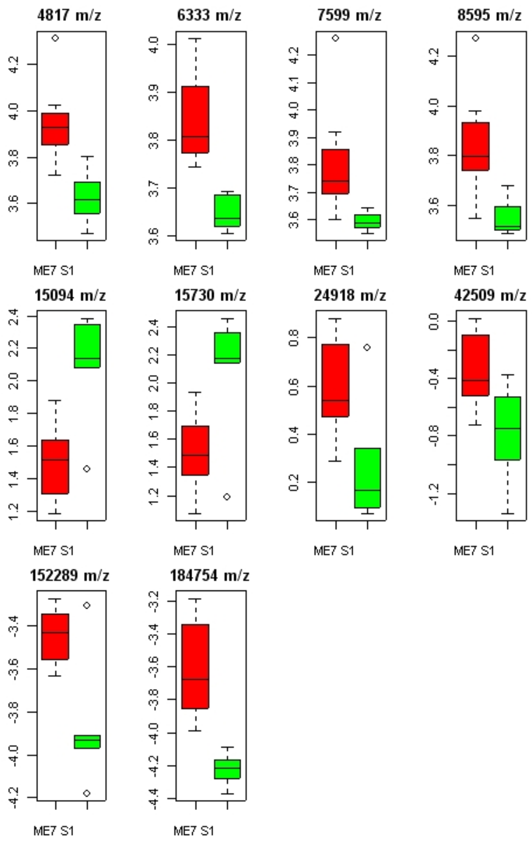

Boxplot of significant proteins

Pairwise Scatterplots of Significant Proteins

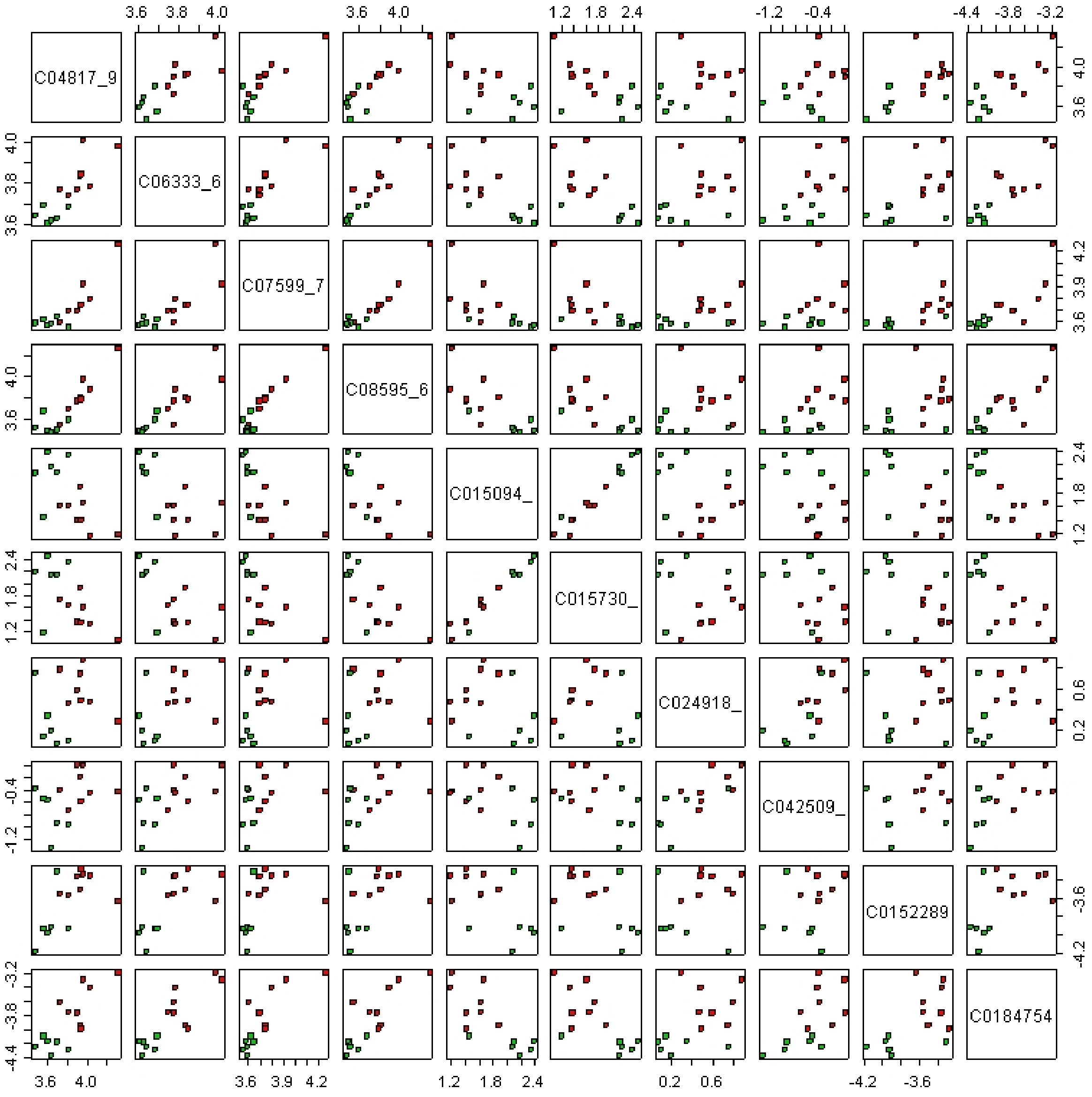

Pairwise Scatterplots of Significant Proteins

Cluster Analysis of samples (Euclidean distance)

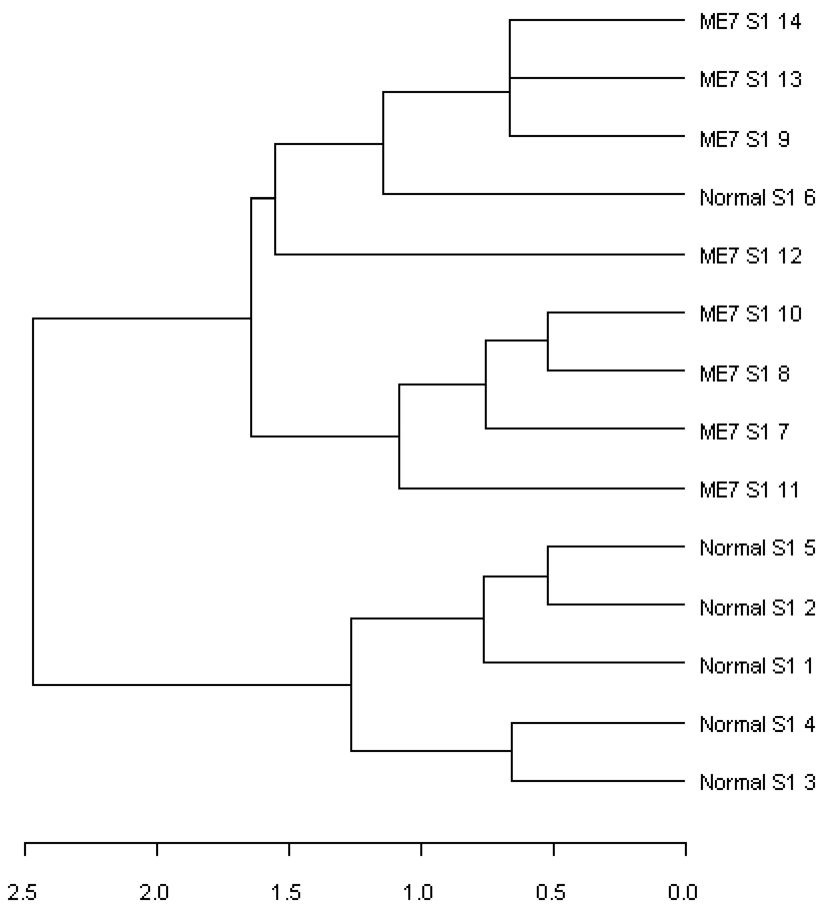

Cluster Analysis of samples (Euclidean distance)

Plot of first three principal components

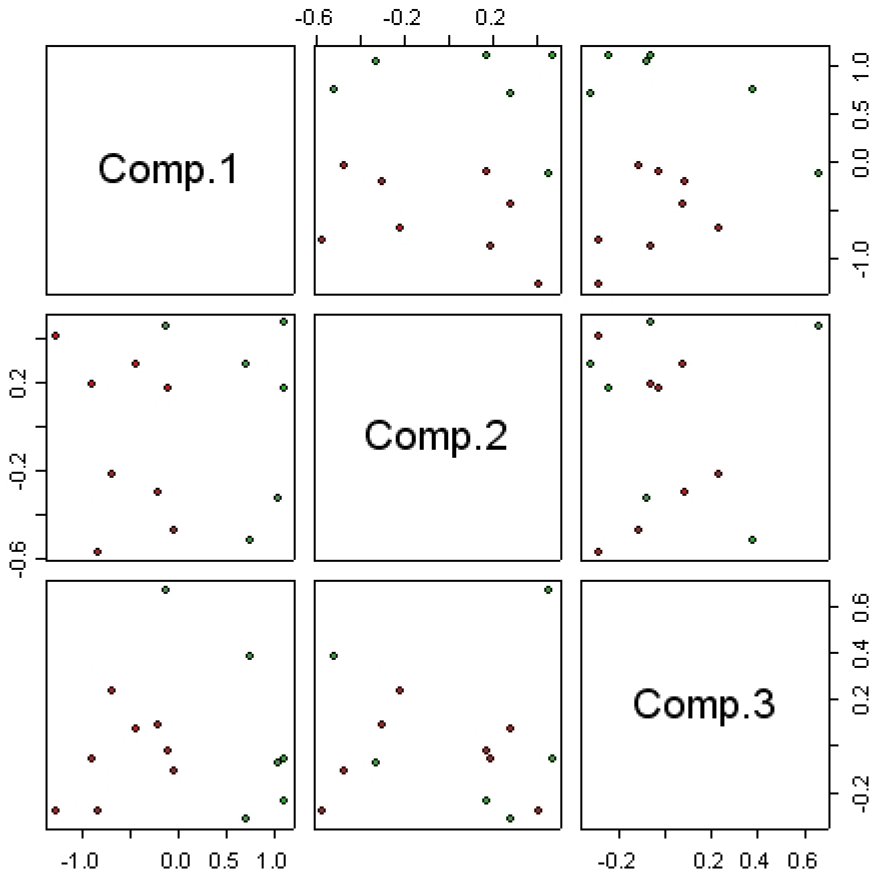

Plot of first three principal components

Scatterplot of linear discriminant function (x-axis)

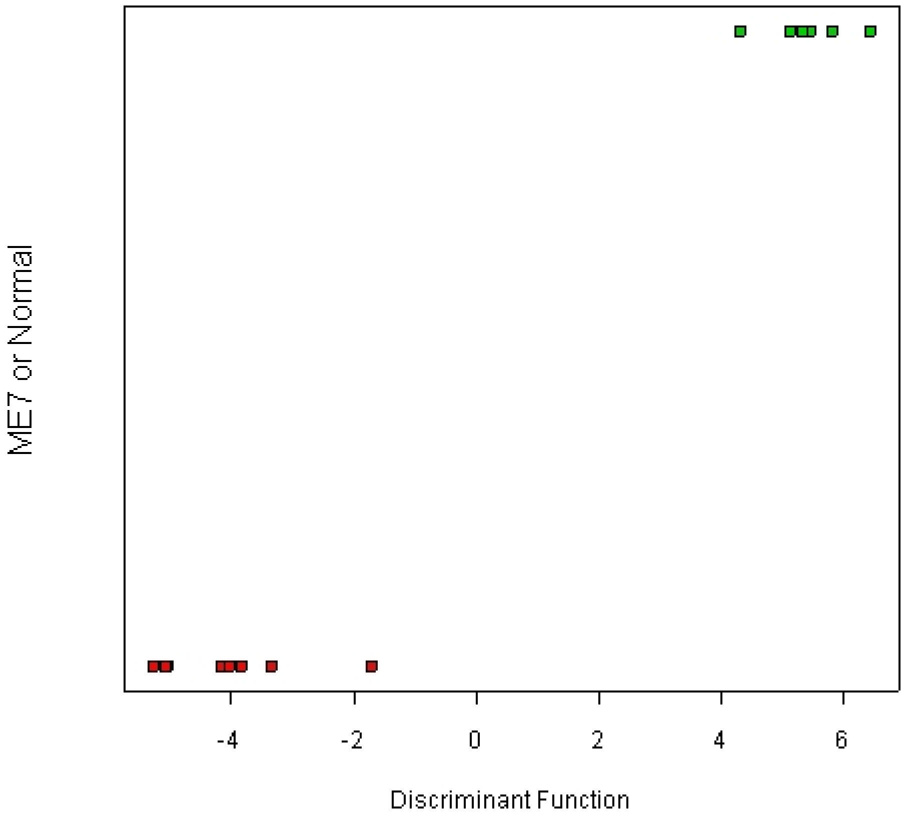

Scatterplot of linear discriminant function (x-axis)

All data

Boxplot of all proteins

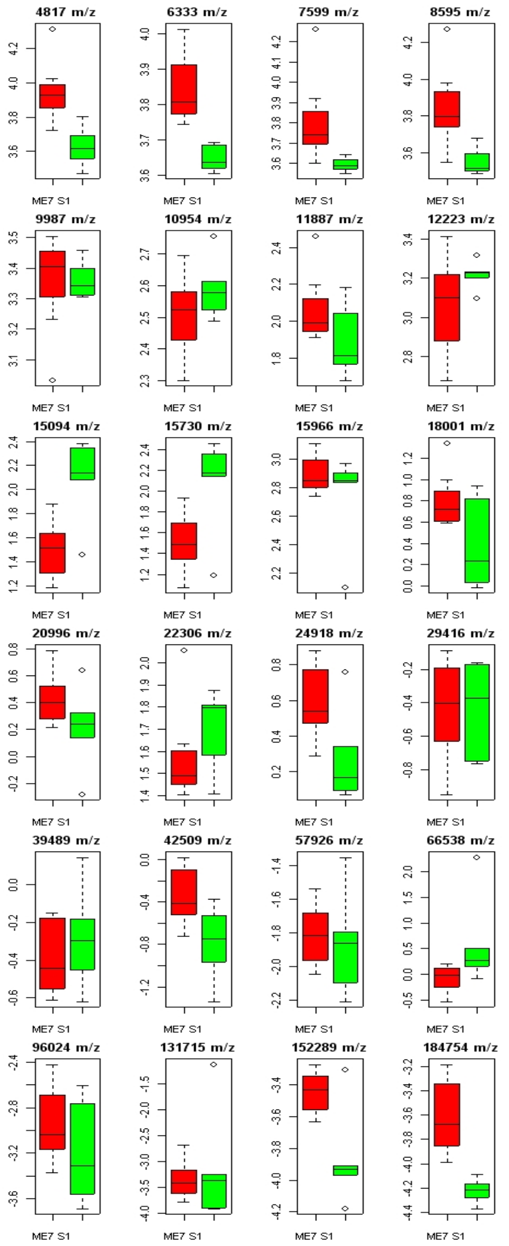

Boxplot of all proteins

Cluster Analysis of samples (Euclidean distance)

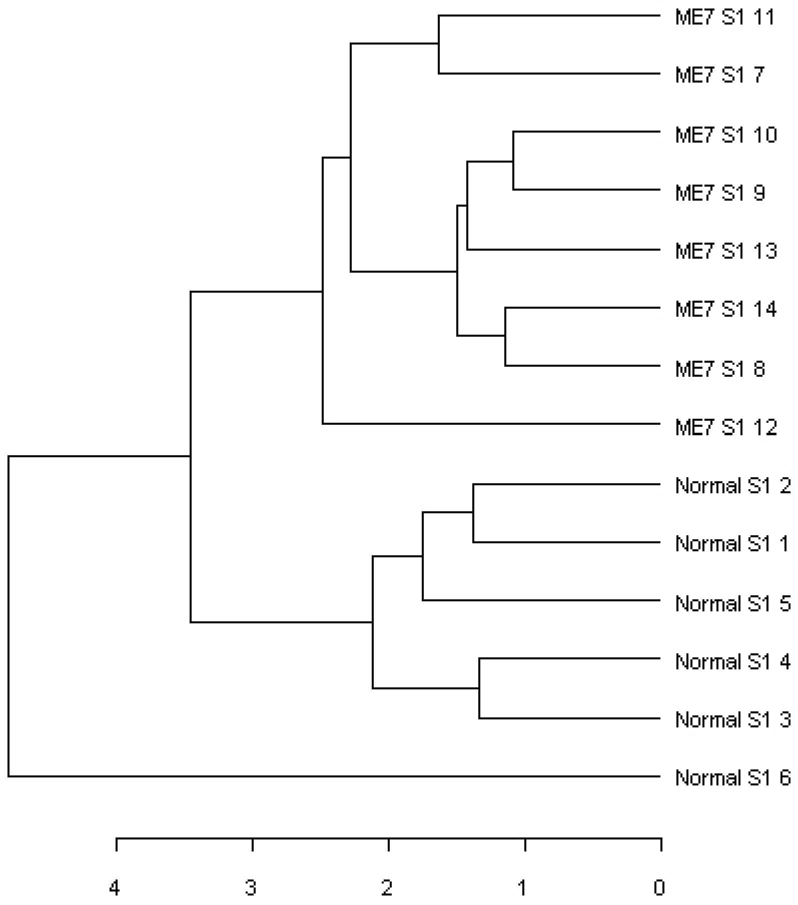

Cluster Analysis of samples (Euclidean distance)

S1 CM10 120

Proteins showing total separation

No proteins showed complete separation

Significant data (p<=0.05)

No Significant Proteins

Data for Significant proteins

	C0GROUP	C0GRP_NA	C0Spectr
13	0	ME7 S1	B34615
14	0	ME7 S1	B34615
15	0	ME7 S1	B34616
16	0	ME7 S1	B34616
17	0	ME7 S1	B34617
18	0	ME7 S1	B34618
19	0	ME7 S1	B34619
20	0	ME7 S1	B34620
21	0	ME7 S1	B34620
1	1	Normal S1	B34609
2	1	Normal S1	B34610
3	1	Normal S1	B34611
4	1	Normal S1	B34611
5	1	Normal S1	B34611
6	1	Normal S1	B34612
7	1	Normal S1	B34612
8	1	Normal S1	B34612
9	1	Normal S1	B34613
10	1	Normal S1	B34613
11	1	Normal S1	B34614
12	1	Normal S1	B34614

All data

Boxplot of all proteins

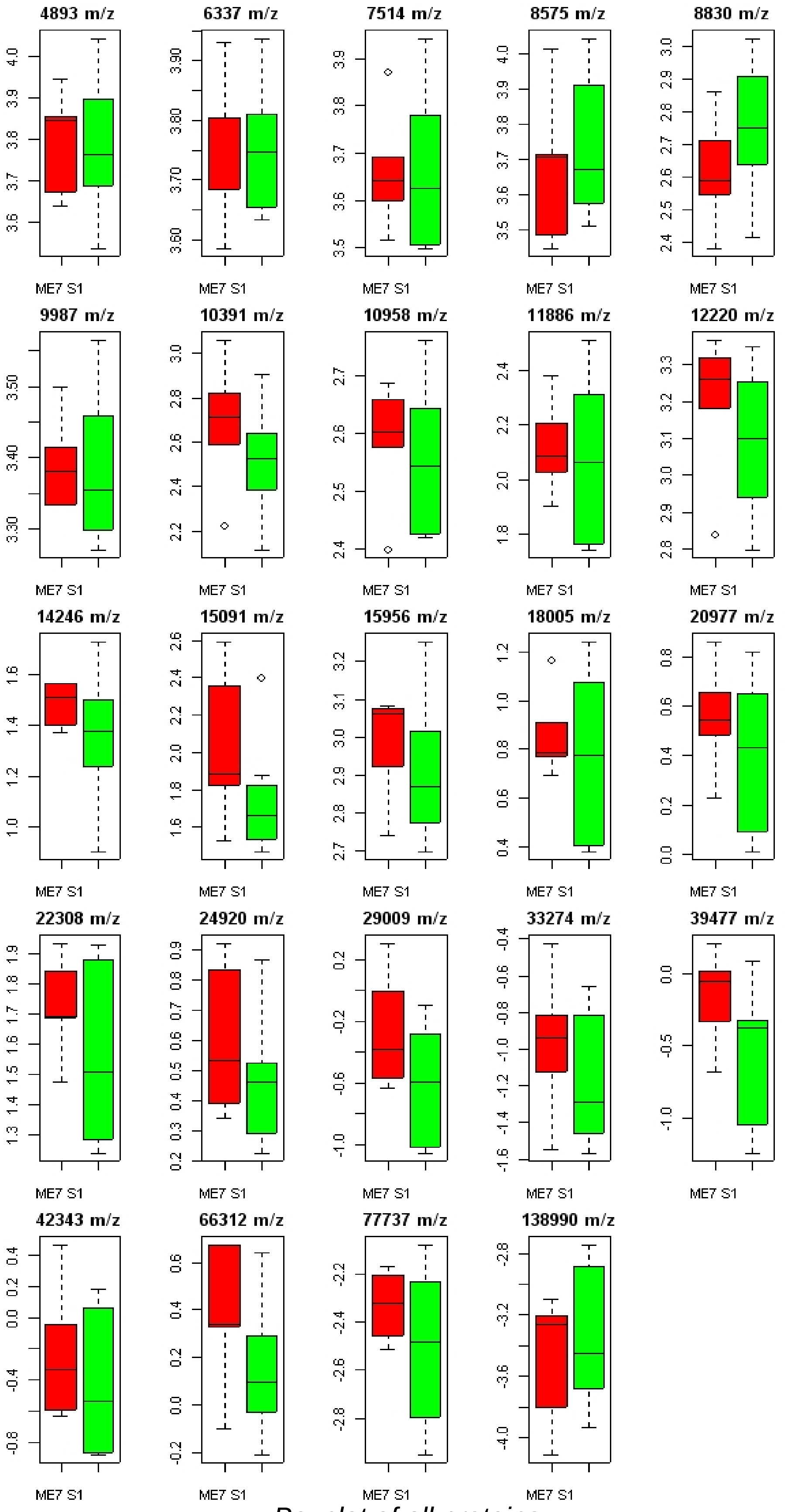

Boxplot of all proteins

Cluster Analysis of samples (Euclidean distance)

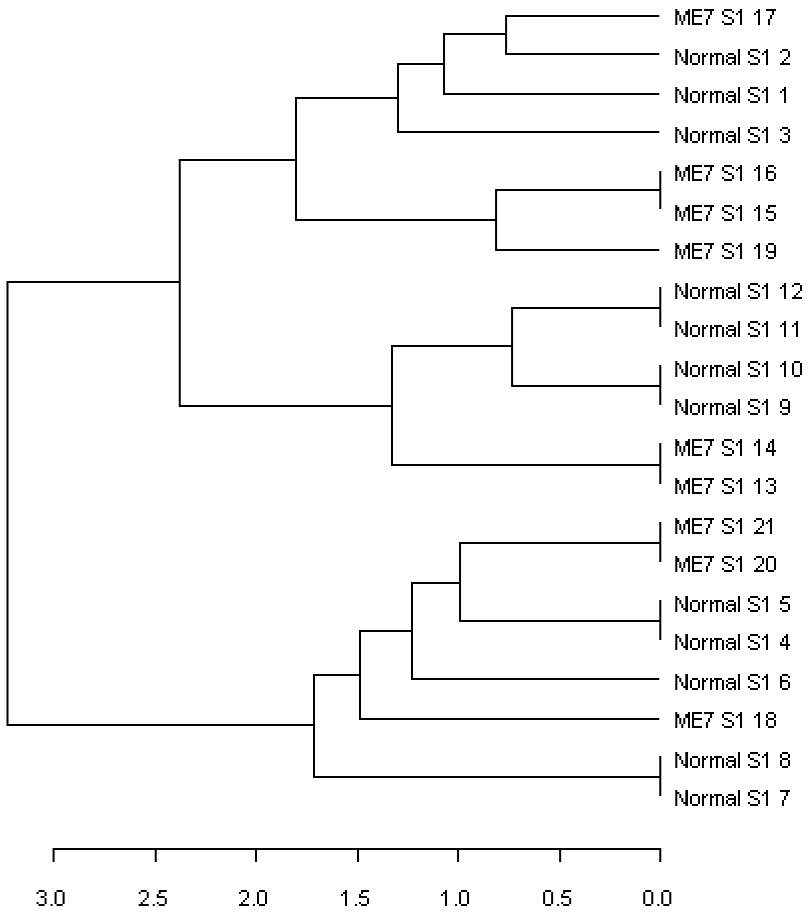

Cluster Analysis of samples (Euclidean distance)

S1 CM10 150

Proteins showing total separation

No proteins showed complete separation

Significant data (p<=0.05)

No Significant Proteins

Data for Significant proteins

	C0GROUP	C0GRP_NA	C0Spectr
8	0	ME7 S1	B35179
9	0	ME7 S1	B35180
10	0	ME7 S1	B35181
11	0	ME7 S1	B35182
12	0	ME7 S1	B35183
13	0	ME7 S1	B35184
14	0	ME7 S1	B35185
1	1	Normal S1	B35173
2	1	Normal S1	B35174
3	1	Normal S1	B35175
4	1	Normal S1	B35176
5	1	Normal S1	B35177
6	1	Normal S1	B35177
7	1	Normal S1	B35178

All data

Boxplot of all proteins

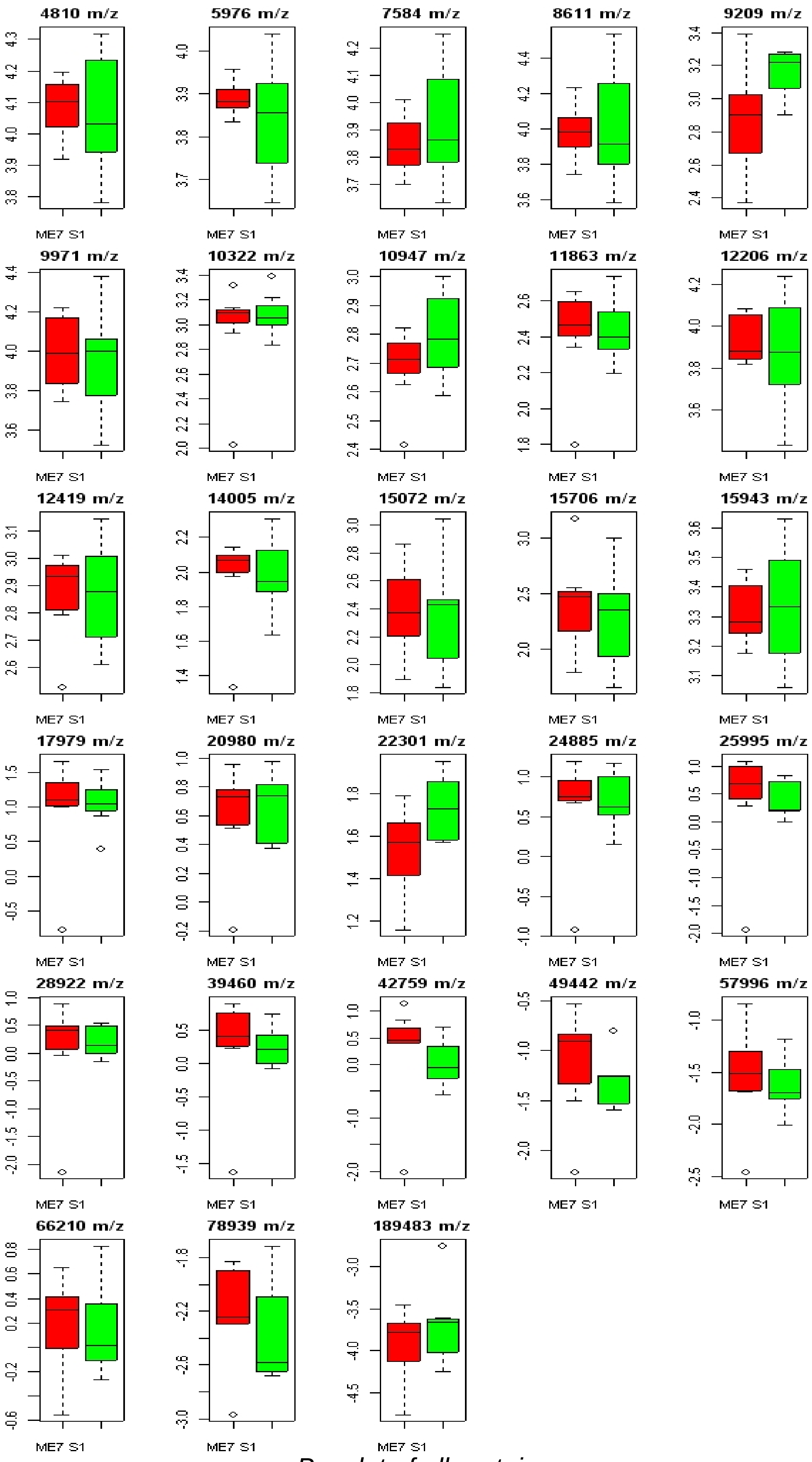

Boxplot of all proteins

Cluster Analysis of samples (Euclidean distance)

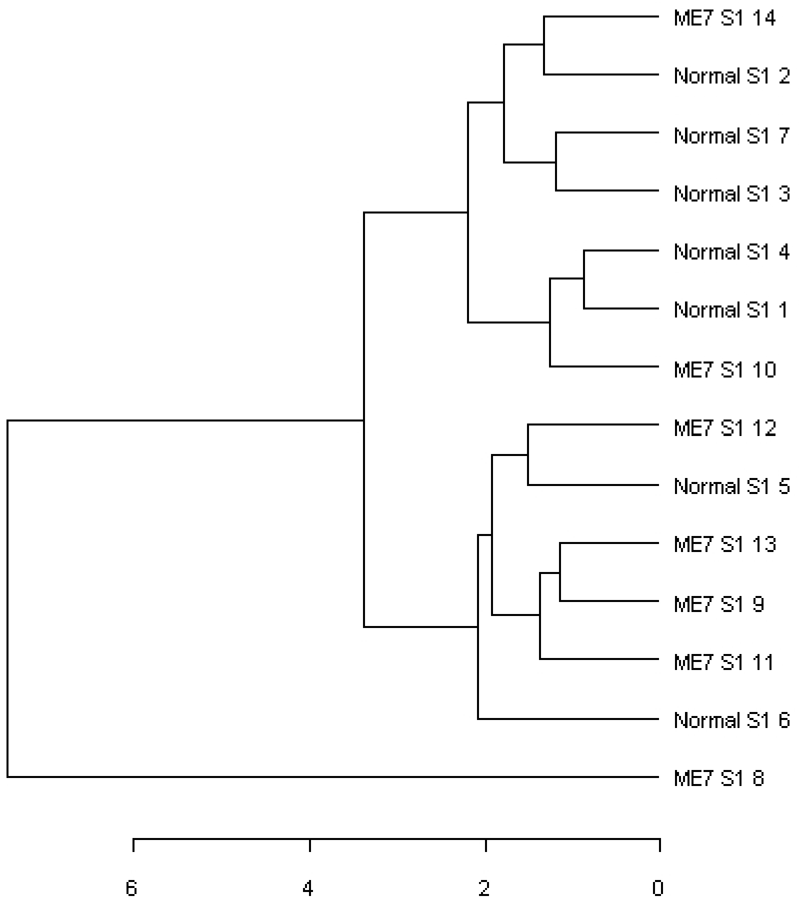

Cluster Analysis of samples (Euclidean distance)

S1 CM10 180

Proteins showing total separation

No proteins showed complete separation

Significant data (p<=0.05)

Significant Proteins (t-test; p<=0.05)

	name	mz	ME7. avg	NORM. avg	t	p
2	C06435_0	6435	3.69	3.86	-2.3	0.0407
8	C012426_	12426	2.74	2.83	-2.5	0.0291
13	C017991_	17991	0.50	0.91	-3.7	0.0043
14	C021102_	21102	0.28	0.49	-3.3	0.0084
15	C022322_	22322	1.49	1.73	-2.3	0.0419
18	C028960_	28960	-0.52	-0.22	-2.4	0.0377
19	C039492_	39492	-0.59	-0.30	-2.7	0.0228

Data for Significant proteins

	C0GROUP	C0GRP_NA	C0Spectr	C06435_0	C012426_	C017991_	C021102_	C022322_	C028960_	C039492_
8	0	ME7 S1	B35770	3.5	2.8	0.30	0.43	1.5	-0.637	-0.7207
9	0	ME7 S1	B35771	3.7	2.7	0.66	0.41	1.4	-0.279	-0.4954
10	0	ME7 S1	B35772	3.8	2.7	0.47	0.23	1.4	-0.288	-0.3421
11	0	ME7 S1	B35773	3.8	2.8	0.49	0.16	1.4	-0.576	-0.4286
12	0	ME7 S1	B35774	3.7	2.8	0.44	0.14	1.5	-0.556	-0.6675
13	0	ME7 S1	B35775	3.7	2.6	0.65	0.30	1.7	-0.785	-0.8809
1	1	Normal S1	B35764	3.9	2.8	0.99	0.52	2.1	-0.018	0.0029
2	1	Normal S1	B35765	4.0	2.8	0.88	0.50	1.6	-0.432	-0.3919
3	1	Normal S1	B35766	3.9	2.8	1.23	0.50	1.9	-0.139	-0.2793
4	1	Normal S1	B35767	3.8	2.9	0.56	0.29	1.5	-0.451	-0.4201
5	1	Normal S1	B35767	3.8	2.8	0.80	0.45	1.6	-0.062	-0.4962
6	1	Normal S1	B35768	4.1	3.0	1.19	0.66	1.8	0.088	-0.1282
7	1	Normal S1	B35769	3.6	2.8	0.70	0.49	1.6	-0.557	-0.4068

Boxplot of significant proteins

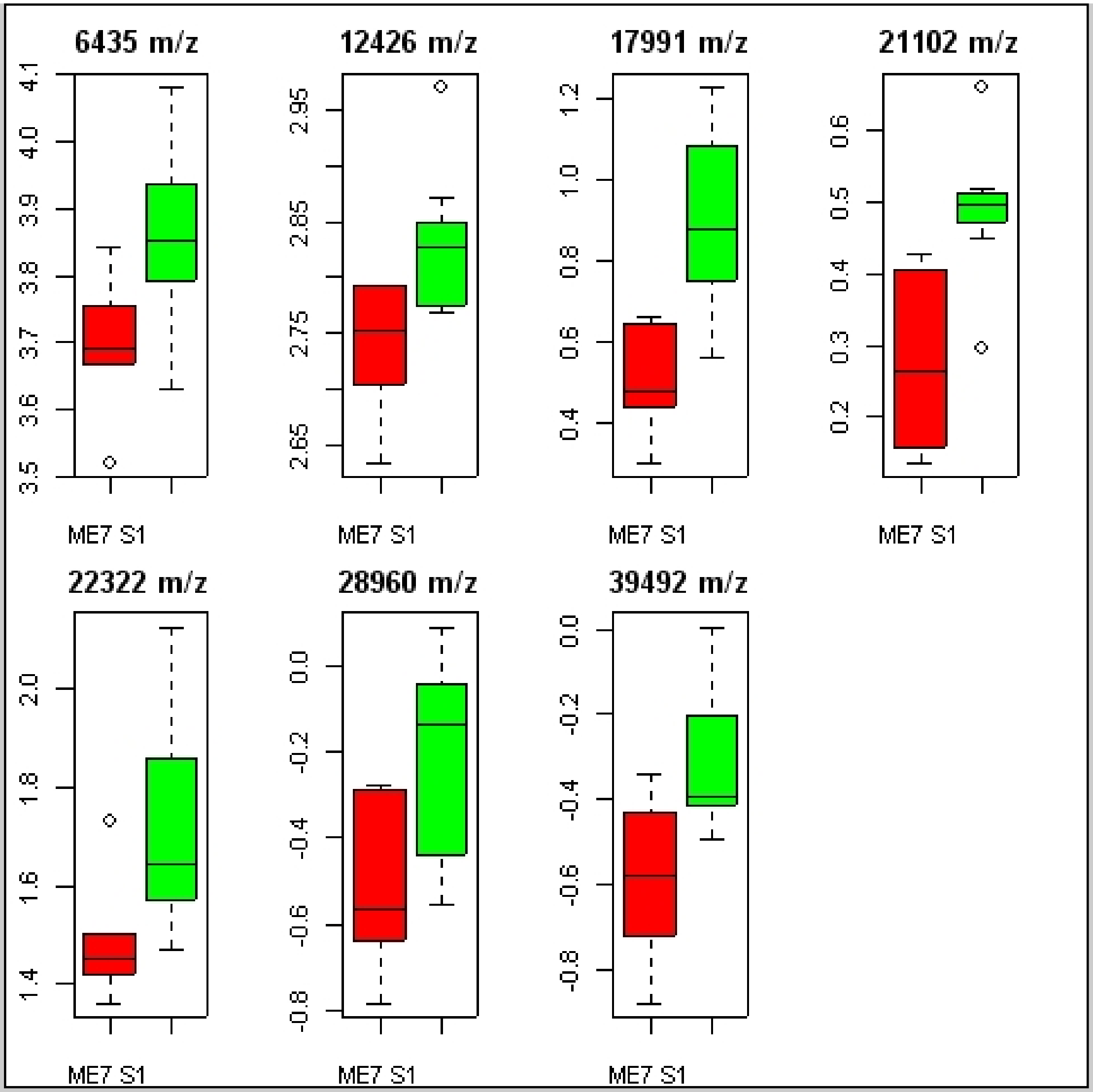

Boxplot of significant proteins

Pairwise Scatterplots of Significant Proteins

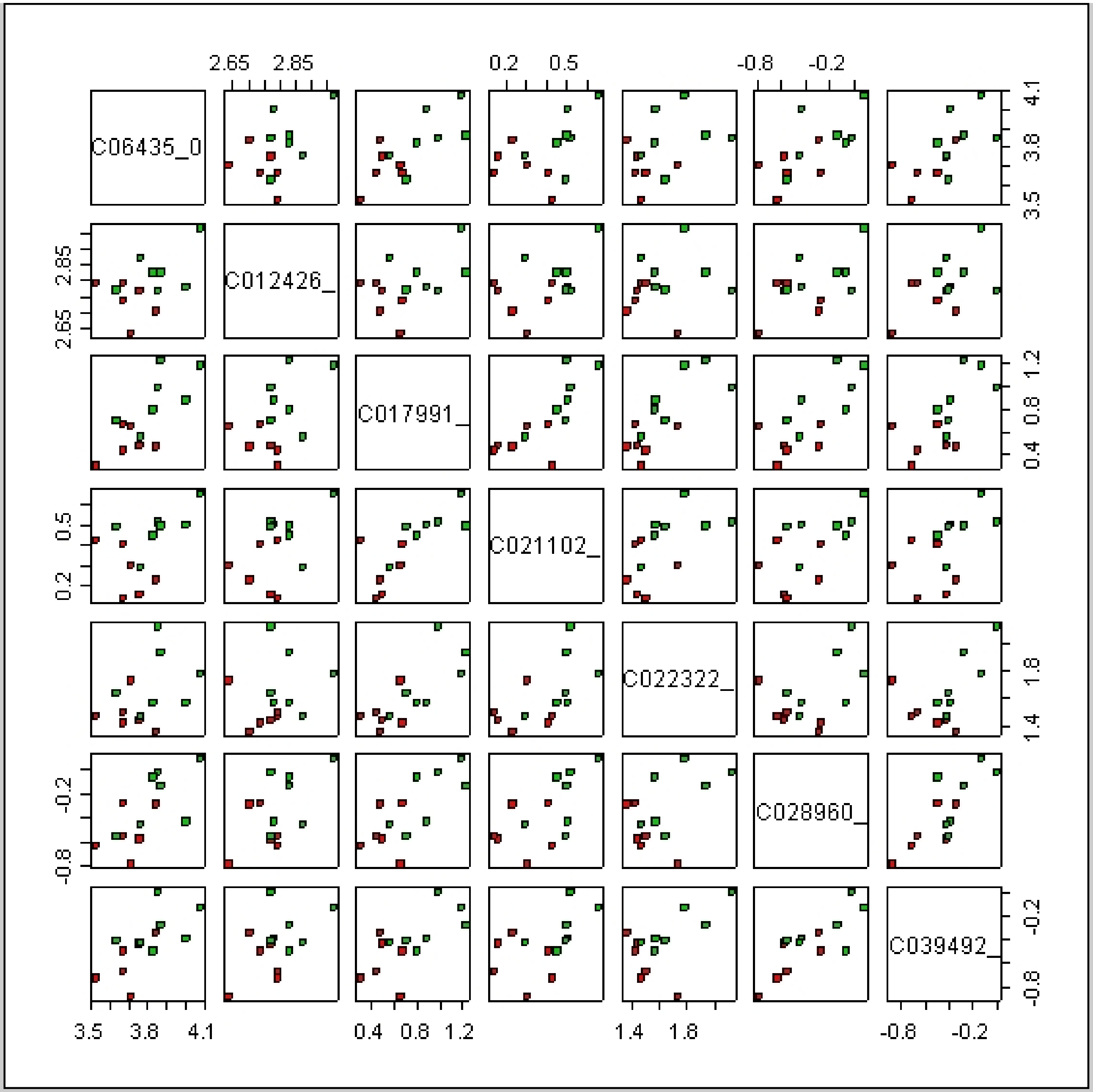

Pairwise Scatterplots of Significant Proteins

Cluster Analysis of samples (Euclidean distance)

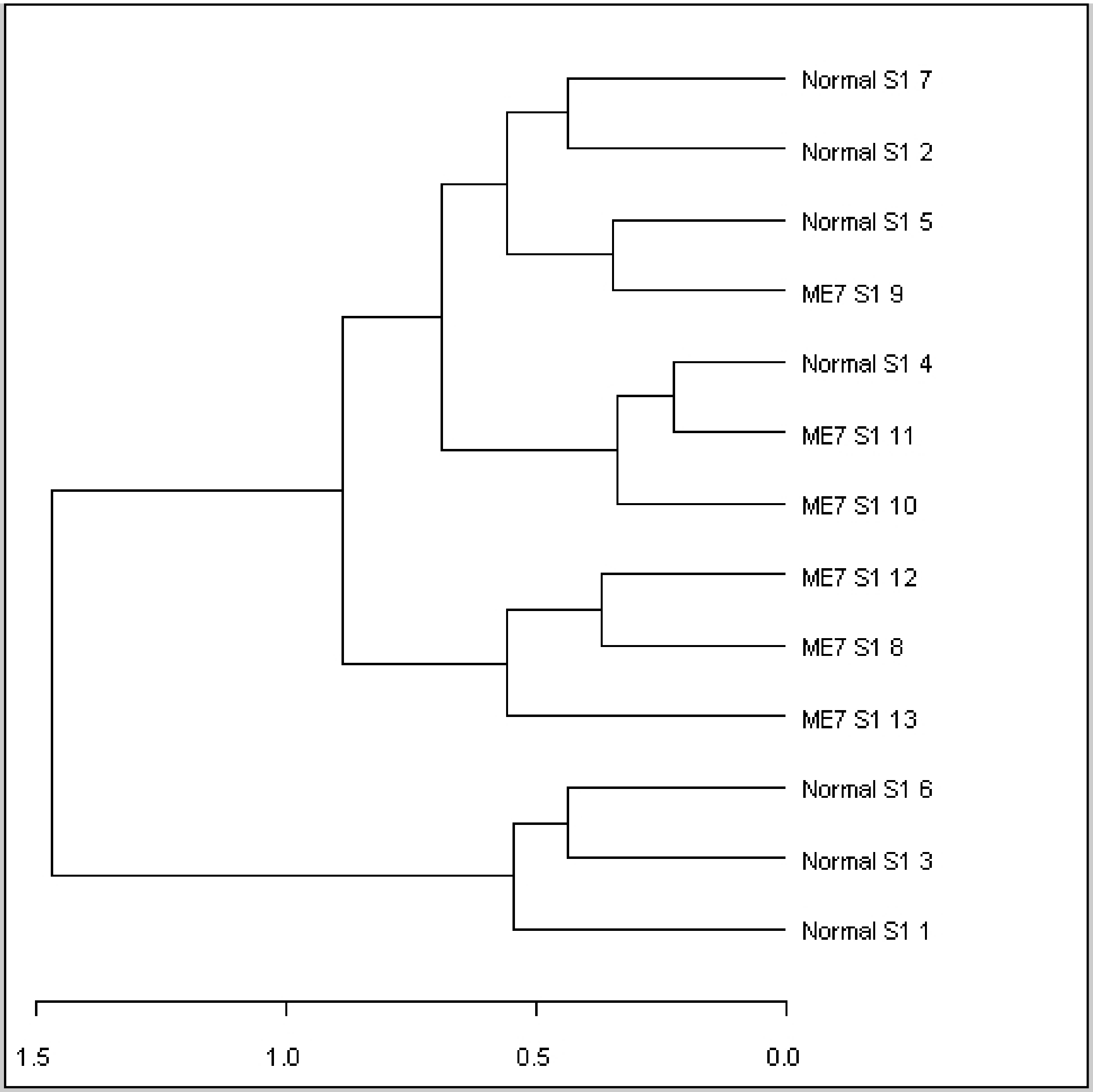

Cluster Analysis of samples (Euclidean distance)

Plot of first three principal components

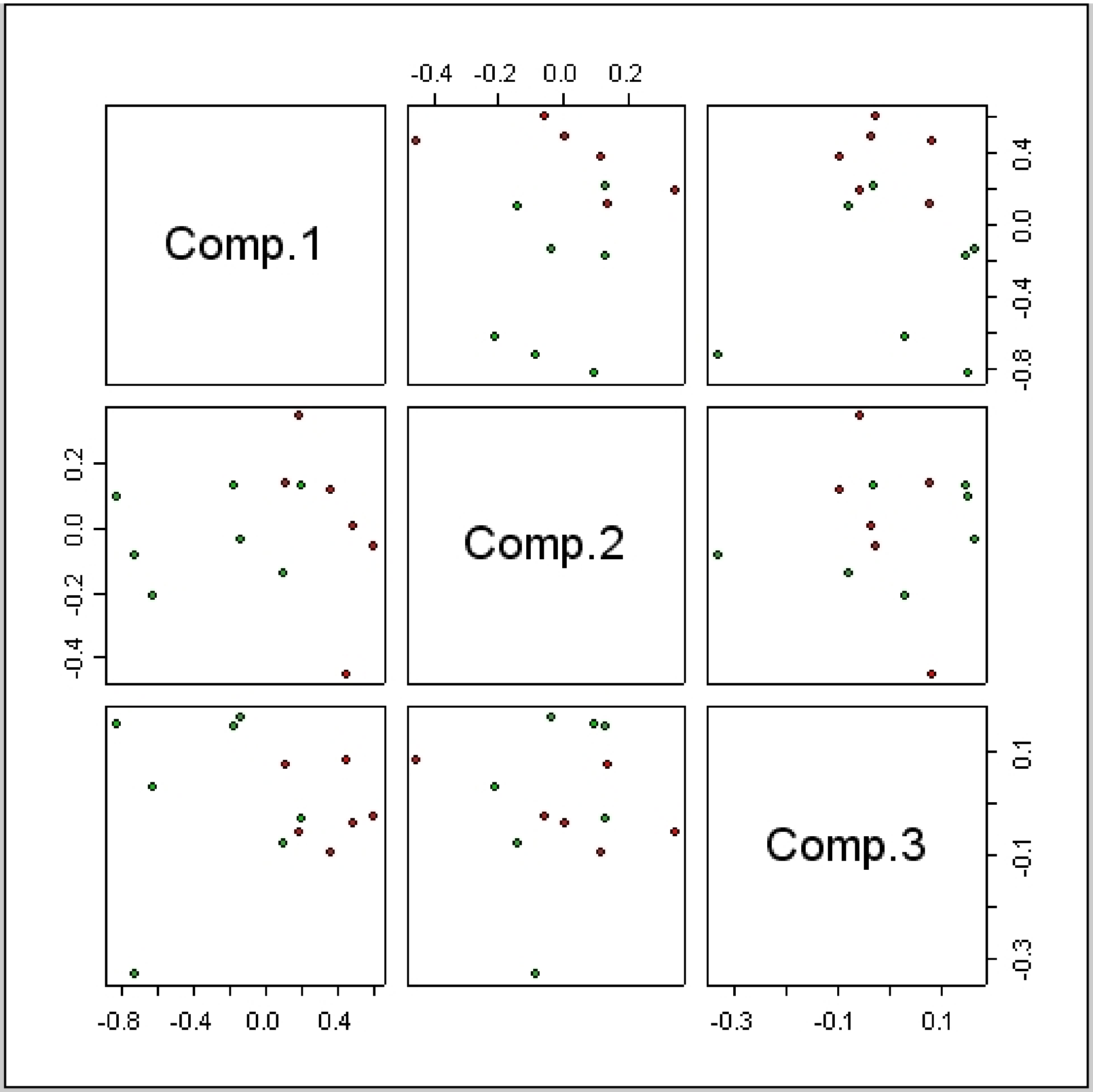

Plot of first three principal components

Scatterplot of linear discriminant function (x-axis)

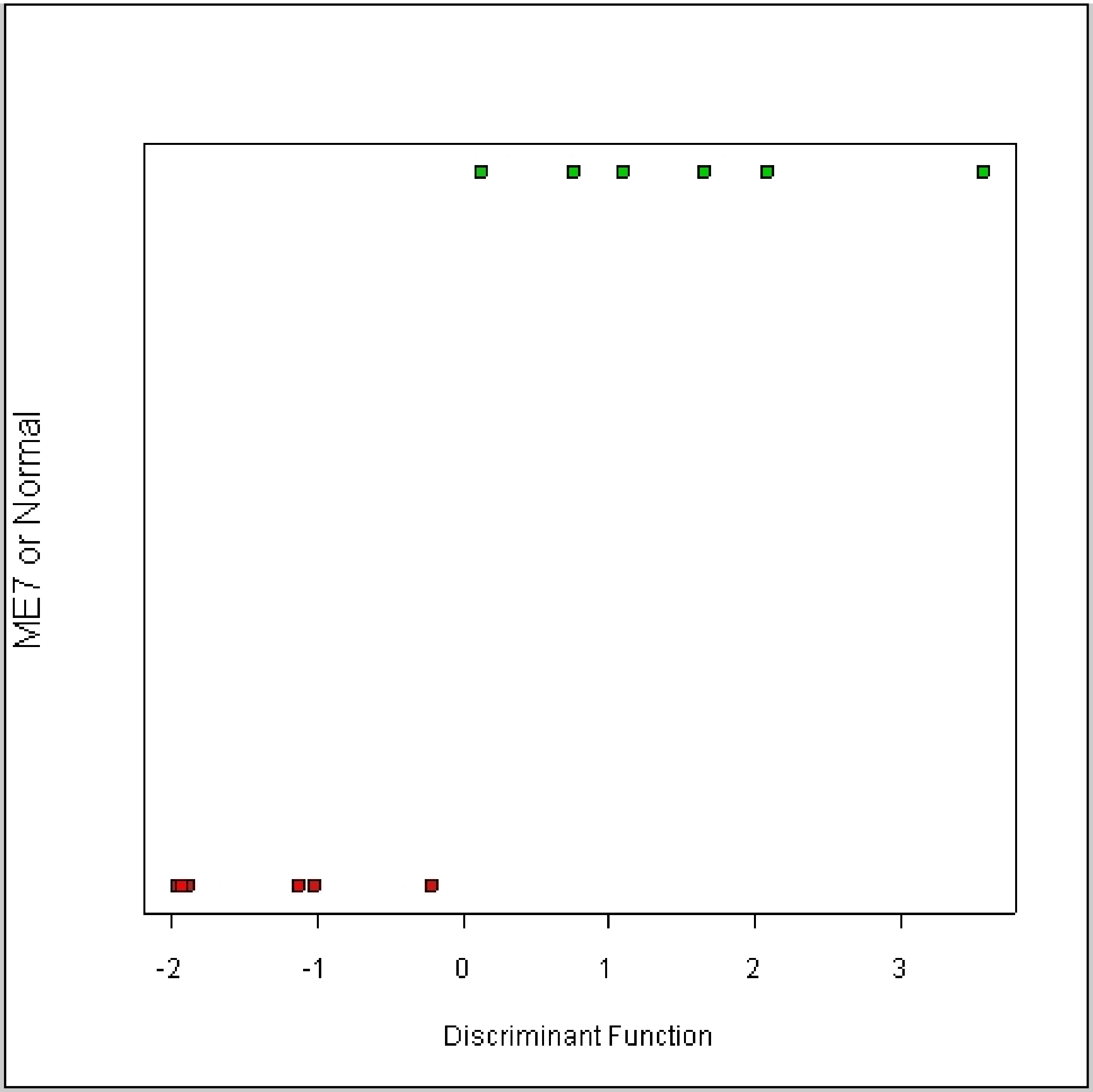

Scatterplot of linear discriminant function (x-axis)

All data

Boxplot of all proteins

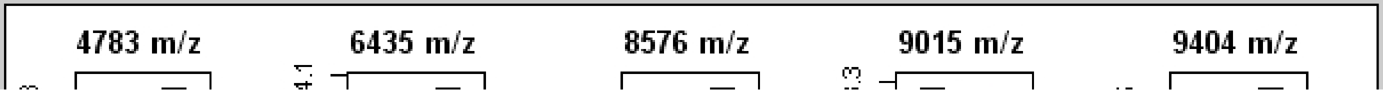

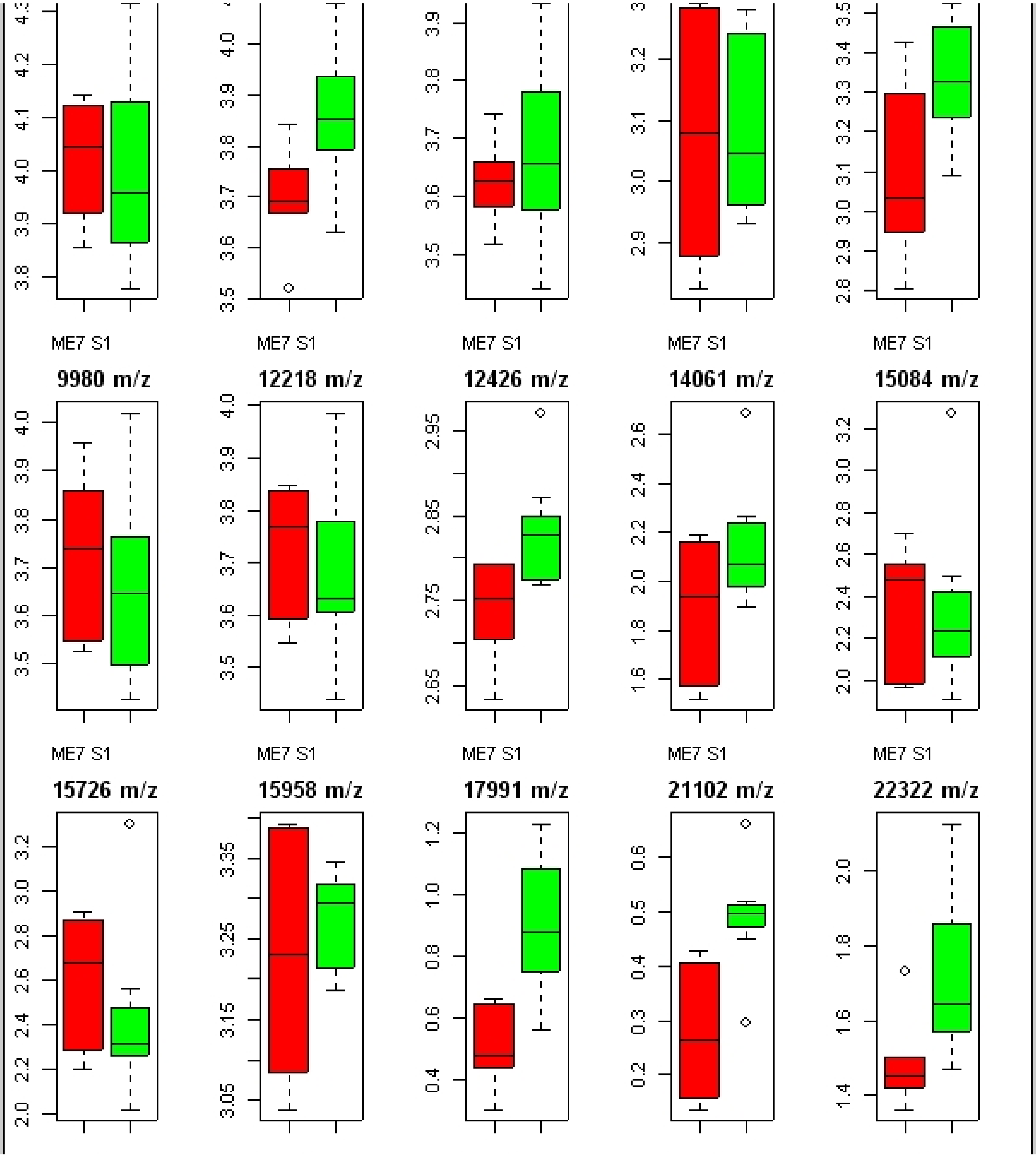
ME7 S1
ME7 S1
ME7 S1
ME7 S1
ME7 S1
9980 m/z
12218 m/z
12426 m/z
14061 m/z
15084 m/z
ME7 S1
ME7 S1
ME7 S1
ME7 S1
ME7 S1
15726 m/z
15958 m/z
17991 m/z
21102 m/z
22322 m/z

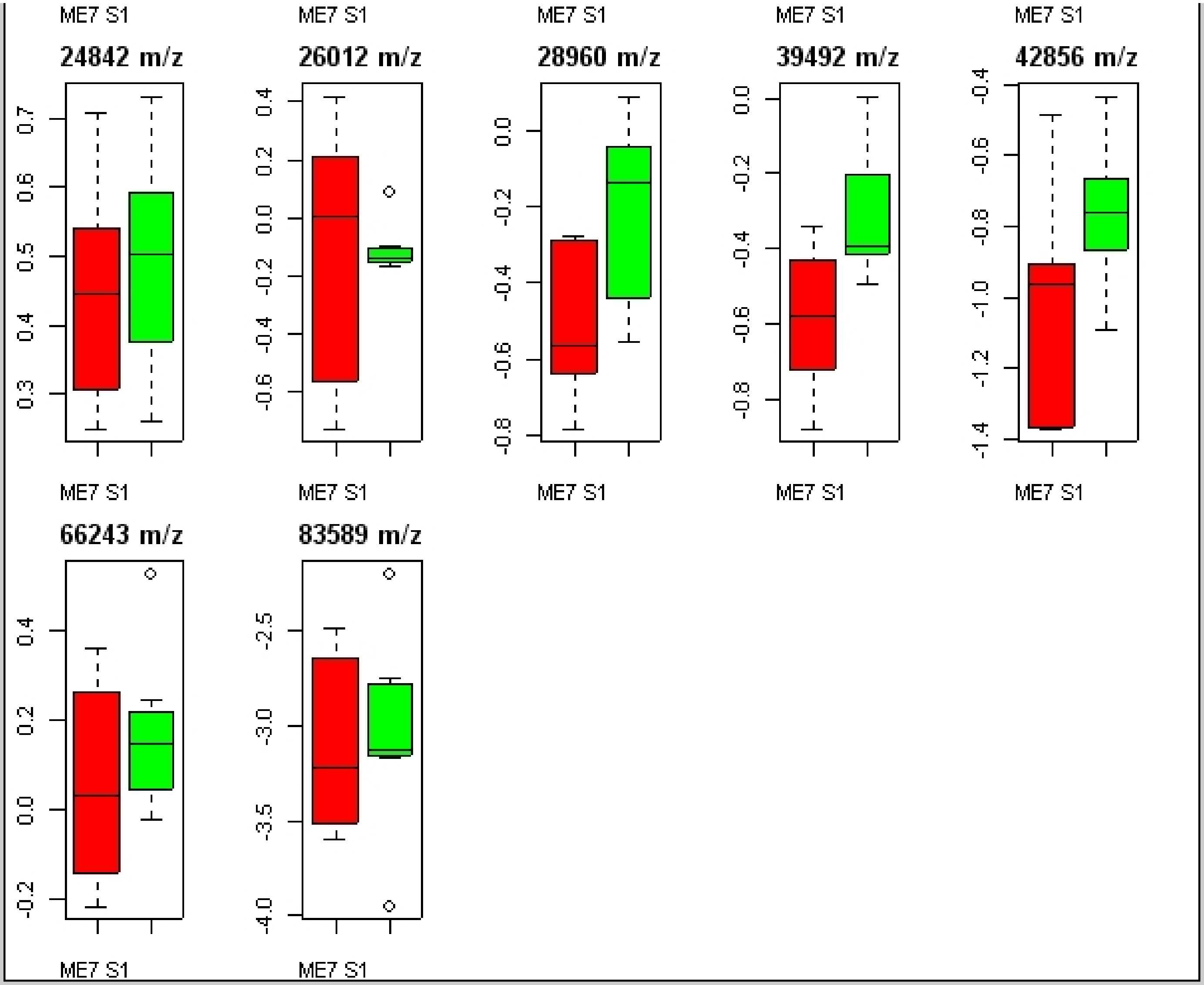

Boxplot of all proteins

Cluster Analysis of samples (Euclidean distance)

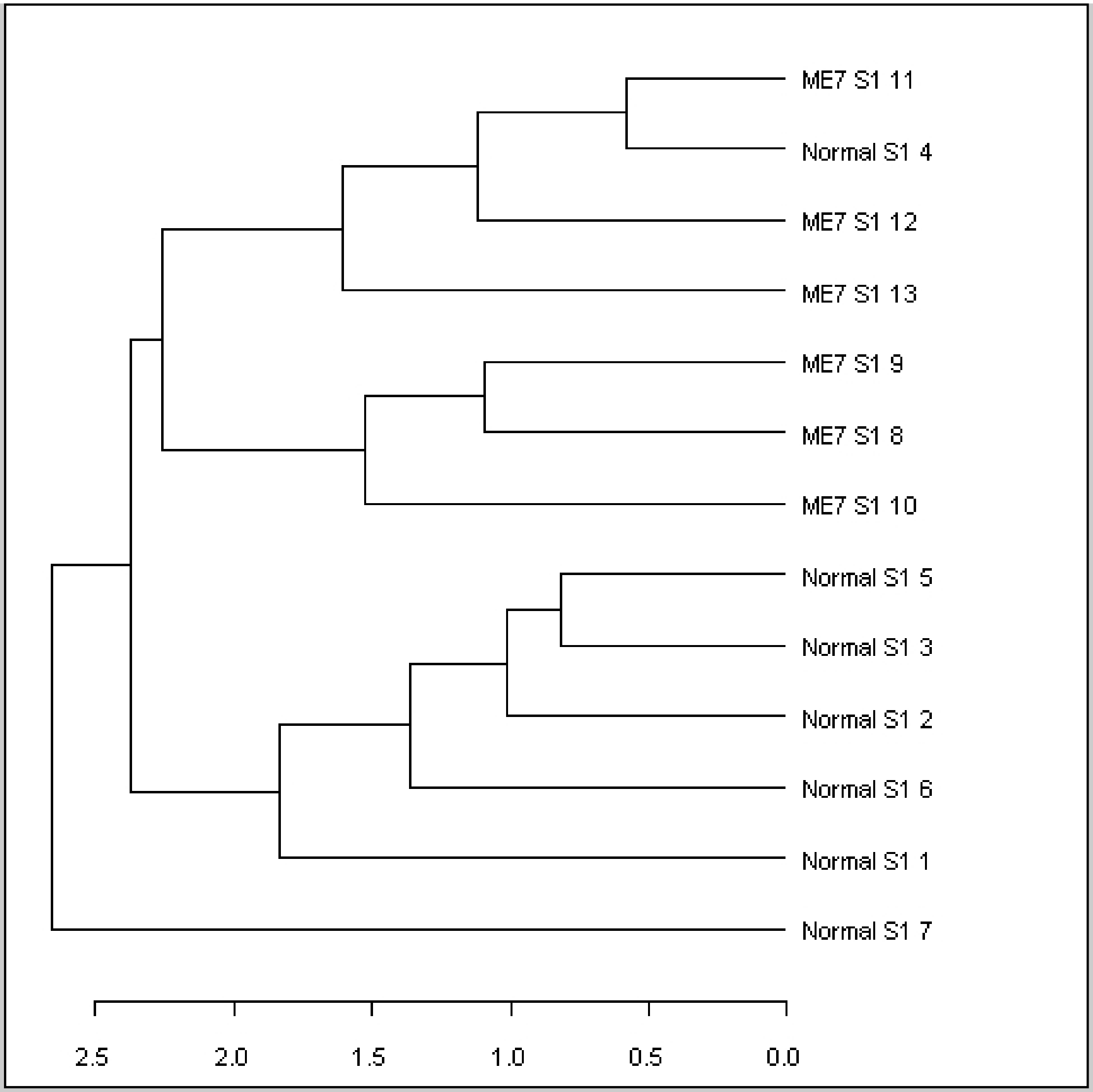

Cluster Analysis of samples (Euclidean distance)

S1 CM10 210

Proteins showing total separation

	C0GROUP	C0GRP_NA	C0Spectr	C09405_7	C010180_	C015714_	C022300_
8	0	ME7 S1	B36260	2.2	3.4	2.6	0.99
9	0	ME7 S1	B36261	2.4	3.1	2.9	0.42
10	0	ME7 S1	B36261	2.5	3.4	3.5	0.57
11	0	ME7 S1	B36262	2.6	3.3	2.5	1.22
12	0	ME7 S1	B36263	2.0	3.7	3.2	0.68
13	0	ME7 S1	B36264	1.9	3.3	2.5	0.65
14	0	ME7 S1	B36265	2.5	3.7	2.3	1.25
1	1	Normal S1	B36248	3.3	2.3	1.7	1.56
2	1	Normal S1	B36249	3.3	2.3	1.3	1.50
3	1	Normal S1	B36250	3.5	2.5	2.2	1.38
4	1	Normal S1	B36251	3.6	2.4	2.0	1.78
5	1	Normal S1	B36252	3.3	2.7	2.1	2.09
6	1	Normal S1	B36253	3.4	2.6	1.9	1.65
7	1	Normal S1	B36253	3.4	2.5	2.2	1.45

Boxplot of proteins showing complete separation

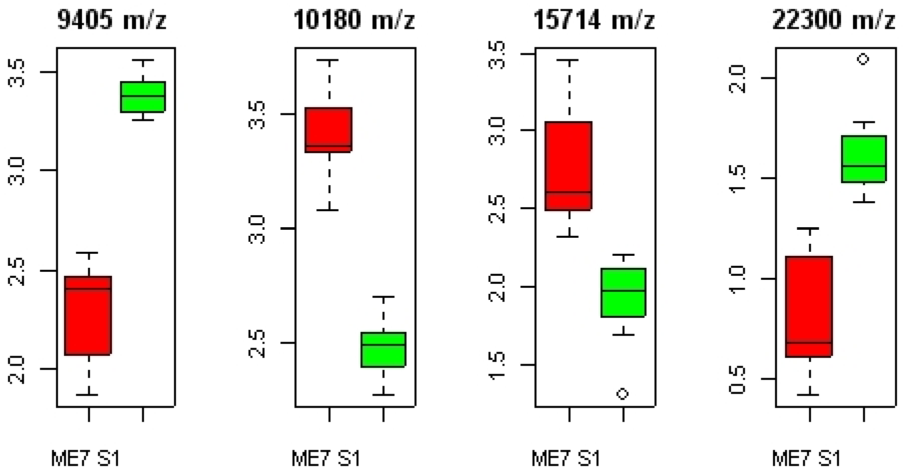

S1 CM10 210

Pairwise Scatterplots of Proteins showing complete separation

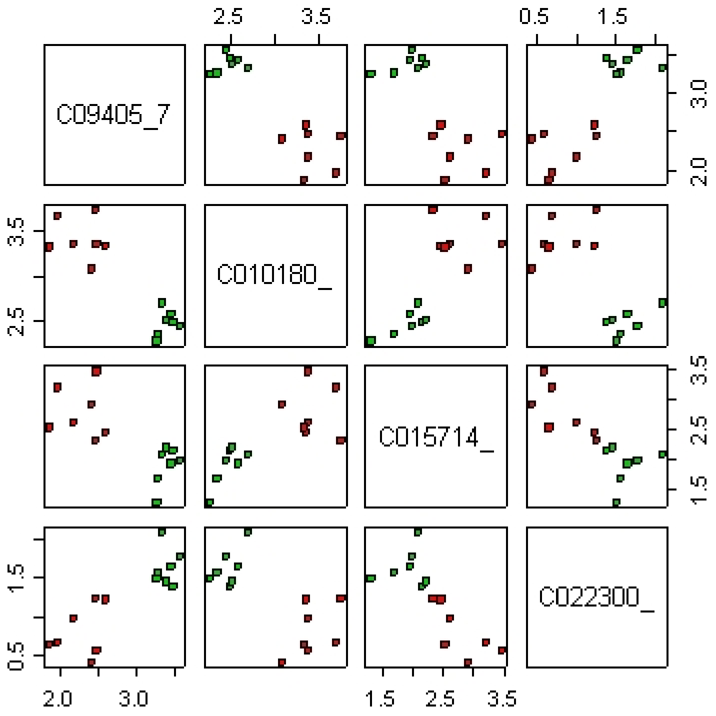

Significant data (p<=0.01)

Significant Proteins (t-test; p<=0.01)

	name	mz	ME7.avg	NORM.avg	t	p
1	C04812_6	4812	3.95	4.21	-3.3	7.5e-03
6	C09405_7	9405	2.28	3.39	-9.8	1.1e-05
8	C010180_	10180	3.41	2.47	9.3	2.8e-06
15	C015714_	15714	2.78	1.90	4.4	9.8e-04
17	C017986_	17986	0.54	1.26	-5.1	5.4e-04
18	C018579_	18579	0.38	0.79	-3.5	5.3e-03
20	C022300_	22300	0.83	1.63	-5.2	2.7e-04
23	C028780_	28780	-0.96	-0.19	-3.9	2.7e-03

S1 CM10 210

Data for Significant proteins

	C0GROUP	C0GRP_NA	C0Spectr	C04812_6	C09405_7	C010180_	C015714_	C017986_	C018579_	C022300_	C028780_
8	0	ME7 S1	B36260	3.9	2.2	3.4	2.6	0.559	0.17	0.99	-0.806
9	0	ME7 S1	B36261	3.8	2.4	3.1	2.9	0.040	0.13	0.42	-1.471
10	0	ME7 S1	B36261	3.8	2.5	3.4	3.5	0.325	0.23	0.57	-1.274
11	0	ME7 S1	B36262	4.1	2.6	3.3	2.5	0.571	0.39	1.22	-0.631
12	0	ME7 S1	B36263	4.0	2.0	3.7	3.2	0.525	0.59	0.68	-1.310
13	0	ME7 S1	B36264	3.8	1.9	3.3	2.5	0.647	0.29	0.65	-1.013
14	0	ME7 S1	B36265	4.3	2.5	3.7	2.3	1.115	0.85	1.25	-0.251
1	1	Normal S1	B36248	4.0	3.3	2.3	1.7	1.371	0.74	1.56	-0.298
2	1	Normal S1	B36249	4.1	3.3	2.3	1.3	1.223	0.44	1.50	-0.345
3	1	Normal S1	B36250	4.2	3.5	2.5	2.2	1.335	0.74	1.38	-0.101
4	1	Normal S1	B36251	4.4	3.6	2.4	2.0	1.270	0.85	1.78	-0.018
5	1	Normal S1	B36252	4.3	3.3	2.7	2.1	1.553	0.94	2.09	0.345
6	1	Normal S1	B36253	4.2	3.4	2.6	1.9	1.157	0.96	1.65	-0.398
7	1	Normal S1	B36253	4.2	3.4	2.5	2.2	0.945	0.87	1.45	-0.542

S1 CM10 210

Boxplot of significant proteins

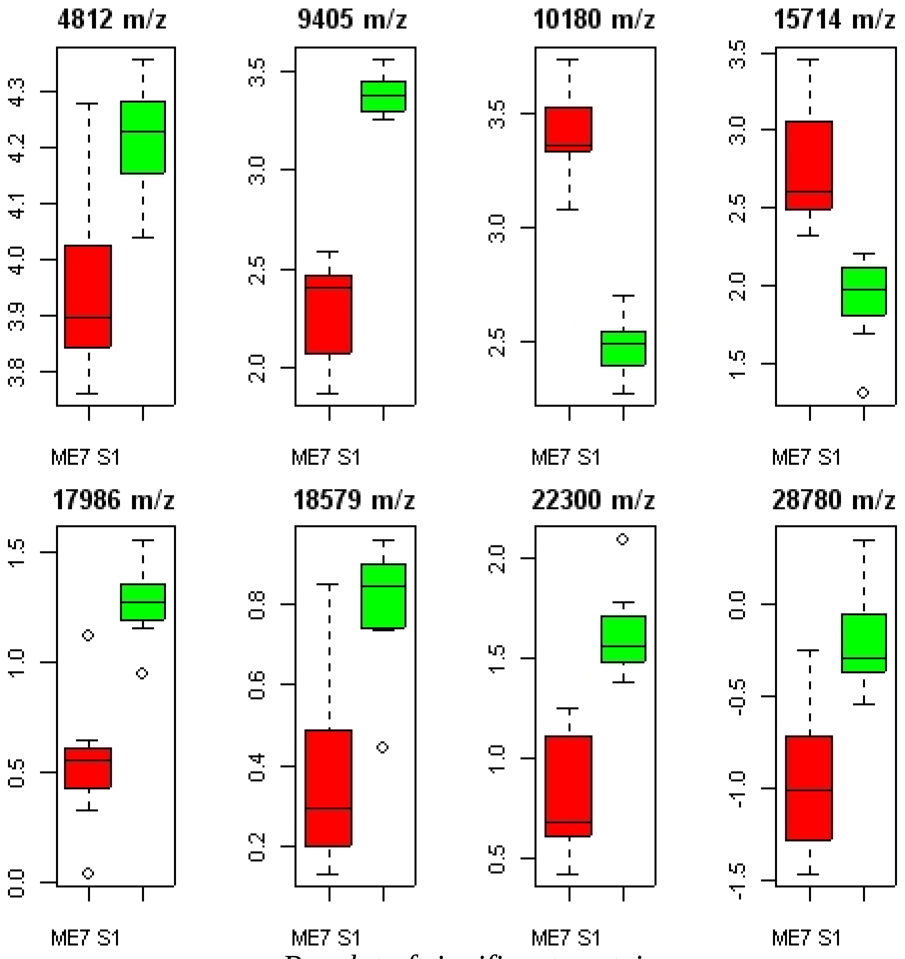

Boxplot of significant proteins

S1 CM10 210

Pairwise Scatterplots of Significant Proteins

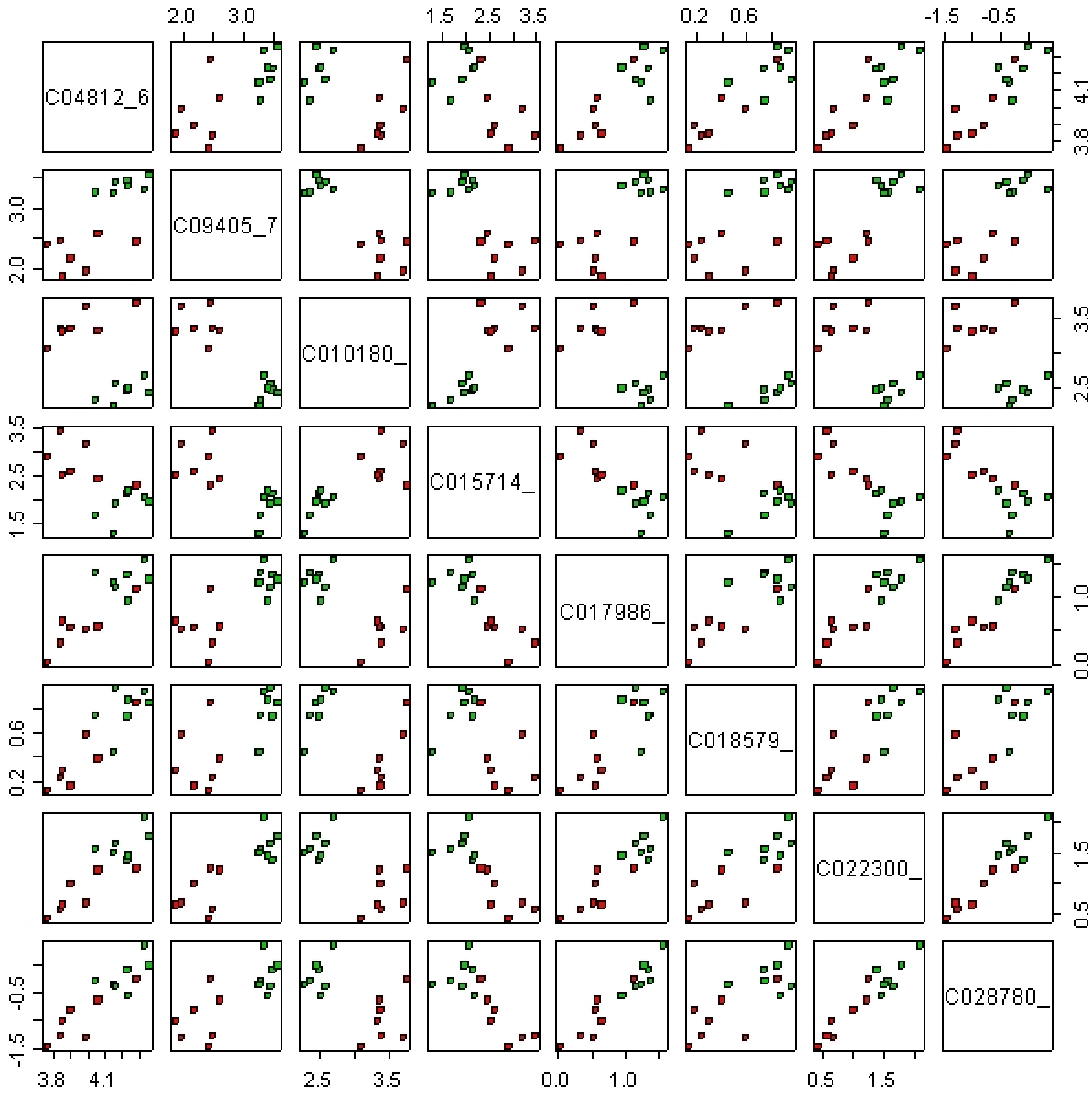

Pairwise Scatterplots of Significant Proteins

S1 CM10 210

Cluster Analysis of samples (Euclidean distance)

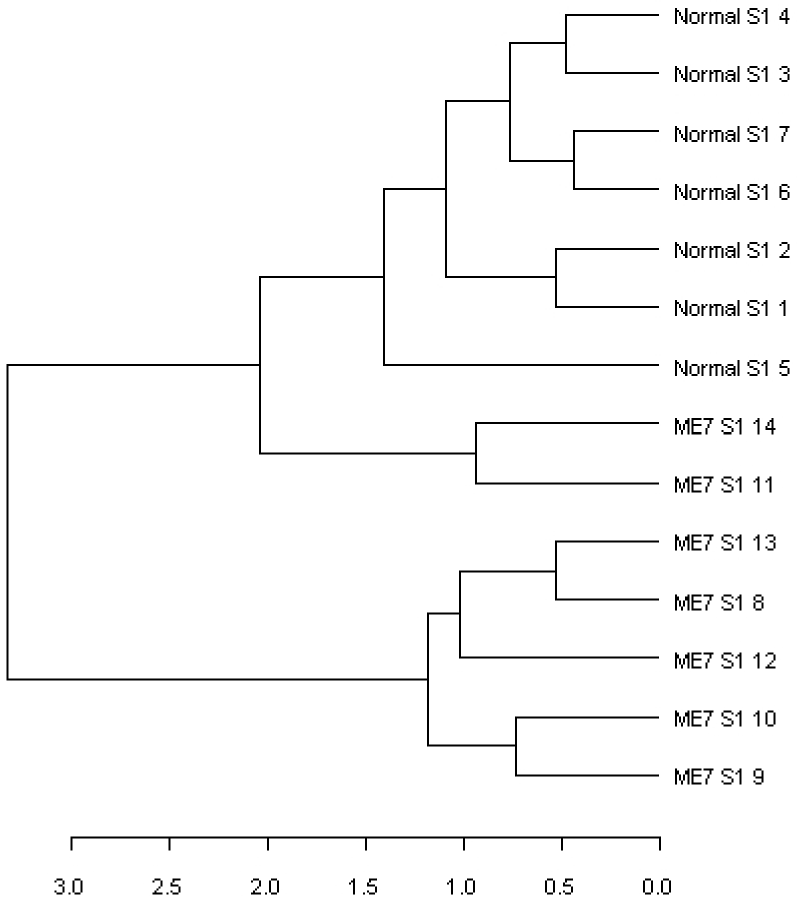

Cluster Analysis of samples (Euclidean distance)

Plot of first three principal components

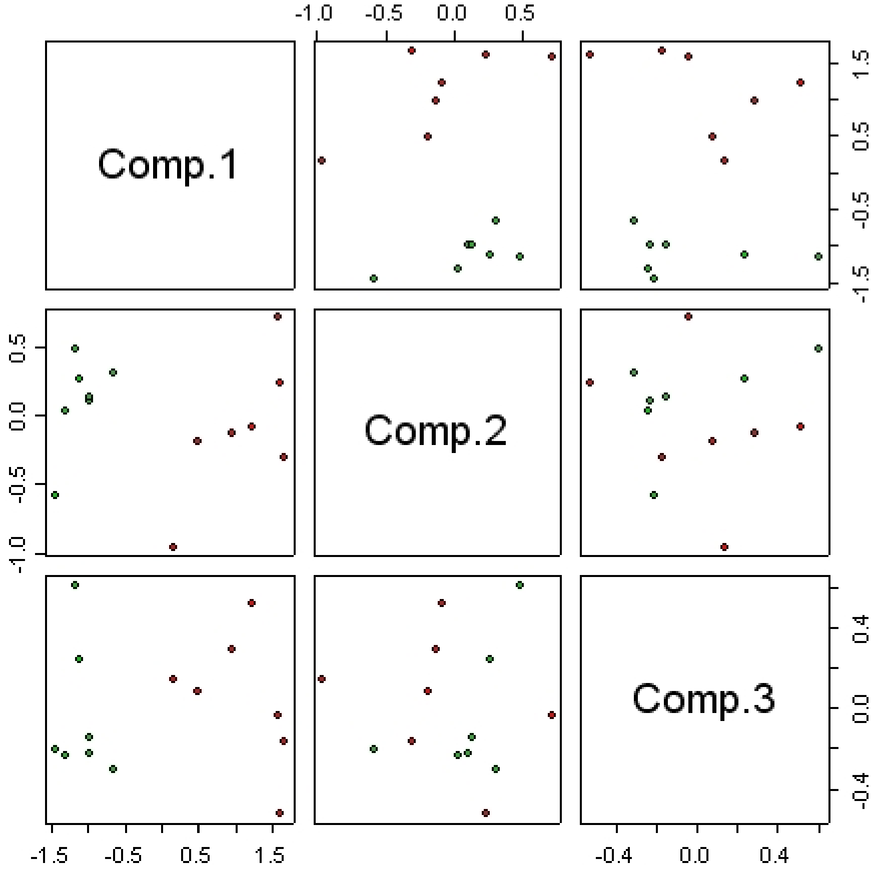

Plot of first three principal components

S1 CM10 210

Scatterplot of linear discriminant function (x-axis)

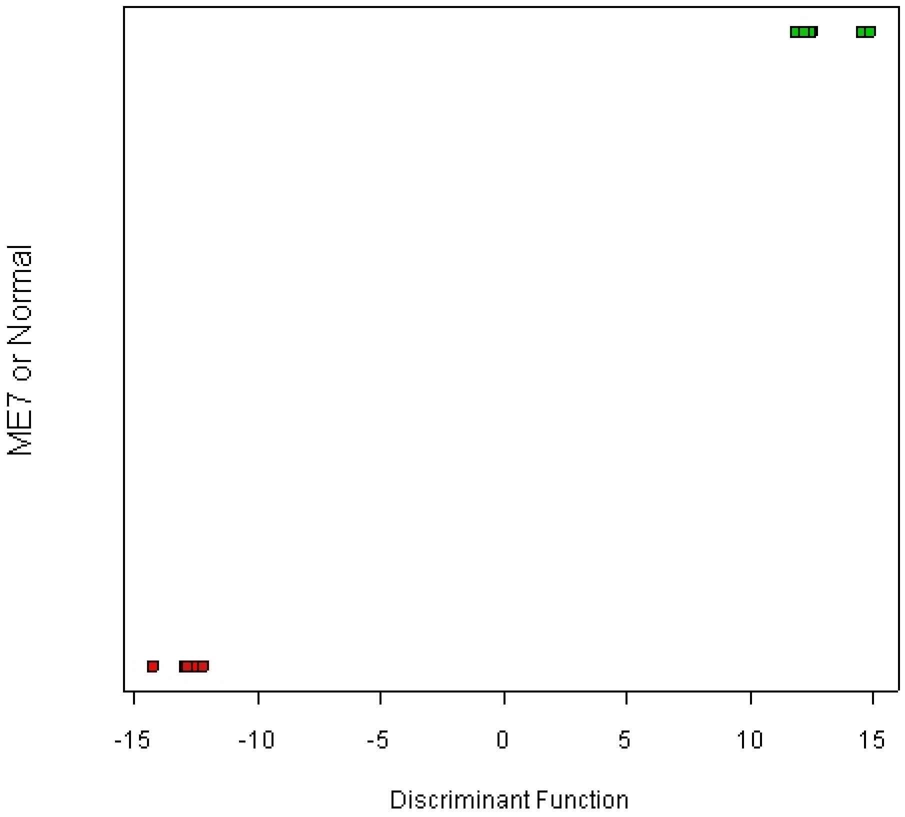

Scatterplot of linear discriminant function (x-axis)

S1 CM10 210

All data *Boxplot of all proteins*

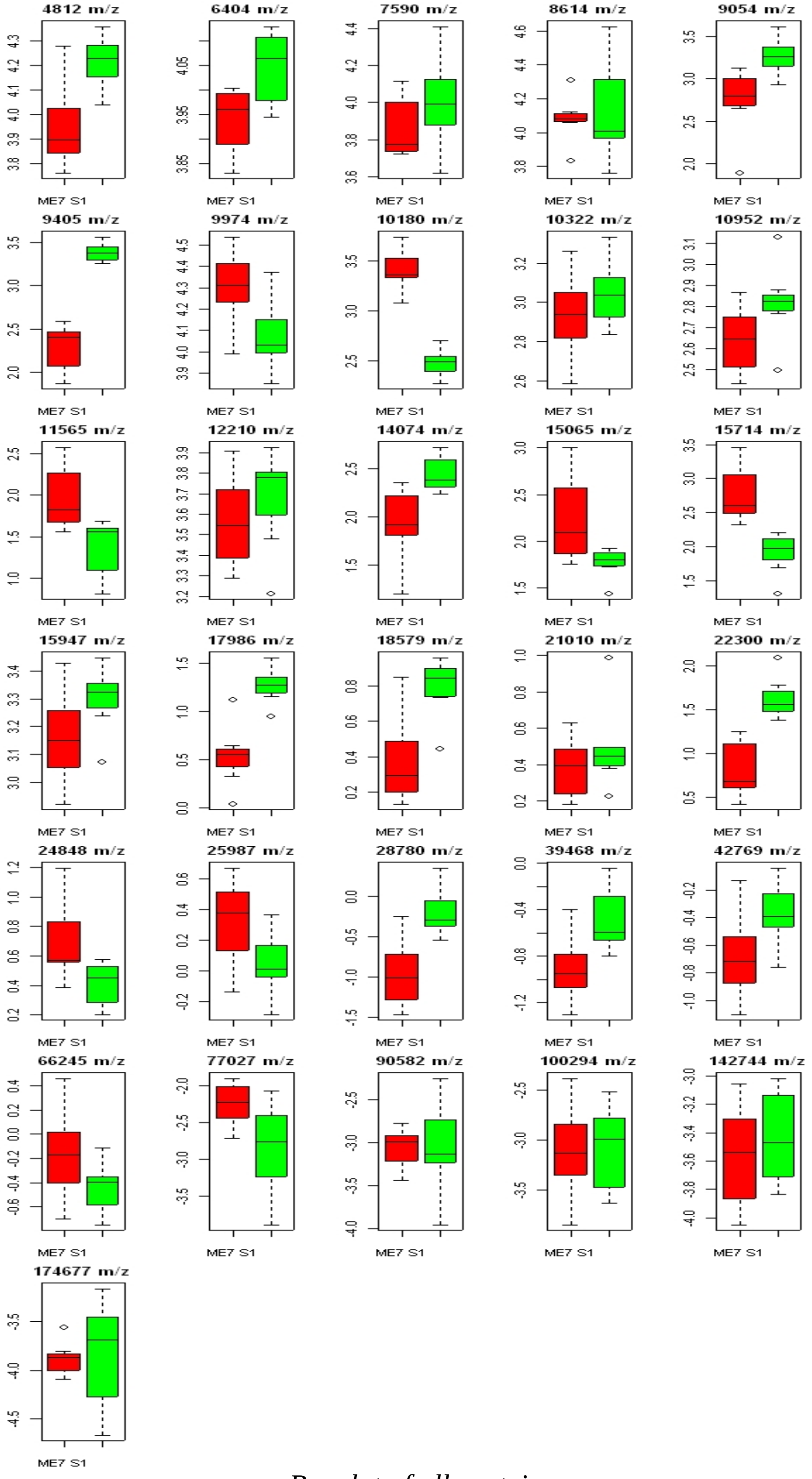

Boxplot of all proteins

S1 CM10 210

Cluster Analysis of samples (Euclidean distance)

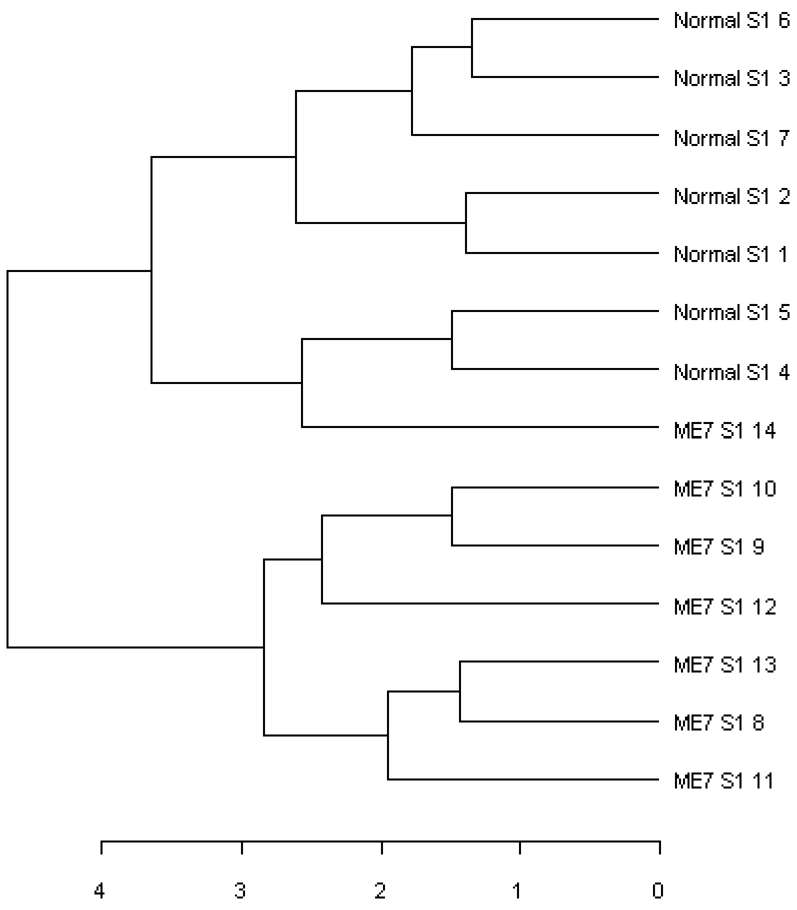

Cluster Analysis of samples (Euclidean distance)

S1 CM10 240

Proteins showing total separation

No proteins showed complete separation

Significant data (p<=0.05)

Significant Proteins (t-test; p<=0.05)

	name	mz	ME7.avg	NORM.avg	t	p
23	C022303_	22303	0.82	1.7	-2.7	0.019

Data for Significant proteins

	C0GROUP	C0GRP_NA	C0Spectr	C022303_
8	0	ME7 S1	B36863	1.03
9	0	ME7 S1	B36864	0.96
10	0	ME7 S1	B36865	1.67
11	0	ME7 S1	B36865	-0.26
12	0	ME7 S1	B36866	1.14
13	0	ME7 S1	B36867	0.99
14	0	ME7 S1	B36868	0.20
1	1	Normal S1	B36857	1.46
2	1	Normal S1	B36858	1.80
3	1	Normal S1	B36859	1.93
4	1	Normal S1	B36860	0.56
5	1	Normal S1	B36860	1.86
6	1	Normal S1	B36861	1.94
7	1	Normal S1	B36862	2.27

Boxplot of significant proteins

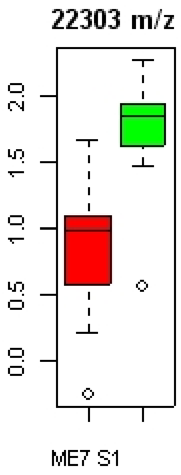

Boxplot of significant proteins

All data

Boxplot of all proteins

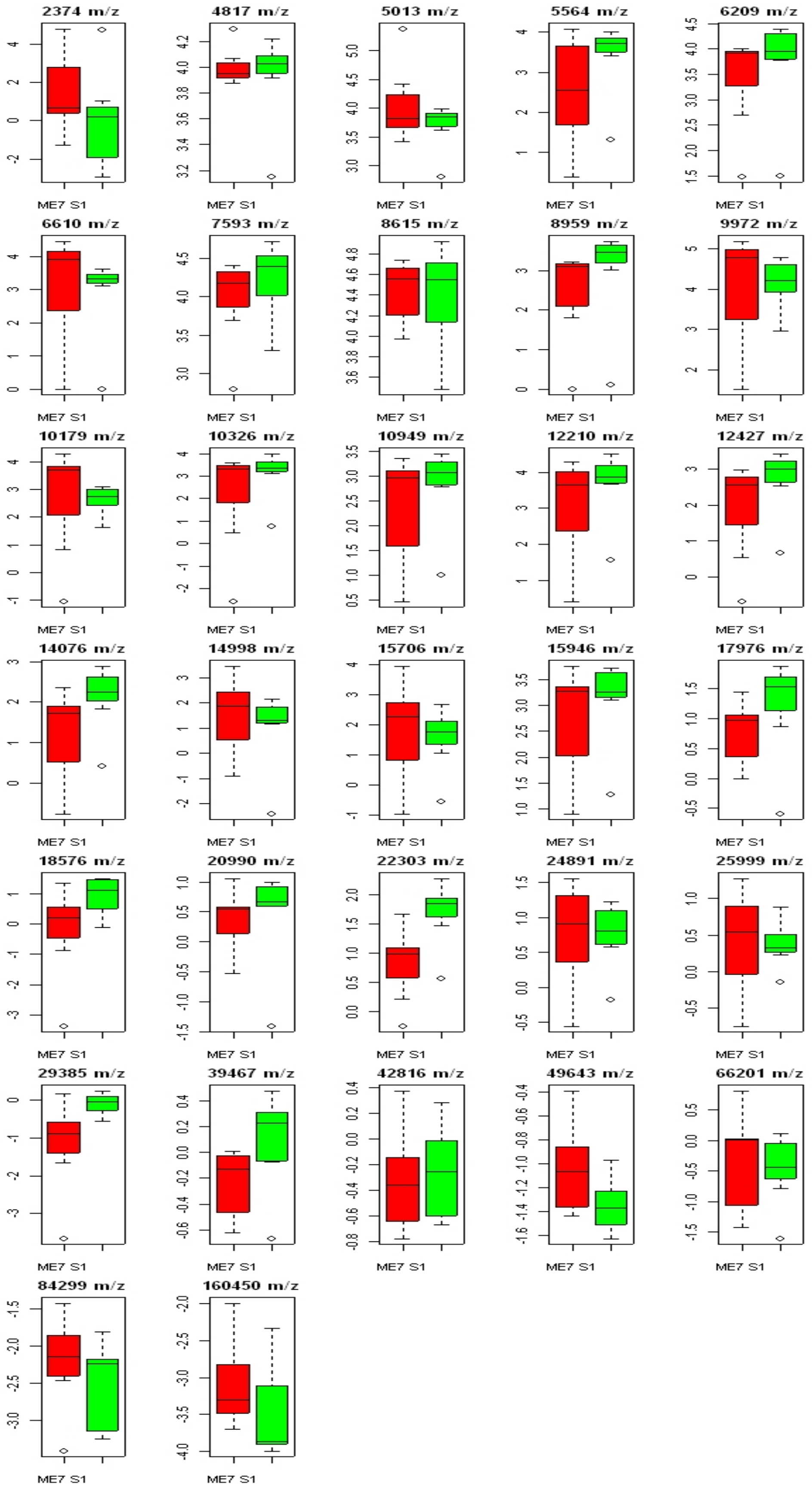

Boxplot of all proteins

Cluster Analysis of samples (Euclidean distance)

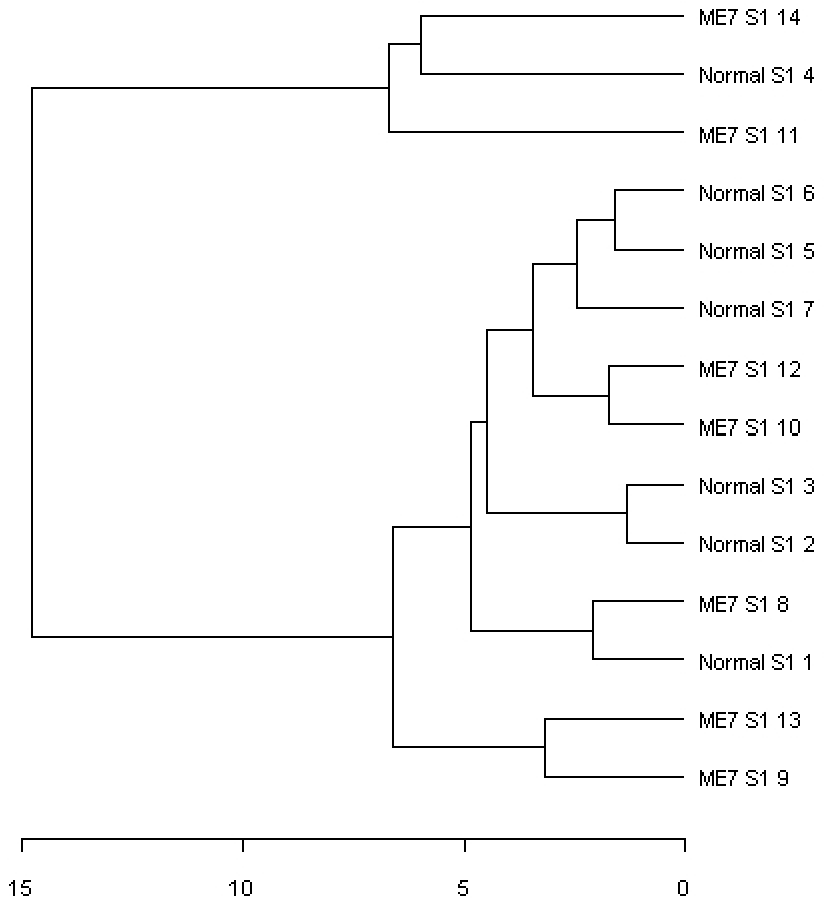

Cluster Analysis of samples (Euclidean distance)

S1 CM10 EP

Proteins showing total separation

	C0GROUP	C0GRP_NA	C0Spectr	C010189_	C013912_	C019956_	C022309_	C024848_	C034200_
1	0	ME7 S1	B36885	3.8	1.20	0.59	1.09	1.45	-1.00
3	0	ME7 S1	B37003	4.1	1.55	0.81	0.89	1.56	-0.77
4	0	ME7 S1	B37004	4.1	0.71	0.97	1.24	1.33	-0.58
5	0	ME7 S1	B37020	3.9	0.97	0.94	0.89	1.83	-0.48
7	0	ME7 S1	B37134	4.1	1.17	1.01	0.99	1.39	-0.76
8	0	ME7 S1	B37283	4.3	1.59	1.31	1.28	1.60	-0.34
10	0	ME7 S1	B37285	3.7	1.25	0.35	0.99	1.52	-1.30
2	1	Normal S1	B37002	2.3	2.79	-0.47	1.46	0.45	-2.96
6	1	Normal S1	B37133	2.7	2.68	-0.38	1.94	1.07	-1.41
9	1	Normal S1	B37284	2.8	2.63	-0.63	1.84	0.99	-1.74
11	1	Normal S1	B37329	2.5	2.55	-0.78	2.14	0.77	-2.57
12	1	Normal S1	B37499	3.0	2.81	-0.23	2.07	1.09	-2.33
13	1	Normal S1	B37500	2.7	2.53	-0.17	1.83	0.97	-2.09
14	1	Normal S1	B37501	2.3	2.60	0.27	1.44	0.62	-2.20

Boxplot of proteins showing complete separation

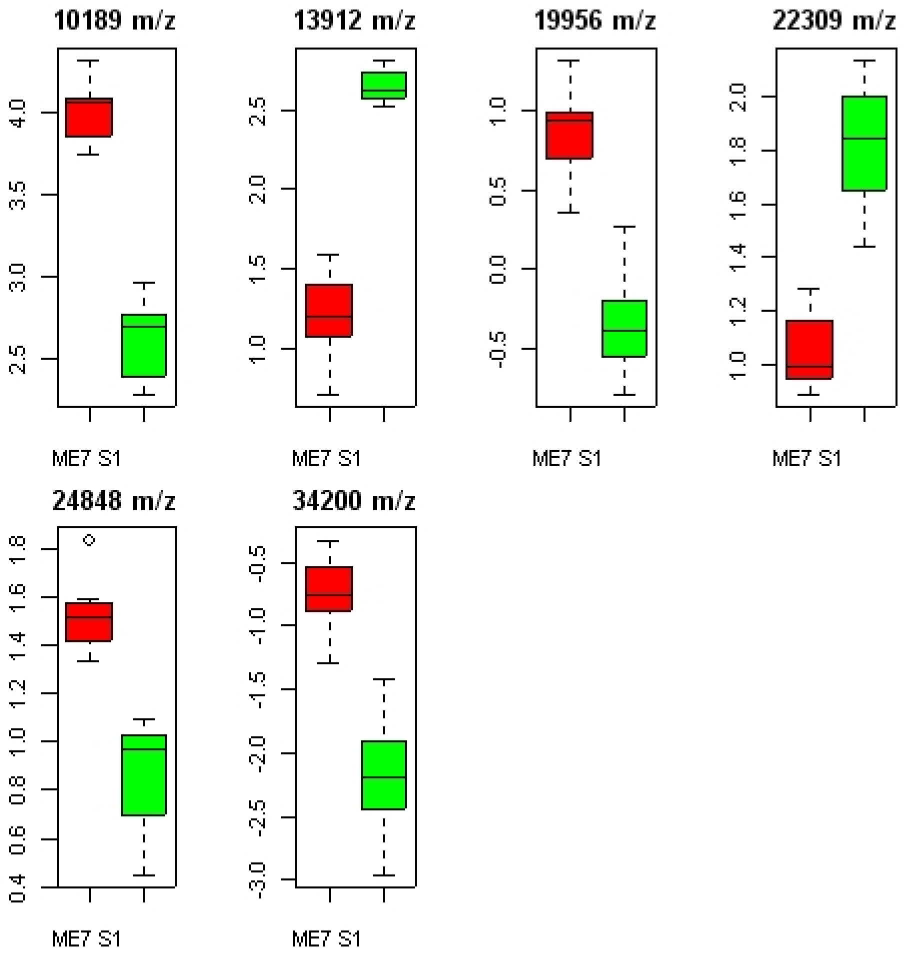

Pairwise Scatterplots of Proteins showing complete separation

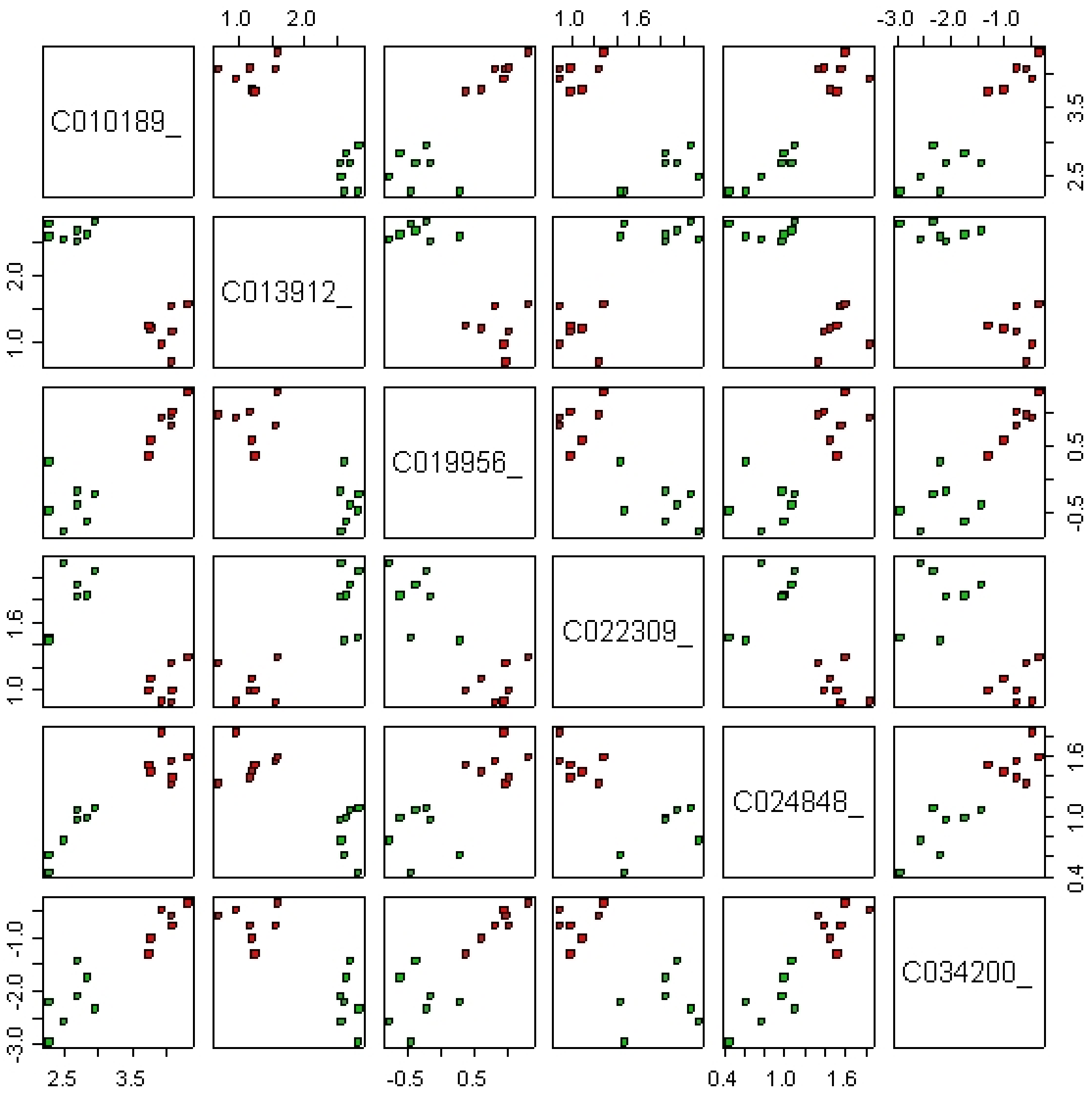

Significant data (p<=0.01)

Significant Proteins (t-test; p<=0.01)

	name	mz	ME7.avg	NORM.avg	t	p
10	C010189_	10189	4.00	2.604	11.2	2.0e-07
14	C013912_	13912	1.21	2.655	-11.7	4.1e-06
20	C017988_	17988	0.91	1.468	-4.8	5.3e-04
22	C019956_	19956	0.85	-0.342	6.9	1.8e-05
24	C022309_	22309	1.05	1.818	-6.4	9.5e-05
25	C024848_	24848	1.52	0.851	6.0	1.1e-04
26	C026014_	26014	0.98	0.349	4.7	5.8e-04
27	C028720_	28720	-0.61	-0.025	-4.5	8.3e-04
28	C034200_	34200	-0.75	-2.184	6.3	8.7e-05

Data for Significant proteins

	C0GROUP	C0GRP_NA	C0Spectr	C010189_	C013912_	C017988_	C019956_	C022309_	C024848_	C026014_	C028720_	C034200_
1	0	ME7 S1	B36885	3.8	1.20	0.89	0.59	1.09	1.45	0.897	-0.669	-1.00
3	0	ME7 S1	B37003	4.1	1.55	1.06	0.81	0.89	1.56	1.041	-0.560	-0.77
4	0	ME7 S1	B37004	4.1	0.71	0.61	0.97	1.24	1.33	0.689	-0.627	-0.58
5	0	ME7 S1	B37020	3.9	0.97	1.17	0.94	0.89	1.83	1.323	-0.811	-0.48
7	0	ME7 S1	B37134	4.1	1.17	0.79	1.01	0.99	1.39	0.800	-0.623	-0.76
8	0	ME7 S1	B37283	4.3	1.59	0.99	1.31	1.28	1.60	1.178	-0.185	-0.34
10	0	ME7 S1	B37285	3.7	1.25	0.89	0.35	0.99	1.52	0.923	-0.790	-1.30
2	1	Normal S1	B37002	2.3	2.79	1.18	-0.47	1.46	0.45	-0.099	-0.488	-2.96
6	1	Normal S1	B37133	2.7	2.68	1.75	-0.38	1.94	1.07	0.753	0.248	-1.41
9	1	Normal S1	B37284	2.8	2.63	1.63	-0.63	1.84	0.99	0.415	0.295	-1.74
11	1	Normal S1	B37329	2.5	2.55	1.53	-0.78	2.14	0.77	0.321	-0.172	-2.57
12	1	Normal S1	B37499	3.0	2.81	1.61	-0.23	2.07	1.09	0.342	-0.028	-2.33
13	1	Normal S1	B37500	2.7	2.53	1.50	-0.17	1.83	0.97	0.570	0.110	-2.09
14	1	Normal S1	B37501	2.3	2.60	1.08	0.27	1.44	0.62	0.136	-0.138	-2.20

Boxplot of significant proteins

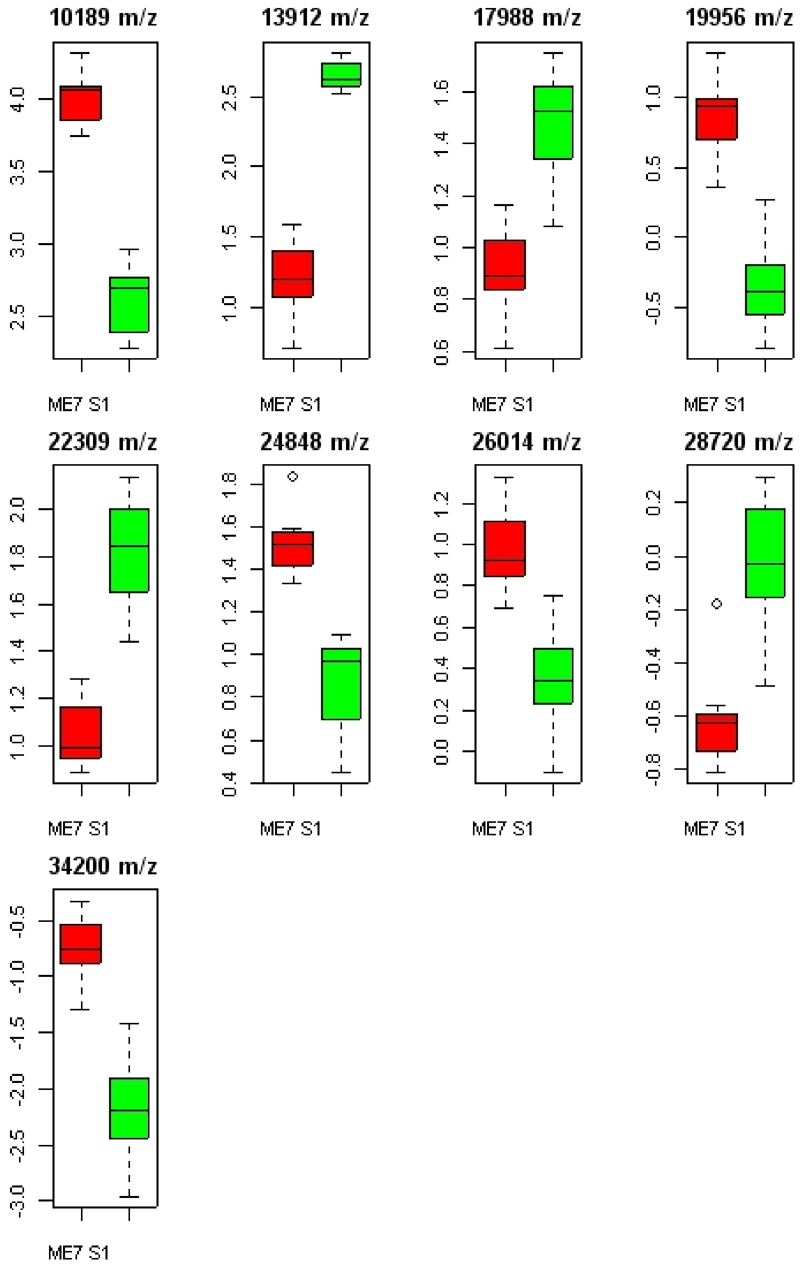

Boxplot of significant proteins

Pairwise Scatterplots of Significant Proteins

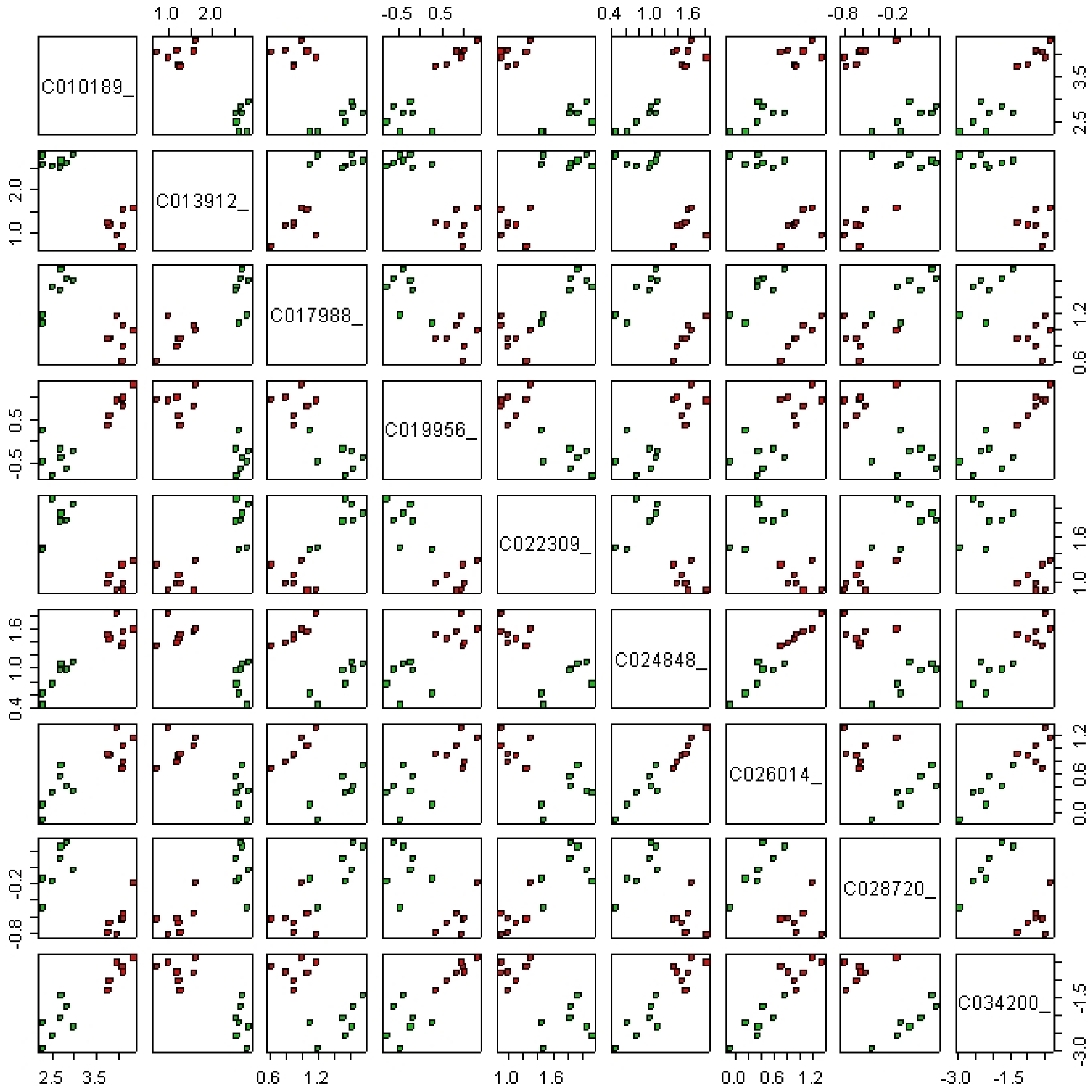

Pairwise Scatterplots of Significant Proteins

Cluster Analysis of samples (Euclidean distance)

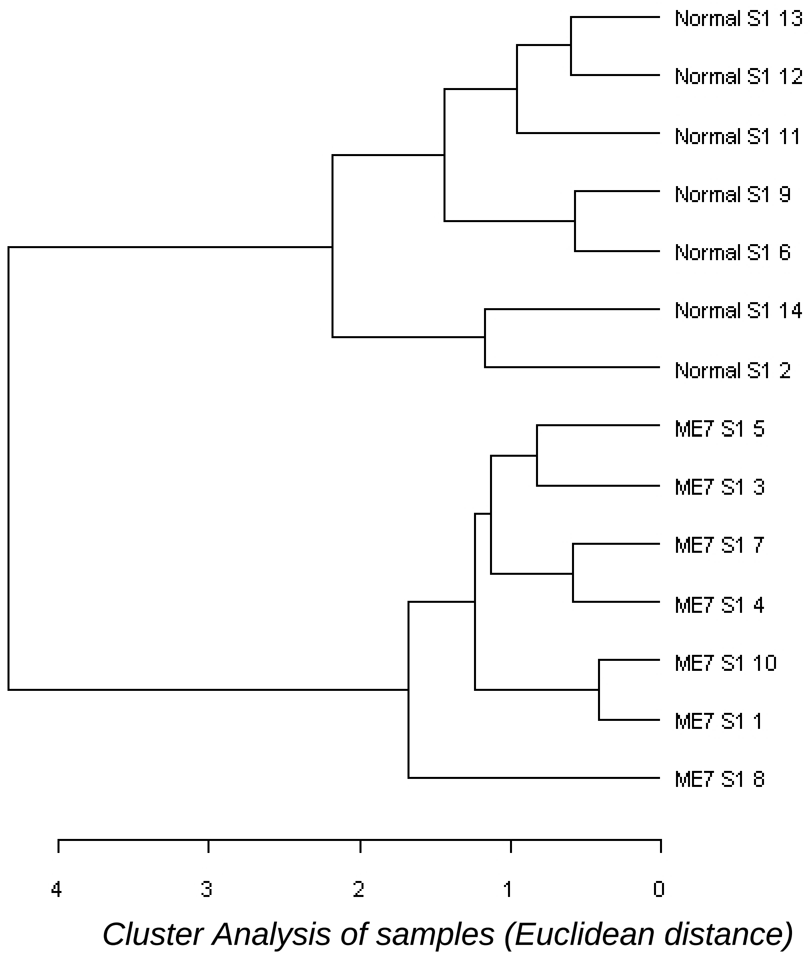

Plot of first three principal components

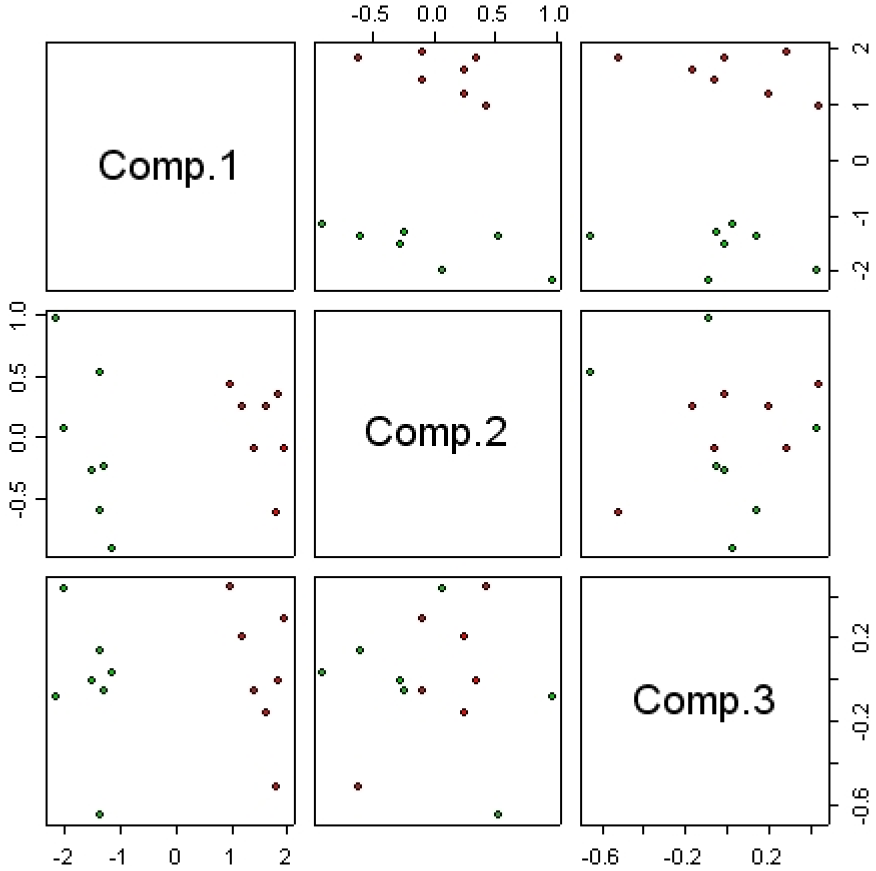

Plot of first three principal components

Scatterplot of linear discriminant function (x-axis)

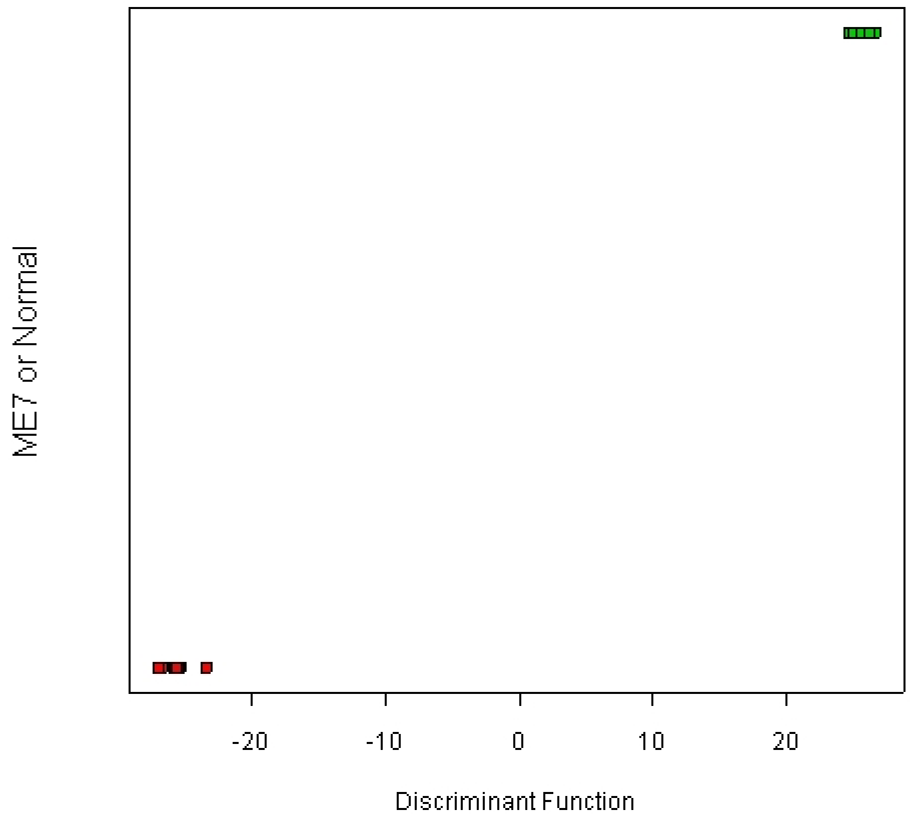

Scatterplot of linear discriminant function (x-axis)

All data

Boxplot of all proteins

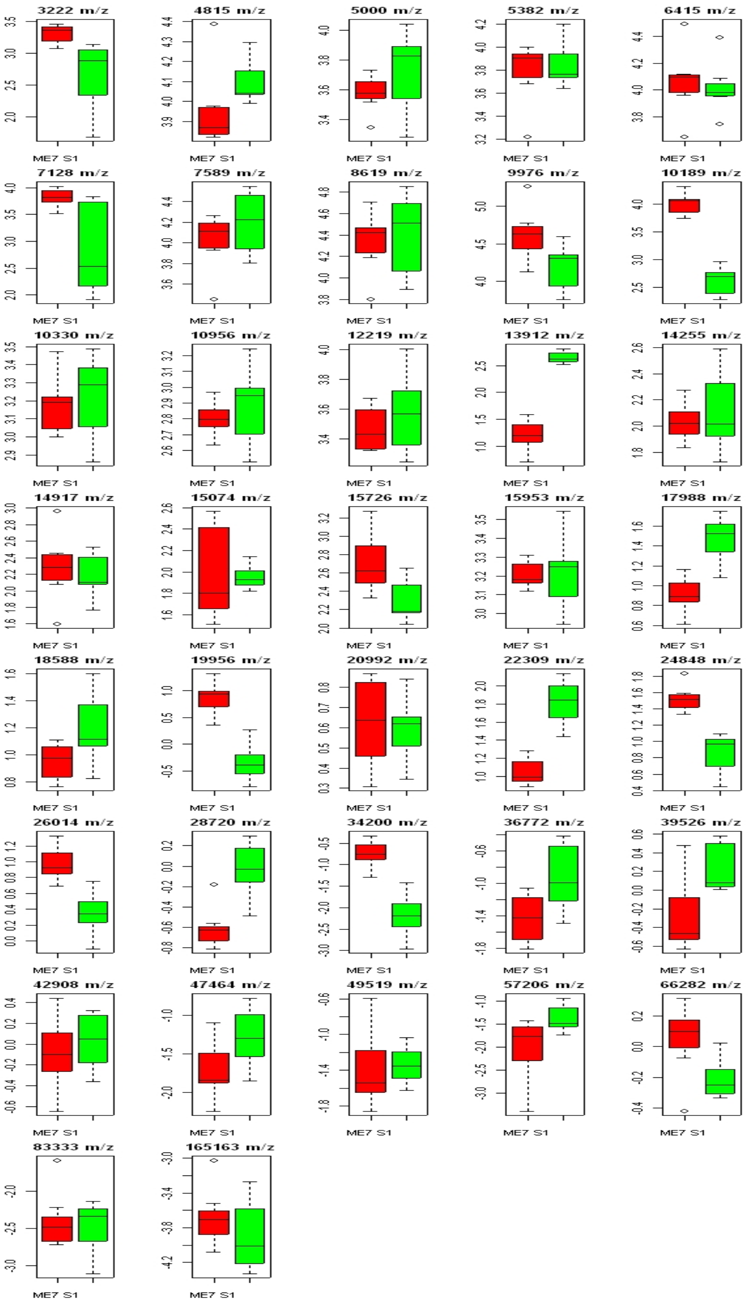

Boxplot of all proteins

Cluster Analysis of samples (Euclidean distance)

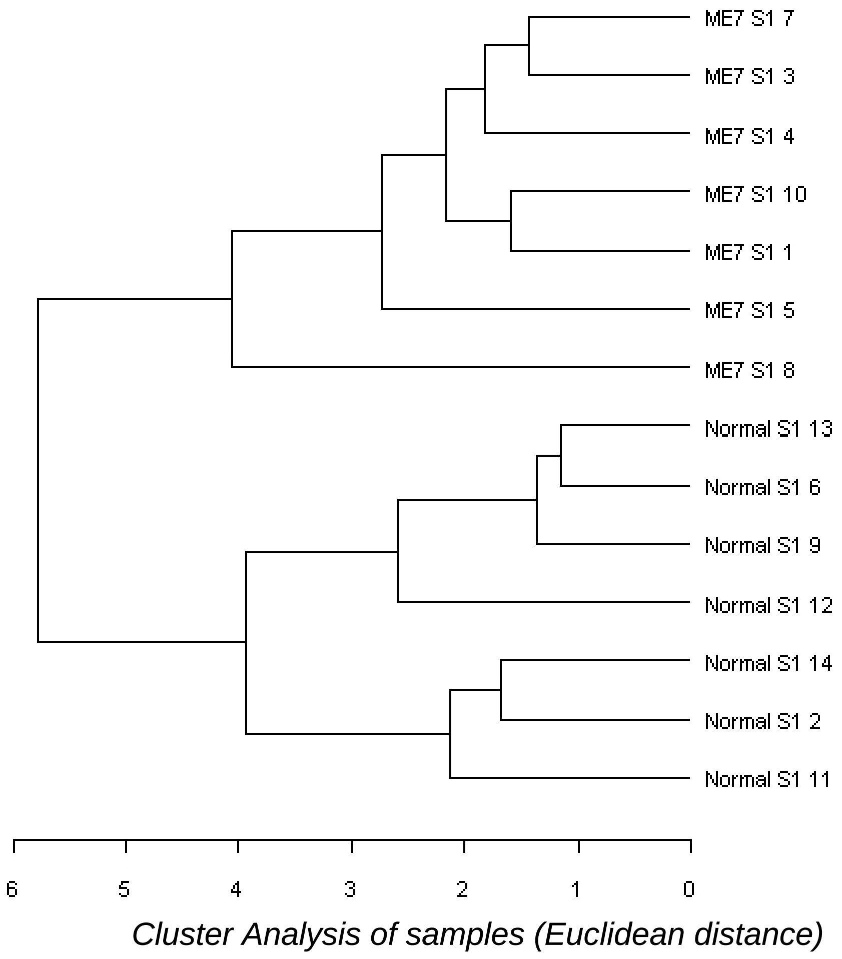

Supplement: Additional file 6 — Full statistical analysis of CM10 supernatant 1 arrays [file 1471-2334-9-188-S6.PDF]
