# Supplementary material for: Differential protein profiling as a potential multi-marker approach for TSE diagnosis
Source: BMC Infect Dis. 2009 Nov 27;9:188. doi: 10.1186/1471-2334-9-188 (PMC2794872; doi:10.1186/1471-2334-9-188)
Supplement: Additional file 7 — Full statistical analysis of Q10 supernatant 1 arrays [file 1471-2334-9-188-S7.PDF]

S1 Q10 30

Proteins showing total separation

No proteins showed complete separation

Significant data (p<=0.05)

Significant Proteins (t-test; p<=0.05)

	name	mz	ME7.avg	NORM.avg	t	p
3	C02400_9	2400	1.57	0.90	2.2	0.0429
28	C012246_	12246	1.68	1.16	3.0	0.0079
29	C013037_	13037	1.56	1.25	2.3	0.0391
41	C035510_	35510	-0.28	0.13	-2.3	0.0342
45	C059642_	59642	-1.55	-1.22	-2.4	0.0323
46	C064051_	64051	-1.63	-1.32	-2.5	0.0242

Data for Significant proteins

	C0GROUP	C0GRP_NA	C0Spectr	C02400_9	C012246_	C013037_	C035510_	C059642_	C064051_
13	0	ME7 S1	Q10 111004-E 1	2.53	1.95	1.80	-0.5424	-2.01	-1.7
14	0	ME7 S1	Q10 111004-C 1	1.45	1.60	1.46	-0.5702	-1.48	-1.5
15	0	ME7 S1	Q10 111004-C 2	1.55	1.40	1.53	-0.0023	-1.27	-1.4
16	0	ME7 S1	Q10 111004-A 1	1.71	1.75	1.34	-0.3301	-1.77	-1.6
17	0	ME7 S1	Q10 111004-A 2	1.09	1.86	1.47	0.2514	-1.33	-1.4
18	0	ME7 S1	Q10 111004-B 1	1.24	1.76	1.72	-0.6004	-1.62	-2.1
19	0	ME7 S1	Q10 111004-B 2	1.39	1.46	1.59	-0.1857	-1.38	-1.7
1	1	Normal S1	Q10 111004-H 1	0.50	1.04	1.18	0.1353	-1.25	-1.6
2	1	Normal S1	Q10 111004-H 2	1.21	1.24	1.20	0.6892	-0.67	-1.0
3	1	Normal S1	Q10 111004-A 1	2.22	-0.24	0.25	-0.4576	-1.37	-1.5
4	1	Normal S1	Q10 111004-B 1	2.14	0.53	0.76	-0.1893	-1.22	-1.5
5	1	Normal S1	Q10 111004-C 1	1.81	1.65	1.68	-0.2084	-1.95	-1.4
6	1	Normal S1	Q10 111004-D 1	0.27	1.52	1.49	0.0071	-1.47	-1.2
7	1	Normal S1	Q10 111004-E 1	-0.53	1.21	1.55	0.0670	-1.31	-1.6
8	1	Normal S1	Q10 111004-E 2	0.59	1.54	1.36	0.4027	-0.93	-1.0
9	1	Normal S1	Q10 111004-F 1	0.41	1.30	1.68	-0.4613	-1.45	-1.6
10	1	Normal S1	Q10 111004-F 2	1.48	1.47	1.61	-0.0072	-1.16	-1.5
11	1	Normal S1	Q10 111004-G 1	0.13	1.46	1.17	0.7601	-0.91	-0.7
12	1	Normal S1	Q10 111004-G 2	0.53	1.17	1.11	0.7860	-0.96	-1.1

Boxplot of significant proteins

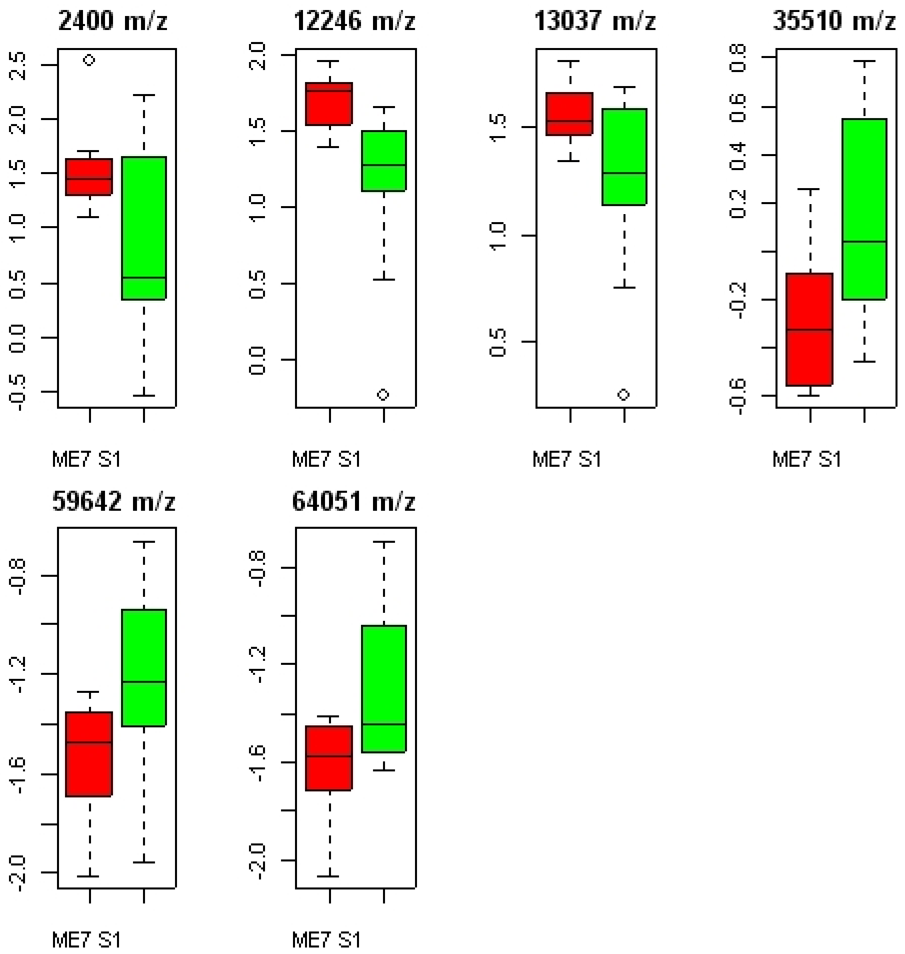

Boxplot of significant proteins

Pairwise Scatterplots of Significant Proteins

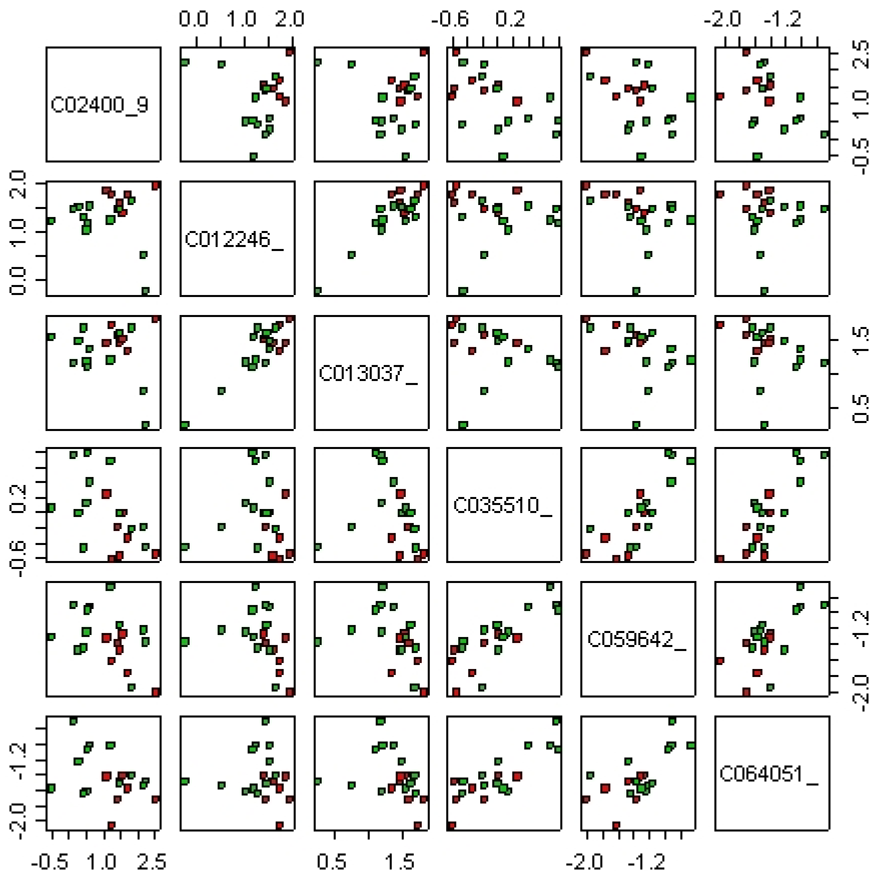

Pairwise Scatterplots of Significant Proteins

Cluster Analysis of samples (Euclidean distance)

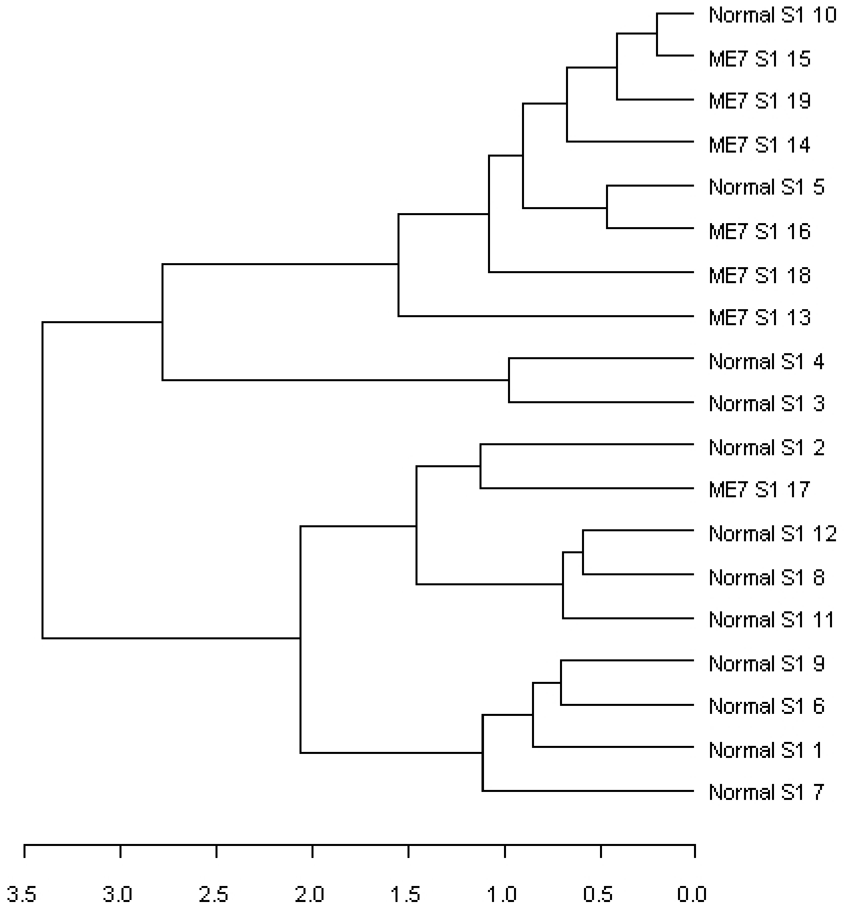

Cluster Analysis of samples (Euclidean distance)

Plot of first three principal components

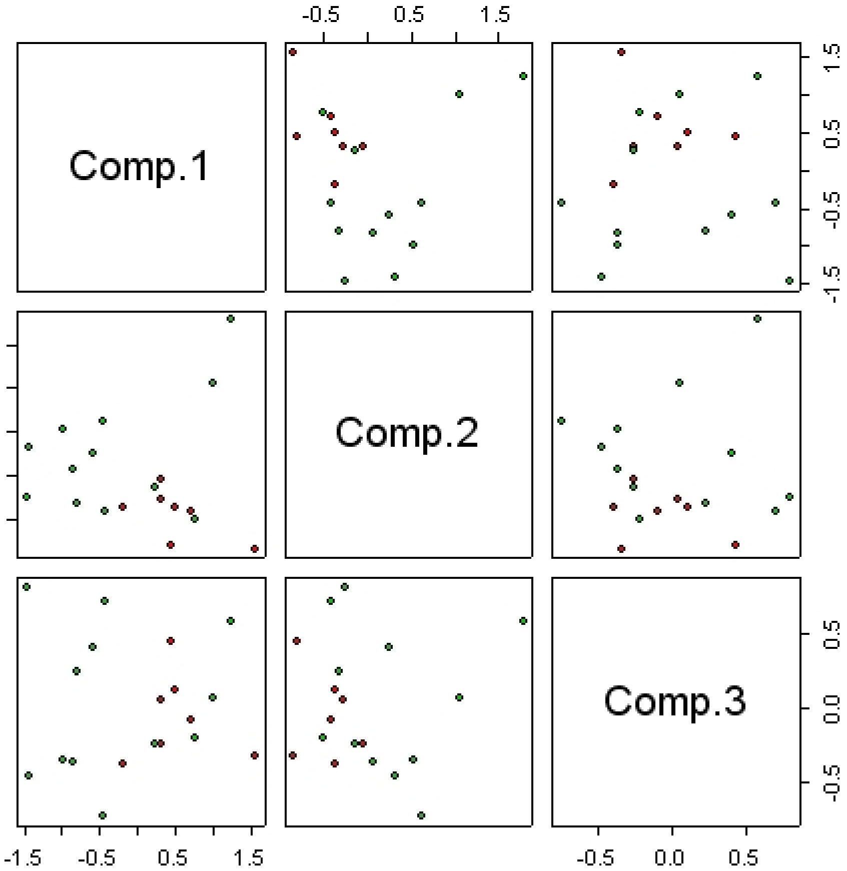

Plot of first three principal components

Scatterplot of linear discriminant function (x-axis)

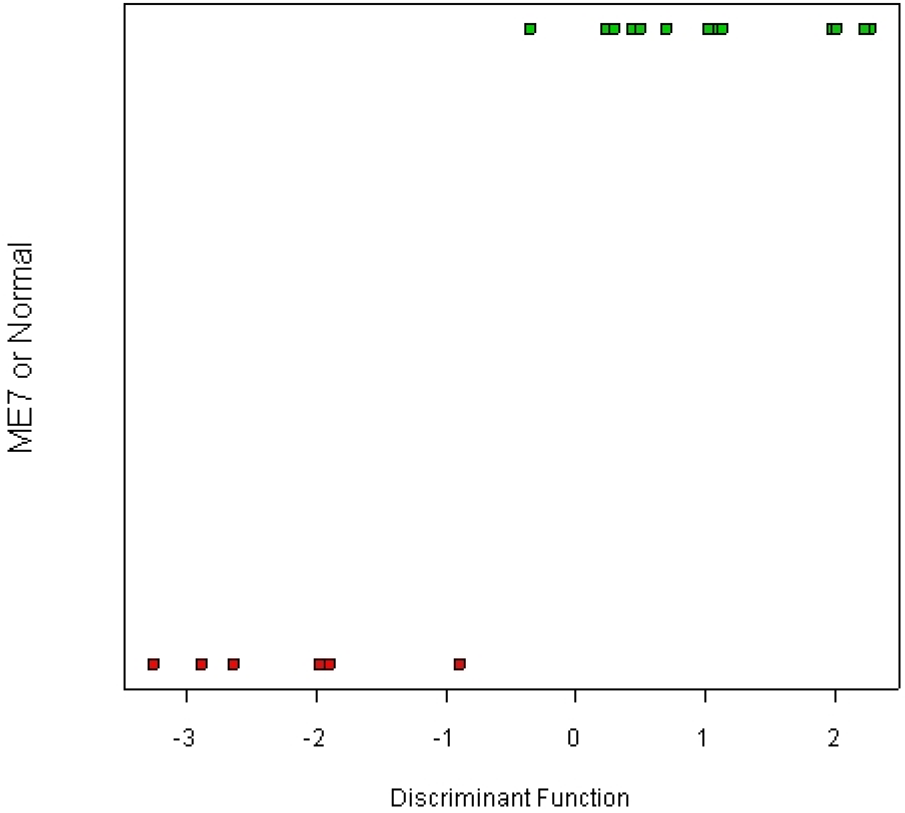

Scatterplot of linear discriminant function (x-axis)

All data *Boxplot of all proteins*

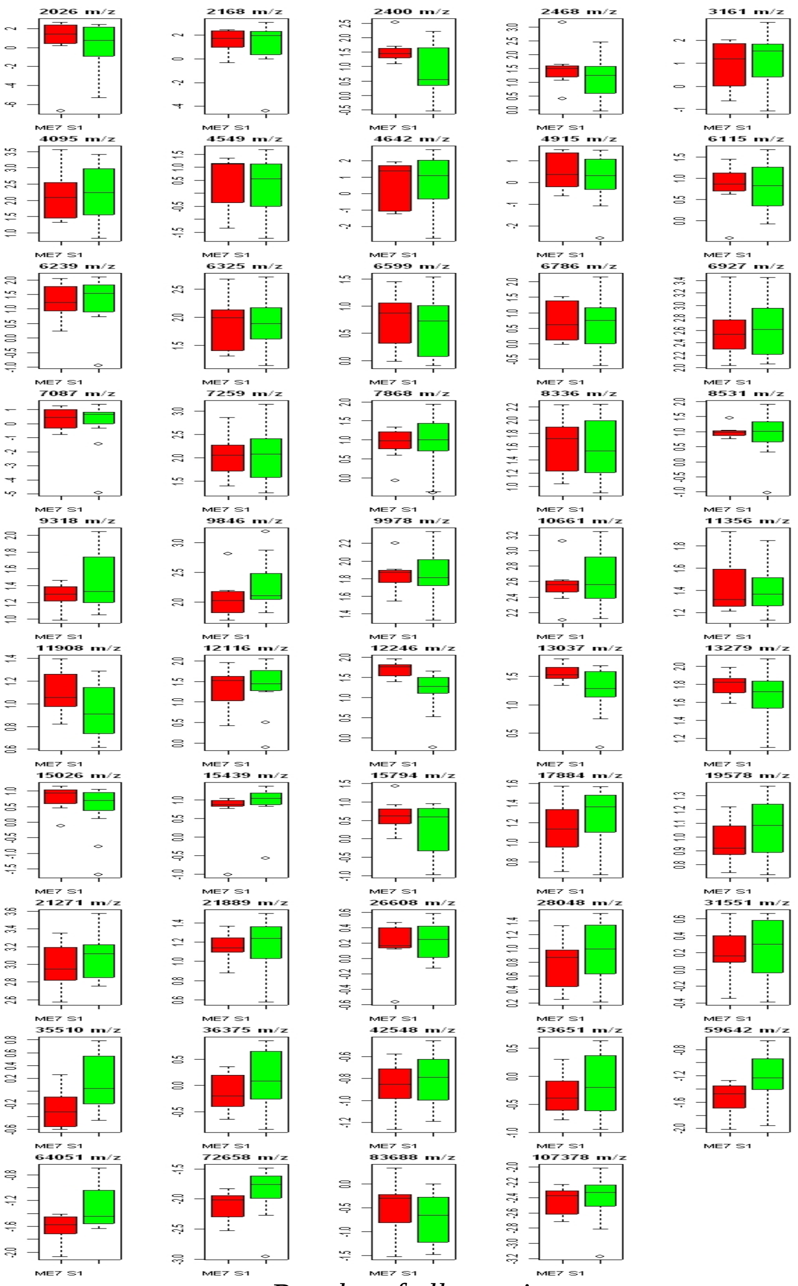

Boxplot of all proteins

Cluster Analysis of samples (Euclidean distance)

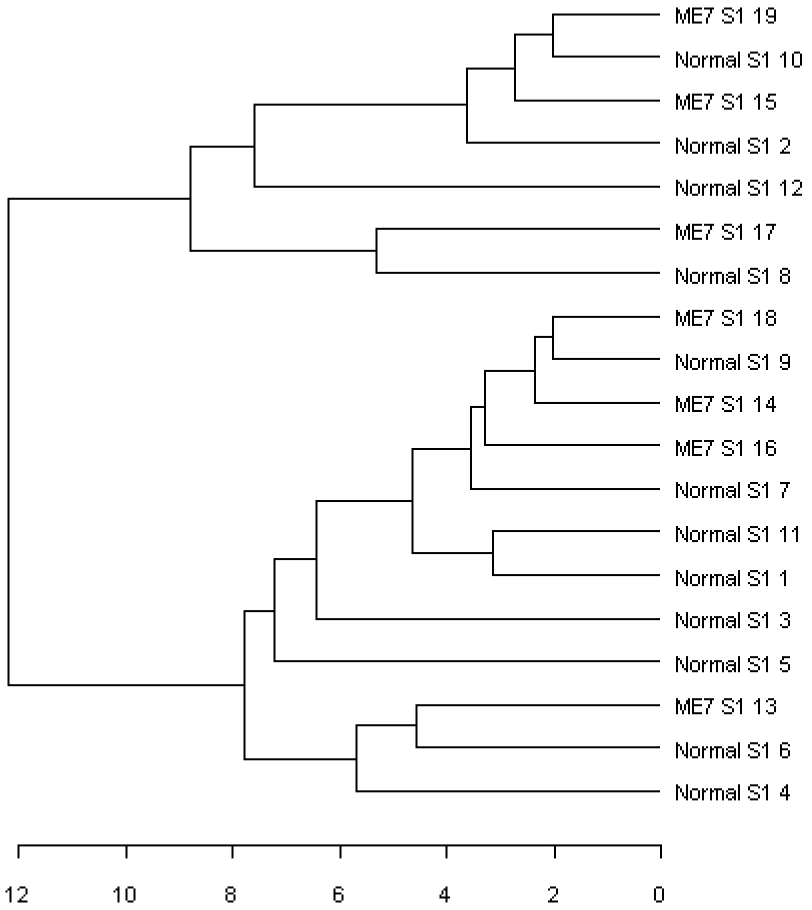

Cluster Analysis of samples (Euclidean distance)

S1 Q10 60

Proteins showing total separation

No proteins showed complete separation

Significant data (p<=0.05)

Significant Proteins (t-test; p<=0.05)

	name	mz	ME7.avg	NORM.avg	t	p
2	C03243_3	3243	2.4	2.1	2.5	0.030
9	C012225_	12225	1.2	1.5	-2.8	0.016

Data for Significant proteins

	C0GROUP	C0GRP_NA	C0Spectr	C03243_3	C012225_
9	0	ME7 S1	B33451	2.7	1.37
10	0	ME7 S1	B33452	2.1	1.29
11	0	ME7 S1	B33453	2.2	1.24
12	0	ME7 S1	B33454	2.4	0.93
13	0	ME7 S1	B33455	2.6	1.31
14	0	ME7 S1	B33456	2.5	1.21
1	1	Normal S1	B33445	2.0	1.48
2	1	Normal S1	B33446	1.9	1.64
3	1	Normal S1	B33447	2.1	1.77
4	1	Normal S1	B33447	2.4	1.25
5	1	Normal S1	B33448	2.4	1.34
6	1	Normal S1	B33449	2.1	1.72
7	1	Normal S1	B33449	2.3	1.64
8	1	Normal S1	B33450	2.0	1.21

Boxplot of significant proteins

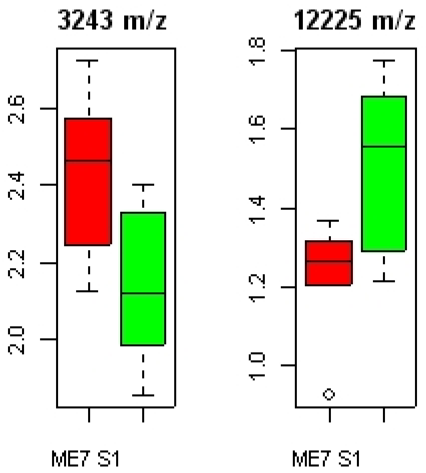

Boxplot of significant proteins

Pairwise Scatterplots of Significant Proteins

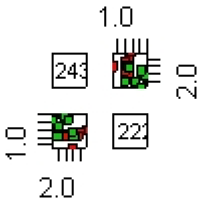

Pairwise Scatterplots of Significant Proteins

All data *Boxplot of all proteins*

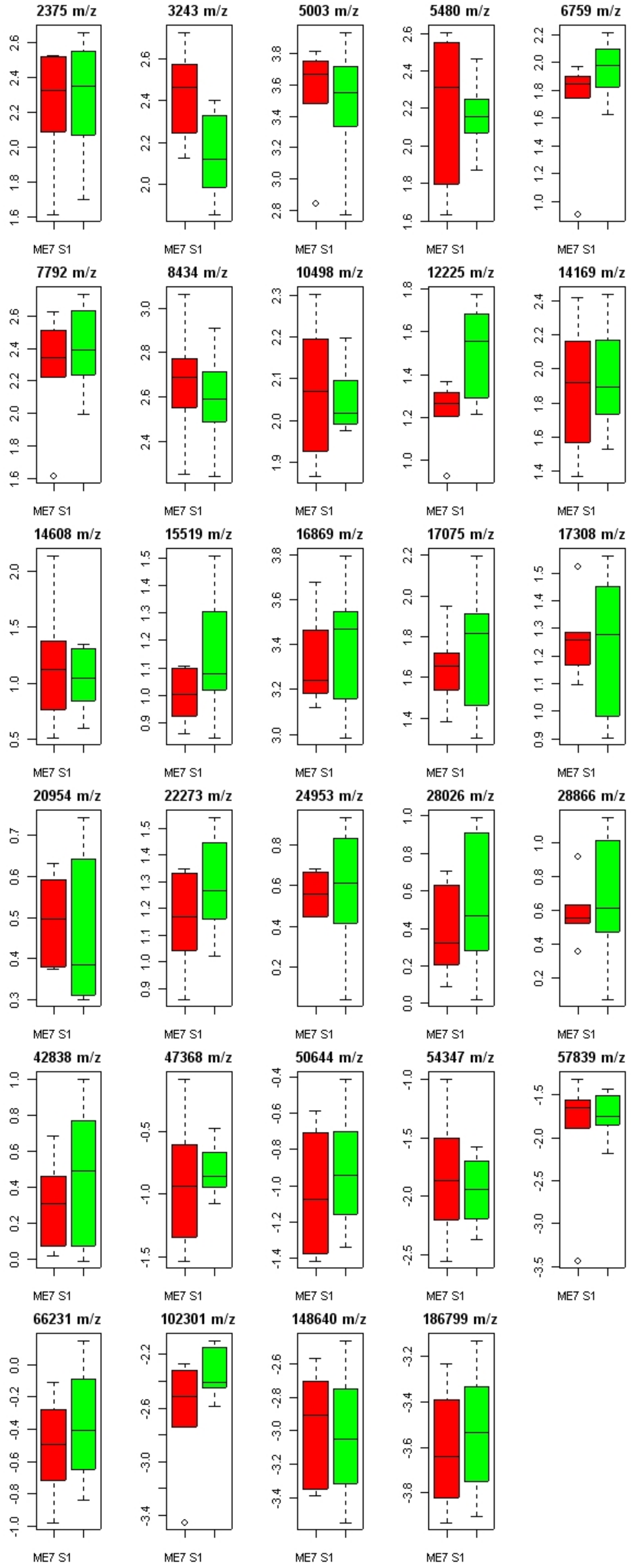

Boxplot of all proteins

Cluster Analysis of samples (Euclidean distance)

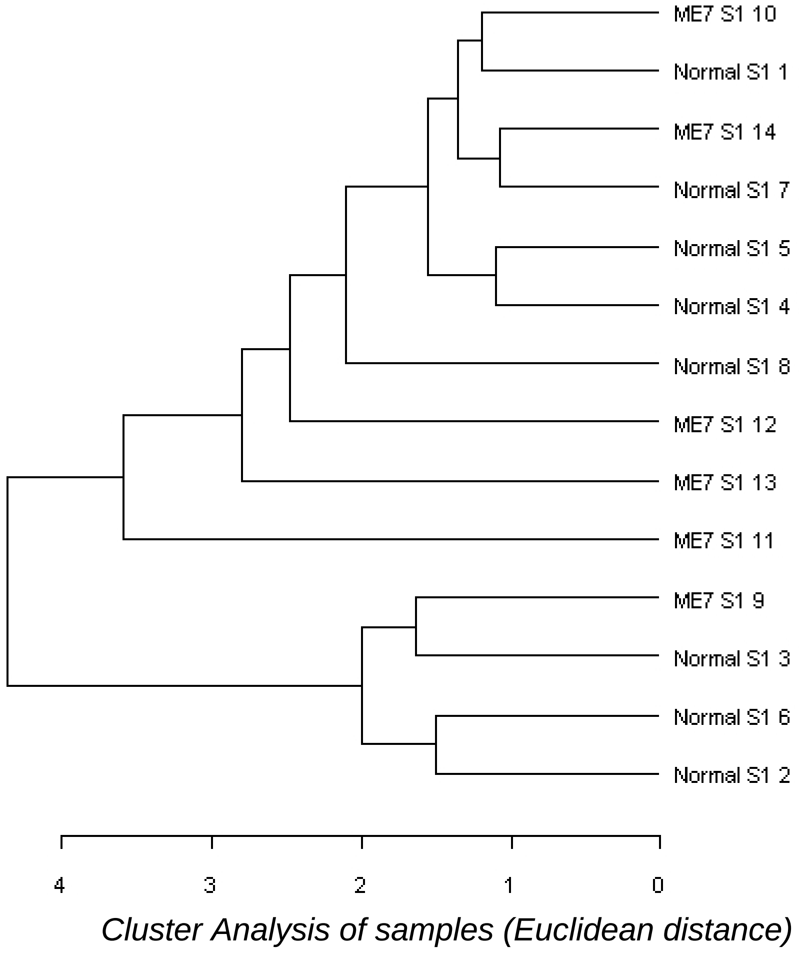

S1 Q10 90

Proteins showing total separation

	GROUP	GRP_NA	Spectr	5234	7670	8441	13642	14183	15262	16885	22292	28105	28898	57359	84779
9	0	ME7 S1	B36863	2.52	2.3	2.8	0.68	2.0	1.10	3.4	1.32	0.144	0.40	-1.9	-3.3
10	0	ME7 S1	B36863	2.52	2.3	2.8	0.68	2.0	1.10	3.4	1.32	0.144	0.40	-1.9	-3.3
11	0	ME7 S1	B36865	2.46	1.9	2.4	0.27	1.4	0.82	3.2	0.98	-0.024	0.17	-2.1	-3.2
12	0	ME7 S1	B36865	2.46	1.9	2.4	0.27	1.4	0.82	3.2	0.98	-0.024	0.17	-2.1	-3.2
13	0	ME7 S1	B36867	2.93	2.4	2.7	0.48	1.9	1.13	3.2	1.11	-0.274	0.16	-2.0	-3.2
14	0	ME7 S1	B36867	2.93	2.4	2.7	0.48	1.9	1.13	3.2	1.11	-0.274	0.16	-2.0	-3.2
1	1	Normal S1	B36857	1.06	3.2	3.0	1.45	2.8	1.65	3.6	2.01	0.496	0.67	-1.8	-3.0
2	1	Normal S1	B36857	1.06	3.2	3.0	1.45	2.8	1.65	3.6	2.01	0.496	0.67	-1.8	-3.0
3	1	Normal S1	B36858	1.22	2.9	2.9	1.33	2.3	1.70	3.4	1.74	0.427	0.60	-1.8	-3.1
4	1	Normal S1	B36858	1.22	2.9	2.9	1.33	2.3	1.70	3.4	1.74	0.427	0.60	-1.8	-3.1
5	1	Normal S1	B36859	0.86	2.9	2.9	1.33	2.4	1.57	3.5	1.72	0.603	0.68	-1.6	-2.8
6	1	Normal S1	B36859	0.86	2.9	2.9	1.33	2.4	1.57	3.5	1.72	0.603	0.68	-1.6	-2.8
7	1	Normal S1	B36860	1.93	2.7	2.9	1.28	2.5	1.68	3.6	1.51	0.629	0.84	-1.7	-2.8
8	1	Normal S1	B36860	1.93	2.7	2.9	1.28	2.5	1.68	3.6	1.51	0.629	0.84	-1.7	-2.8

Boxplot of proteins showing complete separation

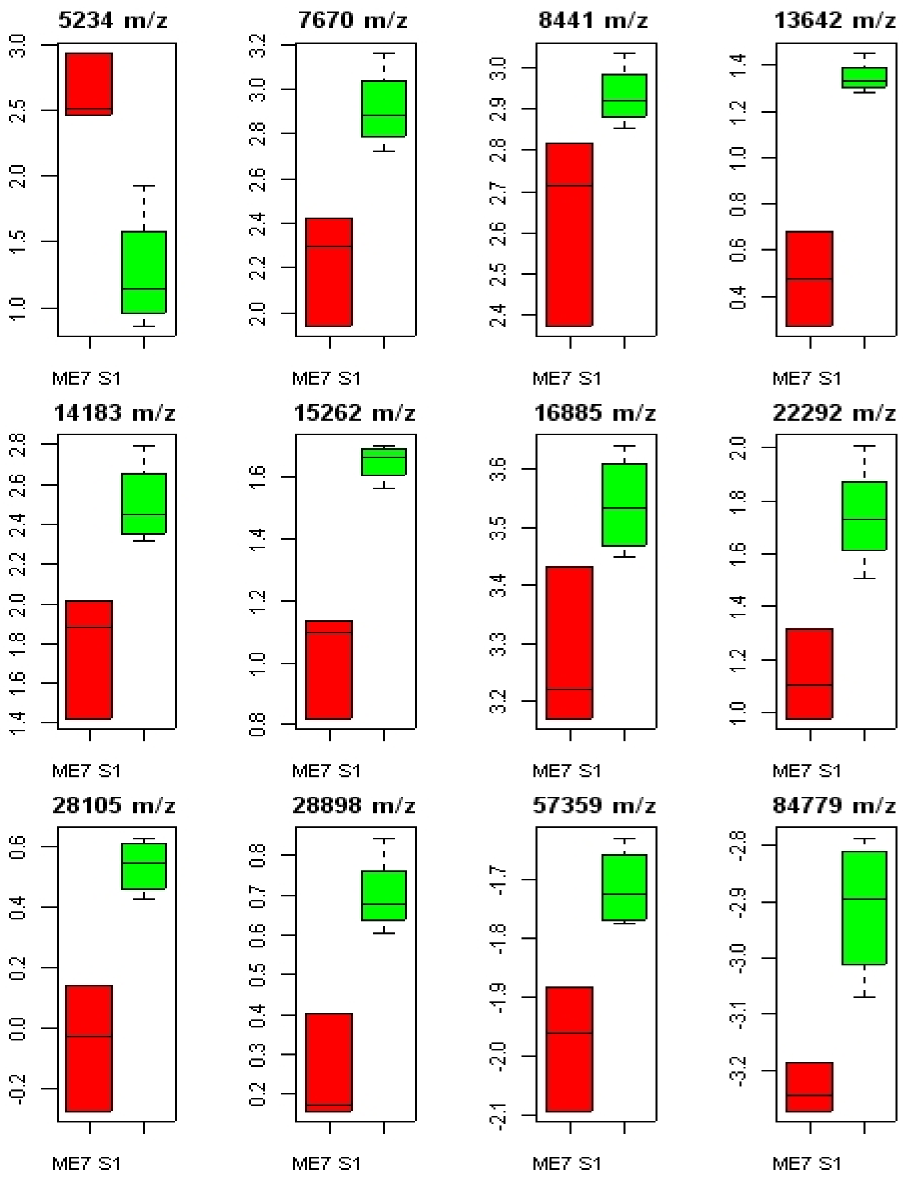

Pairwise Scatterplots of Proteins showing complete separation

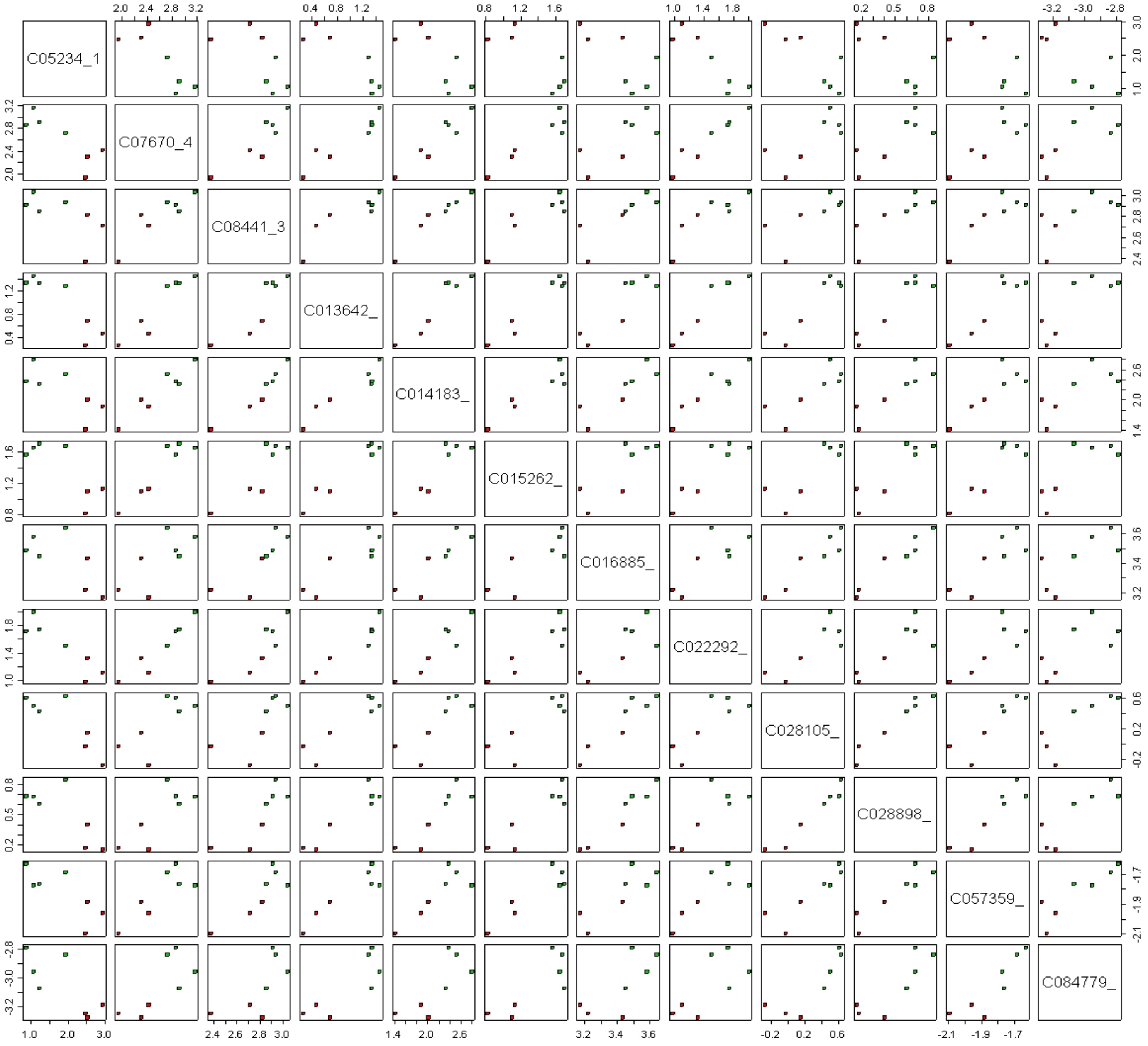

Significant data (p<=0.01)

Significant Proteins (t-test; p<=0.01)

	name	mz	ME7.avg	NORM.avg	t	p
5	C05234_1	5234	2.636	1.27	7.7	9.0e-06
9	C07670_4	7670	2.220	2.91	-6.3	1.3e-04
17	C013642_	13642	0.477	1.35	-11.0	3.4e-05
18	C014183_	14183	1.770	2.50	-5.5	4.4e-04
19	C014620_	14620	0.965	1.60	-4.3	1.4e-03
21	C015262_	15262	1.017	1.65	-9.6	7.7e-05
22	C016885_	16885	3.274	3.54	-4.5	1.8e-03
23	C017090_	17090	1.645	1.91	-3.9	3.9e-03
28	C022292_	22292	1.134	1.74	-6.7	2.5e-05
30	C028105_	28105	-0.051	0.54	-7.1	2.5e-04
31	C028898_	28898	0.243	0.70	-7.6	3.1e-05
36	C057359_	57359	-1.979	-1.71	-5.9	3.2e-04
38	C084779_	84779	-3.235	-2.91	-7.3	4.8e-05

Data for Significant proteins

	C0GROUP	GRP_NA	Spectr	5234_1	7670_4	13642_	14183_	14620_	15262_	16885_	17090_	22292_	28105_	28898_	57359_	84779_
9	0	ME7 S1	B36863	2.52	2.3	0.68	2.0	1.12	1.10	3.4	1.8	1.32	0.144	0.40	-1.9	-3.3
10	0	ME7 S1	B36863	2.52	2.3	0.68	2.0	1.12	1.10	3.4	1.8	1.32	0.144	0.40	-1.9	-3.3
11	0	ME7 S1	B36865	2.46	1.9	0.27	1.4	1.03	0.82	3.2	1.6	0.98	-0.024	0.17	-2.1	-3.2
12	0	ME7 S1	B36865	2.46	1.9	0.27	1.4	1.03	0.82	3.2	1.6	0.98	-0.024	0.17	-2.1	-3.2
13	0	ME7 S1	B36867	2.93	2.4	0.48	1.9	0.75	1.13	3.2	1.5	1.11	-0.274	0.16	-2.0	-3.2
14	0	ME7 S1	B36867	2.93	2.4	0.48	1.9	0.75	1.13	3.2	1.5	1.11	-0.274	0.16	-2.0	-3.2
1	1	Normal S1	B36857	1.06	3.2	1.45	2.8	2.07	1.65	3.6	2.0	2.01	0.496	0.67	-1.8	-3.0
2	1	Normal S1	B36857	1.06	3.2	1.45	2.8	2.07	1.65	3.6	2.0	2.01	0.496	0.67	-1.8	-3.0
3	1	Normal S1	B36858	1.22	2.9	1.33	2.3	1.51	1.70	3.4	1.8	1.74	0.427	0.60	-1.8	-3.1
4	1	Normal S1	B36858	1.22	2.9	1.33	2.3	1.51	1.70	3.4	1.8	1.74	0.427	0.60	-1.8	-3.1
5	1	Normal S1	B36859	0.86	2.9	1.33	2.4	1.12	1.57	3.5	1.8	1.72	0.603	0.68	-1.6	-2.8
6	1	Normal S1	B36859	0.86	2.9	1.33	2.4	1.12	1.57	3.5	1.8	1.72	0.603	0.68	-1.6	-2.8
7	1	Normal S1	B36860	1.93	2.7	1.28	2.5	1.71	1.68	3.6	2.0	1.51	0.629	0.84	-1.7	-2.8
8	1	Normal S1	B36860	1.93	2.7	1.28	2.5	1.71	1.68	3.6	2.0	1.51	0.629	0.84	-1.7	-2.8

Boxplot of significant proteins

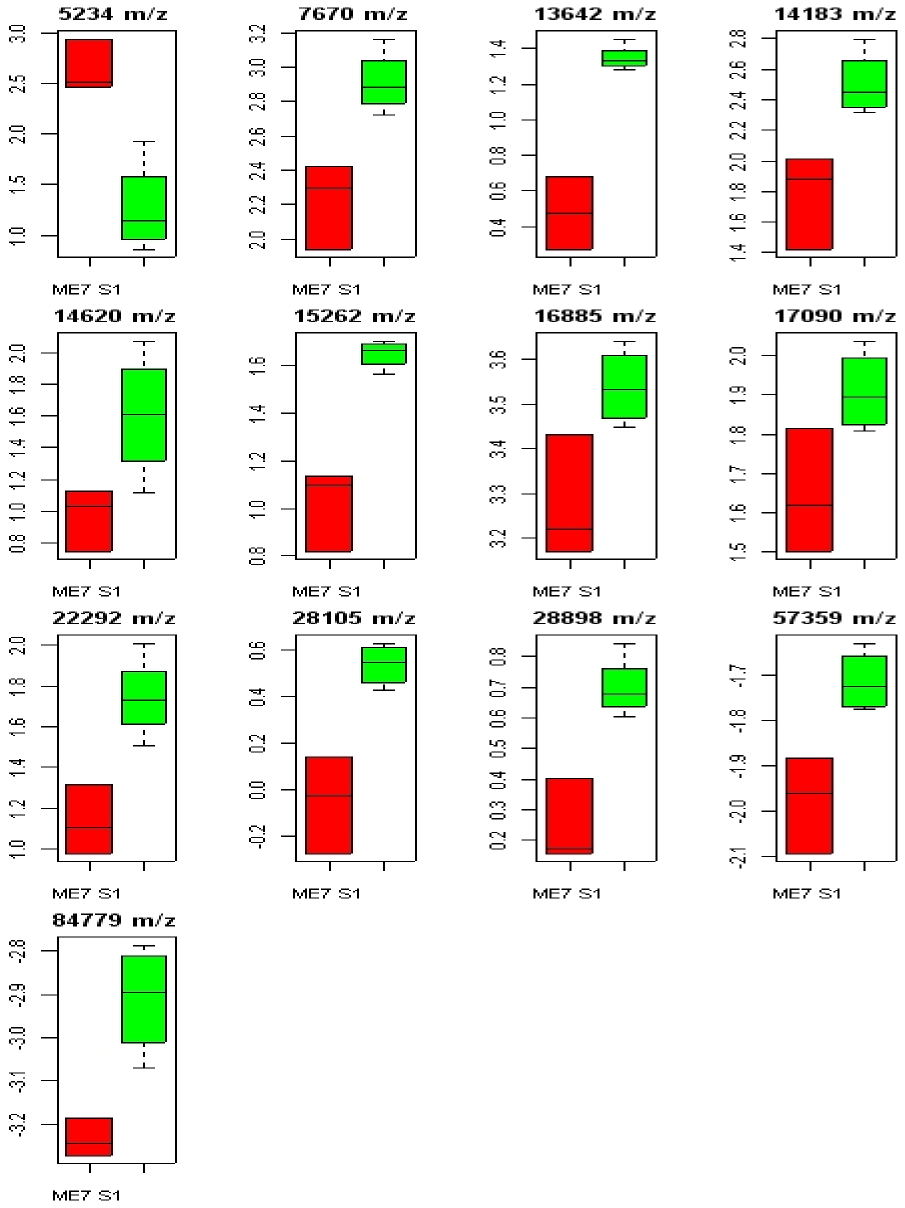

Boxplot of significant proteins

Pairwise Scatterplots of Significant Proteins

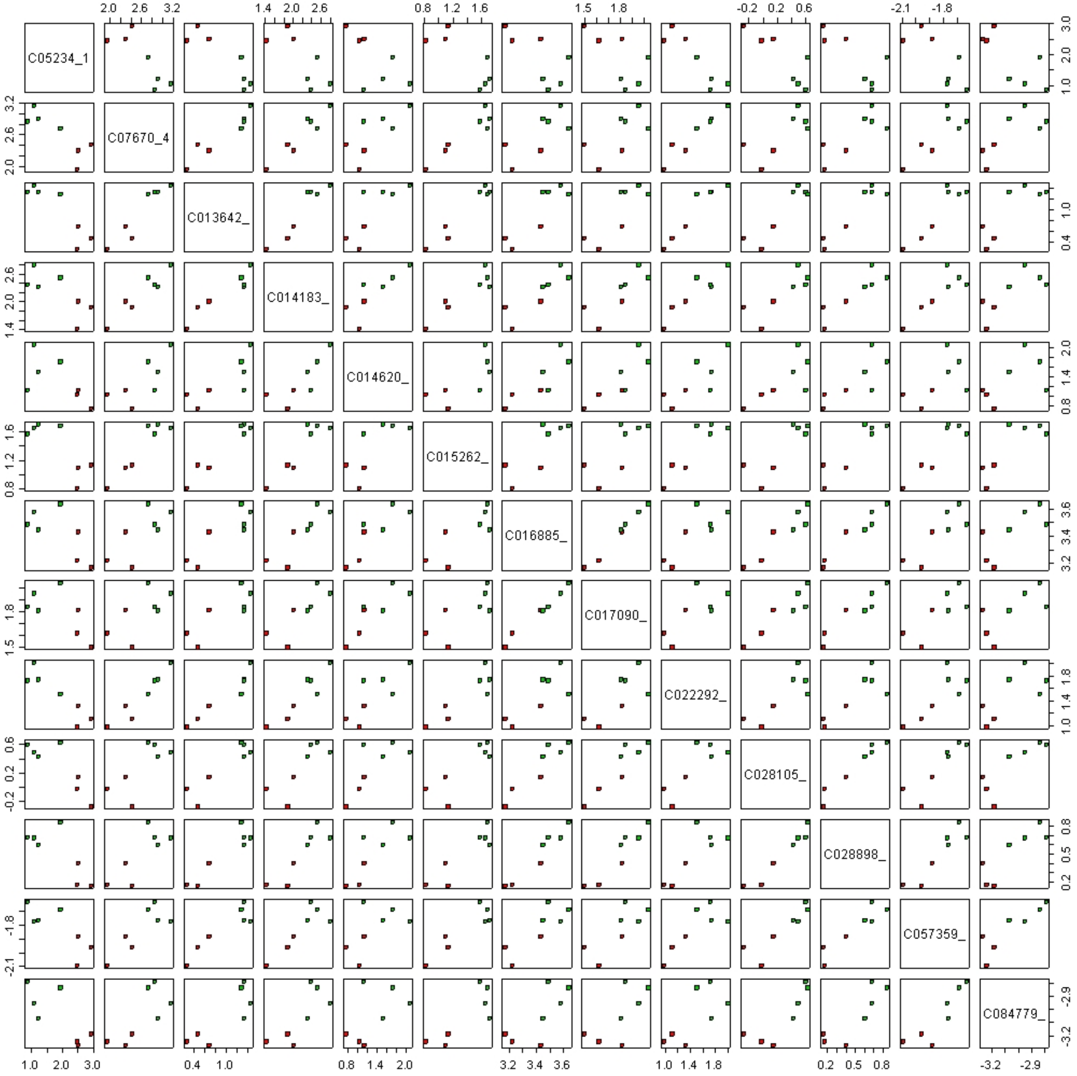

Pairwise Scatterplots of Significant Proteins

Cluster Analysis of samples (Euclidean distance)

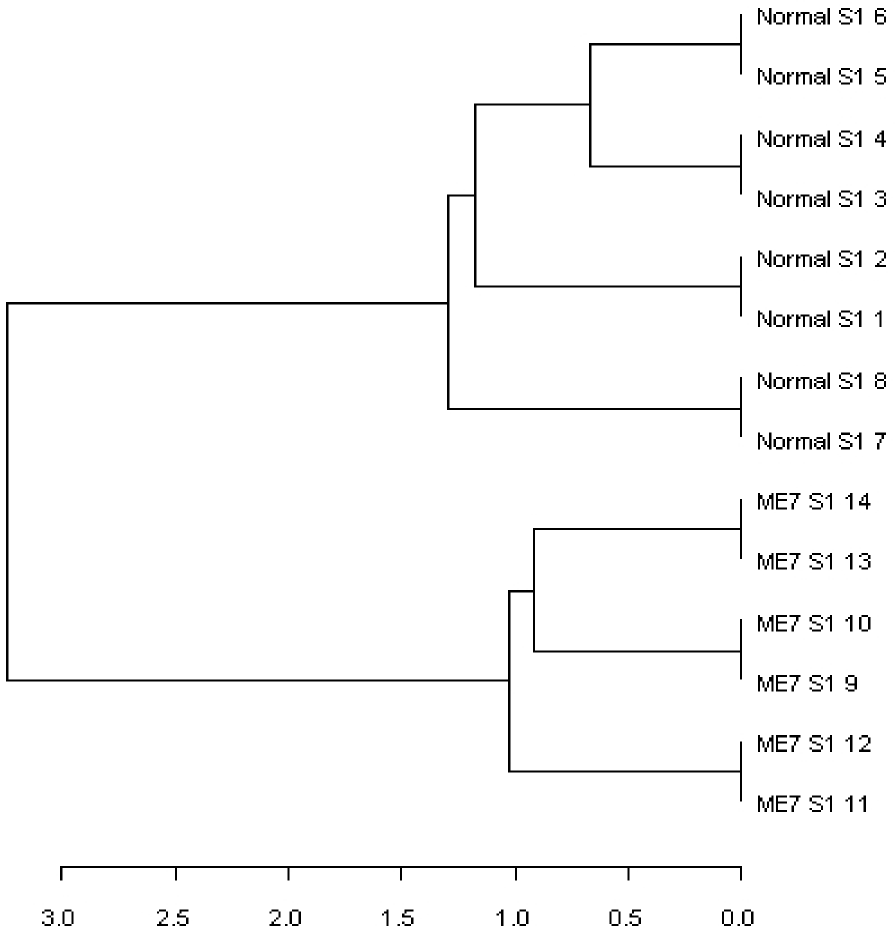

Cluster Analysis of samples (Euclidean distance)

Plot of first three principal components

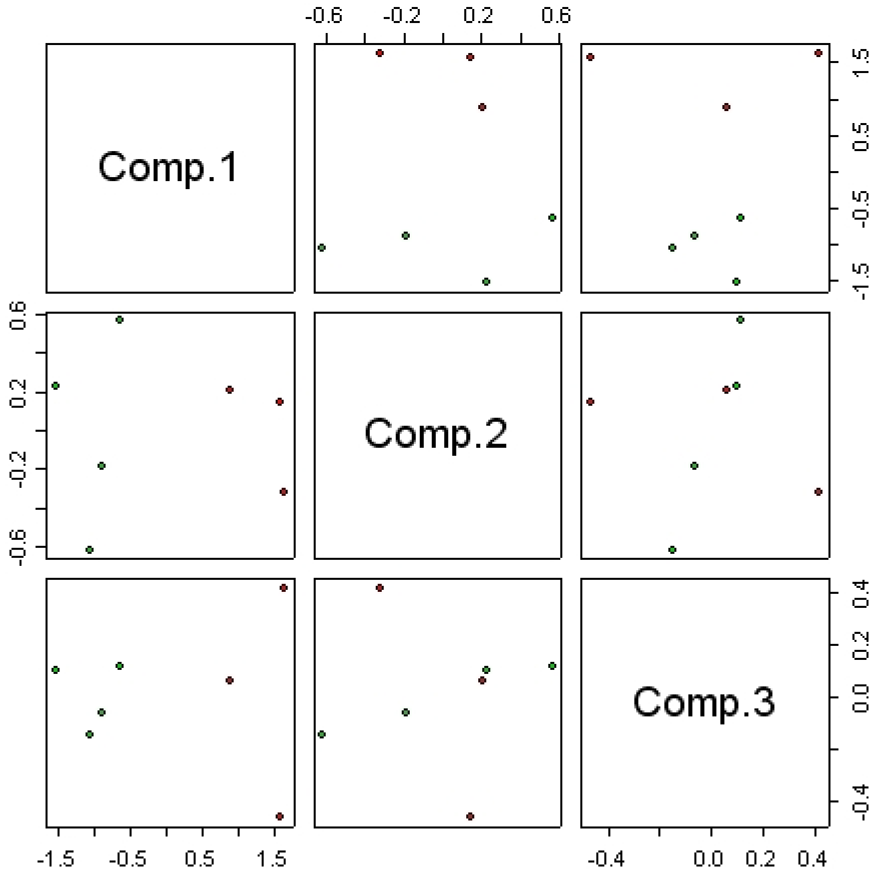

Plot of first three principal components

Scatterplot of linear discriminant function (x-axis)

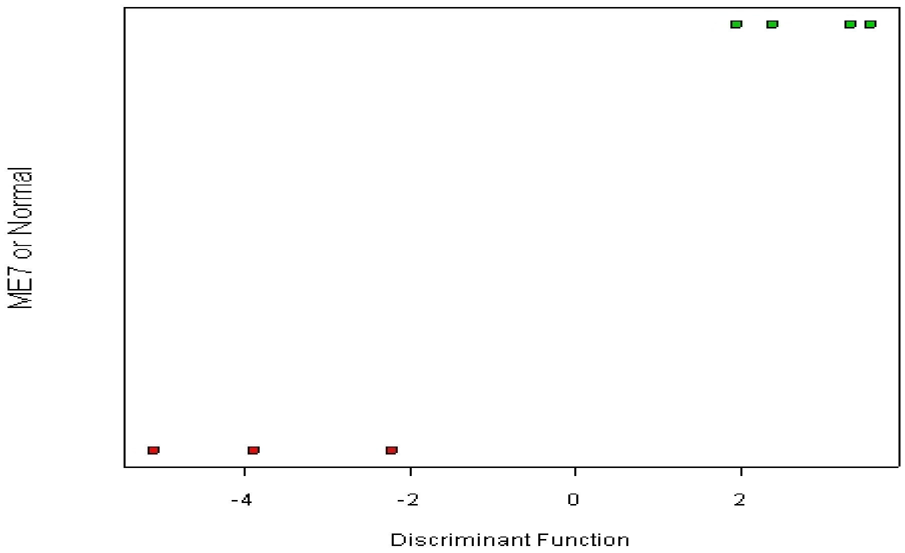

Scatterplot of linear discriminant function (x-axis)

All data

Boxplot of all proteins

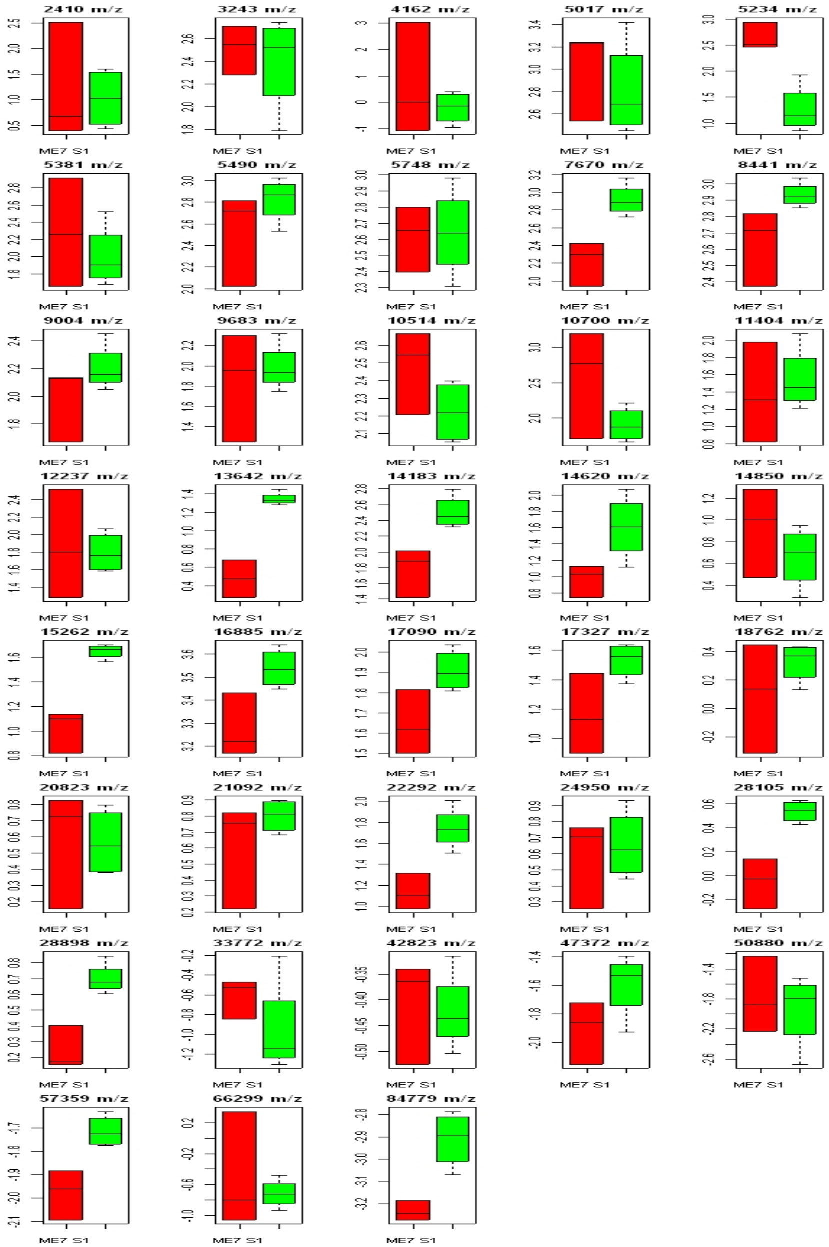

Boxplot of all proteins

Cluster Analysis of samples (Euclidean distance)

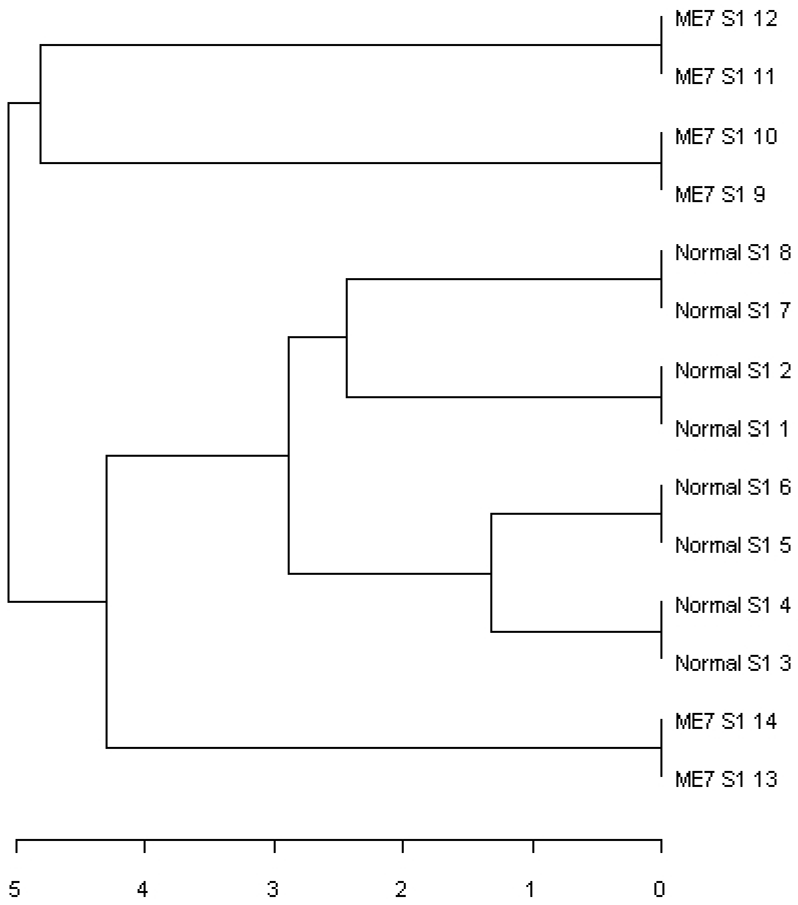

Cluster Analysis of samples (Euclidean distance)

S1 Q10 120

S1 Q10 120

Proteins showing total separation

	C0GROUP	C0GRP_NA	Spectr	5234	6054	8445	10085	10483	10707	13638	14189	14863	15460	16889	17084	17327	22295	26207	28226
1	0	ME7 S1	B36885	2.99	1.8	2.5	2.07	2.8	3.3	0.80	1.7	1.15	0.94	3.1	1.5	0.97	1.1	-0.51	0.095
3	0	ME7 S1	B37003	3.68	1.5	2.9	2.47	2.9	3.3	0.87	1.8	1.22	0.94	3.1	1.6	1.07	1.1	-0.55	0.161
4	0	ME7 S1	B37004	3.29	-3.9	2.5	2.73	2.9	3.8	1.05	2.2	1.32	1.41	3.1	1.4	1.13	1.1	-0.62	-0.148
5	0	ME7 S1	B37020	3.48	1.7	2.7	2.82	3.1	3.3	0.80	1.7	1.65	1.14	3.2	1.6	1.10	1.1	-0.97	-0.167
7	0	ME7 S1	B37134	3.02	1.5	2.9	2.80	3.0	3.4	0.77	1.8	1.32	1.08	3.2	1.6	0.93	1.1	-0.90	-0.215
8	0	ME7 S1	B37283	3.68	1.8	2.4	2.67	3.1	3.6	0.94	1.9	1.47	0.32	3.2	1.5	1.30	1.4	-0.47	-0.020
10	0	ME7 S1	B37285	3.22	1.6	2.8	2.27	3.1	3.5	1.20	2.0	1.40	0.48	3.4	1.9	1.41	1.3	-0.17	0.438
2	1	Normal S1	B37002	0.61	2.7	3.3	1.15	2.3	2.4	1.77	3.0	1.04	1.87	3.9	2.3	1.85	2.0	0.26	0.855
6	1	Normal S1	B37133	1.38	2.6	3.0	1.04	2.1	2.3	1.87	2.6	0.64	1.62	3.6	2.0	1.79	2.1	0.33	0.617
9	1	Normal S1	B37284	1.86	2.6	3.1	1.20	2.1	2.5	1.84	2.6	0.23	1.95	3.6	2.0	1.63	2.0	0.15	0.731
11	1	Normal S1	B37329	0.86	2.9	3.4	0.81	2.0	2.2	1.93	2.6	0.62	1.87	3.8	2.1	1.70	2.1	0.19	0.716
12	1	Normal S1	B37499	1.53	2.7	3.3	0.75	2.3	2.3	1.85	2.6	1.07	1.96	3.8	2.2	1.68	1.8	0.31	0.665
13	1	Normal S1	B37500	0.90	2.6	3.2	0.70	2.0	2.4	2.00	2.7	0.60	2.06	3.7	2.1	1.72	2.1	0.48	0.934
14	1	Normal S1	B37501	1.30	2.7	3.0	0.48	2.2	2.1	1.49	2.6	0.76	1.68	3.7	2.3	1.83	1.5	0.31	0.927

S1 Q10 120

Boxplot of proteins showing complete separation

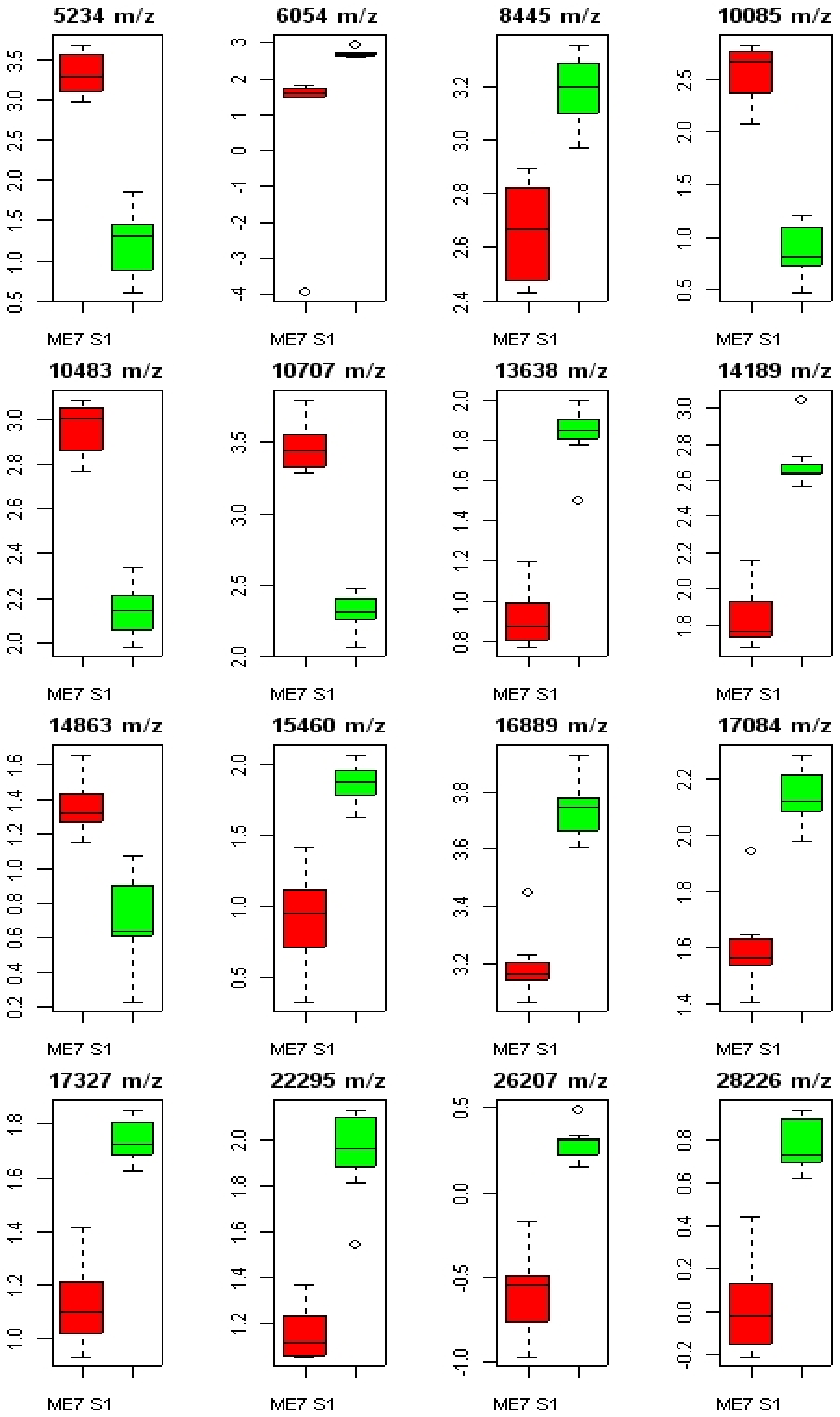

S1 Q10 120

Pairwise Scatterplots of Proteins showing complete separation

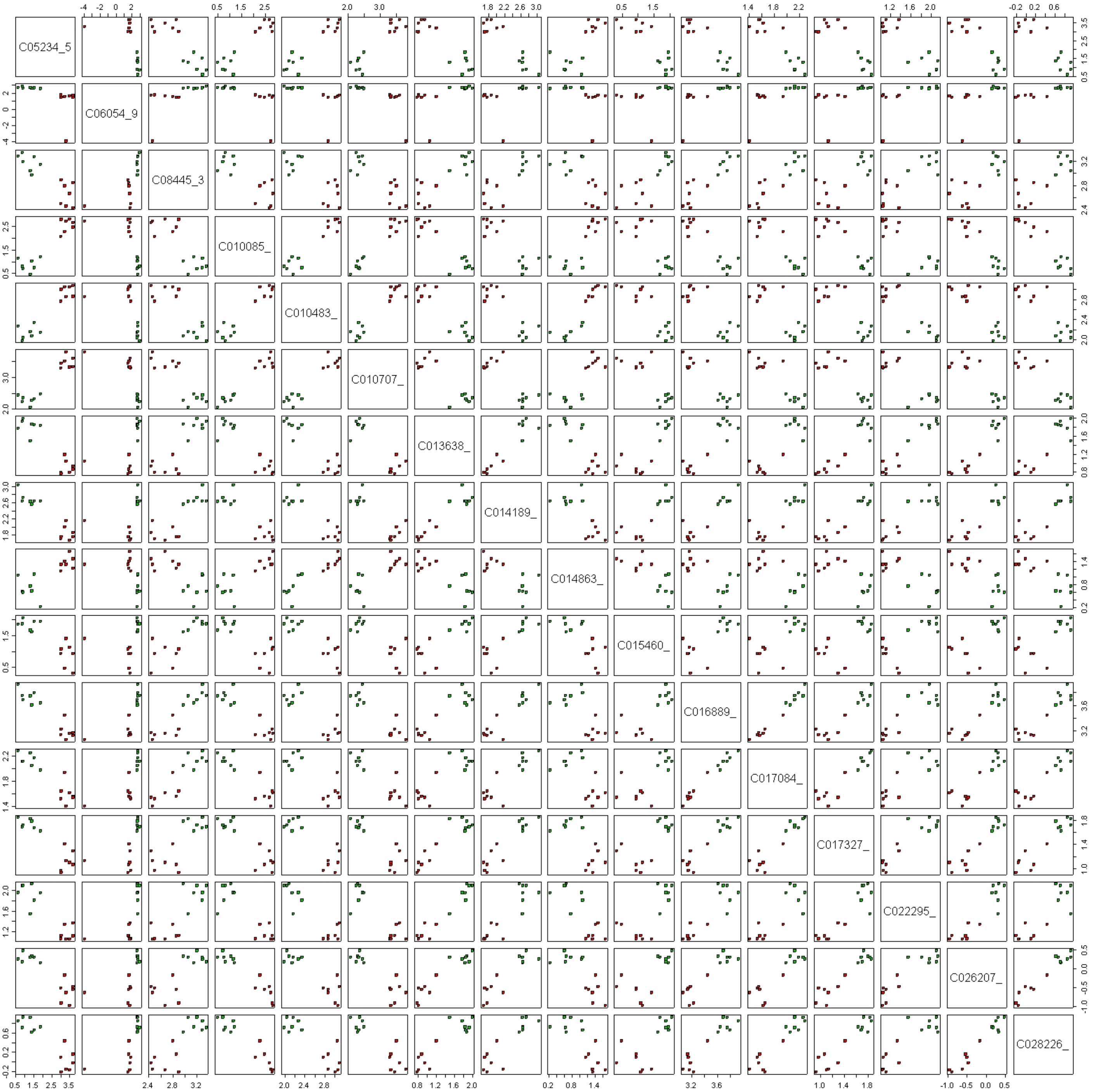

S1 Q10 120

Significant data (p<=1e-04)

Significant Proteins (t-test; p<=1e-04)

	name	mz	ME7.avg	NORM.avg	t	p
3	C05234_5	5234	3.336	1.21	10.8	5.6e-07
9	C07800_9	7800	2.529	3.17	-6.2	9.0e-05
14	C010085_	10085	2.548	0.88	11.4	9.2e-08
16	C010483_	10483	2.956	2.14	12.1	4.3e-08
18	C010707_	10707	3.466	2.31	13.4	3.2e-08
24	C013638_	13638	0.918	1.82	-10.7	1.7e-07
25	C014189_	14189	1.846	2.70	-9.5	6.6e-07
30	C016889_	16889	3.195	3.74	-8.9	1.4e-06
31	C017084_	17084	1.607	2.14	-7.1	2.7e-05
32	C017327_	17327	1.130	1.74	-8.5	1.8e-05
36	C022295_	22295	1.158	1.94	-8.3	6.5e-06
38	C026207_	26207	-0.597	0.29	-8.0	4.8e-05
39	C028226_	28226	0.020	0.78	-7.6	2.7e-05

Data for Significant proteins

	GROUP	GRP_NA	Spectr	5234	7800	10085	10483	10707	13638	14189	16889	17084	17327	22295	26207	28226
1	0	ME7 S1	B36885	2.99	2.5	2.07	2.8	3.3	0.80	1.7	3.1	1.5	0.97	1.1	-0.51	0.095
3	0	ME7 S1	B37003	3.68	2.4	2.47	2.9	3.3	0.87	1.8	3.1	1.6	1.07	1.1	-0.55	0.161
4	0	ME7 S1	B37004	3.29	2.9	2.73	2.9	3.8	1.05	2.2	3.1	1.4	1.13	1.1	-0.62	-0.148
5	0	ME7 S1	B37020	3.48	2.2	2.82	3.1	3.3	0.80	1.7	3.2	1.6	1.10	1.1	-0.97	-0.167
7	0	ME7 S1	B37134	3.02	2.4	2.80	3.0	3.4	0.77	1.8	3.2	1.6	0.93	1.1	-0.90	-0.215
8	0	ME7 S1	B37283	3.68	2.6	2.67	3.1	3.6	0.94	1.9	3.2	1.5	1.30	1.4	-0.47	-0.020
10	0	ME7 S1	B37285	3.22	2.6	2.27	3.1	3.5	1.20	2.0	3.4	1.9	1.41	1.3	-0.17	0.438
2	1	Normal S1	B37002	0.61	3.2	1.15	2.3	2.4	1.77	3.0	3.9	2.3	1.85	2.0	0.26	0.855
6	1	Normal S1	B37133	1.38	3.2	1.04	2.1	2.3	1.87	2.6	3.6	2.0	1.79	2.1	0.33	0.617
9	1	Normal S1	B37284	1.86	3.1	1.20	2.1	2.5	1.84	2.6	3.6	2.0	1.63	2.0	0.15	0.731
11	1	Normal S1	B37329	0.86	3.3	0.81	2.0	2.2	1.93	2.6	3.8	2.1	1.70	2.1	0.19	0.716
12	1	Normal S1	B37499	1.53	3.2	0.75	2.3	2.3	1.85	2.6	3.8	2.2	1.68	1.8	0.31	0.665
13	1	Normal S1	B37500	0.90	3.4	0.70	2.0	2.4	2.00	2.7	3.7	2.1	1.72	2.1	0.48	0.934
14	1	Normal S1	B37501	1.30	2.9	0.48	2.2	2.1	1.49	2.6	3.7	2.3	1.83	1.5	0.31	0.927

S1 Q10 120

Boxplot of significant proteins

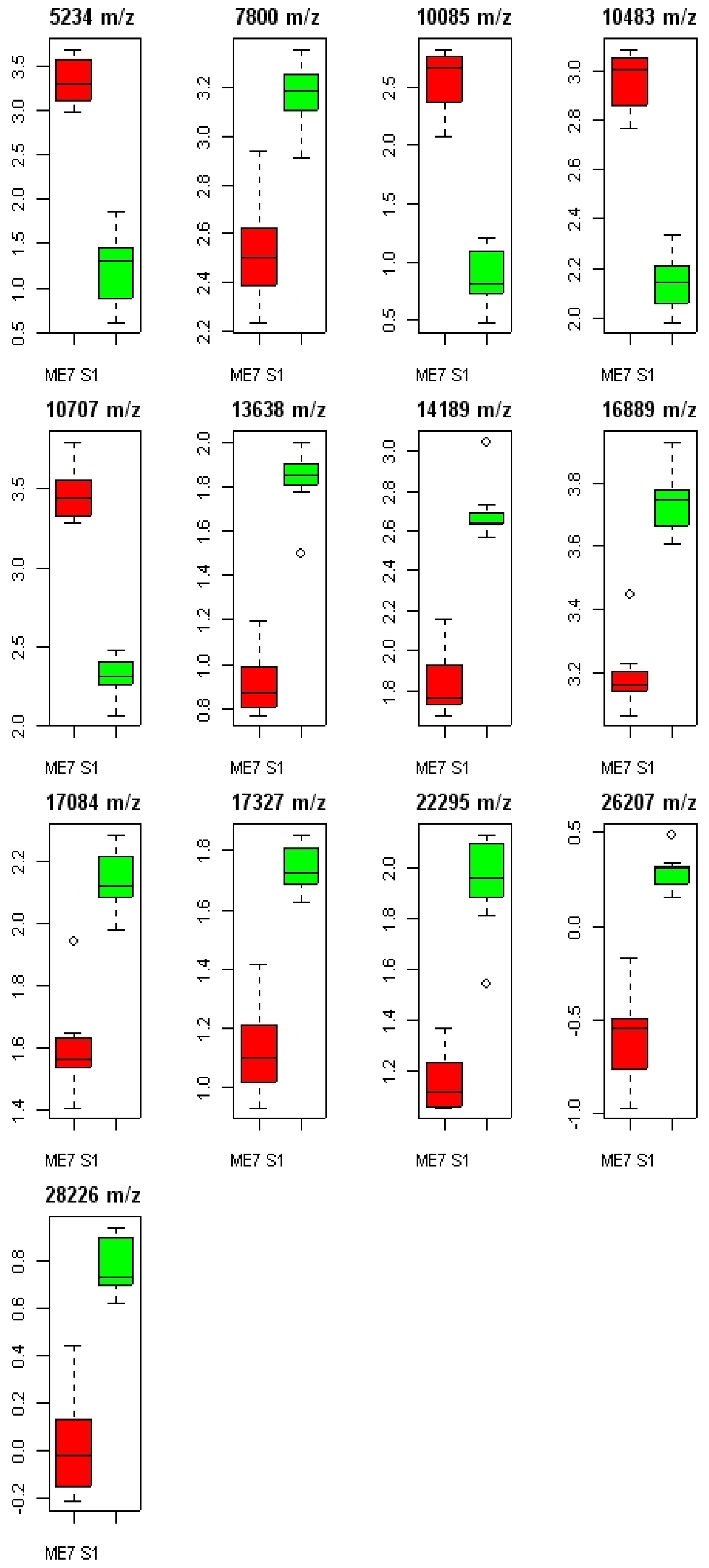

Boxplot of significant proteins

S1 Q10 120

Pairwise Scatterplots of Significant Proteins

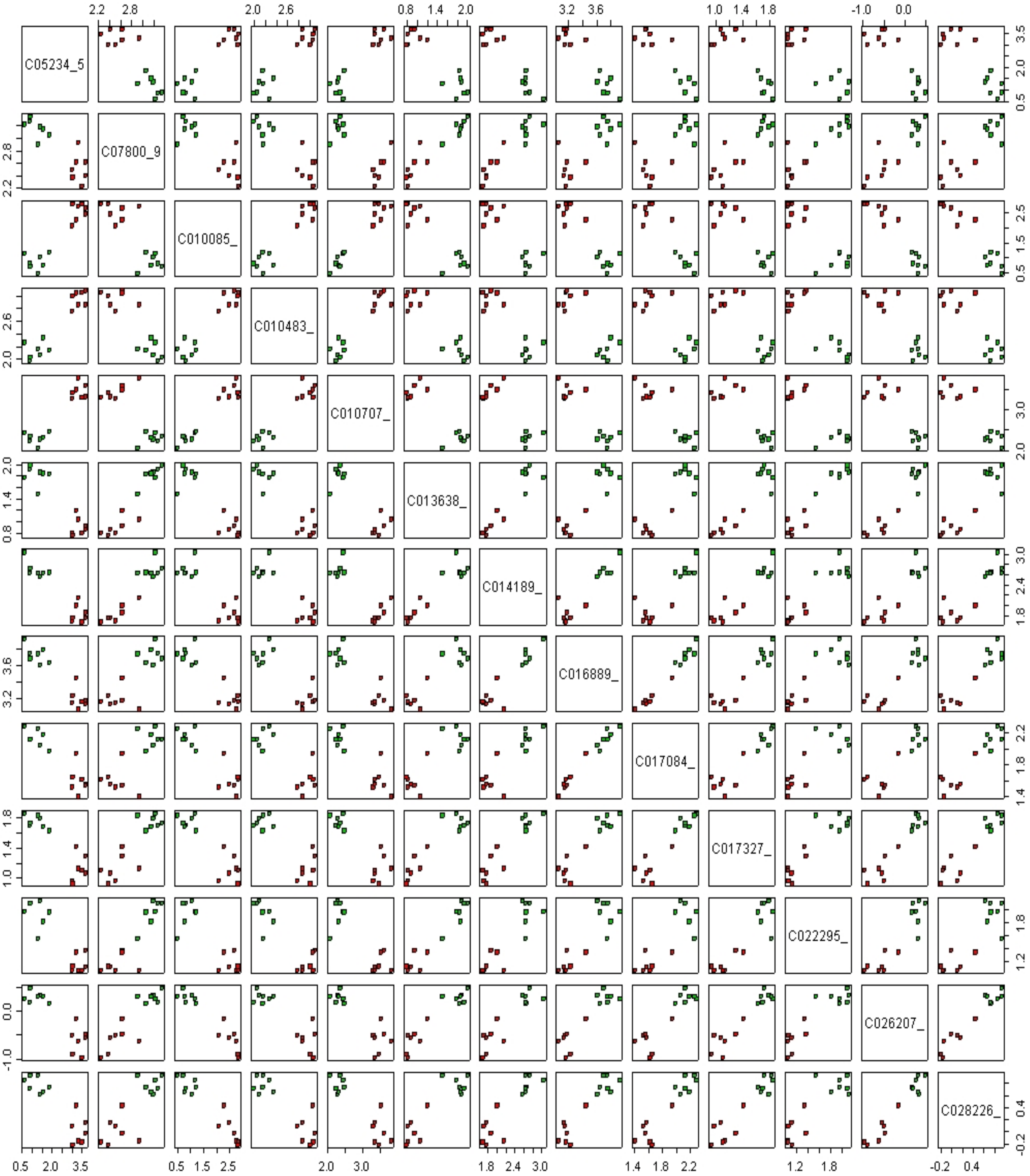

Pairwise Scatterplots of Significant Proteins

S1 Q10 120

Cluster Analysis of samples (Euclidean distance)

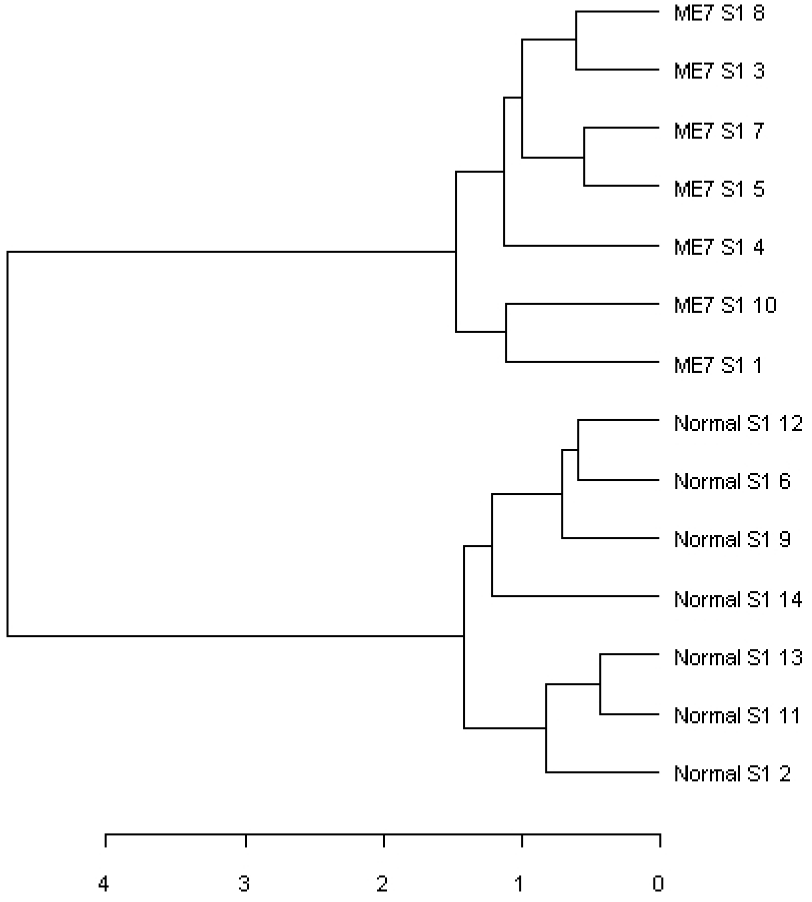

Cluster Analysis of samples (Euclidean distance)

S1 Q10 120

Plot of first three principal components

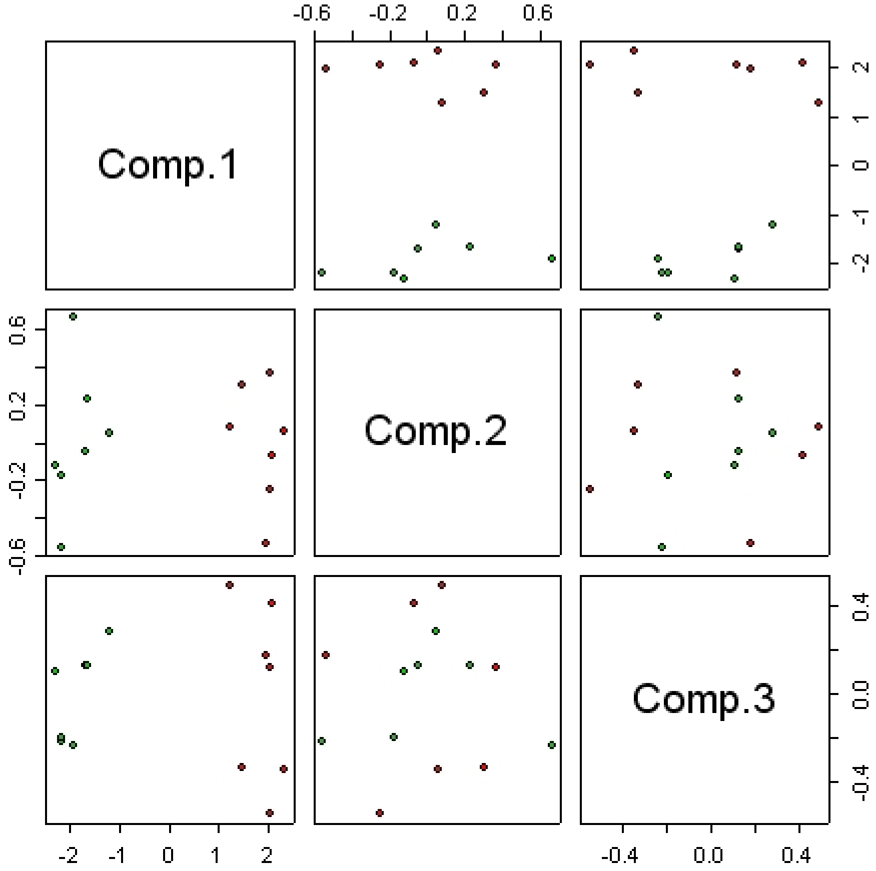

Plot of first three principal components

S1 Q10 120

Scatterplot of linear discriminant function (x-axis)

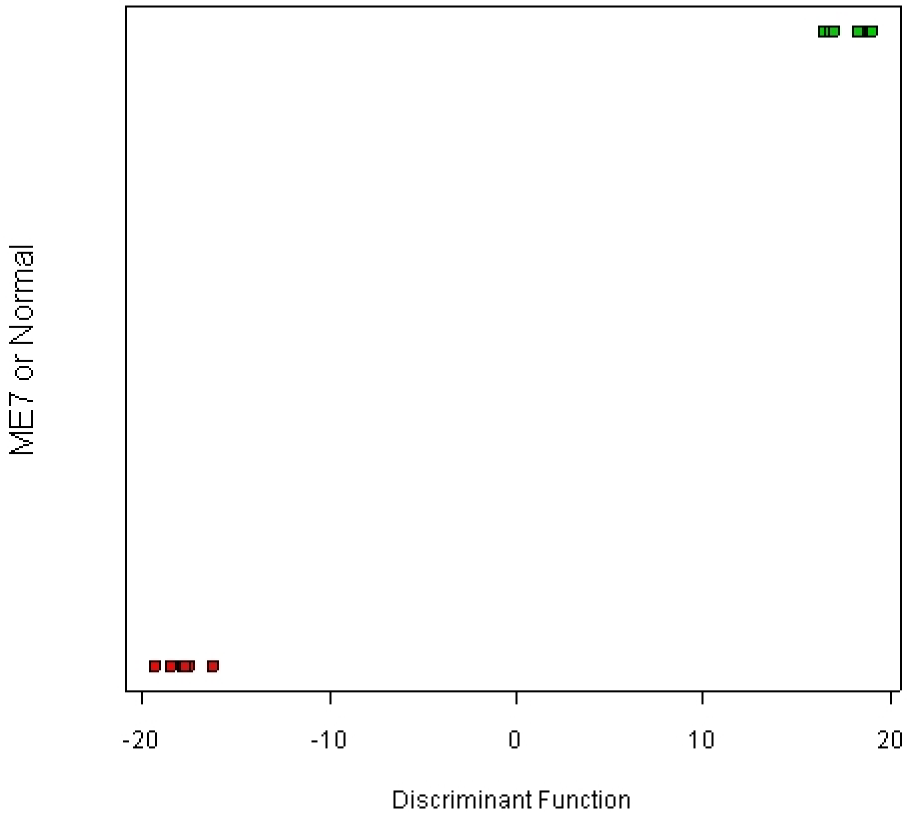

Scatterplot of linear discriminant function (x-axis)

S1 Q10 120

All data

Boxplot of all proteins

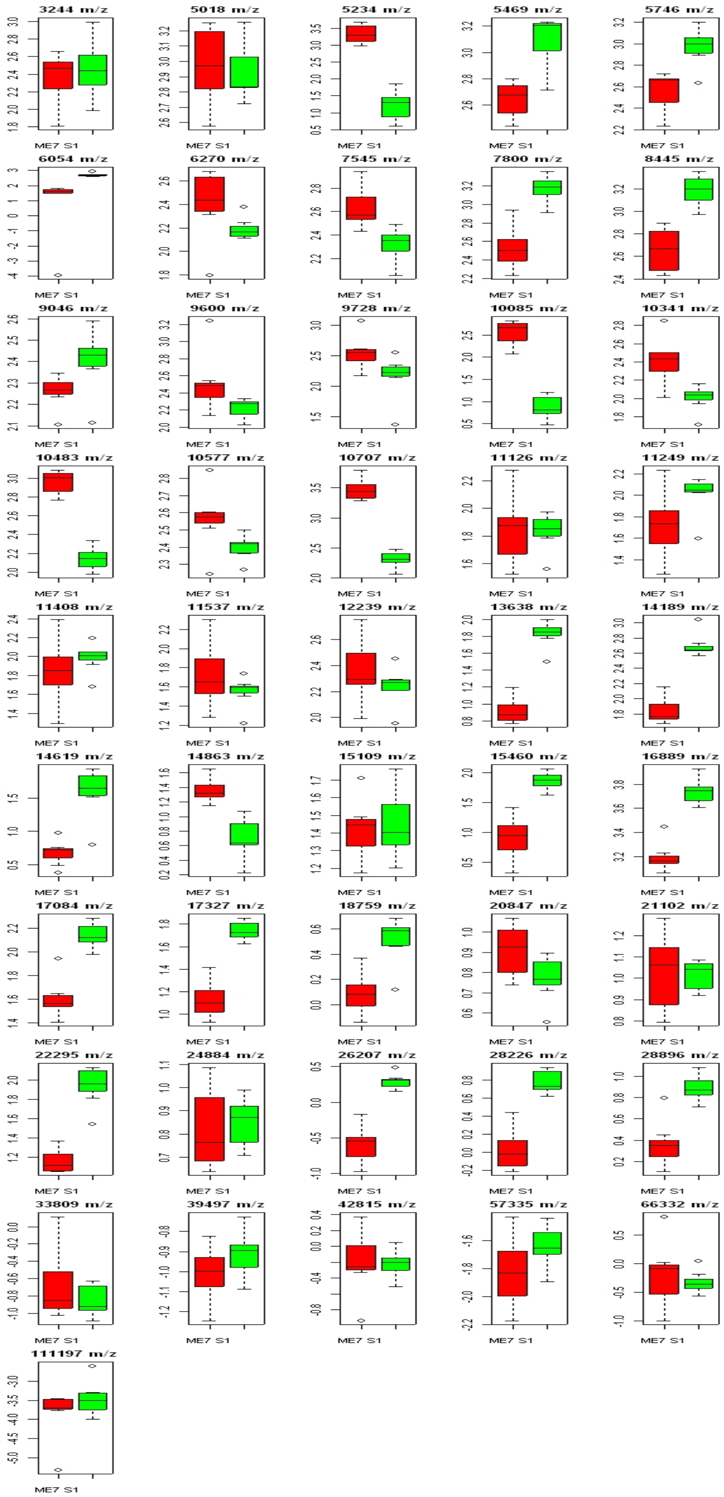

Boxplot of all proteins

S1 Q10 120

Cluster Analysis of samples (Euclidean distance)

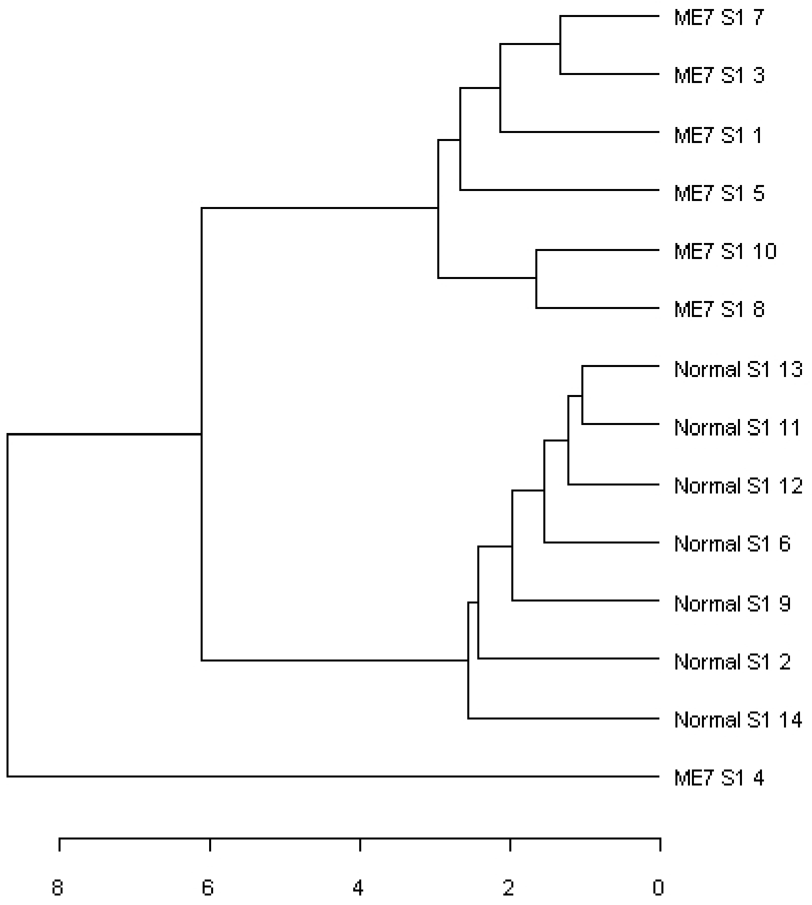

Cluster Analysis of samples (Euclidean distance)

S1 Q10 150

Proteins showing total separation

No proteins showed complete separation

Significant data (p<=0.05)

No Significant Proteins

Data for Significant proteins

	C0GROUP	C0GRP_NA	C0Spectr
8	0	ME7 S1	B35179
9	0	ME7 S1	B35180
10	0	ME7 S1	B35181
11	0	ME7 S1	B35182
12	0	ME7 S1	B35183
13	0	ME7 S1	B35184
14	0	ME7 S1	B35185
1	1	Normal S1	B35173
2	1	Normal S1	B35174
3	1	Normal S1	B35175
4	1	Normal S1	B35176
5	1	Normal S1	B35177
6	1	Normal S1	B35177
7	1	Normal S1	B35178

All data *Boxplot of all*

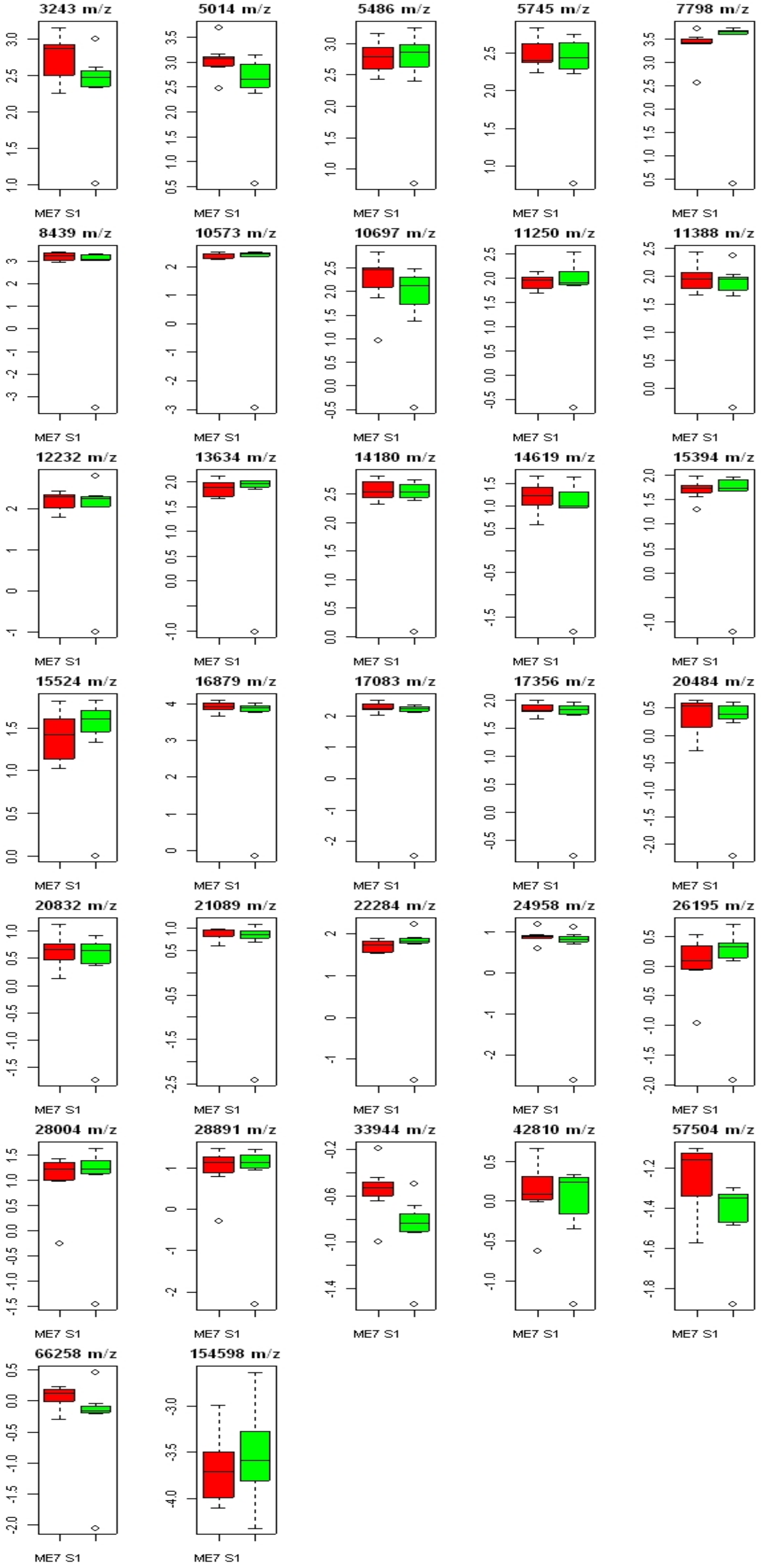

proteins

Boxplot of all proteins

Cluster Analysis of samples (Euclidean distance)

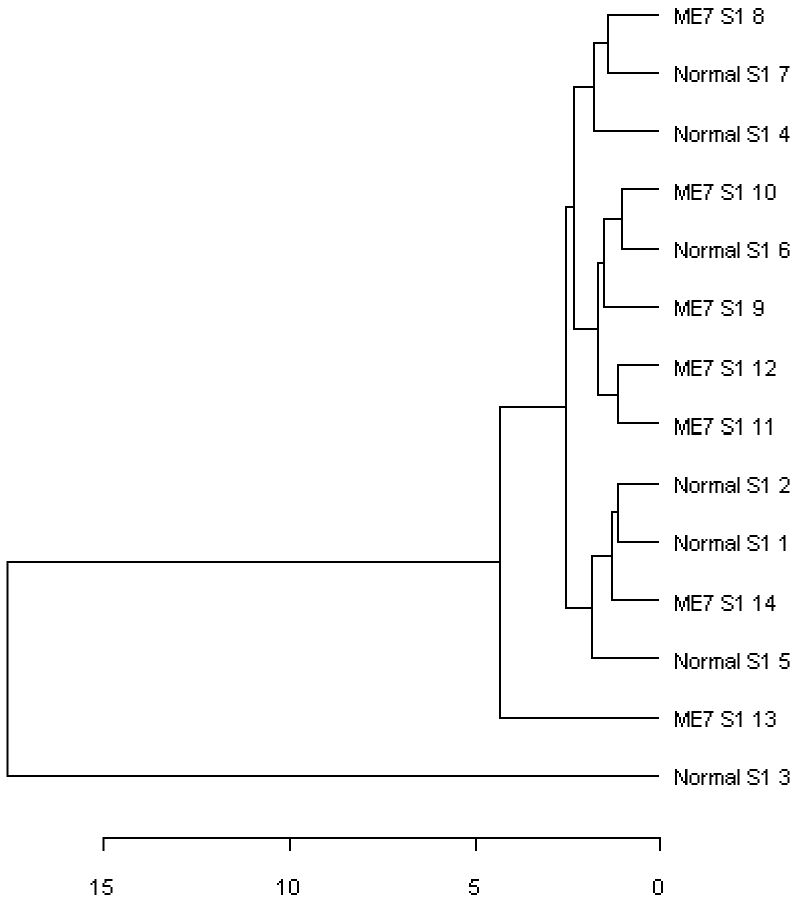

Cluster Analysis of samples (Euclidean distance)

S1 Q10 180

Proteins showing total separation

No proteins showed complete separation

Significant data (p<=0.05)

Significant Proteins (t-test; p<=0.05)

	name	mz	ME7.avg	NORM.avg	t	p
22	C022484_	22484	0.65	1.24	-2.5	0.043
28	C042483_	42483	-0.77	-0.51	-2.6	0.034

Data for Significant proteins

	C0GROUP	C0GRP_NA	C0Spectr	C022484_	C042483_
8	0	ME7 S1	B35770	0.54	-0.99
9	0	ME7 S1	B35771	-0.35	-0.97
10	0	ME7 S1	B35772	0.81	-0.54
11	0	ME7 S1	B35773	0.75	-0.80
12	0	ME7 S1	B35774	1.09	-0.46
13	0	ME7 S1	B35775	1.08	-0.83
1	1	Normal S1	B35764	1.15	-0.46
2	1	Normal S1	B35765	1.33	-0.47
3	1	Normal S1	B35766	1.47	-0.65
4	1	Normal S1	B35767	1.46	-0.46
5	1	Normal S1	B35767	1.20	-0.38
6	1	Normal S1	B35768	1.22	-0.63
7	1	Normal S1	B35769	0.86	-0.52

Boxplot of significant proteins

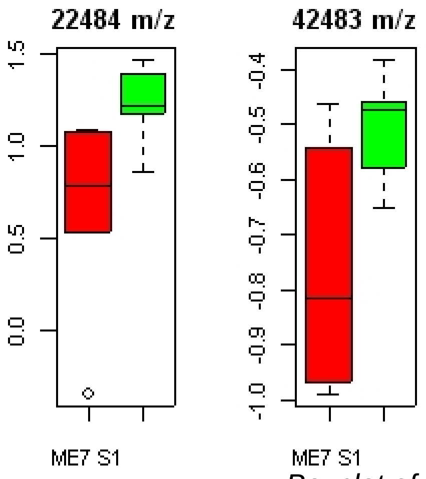

Boxplot of significant proteins

Pairwise Scatterplots of Significant Proteins

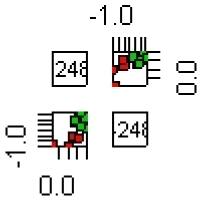

Pairwise Scatterplots of Significant Proteins

All data

Boxplot of all proteins

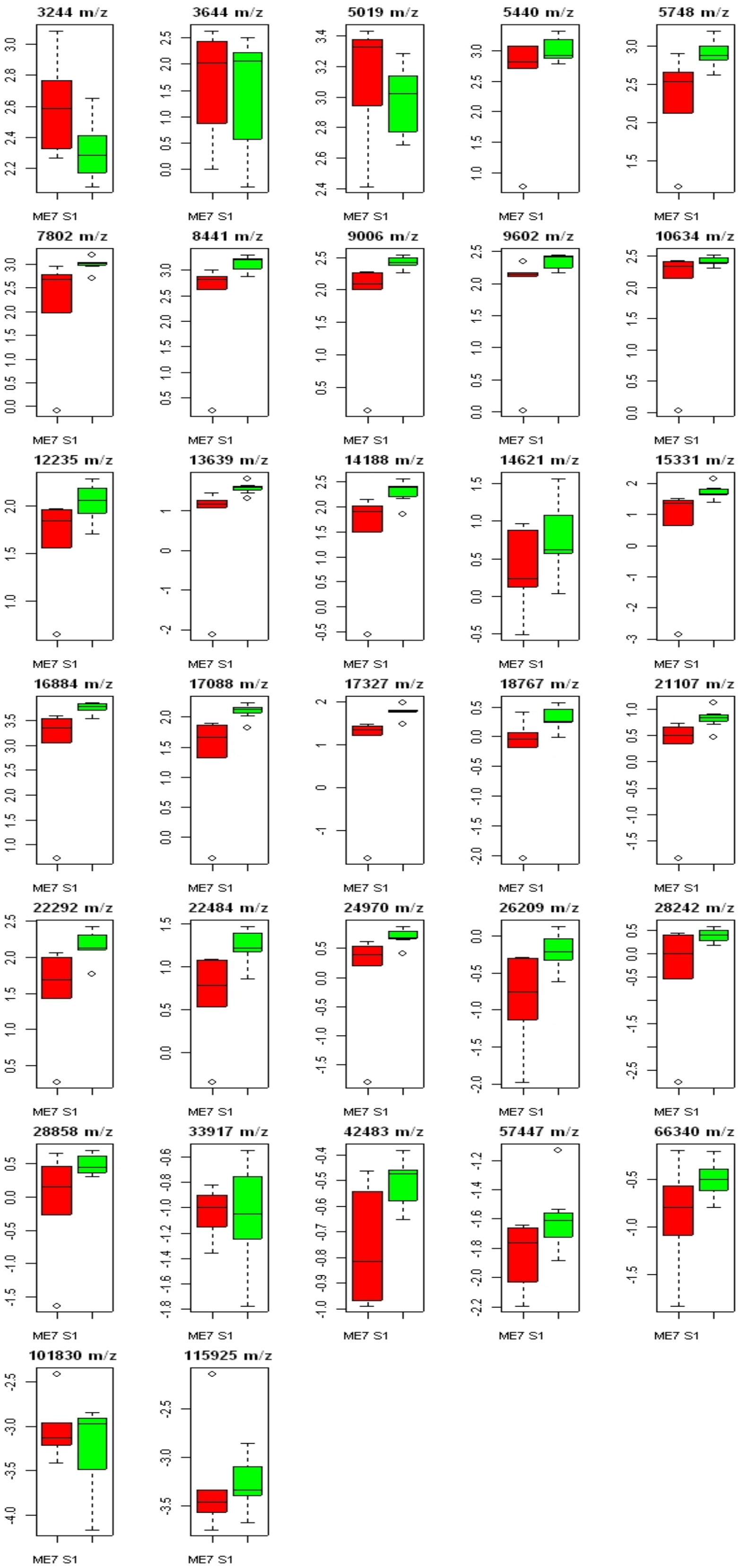

Boxplot of all proteins

Cluster Analysis of samples (Euclidean distance)

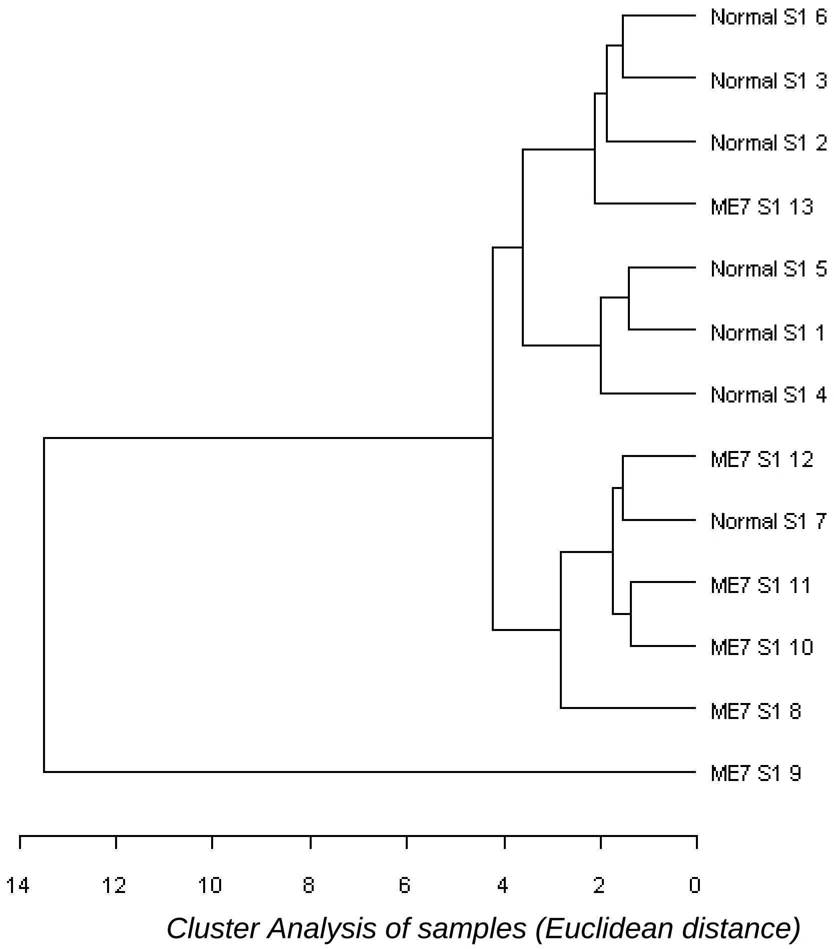

S1 Q10 210

Proteins showing total separation

	C0GROUP	C0GRP_NA	C0Spectr	C05234_1	C06272_1	C010705_	C013645_	C014191_	C014627_	C015333_
8	0	ME7 S1	B36260	2.65	2.6	2.8	1.04	2.3	1.23	1.6
9	0	ME7 S1	B36261	2.57	2.7	3.1	1.32	2.2	0.83	1.4
10	0	ME7 S1	B36261	2.61	2.7	3.2	1.21	2.3	0.90	1.5
11	0	ME7 S1	B36262	3.65	2.8	3.0	1.21	2.4	1.23	1.6
12	0	ME7 S1	B36263	3.49	2.7	3.0	0.98	1.9	0.93	1.3
13	0	ME7 S1	B36264	3.65	2.7	2.5	1.27	1.8	0.95	1.0
14	0	ME7 S1	B36265	2.87	3.0	3.5	1.36	2.5	1.49	1.8
1	1	Normal S1	B36248	1.36	1.9	2.0	1.54	2.5	1.74	2.2
2	1	Normal S1	B36249	0.90	1.8	1.9	1.74	2.6	1.79	2.1
3	1	Normal S1	B36250	0.69	2.3	2.0	1.86	2.6	1.73	2.1
4	1	Normal S1	B36251	1.12	2.1	2.2	1.79	2.8	2.04	2.2
5	1	Normal S1	B36252	1.35	2.3	2.1	1.85	2.8	2.07	2.2
6	1	Normal S1	B36253	0.61	2.3	2.3	1.71	2.8	1.82	2.3
7	1	Normal S1	B36253	1.93	2.3	2.2	1.82	2.7	1.72	2.4

Boxplot of proteins showing complete separation

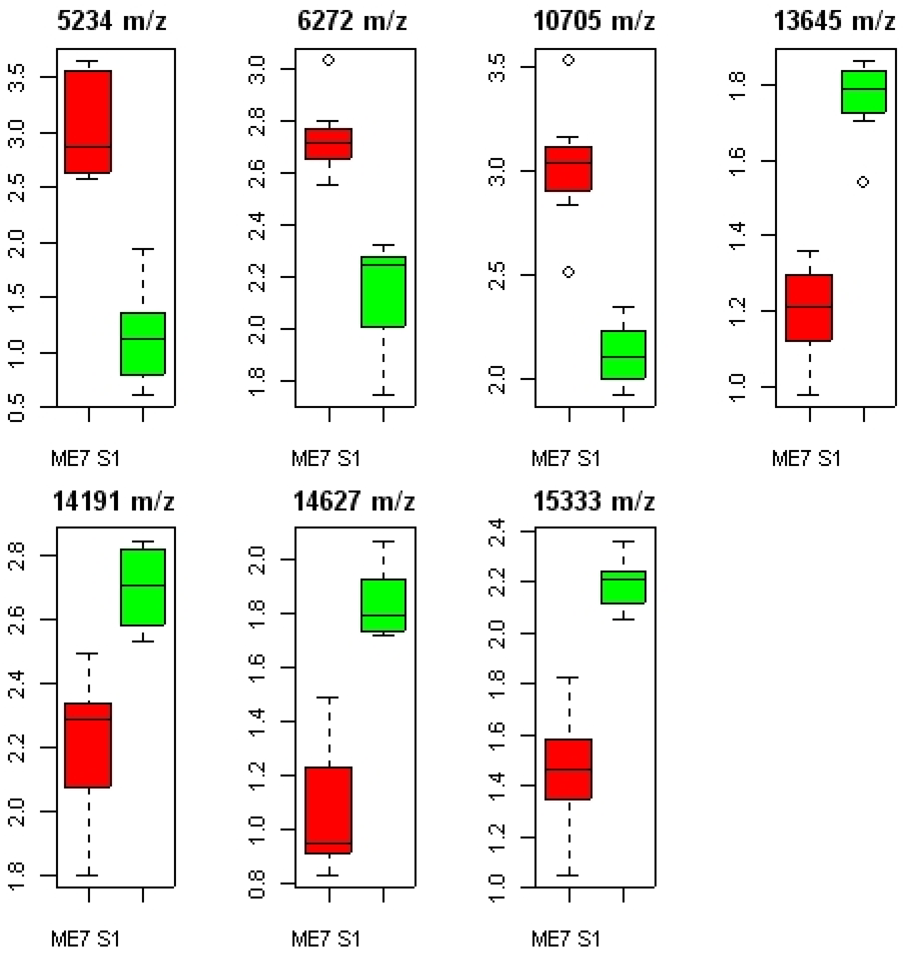

Pairwise Scatterplots of Proteins showing complete separation

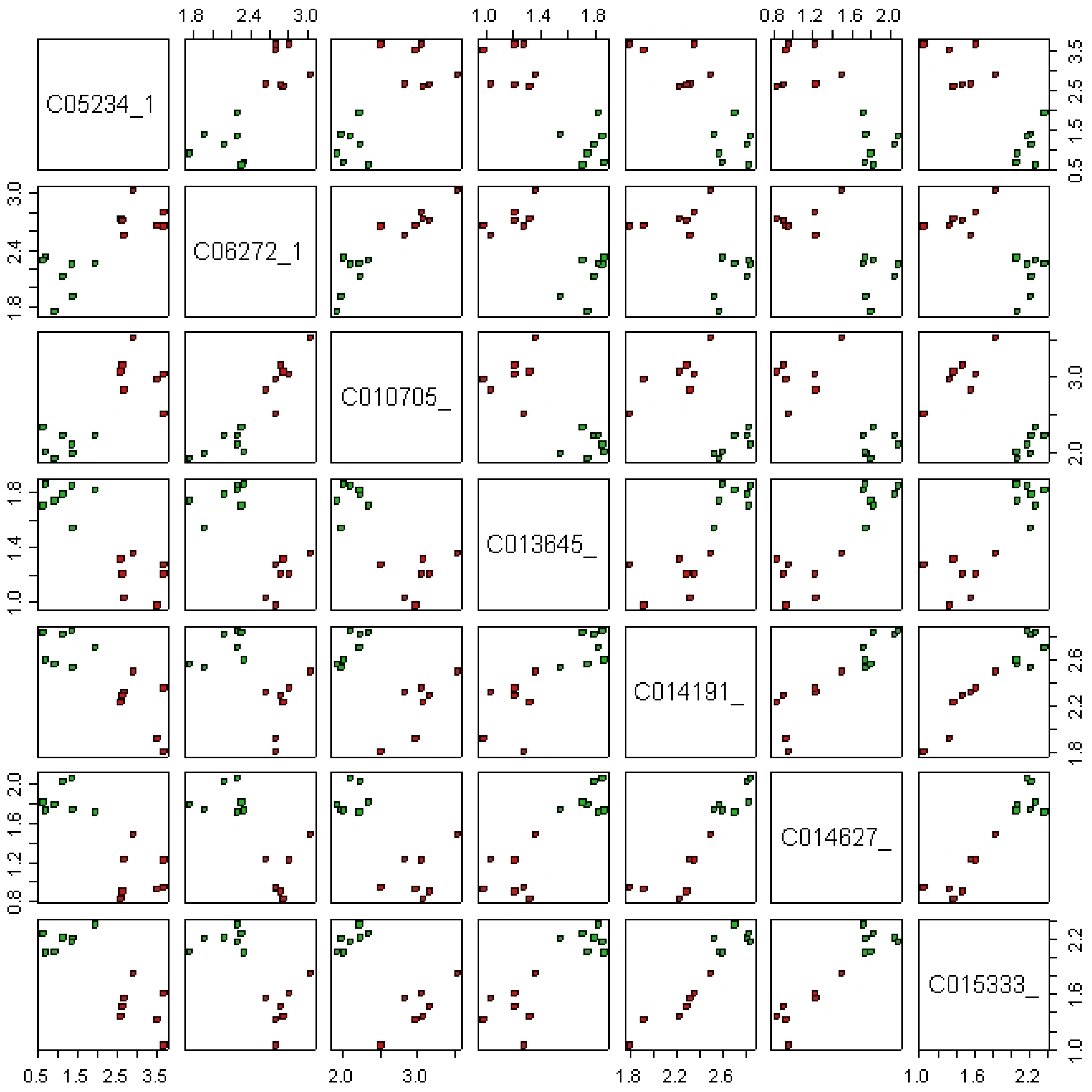

Significant data (p<=0.001)

Significant Proteins (t-test; p<=0.001)

	name	mz	ME7.avg	NORM.avg	t	p
3	C05020_7	5020	3.3	2.7	5.0	3.4e-04
4	C05234_1	5234	3.1	1.1	7.5	7.4e-06
7	C06272_1	6272	2.7	2.1	6.0	1.0e-04
8	C07858_2	7858	2.7	3.2	-5.2	9.1e-04
15	C010705_	10705	3.0	2.1	6.9	8.1e-05
19	C013645_	13645	1.2	1.8	-8.2	4.1e-06
21	C014627_	14627	1.1	1.8	-7.2	3.1e-05
23	C015333_	15333	1.5	2.2	-7.2	7.9e-05

Data for Significant proteins

	C0GROUP	C0GRP_NA	C0Spectr	C05020_7	C05234_1	C06272_1	C07858_2	C010705_	C013645_	C014627_	C015333_
8	0	ME7 S1	B36260	3.2	2.65	2.6	2.7	2.8	1.04	1.23	1.6
9	0	ME7 S1	B36261	3.0	2.57	2.7	2.8	3.1	1.32	0.83	1.4
10	0	ME7 S1	B36261	3.1	2.61	2.7	2.8	3.2	1.21	0.90	1.5
11	0	ME7 S1	B36262	3.5	3.65	2.8	2.7	3.0	1.21	1.23	1.6
12	0	ME7 S1	B36263	3.4	3.49	2.7	2.4	3.0	0.98	0.93	1.3
13	0	ME7 S1	B36264	3.6	3.65	2.7	2.4	2.5	1.27	0.95	1.0
14	0	ME7 S1	B36265	3.2	2.87	3.0	3.0	3.5	1.36	1.49	1.8
1	1	Normal S1	B36248	2.5	1.36	1.9	3.0	2.0	1.54	1.74	2.2
2	1	Normal S1	B36249	2.5	0.90	1.8	3.2	1.9	1.74	1.79	2.1
3	1	Normal S1	B36250	2.5	0.69	2.3	3.2	2.0	1.86	1.73	2.1
4	1	Normal S1	B36251	2.8	1.12	2.1	3.2	2.2	1.79	2.04	2.2
5	1	Normal S1	B36252	2.6	1.35	2.3	3.3	2.1	1.85	2.07	2.2
6	1	Normal S1	B36253	2.8	0.61	2.3	3.2	2.3	1.71	1.82	2.3
7	1	Normal S1	B36253	3.2	1.93	2.3	3.2	2.2	1.82	1.72	2.4

Boxplot of significant proteins

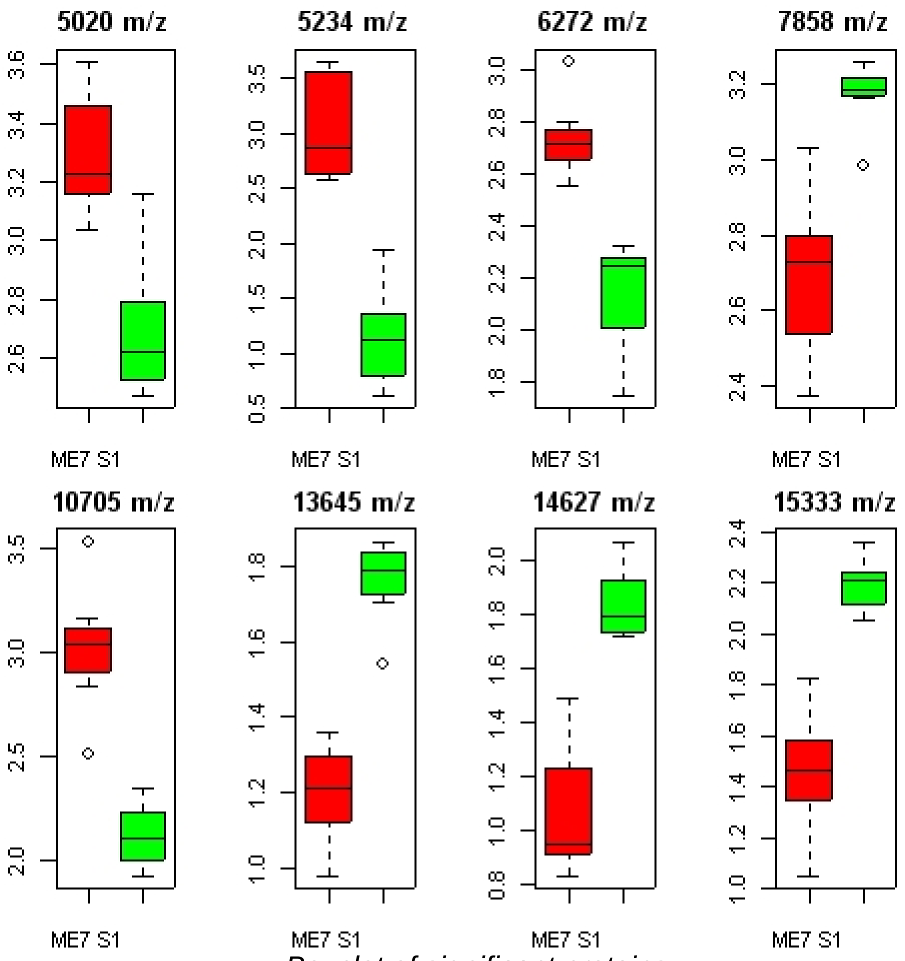

Boxplot of significant proteins

Pairwise Scatterplots of Significant Proteins

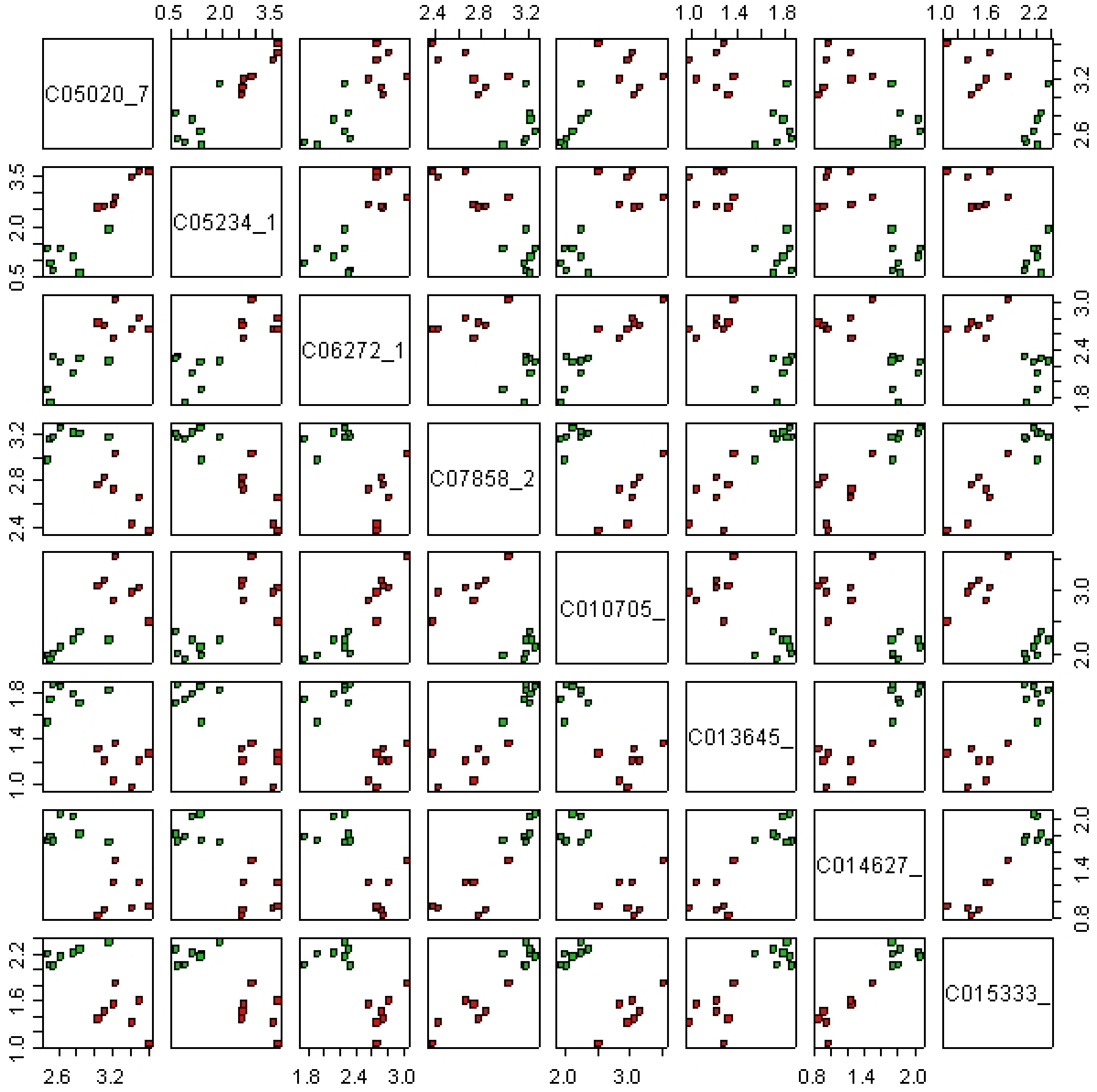

Pairwise Scatterplots of Significant Proteins

Cluster Analysis of samples (Euclidean distance)

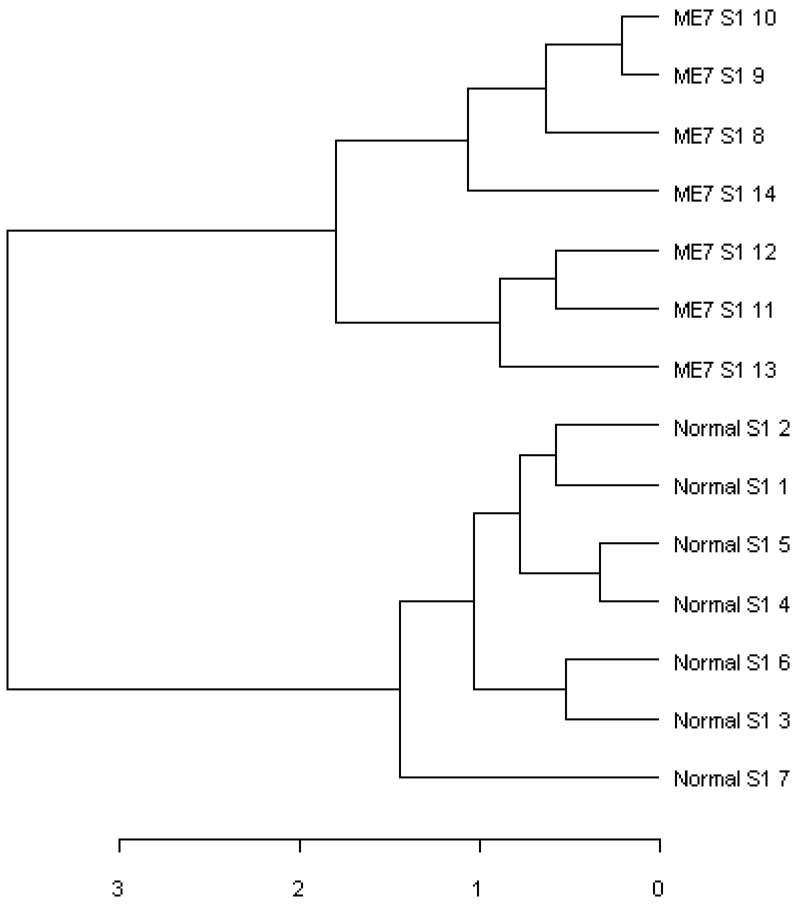

Cluster Analysis of samples (Euclidean distance)

Plot of first three principal components

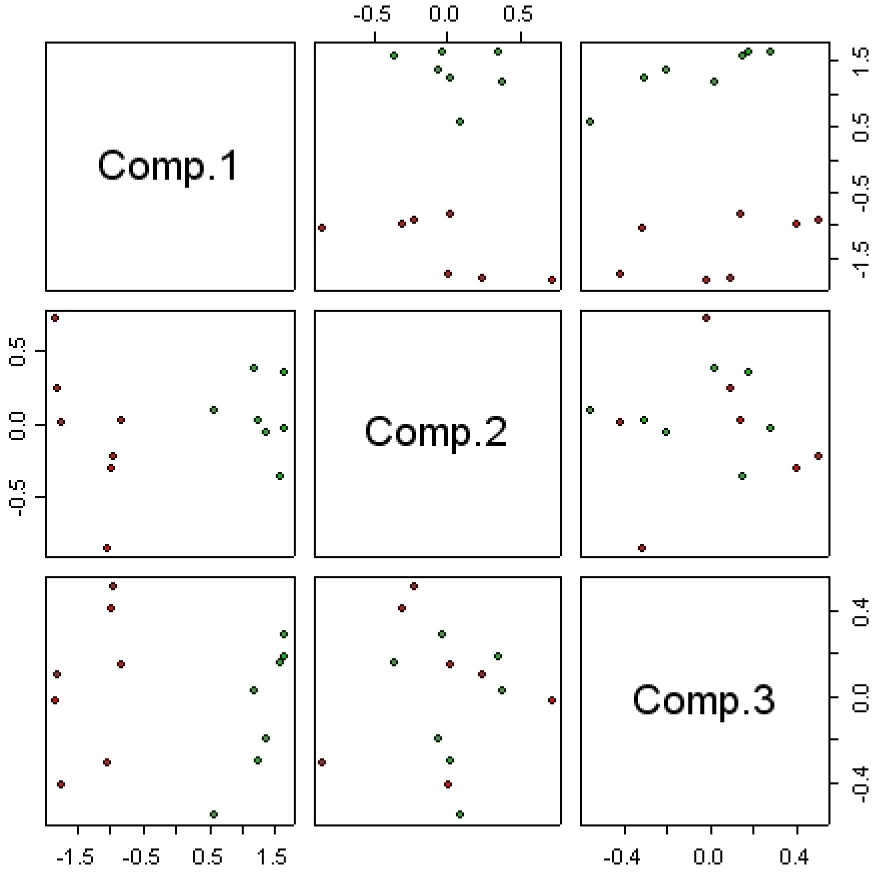

Plot of first three principal components

Scatterplot of linear discriminant function (x-axis)

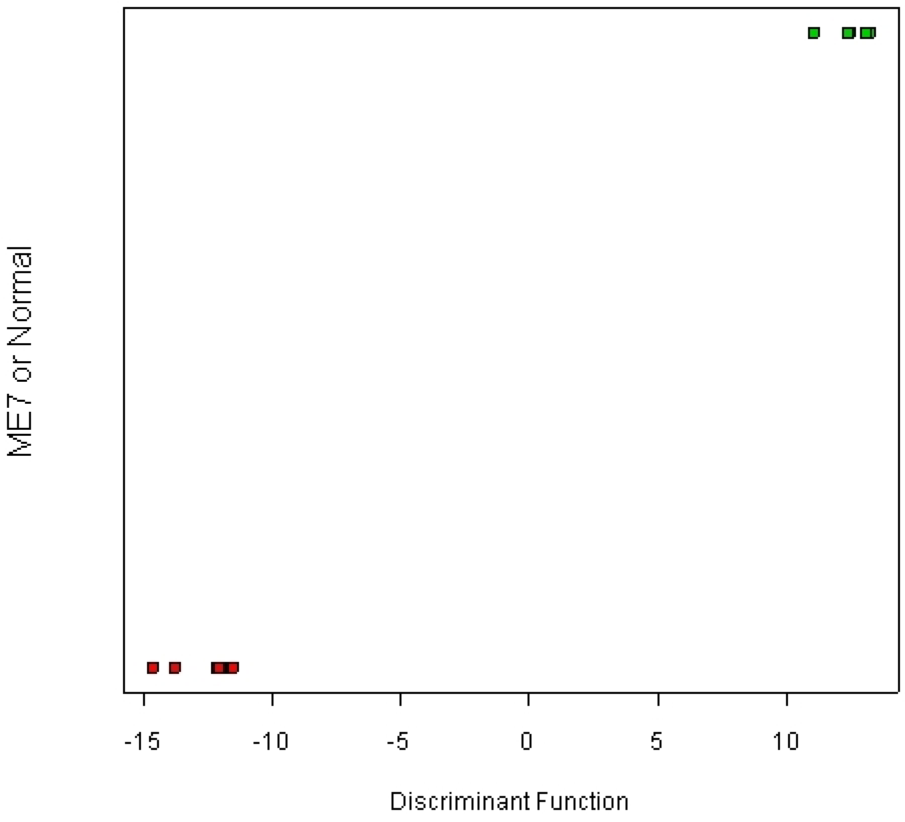

Scatterplot of linear discriminant function (x-axis)

All data

Boxplot of all proteins

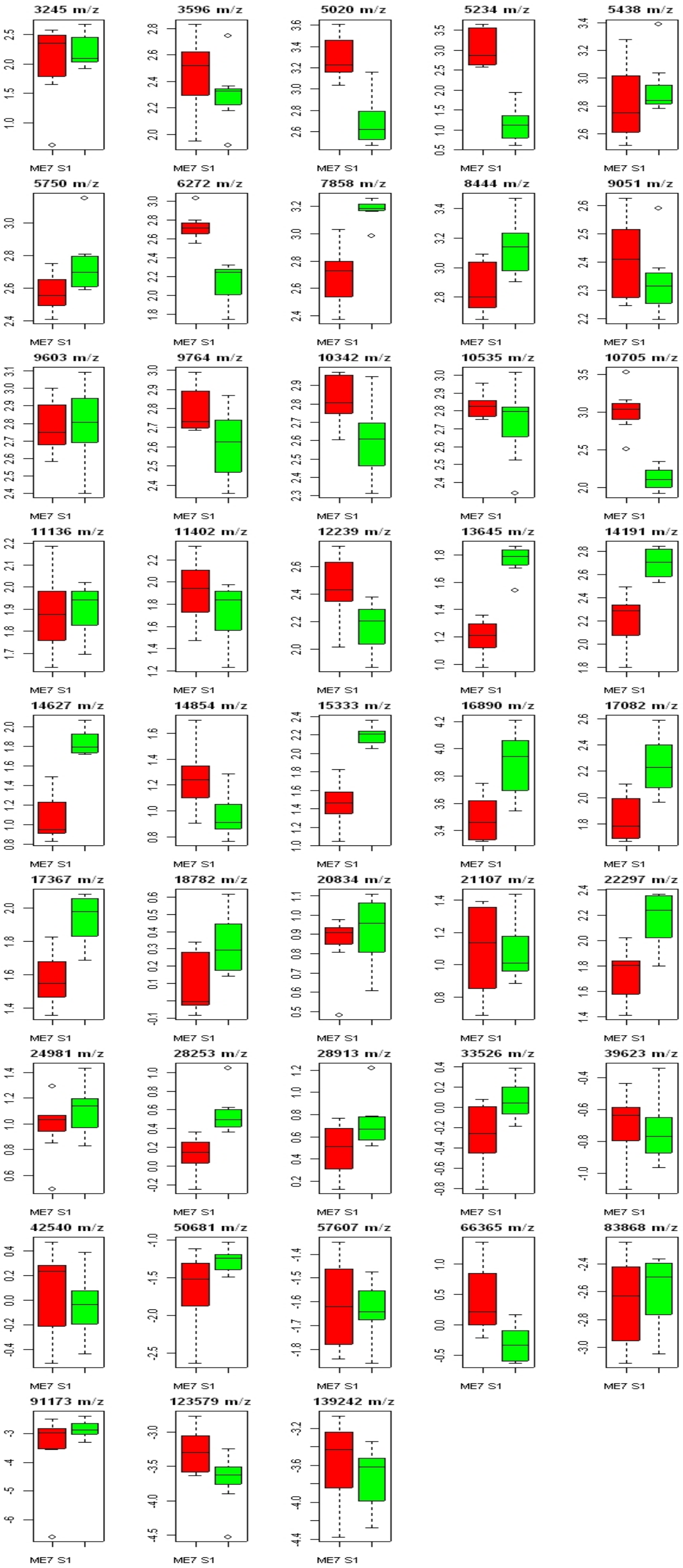

Boxplot of all proteins

Cluster Analysis of samples (Euclidean distance)

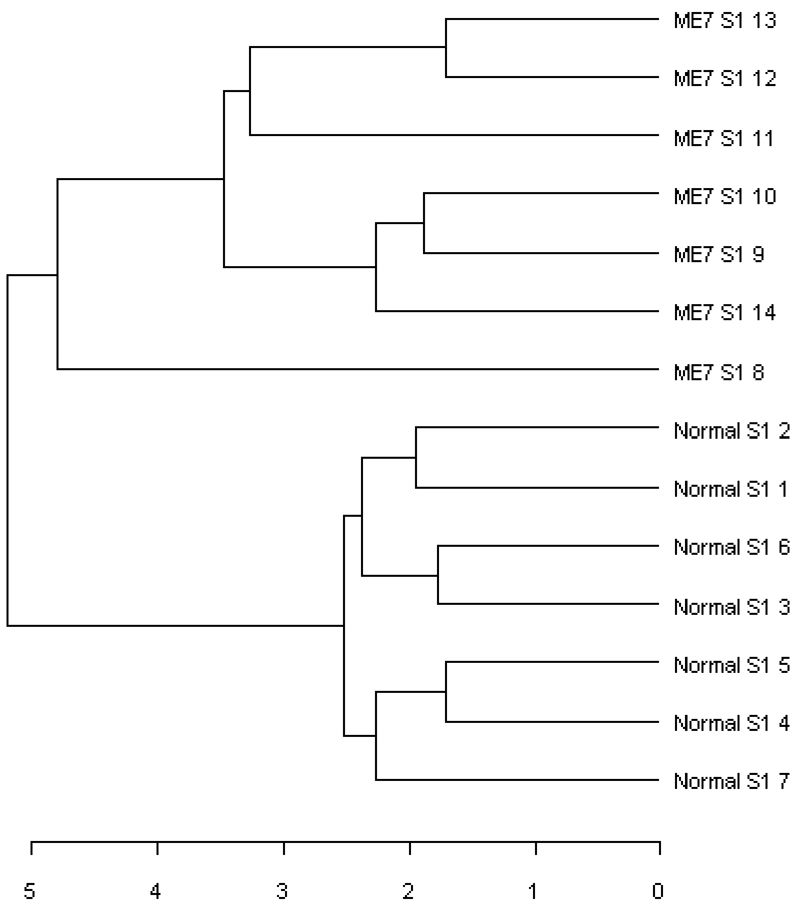

Cluster Analysis of samples (Euclidean distance)

S1 Q10 240

Proteins showing total separation

	C0GROUP	C0GRP_NA	C0Spectr	C05234_1	C013642_	C014185_	C016884_	C022291_	C028099_	C028898_
8	0	ME7 S1	B36863	2.49	0.661	2.0	3.4	1.29	0.1218	0.38
9	0	ME7 S1	B36864	2.44	0.620	2.1	3.2	1.04	0.2076	0.47
10	0	ME7 S1	B36865	2.44	0.248	1.4	3.2	0.96	-0.0466	0.15
11	0	ME7 S1	B36865	2.81	0.086	1.1	2.9	0.69	-0.1436	0.12
12	0	ME7 S1	B36866	2.58	0.772	1.9	3.3	1.11	-0.0663	0.15
13	0	ME7 S1	B36867	2.91	0.454	1.9	3.1	1.08	-0.2967	0.13
14	0	ME7 S1	B36868	3.29	0.438	1.6	3.1	0.85	0.0074	0.33
1	1	Normal S1	B36857	1.04	1.428	2.8	3.6	1.98	0.4734	0.65
2	1	Normal S1	B36858	1.20	1.304	2.3	3.4	1.72	0.4044	0.58
3	1	Normal S1	B36859	0.84	1.309	2.4	3.5	1.70	0.5800	0.66
4	1	Normal S1	B36860	1.90	1.262	2.5	3.6	1.48	0.6061	0.82
5	1	Normal S1	B36860	1.73	1.515	2.6	3.7	1.66	0.6501	0.76
6	1	Normal S1	B36861	1.12	1.469	2.5	3.6	1.79	0.6540	0.84
7	1	Normal S1	B36862	1.77	0.956	2.2	3.5	1.44	0.4623	0.60

Boxplot of proteins showing complete separation

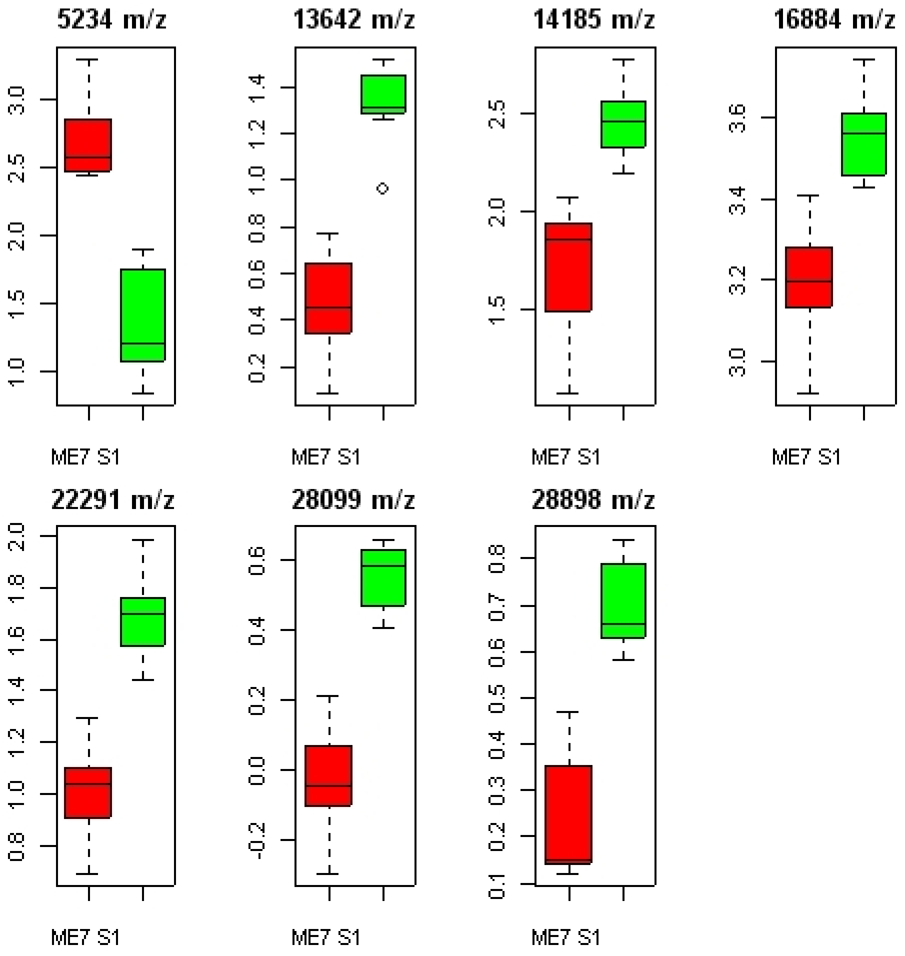

Pairwise Scatterplots of Proteins showing complete separation

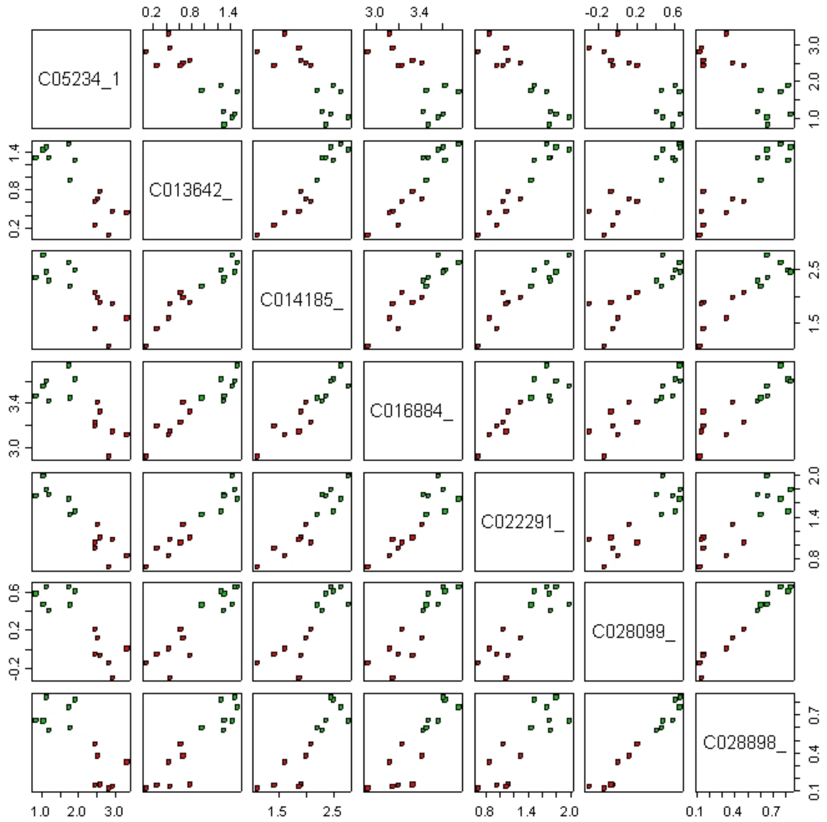

Significant data (p<=0.001)

Significant Proteins (t-test; p<=0.001)

	name	mz	ME7.avg	NORM.avg	t	p
3	C05234_1	5234	2.708	1.37	6.7	3.0e-05
6	C07670_4	7670	2.195	2.86	-5.2	2.4e-04
14	C013642_	13642	0.469	1.32	-7.4	1.2e-05
15	C014185_	14185	1.698	2.46	-4.9	7.6e-04
16	C014623_	14623	0.754	1.53	-5.0	3.2e-04
18	C015234_	15234	1.053	1.56	-5.3	2.6e-04
19	C016884_	16884	3.194	3.55	-4.9	4.9e-04
20	C017090_	17090	1.577	1.94	-5.2	2.2e-04
25	C022291_	22291	1.003	1.68	-6.7	2.2e-05
27	C028099_	28099	-0.031	0.55	-7.9	1.5e-05
28	C028898_	28898	0.247	0.70	-6.8	2.9e-05

Data for Significant proteins

	C0GROUP	C0GRP_NA	C0Spectr	C05234_1	C07670_4	C013642_	C014185_	C014623_	C015234_	C016884_	C017090_	C022291_	C028099_	C028898_
8	0	ME7 S1	B36863	2.49	2.3	0.661	2.0	1.10	1.07	3.4	1.8	1.29	0.1218	0.38
9	0	ME7 S1	B36864	2.44	2.4	0.620	2.1	0.97	1.17	3.2	1.6	1.04	0.2076	0.47
10	0	ME7 S1	B36865	2.44	1.9	0.248	1.4	1.00	0.80	3.2	1.6	0.96	-0.0466	0.15
11	0	ME7 S1	B36865	2.81	1.9	0.086	1.1	0.47	0.82	2.9	1.4	0.69	-0.1436	0.12
12	0	ME7 S1	B36866	2.58	2.1	0.772	1.9	0.46	1.01	3.3	1.7	1.11	-0.0663	0.15
13	0	ME7 S1	B36867	2.91	2.4	0.454	1.9	0.72	1.11	3.1	1.5	1.08	-0.2967	0.13
14	0	ME7 S1	B36868	3.29	2.4	0.438	1.6	0.55	1.39	3.1	1.5	0.85	0.0074	0.33
1	1	Normal S1	B36857	1.04	3.1	1.428	2.8	2.04	1.63	3.6	1.9	1.98	0.4734	0.65
2	1	Normal S1	B36858	1.20	2.9	1.304	2.3	1.48	1.68	3.4	1.8	1.72	0.4044	0.58
3	1	Normal S1	B36859	0.84	2.8	1.309	2.4	1.10	1.54	3.5	1.8	1.70	0.5800	0.66
4	1	Normal S1	B36860	1.90	2.7	1.262	2.5	1.69	1.65	3.6	2.0	1.48	0.6061	0.82
5	1	Normal S1	B36860	1.73	3.1	1.515	2.6	1.68	1.64	3.7	2.1	1.66	0.6501	0.76
6	1	Normal S1	B36861	1.12	3.0	1.469	2.5	1.37	1.53	3.6	2.1	1.79	0.6540	0.84
7	1	Normal S1	B36862	1.77	2.4	0.956	2.2	1.33	1.26	3.5	1.8	1.44	0.4623	0.60

Boxplot of significant proteins

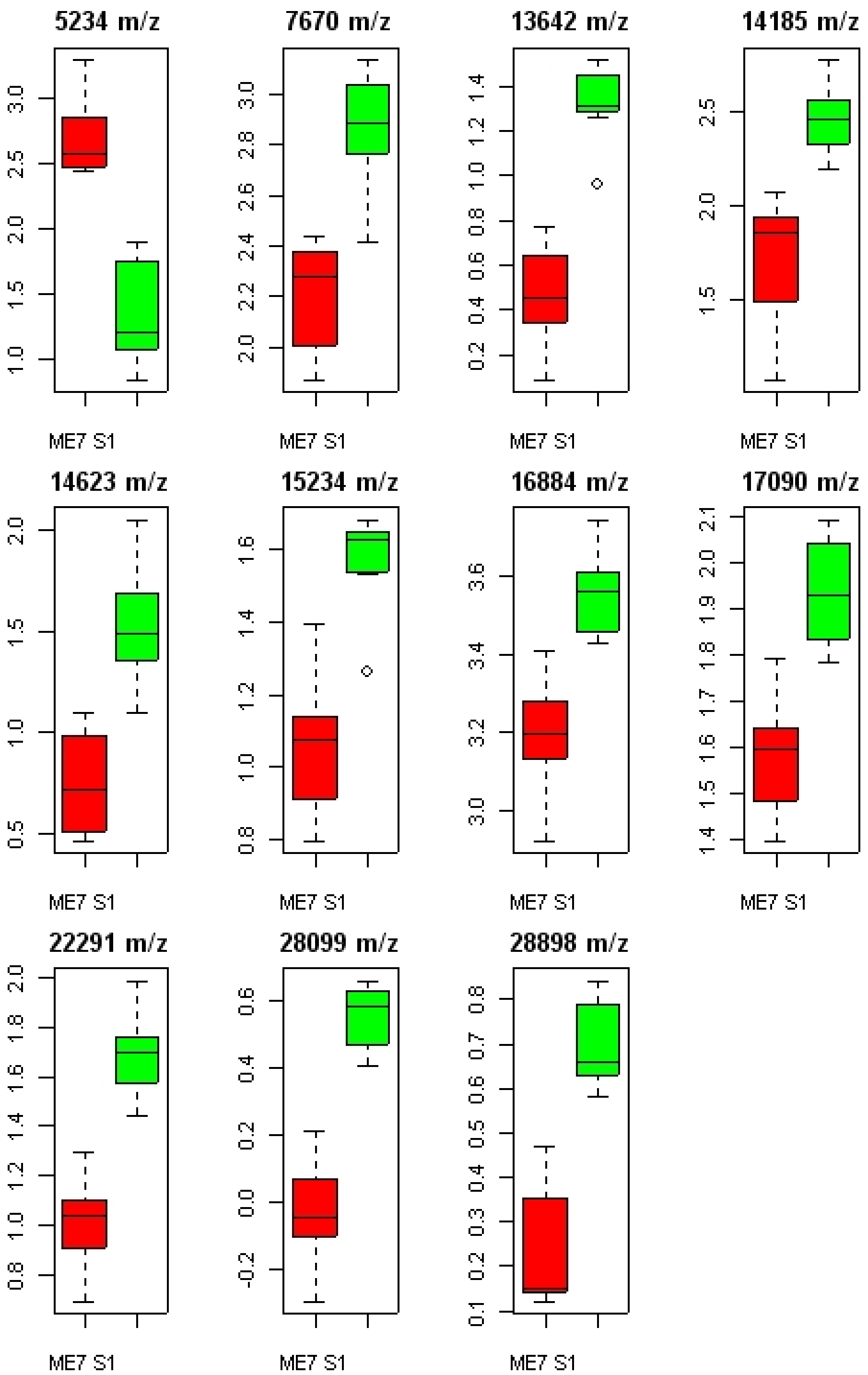

Boxplot of significant proteins

Pairwise Scatterplots of Significant Proteins

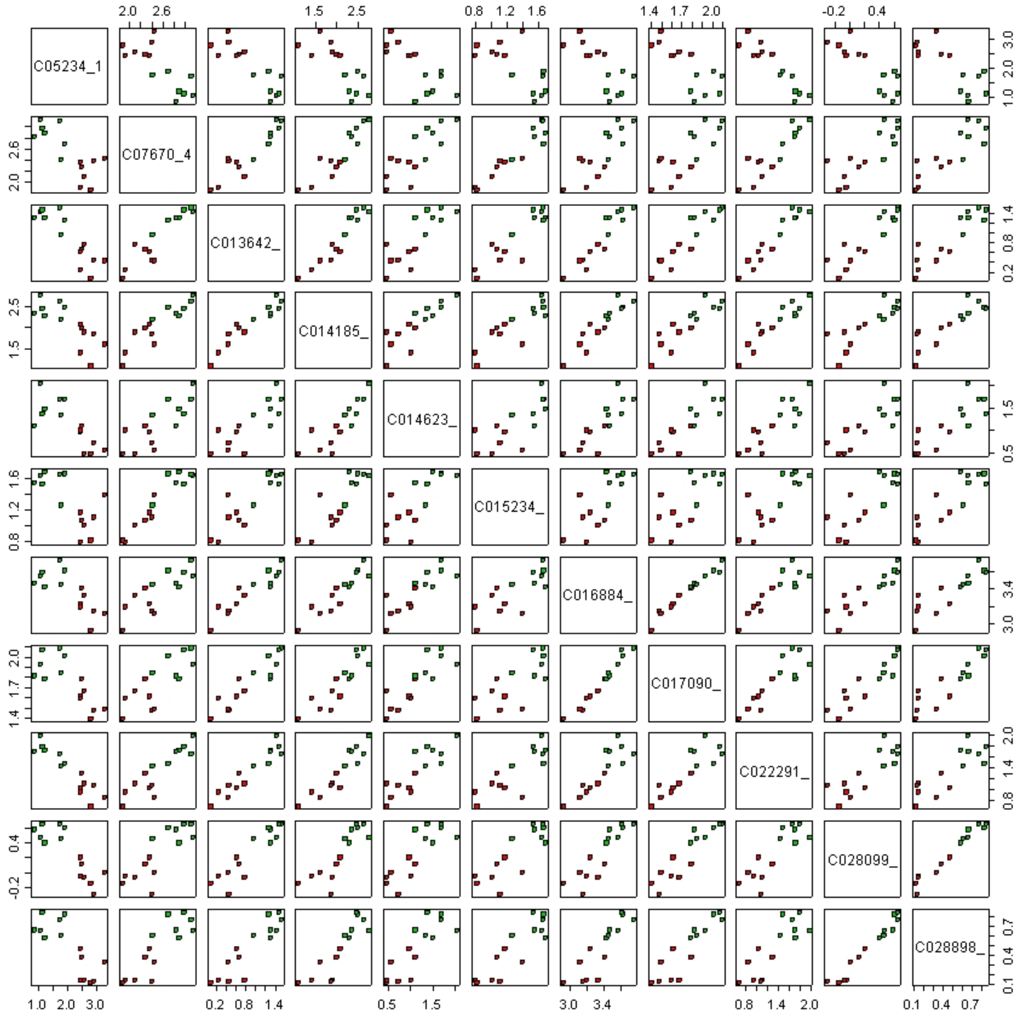

Pairwise Scatterplots of Significant Proteins

Cluster Analysis of samples (Euclidean distance)

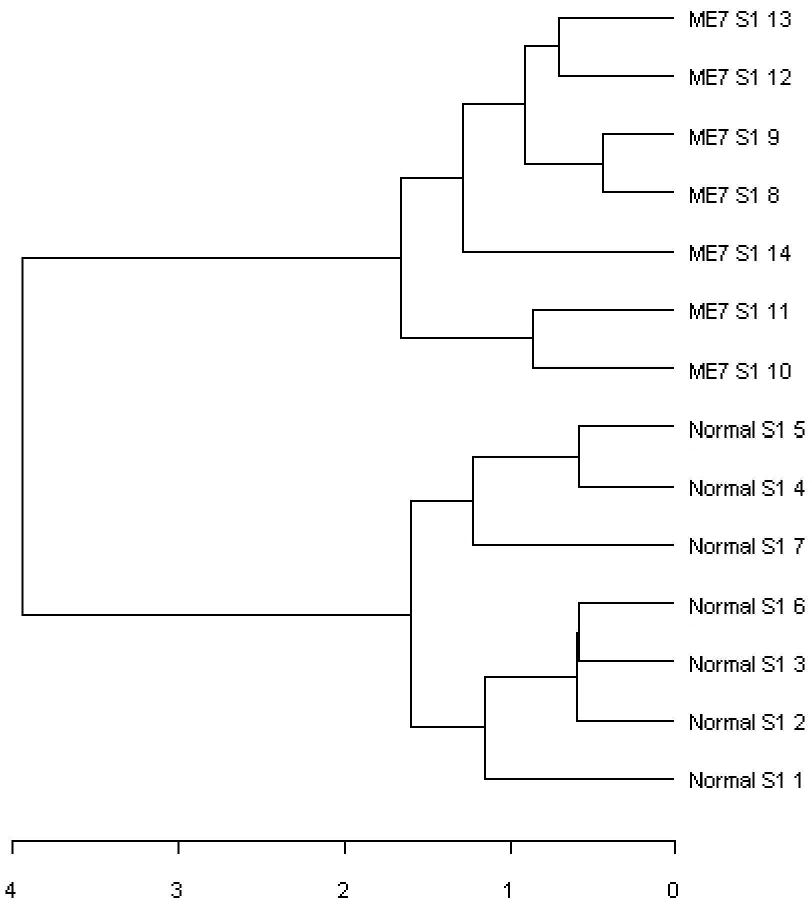

Cluster Analysis of samples (Euclidean distance)

Plot of first three principal components

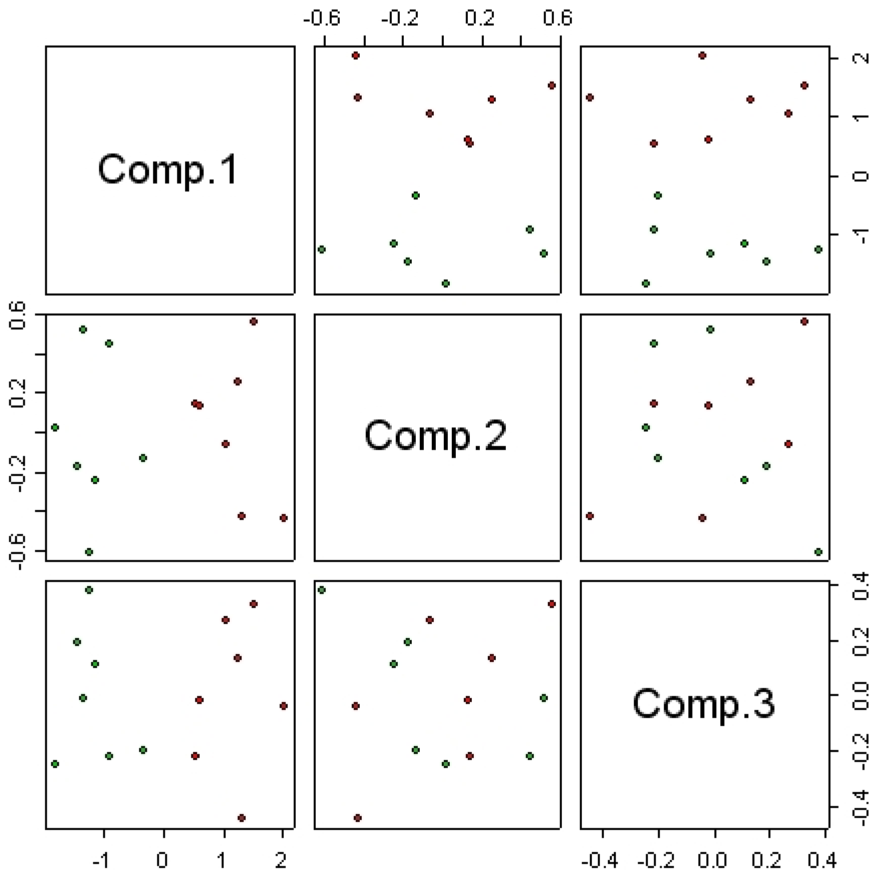

Plot of first three principal components

Scatterplot of linear discriminant function (x-axis)

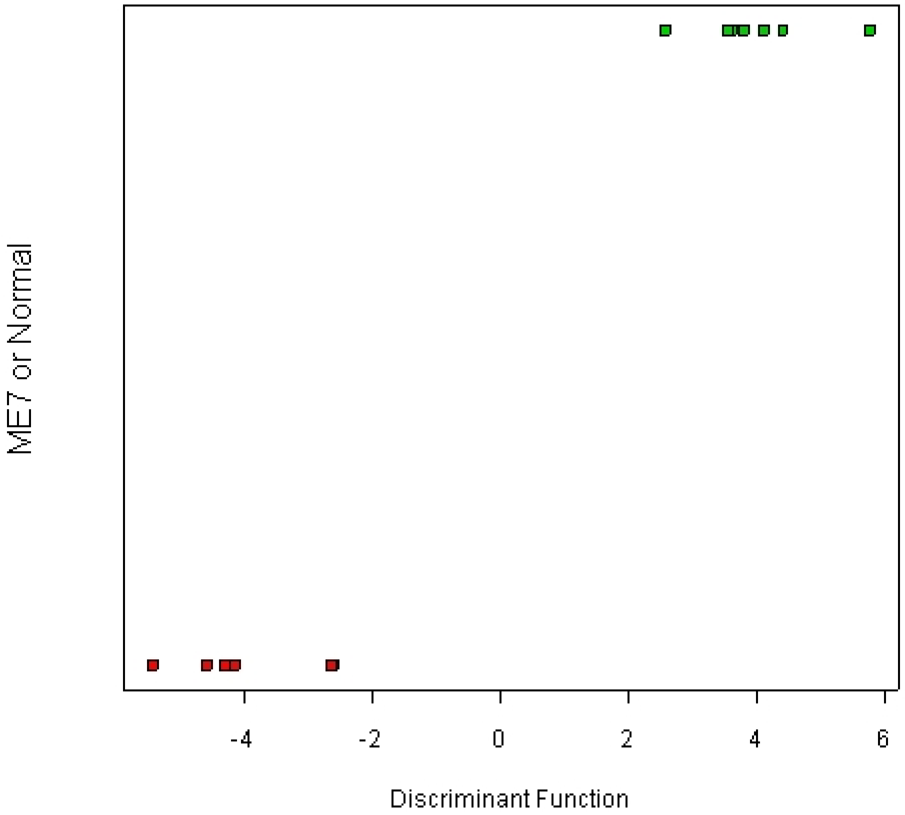

Scatterplot of linear discriminant function (x-axis)

All data*Boxplot of all proteins*

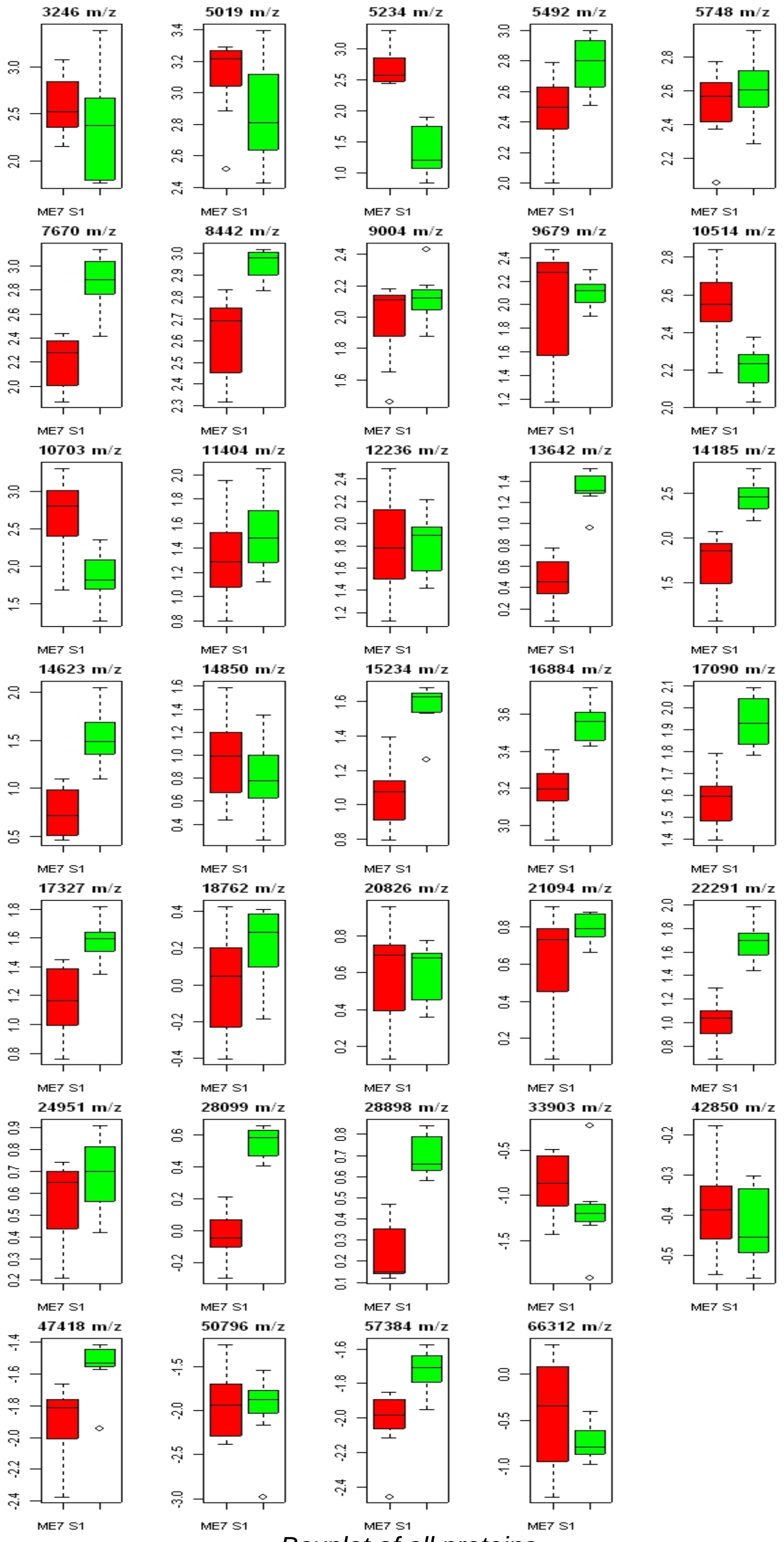

Boxplot of all proteins

Cluster Analysis of samples (Euclidean distance)

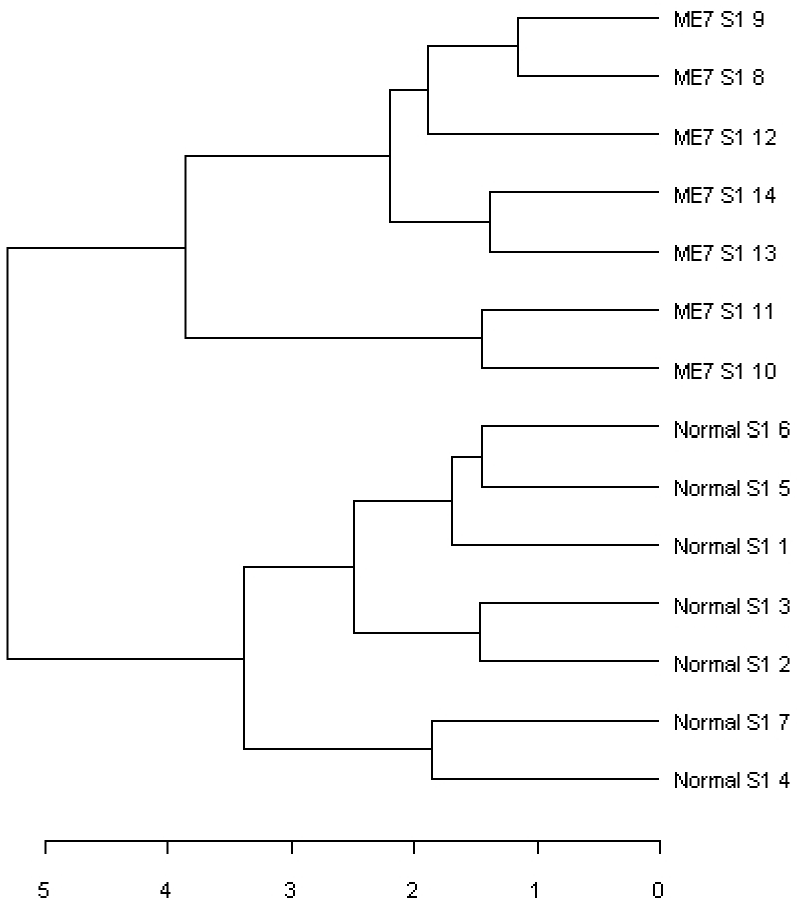

Cluster Analysis of samples (Euclidean distance)

S1 Q10 EP

S1 Q10 EP

Proteins showing total separation

	GROUP	GRP_NA	Spectr	5234	6054	8445	10085	10483	10707	13638	14189	14863	15460	16889	17084	17327	22295	26207	28226
1	0	ME7 S1	B36885	2.98	1.8	2.5	2.06	2.8	3.3	0.79	1.7	1.14	0.93	3.1	1.5	0.96	1.1	-0.52	0.084
2	0	ME7 S1	B36885	2.98	1.8	2.5	2.06	2.8	3.3	0.79	1.7	1.14	0.93	3.1	1.5	0.96	1.1	-0.52	0.084
5	0	ME7 S1	B37003	3.67	1.5	2.8	2.46	2.9	3.3	0.86	1.7	1.21	0.93	3.1	1.6	1.06	1.1	-0.56	0.149
6	0	ME7 S1	B37003	3.67	1.5	2.8	2.46	2.9	3.3	0.86	1.7	1.21	0.93	3.1	1.6	1.06	1.1	-0.56	0.149
7	0	ME7 S1	B37004	3.28	-4.0	2.4	2.72	2.9	3.8	1.03	2.1	1.31	1.40	3.1	1.4	1.12	1.0	-0.64	-0.159
8	0	ME7 S1	B37020	3.47	1.7	2.7	2.81	3.0	3.3	0.79	1.7	1.64	1.13	3.2	1.6	1.09	1.0	-0.98	-0.178
10	0	ME7 S1	B37134	3.00	1.5	2.9	2.79	3.0	3.4	0.76	1.7	1.31	1.07	3.2	1.6	0.92	1.1	-0.91	-0.226
11	0	ME7 S1	B37134	3.00	1.5	2.9	2.79	3.0	3.4	0.76	1.7	1.31	1.07	3.2	1.6	0.92	1.1	-0.91	-0.226
12	0	ME7 S1	B37283	3.67	1.7	2.4	2.66	3.1	3.6	0.93	1.9	1.45	0.31	3.2	1.5	1.28	1.4	-0.48	-0.031
15	0	ME7 S1	B37285	3.20	1.6	2.8	2.26	3.0	3.5	1.19	2.0	1.39	0.47	3.4	1.9	1.40	1.3	-0.18	0.427
3	1	Normal S1	B37002	0.60	2.7	3.3	1.14	2.3	2.4	1.76	3.0	1.03	1.86	3.9	2.3	1.84	2.0	0.25	0.844
4	1	Normal S1	B37002	0.60	2.7	3.3	1.14	2.3	2.4	1.76	3.0	1.03	1.86	3.9	2.3	1.84	2.0	0.25	0.844
9	1	Normal S1	B37133	1.37	2.6	3.0	1.03	2.1	2.3	1.86	2.6	0.63	1.61	3.6	2.0	1.78	2.1	0.32	0.606
13	1	Normal S1	B37284	1.85	2.6	3.1	1.19	2.1	2.5	1.82	2.6	0.22	1.94	3.6	2.0	1.62	1.9	0.14	0.720
14	1	Normal S1	B37284	1.85	2.6	3.1	1.19	2.1	2.5	1.82	2.6	0.22	1.94	3.6	2.0	1.62	1.9	0.14	0.720
16	1	Normal S1	B37329	0.85	2.9	3.3	0.80	2.0	2.2	1.92	2.6	0.61	1.86	3.7	2.1	1.69	2.1	0.18	0.705
17	1	Normal S1	B37329	0.85	2.9	3.3	0.80	2.0	2.2	1.92	2.6	0.61	1.86	3.7	2.1	1.69	2.1	0.18	0.705
18	1	Normal S1	B37499	1.52	2.7	3.3	0.74	2.3	2.3	1.84	2.6	1.06	1.95	3.8	2.2	1.67	1.8	0.29	0.654
19	1	Normal S1	B37500	0.89	2.6	3.2	0.69	2.0	2.3	1.99	2.7	0.59	2.05	3.7	2.1	1.71	2.1	0.47	0.923
20	1	Normal S1	B37501	1.29	2.7	3.0	0.47	2.1	2.1	1.48	2.6	0.75	1.67	3.7	2.2	1.82	1.5	0.30	0.916
21	1	Normal S1	B37501	1.29	2.7	3.0	0.47	2.1	2.1	1.48	2.6	0.75	1.67	3.7	2.2	1.82	1.5	0.30	0.916

S1 Q10 EP

Boxplot of proteins showing complete separation

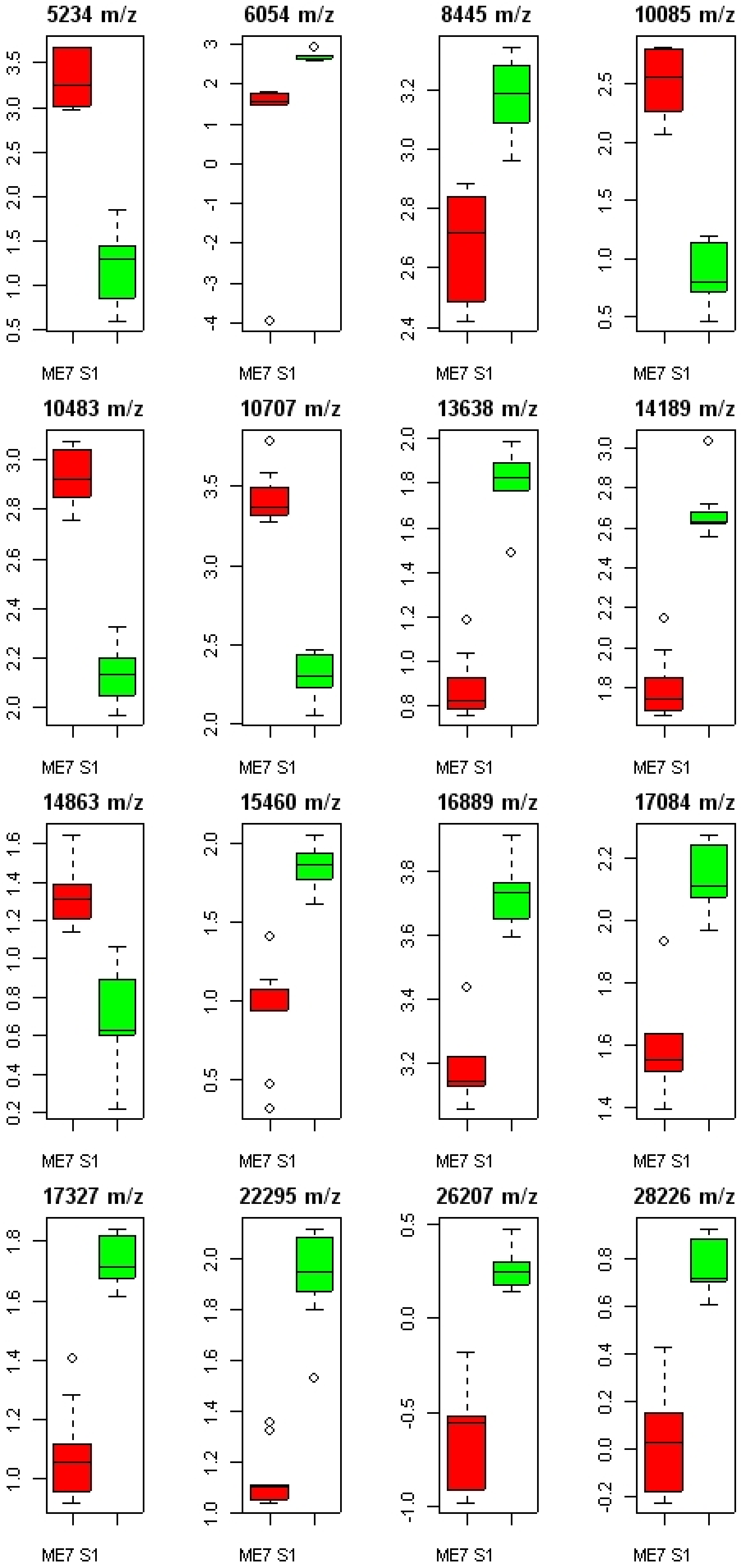

S1 Q10 EP

Pairwise Scatterplots of Proteins showing complete separation

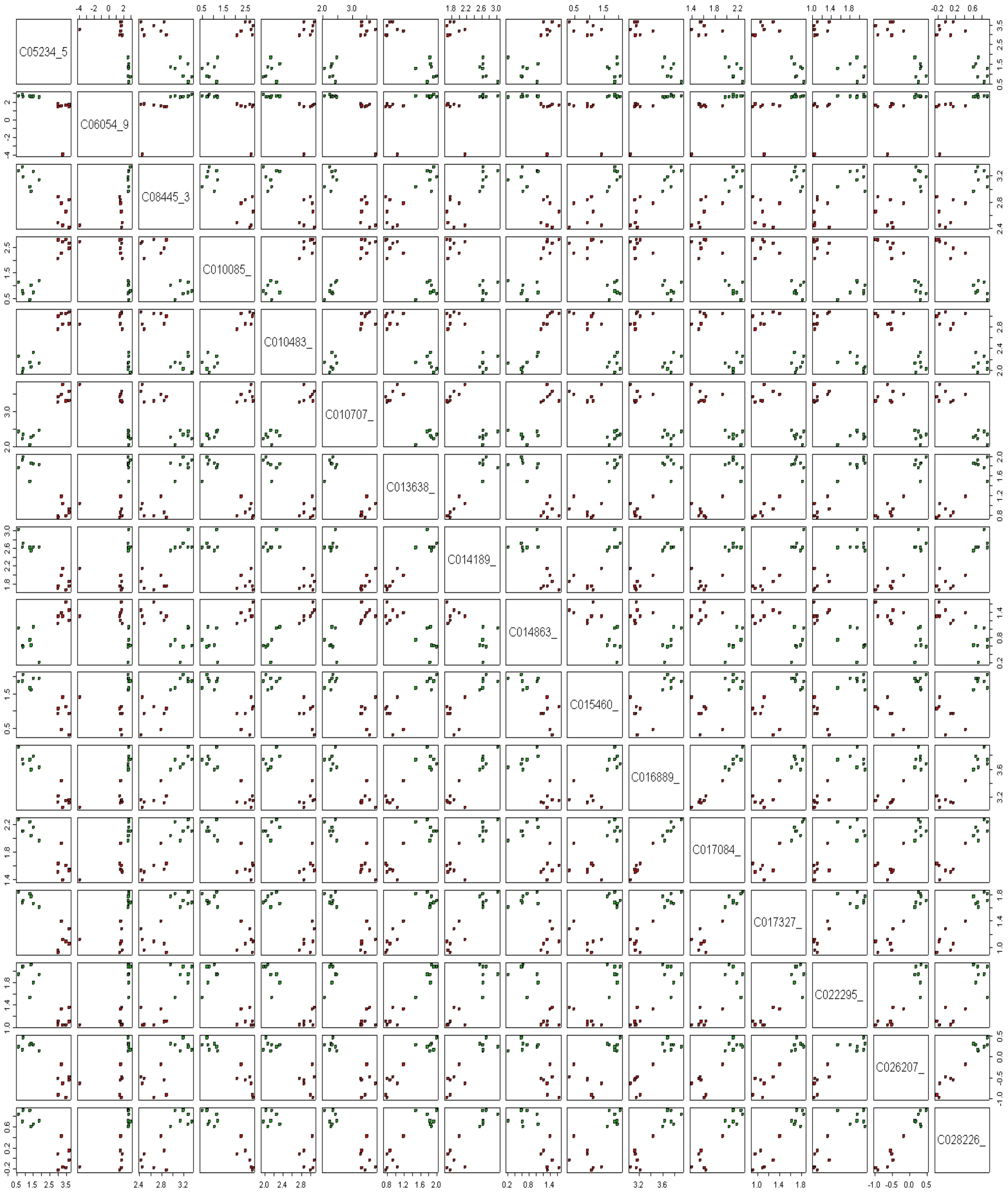

S1 Q10 EP

Significant data (p<=1e-04)

Significant Proteins (t-test; p<=1e-04)

	name	mz	ME7.avg	NORM.avg	t	p
3	C05234_5	5234	3.2926	1.18	12.7	2.8e-10
4	C05469_2	5469	2.6341	3.06	-5.8	1.9e-05
9	C07800_9	7800	2.4870	3.14	-8.5	1.7e-07
10	C08445_3	8445	2.6732	3.18	-7.0	3.8e-06
13	C010085_	10085	2.5071	0.88	13.1	8.5e-11
14	C010483_	10483	2.9222	2.13	15.1	6.2e-12
16	C010707_	10707	3.4203	2.30	16.4	2.2e-12
22	C013638_	13638	0.8764	1.79	-13.8	2.6e-11
23	C014189_	14189	1.8032	2.70	-12.9	7.5e-11
24	C014619_	14619	0.6220	1.53	-6.7	7.8e-06
25	C014863_	14863	1.3128	0.68	6.3	1.3e-05
27	C015460_	15460	0.9167	1.84	-8.6	1.8e-06
28	C016889_	16889	3.1767	3.74	-12.3	1.8e-10
29	C017084_	17084	1.5871	2.14	-9.9	1.5e-08
30	C017327_	17327	1.0764	1.73	-11.6	2.1e-08
31	C018759_	18759	0.0562	0.47	-5.4	3.4e-05
34	C022295_	22295	1.1288	1.91	-10.7	1.1e-08
36	C026207_	26207	-0.6243	0.26	-10.6	2.7e-07
37	C028226_	28226	0.0073	0.78	-10.3	9.2e-08
38	C028896_	28896	0.3270	0.88	-7.8	7.3e-07

S1 Q10 EP

Data for Significant proteins

	GROUP	GRPNA	Spectr	5234	5469	7800	8445	10085	10483	10707	13638	14189	14619	14863	15460	16889	17084	17327	18759	22295	26207	28226	28896_
1	0	ME7 S1	B36885	2.98	2.5	2.5	2.5	2.06	2.8	3.3	0.79	1.7	0.37	1.14	0.93	3.1	1.5	0.96	-0.148	1.1	-0.52	0.084	0.276
2	0	ME7 S1	B36885	2.98	2.5	2.5	2.5	2.06	2.8	3.3	0.79	1.7	0.37	1.14	0.93	3.1	1.5	0.96	-0.148	1.1	-0.52	0.084	0.276
5	0	ME7 S1	B37003	3.67	2.8	2.4	2.8	2.46	2.9	3.3	0.86	1.7	0.48	1.21	0.93	3.1	1.6	1.06	0.102	1.1	-0.56	0.149	0.338
6	0	ME7 S1	B37003	3.67	2.8	2.4	2.8	2.46	2.9	3.3	0.86	1.7	0.48	1.21	0.93	3.1	1.6	1.06	0.102	1.1	-0.56	0.149	0.338
7	0	ME7 S1	B37004	3.28	2.4	2.9	2.4	2.72	2.9	3.8	1.03	2.1	0.96	1.31	1.40	3.1	1.4	1.12	-0.106	1.0	-0.64	-0.159	0.337
8	0	ME7 S1	B37020	3.47	2.7	2.2	2.7	2.81	3.0	3.3	0.79	1.7	0.70	1.64	1.13	3.2	1.6	1.09	0.064	1.0	-0.98	-0.178	0.093
10	0	ME7 S1	B37134	3.00	2.7	2.4	2.9	2.79	3.0	3.4	0.76	1.7	0.71	1.31	1.07	3.2	1.6	0.92	0.073	1.1	-0.91	-0.226	0.195
11	0	ME7 S1	B37134	3.00	2.7	2.4	2.9	2.79	3.0	3.4	0.76	1.7	0.71	1.31	1.07	3.2	1.6	0.92	0.073	1.1	-0.91	-0.226	0.195
12	0	ME7 S1	B37283	3.67	2.6	2.6	2.4	2.66	3.1	3.6	0.93	1.9	0.75	1.45	0.31	3.2	1.5	1.28	0.192	1.4	-0.48	-0.031	0.437
15	0	ME7 S1	B37285	3.20	2.8	2.6	2.8	2.26	3.0	3.5	1.19	2.0	0.71	1.39	0.47	3.4	1.9	1.40	0.357	1.3	-0.18	0.427	0.783
3	1	Normal S1	B37002	0.60	3.0	3.2	3.3	1.14	2.3	2.4	1.76	3.0	1.89	1.03	1.86	3.9	2.3	1.84	0.458	2.0	0.25	0.844	0.982
4	1	Normal S1	B37002	0.60	3.0	3.2	3.3	1.14	2.3	2.4	1.76	3.0	1.89	1.03	1.86	3.9	2.3	1.84	0.458	2.0	0.25	0.844	0.982
9	1	Normal S1	B37133	1.37	3.0	3.2	3.0	1.03	2.1	2.3	1.86	2.6	1.55	0.63	1.61	3.6	2.0	1.78	0.453	2.1	0.32	0.606	0.863
13	1	Normal S1	B37284	1.85	3.2	3.0	3.1	1.19	2.1	2.5	1.82	2.6	0.78	0.22	1.94	3.6	2.0	1.62	0.619	1.9	0.14	0.720	0.822
14	1	Normal S1	B37284	1.85	3.2	3.0	3.1	1.19	2.1	2.5	1.82	2.6	0.78	0.22	1.94	3.6	2.0	1.62	0.619	1.9	0.14	0.720	0.822
16	1	Normal S1	B37329	0.85	3.2	3.3	3.3	0.80	2.0	2.2	1.92	2.6	1.63	0.61	1.86	3.7	2.1	1.69	0.575	2.1	0.18	0.705	0.701
17	1	Normal S1	B37329	0.85	3.2	3.3	3.3	0.80	2.0	2.2	1.92	2.6	1.63	0.61	1.86	3.7	2.1	1.69	0.575	2.1	0.18	0.705	0.701
18	1	Normal S1	B37499	1.52	3.2	3.1	3.3	0.74	2.3	2.3	1.84	2.6	1.92	1.06	1.95	3.8	2.2	1.67	0.574	1.8	0.29	0.654	0.804
19	1	Normal S1	B37500	0.89	3.2	3.3	3.2	0.69	2.0	2.3	1.99	2.7	1.75	0.59	2.05	3.7	2.1	1.71	0.671	2.1	0.47	0.923	0.914
20	1	Normal S1	B37501	1.29	2.7	2.9	3.0	0.47	2.1	2.1	1.48	2.6	1.50	0.75	1.67	3.7	2.2	1.82	0.106	1.5	0.30	0.916	1.072
21	1	Normal S1	B37501	1.29	2.7	2.9	3.0	0.47	2.1	2.1	1.48	2.6	1.50	0.75	1.67	3.7	2.2	1.82	0.106	1.5	0.30	0.916	1.072

S1 Q10 EP

Boxplot of significant proteins

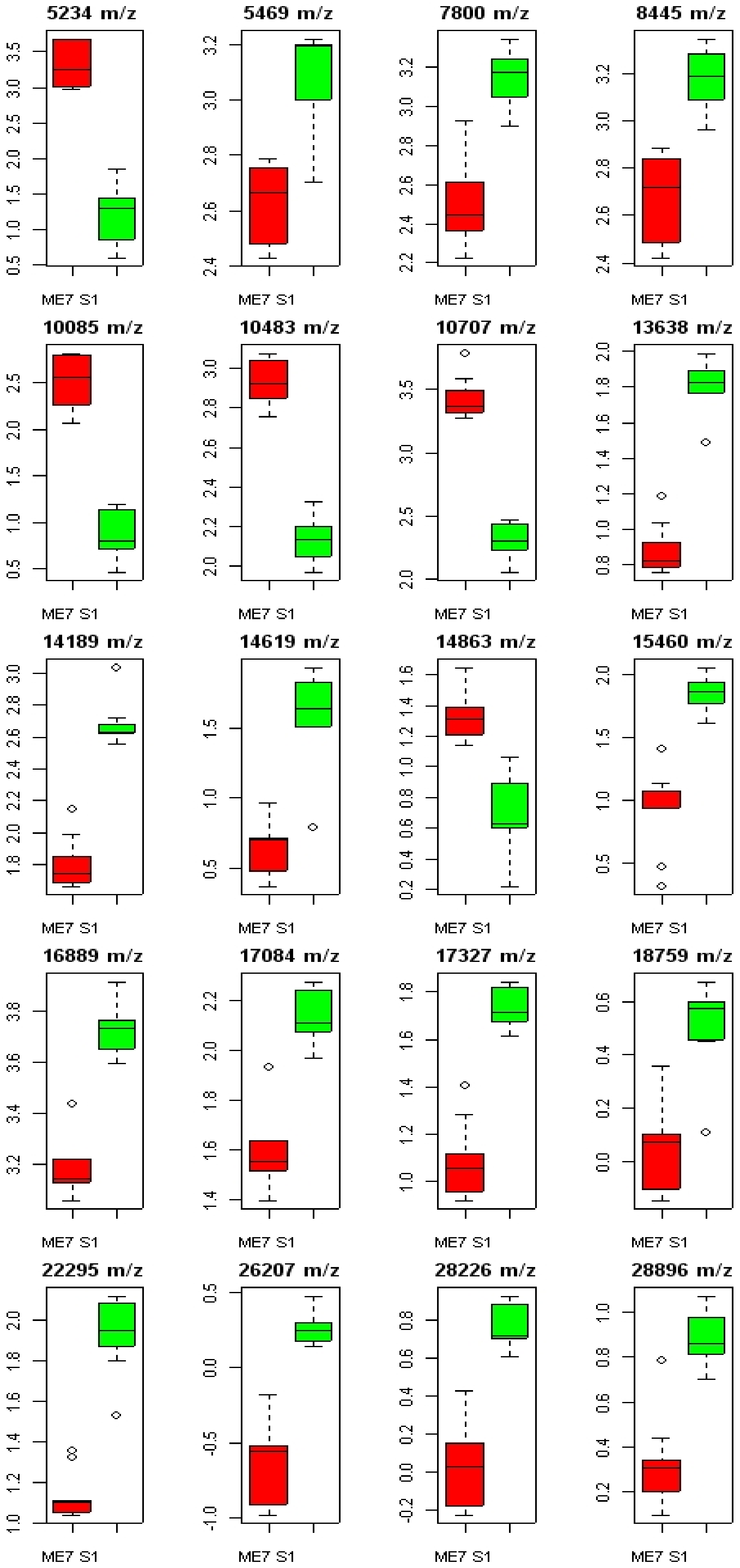

Boxplot of significant proteins
