# Supplementary material for: Differential protein profiling as a potential multi-marker approach for TSE diagnosis
Source: BMC Infect Dis. 2009 Nov 27;9:188. doi: 10.1186/1471-2334-9-188 (PMC2794872; doi:10.1186/1471-2334-9-188)

S2 CM10 30

Proteins showing total separation

No proteins showed complete separation

Significant data (p<=0.05)

Significant Proteins (t-test; p<=0.05)

	name	mz	ME7.avg	NORM.avg	t	p
19	C018505_	18505	2.695	2.25	2.5	0.0263
27	C028118_	28118	1.697	1.16	2.6	0.0229
28	C032813_	32813	0.375	-0.26	2.9	0.0138
29	C036515_	36515	0.034	-0.34	2.5	0.0315
31	C042278_	42278	0.388	-0.25	3.2	0.0089
32	C051238_	51238	-0.346	-0.92	2.4	0.0386

Data for Significant proteins

	C0GROUP	C0GRP_NA	C0Spectr	C018505_	C028118_	C032813_	C036515_	C042278_	C051238_
5	0	ME7S2	B32943	3.1	2.3	1.104	0.5790	1.1295	0.482
6	0	ME7S2	B32944	2.8	1.7	0.427	0.1253	0.4319	-0.031
7	0	ME7S2	B32945	2.3	1.2	-0.056	-0.1841	0.2201	0.079
11	0	ME7S2	B32943	2.6	1.6	0.233	-0.0049	0.2374	-0.773
12	0	ME7S2	B32946	2.5	1.3	-0.173	-0.4634	-0.2894	-0.977
13	0	ME7S2	B32947	3.0	2.1	0.841	0.2765	0.6864	-0.391
14	0	ME7S2	B32948	2.6	1.6	0.246	-0.0870	0.2999	-0.810
1	1	Normal S2	B32937	2.3	1.4	-0.122	-0.2178	-0.0733	-0.774
2	1	Normal S2	B32938	2.4	1.5	-0.043	-0.1029	-0.0052	-0.501
3	1	Normal S2	B32939	2.4	1.3	-0.064	-0.4409	-0.1933	-0.849
4	1	Normal S2	B32940	1.5	0.4	-0.998	-0.6636	-0.8344	-1.202
8	1	Normal S2	B32939	2.2	1.1	-0.288	-0.5656	-0.2586	-1.472
9	1	Normal S2	B32941	2.5	1.3	-0.124	-0.2073	-0.2130	-1.004
10	1	Normal S2	B32942	2.4	1.3	-0.155	-0.1875	-0.1380	-0.631

Boxplot of significant proteins

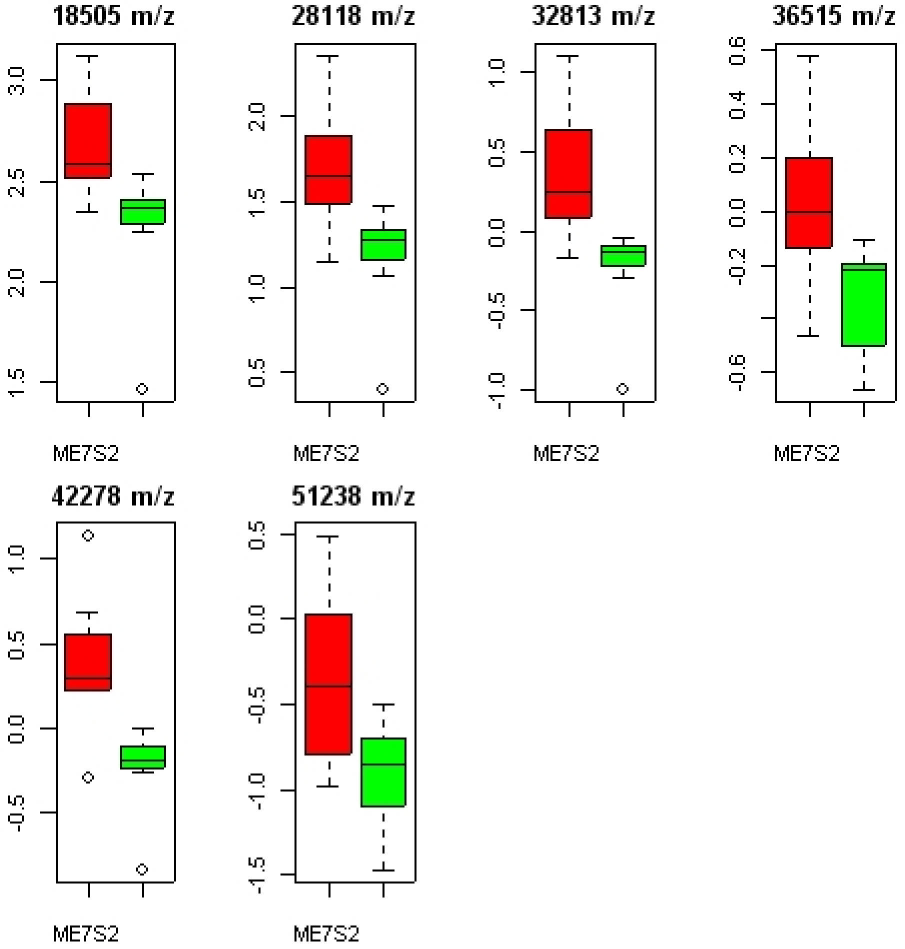

Boxplot of significant proteins

Pairwise Scatterplots of Significant Proteins

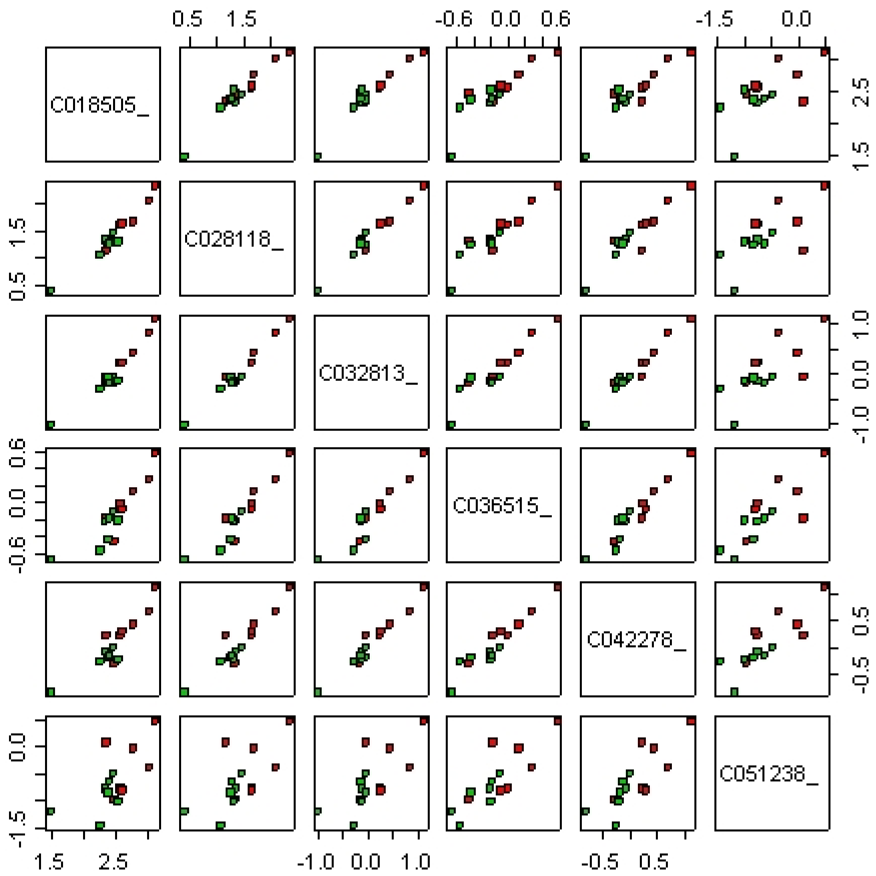

Pairwise Scatterplots of Significant Proteins

Cluster Analysis of samples (Euclidean distance)

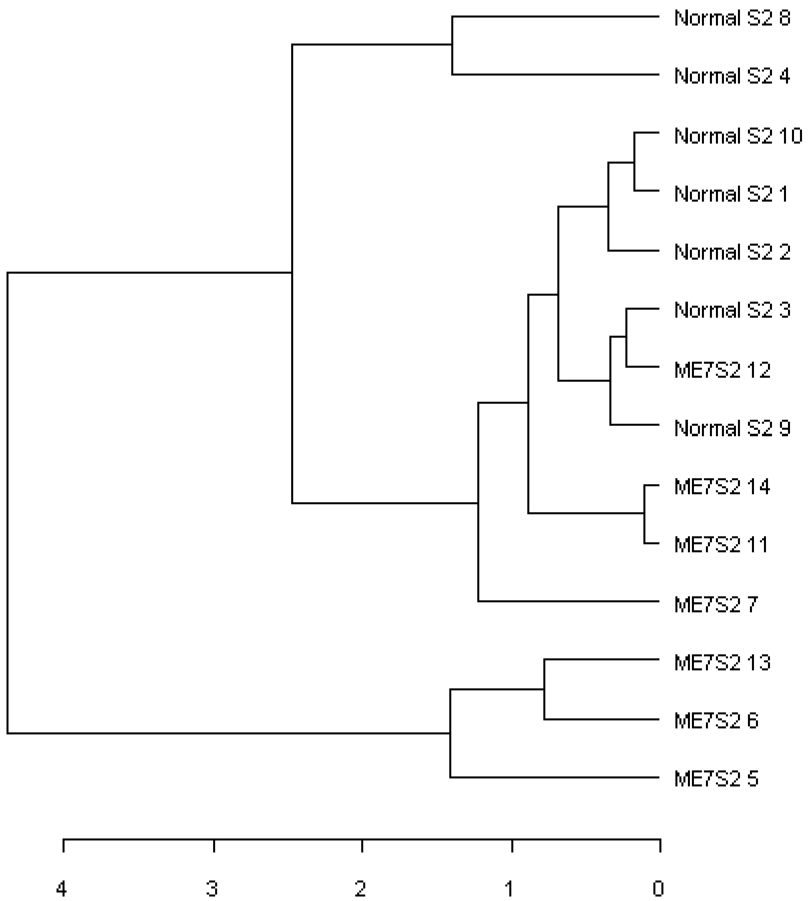

Cluster Analysis of samples (Euclidean distance)

Plot of first three principal components

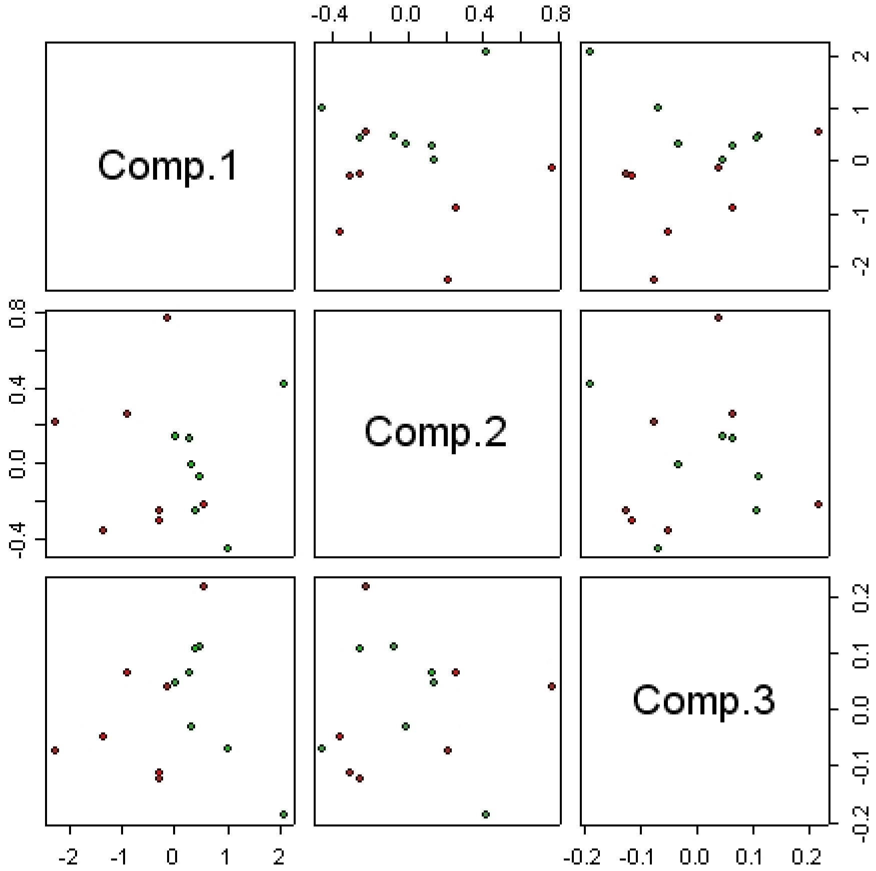

Plot of first three principal components

Scatterplot of linear discriminant function (x-axis)

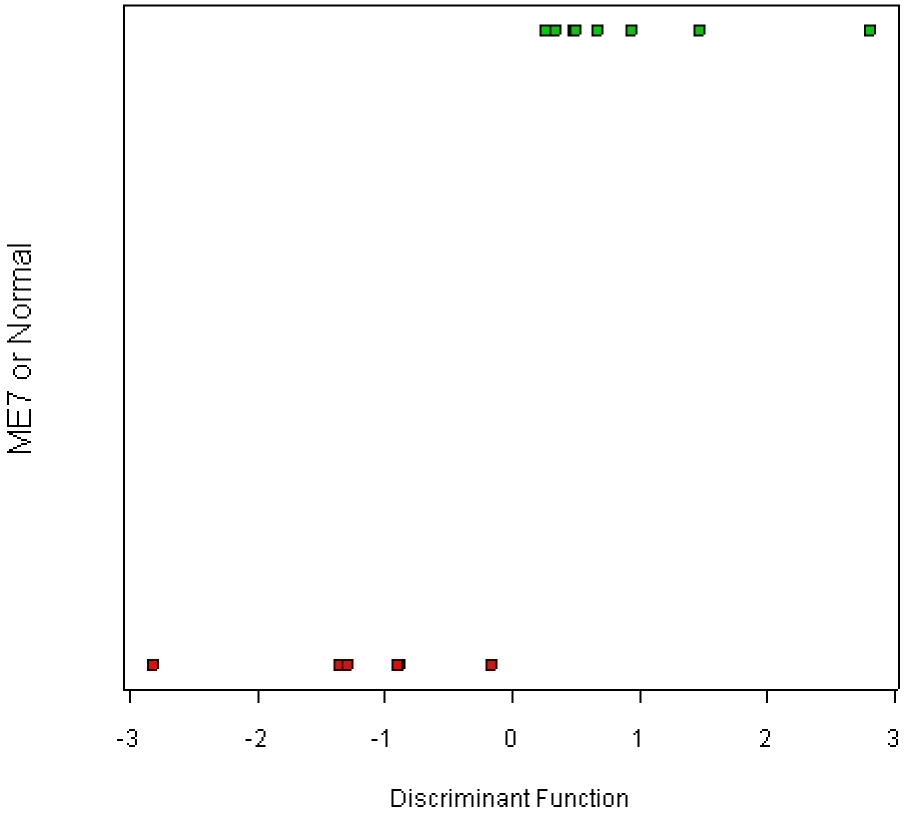

Scatterplot of linear discriminant function (x-axis)

All data

Boxplot of all proteins

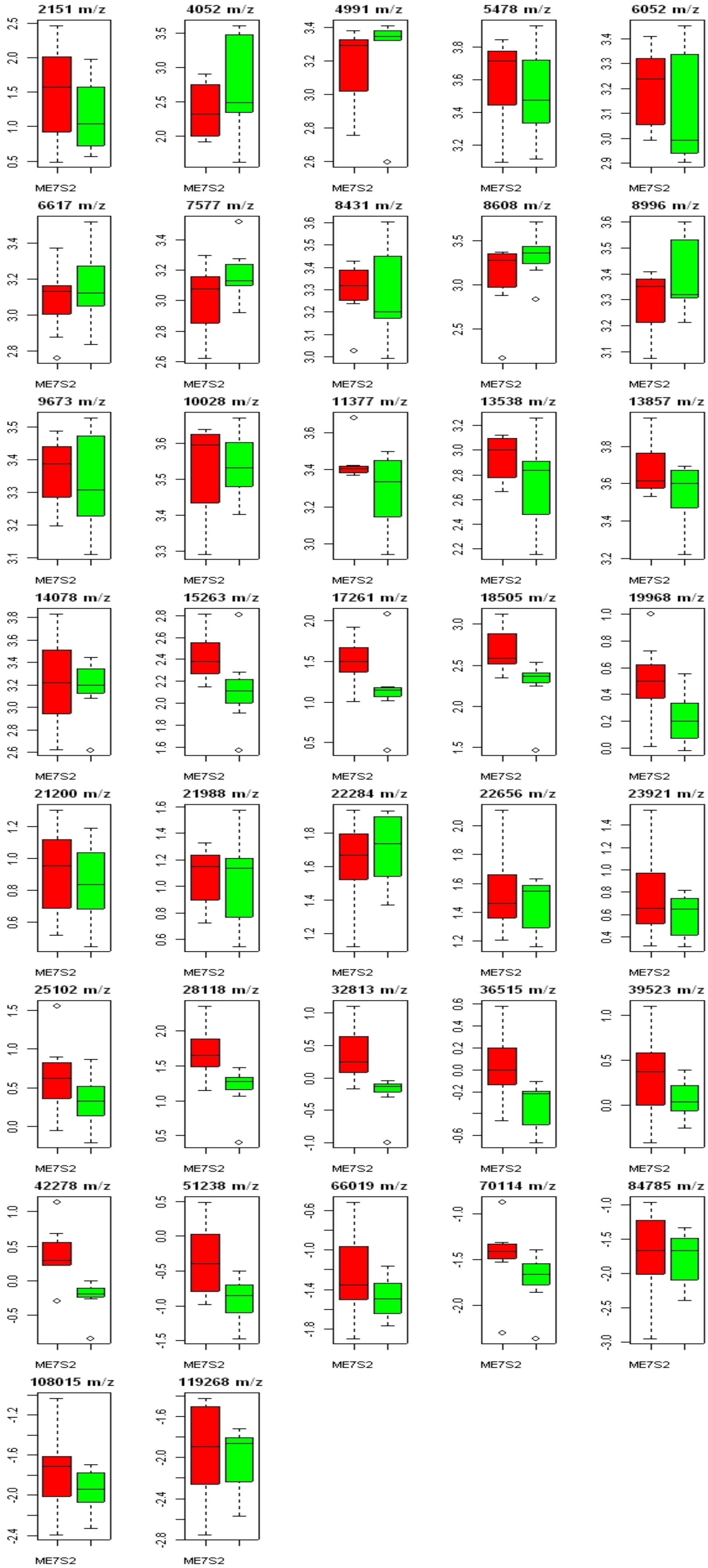

Boxplot of all proteins

Cluster Analysis of samples (Euclidean distance)

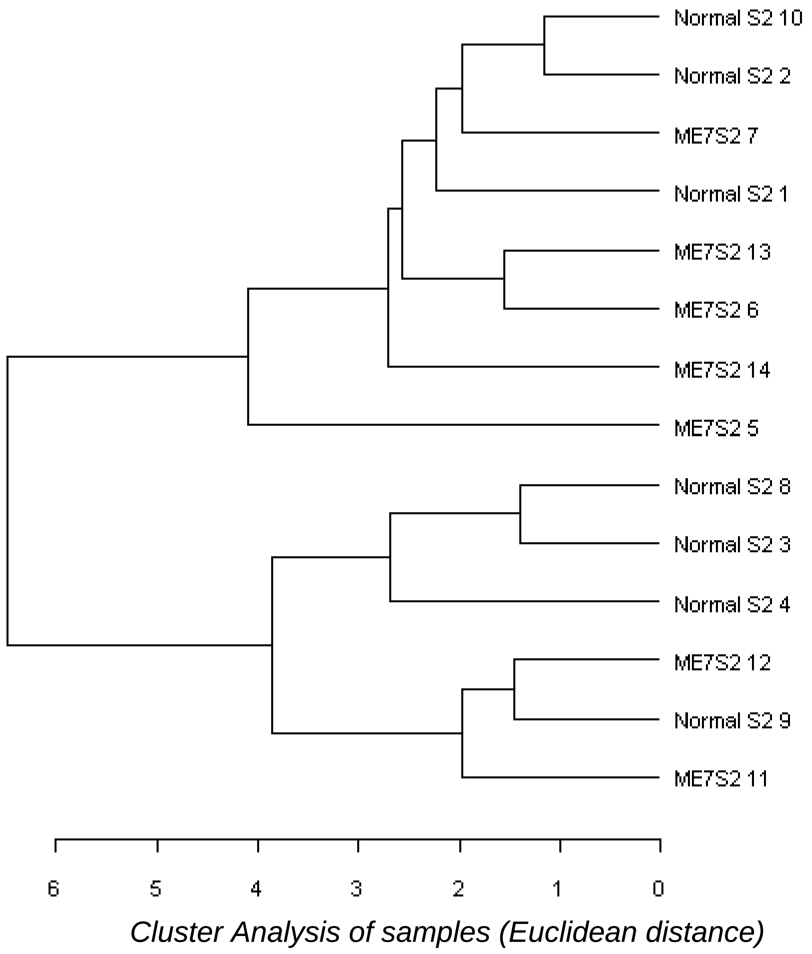

S2 CM10 60

Proteins showing total separation

No proteins showed complete separation

Significant data (p<=0.05)

No Significant Proteins

Data for Significant proteins

	C0GROUP	C0GRP_NA	C0Spectr
5	0	ME7 S2	B33451
6	0	ME7 S2	B33452
7	0	ME7 S2	B33453
12	0	ME7 S2	B33454
13	0	ME7 S2	B33455
14	0	ME7 S2	B33456
1	1	Normal S2	B33445
2	1	Normal S2	B33446
3	1	Normal S2	B33447
4	1	Normal S2	B33449
8	1	Normal S2	B33447
9	1	Normal S2	B33448
10	1	Normal S2	B33449
11	1	Normal S2	B33450

All data

Boxplot of all proteins

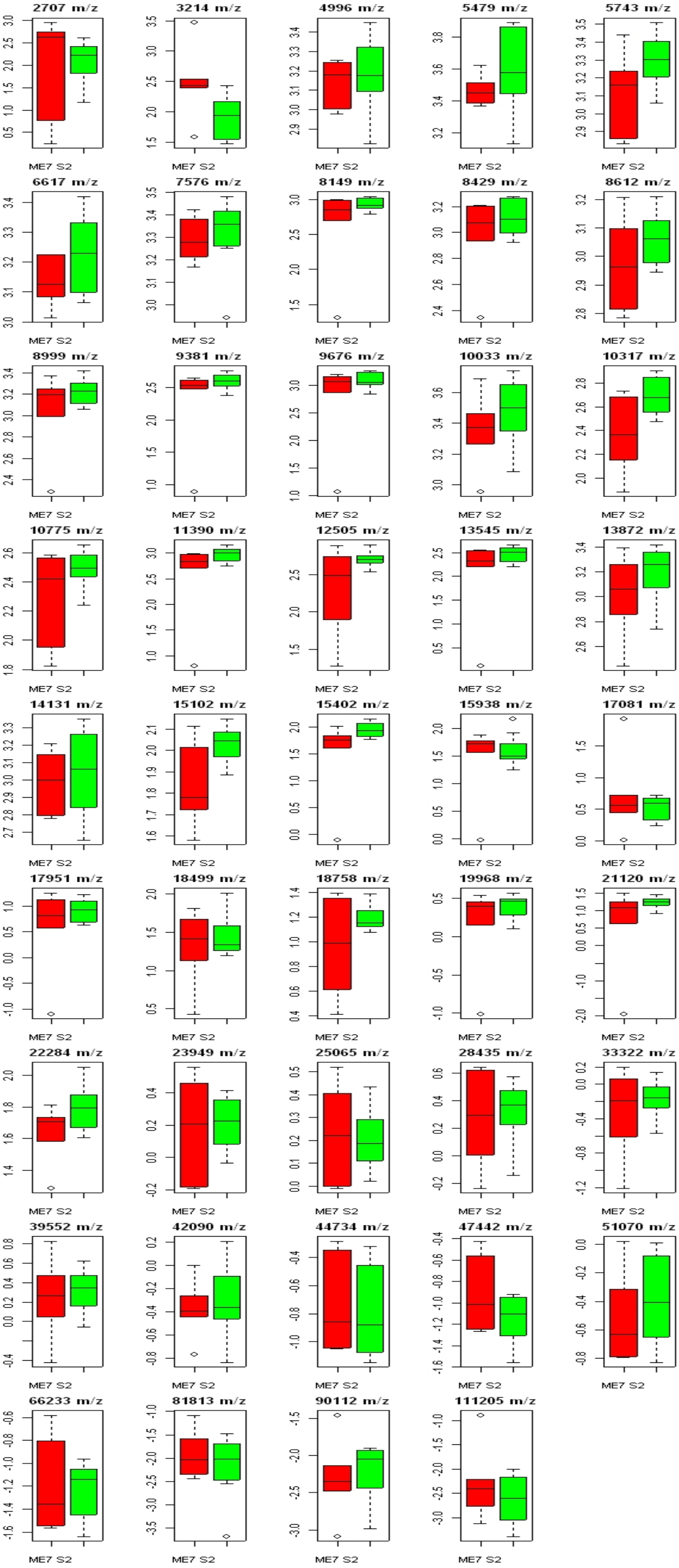

Boxplot of all proteins

Cluster Analysis of samples (Euclidean distance)

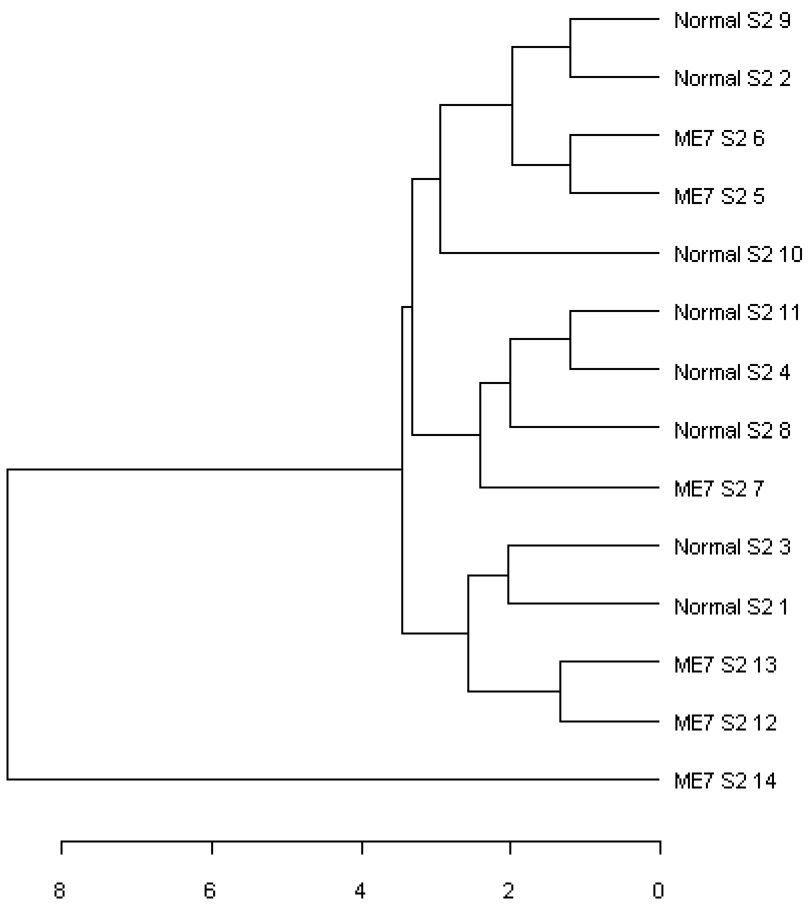

Cluster Analysis of samples (Euclidean distance)

S2 CM10 90

Proteins showing total separation

No proteins showed complete separation

Significant data (p<=0.05)

Significant Proteins (t-test; p<=0.05)

	name	mz	ME7.avg	NORM.avg	t	p
13	C012508_	12508	3.37	3.6	-2.7	0.022
24	C028163_	28163	0.88	1.4	-3.1	0.012
33	C083532_	83532	-2.15	-2.4	2.7	0.020

Data for Significant proteins

	C0GROUP	C0GRP_NA	C0Spectr	C012508_	C028163_	C083532_
4	0	ME7S2	B34041	3.3	0.64	-2.4
5	0	ME7S2	B34045	3.4	1.00	-2.1
6	0	ME7S2	B34046	3.4	0.96	-2.1
7	0	ME7S2	B34047	3.5	1.35	-2.1
11	0	ME7S2	B34041	3.4	0.73	-1.8
12	0	ME7S2	B34043	2.9	0.60	-2.6
13	0	ME7S2	B34044	3.5	0.79	-2.1
14	0	ME7S2	B34045	3.6	0.99	-2.1
1	1	Normal S2	B34036	3.6	1.03	-2.1
2	1	Normal S2	B34039	3.5	1.09	-2.6
3	1	Normal S2	B34040	3.4	1.24	-2.5
8	1	Normal S2	B34033	3.7	1.69	-2.5
9	1	Normal S2	B34034	3.7	1.33	-2.3
10	1	Normal S2	B34035	3.7	1.81	-2.5

Boxplot of significant proteins

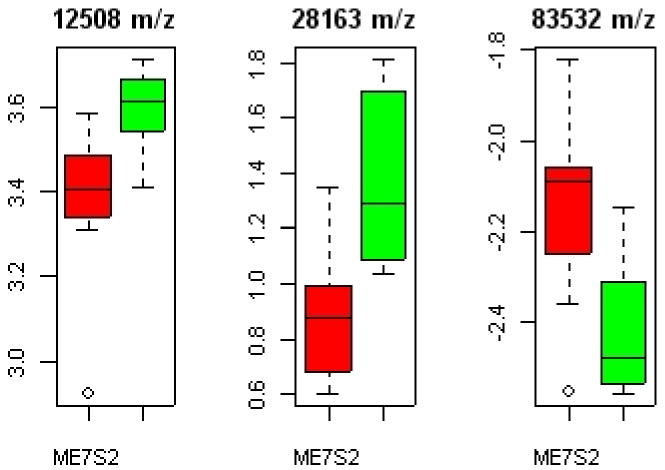

Boxplot of significant proteins

Pairwise Scatterplots of Significant Proteins

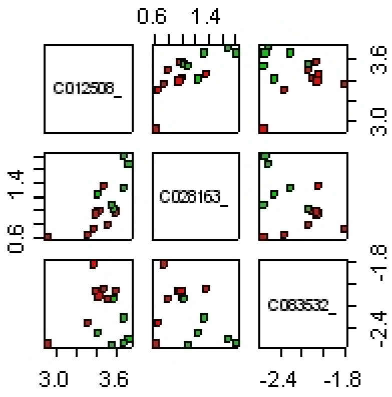

Pairwise Scatterplots of Significant Proteins

Cluster Analysis of samples (Euclidean distance)

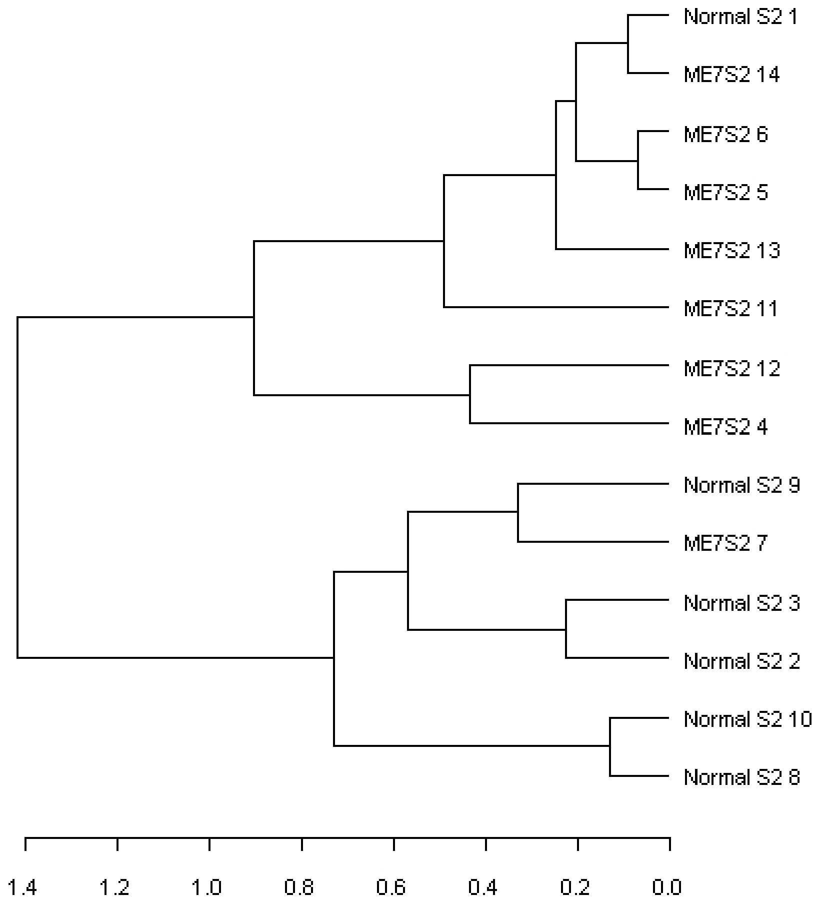

Cluster Analysis of samples (Euclidean distance)

Scatterplot of linear discriminant function (x-axis)

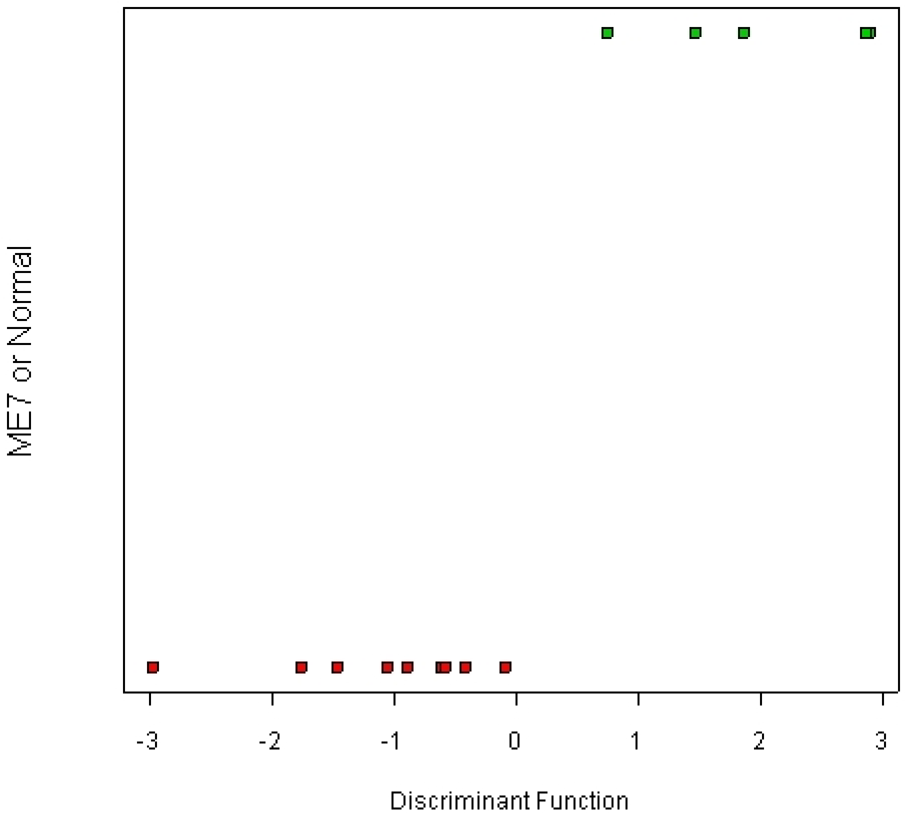

Scatterplot of linear discriminant function (x-axis)

All data

Boxplot of all proteins

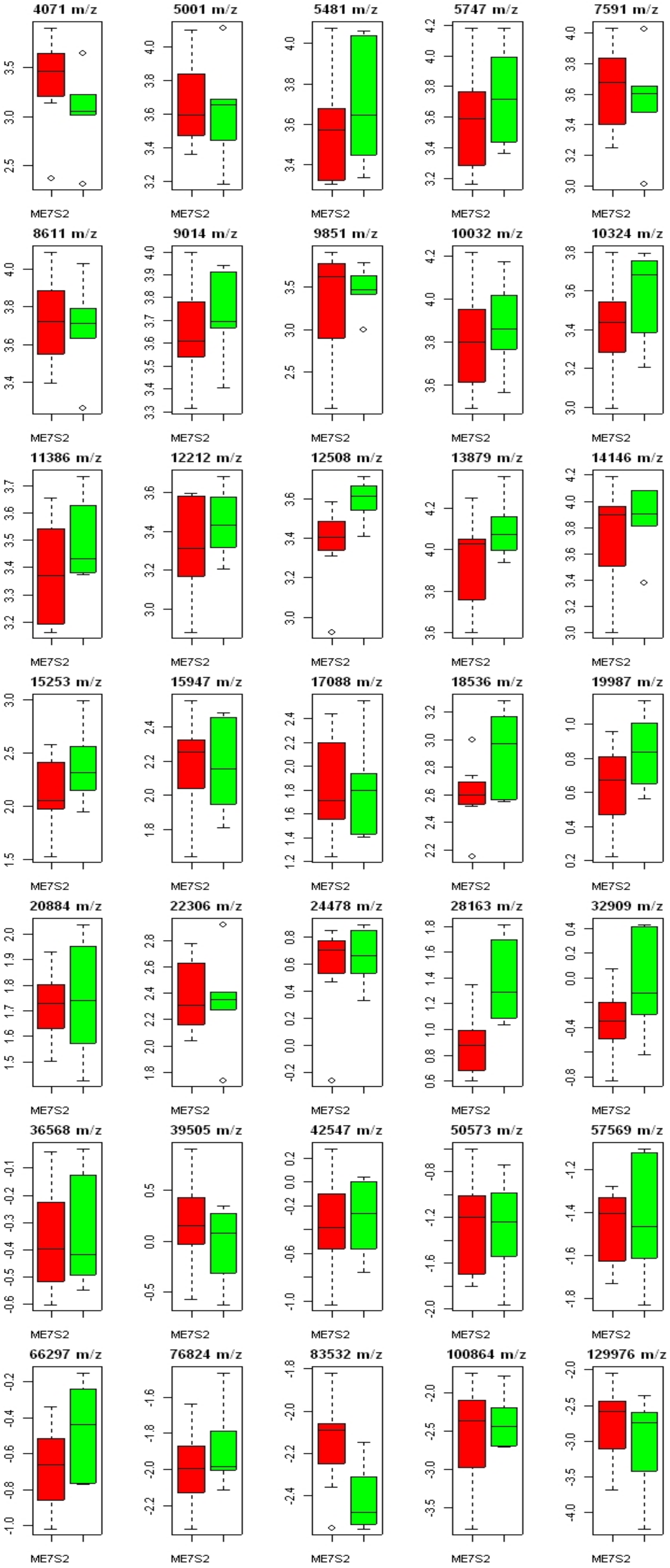

Boxplot of all proteins

Cluster Analysis of samples (Euclidean distance)

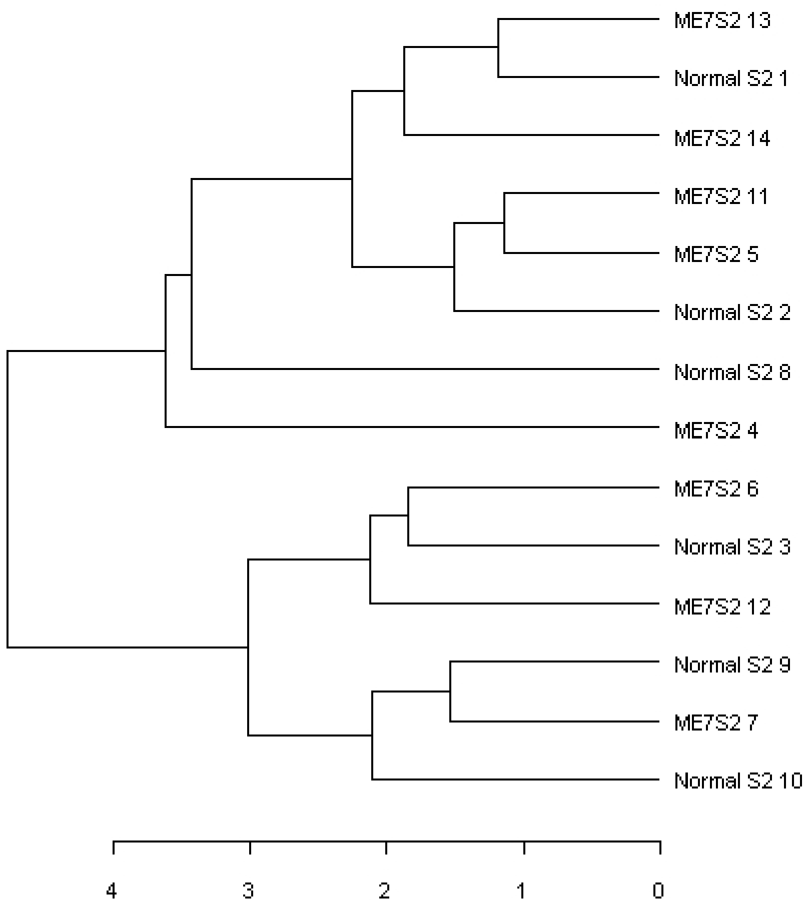

Cluster Analysis of samples (Euclidean distance)

S2 CM10 120

Proteins showing total separation

No proteins showed complete separation

Significant data (p<=0.05)

No Significant Proteins

Data for Significant proteins

	C0GROUP	C0GRP_NA	C0Spectr
5	0	ME7S2	B34617
6	0	ME7S2	B34618
7	0	ME7S2	B34619
11	0	ME7S2	B34615
12	0	ME7S2	B34616
13	0	ME7S2	B34620
1	1	Normal S2	B34609
2	1	Normal S2	B34610
3	1	Normal S2	B34611
4	1	Normal S2	B34612
8	1	Normal S2	B34612
9	1	Normal S2	B34613
10	1	Normal S2	B34614

All data

Boxplot of all proteins

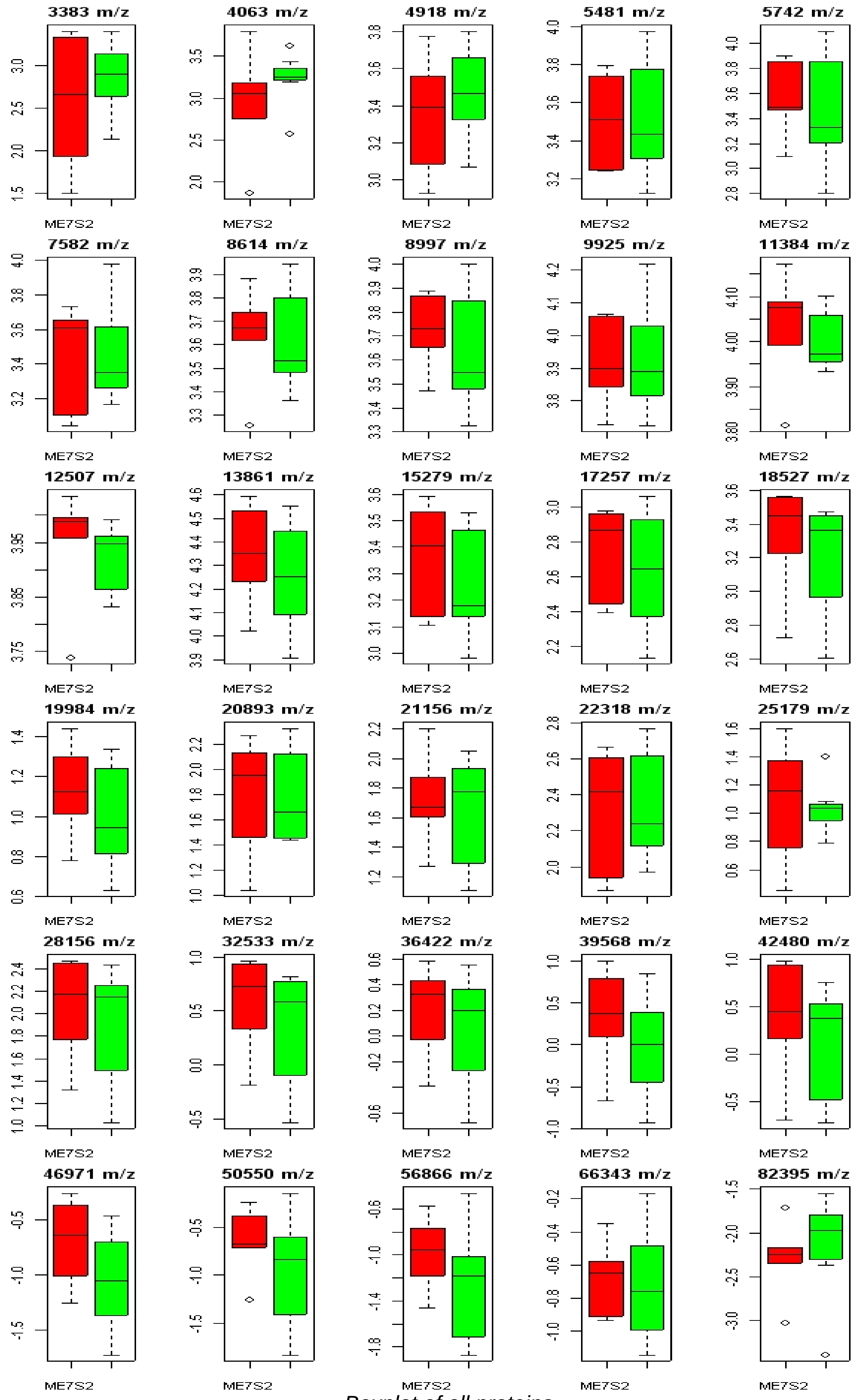

Boxplot of all proteins

Cluster Analysis of samples (Euclidean distance)

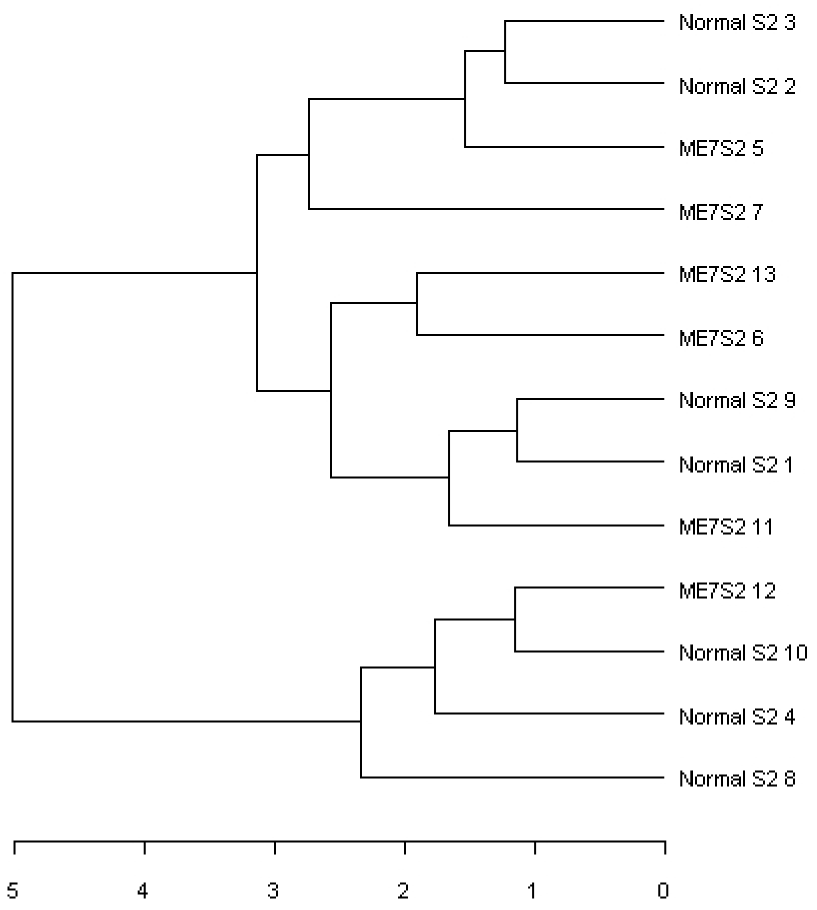

Cluster Analysis of samples (Euclidean distance)

S2 CM10 150

Proteins showing total separation

No proteins showed complete separation

Significant data (p<=0.05)

No Significant Proteins

Data for Significant proteins

	C0GROUP	C0GRP_NA	C0Spectr
4	0	ME7S2	B35179
5	0	ME7S2	B35180
6	0	ME7S2	B35181
7	0	ME7S2	B35182
12	0	ME7S2	B35183
13	0	ME7S2	B35184
14	0	ME7S2	B35185
1	1	Normal S2	B35176
2	1	Normal S2	B35177
3	1	Normal S2	B35178
8	1	Normal S2	B35173
9	1	Normal S2	B35174
10	1	Normal S2	B35175
11	1	Normal S2	B35177

All data

Boxplot of all proteins

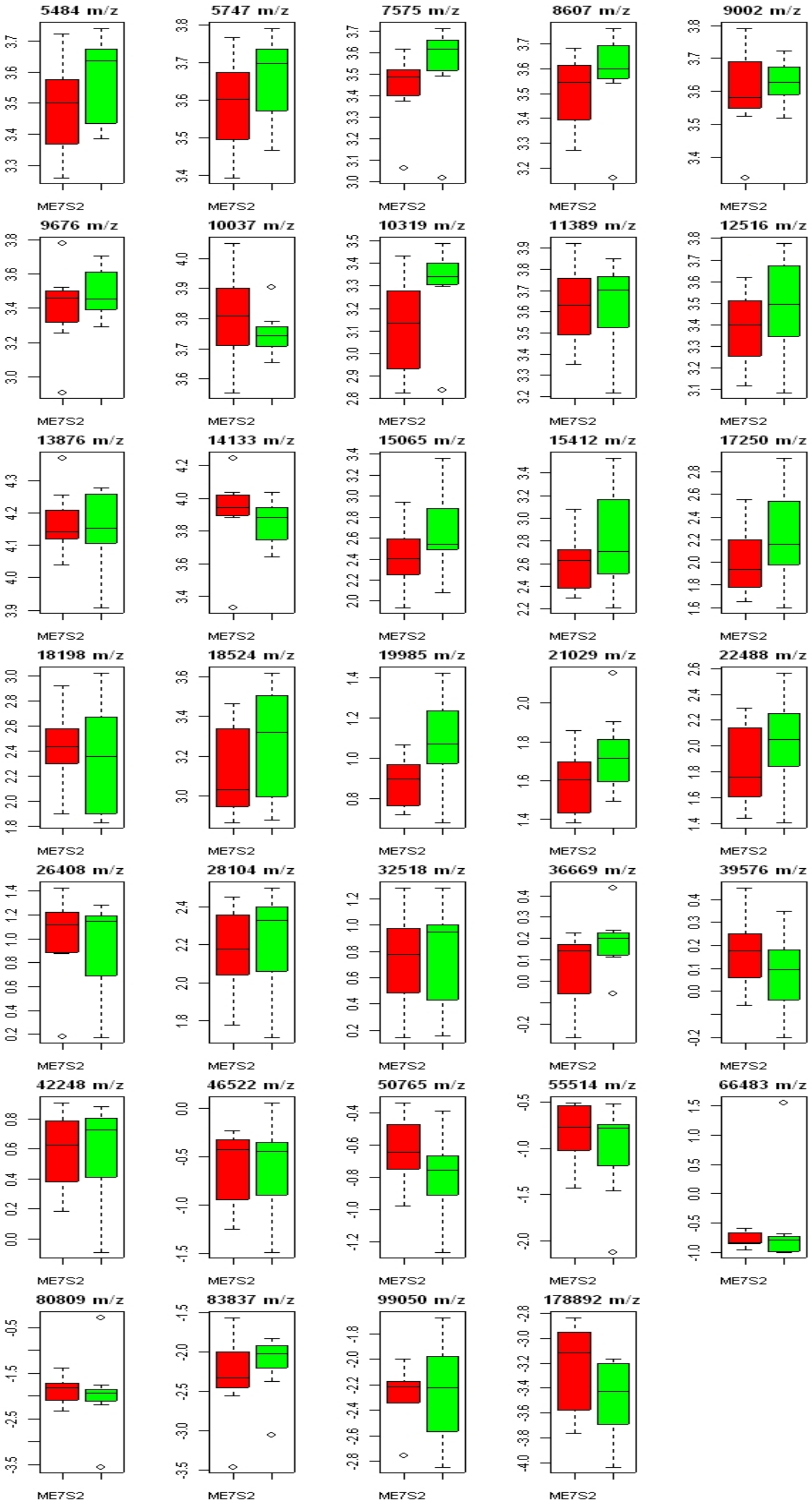

Boxplot of all proteins

Cluster Analysis of samples (Euclidean distance)

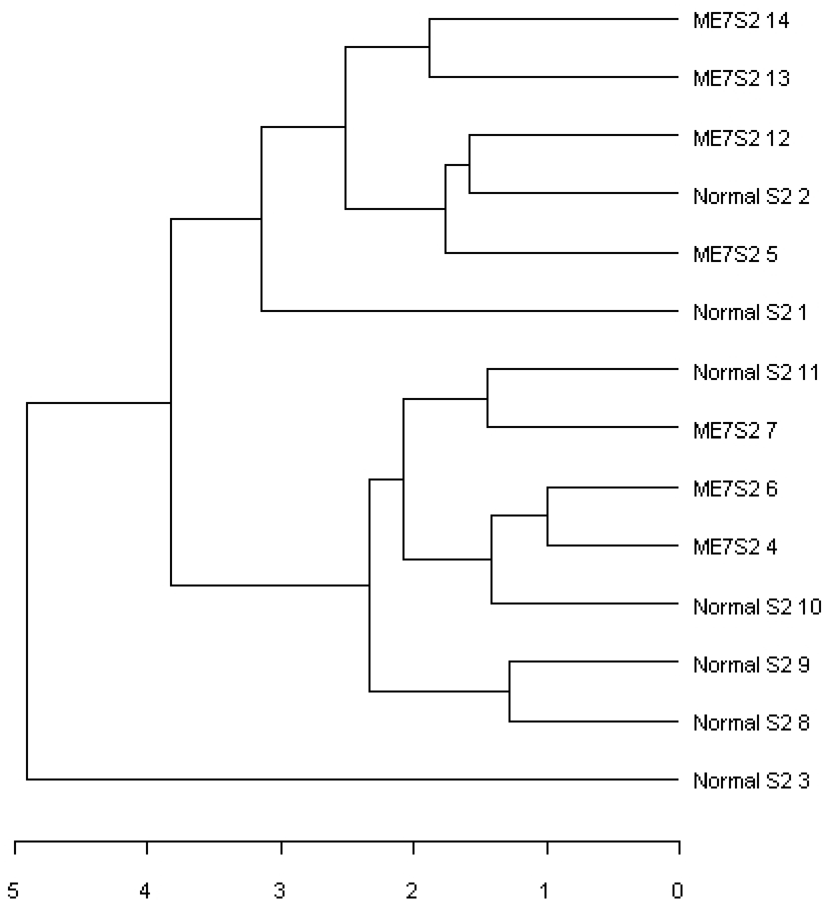

Cluster Analysis of samples (Euclidean distance)

S2 CM10 180

Proteins showing total separation

	C0GROUP	C0GRP_NA	C0Spectr	C010325_
5	0	ME7S2	B35772	2.9
6	0	ME7S2	B35773	3.2
10	0	ME7S2	B35770	2.8
11	0	ME7S2	B35771	2.8
12	0	ME7S2	B35774	3.1
13	0	ME7S2	B35775	2.9
1	1	Normal S2	B35764	3.5
2	1	Normal S2	B35765	3.3
3	1	Normal S2	B35767	3.4
4	1	Normal S2	B35768	3.3
7	1	Normal S2	B35766	3.4
8	1	Normal S2	B35767	3.5
9	1	Normal S2	B35769	3.2

Boxplot of proteins showing complete separation

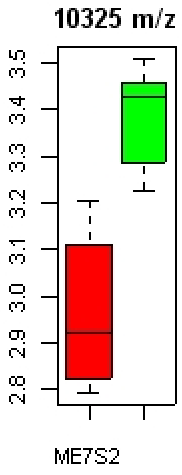

Significant data (p<=0.05)

Significant Proteins (t-test; p<=0.05)

	name	mz	ME7.avg	NORM.avg	t	p
1	C03376_7	3376	2.91105	2.02	3.3	0.0119
3	C05749_5	5749	3.39576	3.61	-2.6	0.0255
9	C09862_3	9862	3.42409	2.77	2.3	0.0494
11	C010325_	10325	2.96222	3.38	-5.3	0.0006
13	C012514_	12514	3.26848	3.51	-2.3	0.0444
17	C017266_	17266	1.54981	1.99	-3.1	0.0135
19	C018528_	18528	2.61744	3.21	-3.9	0.0044
22	C022172_	22172	1.87139	2.16	-2.9	0.0171
26	C032517_	32517	0.03955	0.62	-3.0	0.0131
29	C039522_	39522	-0.11504	0.37	-3.2	0.0128
30	C042249_	42249	0.00036	0.45	-2.3	0.0390
33	C056855_	56855	-1.71664	-1.24	-3.1	0.0146

Data for Significant proteins

	C0GROUP	C0GRP_NA	C0Spectr	C03376_7	C05749_5	C09862_3	C010325_	C012514_	C017266_	C018528_	C022172_	C032517_	C039522_	C042249_	C056855_
5	0	ME7S2	B35772	2.9	3.3	3.0	2.9	3.2	1.2	2.2	1.8	-0.341	-0.248	-0.260	-1.80
6	0	ME7S2	B35773	2.5	3.5	3.3	3.2	3.5	1.5	2.5	1.9	-0.144	0.212	0.083	-1.46
10	0	ME7S2	B35770	3.0	3.4	3.5	2.8	3.0	1.3	2.3	1.7	-0.118	-0.484	-0.310	-1.72
11	0	ME7S2	B35771	2.9	3.2	3.4	2.8	3.3	1.6	2.8	1.7	0.279	-0.154	0.189	-1.65
12	0	ME7S2	B35774	3.1	3.5	3.7	3.1	3.3	2.0	3.0	2.0	0.407	0.340	0.455	-1.83
13	0	ME7S2	B35775	3.1	3.5	3.6	2.9	3.3	1.7	2.9	2.2	0.154	-0.357	-0.154	-1.84
1	1	Normal S2	B35764	1.8	3.8	2.5	3.5	3.4	1.9	3.2	2.3	0.850	0.490	0.560	-1.01
2	1	Normal S2	B35765	2.9	3.5	3.7	3.3	3.3	1.9	3.1	1.9	0.625	0.304	0.529	-1.43
3	1	Normal S2	B35767	1.3	3.3	2.9	3.4	3.7	2.4	3.3	2.1	0.750	0.484	0.626	-0.98
4	1	Normal S2	B35768	1.5	3.8	1.9	3.3	3.2	1.8	3.0	2.2	0.203	0.212	0.072	-1.54
7	1	Normal S2	B35766	2.3	3.7	2.2	3.4	3.7	2.0	3.5	2.3	1.119	0.559	0.879	-0.76
8	1	Normal S2	B35767	1.4	3.5	2.7	3.5	3.6	2.1	3.4	2.3	0.830	0.512	0.704	-1.15
9	1	Normal S2	B35769	2.8	3.6	3.6	3.2	3.6	1.8	2.9	2.1	-0.066	0.033	-0.238	-1.83

Boxplot of significant proteins

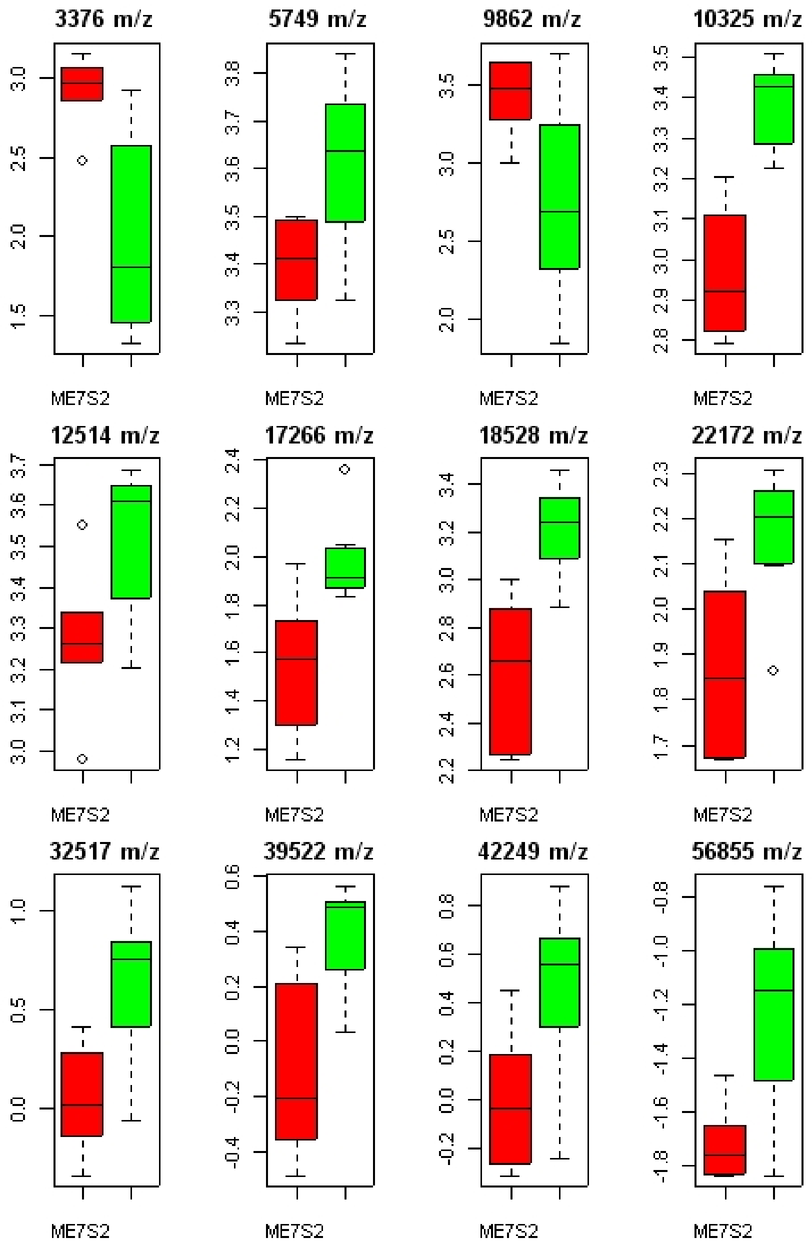

Boxplot of significant proteins

Pairwise Scatterplots of Significant Proteins

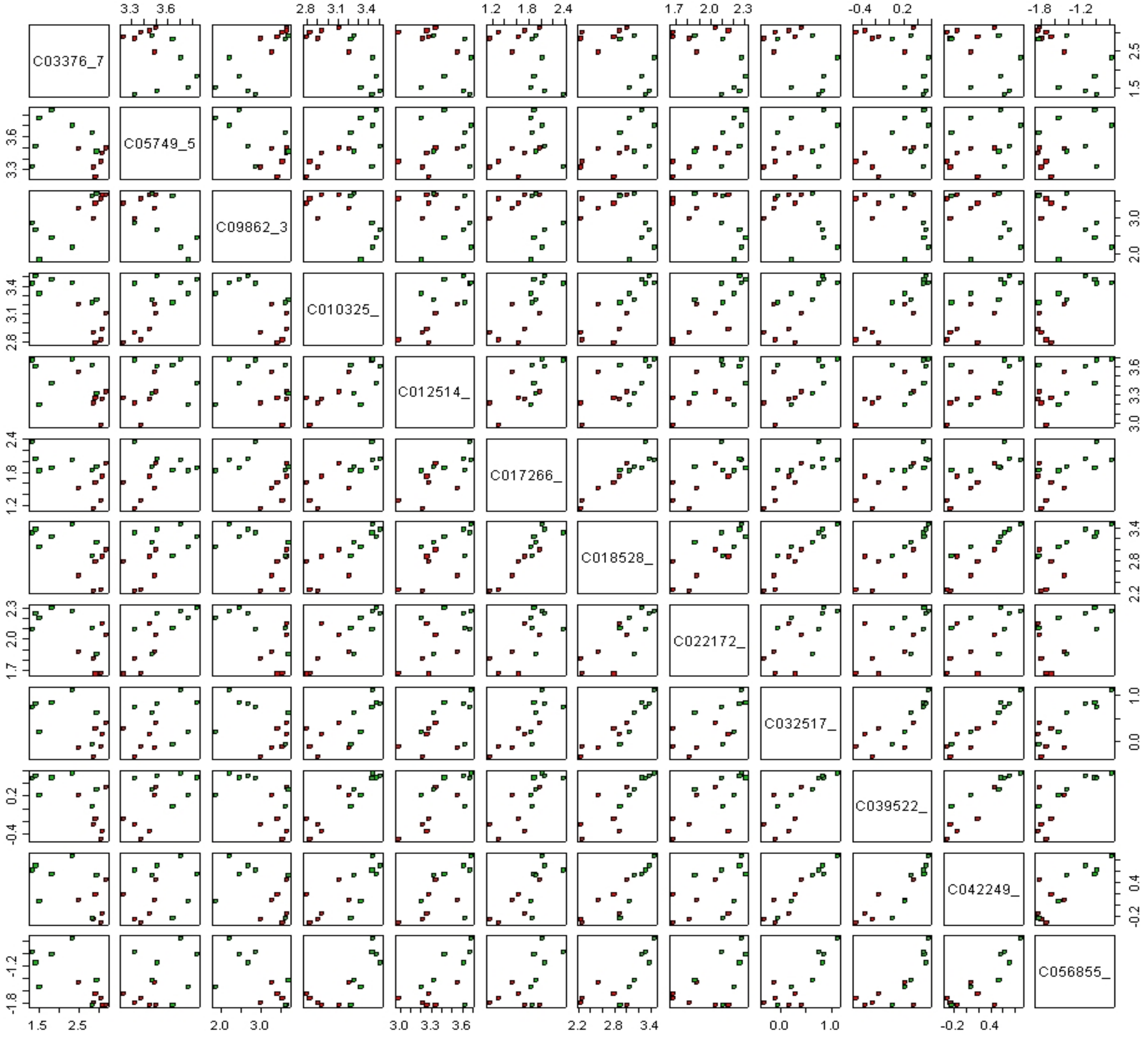

Pairwise Scatterplots of Significant Proteins

Cluster Analysis of samples (Euclidean distance)

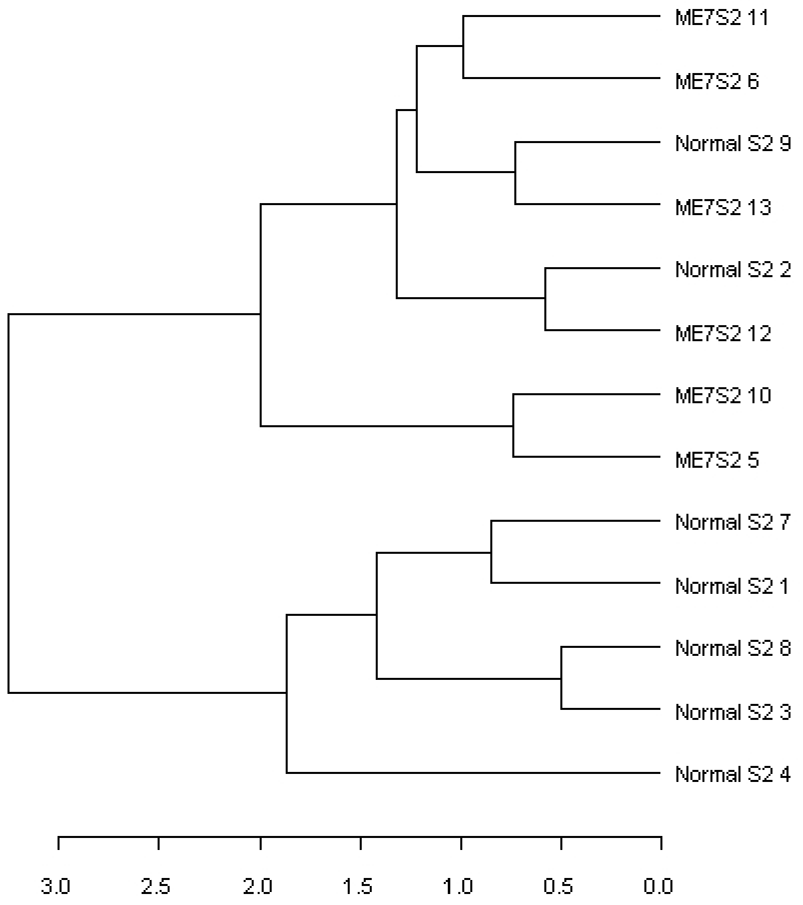

Cluster Analysis of samples (Euclidean distance)

Plot of first three principal components

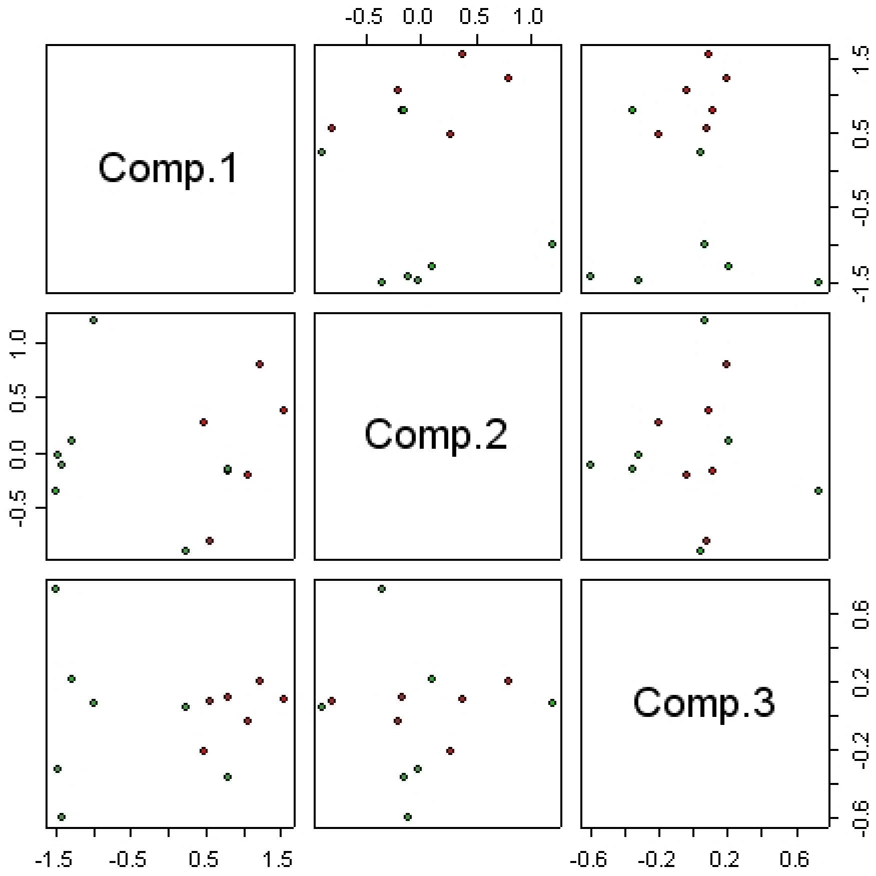

Plot of first three principal components

Scatterplot of linear discriminant function (x-axis)

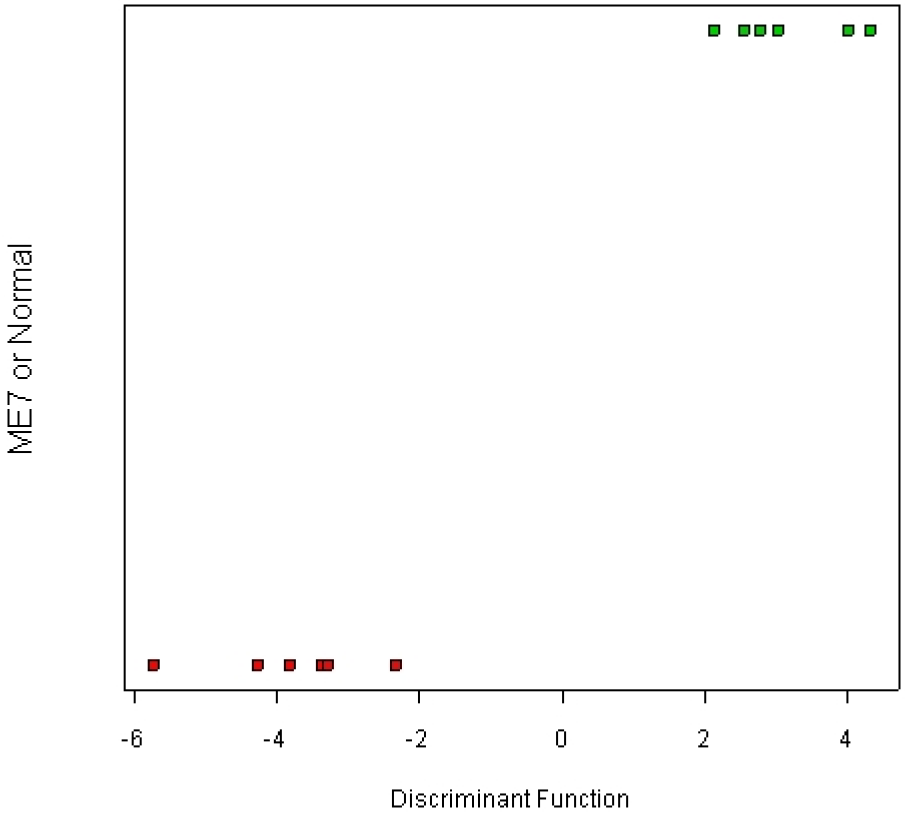

Scatterplot of linear discriminant function (x-axis)

All data

Boxplot of all proteins

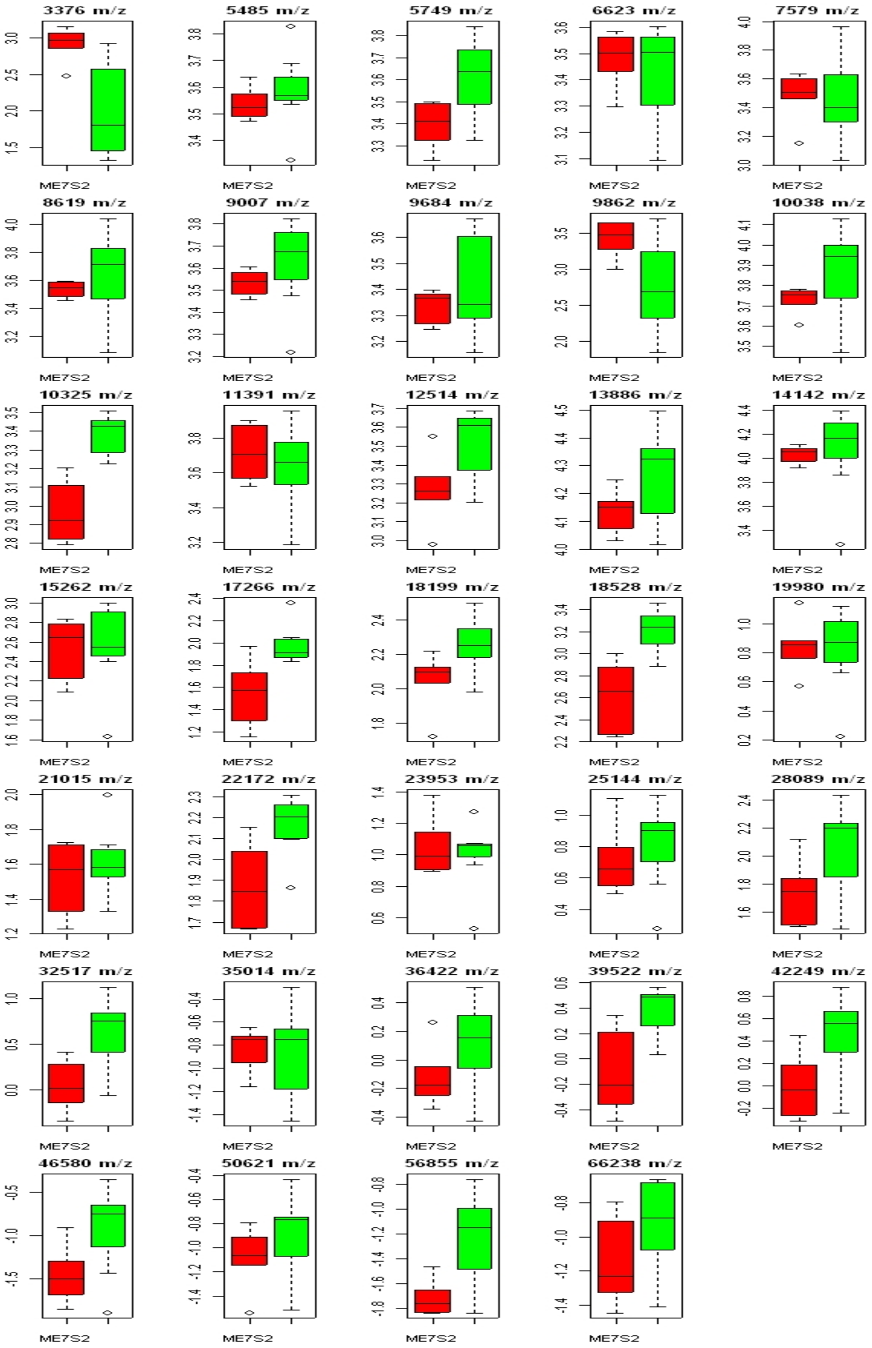

Boxplot of all proteins

Cluster Analysis of samples (Euclidean distance)

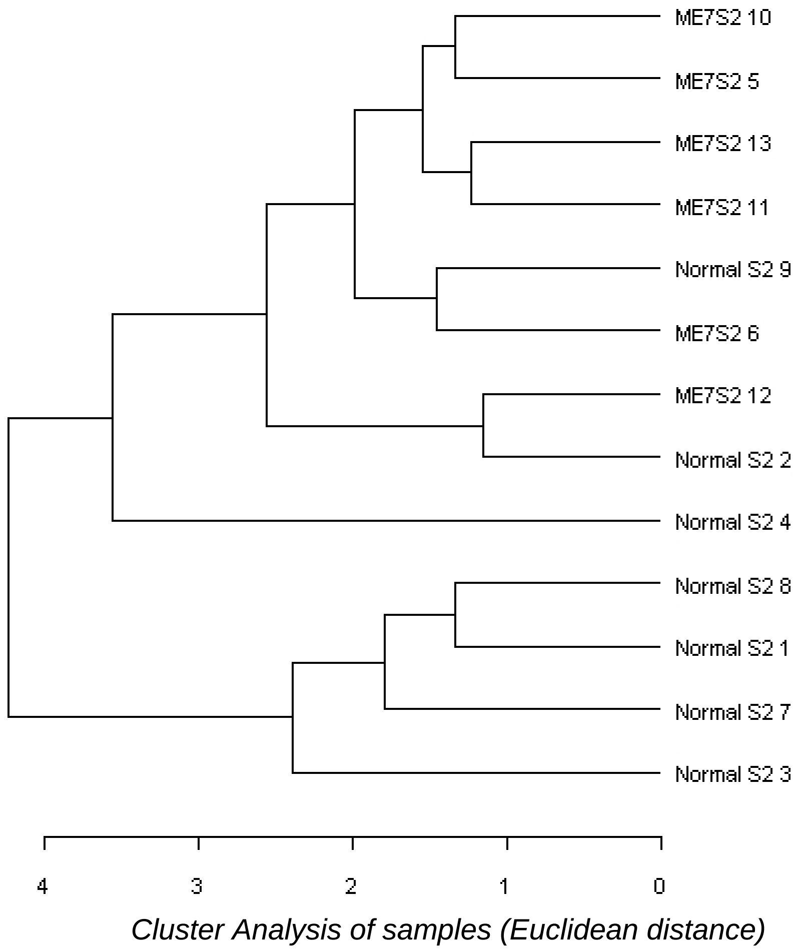

S2 CM10 210

Proteins showing total separation

	C0GROUP	C0GRP_NA	C0Spectr	C09023_0	C09973_5	C010322_	C022303_
5	0	ME7S2	B36260	3.5	3.7	2.9	1.6
6	0	ME7S2	B36261	3.5	3.7	2.6	1.4
7	0	ME7S2	B36265	3.5	3.9	2.8	1.4
8	0	ME7S2	B35770	3.3	3.6	2.8	1.1
12	0	ME7S2	B36262	3.4	3.9	2.8	1.6
13	0	ME7S2	B36263	3.4	3.9	2.7	1.5
14	0	ME7S2	B36264	3.5	3.7	2.8	1.5
1	1	Normal S2	B36248	3.6	3.0	3.0	1.9
2	1	Normal S2	B36250	3.7	3.3	3.0	2.1
3	1	Normal S2	B36252	4.0	3.4	3.4	2.3
4	1	Normal S2	B36253	3.7	3.4	3.4	2.0
9	1	Normal S2	B36249	3.6	3.4	3.4	2.0
10	1	Normal S2	B36251	3.7	3.2	3.1	1.8
11	1	Normal S2	B36253	3.6	3.2	3.0	1.7

Boxplot of proteins showing complete separation

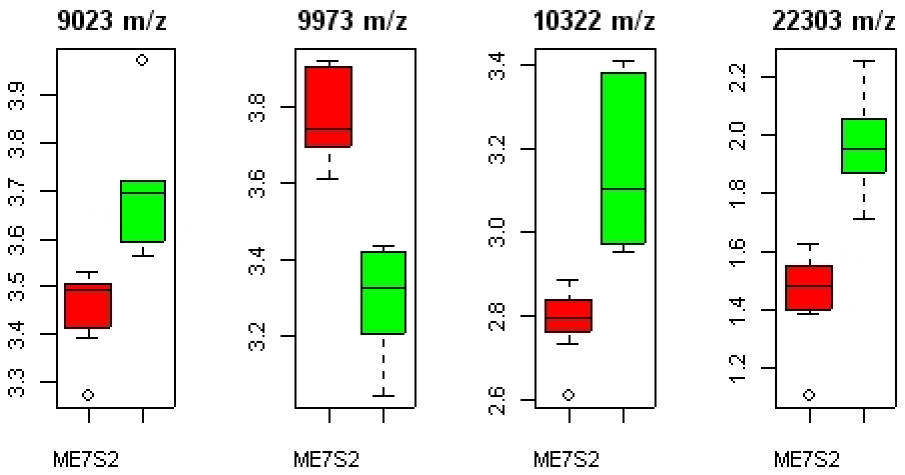

Pairwise Scatterplots of Proteins showing complete separation

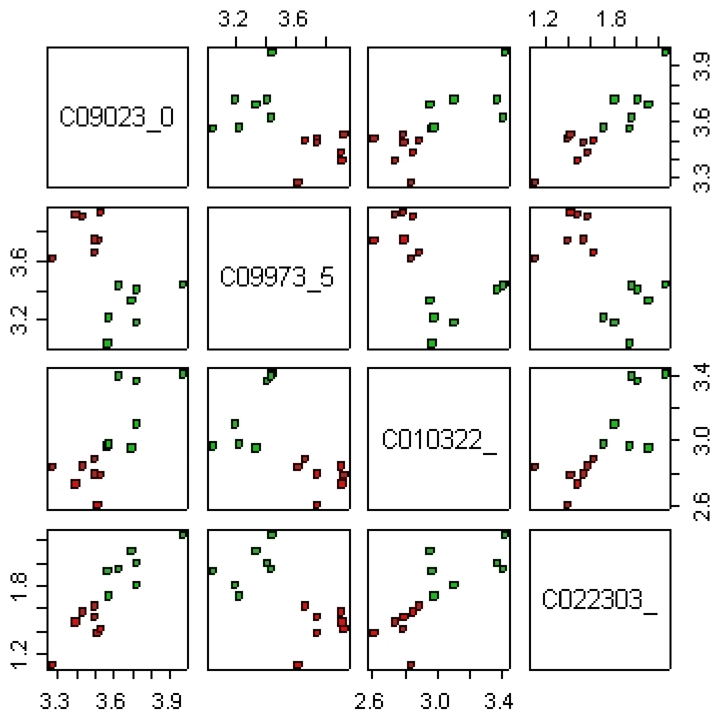

Significant data (p<=0.01)

Significant Proteins (t-test; p<=0.01)

	name	mz	ME7.avg	NORM.avg	t	p
7	C07615_9	7615	3.08	3.40	-3.9	3.9e-03
10	C09023_0	9023	3.45	3.69	-3.9	2.6e-03
12	C09973_5	9973	3.78	3.30	6.6	2.9e-05
14	C010322_	10322	2.79	3.17	-4.3	2.4e-03
27	C022303_	22303	1.44	1.97	-5.5	1.3e-04
33	C039526_	39526	-0.65	-0.14	-4.4	9.9e-04

Data for Significant proteins

	C0GROUP	C0GRP_NA	C0Spectr	C07615_9	C09023_0	C09973_5	C010322_	C022303_	C039526_
5	0	ME7S2	B36260	3.0	3.5	3.7	2.9	1.6	-0.41
6	0	ME7S2	B36261	3.0	3.5	3.7	2.6	1.4	-0.65
7	0	ME7S2	B36265	3.2	3.5	3.9	2.8	1.4	-0.57
8	0	ME7S2	B35770	3.2	3.3	3.6	2.8	1.1	-0.98
12	0	ME7S2	B36262	3.0	3.4	3.9	2.8	1.6	-0.32
13	0	ME7S2	B36263	3.0	3.4	3.9	2.7	1.5	-0.82
14	0	ME7S2	B36264	3.1	3.5	3.7	2.8	1.5	-0.84
1	1	Normal S2	B36248	3.2	3.6	3.0	3.0	1.9	-0.27
2	1	Normal S2	B36250	3.7	3.7	3.3	3.0	2.1	-0.26
3	1	Normal S2	B36252	3.5	4.0	3.4	3.4	2.3	-0.07
4	1	Normal S2	B36253	3.3	3.7	3.4	3.4	2.0	0.12
9	1	Normal S2	B36249	3.3	3.6	3.4	3.4	2.0	0.14
10	1	Normal S2	B36251	3.5	3.7	3.2	3.1	1.8	-0.24
11	1	Normal S2	B36253	3.3	3.6	3.2	3.0	1.7	-0.37

Boxplot of significant proteins

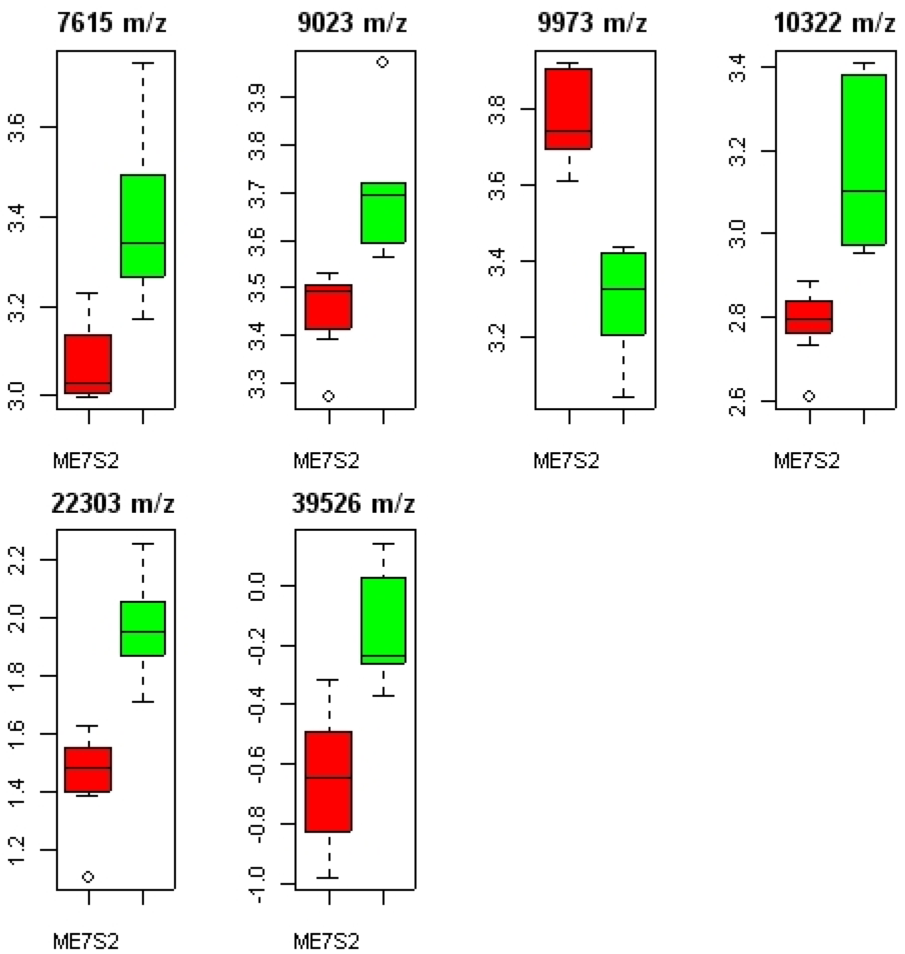

Boxplot of significant proteins

Pairwise Scatterplots of Significant Proteins

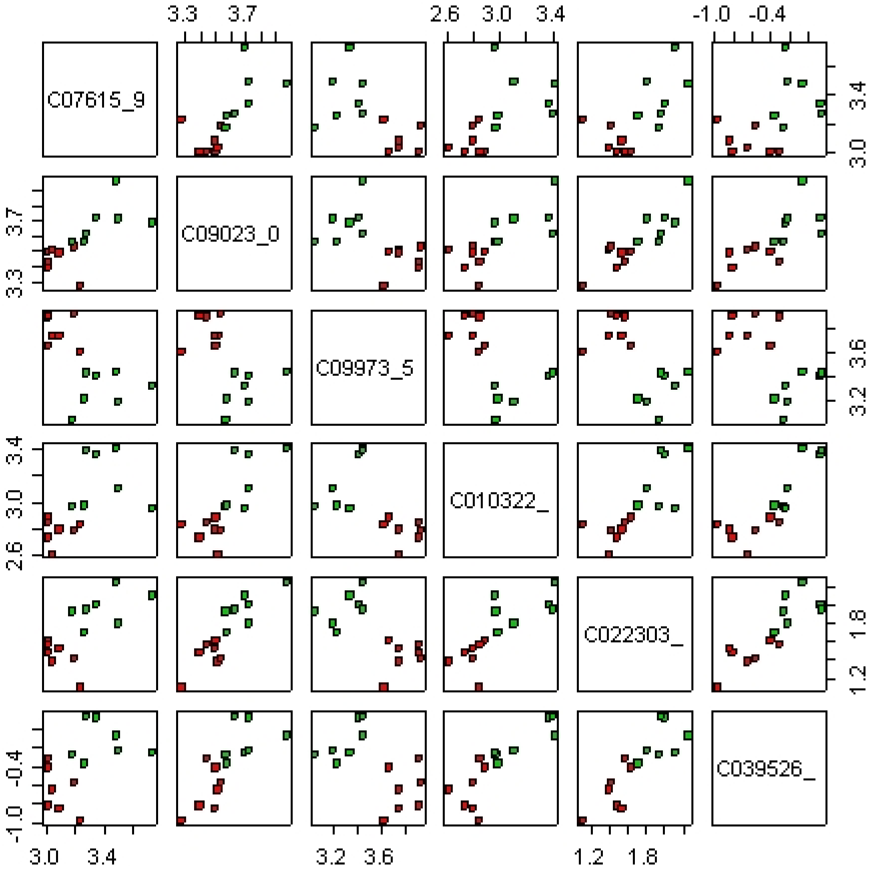

Pairwise Scatterplots of Significant Proteins

Cluster Analysis of samples (Euclidean distance)

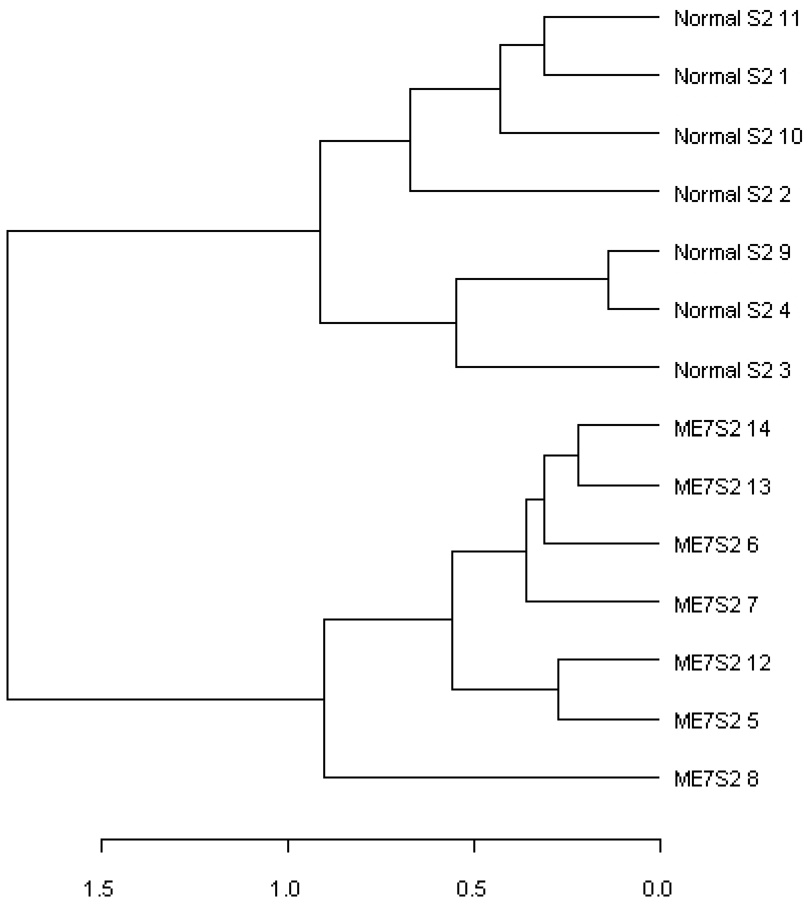

Cluster Analysis of samples (Euclidean distance)

Plot of first three principal components

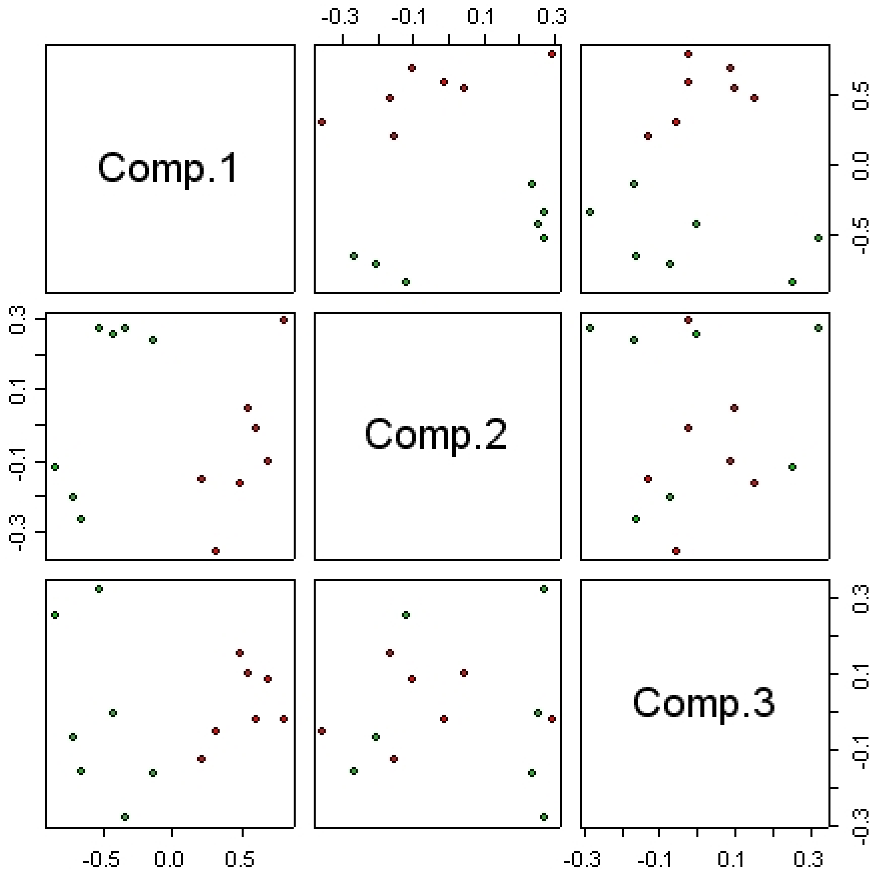

Plot of first three principal components

Scatterplot of linear discriminant function (x-axis)

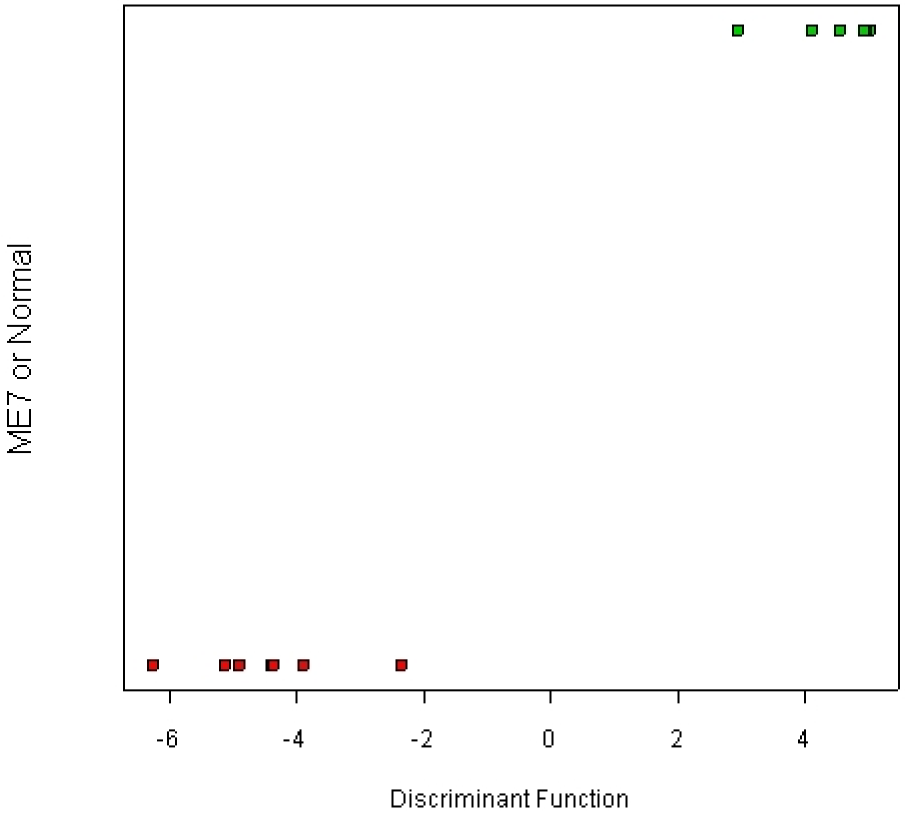

Scatterplot of linear discriminant function (x-axis)

All data

Boxplot of all proteins

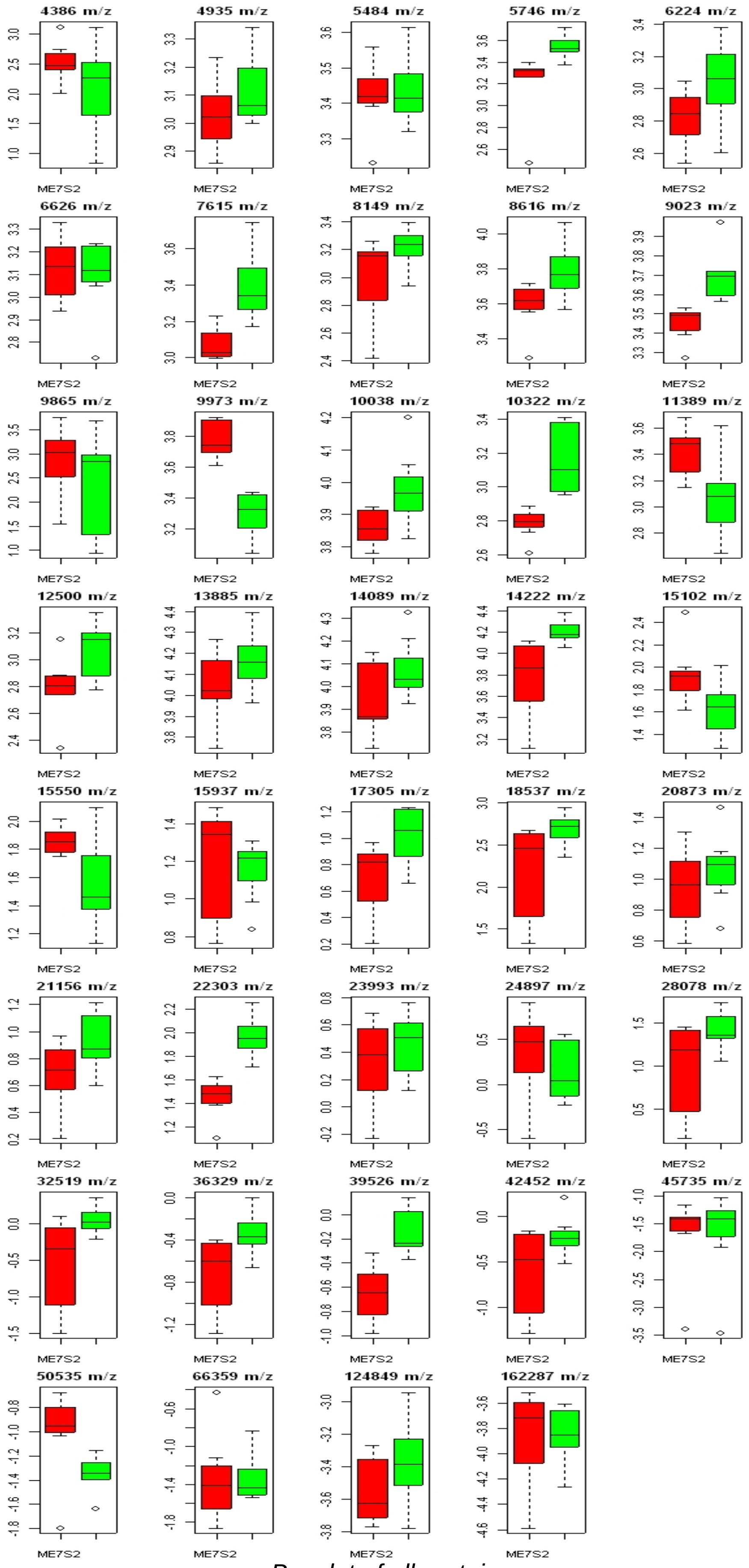

Boxplot of all proteins

Cluster Analysis of samples (Euclidean distance)

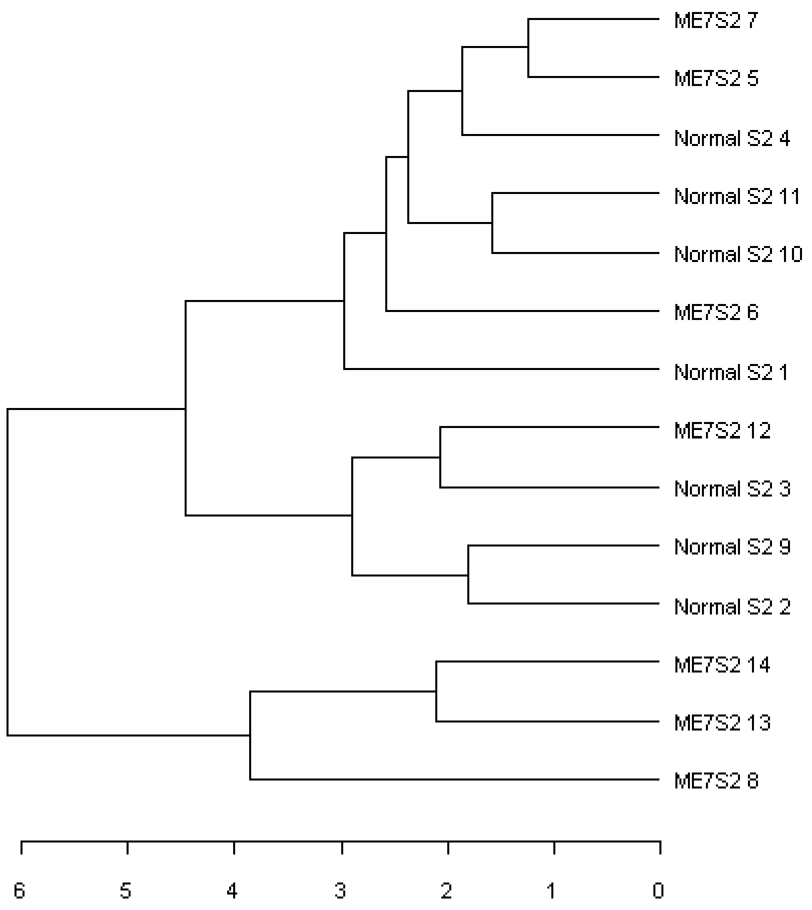

Cluster Analysis of samples (Euclidean distance)

S2 CM10 240

Proteins showing total separation

	C0GROUP	C0GRP_NA	C0Spectr	C04116_7	C05703_9	C09968_2	C011386_	C017096_	C025129_	C034052_	C050556_	C066211_	C0107215
4	0	ME7S2	B36864	2.2	2.8	3.7	3.9	0.608	0.591	-0.93	-1.06	-1.4	-3.2
5	0	ME7S2	B36865	2.2	3.2	3.9	3.7	0.570	0.805	-0.82	-0.89	-1.4	-2.7
6	0	ME7S2	B36866	2.3	3.0	3.8	4.2	0.763	1.005	-0.66	-0.59	-1.5	-3.2
7	0	ME7S2	B36868	2.6	2.8	3.9	3.7	0.088	0.431	-0.94	-1.21	-1.4	-3.3
1	1	Normal S2	B36860	1.8	3.3	2.7	3.7	1.179	0.385	-1.05	-1.41	-1.7	-3.6
2	1	Normal S2	B36861	2.0	3.2	3.0	3.5	0.898	0.306	-1.48	-1.26	-1.9	-3.5
3	1	Normal S2	B36862	2.1	3.4	3.1	3.4	1.286	-0.036	-1.71	-1.51	-1.8	-3.3

Boxplot of proteins showing complete separation

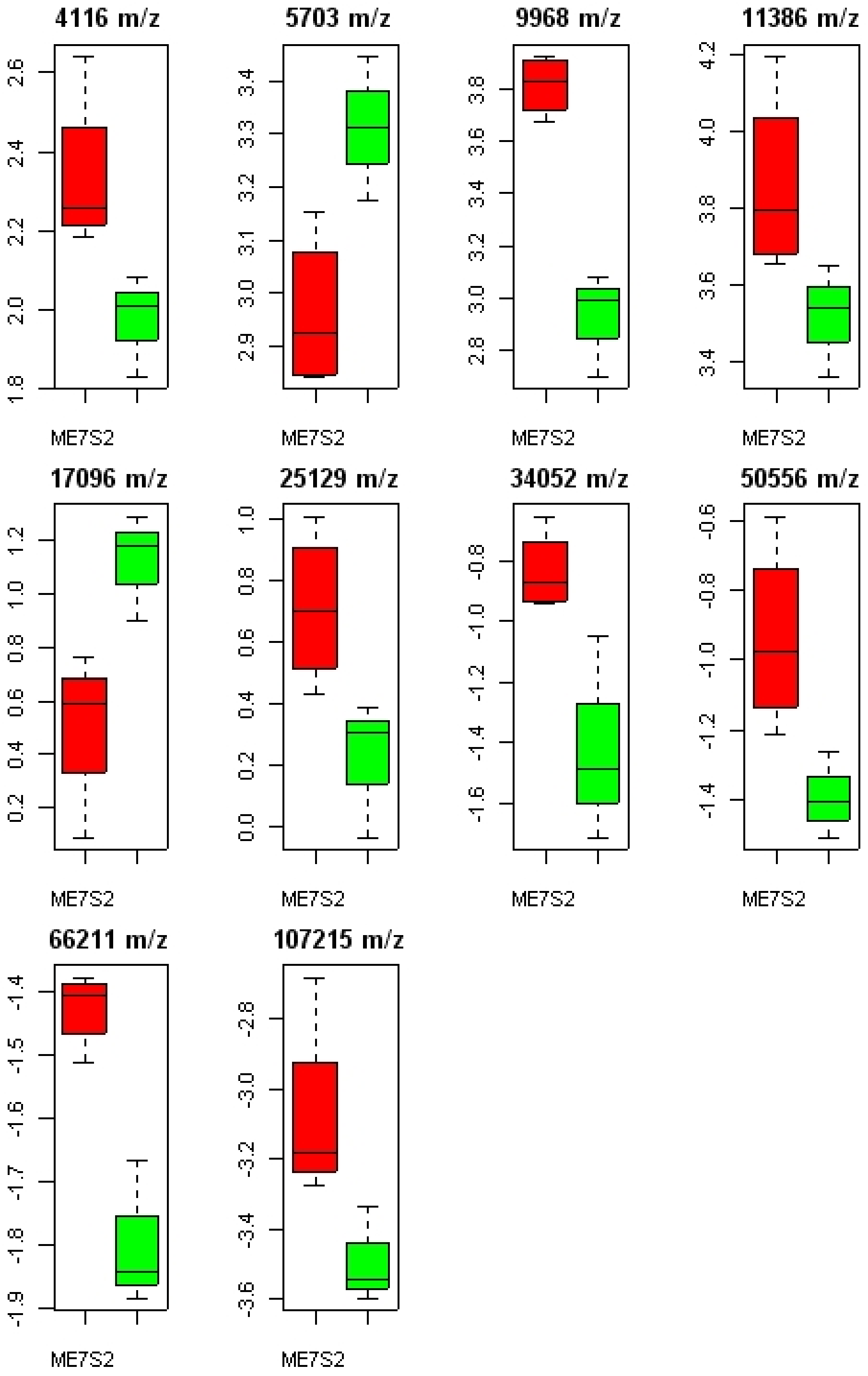
4116 m/z
1.8 2.0 2.2 2.4 2.6
ME7S2
5703 m/z
2.9 3.0 3.1 3.2 3.3 3.4
ME7S2
9968 m/z
2.8 3.0 3.2 3.4 3.6 3.8
ME7S2
11386 m/z
3.4 3.6 3.8 4.0 4.2
ME7S2
17096 m/z
0.2 0.4 0.6 0.8 1.0 1.2
ME7S2
25129 m/z
0.0 0.2 0.4 0.6 0.8 1.0
ME7S2
34052 m/z
-1.6 -1.4 -1.2 -1.0 -0.8
ME7S2
50556 m/z
-1.4 -1.2 -1.0 -0.8 -0.6
ME7S2
66211 m/z
-1.9 -1.8 -1.7 -1.6 -1.5 -1.4
ME7S2
107215 m/z
-3.6 -3.4 -3.2 -3.0 -2.8
ME7S2

Pairwise Scatterplots of Proteins showing complete separation

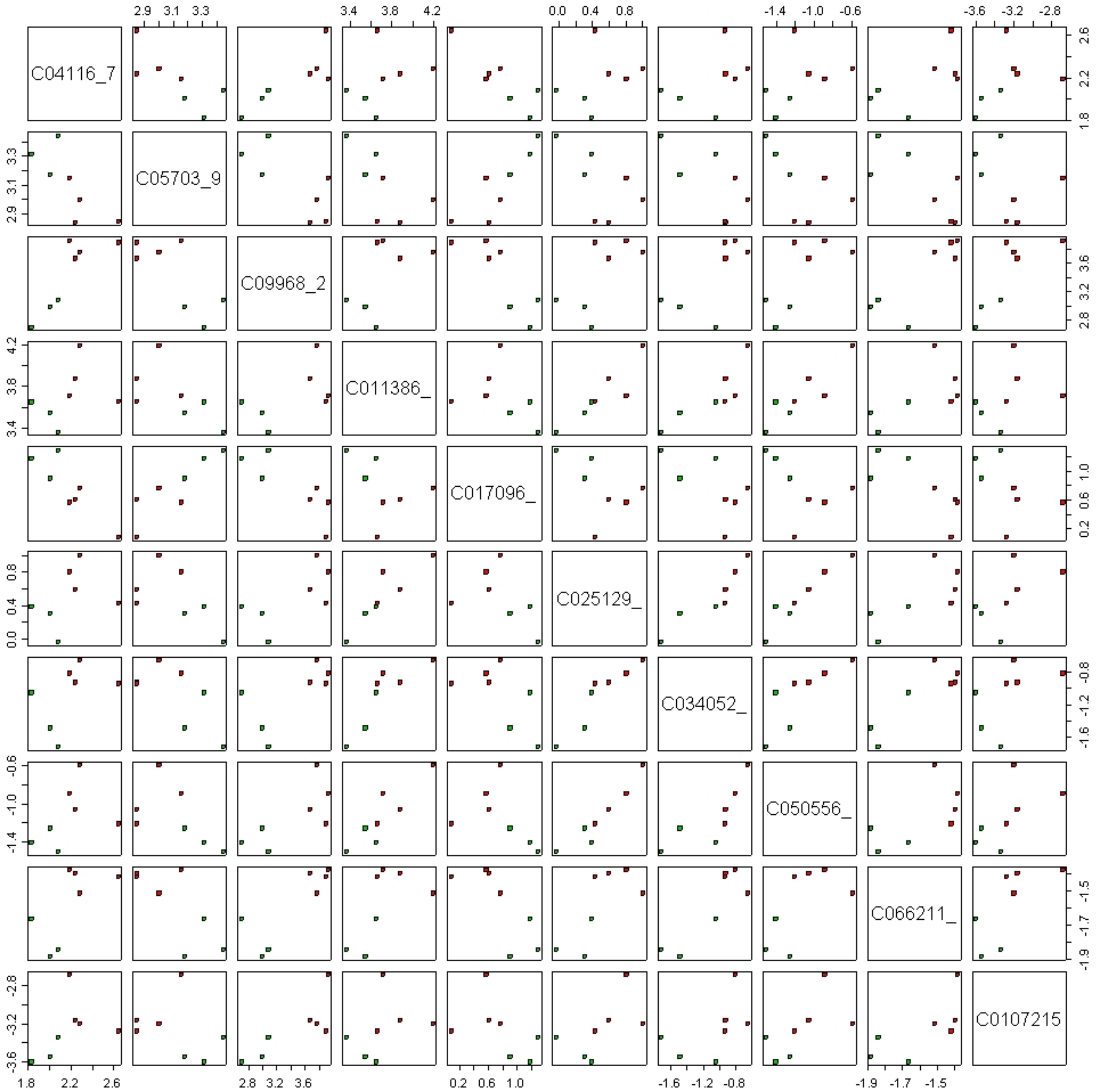

Significant data (p<=0.01)

Significant Proteins (t-test; p<=0.01)

	name	mz	ME7.avg	NORM.avg	t	p
13	C09968_2	9968	3.8	2.9	6.9	0.006

Data for Significant proteins

	C0GROUP	C0GRP_NA	C0Spectr	C09968_2
4	0	ME7S2	B36864	3.7
5	0	ME7S2	B36865	3.9
6	0	ME7S2	B36866	3.8
7	0	ME7S2	B36868	3.9
1	1	Normal S2	B36860	2.7
2	1	Normal S2	B36861	3.0
3	1	Normal S2	B36862	3.1

Boxplot of significant proteins

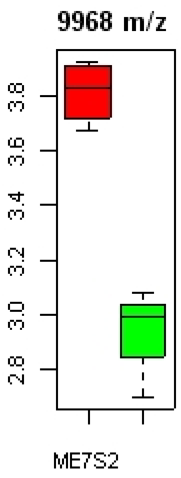

Boxplot of significant proteins

All data

Boxplot of all proteins

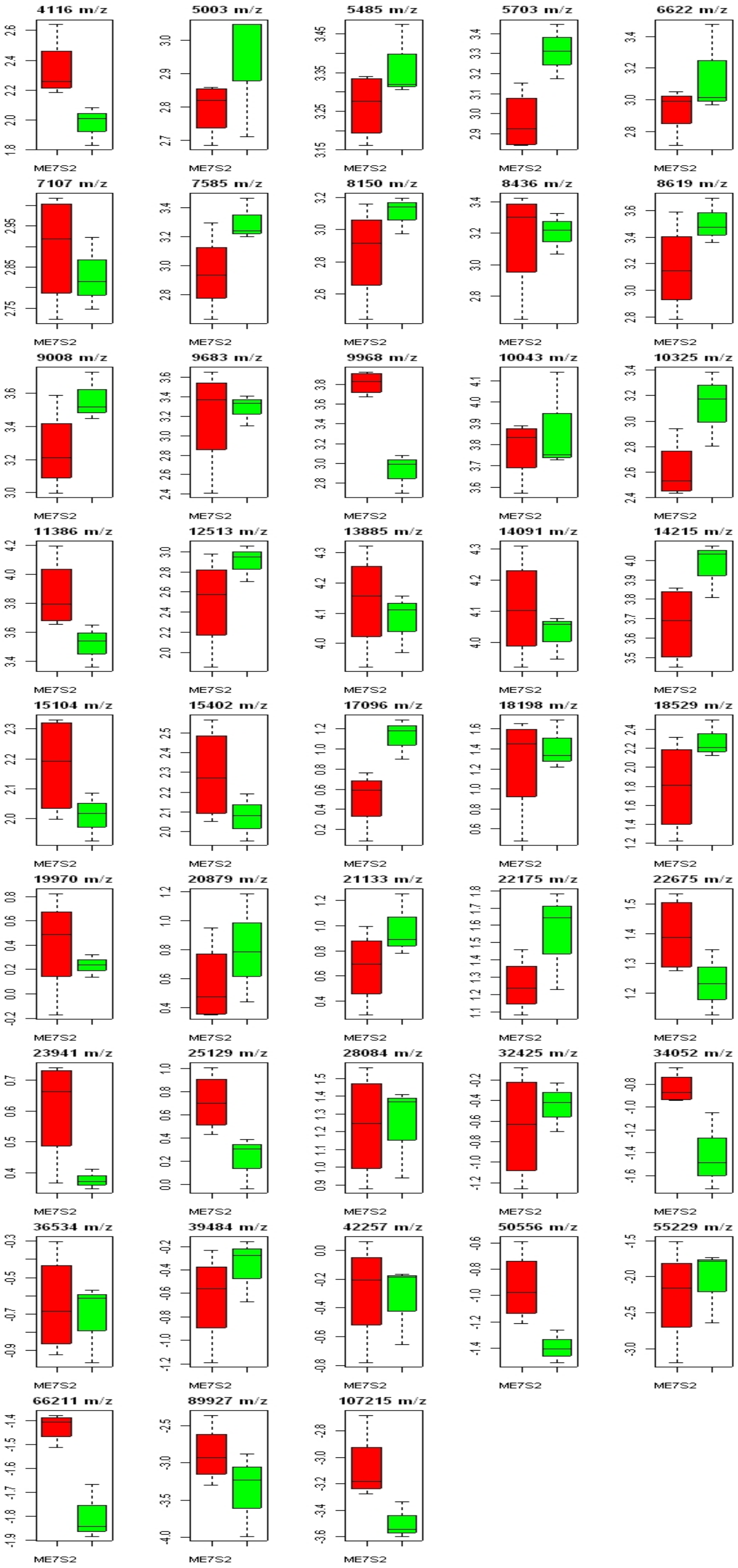

Boxplot of all proteins

Cluster Analysis of samples (Euclidean distance)

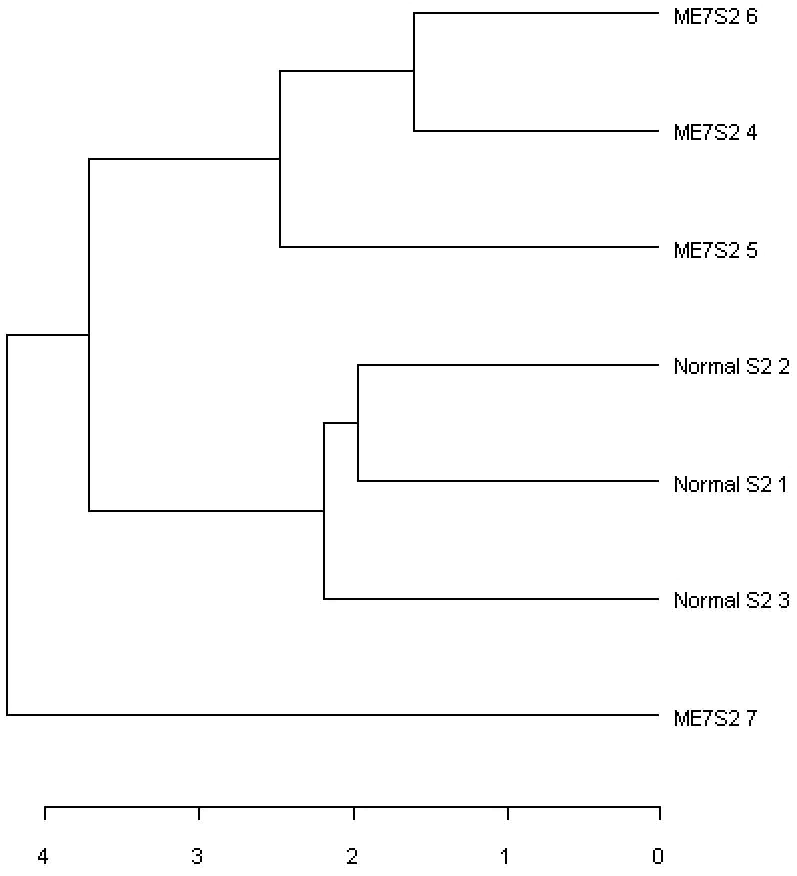

Cluster Analysis of samples (Euclidean distance)

S2 CM10 EP

Proteins showing total separation

	C0GROUP	C0GRP_NA	C0Spectr	C09014_7	C09683_4	C010322_	C012516_	C021027_
1	0	ME7S2	B36885	3.0	3.0	2.0	2.9	0.93
3	0	ME7S2	B37003	3.2	2.8	2.7	2.7	0.72
4	0	ME7S2	B37134	3.2	2.5	2.3	2.4	0.47
8	0	ME7S2	B37004	3.1	2.8	2.9	2.6	1.01
9	0	ME7S2	B37020	3.3	3.0	2.8	2.8	0.82
11	0	ME7S2	B37283	3.4	3.0	3.0	2.9	0.84
12	0	ME7S2	B37285	3.2	3.0	2.7	2.7	0.98
2	1	Normal S2	B37002	3.5	3.7	3.4	3.3	1.31
5	1	Normal S2	B37284	3.5	3.7	3.5	3.3	1.30
6	1	Normal S2	B37329	3.8	3.9	3.5	3.6	1.46
7	1	Normal S2	B37501	3.6	3.5	3.2	3.2	1.01
10	1	Normal S2	B37133	3.8	3.6	3.9	3.5	1.36
13	1	Normal S2	B37499	3.6	3.2	3.5	3.3	1.08
14	1	Normal S2	B37500	3.5	3.2	3.3	3.1	1.13

Boxplot of proteins showing complete separation

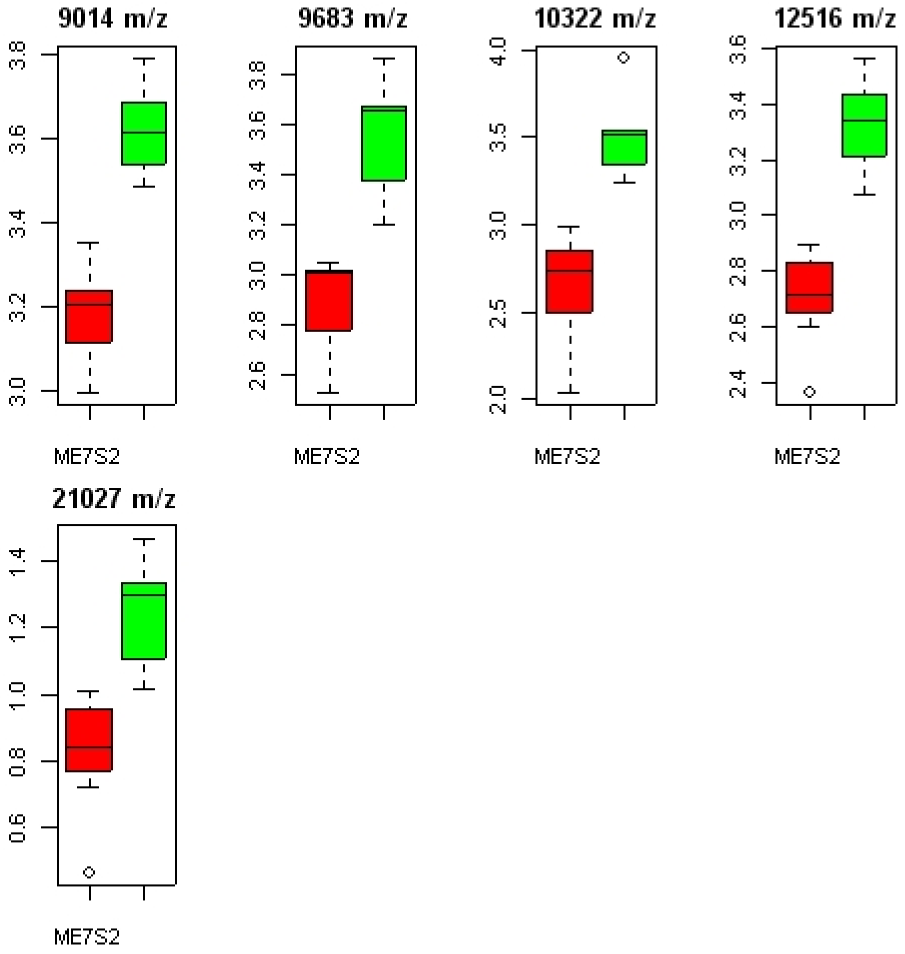

Pairwise Scatterplots of Proteins showing complete separation

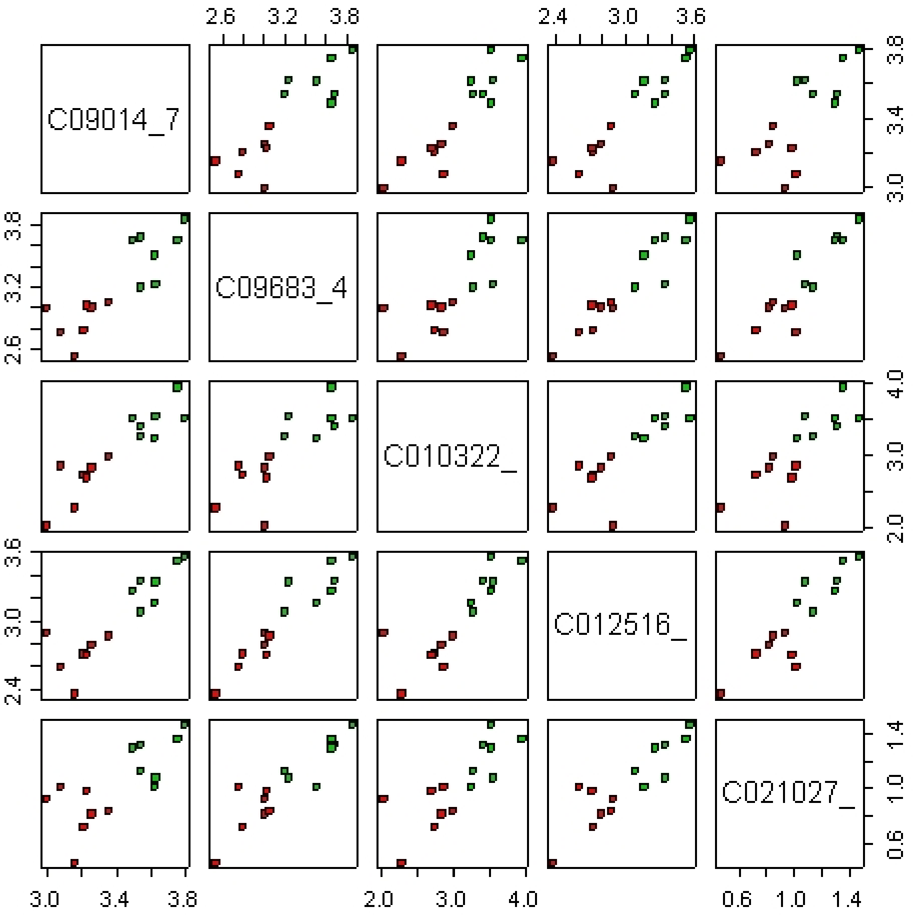

Significant data (p<=0.01)

Significant Proteins (t-test; p<=0.01)

	name	mz	ME7.avg	NORM.avg	t	p
5	C05706_3	5706	3.14	3.50	-3.6	3.9e-03
7	C07586_9	7586	2.91	3.47	-4.6	9.4e-04
8	C08154_4	8154	2.64	3.16	-5.4	2.5e-04
9	C08437_8	8437	2.88	3.33	-3.5	6.0e-03
11	C09014_7	9014	3.18	3.62	-7.1	1.3e-05
12	C09683_4	9683	2.88	3.54	-5.6	1.4e-04
14	C010322_	10322	2.64	3.49	-5.4	2.3e-04
15	C010799_	10799	1.99	2.48	-3.3	8.0e-03
17	C012516_	12516	2.71	3.33	-6.4	3.2e-05
25	C017266_	17266	0.48	0.99	-3.2	8.0e-03
28	C019988_	19988	0.26	0.64	-4.1	1.6e-03
29	C021027_	21027	0.82	1.24	-4.4	9.4e-04
33	C024879_	24879	0.76	0.19	5.5	2.5e-04

Data for Significant proteins

	C0GROUP	C0GRP_NA	C0Spectr	C05706_3	C07586_9	C08154_4	C08437_8	C09014_7	C09683_4	C010322_	C010799_	C012516_	C017266_	C019988_	C021027_	C024879_
1	0	ME7S2	B36885	3.0	2.8	2.7	2.8	3.0	3.0	2.0	2.4	2.9	-0.020	0.549	0.93	0.705
3	0	ME7S2	B37003	3.2	3.0	2.4	2.7	3.2	2.8	2.7	1.8	2.7	0.533	0.302	0.72	0.642
4	0	ME7S2	B37134	2.7	2.9	2.6	2.8	3.2	2.5	2.3	1.5	2.4	0.219	0.121	0.47	0.558
8	0	ME7S2	B37004	3.2	3.2	2.7	2.9	3.1	2.8	2.9	1.9	2.6	0.472	0.175	1.01	0.978
9	0	ME7S2	B37020	3.3	2.9	2.6	2.8	3.3	3.0	2.8	1.8	2.8	0.693	0.404	0.82	0.885
11	0	ME7S2	B37283	3.2	2.7	2.9	3.2	3.4	3.0	3.0	2.1	2.9	0.925	-0.039	0.84	0.722
12	0	ME7S2	B37285	3.2	2.9	2.6	2.9	3.2	3.0	2.7	2.3	2.7	0.564	0.291	0.98	0.842
2	1	Normal S2	B37002	3.7	3.8	3.2	3.6	3.5	3.7	3.4	2.5	3.3	0.832	0.602	1.31	0.095
5	1	Normal S2	B37284	3.6	3.5	3.2	3.4	3.5	3.7	3.5	2.5	3.3	1.346	0.638	1.30	0.069
6	1	Normal S2	B37329	3.5	3.5	3.4	3.7	3.8	3.9	3.5	2.8	3.6	1.286	0.751	1.46	0.146
7	1	Normal S2	B37501	3.4	3.4	3.3	3.2	3.6	3.5	3.2	2.2	3.2	0.903	0.796	1.01	0.329
10	1	Normal S2	B37133	3.7	3.7	3.2	3.4	3.8	3.6	3.9	2.5	3.5	1.232	0.812	1.36	0.625
13	1	Normal S2	B37499	3.3	3.0	3.0	3.1	3.6	3.2	3.5	2.4	3.3	0.584	0.399	1.08	-0.103
14	1	Normal S2	B37500	3.3	3.3	2.8	2.8	3.5	3.2	3.3	2.3	3.1	0.782	0.496	1.13	0.203

Boxplot of significant proteins

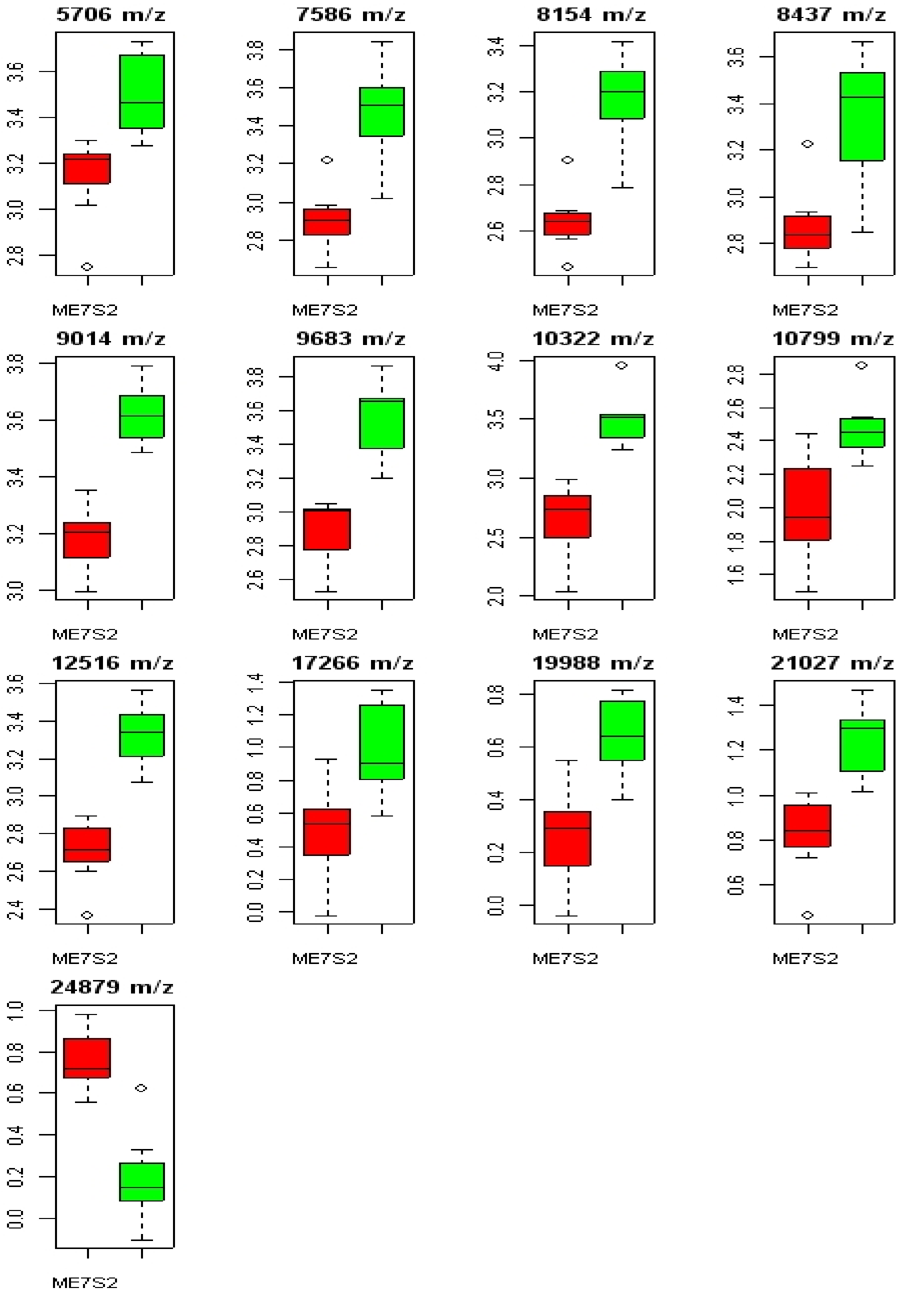

Boxplot of significant proteins

Pairwise Scatterplots of Significant Proteins

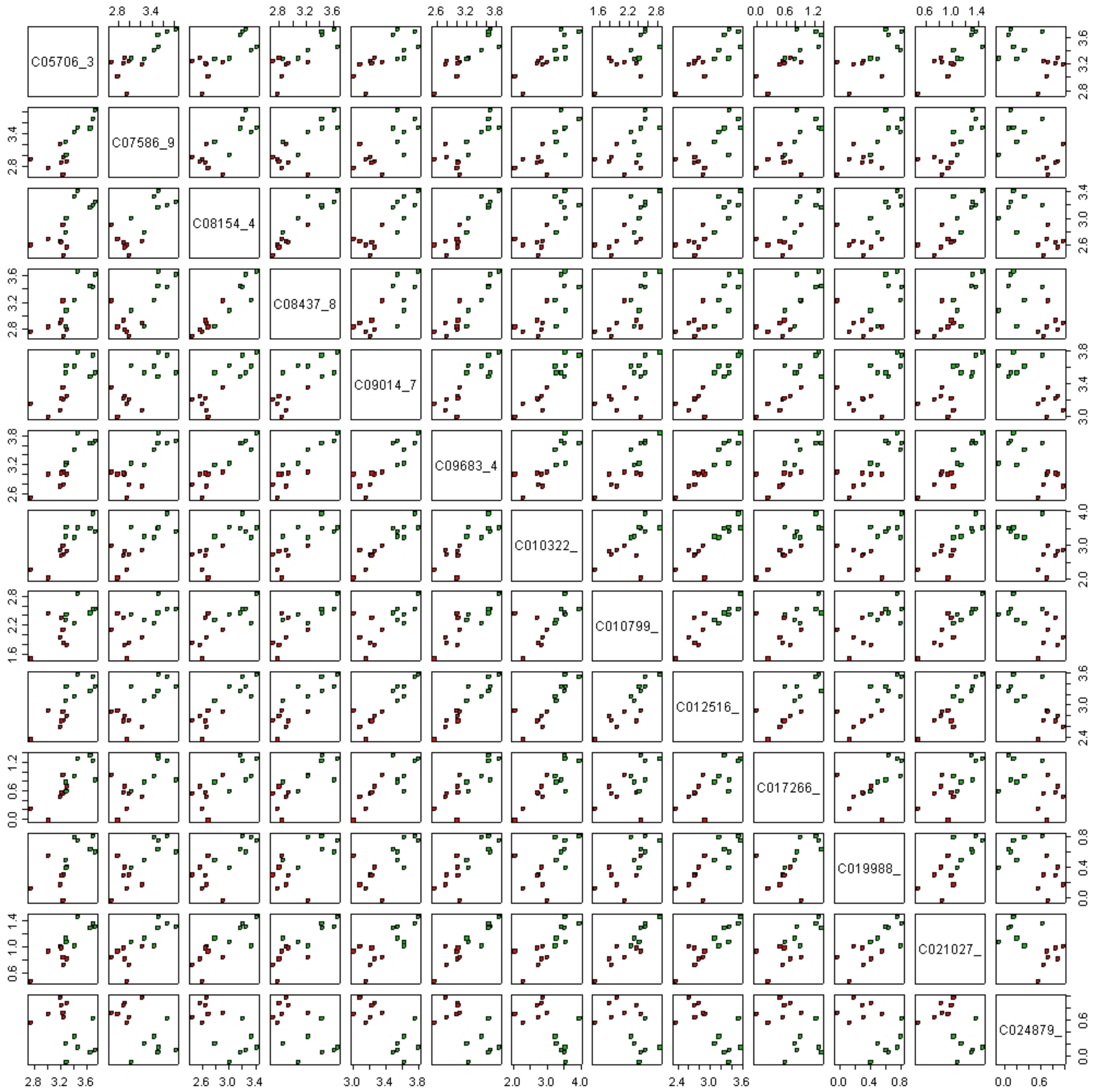

Pairwise Scatterplots of Significant Proteins

Cluster Analysis of samples (Euclidean distance)

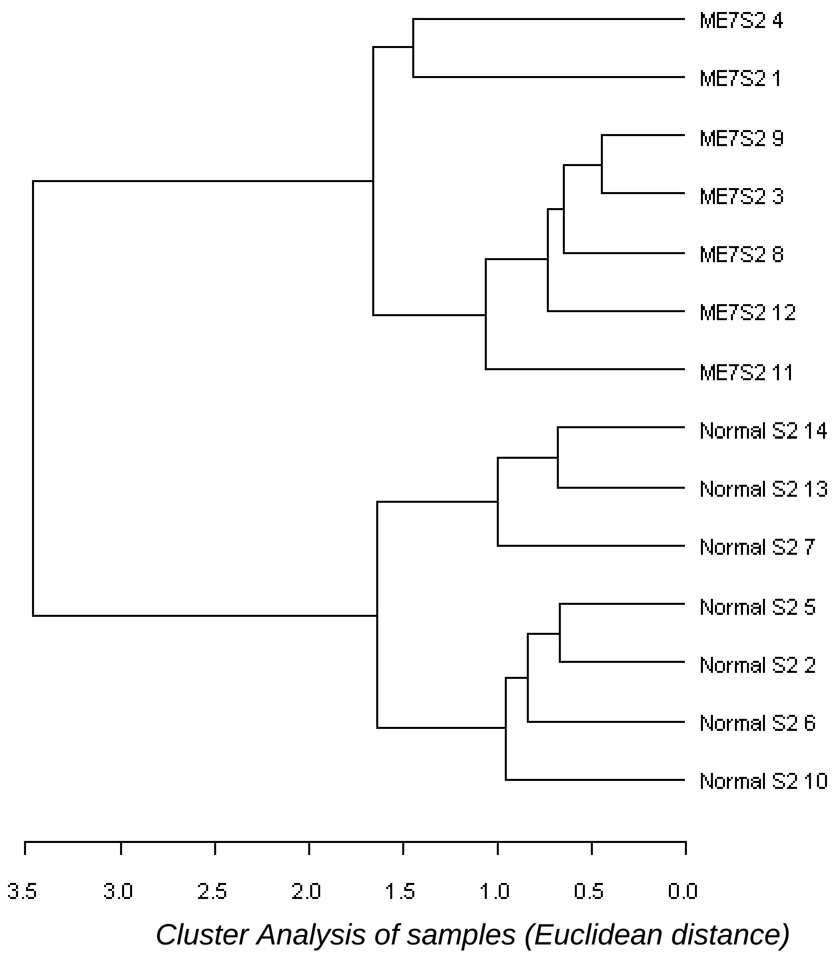

Plot of first three principal components

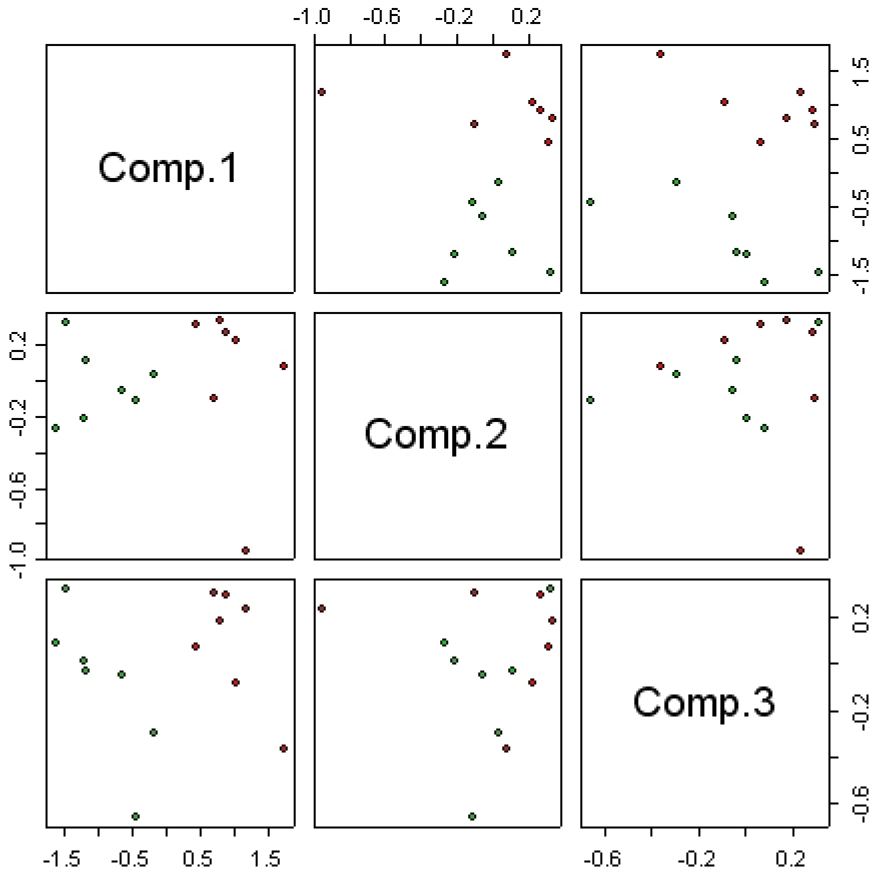

Plot of first three principal components

Scatterplot of linear discriminant function (x-axis)

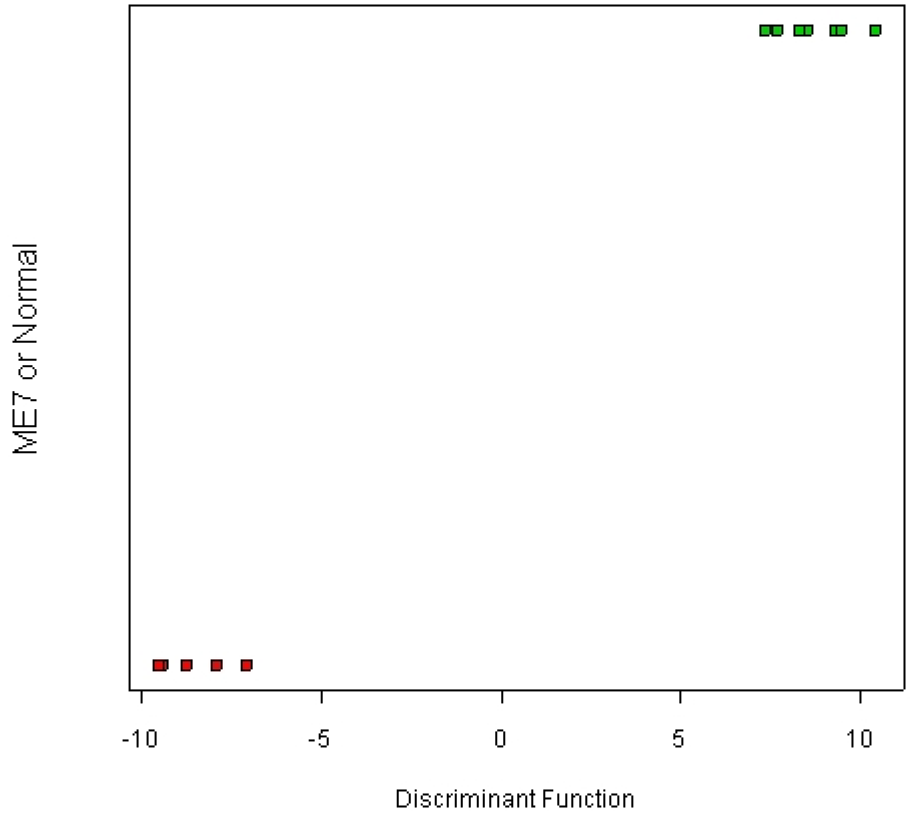

Scatterplot of linear discriminant function (x-axis)

All data

Boxplot of all proteins

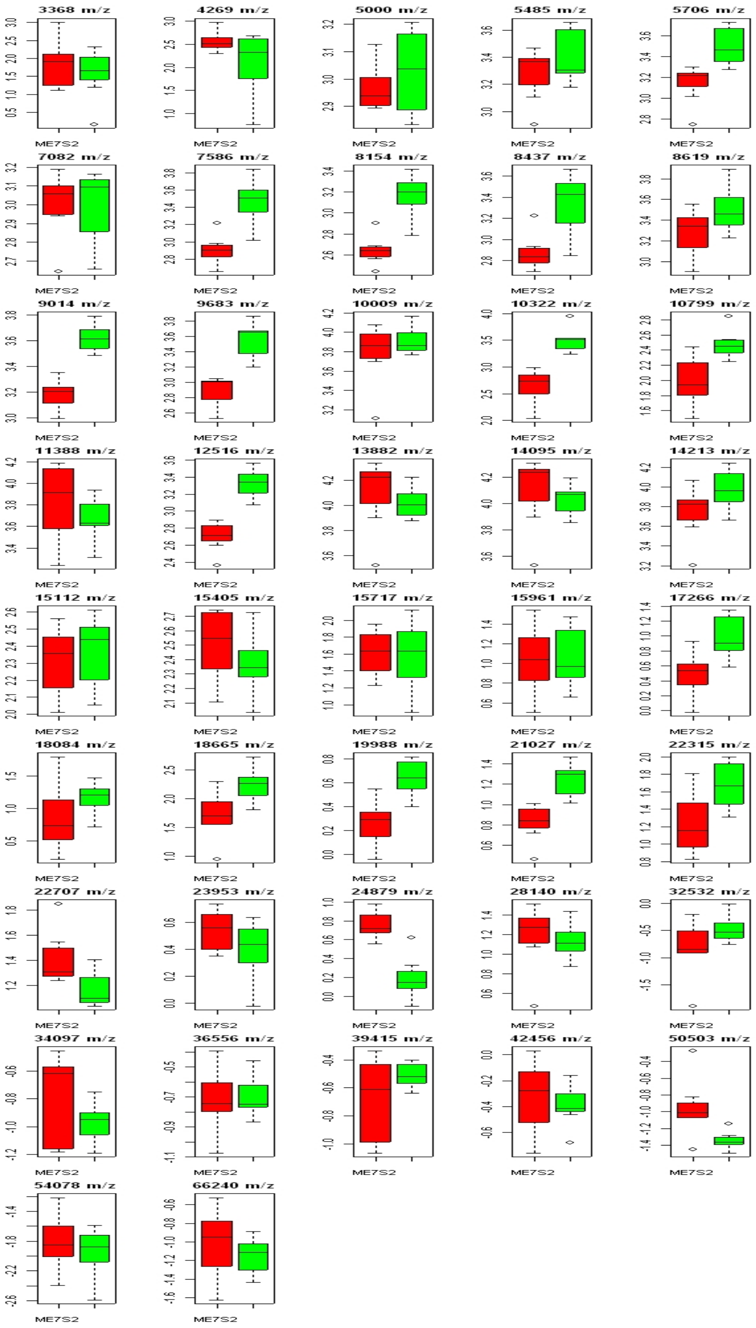

Boxplot of all proteins

Cluster Analysis of samples (Euclidean distance)

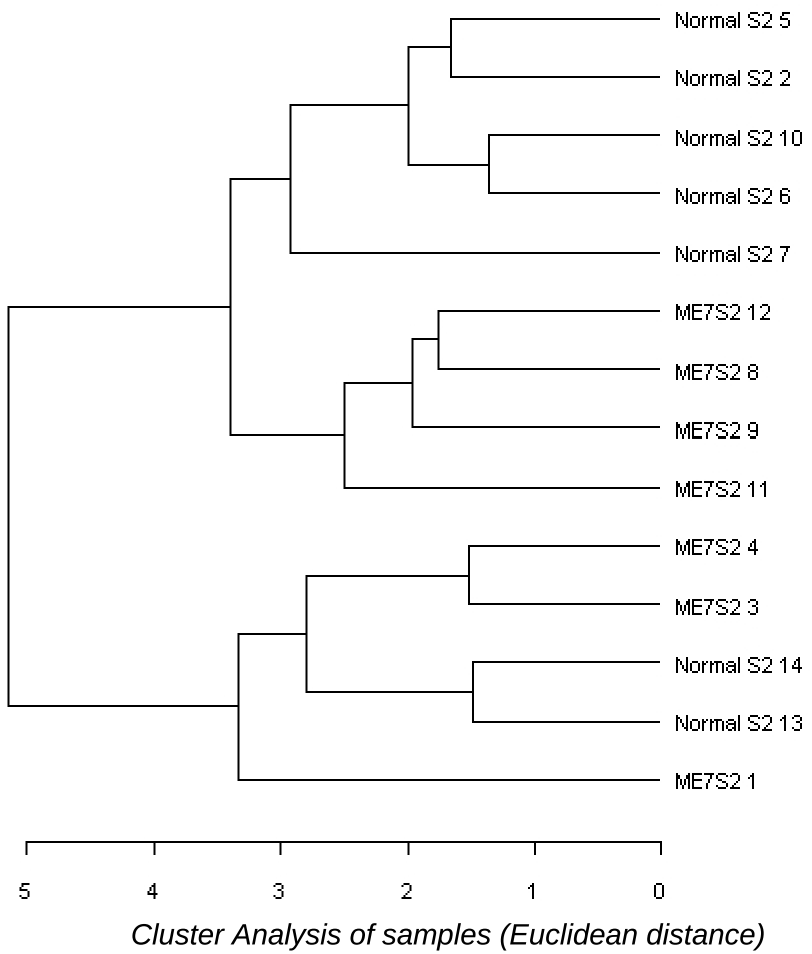

Supplement: Additional file 8 — Full statistical analysis of CM10 supernatant 2 arrays [file 1471-2334-9-188-S8.PDF]
