# Supplementary material for: Differential protein profiling as a potential multi-marker approach for TSE diagnosis
Source: BMC Infect Dis. 2009 Nov 27;9:188. doi: 10.1186/1471-2334-9-188 (PMC2794872; doi:10.1186/1471-2334-9-188)

S2 Q10 30

Proteins showing total separation

	C0GROUP	C0GRP_NA	C0Spectr	C07354_3
5	0	ME7S2	B32943	1.344
6	0	ME7S2	B32944	1.395
7	0	ME7S2	B32945	1.461
11	0	ME7S2	B32943	1.337
12	0	ME7S2	B32946	0.010
13	0	ME7S2	B32947	1.353
14	0	ME7S2	B32948	1.111
1	1	Normal S2	B32937	1.819
2	1	Normal S2	B32938	1.966
3	1	Normal S2	B32939	2.304
4	1	Normal S2	B32940	2.143
8	1	Normal S2	B32939	2.192
9	1	Normal S2	B32941	1.486
10	1	Normal S2	B32942	1.540

Boxplot of proteins showing complete separation

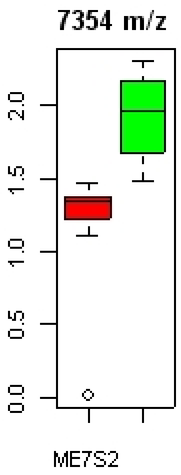

Significant data (p<=0.05)

Significant Proteins (t-test; p<=0.05)

	name	mz	ME7.avg	NORM.avg	t	p
8	C06740_1	6740	2.5	2.8	-3.4	0.0059
11	C07354_3	7354	1.1	1.9	-3.4	0.0066
14	C09376_9	9376	2.9	3.2	-2.6	0.0270
15	C09677_6	9677	3.1	3.3	-2.3	0.0459
16	C011374_	11374	3.2	3.5	-3.4	0.0081
18	C013857_	13857	3.3	3.6	-3.4	0.0094
19	C014083_	14083	3.3	3.5	-3.1	0.0161

Data for Significant proteins

	C0GROUP	C0GRP_NA	C0Spectr	C06740_1	C07354_3	C09376_9	C09677_6	C011374_	C013857_	C014083_
5	0	ME7S2	B32943	2.5	1.344	2.9	3.1	3.3	3.4	3.4
6	0	ME7S2	B32944	2.5	1.395	2.9	3.2	3.1	3.2	3.1
7	0	ME7S2	B32945	2.7	1.461	3.2	3.2	3.3	3.4	3.3
11	0	ME7S2	B32943	2.4	1.337	2.9	3.1	3.1	3.5	3.4
12	0	ME7S2	B32946	2.0	0.010	2.5	2.7	2.8	3.1	3.0
13	0	ME7S2	B32947	2.5	1.353	3.0	3.1	3.4	3.5	3.5
14	0	ME7S2	B32948	2.6	1.111	3.1	3.4	3.3	3.2	3.1
1	1	Normal S2	B32937	2.6	1.819	3.0	3.2	3.3	3.5	3.5
2	1	Normal S2	B32938	2.9	1.966	3.2	3.3	3.4	3.5	3.5
3	1	Normal S2	B32939	2.7	2.304	3.1	3.3	3.5	3.6	3.5
4	1	Normal S2	B32940	3.1	2.143	3.4	3.5	3.6	3.6	3.5
8	1	Normal S2	B32939	2.8	2.192	3.3	3.4	3.5	3.5	3.4
9	1	Normal S2	B32941	2.7	1.486	3.2	3.3	3.6	3.7	3.6
10	1	Normal S2	B32942	2.9	1.540	3.2	3.2	3.4	3.5	3.5

Boxplot of significant proteins

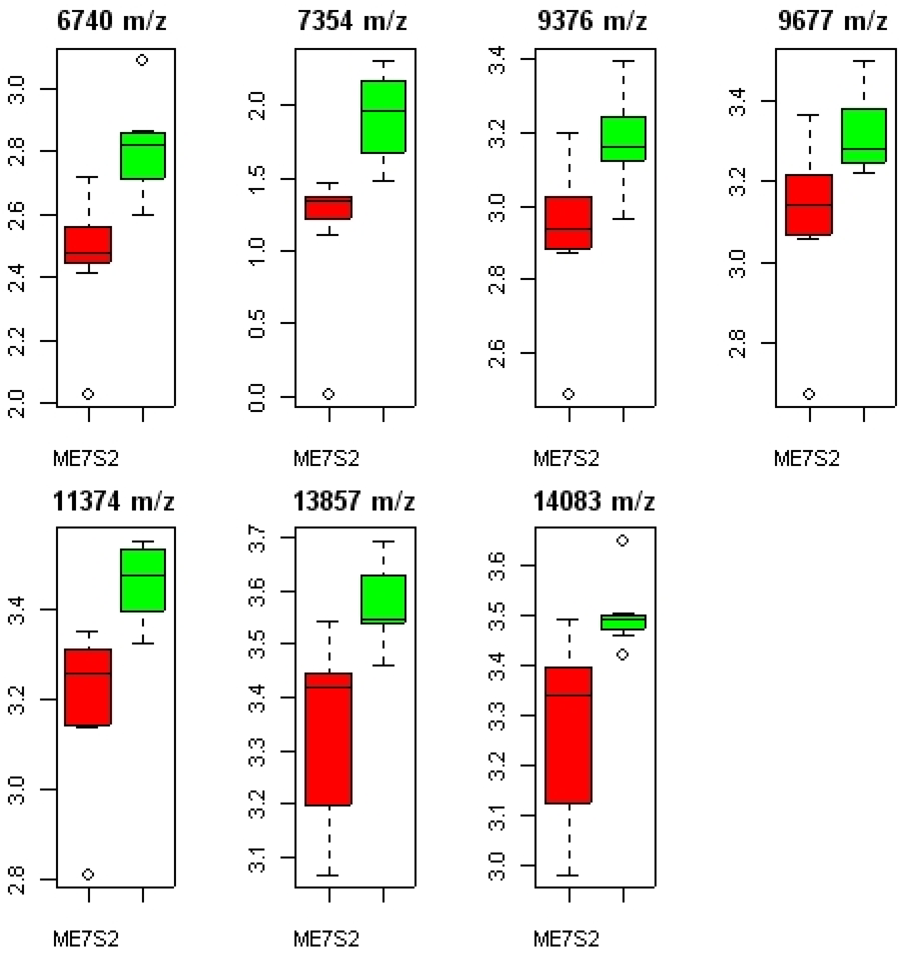

Boxplot of significant proteins

Pairwise Scatterplots of Significant Proteins

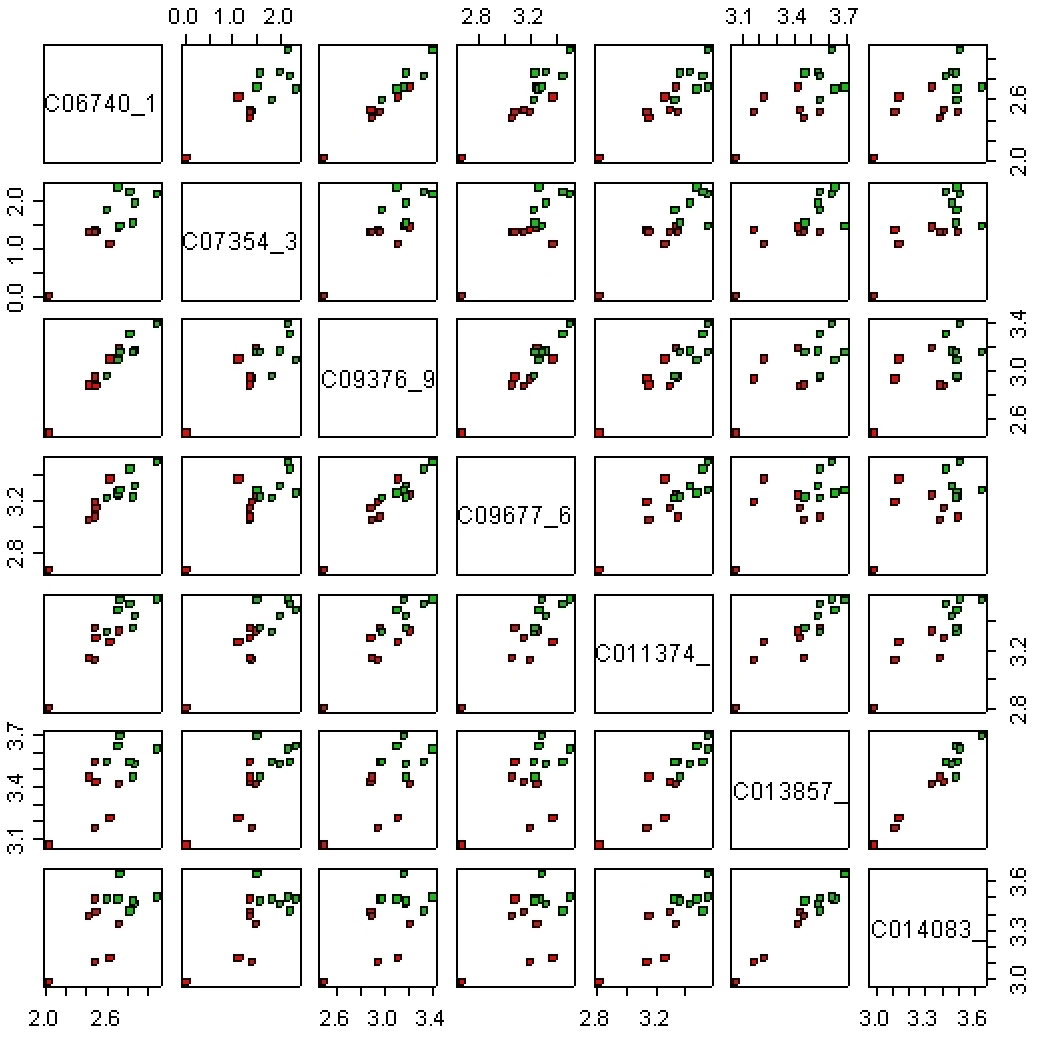

Pairwise Scatterplots of Significant Proteins

Cluster Analysis of samples (Euclidean distance)

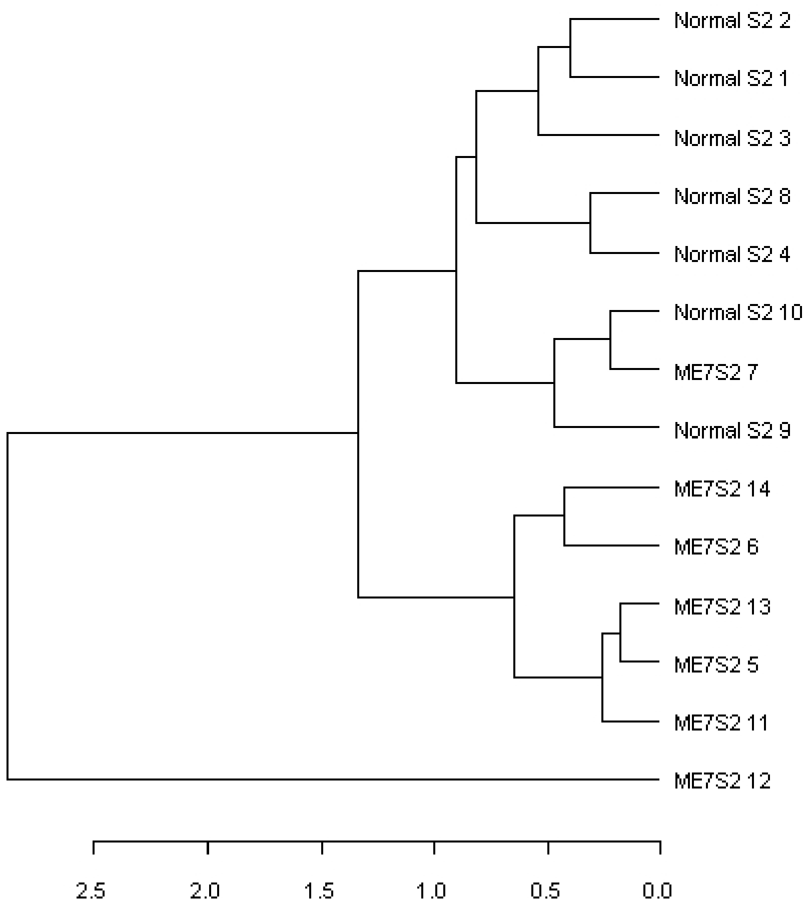

Cluster Analysis of samples (Euclidean distance)

Plot of first three principal components

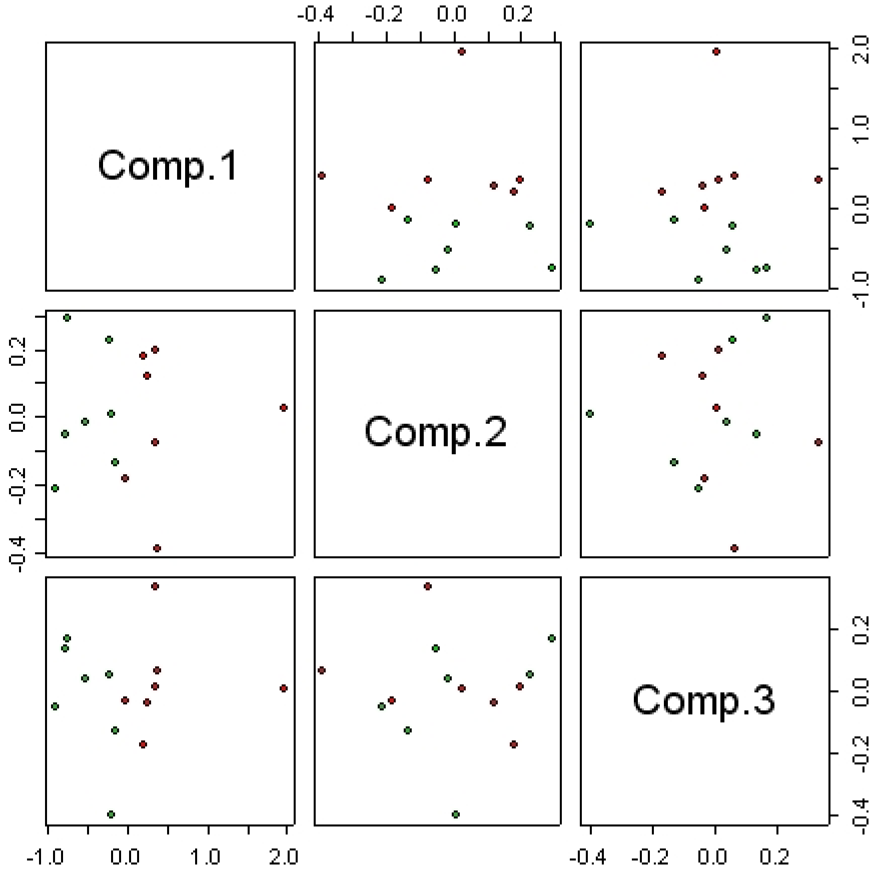

Plot of first three principal components

Scatterplot of linear discriminant function (x-axis)

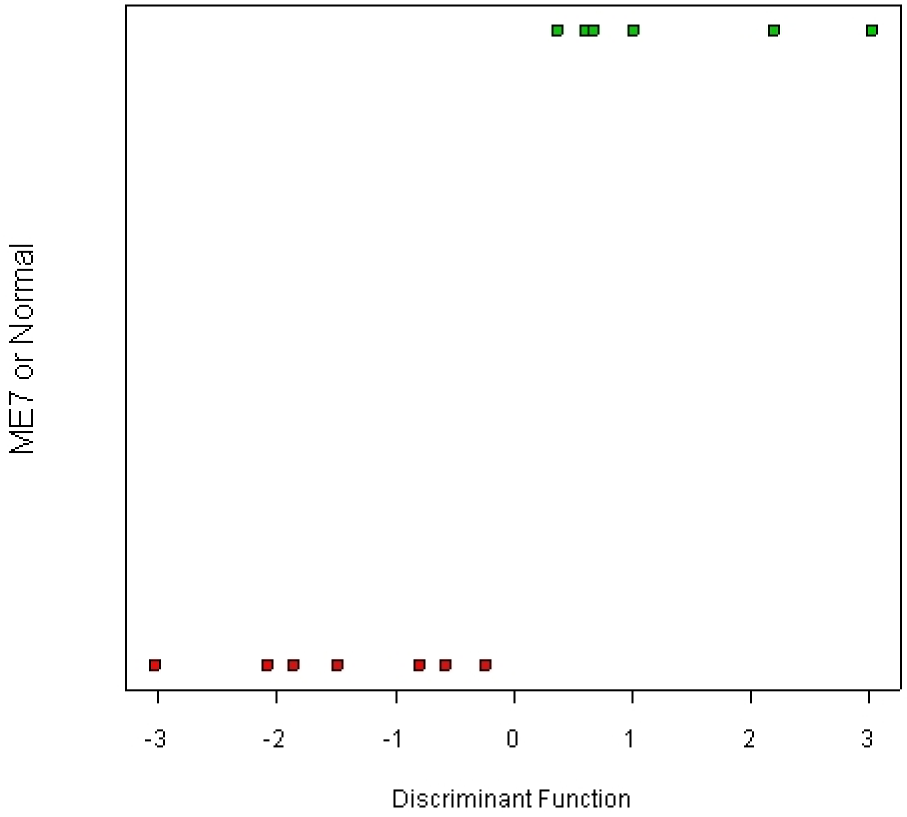

Scatterplot of linear discriminant function (x-axis)

All data

Boxplot of all proteins

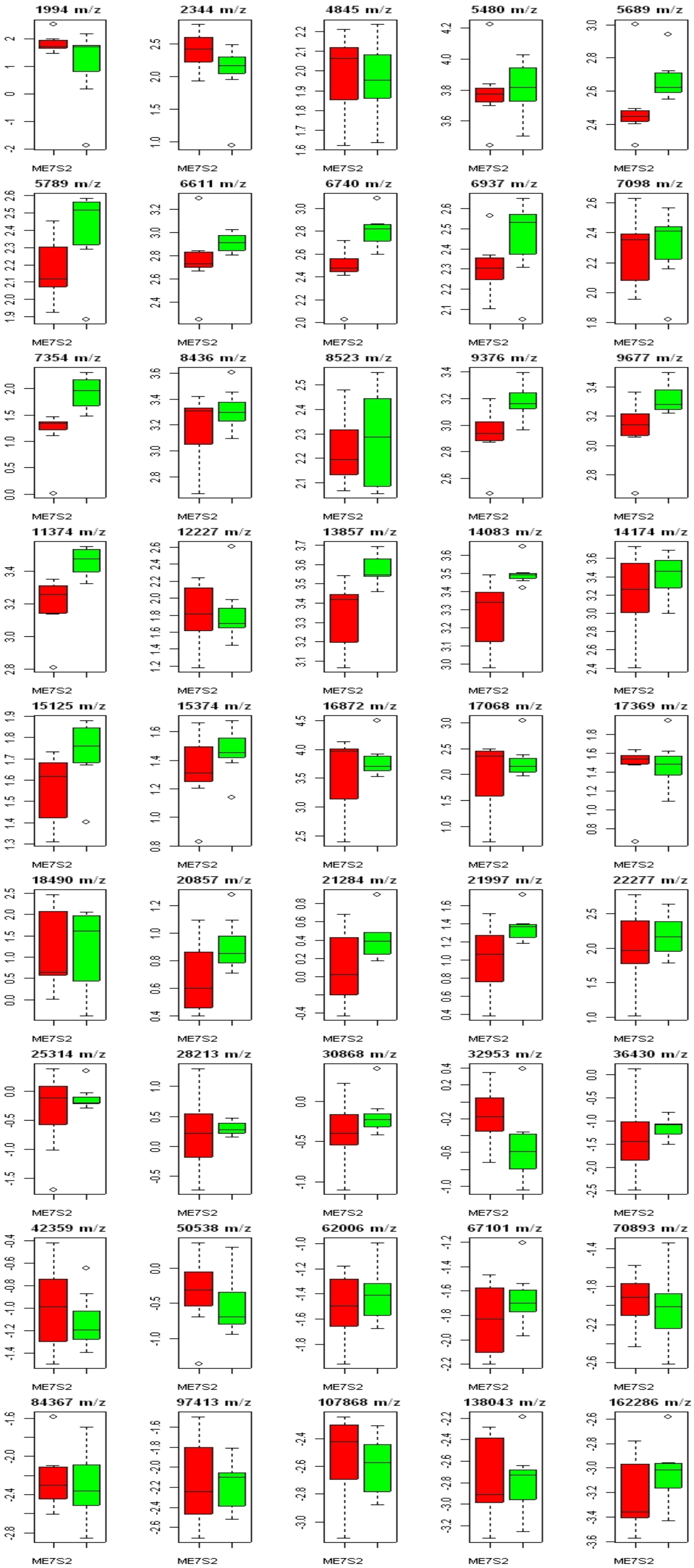

Boxplot of all proteins

Cluster Analysis of samples (Euclidean distance)

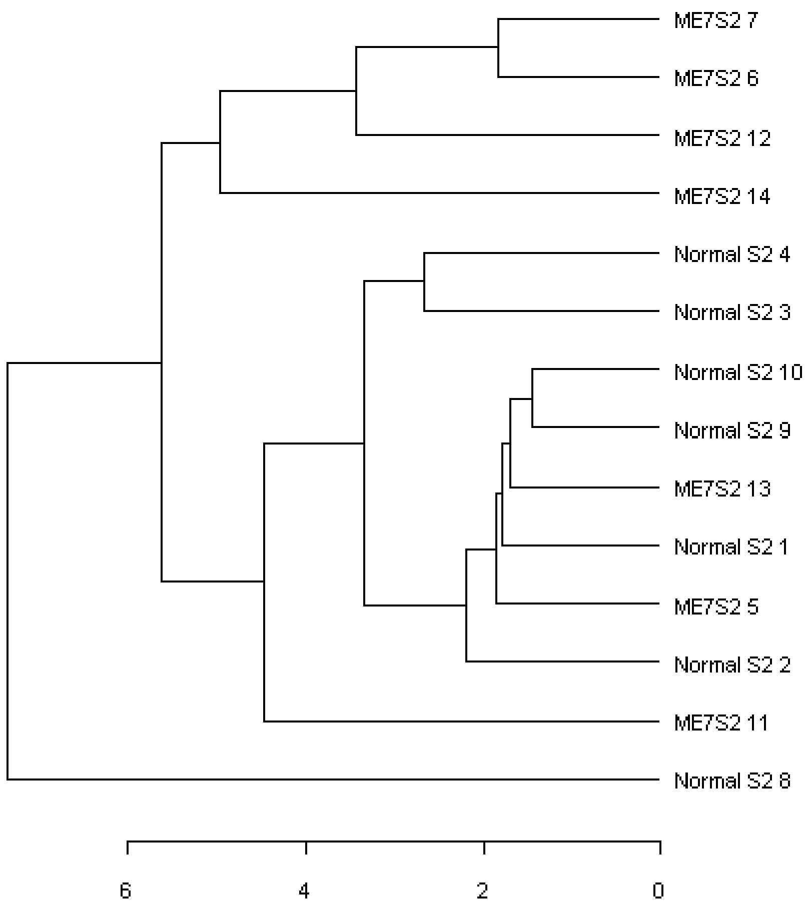

Cluster Analysis of samples (Euclidean distance)

S2 Q10 60

Proteins showing total separation

No proteins showed complete separation

Significant data (p<=0.05)

Significant Proteins (t-test; p<=0.05)

	name	mz	ME7.avg	NORM.avg	t	p
12	C08436_5	8436	3.7	3.5	2.8	0.022

Data for Significant proteins

	C0GROUP	C0GRP_NA	C0Spectr	C08436_5
5	0	ME7 S2	B33451	3.7
6	0	ME7 S2	B33452	3.7
7	0	ME7 S2	B33453	3.8
12	0	ME7 S2	B33454	3.7
13	0	ME7 S2	B33455	3.6
14	0	ME7 S2	B33456	3.7
1	1	Normal S2	B33445	3.2
2	1	Normal S2	B33446	3.6
3	1	Normal S2	B33447	3.7
4	1	Normal S2	B33449	3.6
8	1	Normal S2	B33447	3.6
9	1	Normal S2	B33448	3.6
10	1	Normal S2	B33449	3.3
11	1	Normal S2	B33450	3.6

Boxplot of significant proteins

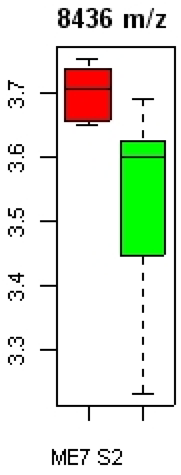

Boxplot of significant proteins

All data

Boxplot of all proteins

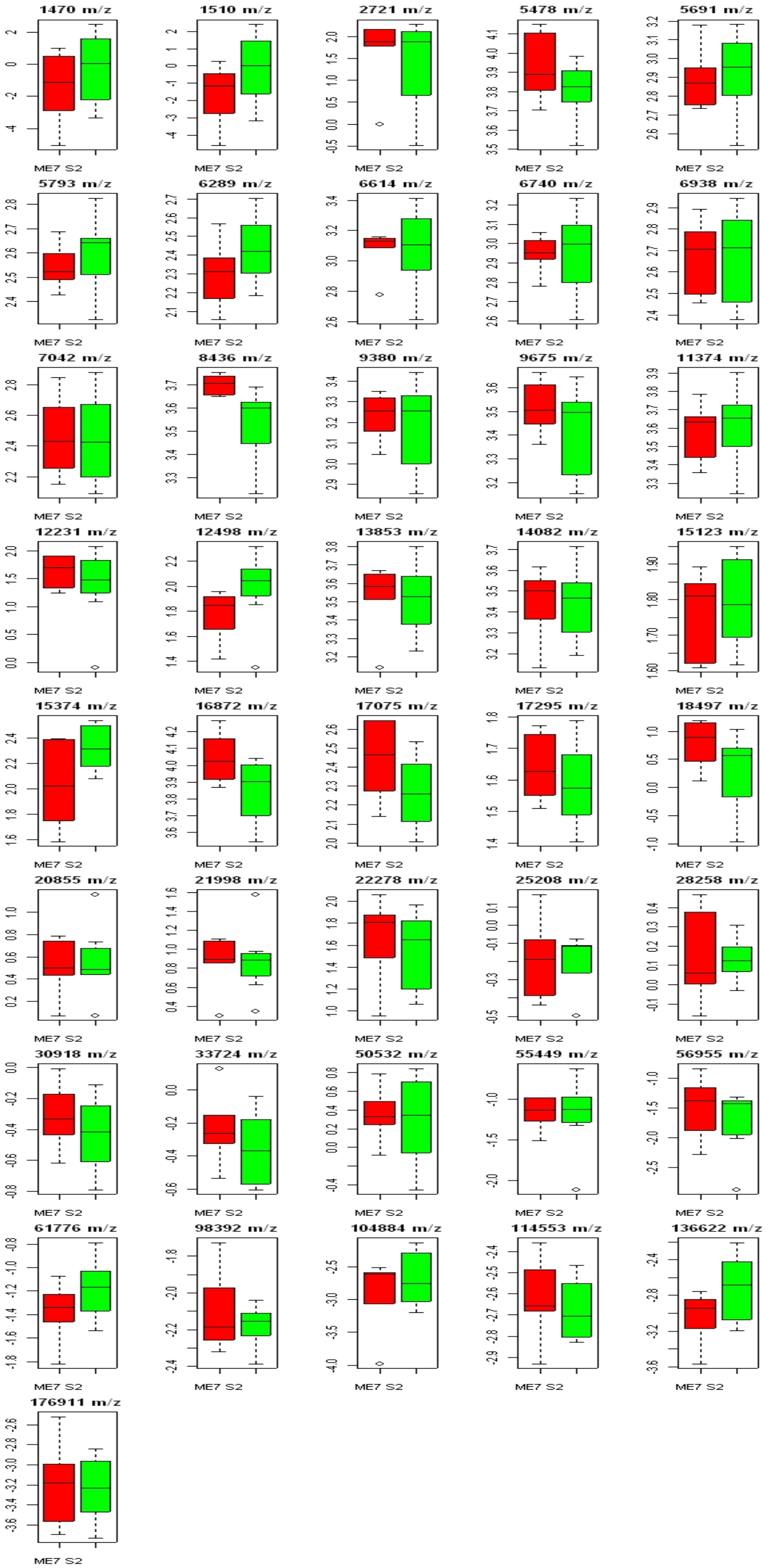

Boxplot of all proteins

Cluster Analysis of samples (Euclidean distance)

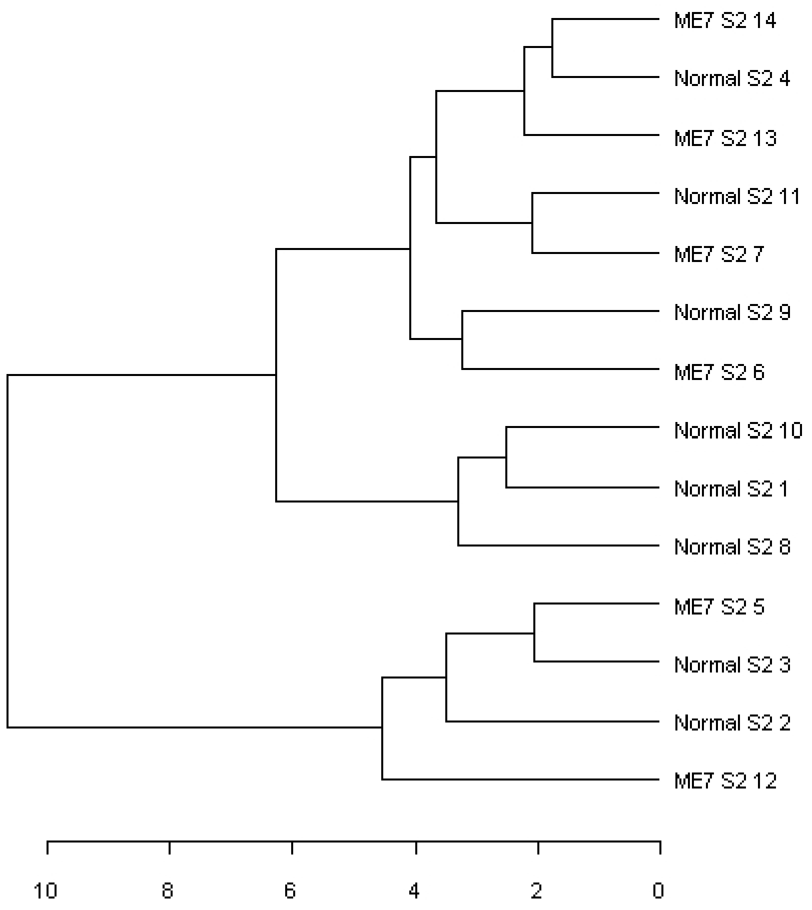

Cluster Analysis of samples (Euclidean distance)

S2 Q10 90

Proteins showing total separation

No proteins showed complete separation

Significant data (p<=0.05)

Significant Proteins (t-test; p<=0.05)

	name	mz	ME7.avg	NORM.avg	t	p
38	C030887_	30887	-1.65	-0.76	-2.3	0.041
42	C050631_	50631	-0.56	-1.35	2.4	0.032
46	C098318_	98318	-2.27	-3.06	2.8	0.031

Data for Significant proteins

	C0GROUP	C0GRP_NA	C0Spectr	C030887_	C050631_	C098318_
4	0	ME7S2	B34041	-2.15	-1.491	-2.2
5	0	ME7S2	B34043	-1.57	-1.607	-2.3
6	0	ME7S2	B34044	-2.32	-0.861	-2.3
7	0	ME7S2	B34045	-2.53	-0.937	-2.2
11	0	ME7S2	B36864	-1.54	-0.072	-2.5
12	0	ME7S2	B36865	-0.74	0.098	-2.6
13	0	ME7S2	B36866	-1.16	0.205	-2.1
14	0	ME7S2	B36868	-1.18	0.182	-1.8
1	1	Normal S2	B34033	0.00	-1.164	-2.5
2	1	Normal S2	B34034	0.00	-1.195	-2.4
3	1	Normal S2	B34035	-1.46	-1.944	-2.6
8	1	Normal S2	B36860	-0.62	-1.017	-3.7
9	1	Normal S2	B36861	-0.66	-0.900	-3.9
10	1	Normal S2	B36862	-1.82	-1.859	-3.4

Boxplot of significant proteins

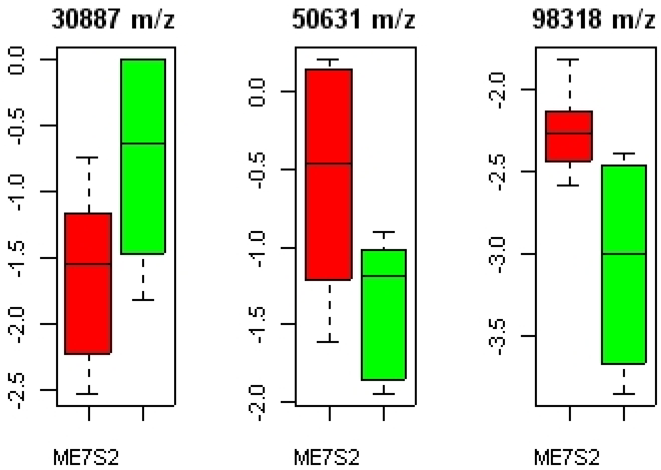

Boxplot of significant proteins

Pairwise Scatterplots of Significant Proteins

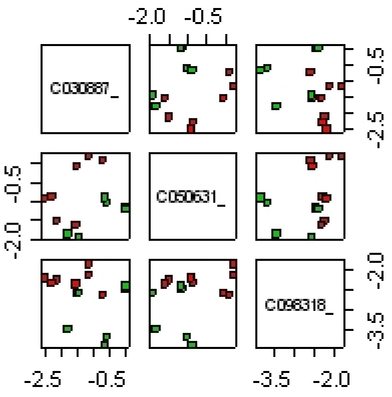

Pairwise Scatterplots of Significant Proteins

Cluster Analysis of samples (Euclidean distance)

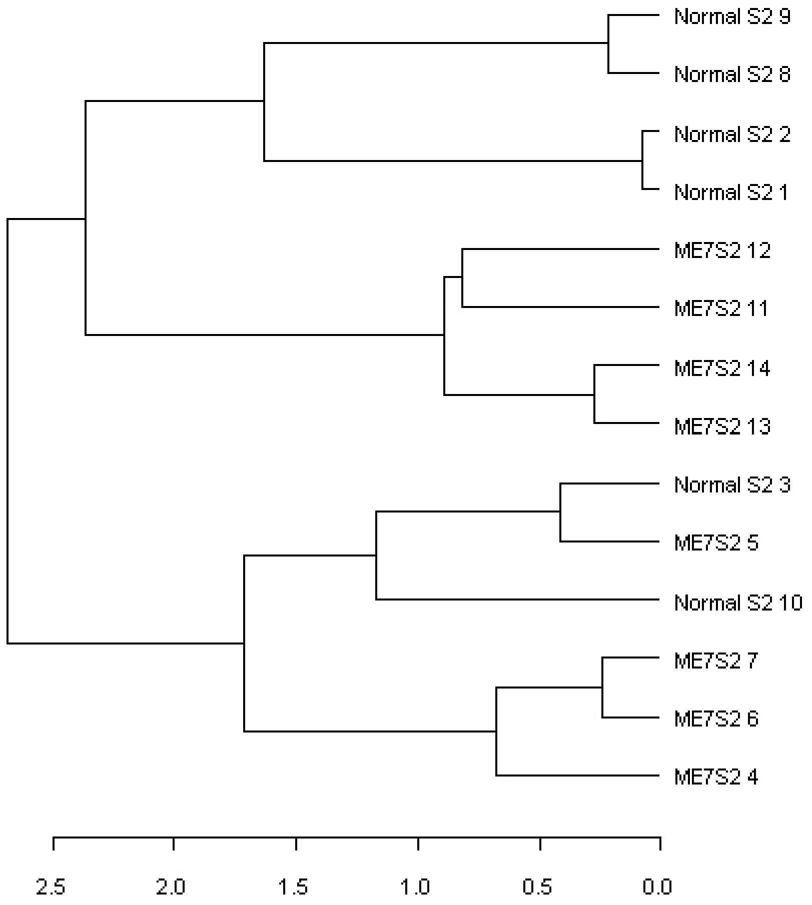

Cluster Analysis of samples (Euclidean distance)

Scatterplot of linear discriminant function (x-axis)

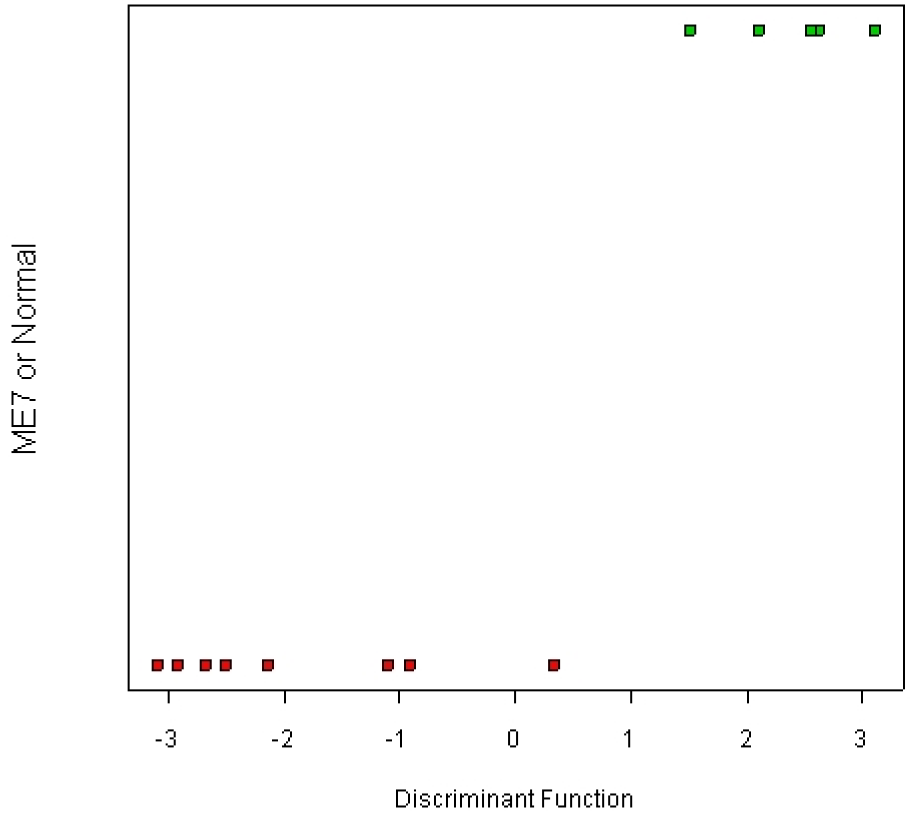

Scatterplot of linear discriminant function (x-axis)

All data

Boxplot of all proteins

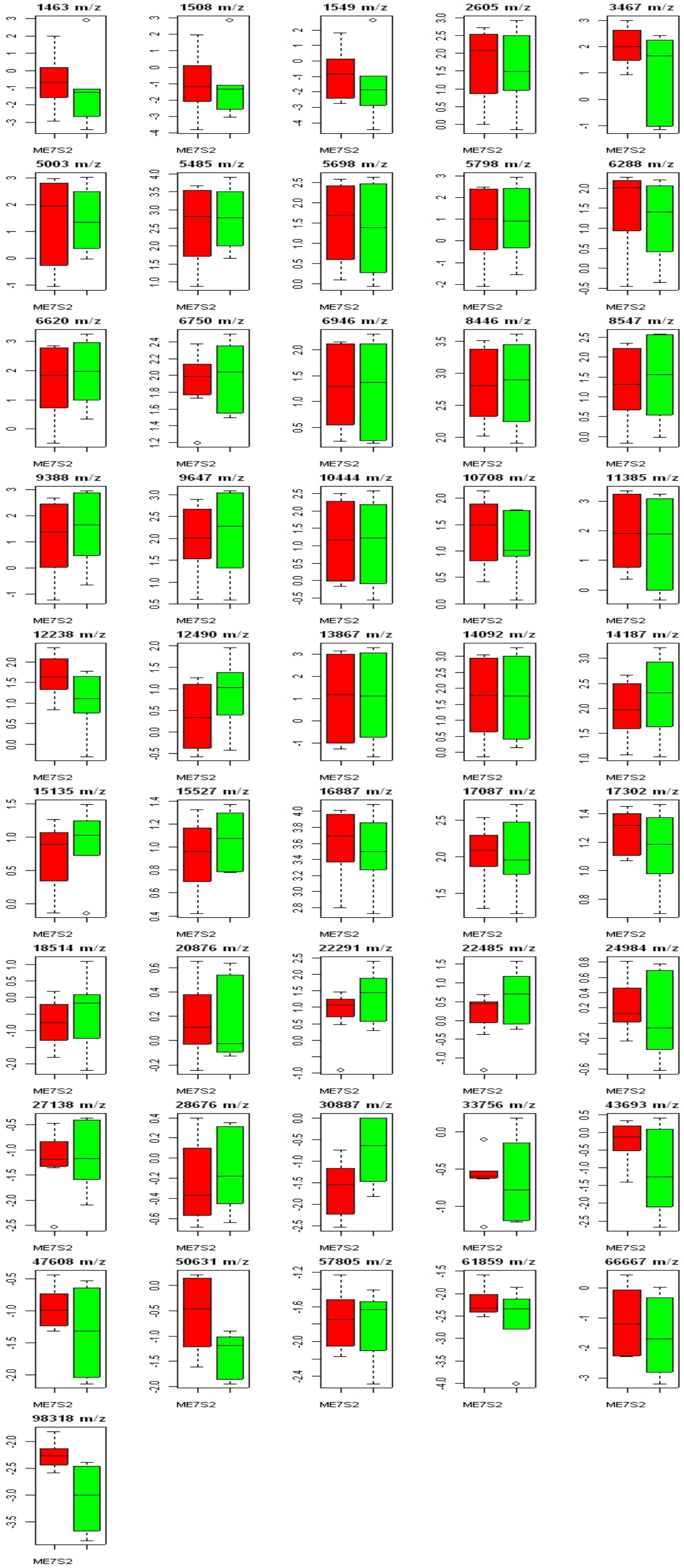

Boxplot of all proteins

Cluster Analysis of samples (Euclidean distance)

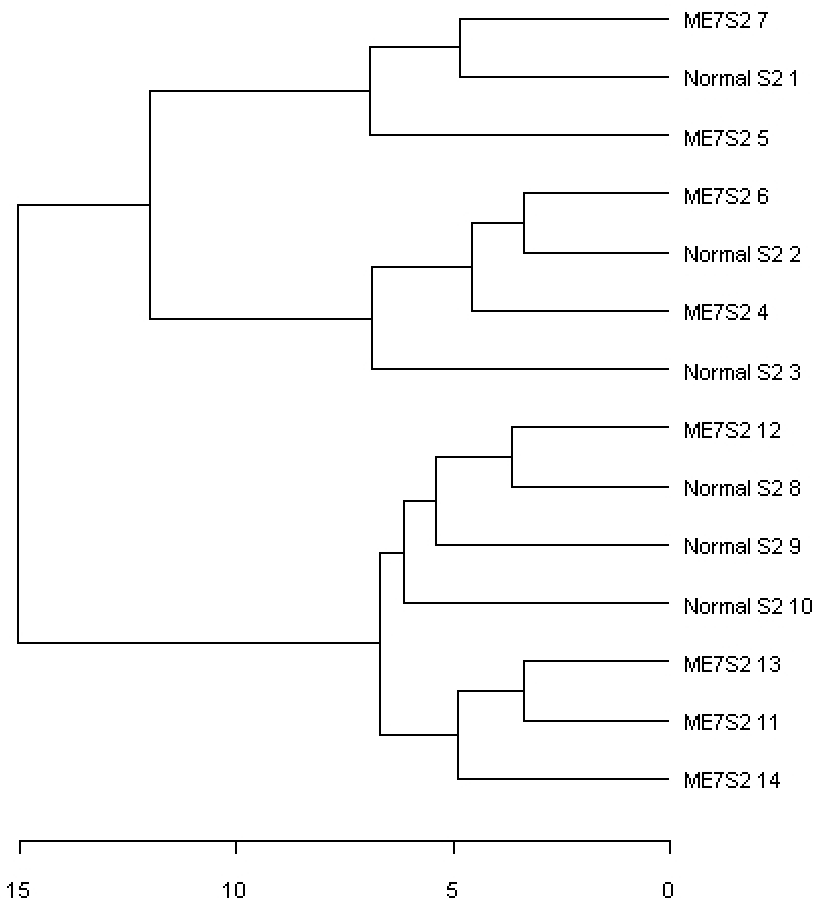

Cluster Analysis of samples (Euclidean distance)

S2 Q10 120

Proteins showing total separation

No proteins showed complete separation

Significant data (p<=0.05)

No Significant Proteins

Data for Significant proteins

	C0GROUP	C0GRP_NA	C0Spectr
5	0	ME7S2	B34615
6	0	ME7S2	B34616
7	0	ME7S2	B34620
12	0	ME7S2	B34615
13	0	ME7S2	B34616
14	0	ME7S2	B34620
1	1	Normal S2	B34611
2	1	Normal S2	B34612
3	1	Normal S2	B34613
4	1	Normal S2	B34614
8	1	Normal S2	B34611
9	1	Normal S2	B34612
10	1	Normal S2	B34613
11	1	Normal S2	B34614

All data

Boxplot of all proteins

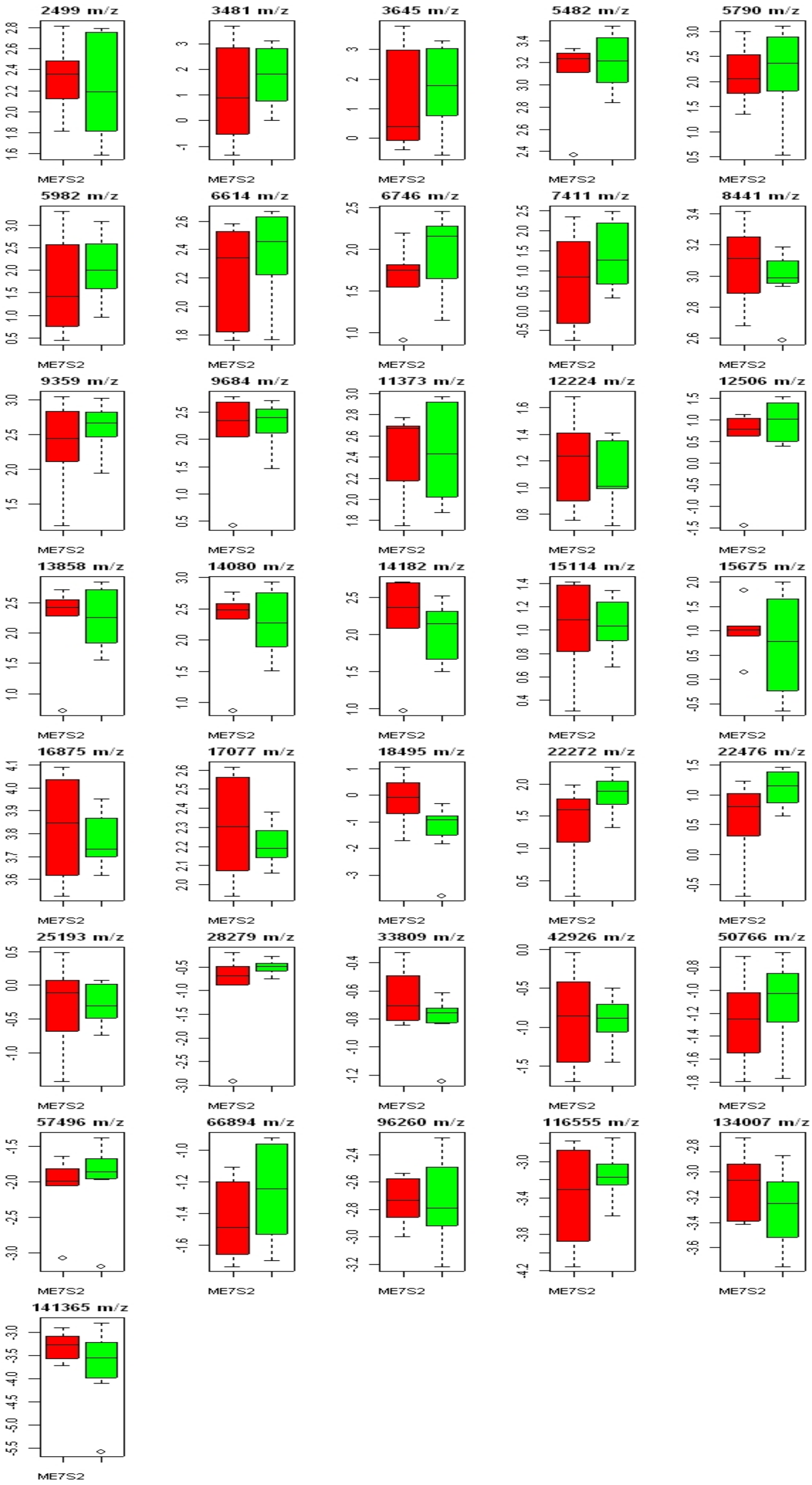

Boxplot of all proteins

Cluster Analysis of samples (Euclidean distance)

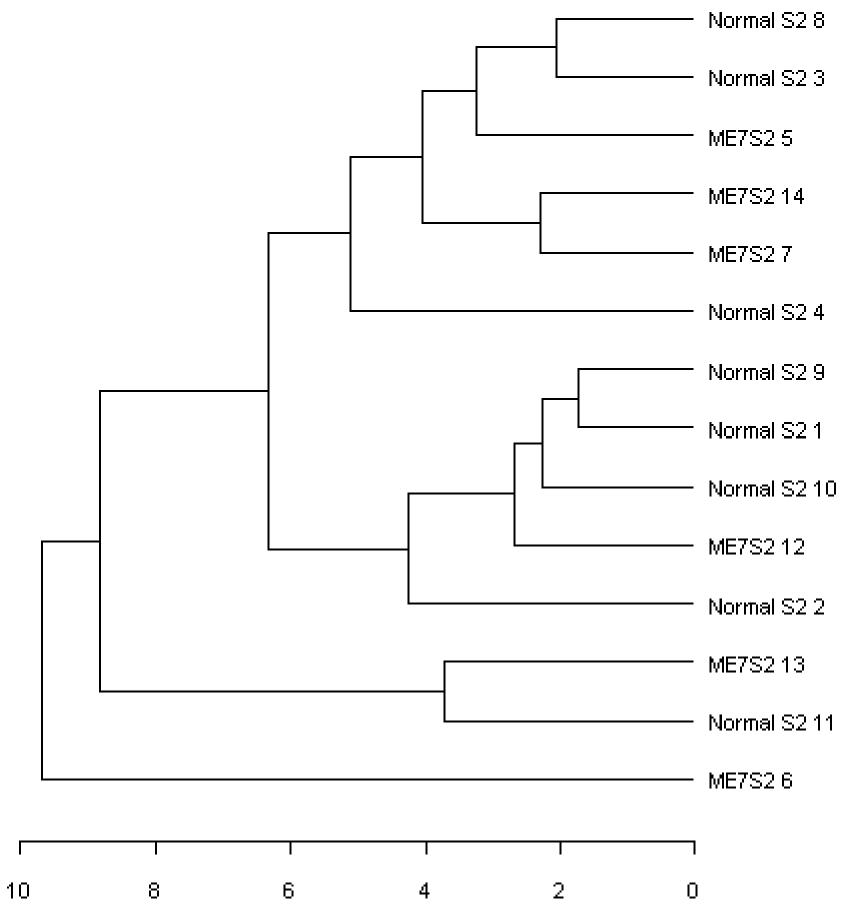

Cluster Analysis of samples (Euclidean distance)

S2 Q10 150

Proteins showing total separation

No proteins showed complete separation

Significant data (p<=0.05)

Significant Proteins (t-test; p<=0.05)

	name	mz	ME7.avg	NORM.avg	t	p
5	C05790_6	5790	2.898	2.58	2.6	0.027
16	C011264_	11264	2.617	2.26	2.9	0.014
27	C016880_	16880	4.205	4.02	2.2	0.045
28	C017084_	17084	2.624	2.34	2.3	0.042
30	C018507_	18507	-0.102	0.61	-2.6	0.026
38	C033749_	33749	0.017	-0.42	2.6	0.025

Data for Significant proteins

	C0GROUP	C0GRP_NA	C0Spectr	C05790_6	C011264_	C016880_	C017084_	C018507_	C033749_
5	0	ME7S2	B35183	3.1	2.7	4.1	2.3	-1.05	-0.397
6	0	ME7S2	B35184	2.9	2.6	4.2	2.9	0.25	0.167
7	0	ME7S2	B35185	2.8	2.3	4.1	2.4	-0.43	-0.087
11	0	ME7S2	B35179	3.1	3.1	4.3	2.7	-0.24	0.047
12	0	ME7S2	B35180	2.9	2.6	4.1	2.3	-0.27	-0.304
13	0	ME7S2	B35181	2.8	2.4	4.4	2.8	0.43	0.107
14	0	ME7S2	B35182	2.6	2.6	4.4	2.9	0.60	0.586
1	1	Normal S2	B35173	2.6	2.4	4.0	2.3	1.18	-0.624
2	1	Normal S2	B35174	2.7	2.6	3.7	1.9	0.27	-0.990
3	1	Normal S2	B35175	2.9	2.4	4.2	2.6	0.55	-0.137
4	1	Normal S2	B35177	2.6	2.1	4.1	2.5	0.40	-0.180
8	1	Normal S2	B35176	2.7	2.0	4.0	2.4	-0.10	-0.211
9	1	Normal S2	B35177	2.5	2.1	4.1	2.4	0.95	-0.477
10	1	Normal S2	B35178	2.0	2.1	4.0	2.2	1.01	-0.295

Boxplot of significant proteins

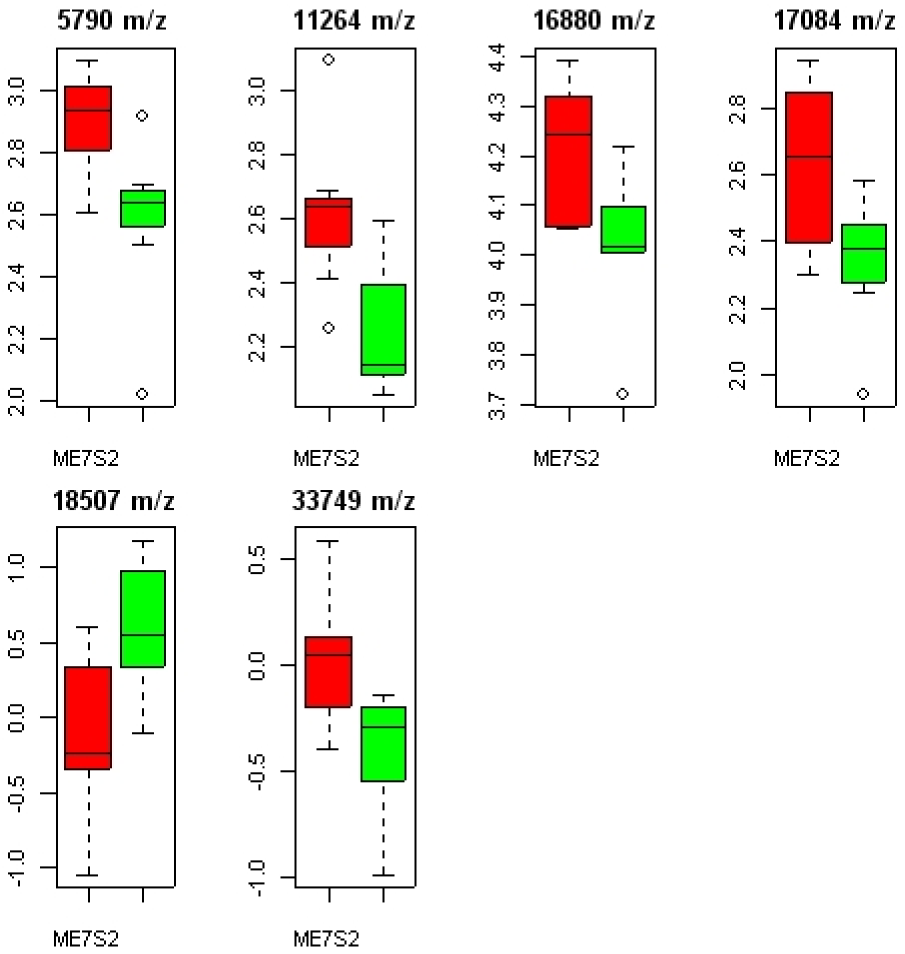

Boxplot of significant proteins

Pairwise Scatterplots of Significant Proteins

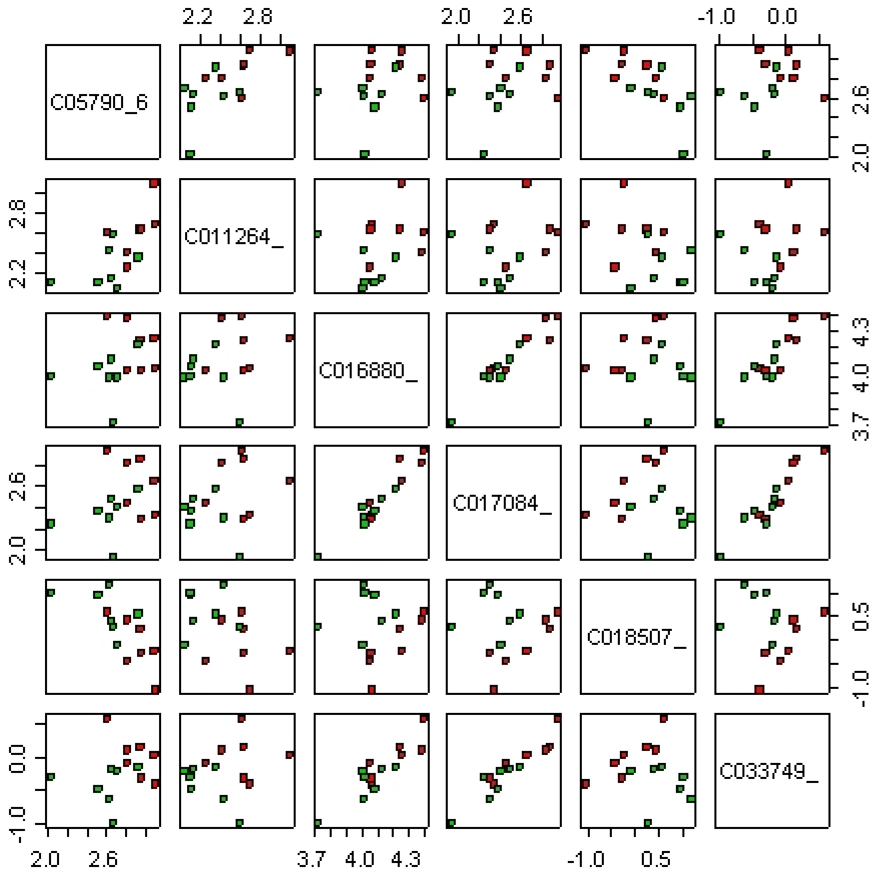

Pairwise Scatterplots of Significant Proteins

Cluster Analysis of samples (Euclidean distance)

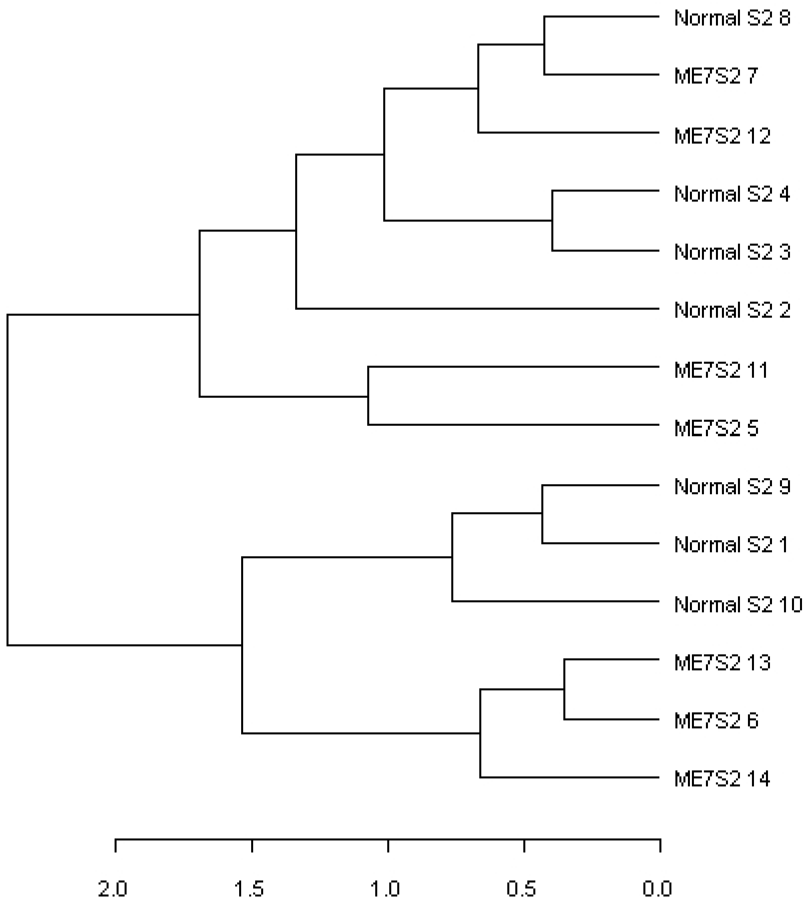

Cluster Analysis of samples (Euclidean distance)

Plot of first three principal components

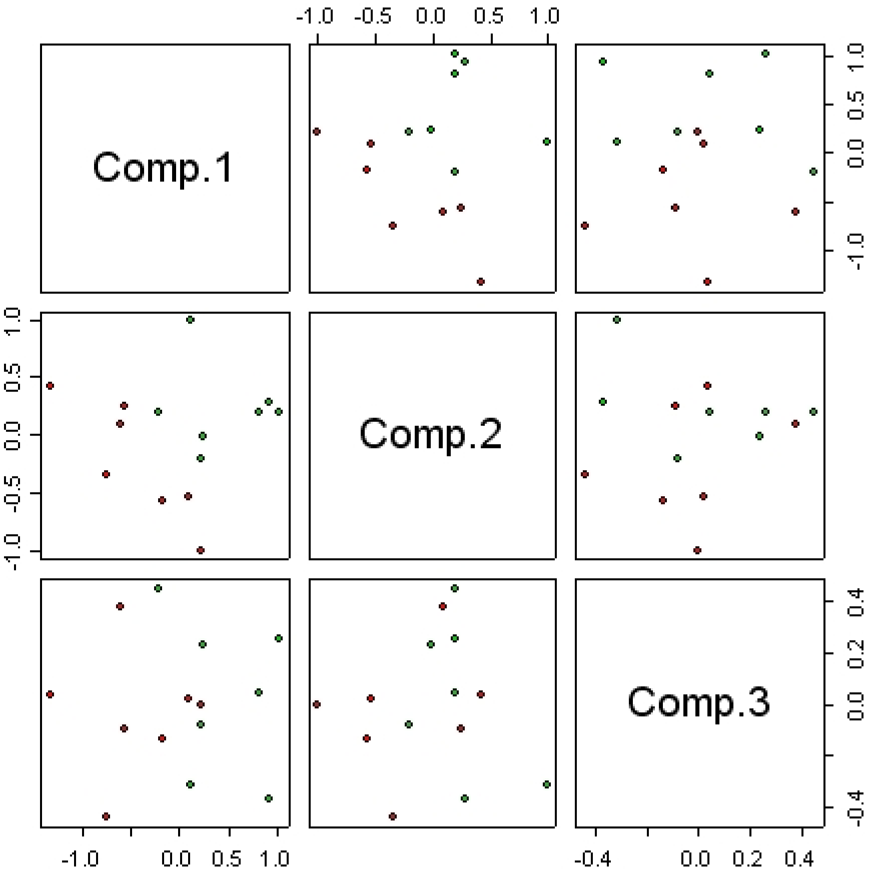

Plot of first three principal components

Scatterplot of linear discriminant function (x-axis)

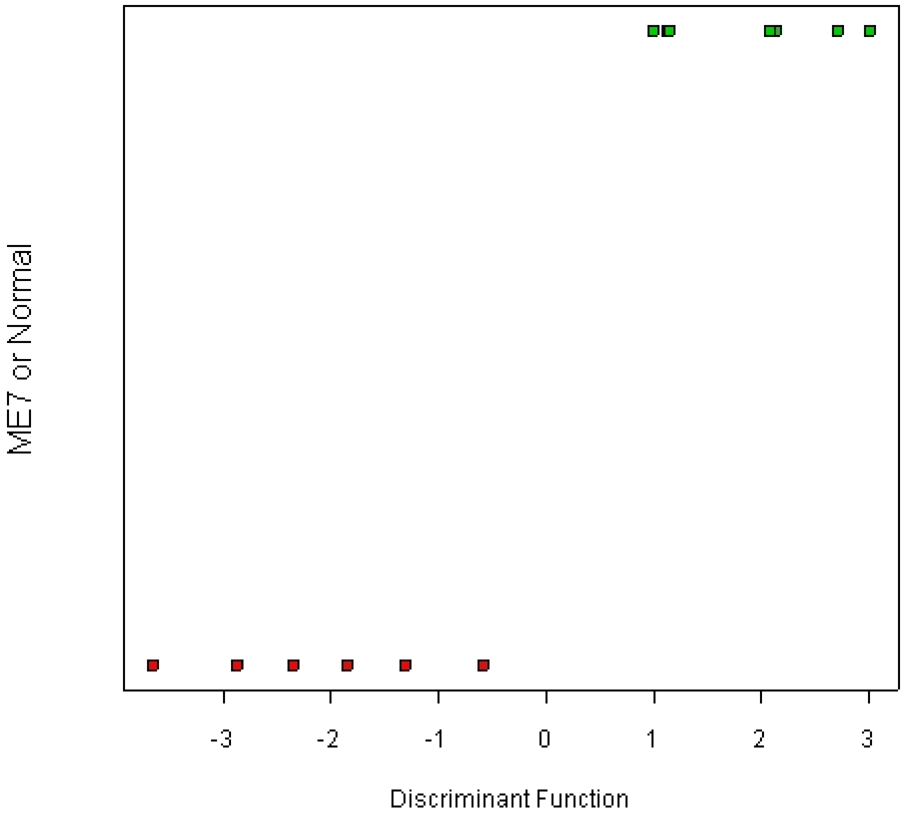

Scatterplot of linear discriminant function (x-axis)

All data

Boxplot of all proteins

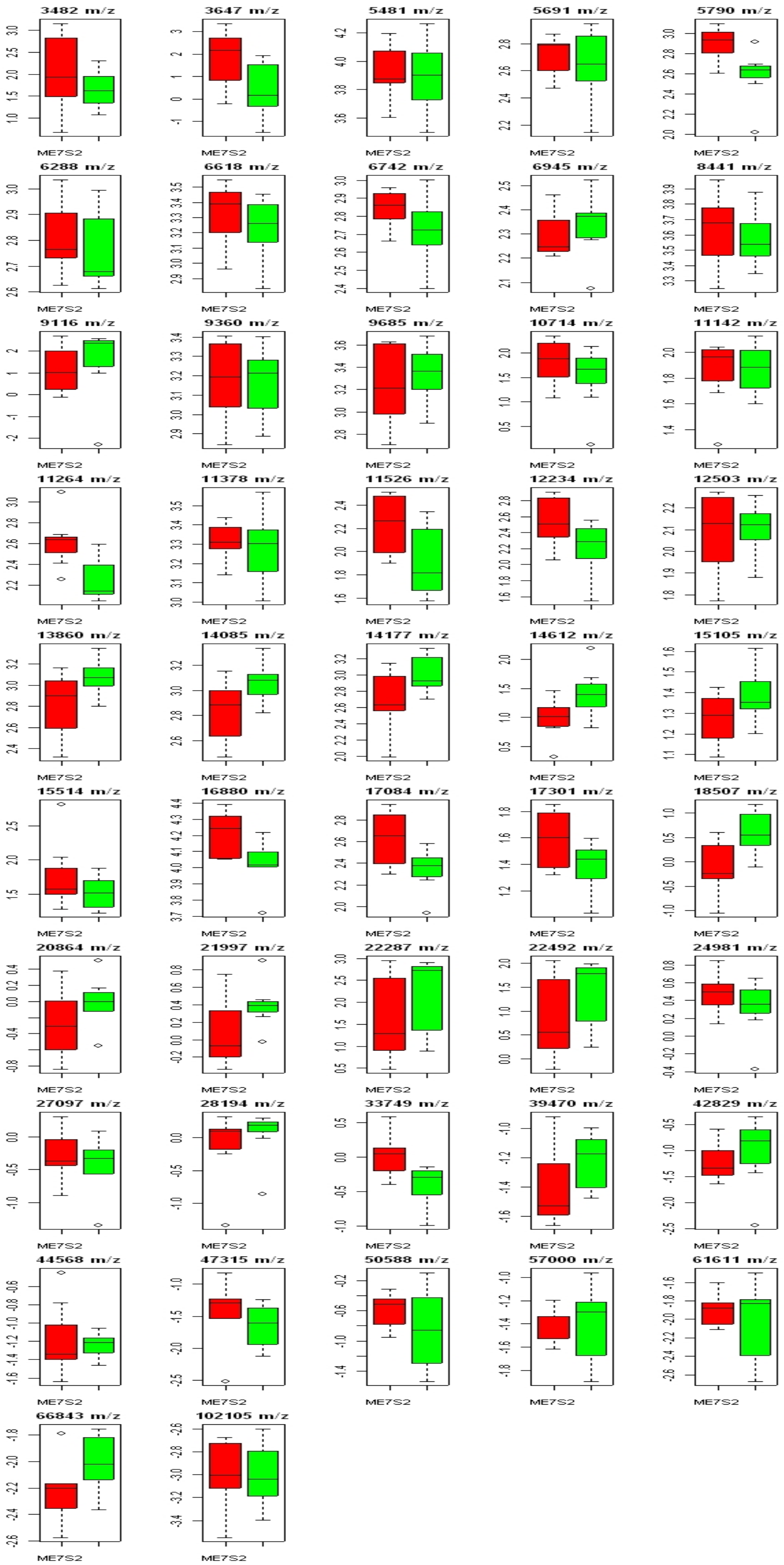

Boxplot of all proteins

Cluster Analysis of samples (Euclidean distance)

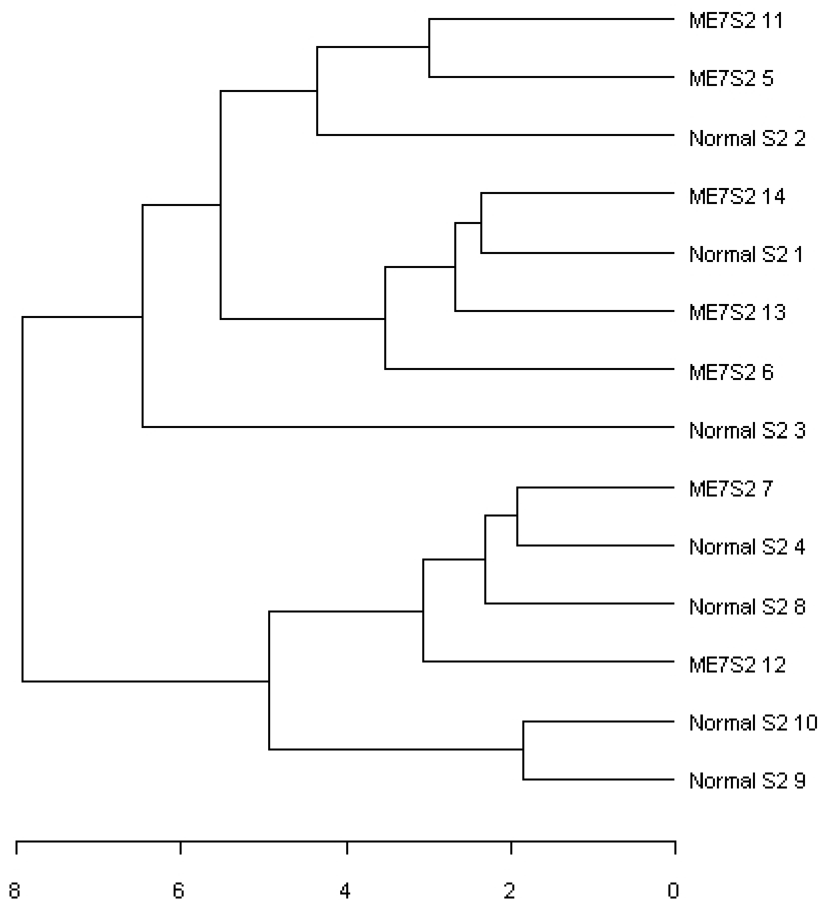

Cluster Analysis of samples (Euclidean distance)

S2 Q10 180

Proteins showing total separation

	C0GROUP	C0GRP_NA	C0Spectr	C025072_
5	0	ME7S2	B35772	-0.0143
6	0	ME7S2	B35773	-0.1265
10	0	ME7S2	B35770	-0.9801
11	0	ME7S2	B35771	-0.0076
12	0	ME7S2	B35774	-0.3779
13	0	ME7S2	B35775	-0.5185
14	0	ME7S2	B35770	-0.1039
1	1	Normal S2	B35764	0.0938
2	1	Normal S2	B35765	0.1196
3	1	Normal S2	B35767	0.1898
4	1	Normal S2	B35768	0.3905
7	1	Normal S2	B35766	0.0949
8	1	Normal S2	B35767	0.1528
9	1	Normal S2	B35769	0.0086

Boxplot of proteins showing complete separation

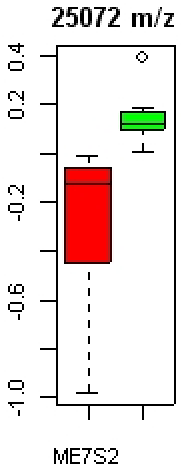

Significant data (p<=0.05)

Significant Proteins (t-test; p<=0.05)

	name	mz	ME7.avg	NORM.avg	t	p
3	C03643_6	3643	2.76	2.164	3.0	0.0126
26	C014633_	14633	0.78	1.643	-2.5	0.0451
38	C025072_	25072	-0.30	0.150	-3.2	0.0138
40	C028325_	28325	-0.45	0.018	-3.3	0.0100
42	C039479_	39479	-1.45	-0.969	-3.6	0.0039
47	C066930_	66930	-2.11	-1.725	-2.6	0.0255
49	C088134_	88134	-2.64	-3.227	2.2	0.0499

Data for Significant proteins

	C0GROUP	C0GRP_NA	C0Spectr	C03643_6	C014633_	C025072_	C028325_	C039479_	C066930_	C088134_
5	0	ME7S2	B35772	2.9	1.14	-0.0143	-0.112	-1.25	-2.1	-2.7
6	0	ME7S2	B35773	2.6	1.41	-0.1265	-0.100	-1.06	-2.3	-3.2
10	0	ME7S2	B35770	3.1	-1.23	-0.9801	-0.984	-1.66	-1.6	-2.0
11	0	ME7S2	B35771	2.4	0.98	-0.0076	-0.309	-1.17	-2.2	-2.1
12	0	ME7S2	B35774	2.9	1.09	-0.3779	-0.592	-1.54	-1.8	-2.5
13	0	ME7S2	B35775	3.0	1.12	-0.5185	-0.795	-1.72	-2.3	-3.1
14	0	ME7S2	B35770	2.4	0.97	-0.1039	-0.289	-1.73	-2.5	-2.9
1	1	Normal S2	B35764	2.2	1.60	0.0938	0.065	-1.00	-2.0	-3.0
2	1	Normal S2	B35765	2.6	1.36	0.1196	-0.105	-1.20	-1.8	-2.8
3	1	Normal S2	B35767	2.1	1.72	0.1898	0.053	-0.69	-1.7	-3.4
4	1	Normal S2	B35768	1.5	1.77	0.3905	0.220	-0.86	-1.7	-2.6
7	1	Normal S2	B35766	2.2	1.78	0.0949	0.016	-0.85	-1.6	-3.5
8	1	Normal S2	B35767	1.8	1.89	0.1528	0.128	-0.92	-2.0	-4.2
9	1	Normal S2	B35769	2.8	1.38	0.0086	-0.251	-1.27	-1.2	-3.0

Boxplot of significant proteins

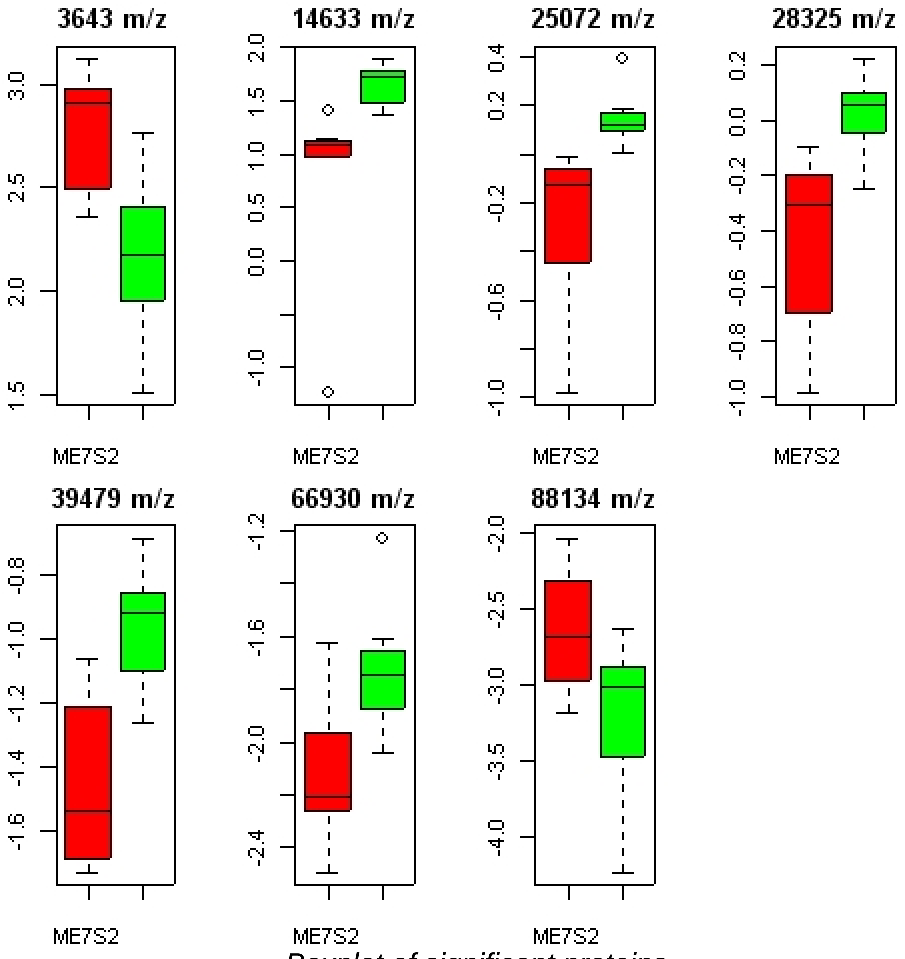

Boxplot of significant proteins

Pairwise Scatterplots of Significant Proteins

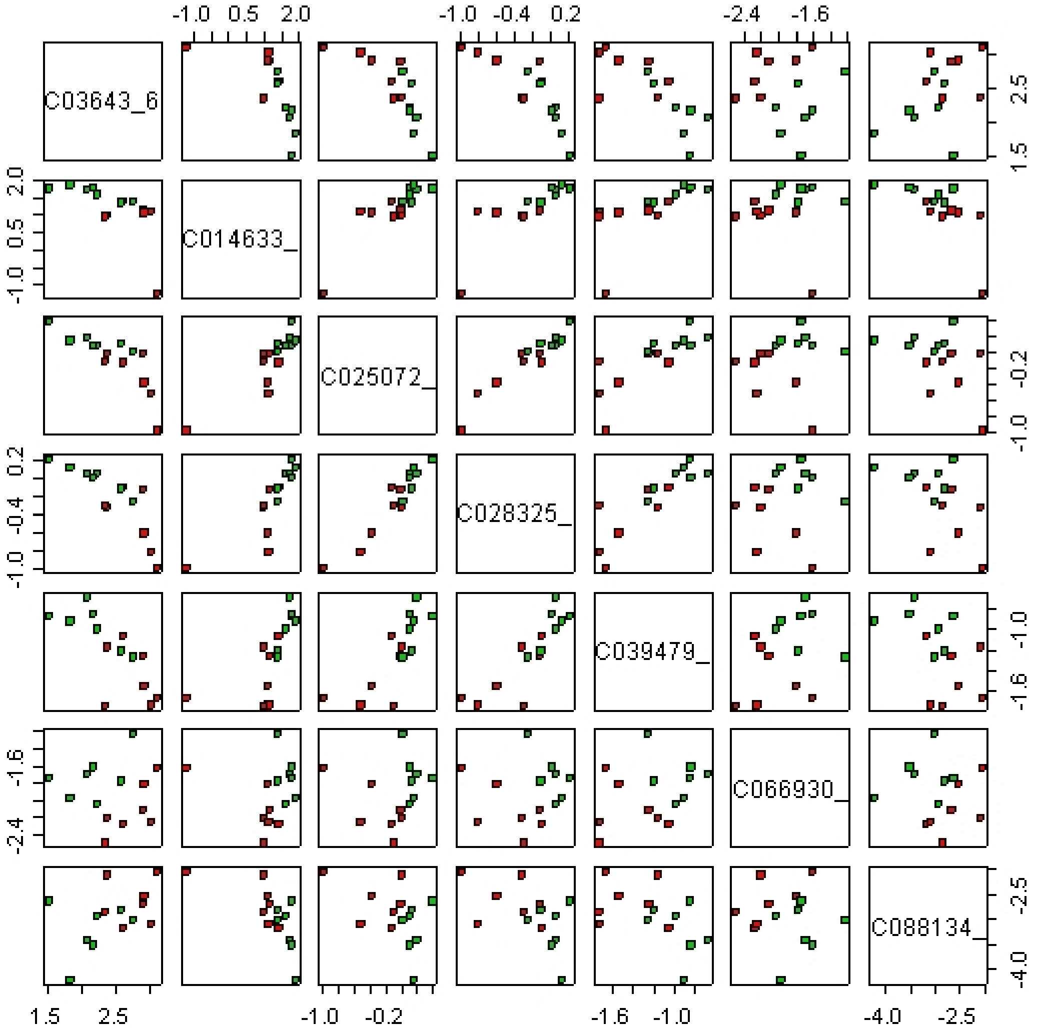

Pairwise Scatterplots of Significant Proteins

Cluster Analysis of samples (Euclidean distance)

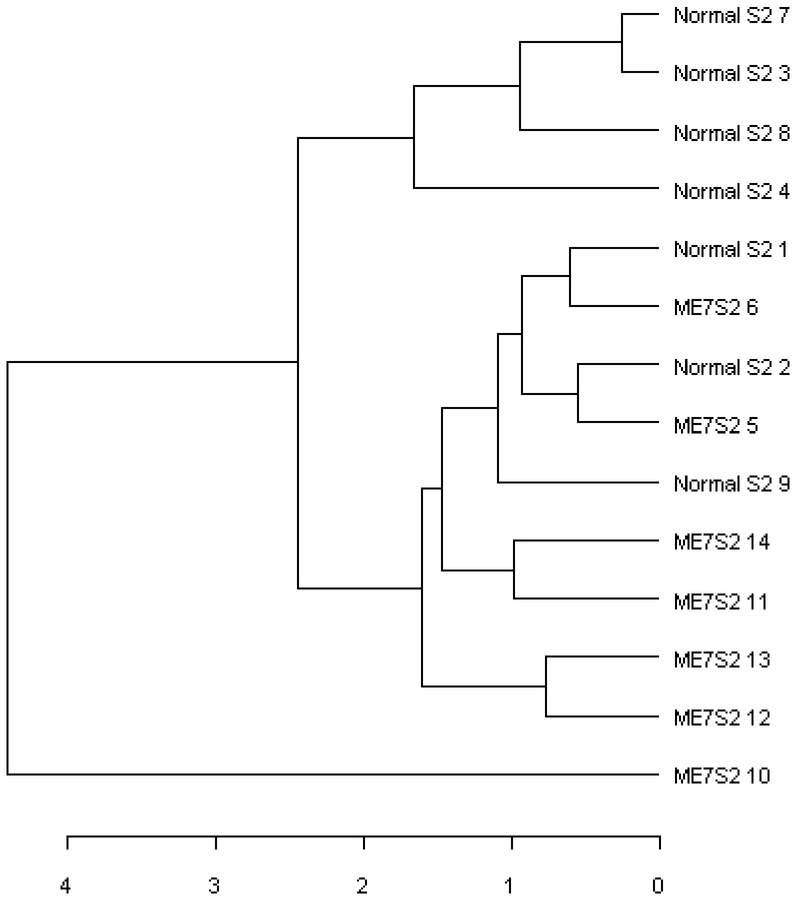

Cluster Analysis of samples (Euclidean distance)

Plot of first three principal components

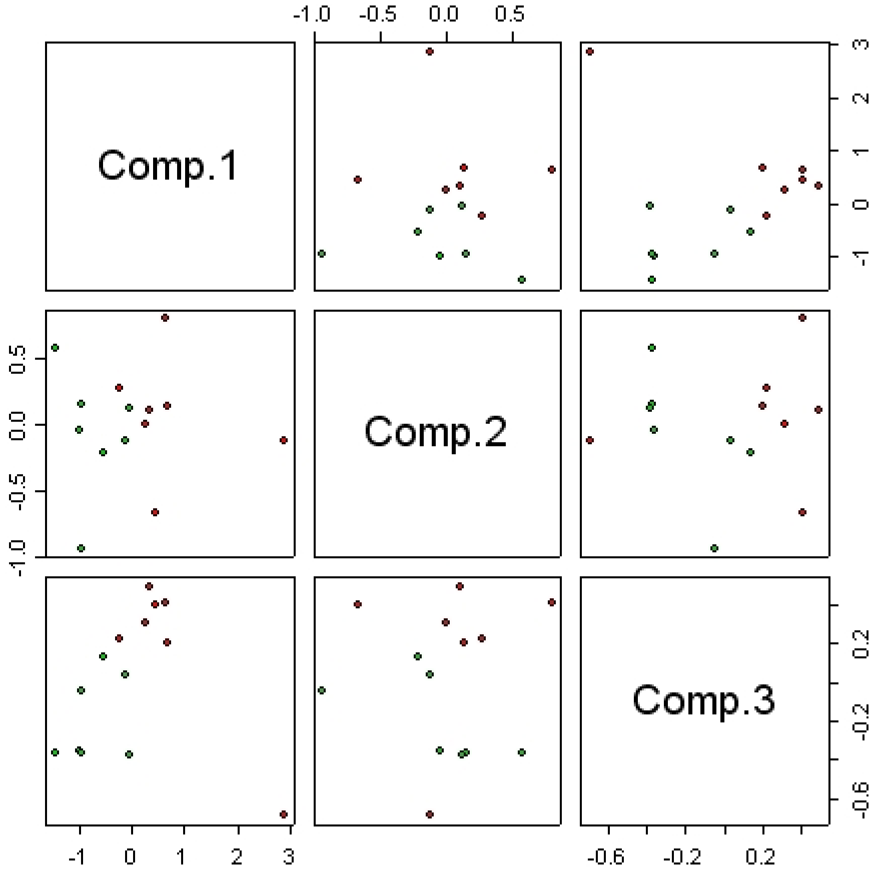

Plot of first three principal components

Scatterplot of linear discriminant function (x-axis)

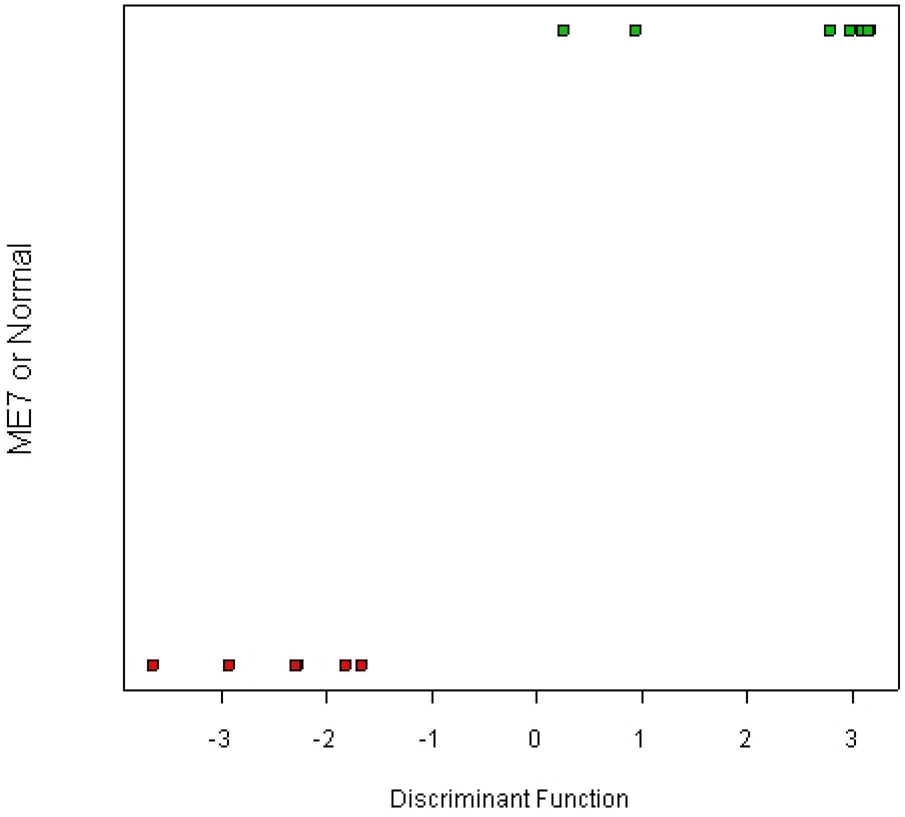

Scatterplot of linear discriminant function (x-axis)

All data

Boxplot of all proteins

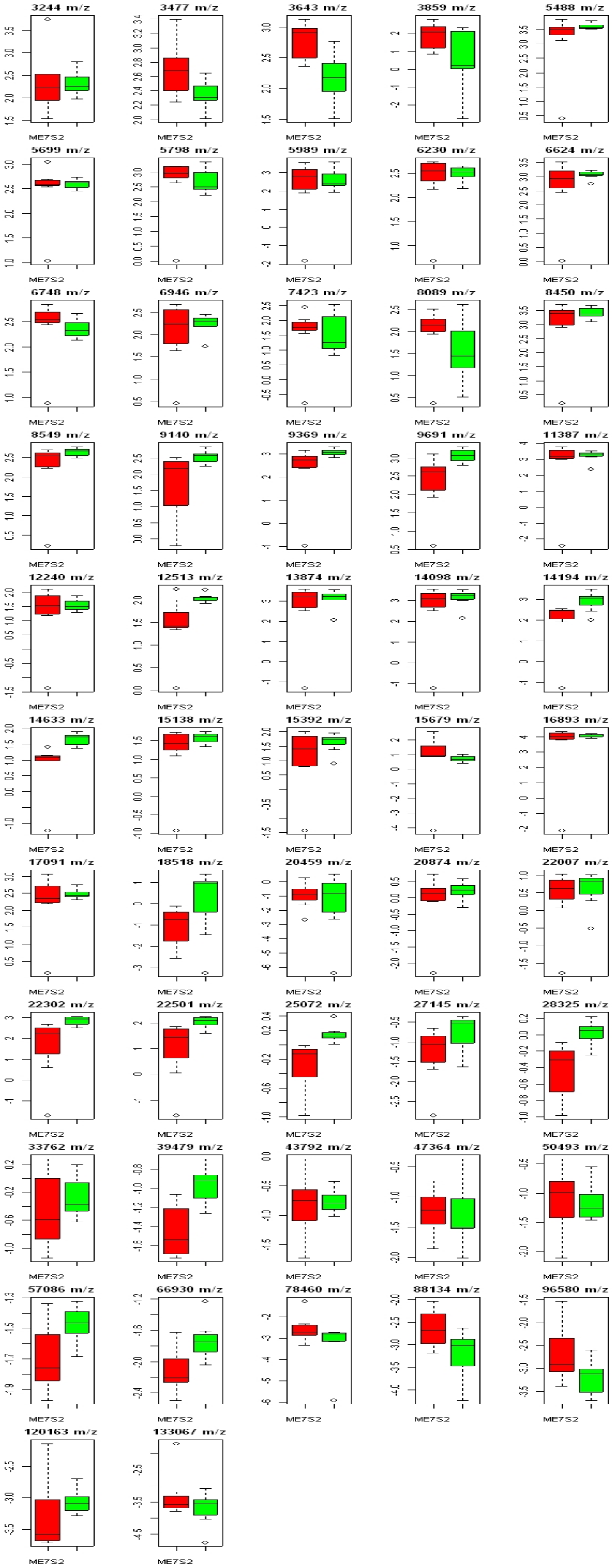

Boxplot of all proteins

Cluster Analysis of samples (Euclidean distance)

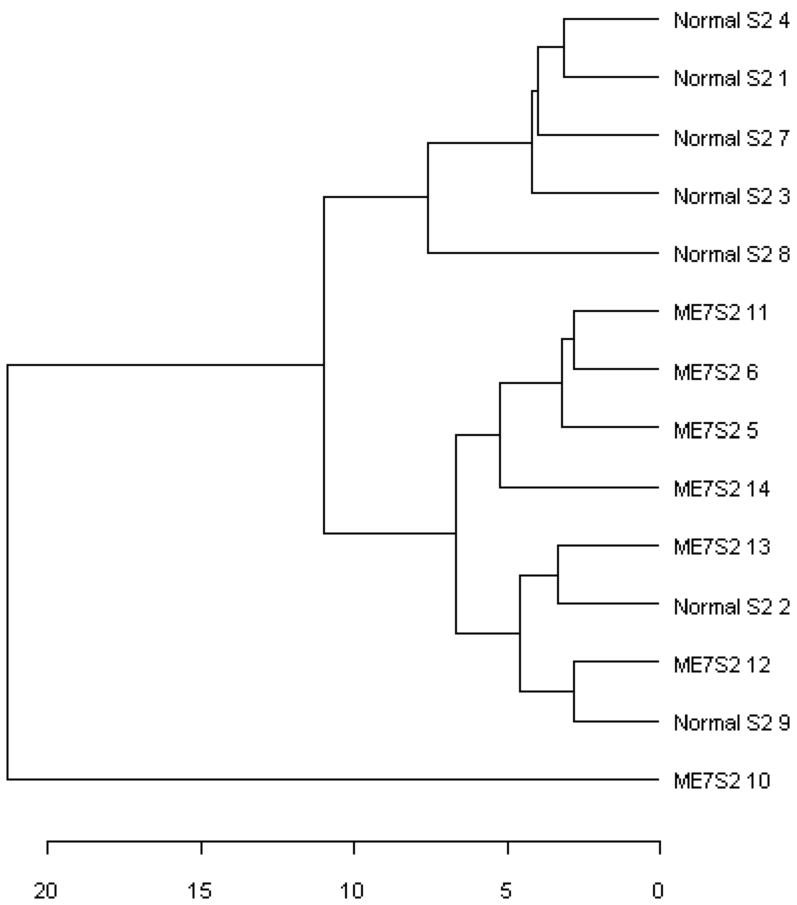

Cluster Analysis of samples (Euclidean distance)

S2 Q10 210

Proteins showing total separation

	C0GROUP	C0GRP_NA	C0Spectr	C010712_	C014625_	C022297_	C022496_	C050458_	C098034_
5	0	ME7S2	B36260	1.97	1.4	2.5	1.7	0.1023	-2.2
6	0	ME7S2	B36261	2.45	1.3	2.6	1.6	-0.0261	-2.1
7	0	ME7S2	B36265	2.19	1.5	2.4	1.6	0.8111	-1.4
11	0	ME7S2	B36262	2.01	1.5	2.3	1.6	0.0074	-2.2
12	0	ME7S2	B36263	2.56	1.3	2.5	1.6	0.5289	-1.5
13	0	ME7S2	B36264	1.91	1.5	2.2	1.4	0.0593	-2.3
1	1	Normal S2	B36248	0.64	1.8	2.9	2.1	-0.7045	-3.3
2	1	Normal S2	B36250	1.33	2.0	2.8	1.8	-1.4857	-2.7
3	1	Normal S2	B36252	0.77	1.9	3.0	2.2	-0.9005	-3.5
4	1	Normal S2	B36253	1.44	1.6	3.0	2.2	-1.3249	-2.7
8	1	Normal S2	B36249	1.26	1.9	2.6	1.7	-0.9447	-3.8
9	1	Normal S2	B36251	1.11	1.9	2.7	2.0	-0.1492	-3.4
10	1	Normal S2	B36253	1.45	1.8	2.9	2.1	-0.6335	-2.9

Boxplot of proteins showing complete separation

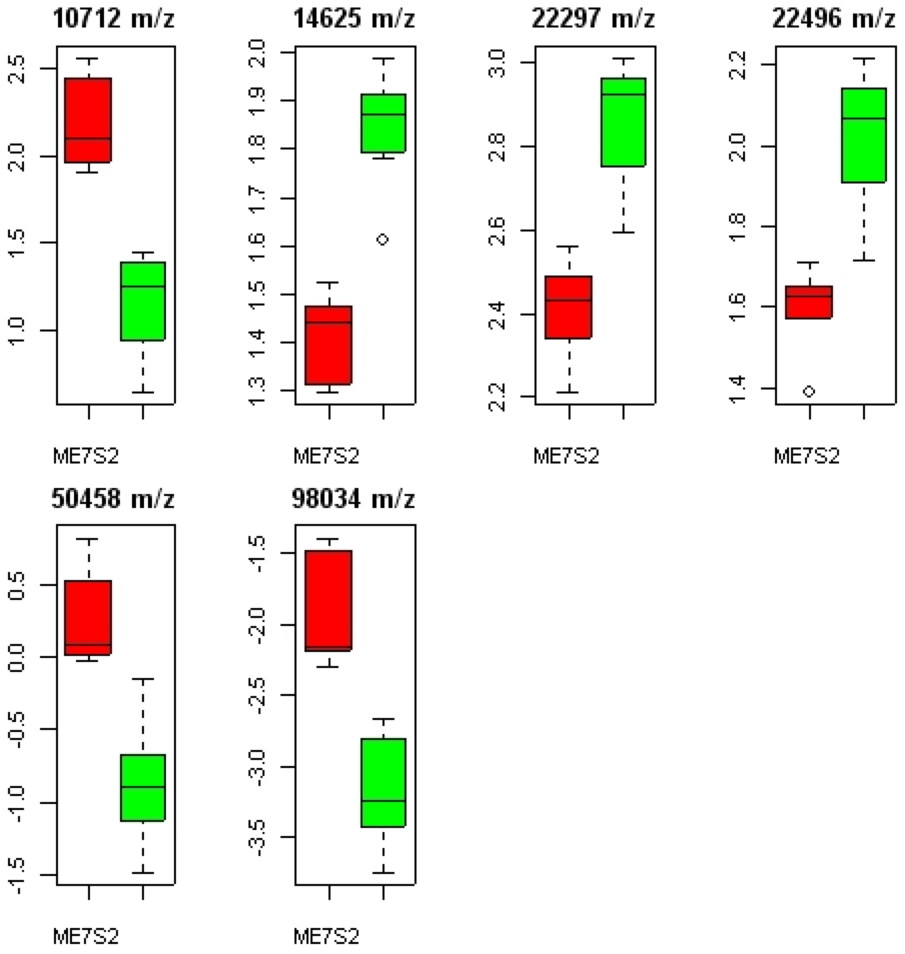

Pairwise Scatterplots of Proteins showing complete separation

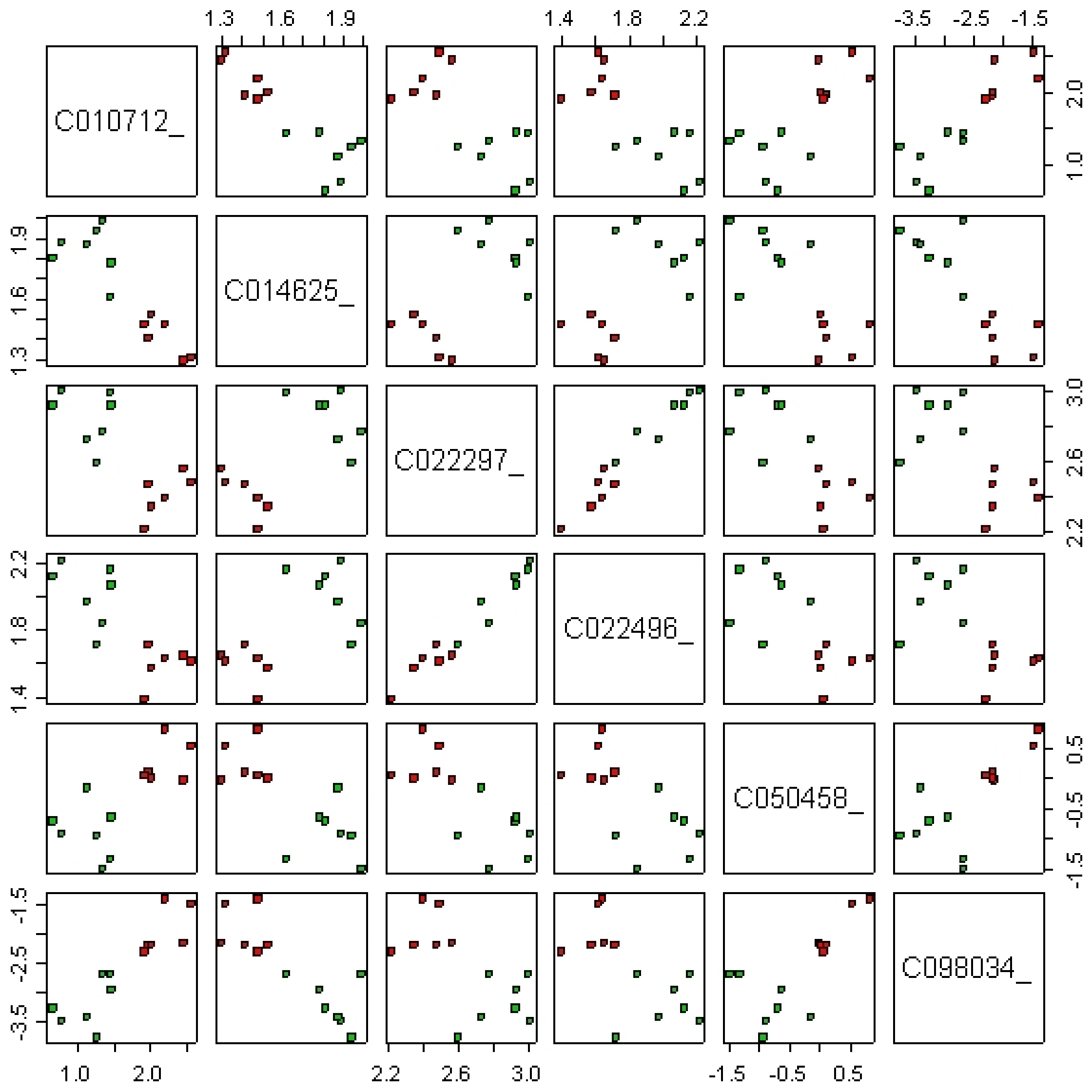

Significant data (p<=0.05)

Significant Proteins (t-test; p<=0.05)

	name	mz	ME7.avg	NORM.avg	t	p
13	C010712_	10712	2.18	1.14	6.3	5.8e-05
14	C011145_	11145	1.55	1.96	-4.4	1.0e-03
15	C011382_	11382	3.49	3.08	2.3	4.4e-02
22	C014625_	14625	1.42	1.84	-7.1	2.2e-05
26	C017091_	17091	2.21	2.51	-2.8	1.6e-02
29	C022297_	22297	2.41	2.85	-5.7	1.4e-04
30	C022496_	22496	1.60	2.01	-5.1	4.5e-04
36	C042895_	42895	-0.10	-0.58	2.3	4.1e-02
37	C050458_	50458	0.25	-0.88	5.1	3.4e-04
39	C066563_	66563	-1.25	-1.71	3.1	1.4e-02
40	C098034_	98034	-1.95	-3.17	5.4	2.2e-04

Data for Significant proteins

	C0GROUP	C0GRP_NA	C0Spectr	C010712_	C011145_	C011382_	C014625_	C017091_	C022297_	C022496_	C042895_	C050458_	C066563_	C098034_
5	0	ME7S2	B36260	1.97	1.6	3.7	1.4	2.1	2.5	1.7	-0.368	0.1023	-1.40	-2.2
6	0	ME7S2	B36261	2.45	1.8	3.4	1.3	2.5	2.6	1.6	-0.056	-0.0261	-0.90	-2.1
7	0	ME7S2	B36265	2.19	1.6	3.7	1.5	2.2	2.4	1.6	0.158	0.8111	-0.94	-1.4
11	0	ME7S2	B36262	2.01	1.5	3.6	1.5	2.0	2.3	1.6	-0.080	0.0074	-1.59	-2.2
12	0	ME7S2	B36263	2.56	1.5	2.9	1.3	2.3	2.5	1.6	0.230	0.5289	-1.06	-1.5
13	0	ME7S2	B36264	1.91	1.3	3.7	1.5	2.1	2.2	1.4	-0.503	0.0593	-1.59	-2.3
1	1	Normal S2	B36248	0.64	2.1	3.5	1.8	2.6	2.9	2.1	-0.875	-0.7045	-1.72	-3.3
2	1	Normal S2	B36250	1.33	1.9	3.2	2.0	2.3	2.8	1.8	-1.060	-1.4857	-1.84	-2.7
3	1	Normal S2	B36252	0.77	2.1	3.3	1.9	2.4	3.0	2.2	-0.779	-0.9005	-1.76	-3.5
4	1	Normal S2	B36253	1.44	2.1	2.6	1.6	2.5	3.0	2.2	-0.988	-1.3249	-1.97	-2.7
8	1	Normal S2	B36249	1.26	1.7	3.2	1.9	2.2	2.6	1.7	-0.280	-0.9447	-1.71	-3.8
9	1	Normal S2	B36251	1.11	1.8	3.1	1.9	2.7	2.7	2.0	0.070	-0.1492	-1.32	-3.4
10	1	Normal S2	B36253	1.45	2.0	2.7	1.8	2.8	2.9	2.1	-0.179	-0.6335	-1.68	-2.9

Boxplot of significant proteins

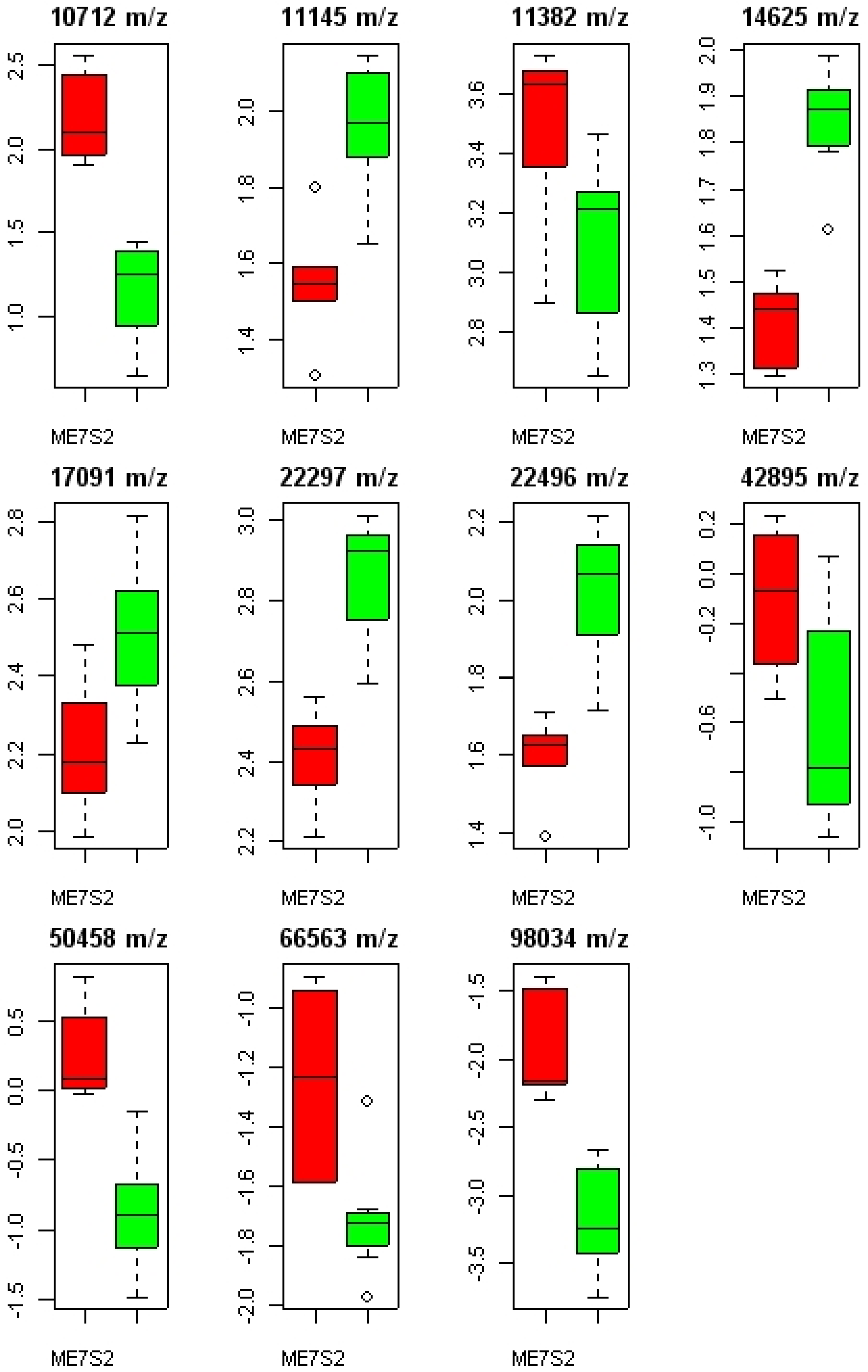

Boxplot of significant proteins

Pairwise Scatterplots of Significant Proteins

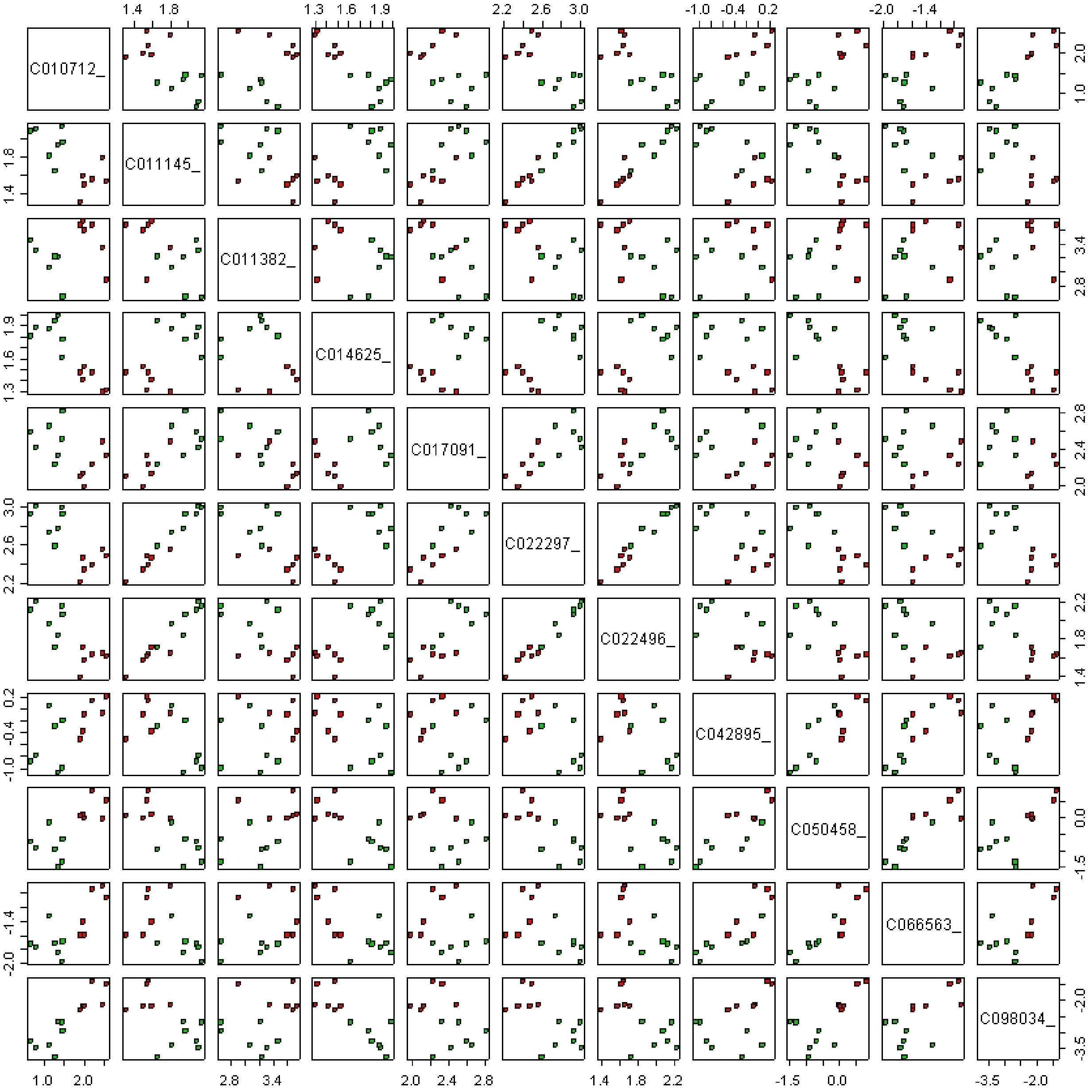

Pairwise Scatterplots of Significant Proteins

Cluster Analysis of samples (Euclidean distance)

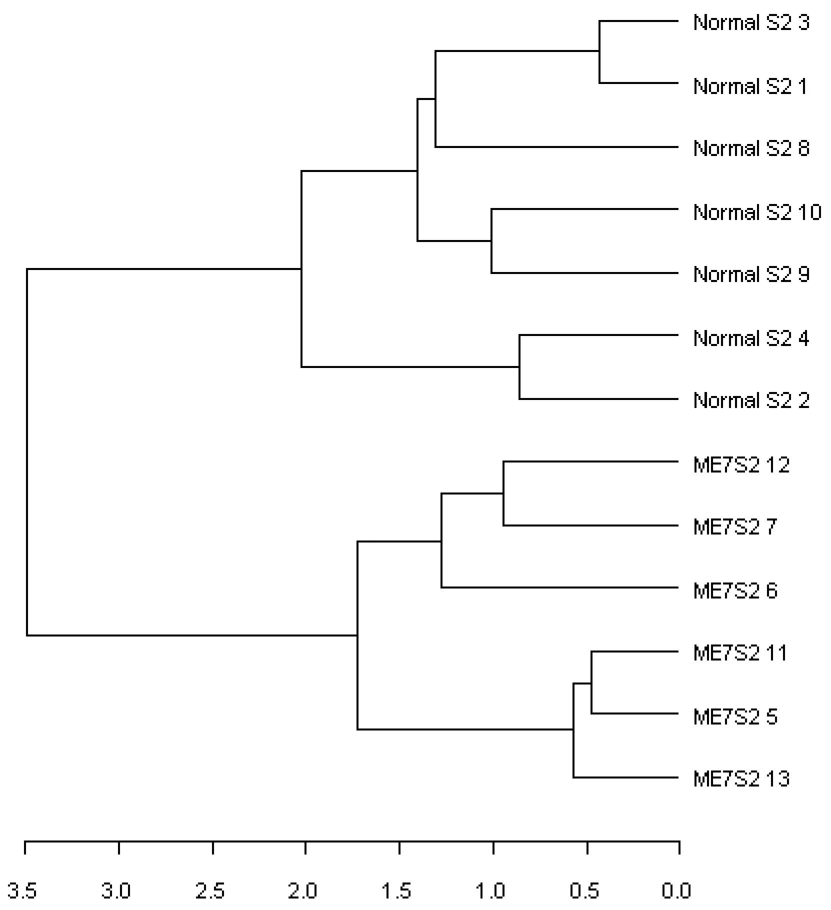

Cluster Analysis of samples (Euclidean distance)

Plot of first three principal components

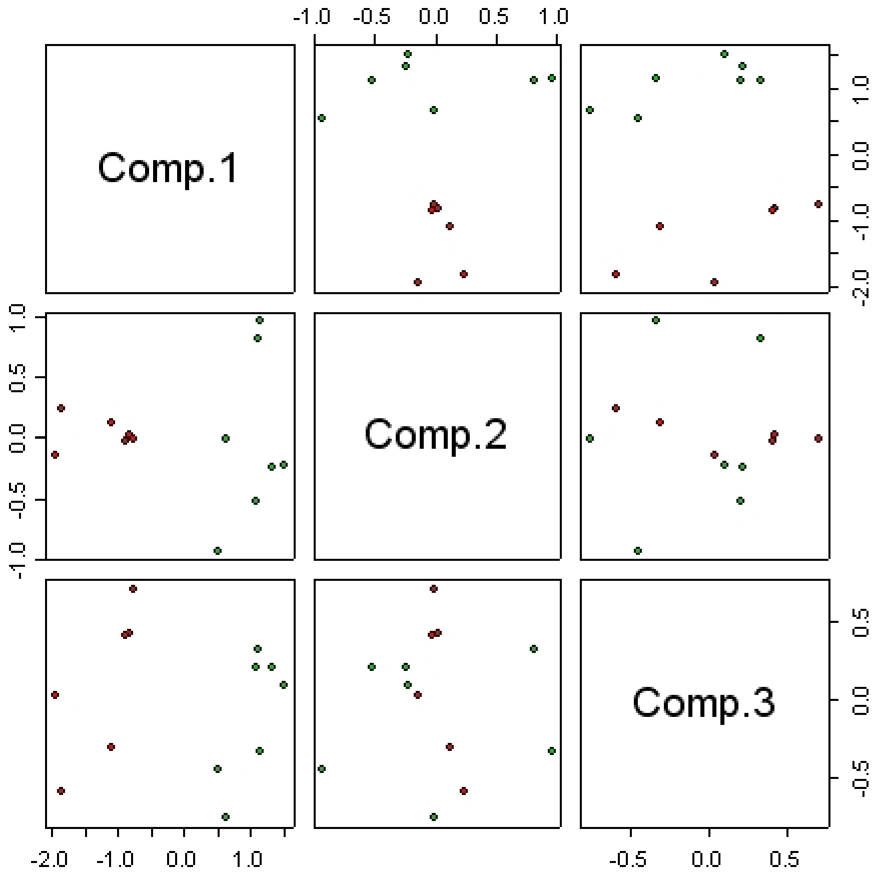

Plot of first three principal components

Scatterplot of linear discriminant function (x-axis)

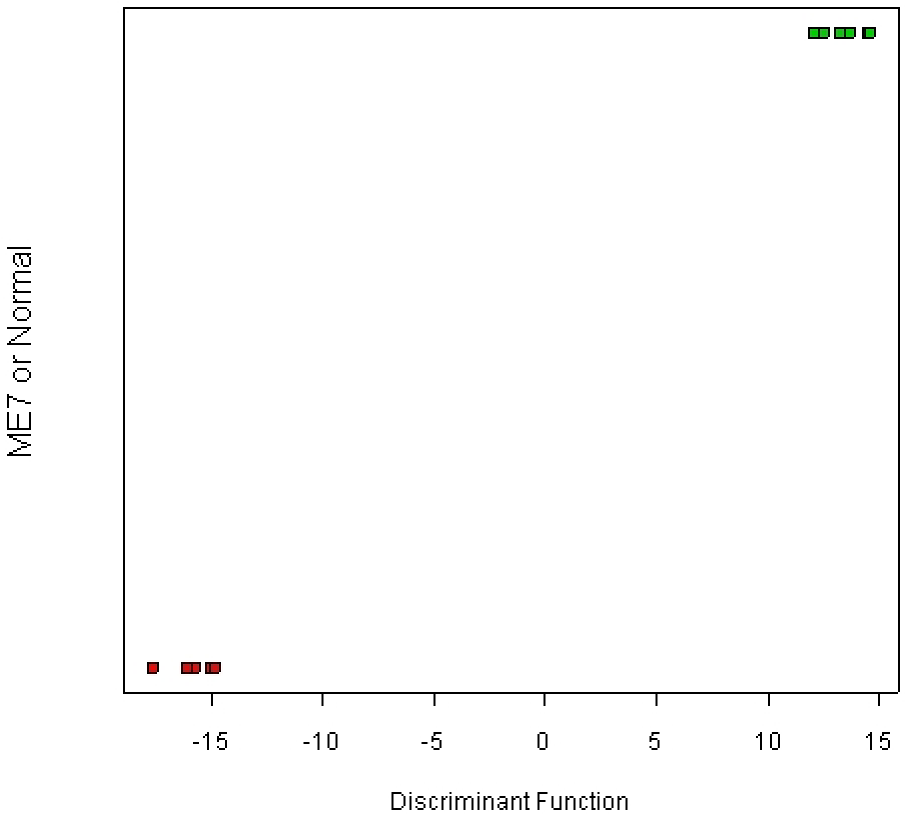

Scatterplot of linear discriminant function (x-axis)

All data

Boxplot of all proteins

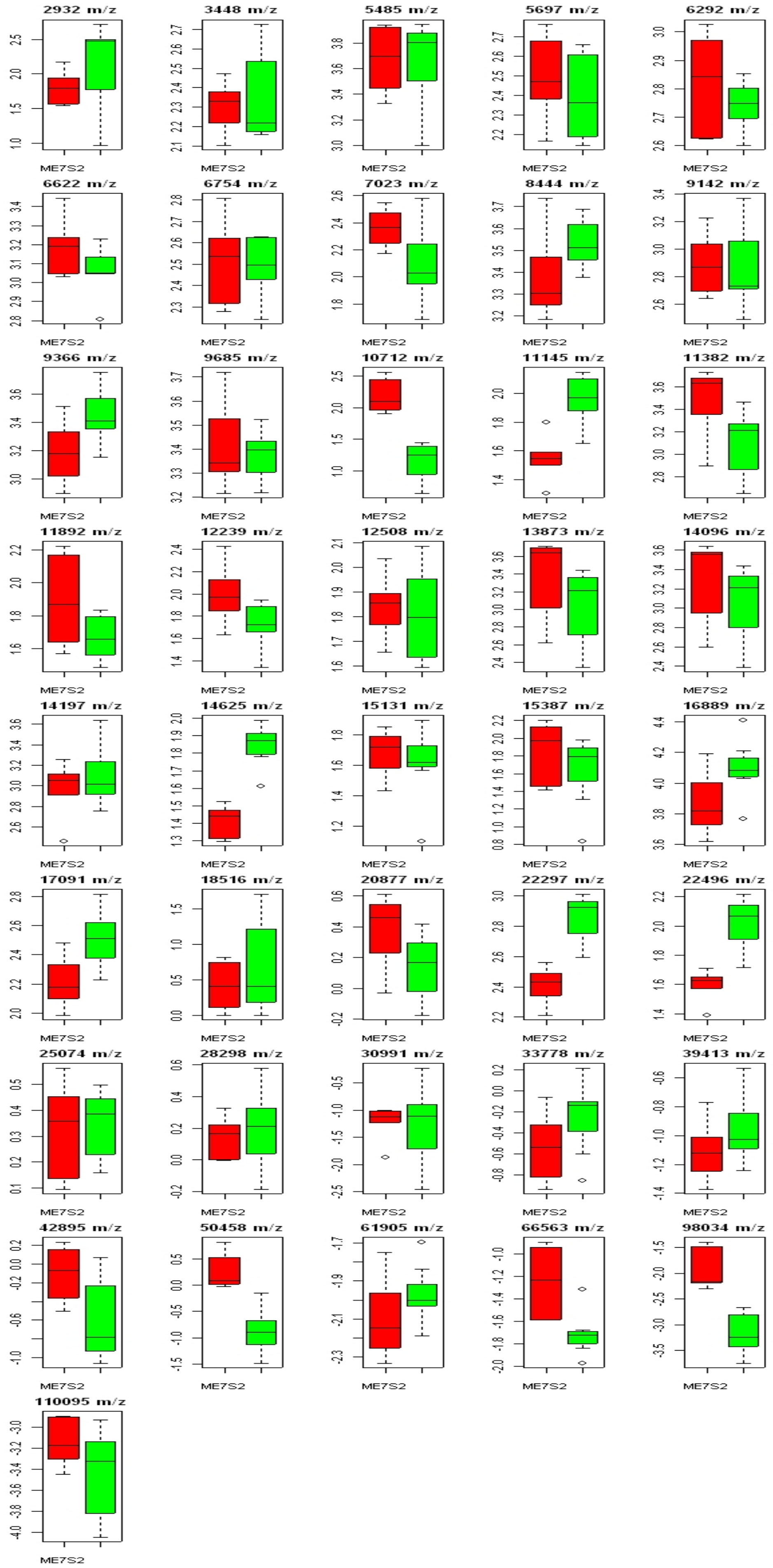

Boxplot of all proteins

Cluster Analysis of samples (Euclidean distance)

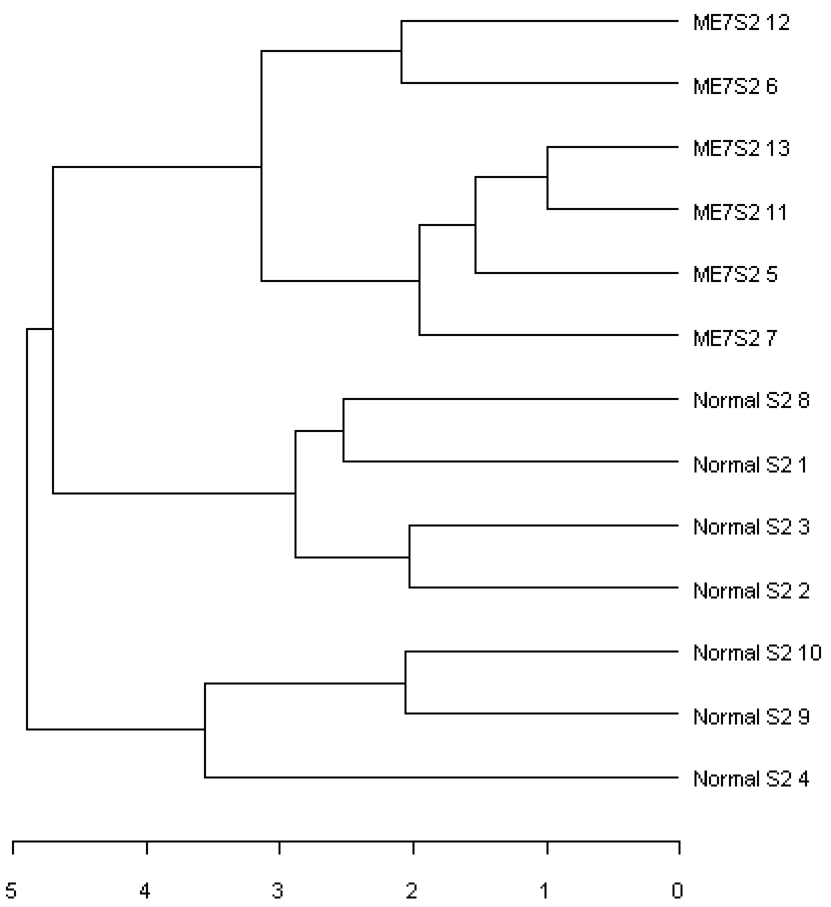

Cluster Analysis of samples (Euclidean distance)

S2 Q10 240

Proteins showing total separation

	C0GROUP	C0GRP_NA	C0Spectr	C044721_	C050485_	C067150_	C096118_	C0101150
5	0	ME7S2	B36863	-1.32	0.430	-2.2	-2.3	-2.3
6	0	ME7S2	B36865	-0.55	0.350	-2.1	-2.3	-2.3
7	0	ME7S2	B36867	0.77	0.308	-2.2	-1.6	-2.5
11	0	ME7S2	B36864	-0.12	0.117	-2.1	-2.4	-2.6
12	0	ME7S2	B36865	-0.44	0.287	-2.1	-2.4	-2.5
13	0	ME7S2	B36866	-1.21	0.395	-2.1	-2.2	-1.9
14	0	ME7S2	B36868	0.41	0.372	-1.9	-1.6	-2.1
1	1	Normal S2	B36857	-2.40	0.065	-2.5	-3.4	-3.8
2	1	Normal S2	B36858	-2.14	-1.079	-2.4	-2.9	-2.9
3	1	Normal S2	B36859	-1.66	-0.165	-2.6	-3.0	-3.3
4	1	Normal S2	B36860	-1.55	-0.641	-3.2	-3.1	-3.7
8	1	Normal S2	B36860	-2.15	-0.827	-2.6	-3.5	-3.5
9	1	Normal S2	B36861	-1.66	-0.711	-3.0	-3.7	-3.4
10	1	Normal S2	B36862	-1.86	-1.670	-2.3	-3.3	-3.0

Boxplot of proteins showing complete separation

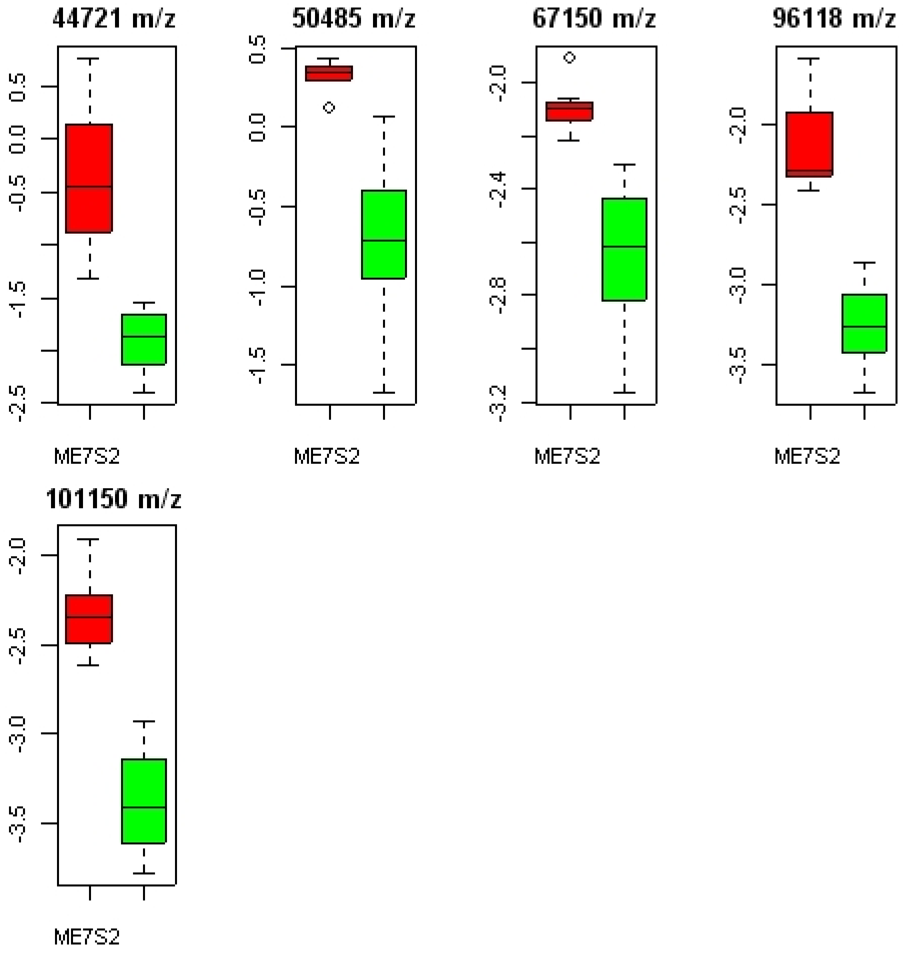

Pairwise Scatterplots of Proteins showing complete separation

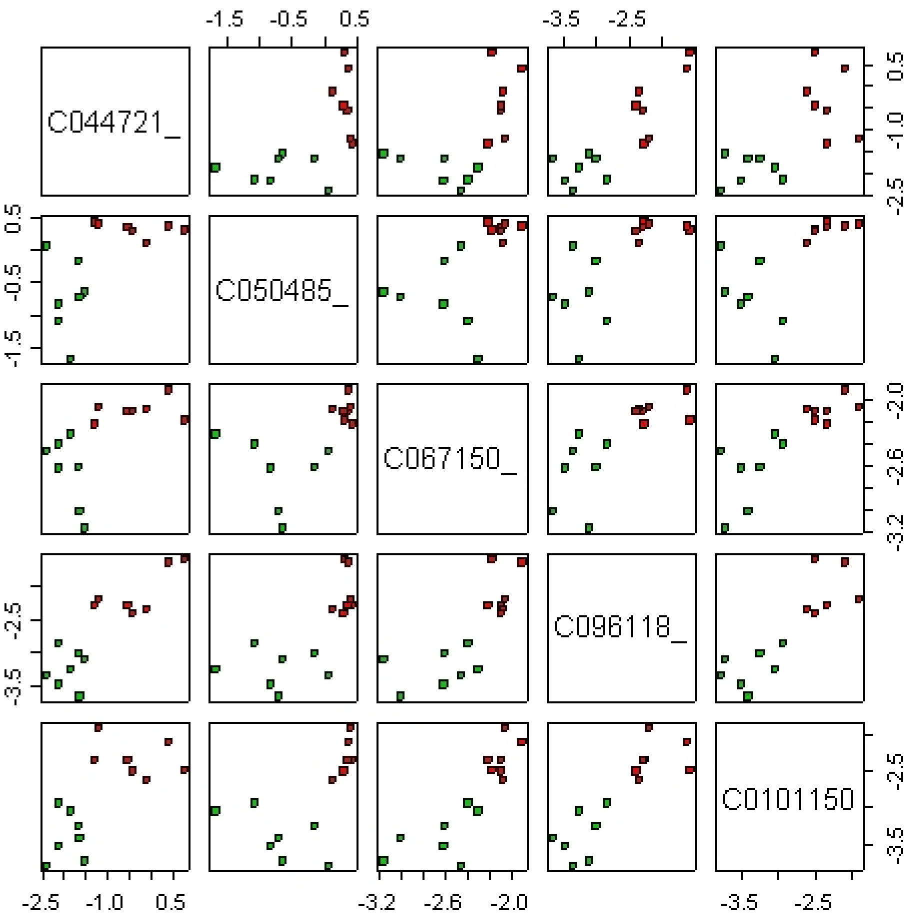

Significant data (p<=0.01)

Significant Proteins (t-test; p<=0.01)

	name	mz	ME7.avg	NORM.avg	t	p
11	C09376_5	9376	2.52	2.99	-3.4	5.9e-03
13	C010718_	10718	2.00	1.05	5.5	2.0e-04
15	C012246_	12246	2.15	1.54	3.6	3.8e-03
19	C014199_	14199	2.61	3.12	-3.8	2.6e-03
28	C022485_	22485	0.73	1.38	-3.5	6.5e-03
36	C044721_	44721	-0.35	-1.92	4.9	1.1e-03
37	C050485_	50485	0.32	-0.72	4.7	2.7e-03
40	C067150_	67150	-2.09	-2.65	4.5	2.8e-03
42	C096118_	96118	-2.11	-3.25	6.8	2.5e-05
43	C0101150	101150	-2.33	-3.38	6.8	2.5e-05

Data for Significant proteins

	C0GROUP	C0GRP_NA	C0Spectr	C09376_5	C010718_	C012246_	C014199_	C022485_	C044721_	C050485_	C067150_	C096118_	C0101150
5	0	ME7S2	B36863	2.2	1.44	1.7	2.7	0.28	-1.32	0.430	-2.2	-2.3	-2.3
6	0	ME7S2	B36865	2.8	2.06	1.9	2.8	0.95	-0.55	0.350	-2.1	-2.3	-2.3
7	0	ME7S2	B36867	2.2	2.00	2.4	2.2	1.01	0.77	0.308	-2.2	-1.6	-2.5
11	0	ME7S2	B36864	2.6	2.04	2.0	2.6	0.87	-0.12	0.117	-2.1	-2.4	-2.6
12	0	ME7S2	B36865	2.9	2.01	2.1	2.8	0.63	-0.44	0.287	-2.1	-2.4	-2.5
13	0	ME7S2	B36866	2.7	2.11	2.4	2.9	0.71	-1.21	0.395	-2.1	-2.2	-1.9
14	0	ME7S2	B36868	2.2	2.33	2.5	2.3	0.67	0.41	0.372	-1.9	-1.6	-2.1
1	1	Normal S2	B36857	2.6	0.81	1.4	3.4	0.64	-2.40	0.065	-2.5	-3.4	-3.8
2	1	Normal S2	B36858	3.2	0.73	1.1	3.0	1.99	-2.14	-1.079	-2.4	-2.9	-2.9
3	1	Normal S2	B36859	2.8	1.48	1.3	2.9	1.37	-1.66	-0.165	-2.6	-3.0	-3.3
4	1	Normal S2	B36860	3.0	1.42	1.8	3.3	1.33	-1.55	-0.641	-3.2	-3.1	-3.7
8	1	Normal S2	B36860	3.1	1.24	1.8	3.4	1.20	-2.15	-0.827	-2.6	-3.5	-3.5
9	1	Normal S2	B36861	3.0	1.16	2.0	3.1	1.35	-1.66	-0.711	-3.0	-3.7	-3.4
10	1	Normal S2	B36862	3.1	0.51	1.3	2.7	1.75	-1.86	-1.670	-2.3	-3.3	-3.0

Boxplot of significant proteins

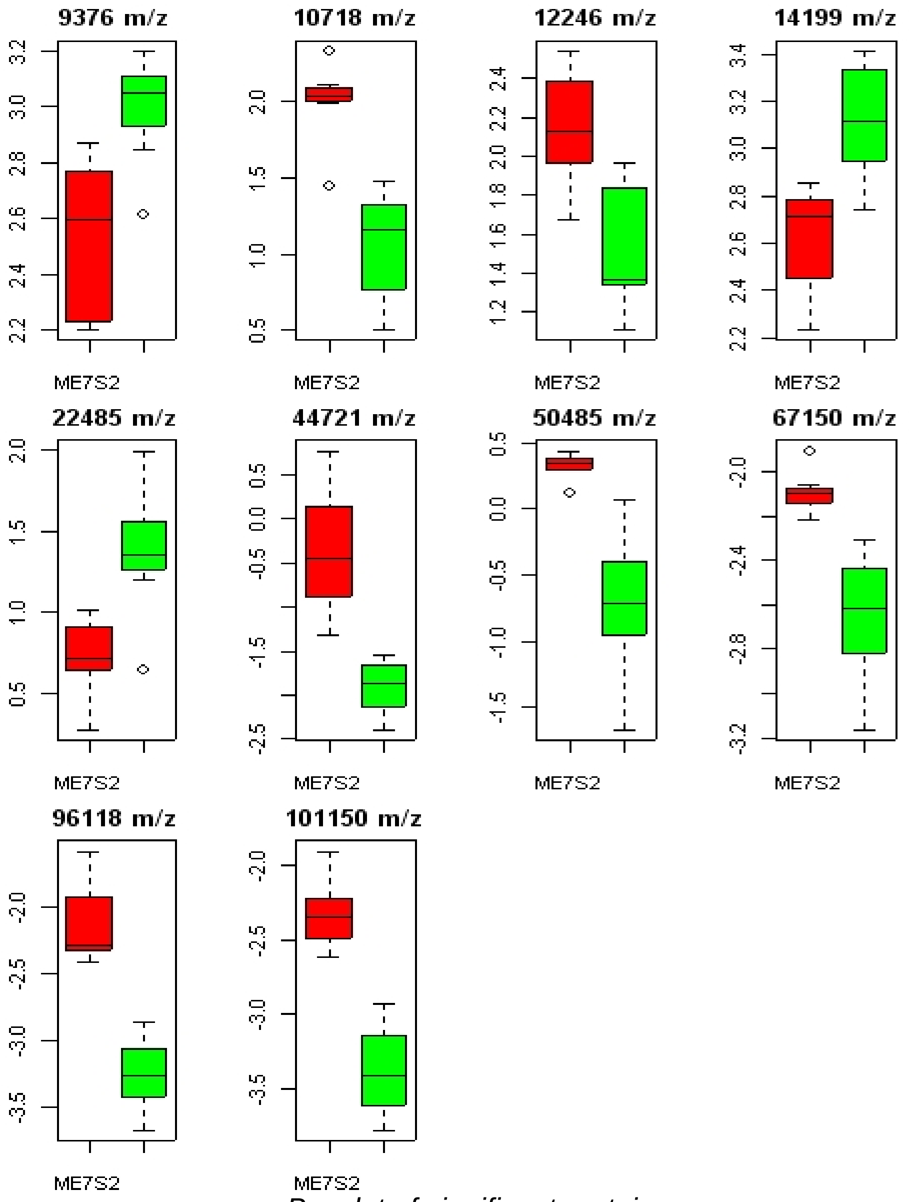

Boxplot of significant proteins

Pairwise Scatterplots of Significant Proteins

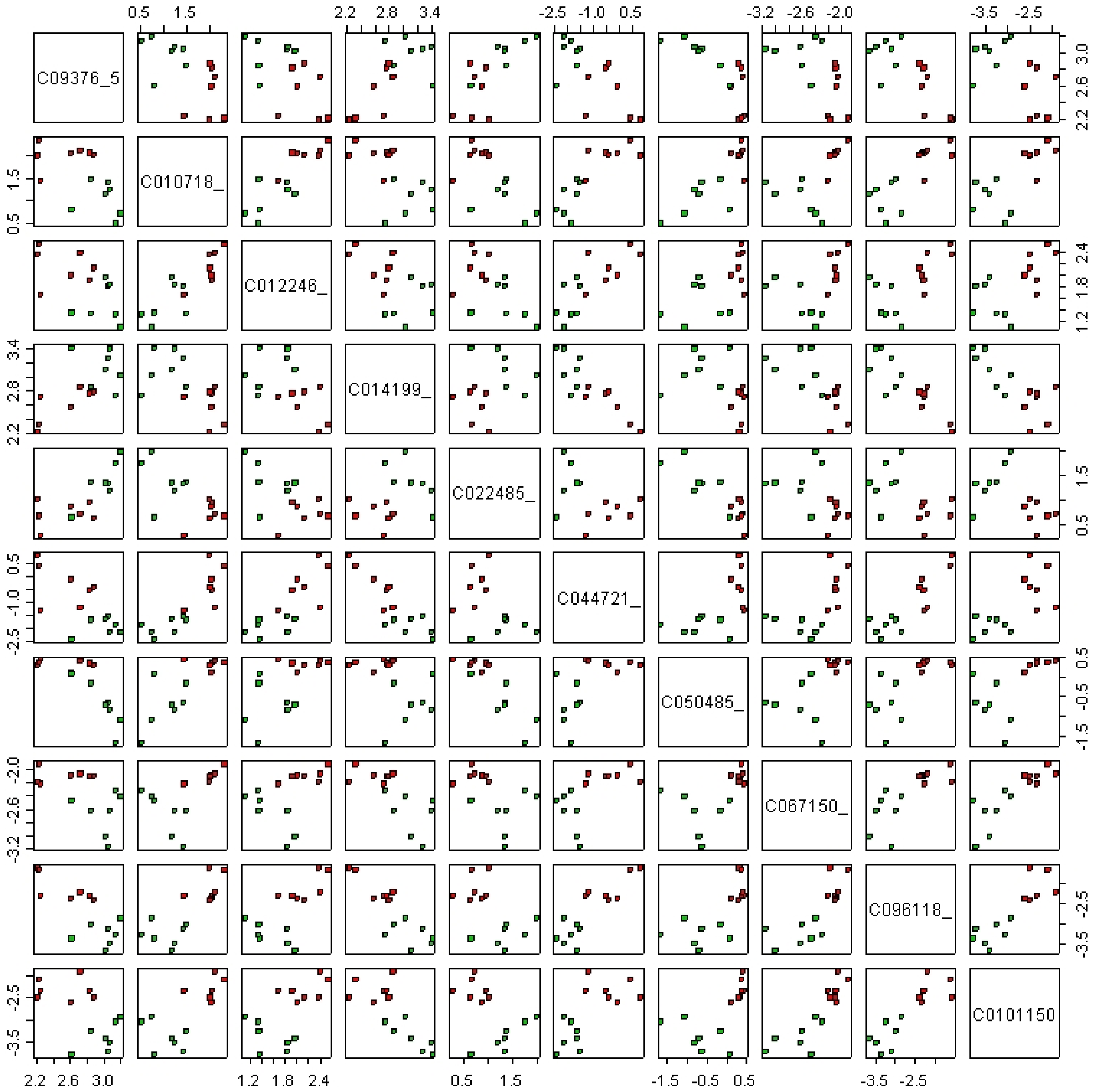

Pairwise Scatterplots of Significant Proteins

Cluster Analysis of samples (Euclidean distance)

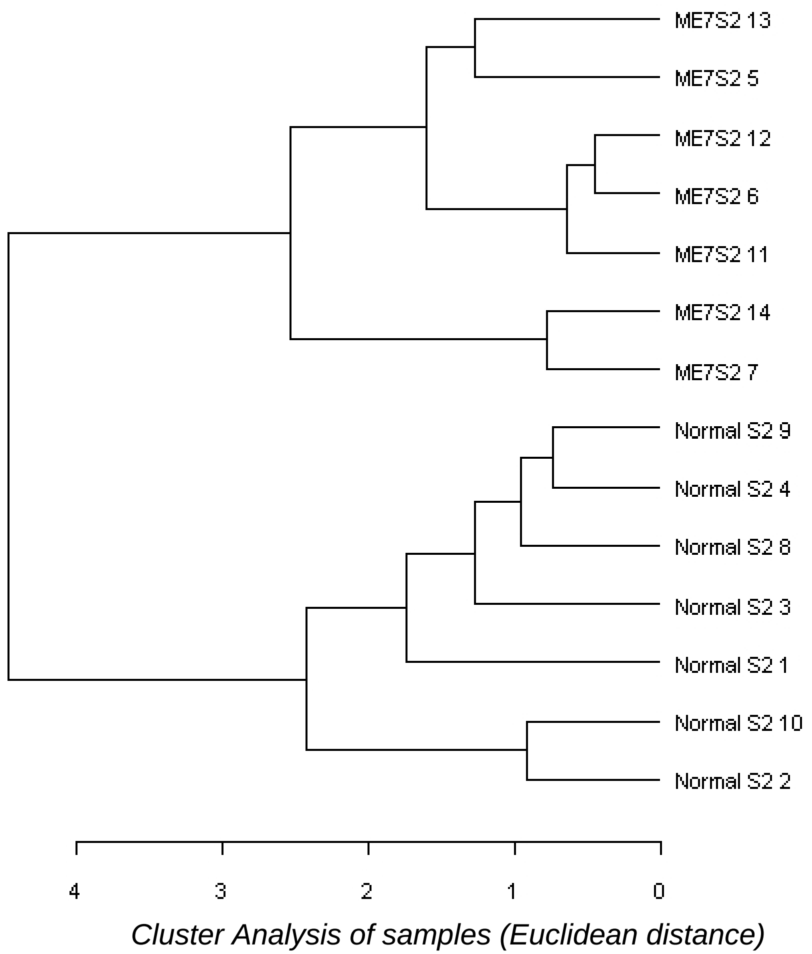

Plot of first three principal components

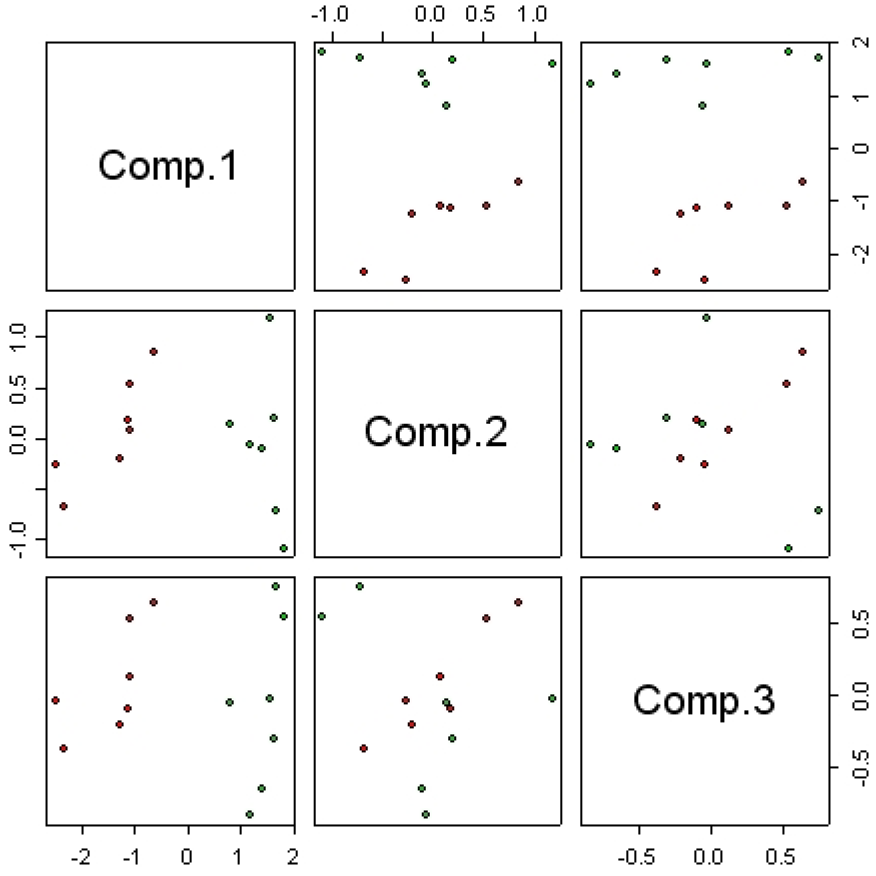

Plot of first three principal components

Scatterplot of linear discriminant function (x-axis)

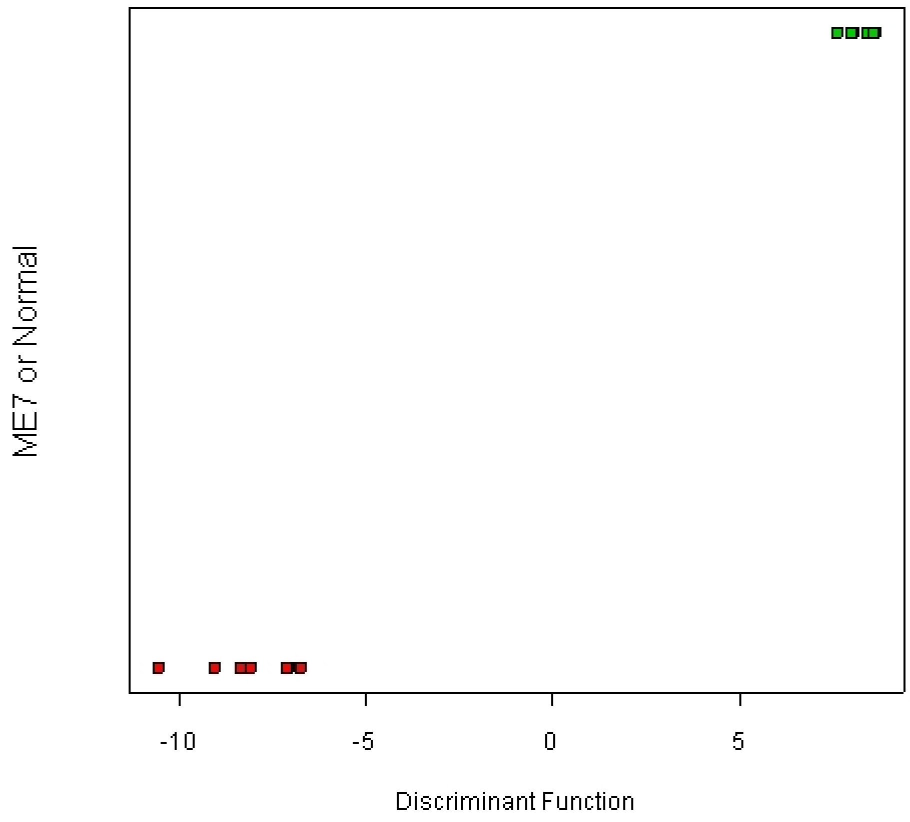

Scatterplot of linear discriminant function (x-axis)

All data

Boxplot of all proteins

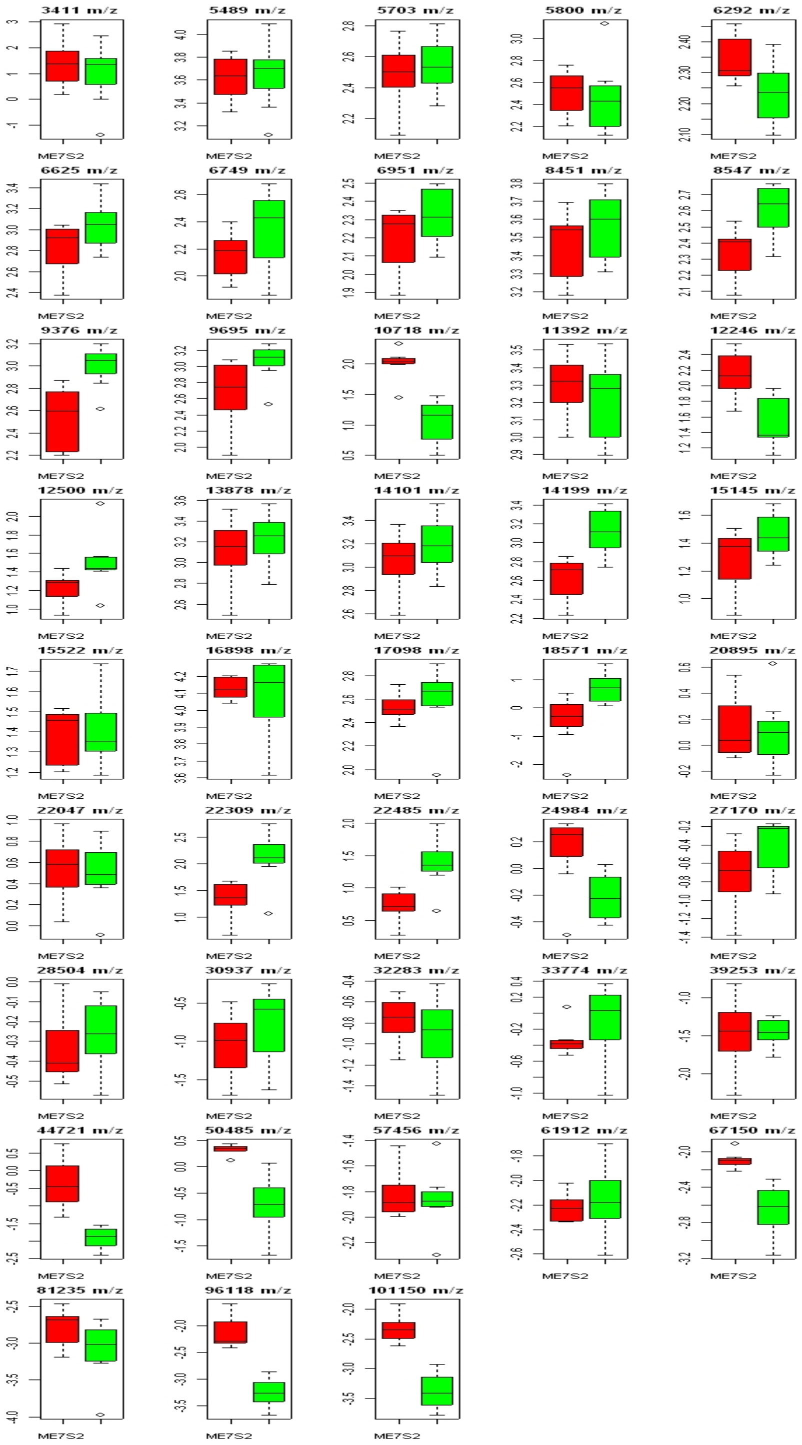
3411 m/z
5489 m/z
5703 m/z
5800 m/z
6292 m/z
6625 m/z
6749 m/z
6951 m/z
8451 m/z
8547 m/z
9376 m/z
9695 m/z
10718 m/z
11392 m/z
12246 m/z
12500 m/z
13878 m/z
14101 m/z
14199 m/z
15145 m/z
15522 m/z
16898 m/z
17098 m/z
18571 m/z
20895 m/z
22047 m/z
22309 m/z
22485 m/z
24984 m/z
27170 m/z
28504 m/z
30937 m/z
32283 m/z
33774 m/z
39253 m/z
44721 m/z
50485 m/z
57456 m/z
61912 m/z
67150 m/z
81235 m/z
96118 m/z
101150 m/z
ME7S2

Boxplot of all proteins

Cluster Analysis of samples (Euclidean distance)

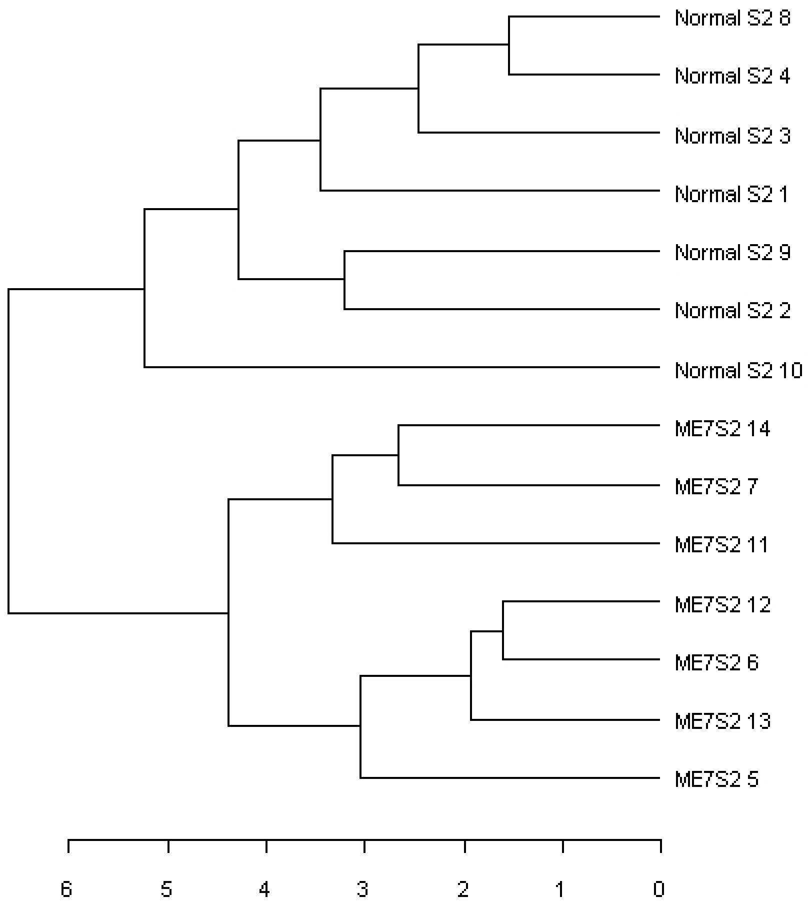

Cluster Analysis of samples (Euclidean distance)

S2 Q10 EP

Proteins showing total separation

	C0GROUP	C0GRP_NA	C0Spectr	C05485_8	C08446_7	C08543_5	C017087_	C033748_	C045959_	C097076_
1	0	ME7S2	B36885	3.1	3.1	2.3	2.3	-0.928	-1.110	-1.86
3	0	ME7S2	B37003	3.3	3.1	2.1	2.6	-0.335	0.708	-0.89
4	0	ME7S2	B37134	3.4	2.9	1.8	2.1	-0.809	0.358	-1.28
8	0	ME7S2	B37004	3.1	3.2	2.0	2.2	-0.220	-0.699	-1.94
9	0	ME7S2	B37020	3.4	3.3	1.9	2.4	-0.414	-0.043	-2.08
11	0	ME7S2	B37283	3.4	3.6	2.2	2.7	-0.166	-0.205	-1.36
12	0	ME7S2	B37285	3.3	3.3	1.9	2.2	-0.298	-0.564	-1.92
2	1	Normal S2	B37002	3.6	3.6	2.4	2.8	0.226	-2.275	-3.59
5	1	Normal S2	B37284	3.4	3.6	2.4	2.9	0.289	-1.241	-3.48
6	1	Normal S2	B37329	3.8	4.0	2.6	2.9	0.062	-2.090	-3.01
7	1	Normal S2	B37501	3.5	3.6	2.4	2.9	0.231	-2.530	-3.85
10	1	Normal S2	B37133	3.7	3.6	2.3	2.9	0.442	-2.185	-3.36
13	1	Normal S2	B37499	3.6	3.7	2.4	2.8	0.318	-1.707	-3.47
14	1	Normal S2	B37500	3.6	3.7	2.5	2.9	0.602	-1.510	-2.97

Boxplot of proteins showing complete separation

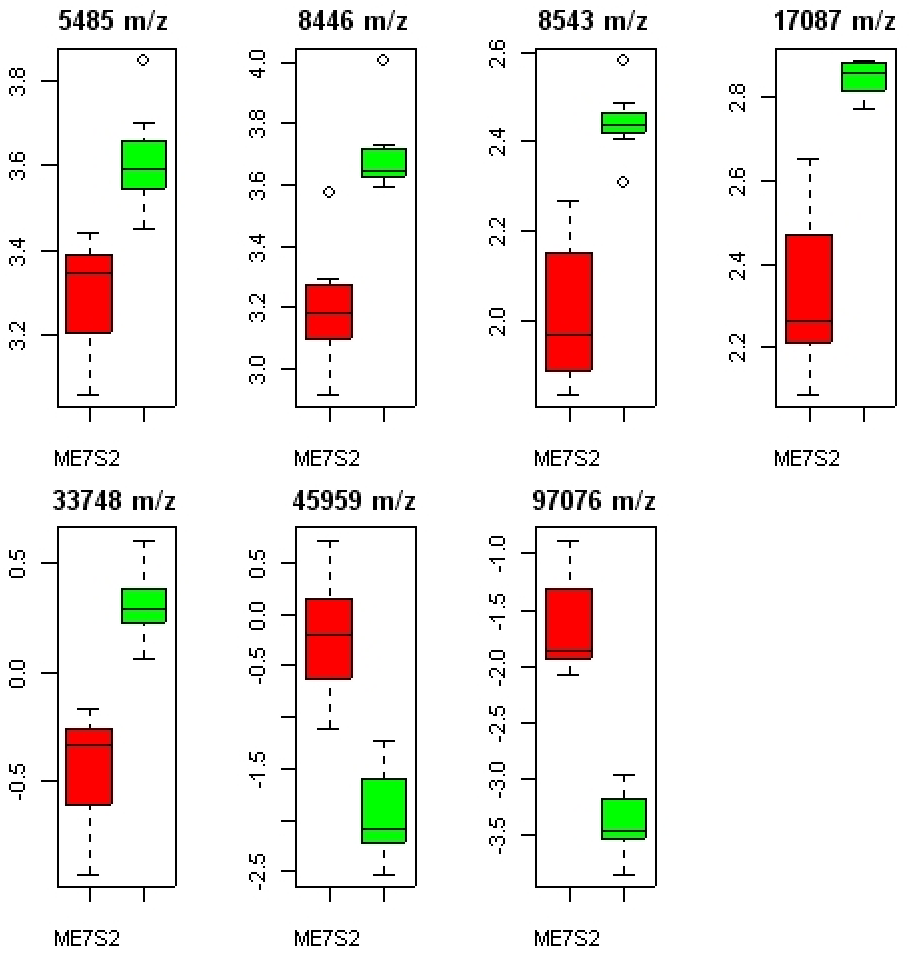

Pairwise Scatterplots of Proteins showing complete separation

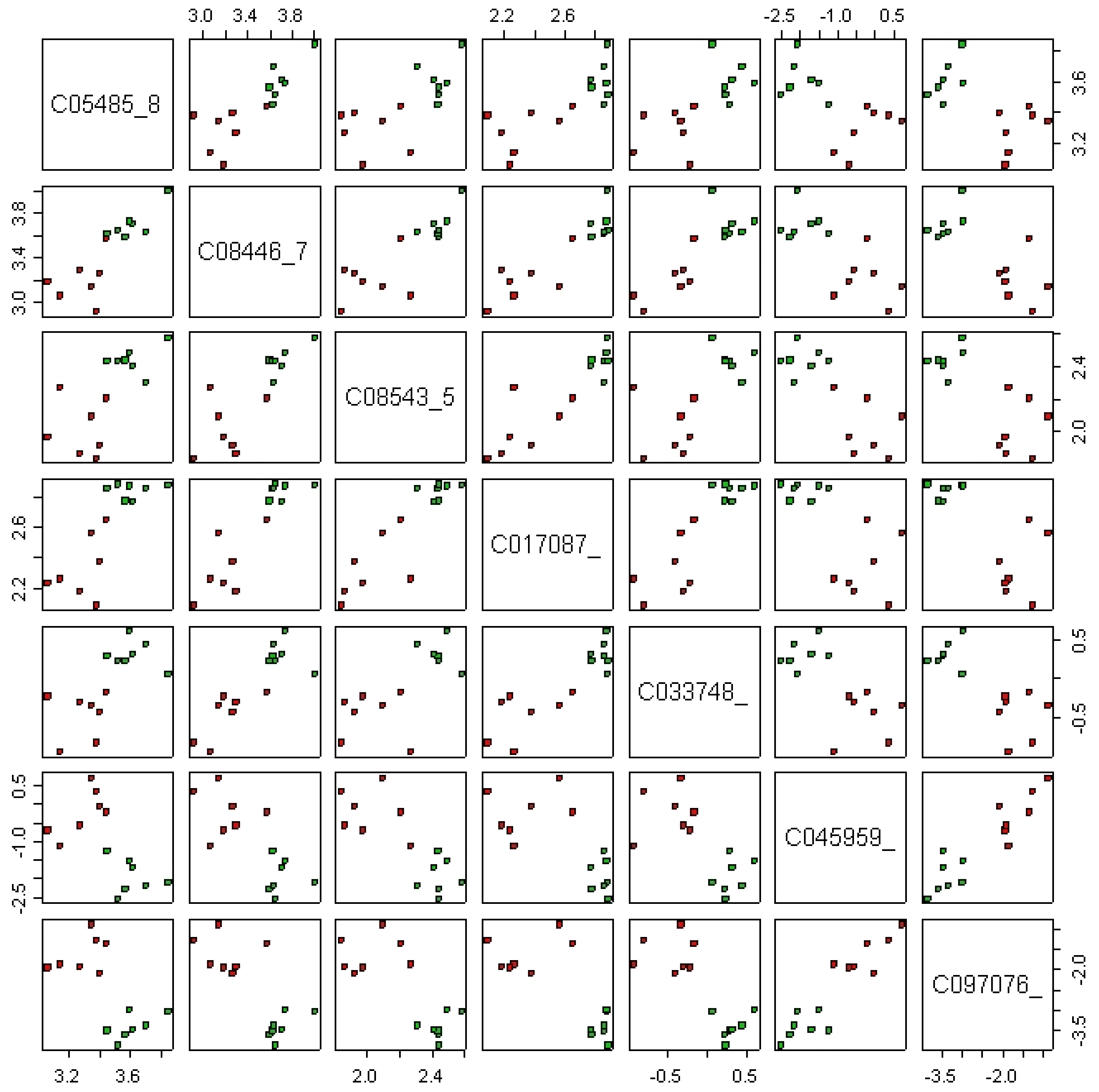
C05485_8
C08446_7
C08543_5
C017087_
C033748_
C045959_
C097076_

Significant data (p<=0.001)

Significant Proteins (t-test; p<=0.001)

	name	mz	ME7.avg	NORM.avg	t	p
2	C05485_8	5485	3.29	3.61	-4.4	8.3e-04
7	C08446_7	8446	3.20	3.70	-5.3	2.9e-04
8	C08543_5	8543	2.02	2.44	-5.9	2.8e-04
9	C09364_1	9364	2.33	2.75	-5.3	2.8e-04
10	C09688_8	9688	2.39	2.90	-4.8	5.9e-04
11	C010726_	10726	2.29	1.43	5.5	1.5e-04
22	C017087_	17087	2.34	2.84	-6.3	4.7e-04
27	C025007_	25007	0.27	-0.16	4.8	4.2e-04
31	C033748_	33748	-0.45	0.31	-5.9	1.8e-04
34	C045959_	45959	-0.22	-1.93	5.8	1.2e-04
38	C066581_	66581	-1.99	-2.64	4.6	5.9e-04
39	C089273_	89273	-2.07	-3.48	5.1	2.7e-04
40	C097076_	97076	-1.62	-3.39	8.6	3.6e-06

Data for Significant proteins

	C0GROUP	C0GRP_NA	C0Spectr	C05485_8	C08446_7	C08543_5	C09364_1	C09688_8	C010726_	C017087_	C025007_	C033748_	C045959_	C066581_	C089273_	C097076_
1	0	ME7S2	B36885	3.1	3.1	2.3	2.7	2.6	2.1	2.3	0.228	-0.928	-1.110	-1.9	-2.4	-1.86
3	0	ME7S2	B37003	3.3	3.1	2.1	2.4	2.4	2.4	2.6	0.499	-0.335	0.708	-1.8	-1.2	-0.89
4	0	ME7S2	B37134	3.4	2.9	1.8	2.3	2.2	2.3	2.1	0.292	-0.809	0.358	-2.1	-1.6	-1.28
8	0	ME7S2	B37004	3.1	3.2	2.0	2.2	2.5	2.6	2.2	0.048	-0.220	-0.699	-1.9	-2.9	-1.94
9	0	ME7S2	B37020	3.4	3.3	1.9	2.4	2.2	2.5	2.4	0.425	-0.414	-0.043	-2.6	-2.2	-2.08
11	0	ME7S2	B37283	3.4	3.6	2.2	2.2	2.5	2.4	2.7	0.299	-0.166	-0.205	-1.9	-2.2	-1.36
12	0	ME7S2	B37285	3.3	3.3	1.9	2.1	2.4	1.8	2.2	0.104	-0.298	-0.564	-1.8	-2.2	-1.92
2	1	Normal S2	B37002	3.6	3.6	2.4	2.9	3.2	1.1	2.8	-0.197	0.226	-2.275	-2.7	-3.4	-3.59
5	1	Normal S2	B37284	3.4	3.6	2.4	2.6	2.5	1.3	2.9	-0.381	0.289	-1.241	-2.9	-4.0	-3.48
6	1	Normal S2	B37329	3.8	4.0	2.6	2.7	3.1	1.2	2.9	-0.365	0.062	-2.090	-2.6	-2.7	-3.01
7	1	Normal S2	B37501	3.5	3.6	2.4	2.9	3.0	1.1	2.9	-0.144	0.231	-2.530	-3.0	-3.4	-3.85
10	1	Normal S2	B37133	3.7	3.6	2.3	2.8	2.9	1.7	2.9	0.067	0.442	-2.185	-2.7	-3.3	-3.36
13	1	Normal S2	B37499	3.6	3.7	2.4	2.6	2.8	1.5	2.8	-0.069	0.318	-1.707	-2.3	-3.4	-3.47
14	1	Normal S2	B37500	3.6	3.7	2.5	2.7	2.8	1.9	2.9	-0.010	0.602	-1.510	-2.3	-4.2	-2.97

Boxplot of significant proteins

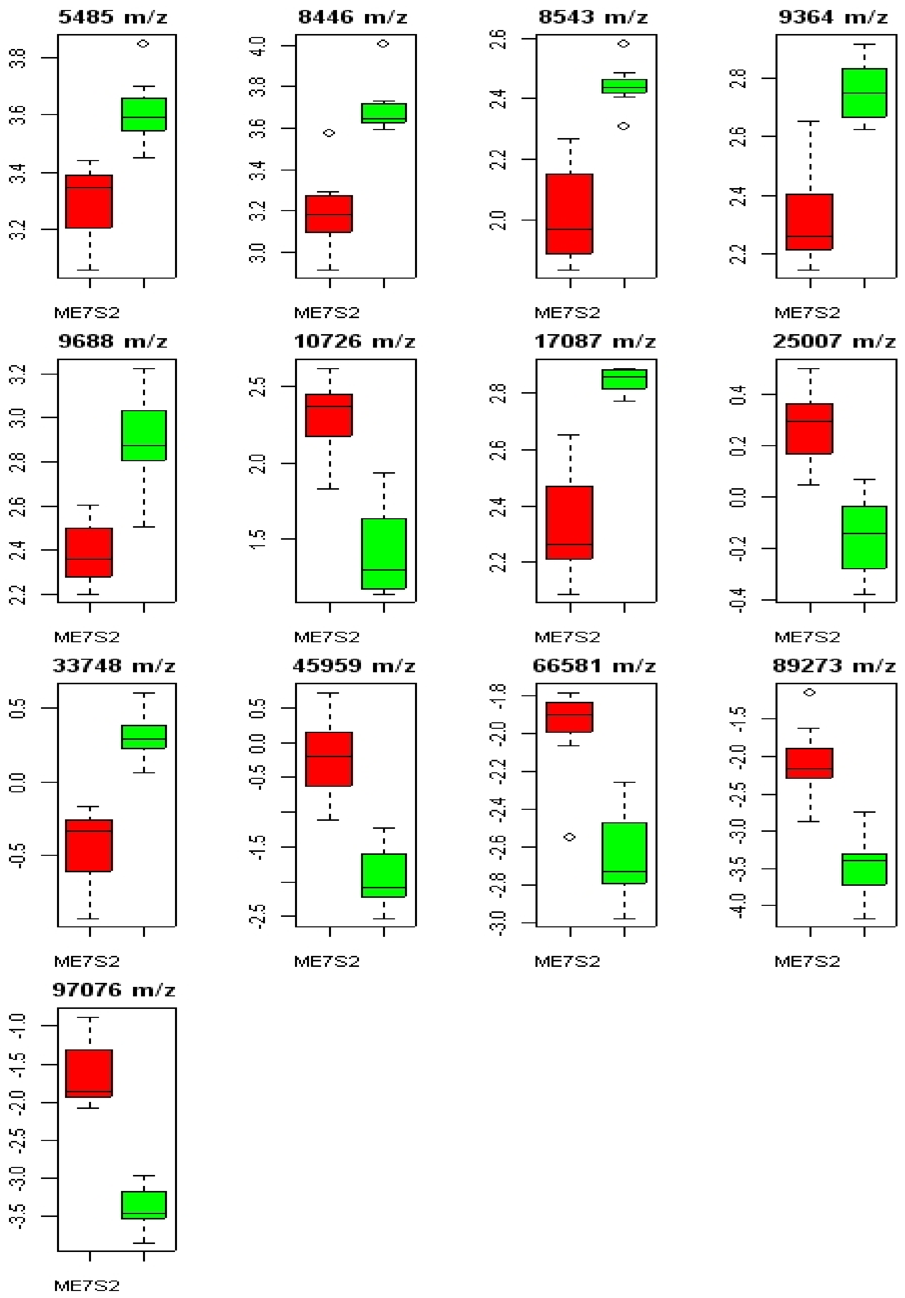

Boxplot of significant proteins

Pairwise Scatterplots of Significant Proteins

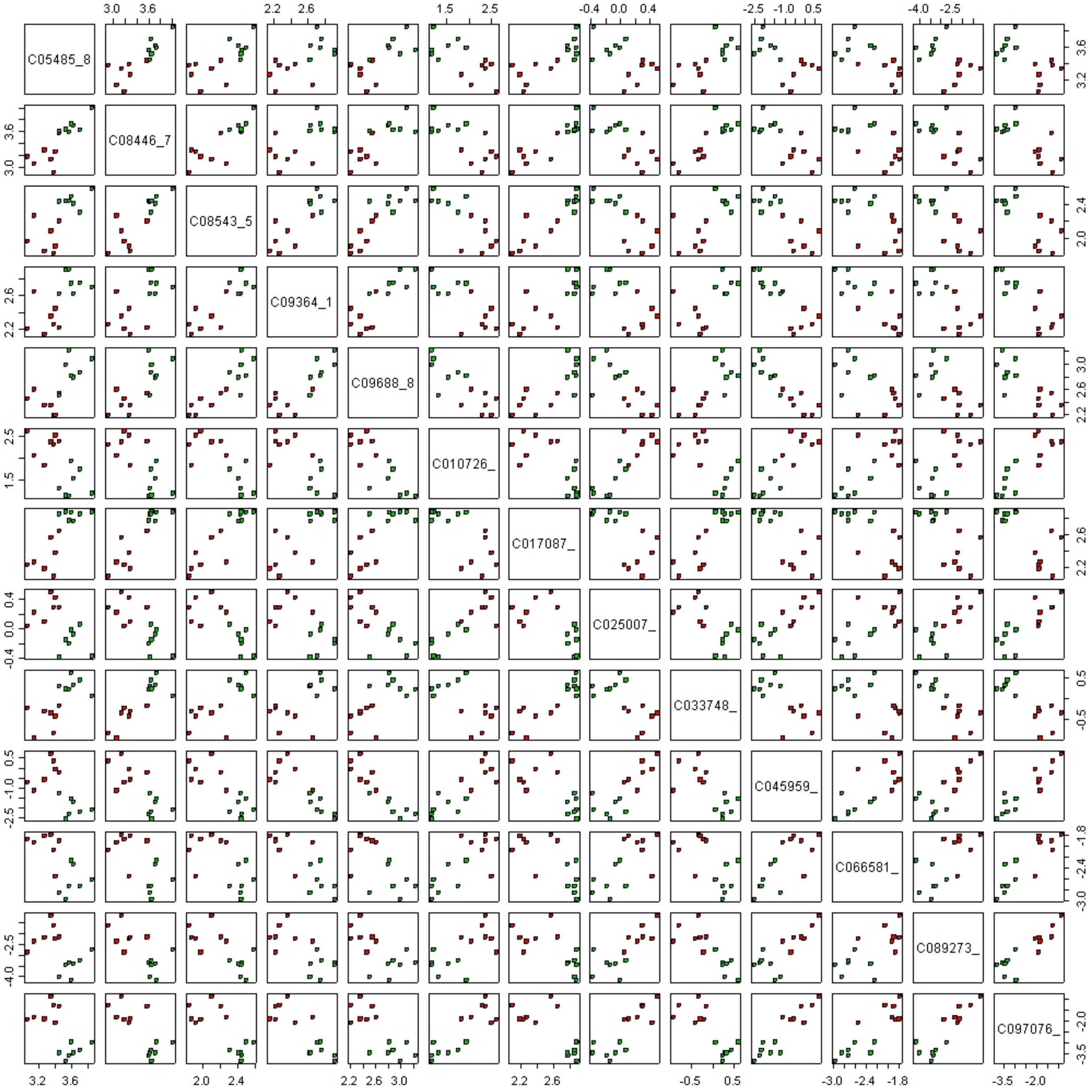

Pairwise Scatterplots of Significant Proteins

Cluster Analysis of samples (Euclidean distance)

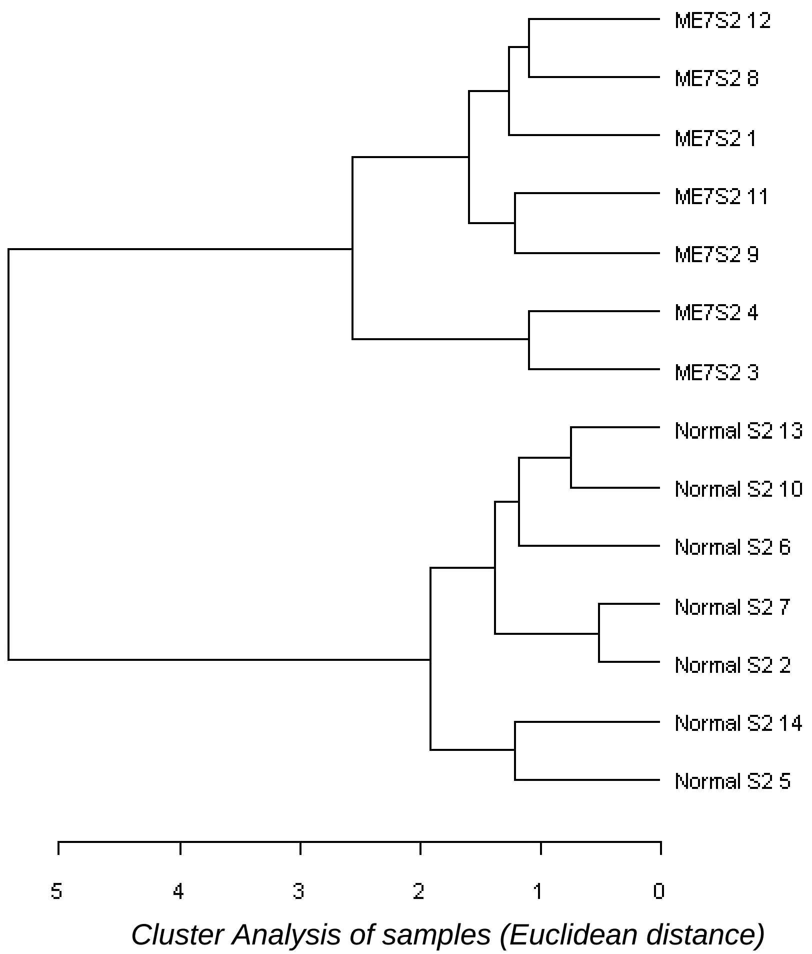

Plot of first three principal components

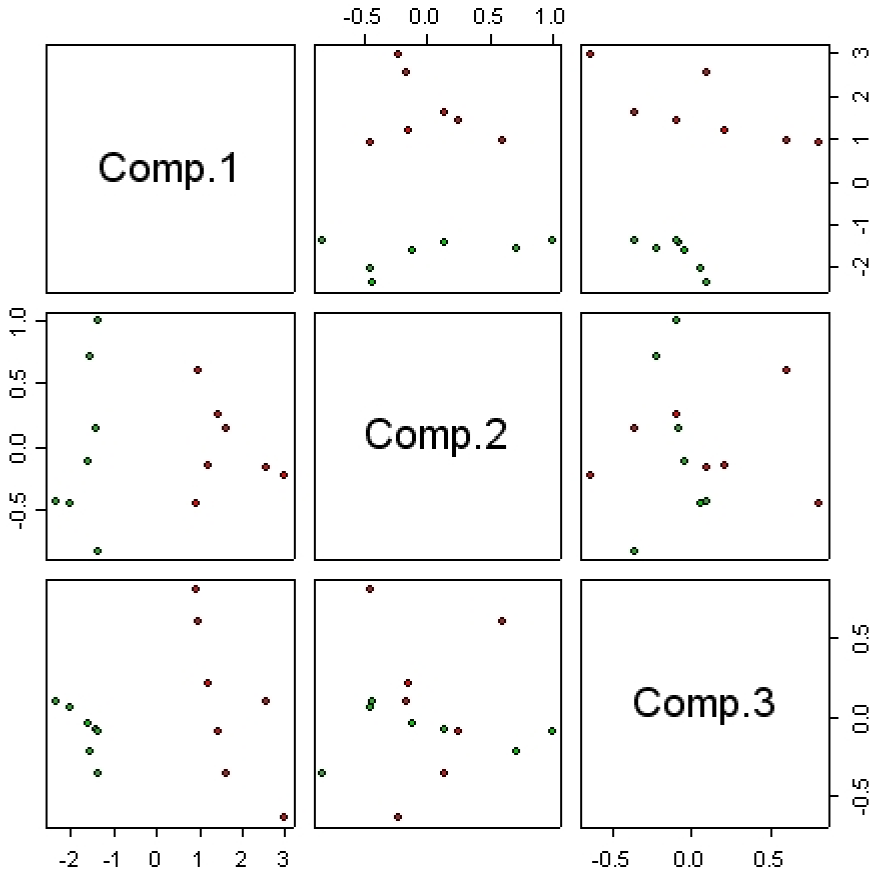

Plot of first three principal components

Scatterplot of linear discriminant function (x-axis)

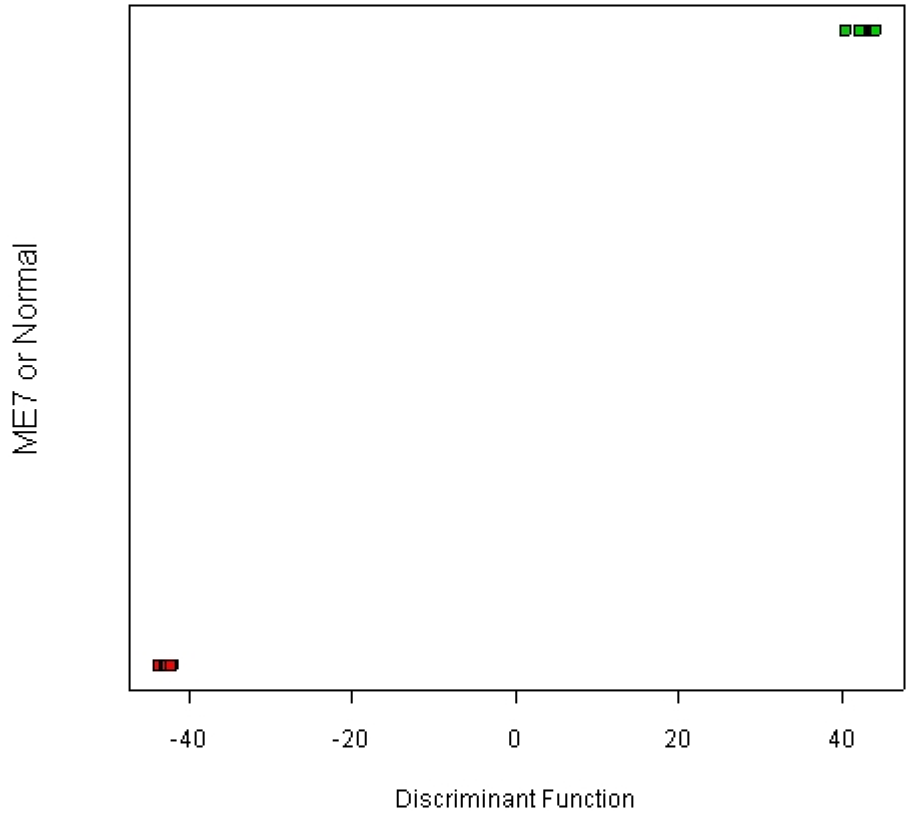

Scatterplot of linear discriminant function (x-axis)

All data

Boxplot of all proteins

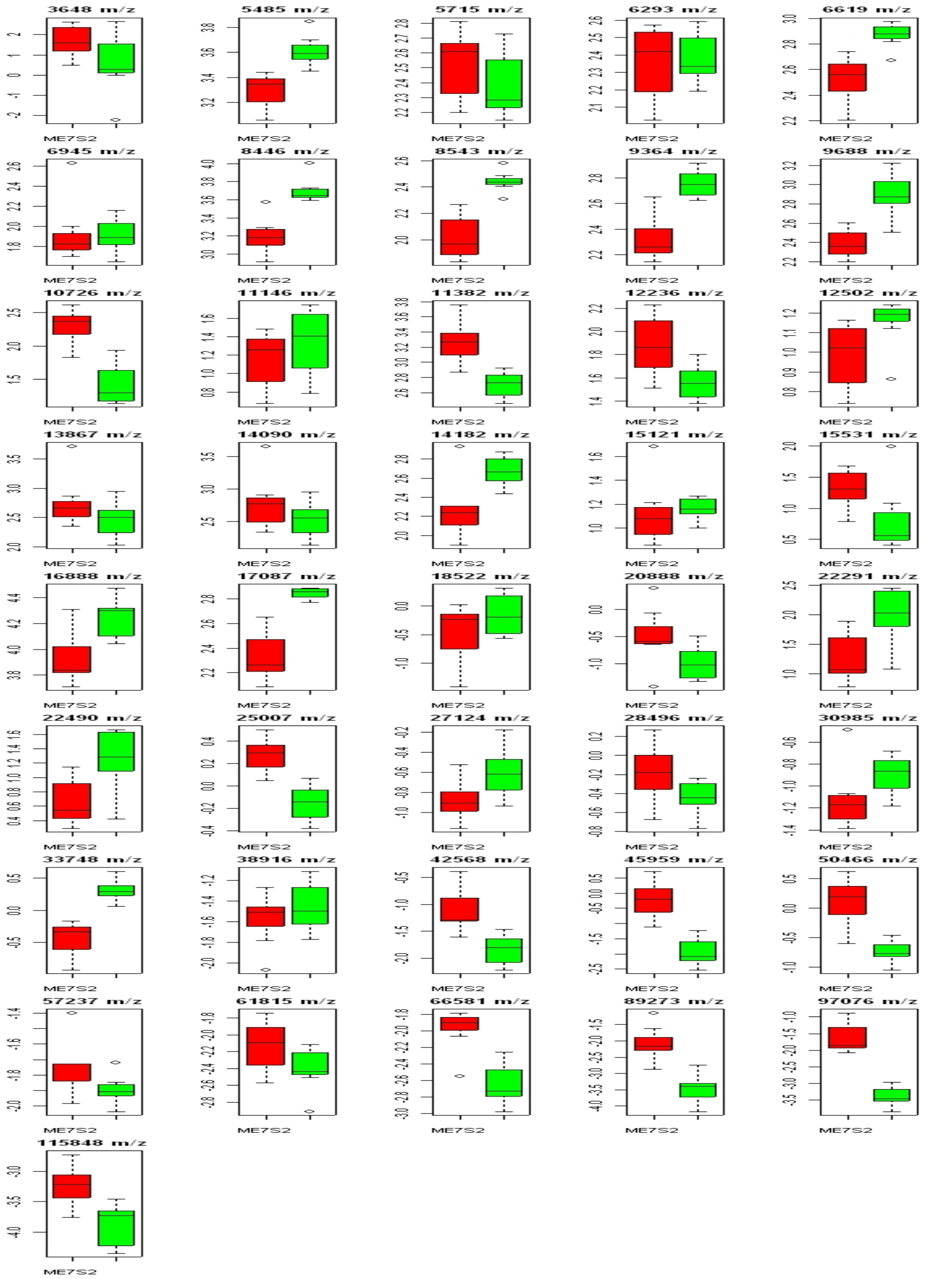

Boxplot of all proteins

Cluster Analysis of samples (Euclidean distance)

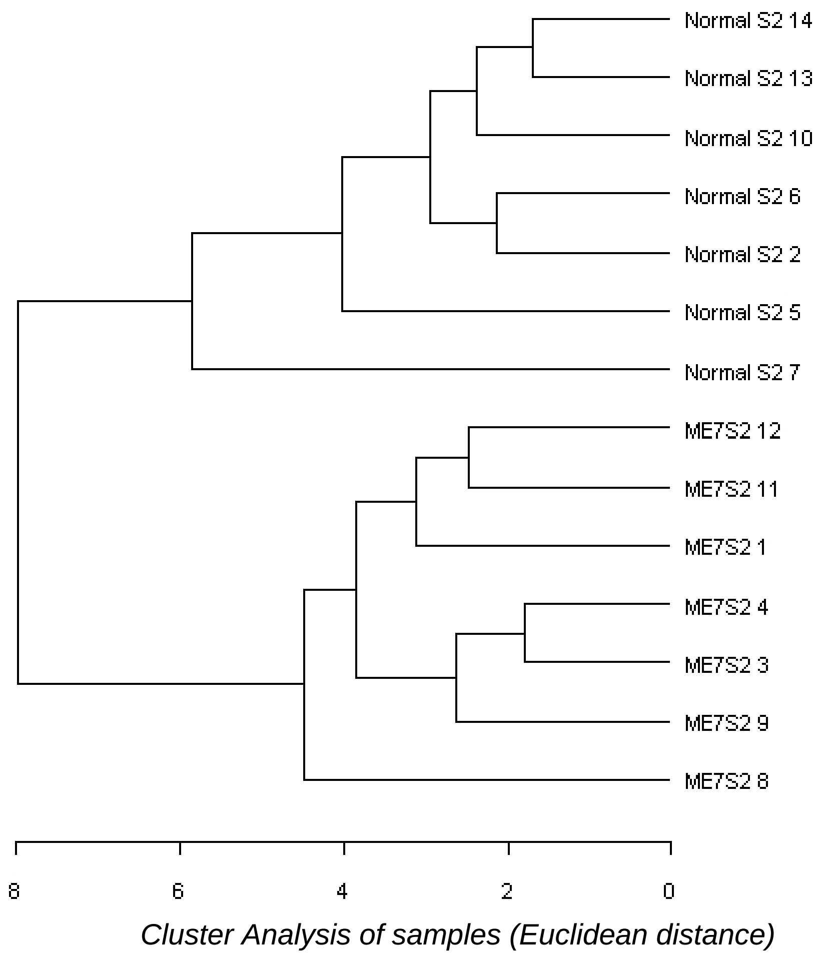

Supplement: Additional file 9 — Full statistical analysis of Q10 supernatant 2 arrays [file 1471-2334-9-188-S9.PDF]
